# ﻿A monograph of the genus *Polylepis* (Rosaceae)

**DOI:** 10.3897/phytokeys.203.83529

**Published:** 2022-08-01

**Authors:** Tatiana Erika Boza Espinoza, Michael Kessler

**Affiliations:** 1 Institute for Nature, Earth and Energy (INTE), Pontificia Universidad Católica del Perú (PUCP), Av. Universitaria 1801, Lima 15088, Peru Pontificia Universidad Católica del Perú (PUCP) Lima Peru; 2 Department of Systematic and Evolutionary Botany, University of Zurich, Zollikerstrasse 107, CH-8008 Zurich, Switzerland University of Zurich Zürich Switzerland

**Keywords:** Andes, morphology, new taxa, taxonomy

## Abstract

We present a monograph of the high Andean tree genus *Polylepis* (Rosaceae), based on a species concept considering morphological, climatic and biogeographic distinctness as indicators of evolutionary independence. In total, we recognize 45 species of *Polylepis*, grouped in five sections. Polylepissect.Sericeae is represented by 15 species in four subsections, P.sect.Reticulatae by seven species, P.sect.Subsericantes by three species, P.sect.Australes by two species and P.sect.Incanaee by three subsections with 18 species. We describe seven new species, one from Colombia (*P.frontinensis*), one from Ecuador (*P.simpsoniae*) and five from Peru (*P.acomayensis*, *P.fjeldsaoi*, *P.occidentalis*, *P.pilosissima* and *P.sacra*). Three species from Peru (*P.albicans*, *P.pallidistigma* and *P.serrata*) are re-instated as valid species. Two taxa from Bolivia (*P.incanoides* and *P.nana*) are elevated from subspecies to species rank. The morphology, habitat, distribution, ecology and conservation status of each species are documented. We also provide an identification key to the species of the genus and general introductions on taxonomic history, morphology, evolution, ecology and conservation.

## ﻿Introduction

*Polylepis* may well be the most emblematic tree genus of the central and northern Andes. It often occurs in an otherwise treeless landscape, forming the highest forests in the Western Hemisphere at elevations of over 4800 m. *Polylepis* forests contain unique biodiversity and provide habitats for a wide range of Andean plants and animals. However, they also provide crucial ecosystem services for the people living in the Andes: clean water, protection against erosion, firewood, fodder and medicinal plants, amongst others. On the other hand, it has been estimated that over 90% of the natural cover by *Polylepis* forests has been lost over millennia of human land use, so that the majority of species are considered to be of conservation concern. For this reason, studies on the physiology, ecology and conservation of the genus *Polylepis* have led to the publication of hundreds of scientific papers and reports and to the establishment of numerous conservation areas and projects to safeguard the last forest remnants.

Against this background, the poor taxonomic knowledge of the genus is troublesome. Although the first monograph of the genus was already published in 1911, to date, there is still no consensus on the number of species and their delimitation, greatly hampering conservation and management of the species and forests. The reasons for the taxonomic chaos in the genus are to be found in the high phenological variability of the individual species, coupled with great similarity between species, extensive hybridization and gene flow between species, polyploidization and, possibly, even apomictic reproduction (asexual seed reproduction). Due to these reasons, standard species concepts are difficult to apply to the genus, so that different authors have taken different approaches, arriving at different species circumscriptions. The last comprehensive monograph of the genus was published in 1979 and, due to the numerous discoveries since then, there was a strong need for a modern taxonomic treatment of the genus.

In this study, we undertook this effort by combining 35 years of field and herbarium experience of the co-author, MK, with the fresh view of PhD student TB (co-author). Together, we have studied all, but three species (*P.longipilosa*, *P.occidentalis*, *P.quadrijuga*) of the genus in the field and have applied a novel species concept for *Polylepis* which is based on a combination of morphological, ecological and biogeographical distinctness. Our species concept is narrower than previous concepts and accounts for the evolutionary independence of geographically disjunct forms that were previously combined in broadly defined species. As a result, we here recognize 45 species (up from 26 in the last global count published in 2006), including seven newly-described species. We consider that this classification more accurately reflects the evolutionary variability of *Polylepis* than previous classifications and will provide a workable background for the conservation and management of *Polylepis* forests and associated biota. Naturally, every new classification must stand the test of practicability and we look forward to this in the future. Groves (2001) nicely outlined this challenge: “*Taxonomy, like other fields of biology (ecology, ethology, physiology, genetics), is a dynamic science. Classifications are not engraved in stone, nor should they be; it is unfortunate that advances in the taxonomic field, unlike those in ecology and other disciplines, often require changing the names we give to species […] but that is the way it must be, and the irritation felt […] will pass quickly. Indeed, new predictions, to be tested in the field, may well emerge from the reclassification.*” Only time will tell if the classification of *Polylepis* that we present here will, indeed, as we hope, lead to such new predictions and increase our understanding of the evolution of high Andean forests and their biodiversity.

## ﻿Materials and methods

### ﻿Species concepts and sectional delimitations

Bitter (1911) used a typological species concept for *Polylepis* in which specimens that were morphologically different to each other were assigned to different species, resulting in a treatment with 33 species. Unfortunately, Bitter had few specimens available and did not know the genus in the field. Consequently, he did not consider the intraspecific morphological variability of the species in his taxonomic study. Based on many more herbarium collections, as well as personal fieldwork, Simpson (1979) had a better idea of the natural variability of the species and used the biological species concept (Mayr 2000) which helped her to reduce the number of species recognized until that time to 15. However, some of the species recognized by her were very variable and, in a subsequent publication (Simpson 1986), she indicated that some of her species would be better treated as “species groups”, although, except for *P.tarapacana* and *P.tomentella*, she did not separate them as different species. Later, Kessler and Schmidt-Lebuhn (2006) used a phylogenetic species concept recognizing at species rank, populations with distinct morphology, biogeography and ecology, even if they show hybridization with other species. Based on this narrower delimitation of species, they increased the number of species to 26.

The species concept used here is the general lineage concept of de Queiroz (1998, 2007), in which a species is a segment of an evolutionary lineage at the population level. To separate species, we have used the species criterion of Davis and Heywood (1973) of phenetic similarity, with discontinuities that may reflect geographical, ecological or reproductive isolation. We applied this concept to morphological traits and also to biogeographical and ecological data. Biogeographically, we considered that allopatric populations of morphologically or ecologically different populations are species because they are reproductively isolated from each other and, hence, evolutionarily independent. Ecologically, we considered that morphologically- and biogeographically distinct populations that also have different climatic niches are species because they show different adaptations to the environment. While ecological data are not commonly used to define species, we consider this to be valuable information, especially since, in most cases, there are different ecological conditions within dispersal distance, showing that the ecological differences are not simply due to the different geographical ranges inhabited by the species. For instance, *P.simpsoniae* and *P.weberbaueri*, which have previously been considered to be conspecific, grow at different elevations (2500–3800 m and 3500–4970 m, respectively), yet in their distribution ranges, there are mountains that would allow them to grow at lower and higher elevations, respectively. The rather narrow species concept applied here allows us to better identify independent evolutionary entities for the conservation and management of *Polylepis* species and forests. We do not recognize infraspecific taxa, such as subspecies or varieties as done by Bitter (1911) and Kessler (1995a), because on present knowledge, we are unable to rank the distinctness of population. For this reason, we consider it easier to simply recognize species.

Species delimitation and identification in *Polylepis* relies on populations instead of single herbarium specimens, because there is phenotypical variability even within a single plant (e.g., leaf size, texture, shape and indumentum). This variability depends on the growing stage of a branch and its exposure (sun or shade). Between individuals of a species, phenotypic variability is even more pronounced depending on genetic background, as well as growing and microhabitat conditions, with individuals from sheltered and humid sites having larger leaves with more leaflets, less dense hair cover and longer inflorescences with more flowers.

We also, for the first time, provide a formal sectional and subsectional infrageneric classification of the genus to facilitate communication. This classification is based on morphological differentiation supported by climatic niches and ploidy levels. In this sectional classification, we clustered species, based on morphological similarity (number of lateral leaflet pairs, indument type of the lower leaflet surfaces, stipule sheaths and/or fruits, leaflet apex shape and fruit shape). This morphologically based separation is broadly supported by biogeography (with most species of a section or subsection replacing each other geographically), similarity in climatic niches and similarity in ploidy levels. In the two larger, more variable sections, we also applied a subsectional classification to group similar species. The orthography of sectional and subsectional names has been designated in accordance with the ‘Shenzhen Code’ (Turland et al. 2018).

The sections defined by us largely correspond to the informal groups as outlined by Simpson (1979, 1986) which are, at least, partly upheld by molecular analyses (see Taxonomic History). However, *P.subsericans* was placed by Simpson (1979) in her *sericea* group, although she mentioned that it might be shown to be in an intermediate position between the *sericea* group and the *incana* complex. Here, we place it in a separate section Subsericantes. Additionally, Simpson (1979) included *P.hieronymi* within the *sericea* group and we are placing it in section Reticulatae, based on morphological similarity with the species in this section.

### ﻿Morphological analyses

Our study is based primarily on the examination of the external morphology of herbarium specimens, supplemented with observation of rehydrated material, photographs and living plants in the field. Field studies by TB were conducted in Peru (Ancash, Arequipa, Ayacucho, Cajamarca, Cerro de Pasco, Cusco, Huancavelica, Junín, Lima, Moquegua, Puno and Tacna) and Ecuador (Azuay, Chimborazo, Loja, Napo and Pichincha). MK has studied *Polylepis* in Venezuela (Mérida), Colombia (Antioquia), Ecuador (Azuay, Napo, Pichincha), Peru (Ancash, Arequipa, Cuzco, Lima and Puno), Bolivia (Chuquisaca, Cochabamba, La Paz, Oruro, Potosí, Tarija) and Argentina (Córdoba, Jujuy, Salta, Tucumán). Together, we have studied all, but three species of *Polylepis* in the field. Voucher specimens were collected according to standard herbarium techniques. Photographs of plants and associated notes were taken in the field.

All the herbarium specimens representing each species were carefully observed and those spanning the morphological variation and geographical distribution range were chosen for measurements. Characters were measured from corresponding positions on mature, reproductive plants in order to minimize variation due to developmental differences. We have chosen characters for measurements, based in part on those that have been previously used to differentiate species within the genus *Polylepis* (Bitter 1911; Simpson 1979; Romoleroux 1996; Kessler 1995b; Mendoza and Cano 2012). We examined more than 1400 specimens (including type material) from the Herbaria AAU, COL, CUZ, F, GOET, HUA, LOJA, MEDEL, MERF, MO, NY, QCA, US, USM, VEN and Z/ZT and the material available on Jstor (https://plants.jstor.org/) and other online Herbaria (COL, F, NY, US). The terminology used for describing the morphological characteristics of the species was based on Simpson (1979), Hickey and King (2000) and Stearn (2004).

### ﻿Geographical distributions

We combined occurrence records, based on herbarium specimens with field locations and observations from previous studies from multiple sources into a comprehensive, relational database. This database comprises approximately 3,250 quality-checked records of all species of *Polylepis*. All coordinates were cleansed and georeferenced if needed. We created the distribution maps using QGIS 2.18.14.

### ﻿Environmental data analyses

We extracted Mean Annual Precipitation (MAP) and Mean Annual Temperature (MAT) as climatic variables from the global climatic model CHELSA version 1.2 (Karger et al. 2017) working with data points from the record database (see above). We used R version 3.0.5 to analyze the climatic data applying the R packages “devtools” and “easyGgplot” to determine and visualize the climatic differences between species. To illustrate the environmental preferences of *Polylepis* species, we used the function boxplot. We ran ANOVAs to analyze the variance on the climatic variables among the *Polylepis* species to define their climatic niche differences, followed by Tukey HSD tests to determine the significant differences among the *Polylepis* species.

### ﻿Conservation status

We based our conservation assessment on the International Union for Conservation of Nature guidelines (IUCN 2015, see <http://www.iucnredlist.org>). A full assessment following IUCN criteria gives a comprehensive evaluation of extinction risk, based on population size reduction (Criterion A), geographic range (Criterion B), population size (Criteria C and D) and qualitative estimates (Criterion E). We used the package ‘ConR’ (Dauby 2018) to estimate the geographical range parameters for a preliminary assessment of the conservation status following Criterion B. Extinction risks were assessed globally.

## ﻿Taxonomic history

The genus *Polylepis* was described by Ruiz and Pavón (1794). The good original generic description and the large number of diagnostic features of the genus (see Morphology) meant that no generic synonyms were subsequently described. Except for *Acaenaochreata* by Weddell (1855), which is listed here as *Polylepisochreata*, all species were correctly classified in the genus *Polylepis* by their describers. This accuracy of the delimitation of the genus contrasts sharply with the highly complicated taxonomic situation within the genus.

Along with the circumscription of the genus, Ruiz and Pavón (1794) described the first species, *Polylepisracemosa*. Subsequently, Humboldt, Bonpland and Kunth (1824) described three other species: *P.incana*, *P.lanuginosa* and *P.villosa*; the latter was considered synonymous with *P.racemosa* by Simpson (1979), as is the case here. Weddell (1855, 1857) described three other species: *P.tomentella*, *P.sericea* and *Acaenaochreata*, as well as two varieties within *P.lanuginosa*, of which var.microphylla is here recognized as an independent species (*P.microphylla*). *Polylepistarapacana* was described by Philippi (1891). Subsequently, Hieronymus (1895, 1896), based on collections by Lehmann and Stübel, described five other species: *P.besseri*, *P.lehmannii* (= *P.lanuginosa*), *P.pauta*, *P.reticulata*, and *P.stuebelii* (= *P.pauta*). In 1898, O. Kuntze published the description of two new varieties within *P.racemosa* (*tomentosa* and *lanata*) from Bolivia, of which the latter is now treated at species level. Pilger (1906) described five additional species, based on the material collected by Weberbauer in Peru and Bolivia: *P.albicans*, *P.hieronymi*, *P.multijuga*, *P.serrata* and *P.weberbaueri*. Thus, at the beginning of the 20^th^ century, 18 species of *Polylepis* and a few subspecies and varieties had been described.

Bitter (1911) published the first revision of *Polylepis*. He recognized all the species described so far, raised two subspecies and varieties to species rank and described 13 other species, nine subspecies and 18 varieties, so that he recognized a total of 33 species. Unfortunately, Bitter did not consider the variability of individual populations and adhered to a typological species concept, so that many of his taxa were based on only one herbarium specimen. The result was a completely confusing taxonomic classification of the genus. Only six species newly described or recognized by Bitter are recognized as valid here: *P.australis*, *P.crista-galli*, *P.pallidistigma*, *P.quadrijuga*, *P.rugulosa* and *P.triacontandra*. Subsequently, Bitter (1913) described another variety of *P.australis*, while other authors described five further *Polylepis* species, although only one of these is still recognized as accepted: Benoist (1934)*P.subintegra* (= *P.ochreata*), Macbride (1934)*P.subsericans* and Cuatrecasas (1941, 1942) *P.boyacensis* (= *P.quadrijuga*), *P.cocuyensis* (= *P.quadrijuga*) and *P.quindiensis* (= *P.sericea*).

In the first modern revision of the genus *Polylepis*, Simpson (1979) brought order into this chaos. She reduced the number of species to 15 broadly delimited species of which only one (*P.pepei*) was described as new; infraspecific taxa were not recognized. Simpson showed a tendency to expand the species and some of the species as circumscribed by her include notable variability. Accordingly, not much later, several authors separated some of Simpson’s species into different species, based on morphology and biogeography. These include the separation of *P.tarapacana* from *P.tomentella* by Simpson (1986) herself, of *P.rugulosa* from *P.besseri* by Kessler (1995c) and of *P.microphylla* from *P.weberbaueri* by Romoleroux (1996). Kessler (1995b), taking into account the variability with *P.besseri* and *P.racemosa* as defined by Simpson (1979), but not wanting to digress too far from Simpson’s (1979) treatment, recognized three subspecies within each of them. Later, all of these were raised to species level by Kessler and Schmidt-Lebuhn (2006). In addition, after Simpson’s treatment, several new species were described, including *P.neglecta* (Kessler 1995b), *P.canoi* (Mendoza 2005) and *P.pacensis* (Kessler and Schmidt-Lebuhn 2006), so that, in 2006, the latter authors recognized 26 species. Since then, *P.rodolfovasquezii* was described by Valenzuela and Villalba (2015).

Accordingly, prior to the present study, there were 88 names available in *Polylepis*, of which 27 were recognized as valid at species level. In our study, we consistently applied a narrow species concept (see Material and Methods), re-instating four species to species level and describing 11 species as new. Seven of these new species are described here, while four have been described in collaboration with colleagues, namely *P.argentea* (Boza Espinoza et al. 2019) and *P.humboldtii*, *P.longipilosa* and *P.loxensis* (Boza Espinoza et al. 2020a).

### ﻿Infrageneric classification

In his revision of the genus, Bitter (1911), established the two sections *Dendracaena* and *Gymnopodae*. He also recognized 11 informal groups, but he never designated if he created these as subsections or series. In the classification presented by Simpson (1979, 1986), three informal and apparently natural species groups were defined, based on morphological similarity and ecological specialization rather than a phylogenetic concept. These informal groups agreed with Bitter’s sections, except that his section Gymnopodae that was split into two. All species under Bitter’s section Dencracaena were placed in the *sericea* group, whereas section Gymnopodae was divided into the *reticulata* group and the *incana* complex. Simpson’s informal classification has a certain level of agreement with the AFLP phylogeny presented by Schmidt-Lebuhn et al. (2006a) which provided evidence that the *sericea* group represents a paraphyletic grade that subtends the other two groups (*reticulata* group and *incana* group) which are each considered to be monophyletic.

The infrageneric classification adopted here includes sections partly corresponding to the three informal and meaningful groups previously defined, based on morphology and supported by climatic niches and ploidy levels. We propose the five sections *Sericeae*, *Reticulatae*, *Subsericantes*, *Australes* and *Incanaee* (Table 1). Section Sericeae includes species with usually sericeous lower leaflet surfaces and/or stipule sheaths, usually with many pairs of lateral leaflets and fruits with variable numbers of spines. Within this section, we recognized four subsections: *Lanuginosae*, *Pauta*, *Sericeae* and *Pepea*. Species in section Reticulatae have relatively few lateral leaflets pairs, rugose or shiny upper leaflet surfaces and emarginate leaflet apices. Section Subsericantes includes three species with characters intermediate between those of sections *Sericeae* and *Incanaee*, with only one pair of lateral leaflets, pilose or strigose hairs on the leaflets and fruits with 3–4 irregular flattened ridges with a series of spines. Section Australes includes two species with numerous, largely glabrous leaflets and very distinctive winged fruits. Finally, species in section Incanaee usually have few lateral leaflet pairs (often only one), frequently glabrous upper leaflet surfaces and fruits with variable number of flattened ridges with a series of spines. Within this section, we recognize three subsections: *Racemosae*, *Besseria* and *Incanaee*. We refrain from using Bitter’s (1911) sectional names because his classification differs in important aspects from ours and we consider that keeping distinct names for different classifications will avoid confusion.

**Table 1.** Alignment of the species of *Polylepis* according to the infrageneric classifications of Bitter (1911), Simpson (1979) and the present study.

Species	Bitter (1911)		Simpson (1979)	This study	
	Section	Group	Group	Section	Subsection
*P.lanuginosa*	*Dendracaena*	*unnamed / Latifoliatae*	*Sericeae*	*Sericeae*	*Lanuginosae*
*P.multijuga*		*Plurijugae*			
*P.longipilosa*		—			*Pauta*
*P.pauta*		*Annulatipilosae*			
*P.serrata*		*Plurijugae*			
*P.albicans*		*unnamed*			*Sericeae*
*P.argentea*		—			
*P.canoi*		—			
*P.frontinensis*		—			
*P.humboldtii*		—			
*P.loxensis*		—			
*P.ochreata*		*Annulatipilosae*			
*P.sericea*					
*P.pepei*		—			*Pepea*
*P.rodolfo-vasquezii*		—			
*P.hieronymi*		*Subtustomentosae*		*Reticulatae*	
*P.microphylla*		*unnamed*	*Reticulatae*		
*P.occidentalis*		—			
*P.quadrijuga*		*Supranitidae*			
*P.reticulata*					
*P.simpsoniae*					
*P.weberbaueri*					
*P.australis*		unnamed*	*Incanaee*	*Australes*	
*P.neglecta*	*Gymnopodae*	—			
*P.flavipila*		—		*Subsericantes*	
*P.pilosissima*		—			
*P.subsericans*		—	*Sericeae / Incanaee*		
*P.acomayensis*		—	*Incanaee*	*Incanaee*	*Racemosae*
*P.incarum*		—			
*P.lanata*		—			
*P.pacensis*		—			
*P.racemosa*		unnamed*			
*P.sacra*		—			
*P.triacontandra*		*Paucijugae*			
*P.besseri*		*unnamed*			*Besseria*
*P.crista-galli*		*Paucijugae*			
*P.pallidistigma*					
*P.rugulosa*					
*P.subtusalbida*		—			
*P.fjeldsaoi*		—			*Incanaee*
*P.incana*		*Paucijugae*			
*P.incanoides*		—			
*P.nana*		—			
*P.tarapacana*		*Paucijugae*			
*P.tomentella*					

* Same unnamed group.

## ﻿Morphology

### ﻿Growth habit

**Figure 1.**
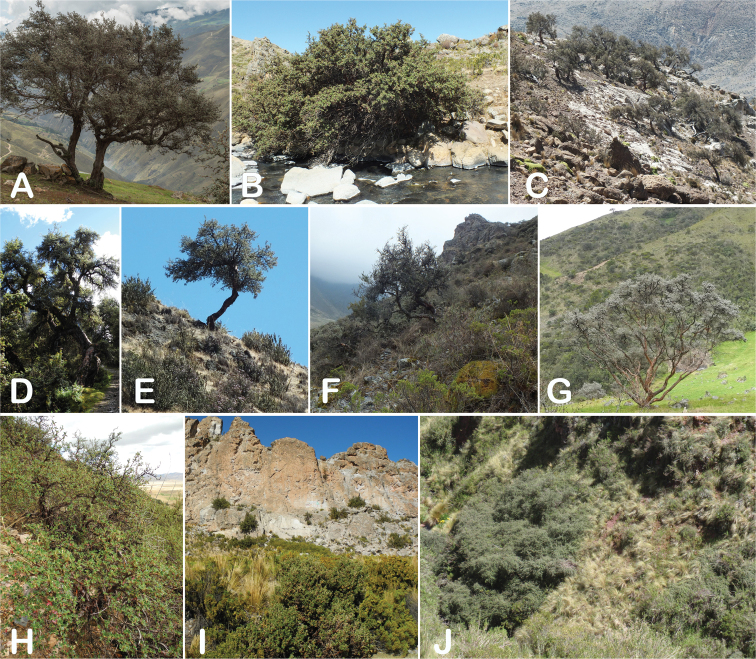
Growth habit of *Polylepis* species: tree growth form: **A***P.microphylla*, Chimborazo, Ecuador **B***P.fjeldsaoi*, Lucanas, Peru **C***P.rugulosa*, Moquegua, Perú **D***P.sacra*, Mantanay, Cusco, Peru **E***P.acomayensis*, Paruro, Cusco, Peru **F***P.pilosissima*, Lima, Perú **G***P.simpsoniae*, Cajas, Azuay, Ecuador; shrubby growth form **H***P.pallidistigma*, Azángaro, Puno, Peru **I***P.tarapacana*, Santa Rosa, Puno, Peru **J***P.microphylla*, Chacan, Cusco, Peru. Photographs **A** E. Bastidas **B, C, E** E.G. Urquiaga F **D, F–I** T.E. Boza E.

All species of *Polylepis* are woody plants growing as trees, multi-stemmed trees or shrubs (Fig. 1). As a result of their peculiar branching pattern, young plants of all species display shrub-like growth in the first few years. Adult height is usually between 1 m and 20 m, but trees of up to 32 m have been measured (Hertel and Wesche 2008). Simpson (1979) claimed that *P.pepei* is the only species of the genus to have a purely shrub-like growth form, but we have seen single-stemmed individuals up to 3 m tall that thus fulfil the criteria of a tree (Miehe et al. 2007). On the other hand, *P.microphylla*, *P.nana* and *P.rodolfovasquezii* mainly also have shrub-like growth, even though occasional trees are found in *P.microphylla* and *P.rodolfovasquezii*. Many individuals of *P.tarapacana* also only reach shrub form at their distributional limit in arid south-western Bolivia. Generally speaking, tree height within species decreases with decreasing temperatures (at high elevations) and precipitation (Hoch and Körner 2005; Kessler et al. 2014; Camel et al. 2019a), but in arid regions, there are complex interactions, so that shady (colder but more humid) habitats have taller trees than sunny (warm) slopes (Kessler et al. 2007). In many places, excessive logging or fires destroy the apical meristem, leading to the formation of numerous new shoots and thus to shrub-like growth. The trunk and branches usually have a gnarled, often twisted habit; occasionally the trunk lies on the ground for several meters (Young 1993). The trunk can be over 1 m thick (Koepcke 1961; Young 1993; Hertel and Wesche 2008) but is usually much slimmer.

### ﻿Bark

**Figure 2.**
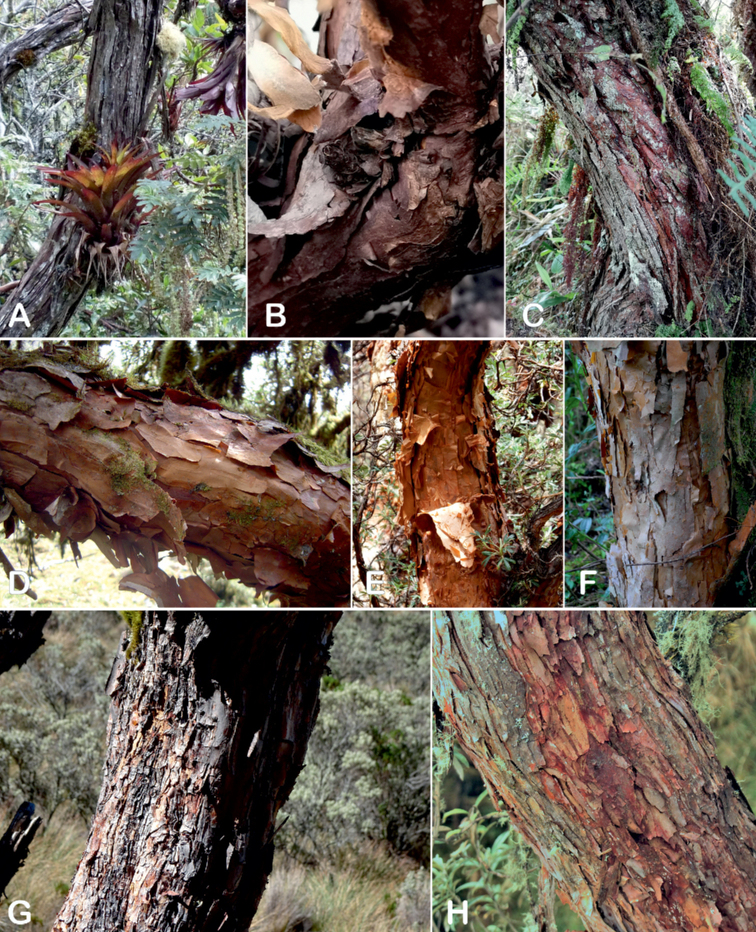
The multi-layered, shredding bark characteristic of all species of the genus **A***P.multijuga* Cajamarca, Peru **B***P.hieronymi* cultivated at Zurich Botanical Garden **C***P.humboldtii* Chimborazo, Ecuador **D***P.pepei* La Paz, Bolivia **E***P.incana* Papallacta, Ecuador **F***P.pauta* Ecuador **G***P.sericea* Colombia **H***P.canoi* Junin, Peru. Photographs **A, E** E.G. Urquiaga F. **B, C, F** T.E. Boza E. **D** A. Fuentes **G** A. Möhl **H** H.R. Quispe.

The bark of *Polylepis* is one of the characteristic features of the genus. Indeed, the name *Polylepis* is derived from the Greek words *poly* (many) and *lepis* (layers, skins), referring to the shredding, multilayered bark that is common to all species of the genus. The bark can be made up of more than 100 such layers (Miyagawa 1975; Kessler 1995a) and may be up to 3 cm thick, but is usually no thicker than 1 cm (Kessler 1995a). Within the genus, there are differences in the thickness, structure and color of the bark. In section Sericeae, species. such as *P.pepei*, *P.rodolfovasquezii* and *P.sericea*, have a bark that is thin, flakes off in relatively thick, long stripes and, when fresh, has a light brown to orange-brown color (Fig. 2). The bark of members of section Reticulatae, such as *P.hieronymi*, has a similar structure, but a more grey-brown color (Kessler 1995a). In species of sections *Australes*, *Incanaee* and *Subsericantes*, the bark typically is significantly thicker, flakes off in thin, short pieces and has a deep red-brown color. Despite these general trends, because the bark characteristics also change with tree size and environmental conditions (wind, cover of epiphytes, etc.), there is much variation in bark development between individuals of the same species and bark characters are not of taxonomic value at the species level.

### ﻿Branching pattern

**Figure 3.**
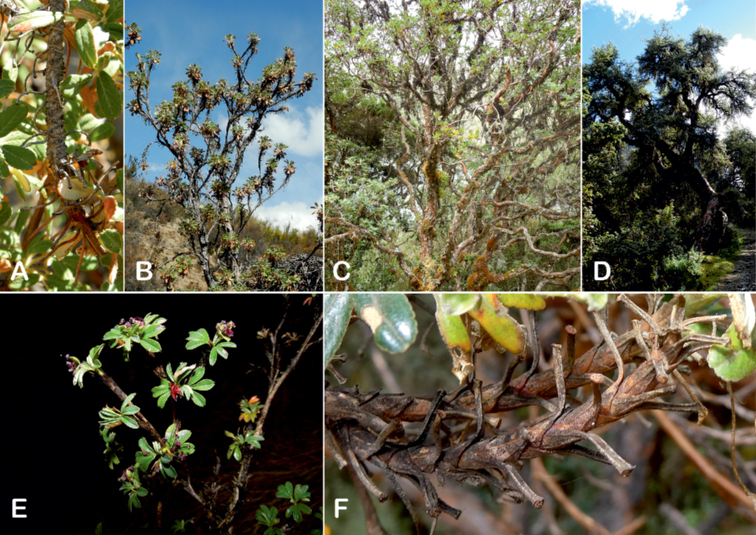
Branching patterns of *Polylepis*: petioles remaining on the branches **A***P.rugulosa* Moquegua, Peru **F***P.incana* Napo, Ecuador; leaves closely clustered at the top of the branches **B***P.pallidistigma* Puno, Peru **E***P.rodolfo-vasquezii* Huancavelica, Peru; twisted stems and branches **C***P.humboldtii* Chimborazo, Ecuador **D***P.sacra* Cusco, Peru. Photographs **A–D** T.E. Boza E. **E** G. Vargas **F** E.G. Urquiaga F.

The branching of *Polylepis* is sympodial (Bitter 1911; Simpson 1979, 1986; Kessler 1995a). *Polylepis* trees tend to have twisted stems and branches which might be related to the windy, cold and arid habitats (Simpson 1979). The branches often show a striking arrangement of the leaves: on young shoots, the leaves are usually all closely clustered at the top, causing a shrub-like growth, whereas the basal internodes stretch rather significantly afterwards, typically for 5–12 cm (Bitter 1911; Simpson 1979; Kessler 1995a) (Fig. 3). This branching mode is a typical feature of the genus but appears not to be useful to distinguish species. Only the degree of compression of the end rosette varies between species, with species of section Sericeae having less compressed shoots (Bitter 1911). However, this character is difficult to quantify, since its expression can vary greatly depending on the location and age between individuals of a species and even on a plant, for example, on shade and sun shoots (Kessler 1995a). In *Polylepis*, the petioles usually remain on the branches for a long time even after the leaves have fallen off.

### ﻿Stipule sheaths

**Figure 4.**
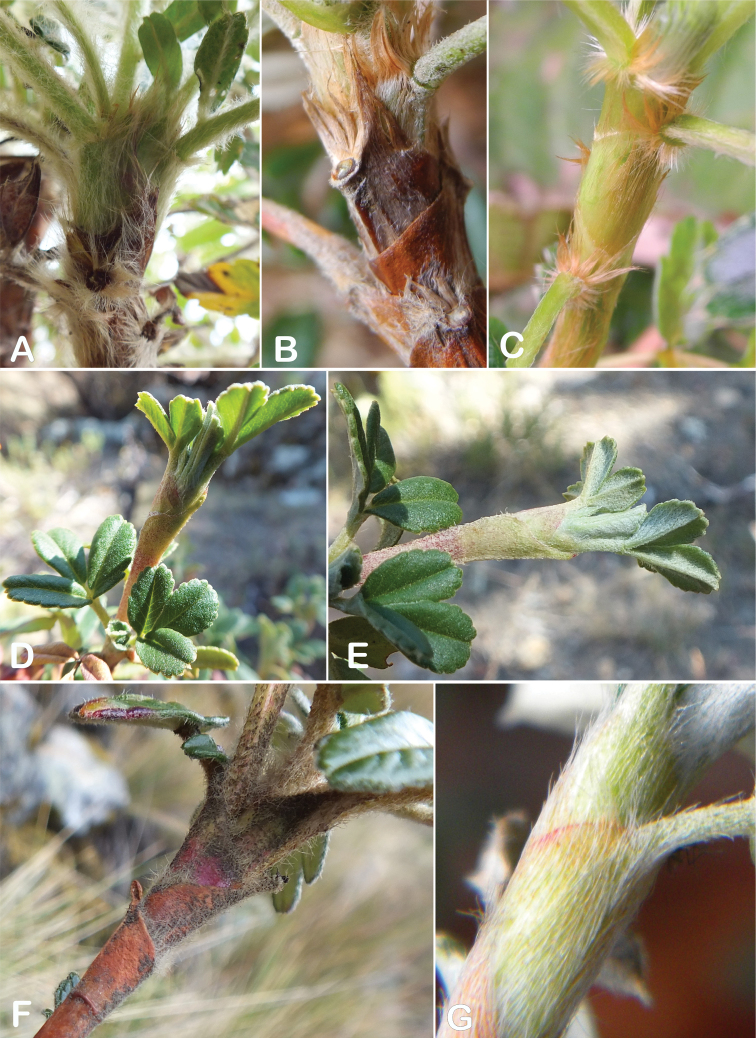
Stipule sheaths congested at the ends of the shoots **A***P.multijuga* Cajamarca, Peru **B***P.reticulata* Azuay, Ecuador **C***P.ochreata* Pichincha, Ecuador **D, E***P.fjeldsaoi* Ayacucho, Peru **F***P.weberbaueri* Ancash, Peru **G***P.subsericans* Cusco, Peru. Photographs **A, D–F** E.G. Urquiaga F. **B, C, G** T.E. Boza E.

Another characteristic feature of the genus is the growth of the two stipules fused around the branch, forming a sheath. The sheaths are congested at the ends of the shoots and shaped like tubes nested inside each other (Fig. 4). These remain intact for several years even after the leaves have fallen off and provide some taxonomically important features: a) Small spurs can be found on both sides of the petiole at the upper margin. Their size, shape and hairiness are relatively constant within the species; b) The hair type on the outside of the stipule sheaths also varies significantly between the species; c) Long, coarse or woolly hair often extends from the inside of the stipule sheaths beyond the edge of the sheaths. With increasing age, the stipule sheaths become bald; an examination of young shoots is therefore necessary (Simpson 1979; Kessler 1995a). However, short glandular hairs are often easier to find on older stipule sheaths than on young sheaths, since they are then no longer covered by longer wool or felt hairs.

### ﻿Indument

**Figure 5.**
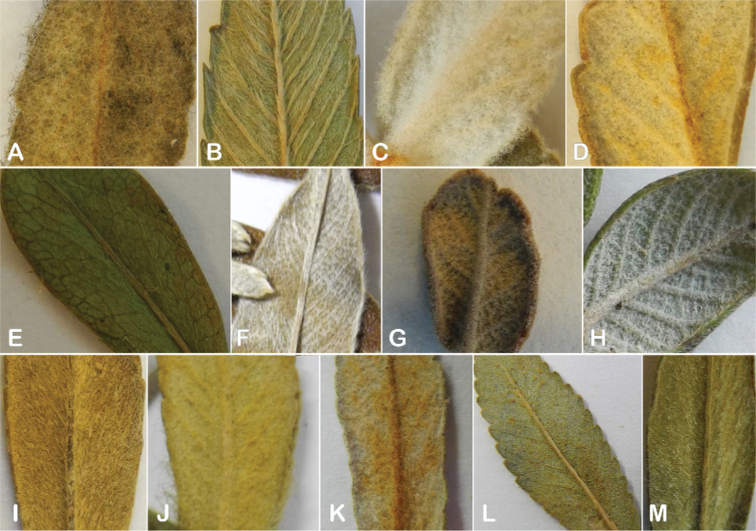
Lower leaflet surfaces of *Polylepis* species with different types of hairs. Lanate: **A***P.lanata***B***P.serrata*. Pannose: **C***P.besseri***D***P.rugulosa*. Sericeous: **E***P.rodolfo-vasquezii***F***P.sericea*. Tomentose: **G***P.microphylla***H***P.reticulata*. Pilose: **I***P.flavipila***J***P.pilosissima*. Villous: **K***P.acomayensis*. Puberulous: **L***P.neglecta*. Strigose: **M***P.subericans*. Photographs by T. E. Boza E.

The types and density of hairs represent some of the most important taxonomic features within the genus, although there are significant fluctuations especially in the length and density of the hair within species (Fig. 5). Hairs can be found on the stipule sheaths, petioles and rachises, under- and uppersides of the leaflets, flower bracts, sepals, stamens, styles and fruits, often with different hairs on different organs.

Bitter (1911) distinguished two groups of hairs: multicellular, mostly glandular *capilli resiniferi* or *capilli pulverulenti* and unicellular, non-glandular hairs as *pili*. Since there is a great variability in this last group in particular (Simpson 1979; Kessler 1995a; Romoleroux 1996), we here recognized eight hair types, with the terms following Hickey and King (2000).

1. Lanate: woolly, long, interwoven hair that gives a rough, woolly impression.
2. Pannose: felt-like, composed of densely matted woolly hairs.
3. Pilose: softly hairy, with short hairs.
4. Puberulous: slightly hairy (minutely pubescent).
5. Sericeous: silky, short to long, straight, smooth-fitting hair that give a silky impression.
6. Strigose: long, straight, rough or stiff hairs or bristles that give a rough-haired impression.
7. Tomentose: densely covered in soft and very curled hairs.
8. Villous: covered with long, shaggy hairs.

In some species, glandular hairs are intermixed with the longer hairs, often tinting the latter ones yellowish, as in *P.flavipila* and *P.incana*. In other species, only glandular hairs are found on some organs. In the extreme case of *P.tarapacana*, the resin forms a thick, translucent layer on the upperside of the leaflets.

### ﻿Leaves and leaflets

The imparipinnate leaves provide some of the most important taxonomic features within the genus, especially since their features are often correlated with those of the inflorescences and fruits. In addition, they can be determined, based on vegetative material. Important features are:

**Figure 6.**
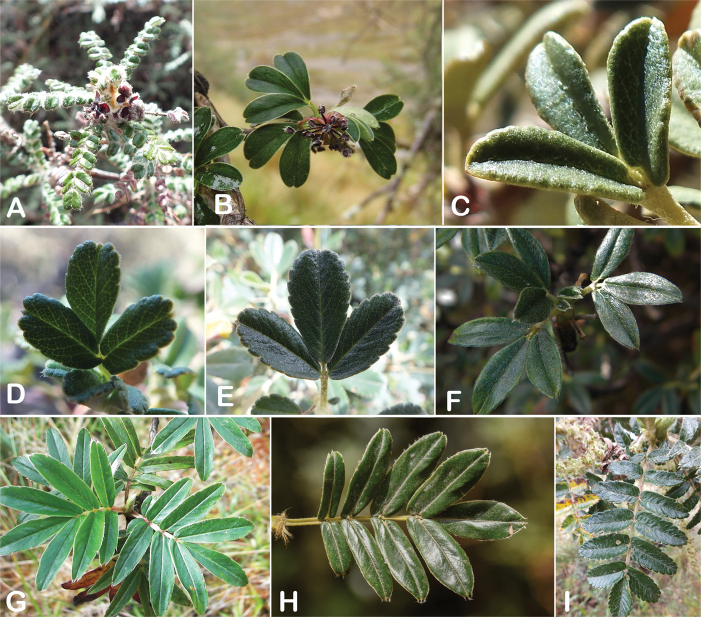
Leaflet sizes in *Polylepis*: **A***P.microphylla*; 0.3–0.7 × 0.2–0.5 cm **B***P.rodolfo-vasquezii*; 0.9–1.1 × 0.4–0.6 cm **C***P.tarapacana*; 0.7–0.8 × 0.3–0.4 cm **D***Polylepisfjeldsaoi*; 1.2–2.1 × 0.6–0.7 cm **E***P.incana*; (1.4–)1.8–2.7 × 0.4–0.7 cm **F***P.subsericans*; (1.3–)1.7–2.8 × 0.5–0.7 cm **G***P.canoi*; (2.4–)3.4–3.9 × (0.8–)1.1–1.5 cm **H***P.humboldtii*;1.8–2.8 × 0.6–0.9 cm **I***P.multijuga*; 2.9–3.6(–5.4) × 1.1–2.0 cm. Photographs **A, D, E, I** E.G. Urquiaga F. **B** G. Vargas **F** T.E. Boza E. **G** H. Huaylla **H** E. Bastidas.

**Figure 7.**
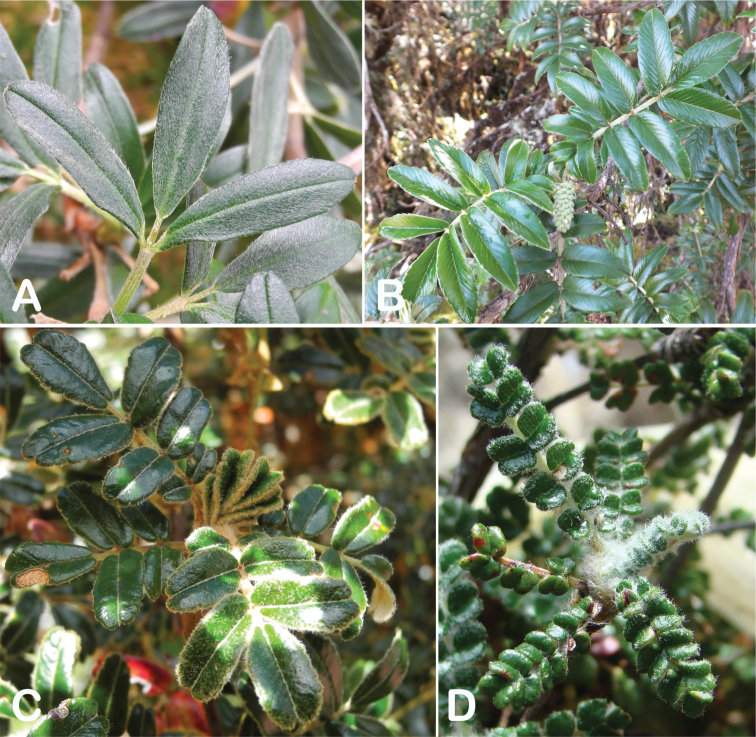
Number of lateral leaflets pairs in *Polylepis*: **A***P.subsericans* (1) **B***P.serrata* (4–7) **C***P.weberbaueri* (2–3) **D***P.microphylla* (3–6). Photographs by T. E. Boza E.

**Figure 8.**
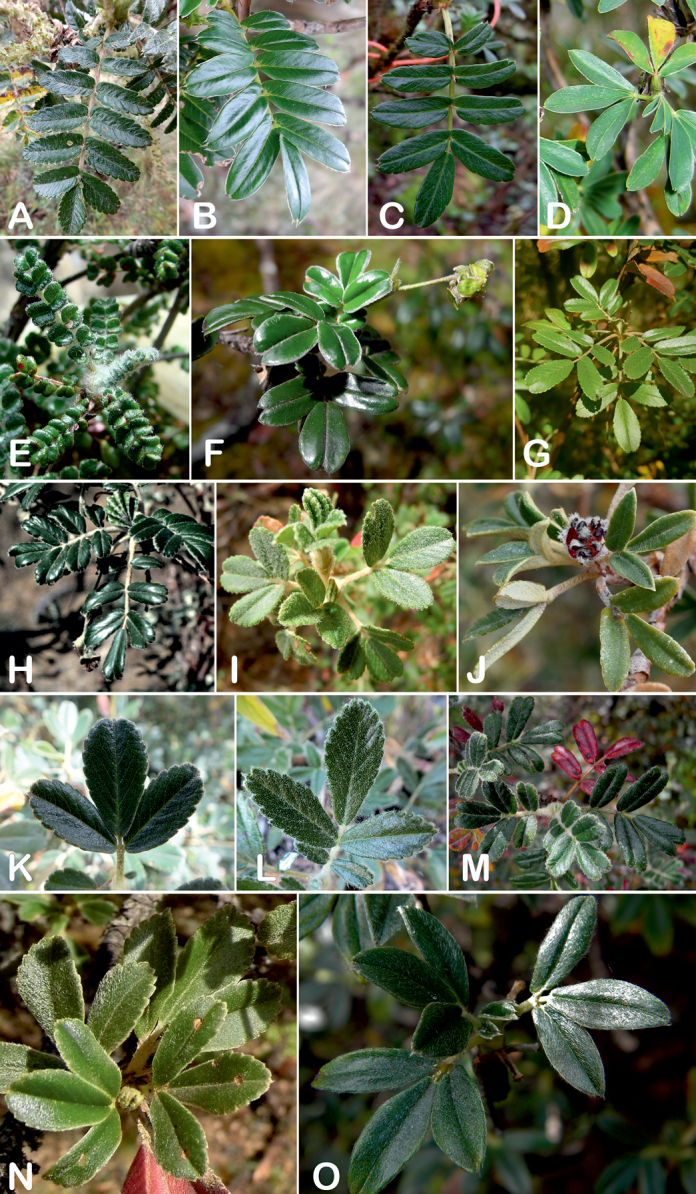
Leaves showing leaflet shapes, margins and apices in the sections and subsections of *Polylepis*: section Sericeae: **A***P.multijuga*; elliptic, serrate, obtuse (subsect. Lanuginosae) **B***P.ochreata*; elliptic, entire to slightly serrate, emarginate (subsect. Sericeae) **C***P.pauta*, elliptic, crenate, emarginate (subsect. Pauta) **D***P.rodolfo-vaquezii*, elliptic, entire, emarginate (subsect. Pepea). Section Reticulatae: **E***P.microphylla*, broadly elliptic, entire, deeply emarginate **F***P.reticulata*, elliptic to obovate, entire or slightly crenate, deeply emarginate. Section Australes: **G***P.australis*, elliptic, serrate, emarginate. Section Incanaee: **H***P.besseri*, obovate, crenate, obtuse or emarginate and **I***P.pallidistigma*, elliptic, crenate, round or emarginate (subsect. Besseria) **J***P.tarapacana*, obovate, entire or very slightly crenate, obtuse or acute and **K***P.incana*, elliptic to obovate, crenate, obtuse to emarginate (subsect. Incanaee) **L***P.acomayensis*, narrowly obovate, crenate, round to emarginate and **M***P.sacra*, obovate, crenate, emarginate (subsect. Racemosae) **N***P.flavipila*, obovate, crenate, acute or emarginate. Section Subsericantes: **O***P.subsericans*, narrowly elliptic, entire to slightly serrate, round or emarginate. Photographs **A, E, K, L** E.G. Urquiaga F. **B–G, M, O** T.E. Boza E. **H** M. Kessler **J** A. Domic **D** G. Vargas.

1. Number of lateral leaflets pairs, which can range from 1 to 7 (Fig. 6), but often varies within the species and even on one specimen, for example, on sun and shade branches. Plants growing under more humid conditions tend to have more leaflets, so that even species which normally only have a single pair of lateral leaflets (e.g., *P.tomentella*) can have a second pair of smaller ones (Kessler 1995a, b).
2. Leaflets size (Fig. 7). For the largest pair of leaflets in a leaf, this ranges from about 0.3–0.7 × 0.2–0.5 cm in *P.microphylla* to 2.9–3.6(–5.4) × 1.1–2.0 cm in *P.multijuga*.
3. Leaflet shape (Fig. 8), which can range from elliptic to ovate and obovate.
4. Leaflet apex (Fig. 8), which can range from acute to deeply emarginate.
5. Leaflet margin (Fig. 8), which can range from entire to serrate.
6. Number of teeth/crenations in non-entire leaflets.
7. Upper leaflet surface hair type, density and length.
8. Lower leaflet surface hair type, density and length (Fig. 5).

### ﻿Inflorescences and flowers

**Figure 9.**
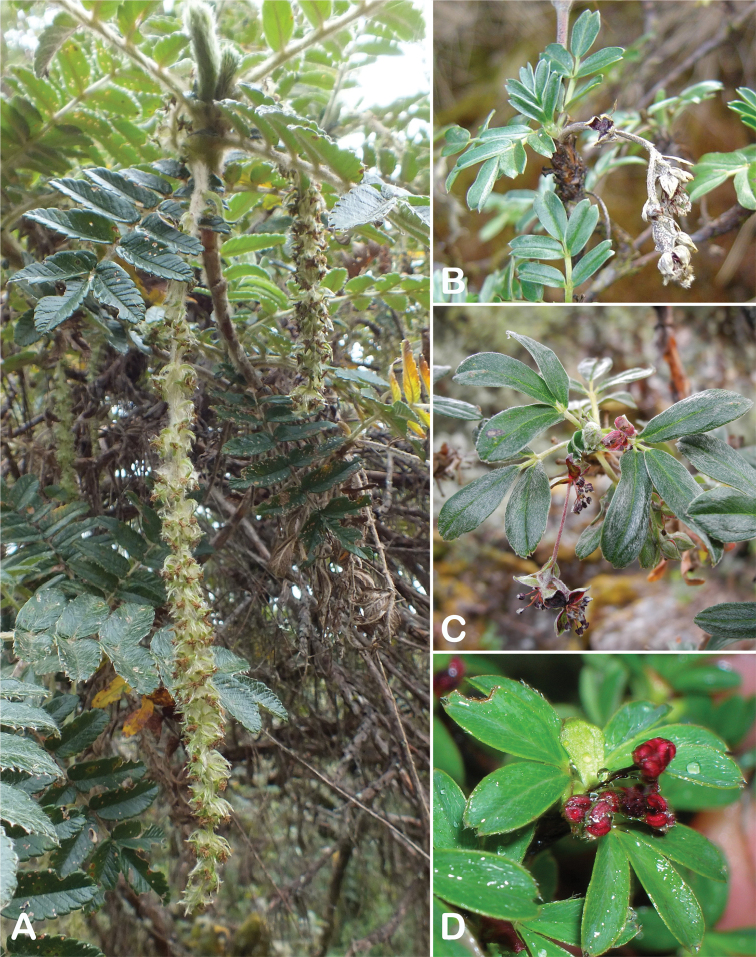
Inflorescence length and number of flowers in *Polylepis*: **A***P.multijuga* (15.4–36.0 cm; 47–83) **B***P.pepei* (1.2–3.5 cm; 3) **C***P.subsericans* (1.9–5.6 cm; 3–6) **D***P.rodolfo-vasquezii* (0.9–1.1 cm; 1). Photographs **A** E. G. Urquiaga F. **B** A. Fuentes **C, D** T.E. Boza E.

The inflorescences are simple clusters, rarely branched, generally long and pendulous as in *P.multijuga* (15.4–36.0 cm; 47–83 flowers), *P.ochreata* (8.1–17.4 cm; 21–49 flowers) and *P.serrata* (7.6–17.3 cm; 16–35 flowers). In other species, the inflorescences are more reduced, in the extreme to the axillary region of the leaves, such as in *P.microphylla* (3.8–5.3 cm; 1–3 flowers), *P.pepei* (1.2–3.5 cm; 3 flowers) and *P.rodolfovasquezii* (0.9–1.1 cm; 1 flower) (Fig. 9).

**Figure 10.**
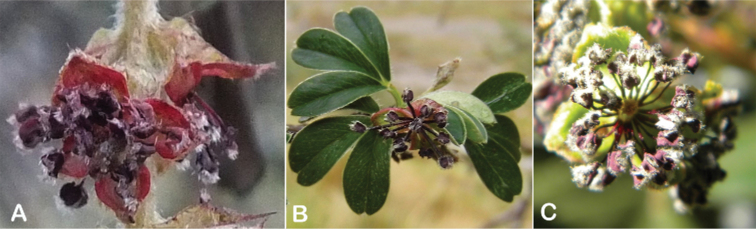
Number of stamens in *Polylepis***A***P.albicans* (7–18) **B***P.rodolfo-vasquezii* (13–15) **C***P.sacra* (23–27). Photographs **A, C** T.E. Boza E. **B** G. Vargas.

The flowers of *Polylepis* are hermaphroditic and have a number of adaptations to wind pollination: the petals are missing, the 3–4 sepals are mostly green or rarely red, nectar or fragrances are missing, the stamens are exerted and the styles are multilobed. Previous taxonomic work has rarely taken into account flower features. However, Simpson (1979) previously pointed out that the number of stamens differs among and within the species (Fig. 10). Indeed, we have found great variability, fluctuating from *P.pepei* (5–9 stamens per flower) to *P.sacra* (23–27) and we use this trait for species delimitation. The anthers have long purple filaments, are always hairy, and colored red or violet, apparently without taxonomically informative variation (Bitter 1911; Simpson 1979; Kessler 1995a). In contrast, the length of the styles differs between species and is here used for the first time to differentiate species in *Polylepis*. Style length ranges from 0.9–2.0 mm in *P.australis* to 3.0–4.9 mm in *P.pepei*.

### ﻿Fruits

**Figure 11.**
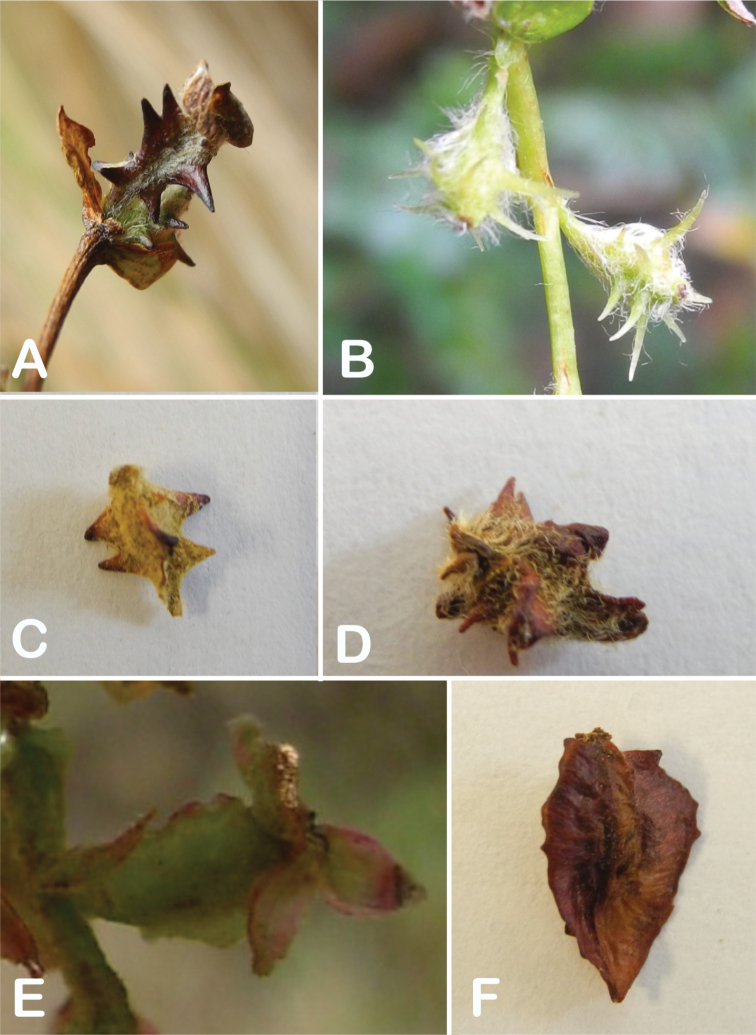
Fruit type in *Polylepis*: **A***P.reticulata*: variable numbers and placement of flattened spines **B***P.pauta*: with variable numbers and placement of flattened spines **C***P.flavipila*: irregular flattened ridges with a series of spines **D***P.subsericans*: irregular flattened ridges with a series of spines **E, F***P.australis*: 2–3 irregular and pronounced thin wings. Photographs by T. E. Boza E.

The fruits are indehiscent achenes that envelop the single carpel with only one ovule. The surface has differently-shaped protuberances including irregular flattened ridges (as in section Subsericantes), flattened spines (in sections *Sericeae* and *Reticulatae*), thick wings and ridges (in sections *Incanaee* and *Subsericantes*) (Fig. 11) and thin wings (in section Australes). These traits are thus useful for sectional and subsectional delimitation, but, with a single exception (*P.crista-galli*), do not have taxonomic value for species delimitation. On the other hand, the extent and type of hair on the fruits are of taxonomic value at species level. The seeds are elongated, spindle-shaped, with a thin or subcoriaceous testa (Romoleroux 1996).

## ﻿Evolution

The evolutionary history of *Polylepis* is poorly understood. This is linked to the complex taxonomy of the genus, which has led to highly variable classifications over time (see Taxonomic history) and which, in itself, is due to the recent and presumably ongoing radiation of the genus coupled with hybridization and polyploidization. We here review these aspects with respect to their relevance for the taxonomic treatment of the genus.

### ﻿Pollination biology and seed dispersal

*Polylepis* is wind pollinated. Earlier studies suggested low dispersal distances in the range of a few tens of meters (Salgado-Laboriau 1979; Salgado-Laboriau et al. 1984; Kessler 1995a). However, pollen dispersal of up to 80 km was later documented in *P.australis* (Seltmann et al. 2009a) and genetic studies also document extensive gene flow between populations (Schmidt-Lebuhn et al. 2006b; Seltmann et al. 2009a), suggesting extensive long-distance pollen dispersal. Likely, as is typical for wind-dispersed plants (Whitehead 1983; Friedman and Barrett 2009), most pollen is dispersed over short distances, while there is also substantial long-distance pollen dispersal. Selfing can occur in *Polylepis*, although in *P.australis*, seed germination rates are lower in selfing than in outcrossed pollination, presumably due to incomplete gametophytic self-incompatibility (Seltmann et al. 2009b).

The fruits of *Polylepis* are poorly adapted for long-distance dispersal. Some species have long thin spines that appear to be adapted for ectozoochoric dispersal, whereas others have thin wings designed for wind dispersal (Simpson 1979, 1986). Nevertheless, actual fruit dispersal distances appear to be in the range of tens of meters (Enrico et al. 2004; Torres et al. 2008; Quinteros-Casaverde et al. 2012). Dispersal of *Polylepis* fruits over distances of hundreds of meters to a few kilometers may only occasionally occur in the fur of animals or in mud on the animal’s feet. Accordingly, gene flow in *Polylepis* is likely to be mainly due to pollen dispersal (Schmidt-Lebuhn et al. 2007).

A further potential unknown complication in *Polylepis* may be the occurrence of apomixis (asexual seed production). Apomixis leads to taxonomic complications because the scarcity of sexual reproduction leads to the formation of numerous clonal lineages that are evolutionarily partly independent (Campbell and Dickinson 1990; Richards et al. 1996). Apomixis is well known in taxonomically complex genera of Rosaceae such as *Alchemilla*, *Crataegus*, *Rubus* and *Sorbus* (Nybom 1988; Talent and Dickinson 2007; Gehrke et al. 2008; Pellicer et al. 2012; Samaniego et al. 2018). Kerr (2004) suggested that apomixis might occur in *Polylepis*, but so far, this has not been confirmed.

### ﻿Hybridization

Hybridization is widespread in the plant kingdom and is also well known in Rosaceae (Dickinson et al. 2007; Lo et al. 2009; Robertson et al. 2010). Hybridization includes a wide range of phenomena, ranging from occasional hybridization events between evolutionarily independent species where the offspring does not further reproduce, to hybridogenic species formation (Mallet 2007; Rieseberg and Willis 2007; Whitney et al. 2010). *Polylepis* is no exception in this regard, although this was long overlooked due to the similarity of the species and, accordingly, the difficulty of identifying hybrids.

Simpson (1979, 1986) was the first to suggest hybridization between species of *Polylepis*, based on morphologically intermediate specimens between *P.incana* and *P.racemosa*. Later, based on her morphological taxonomic revision of the Ecuadorean species, Romoleroux (1996) proposed that the following species hybridize where their ranges meet or overlap: *P.incana* and *P.pauta*, *P.incana* and *P.ochreata* (as *P.sericea*), *P.incana* and *P.reticulata* and *P.pauta* and *P.ochreata* (as *P.sericea*). These hybrids are also recognized in the present treatment.

For Bolivia, also based on morphological intermediacy in mixed species forests, Kessler (1995a, b) identified further putative hybrid individuals and populations. These ranged from occasional individuals found where two species meet, to large hybrid zones up to hundreds of kilometers wide, although some of these are now interpreted differently. Kessler (1995a, b) reported occasional hybrids between *P.besseri* and *P.incanoides*, *P.besseri* and *P.tomentella*, *P.incanoides* and *P.subtusalbida*, *P.incarum* and *P.triacontandra* and *P.neglecta* and *P.subtusalbida*. All of these are also recognized here. More extensive hybridization was found in an extensive introgression zone at the locality Mojón, Cochabamba, where *P.besseri*, *P.lanata* and *P.subtusalbida* meet and form a large hybrid swarm in which all possible combinations of character traits among the three species can be found in an area of a few square kilometers. At an even larger scale, in southern Bolivia, there is a large region about 200 km wide where *P.tarapacana* and *P.tomentella* broadly intergrade. Simpson (1979) used this intergradation to justify treating both as conspecific, but later considered that the species are sufficiently distinct over most of their respective ranges to be treated as distinct species (Simpson 1986). Kessler (1995a, b) followed suit and treated the intermediate populations as a hybridization zone. A further putative intermediate population was recognized by Kessler (1995a, b) between *P.lanata* and *P.triacontandra*. However, this population was later studied in more detail and is now recognized as the distinct species *P.pacensis* (Kessler and Schmidt-Lebuhn 2006).

Besides these hybridization events between extant taxa, there is also indirect evidence for speciation via hybridization. Schmidt-Lebuhn et al. (2006a) proposed that *P.crista-galli* may have originated from hybridization between an ancestor of *P.australis/neglecta* and *P.subtusalbida*. This is based on the fact that, in the small area of geographical overlap between *P.neglecta* and *P.subtusalbida*, Kessler (1995a, b) found two hybrid individuals with intermediate characters between the parent species and that are morphologically indistinguishable from *P.crista-galli*. Genetically, *P.crista-galli* also appears to be intermediate between the putative parental species (Schmidt-Lebuhn et al. 2006a). Interestingly, the current range of *P.crista-galli* in southern Bolivia to northernmost Argentina almost perfectly fills the distributional gap between *P.neglecta* (central Bolivia) and *P.australis* (Argentina). This suggests that an ancestral form of *P.australis/neglecta* might have occurred from central Bolivia to Argentina and that the central part of this range was lost due to hybridization with *P.subtusalbida* or a related species, resulting in the formation of *P.crista-galli*. Based on morphological intermediacy, in the present study, we also propose further candidates for hybridogenic speciation, such as *P.albicans* or *P.frontinensis*, in both cases involving a member of section Sericeae and a member of section Reticulatae. Other species of potential hybridogenic origin include *P.incarum* and *P.racemosa*.

Summarizing, based on morphological traits, hybridization in *Polylepis* appears to be common, ranging from cases where species occur sympatrically, but where no putative hybrids have yet been found (Kessler 1995a) to cases of occasional primary hybridization between two well characterized species, large hybrid swarms between two or three species and, finally, even cases of hybridogenic speciation that may have wiped out part or all of the parent species. Interestingly, the majority of hybridization events known in *Polylepis* are between species of different sections or subsections. This may partly be because species of a section or subsection are mostly allopatric, so that they cannot easily hybridize, but also because it might be difficult to recognize hybrids between species that are morphologically very similar, as is typical within sections or subsections.

Direct molecular evidence of hybridization in *Polylepis* is still lacking. However, both Kerr (2004) and Schmidt-Lebuhn et al. (2006a), using molecular markers, found low levels of phylogenetic resolution, based on AFLP (amplified fragment length polymorphism) and sequence data, respectively and that geographically separate accessions of widespread species tend to cluster genetically with geographically close individuals of unrelated species rather than with their conspecific samples from geographically more distant locations. Based on incongruence between chloroplast and nuclear markers, Kerr (2004) even found evidence of hybridization between *Polylepis* and the closely related genus *Acaena*. All this suggests that gene flow between species of *Polylepis* may be even more frequent than inferred from morphology alone.

### ﻿Ploidy levels

Changes in ploidy level are an important macro- and microevolutionary processes (Adams and Wendel 2005; Jiao et al. 2011). Polyploidy results from either auto- or allopolyploidization, the latter often linked to hybridization, where it allows for the stabilization of genomes of mixed origin (de Wet 1971; Tate et al. 2005). Besides its evolutionary implications (Weiss-Schneeweiss et al. 2013), polyploidy is also of taxonomic relevance, since populations of different ploidy levels are at least partly reproductively isolated, allowing for divergent evolutionary trajectories that can be treated at species level (Soltis et al. 2007). Rosaceae is renowned for the occurrence of polyploid complexes, for example, in *Crataegus* (Lo et al. 2010) and *Sorbus* (Robertson et al. 2010; Pellicer et al. 2012).

The following account of ploidy levels in *Polylepis* is based on Boza Espinoza et al. (2020b). Ploidy levels in *Polylepis* have been studied by direct chromosome counts, nucleus size measurements via flow cytometry and guard cell measurements (Table 2). Each of these methods has its own advantages and limitations. Direct chromosome counts are difficult in *Polylepis* due to the small size of the chromosomes and high chromosome numbers and because they require live plant material (Simpson 1979; Kessler 1995b; Quija-Lamina et al. 2010; Schmidt-Lebuhn et al. 2010). Genome size measurements via flow cytometry also require live material and do not provide direct numbers of chromosomes. Guard cell size is well known to be correlated to ploidy level in angiosperms in general (Masterson 1994; Sugimoto-Shirasu and Roberts 2003; Beaulieu et al. 2008) and Rosaceae in particular (Joly and Bruneau 2007) and has been shown to be correlated to genome size in *Polylepis* (Schmidt-Lebuhn et al. 2010). Still, there is notable variation of guard cell sizes within species, even when only a single ploidy level is known in the species (Schmidt-Lebuhn et al. 2010; Boza Espinoza et al. 2020b). This variation may be due to anatomical plasticity of a species depending on growth conditions, differences in measurement methods or aneuploidy. Thus, guard cell measurements allow inference of major ploidy levels, but not of minor variations in chromosome numbers or genome sizes. Such variations have been found in detailed studies of Ecuadorean species, based on genome measurements and chromosome counts (Quija-Lamina et al. 2010; Quijia Lamiña et al. 2010; Montalvo 2013; Zurita et al. 2013; Segovia-Salcedo 2014; Segovia-Salcedo and Quijia-Lamiña 2014; Caiza et al. 2018). Based on values of dozens of individuals of each species, these studies documented that many species have variable chromosome numbers and genome sizes, suggesting reductions in chromosome numbers that could be the result of aneuploidy (loss of DNA and reduction of chromosome size) and dysploidy (chromosome fusion) (Stebbins 1971; Morgan et al. 1994; Mishima et al. 2002).

**Table 2.** Overview of the available data on genome sizes, chromosome numbers and guard cell sizes in species of *Polylepis*. Where possible, literature records were assigned to this taxonomy, but a few data points (especially from the hybrid zone at Mojanda, Ecuador) had to be excluded because they could not be unambiguously assigned to a species. Depending on data source, we report the mean ± standard deviation, only the mean or a range. Data sources: 1. Kessler (1995b); 2. Quijia Lamiña et al. (2010); 3. Montalvo (2013); 4. Zurita et al. (2013); 5. Segovia-Salcedo and Quijia-Lamiña 2014; 6. Segovia-Salcedo (2014); 7. Kessler et al. (2014); 8. Caiza et al. (2018); 9. Boza et al. (2020). Abbreviations: Ar = Argentina, Ec = Ecuador, cult. = cultivated (in botanical garden), ind. = individuals, pl = planted.

Species	Genome size		Chromosome number		Guard cell length		Inferred ploidy levels (2n = /x =)
	Voucher/locality	Size (pg)	Voucher/locality	N	Voucher/locality	Length (µm)	
**Section Sericeae**							
**Subsection Lanuginosae**							
*P.lanuginosa*	3 ind. Sangay, Ec ^6^	*1.42* ± *0.13*					diploid / x = 6
			6 ind. Zhud, Ec ^2^	*38–42*			
					*Laegaard 55036* ^1^	*10.8* ± *1.4*	
					*Laegaard 102637* ^1^	*11.8* ± *1.6*	
*P.multijuga*					*Boza 3070* ^9^	11.0 ± 2.0	diploid / x = 6
					*Boza 3074* ^9^	11.2 ± 1.7	
					*Boza 3076* ^9^	12.8 ± 2.3	
**Subsection Pauta**							
*P.longipilosa*					*Jaramillo 10862* ^9^	10.3 ± 1.6	diploid / x = 6
*P.pauta*	2 ind. Oyacachi, Ec ^6^	*3.21* ± *0.04*					tetraploid / x = 12 plus aneuploids; perhaps also diploid / x = 6
	2 ind. Oyacachi, Ec ^6^	*3.37* ± *0.18*					
			25 ind. Papallacta, Ec ^2, 5^	*67–83*			
			16 ind. Papallacta, Ec ^6^	*72*			
			15 ind. Cayambe-Coca, Ec. ^3^	*68–77*			
					*Kessler 2749* ^1^	*12.5* ± *1.8*	
					*Laegaard 102327* ^1^	*16.5* ± *2.8*	
*P.pauta*					Oyacachi, Ec ^8^	*14.4* ± *2.5*	
					Papallacta, Ec ^8^	*12.3* ± *1.9*	
					Papallacta, Ec ^9^	12.7 ± 1.9	
					Papallacta, Ec ^9^	12.7 ± 2.1	
					Papallacta, Ec ^9^	16.6 ± 1.6	
*P.serrata*	Cult. Göttingen ^1^	*1.57* ± *0.11*			Cult. Göttingen ^1^	*10.6* ± *0.9*	diploid / x = 6
	Cult. Göttingen ^1^	*1.61* ± *0.11*			Cult. Göttingen ^1^	*12.7* ± *1.8*	
**Subsection Sericeae**							
*P.albicans*					*Boza 3014* ^9^	10.8 ± 1.3	diploid / x = 6
					*Frimer 44* ^9^	13.5 ± 1.4	
					*Renvoize 5074* ^9^	12.2 ± 1.2	
*P.argentea*	Cult. Göttingen ^1^	*1.63* ± *0.15*			Cult. Göttingen ^1^	*12.4* ± *2.4*	diploid / x = 6
	Cult. Göttingen ^1^	*1.67* ± *0.15*			Cult. Göttingen ^1^	*13.0* ± *1.7*	
					Cult. Zurich ^9^	13.9 ± 1.7	
					*Chevarria 1035* ^9^	13.4 ± 1.2	
					*Hanold 85* ^9^	13.4 ± 1.2	
*P.canoi*					*Kessler 2880* ^1^	*14.3* ± *2.6*	diploid / x = 6
*P.frontinensis*					*Kessler 2772* ^9^	11.5 ± 1.3	diploid / x = 6
					*Kessler 2776* ^9^	13.0 ± 1.3	
*P.humboldtii*					*Carate 185* ^9^	12.4 ± 1.4	diploid / x = 6
*P.loxensis*			25 ind. Fierro Urco, Ec ^2, 5^	*39–42*			
					*Laegaard 19109* ^9^	12.0 ± 1.5	
					*Lewis 3804* ^9^	11.4 ± 1.1	
*P.ochreata*	2 ind. Yanacocha, Ec ^6^	*3.41* ± *0.09*					diploid / x = 6, tetraploid / x = 12, and hexaploid / x = 18; perhaps plus aneuploids or hybrids
	2 ind. El Ángel, Ec ^6^	*4.66* ± *0.57*					
			8 ind. El Ángel, Ec ^2, 5^	*37–40*			
			9 ind. Yanacocha, Ec ^2, 5^	*59–77*			
			16 ind. Yanacocha, Ec ^6^	*82*			
			15 ind. Yanacocha, Ec ^3^	*73–88*			
					*Molau 2536* ^9^	10.7 ± 1.3	
					*Laegaard 54474* ^9^	11.9 ± 1.1	
					*Romoleroux 1060* ^9^	11.7 ± 1.0	
					Yanacocha, Ec ^8^	*14.1* ± *2.3*	
*P.sericea*					*Dorr 5220* ^9^	13.3 ± 1.8	diploid / x = 6
**Subsection Pepea**							
*P.pepei*					*Kessler 2795* ^1^	*10.9* ± *1.6*	diploid / x = 6
					*Kessler 3386* ^1^	*11.8* ± *1.6*	
*P.rodolfo-vasquezii*	Cult. Göttingen ^1^	*1.60* ± *0.07*			Cult. Göttingen ^1^	*10.2* ± *1.5*	diploid / x = 6
	Cult. Göttingen ^1^	*1.70* ± *0.05*			Cult. Göttingen ^1^	*10.8* ± *1.3*	
**Section Reticulatae**							
*P.hieronymi*	Cult. Göttingen ^1^	*1.52* ± *0.02*			Cult. Göttingen ^1^	*12.6* ± *1.8*	diploid / x = 6
	Cult. Göttingen ^1^	*1.49* ± *0.04*			Cult. Göttingen ^1^	*11.9* ± *2.0*	
	52 ind. Ar ^7^	*1.45–1.57*					
					*Beck 9345* ^1^	*13.2* ± *1.8*	
					*Kessler 3123* ^1^	*11.2* ± *1.0*	
*P.microphylla*	Cult. Göttingen ^1^	*1.53* ± *0.06*			Cult. Göttingen ^1^	*13.9* ± *1.1*	diploid / x = 6 and tetraploid / x = 12 plus aneuploids
	Cult. Göttingen ^1^	*1.53* ± *0.07*			Cult. Göttingen ^1^	*14.3* ± *2.2*	
	2 ind. Ozongoche, Ec ^6^	*2.03* ± *0.22*					
			8 ind. Achupallas, Ec ^2, 6^	*70–82*			
					*Galiano 1999* ^1^	*14.2* ± *1.8*	
					Achupallas, Ec ^8^	*10.7* ± *1.9*	
*P.occidentalis*					*Diaz 2879* ^9^	11.3 ± 1.2	diploid / x = 6
					*Diaz 4012* ^9^	10.8 ± 1.0	
					*Sánchez 10285* ^9^	12.7 ± 1.6	
*P.quadrijuga*					*Gradstein s.n* ^1^	*12.2* ± *1.7*	diploid / x = 6
					*Gradstein s.n* ^1^	*12.3* ± *2.0*	
					*Olivares 570* ^9^	14.3 ± 1.3	
*P.reticulata*			11 ind. Soldados, Ec ^2^	*36–42*			diploid / x = 6 plus higher ploidy (hexaploid / x = 18?; in cultivated plants only?)
			3 ind. Oyacachi, Ec (pl) ^6^	~*118*			
					*Kessler 2746a* ^1^	*12.2* ± *1.4*	
					*Laegaard 102691* ^1^	*10.0* ± *0.9*	
					Cajas, Ec ^9^	10.5 ± 1.8	
					Cajas, Ec ^9^	12.3 ± 1.6	
					Cajas, Ec ^9^	11.4 ± 1.4	
*P.simpsoniae*	3 ind. Sangay, Ec ^6^	*1.40* ± *0.08*					diploid / x = 6
			25 ind. Zhud, Ec ^2^	*37–42*			
			2 ind. Sangay, Ec ^6^	*38*			
					*Laegaard 102677* ^1^	*12.2* ± *1.0*	
					Cajas, Ec ^9^	9.1 ± 1.1	
*P.weberbaueri*					*Acleto 364* ^1^	*12.3* ± *0.9*	diploid / x = 6
					*Boza 3018* ^9^	14.1 ± 1.1	
					*Boza 3148* ^9^	14.3 ± 1.5	
					*Smith 9568* ^9^	14.9 ± 1.1	
**Section Australes**							
*P.australis*	Cult. Göttingen ^1^	*2.98* ± *0.06*			Cult. Göttingen ^1^	*16.7* ± *2.6*	tetraploid / x = 12 and diploid / x = 6 plus triploid/ x = 9 hybrids and hexaploid / x = 18 autopolyploid derivate
	Cult. Göttingen ^1^	*3.03* ± *0.03*			Cult. Göttingen ^1^	*17.4* ± *1.6*	
	261 indiv. ^7^	*2.84–2.97*					
	75 indiv. ^7^	*1.44–1.54*					
	24 indiv. ^7^	*2.09–2.24*					
	1 indiv. ^7^	*4.15*					
					*Kessler 3350* ^1^	*18.9* ± *1.9*	
					*Lazaro 6695* ^9^	17.6 ± 2.4	
					*Lorentz 760* ^1^	*12.8* ± *1.4*	
					*Venturi 3010* ^9^	12.2 ± 1.8	
					*w/colector 2330* ^9^	15.5 ± 2.5	
					Cult. Zurich ^9^	22.7 ± 2.7	
*P.neglecta*	Cult. Göttingen ^1^	*1.54* ± *0.06*	Cult. Göttingen ^1^	~*80*	Cult. Göttingen ^1^	*13.9* ± *1.1*	diploid / x = 6; perhaps also tetraploid / x = 12
	Cult. Göttingen ^1^	*1.55* ± *0.09*			Cult. Göttingen ^1^	*14.3* ± *2.2*	
					*Kessler 3531* ^1^	*13.6* ± *2.3*	
					*Kessler 3633* ^1^	*13.2* ± *2.0*	
**Section Subsericantes**							
*P.flavipila*					*Boza 3163* ^9^	17.9 ± 1.8	tetraploid / x = 12
					*Boza 3167* ^9^	16.0 ± 1.5	
					*Boza 3168* ^9^	15.3 ± 1.6	
*P.pilosissima*					*Kessler 3426* ^1^	*18.0* ± *2.0*	tetraploid / x = 12
					*Kessler 3591* ^1^	*17.2* ± *2.2*	
					*Boza 3023* ^9^	17.3 ± 1.6	
					*Cerrate 1265* ^9^	15.6 ± 1.4	
					*Gentry 638* ^9^	16.2 ± 1.3	
					*Kessler 3428* ^9^	15.7 ± 1.1	
*P.subsericans*	Cult. Göttingen ^1^	*3.12* ± *0.18*			Cult. Göttingen ^1^	*16.6* ± *2.2*	tetraploid / x = 12
	Cult. Göttingen ^1^	*3.21* ± *0.11*			Cult. Göttingen ^1^	*17.3* ± *2.2*	
					*Toivonen s.n* ^1^	*18.3* ± *1.5*	
					*Toivonen s.n* ^1^	*18.5* ± *2.4*	
					*Sylvester 428* ^9^	13.9 ± 1.2	
					*Sylvester 868* ^9^	16.2 ± 1.7	
					*Sylvester 1287* ^9^	15.9 ± 1.8	
**Section Incanaee**							
**Subsection Racemosae**							
*P.acomayensis*					*Boza 3135* ^9^	16.2 ± 1.8	tetraploid / x = 12
					*Boza 3141* ^9^	15.1 ± 1.4	
*P.incarum*					*Jimenez 2716* ^1^	*18.3* ± *0.8*	tetraploid / x = 12
					*Kessler 3465* ^1^	*17.2* ± *1.9*	
					*Jimenez 2716* ^9^	17.6 ± 1.6	
					*Kessler 13515* ^9^	16.9 ± 2.1	
					*Shepard 150* ^9^	17.8 ± 2.2	
*P.lanata*					*Kessler 2851* ^1^	*19.6* ± *2.1*	tetraploid / x = 12
					*Kessler 2962* ^1^	*18.8* ± *1.7*	
*P.pacensis*					*Kessler 3028* ^1^	*15.9* ± *2.7*	tetraploid / x = 12
					*Mendez & Arcienaga 14* ^1^	*17.7* ± *1.3*	
					*Kessler 14528* ^9^	19.2 ± 1.7	
					*Lopez & Bermejo 4* ^9^	19.1 ± 1.9	
					*Lopez & Bermejo 10* ^9^	19.7 ± 2.0	
*P.racemosa* (all pl)	2 ind. Cotopaxi, Ec. ^6^	*4.48* ± *0.19*					tetraploid / x = 12 to octoploid / x = 24, with many intermediate and aneuploid ploidy levels
	2 ind. Cotopaxi, Ec. ^6^	*2.63* ± *0.20*					
	3 ind. Oyacachi, Ec. ^6^	*4.57* ± *0.11*					
			12 ind. Oyacachi, Ec ^2, 5^	*80–82*			
			2 ind. Oyacachi, Ec ^2, 5^	*72–77*			
			10 ind. Oyacaci, Ec. ^6^	*62–80*			
					*Ferreyra 12418* ^1^	*18.0* ± *1.6*	
					Papallacta, Ec ^8^	*21.7* ± *3.8*	
					Oyacachi, Ec ^8^	*17.6* ± *2.6*	
					*Arce 161* ^9^	17.2 ± 1.5	
					*Arce 167* ^9^	15.1 ± 1.3	
					*Arce 207* ^9^	13.8 ± 1.4	
					*Bird 1384* ^9^	16.1 ± 1.3	
					*Boza 3020* ^9^	16.7 ± 1.6	
					*Boza 3030* ^9^	15.1 ± 1.0	
					*Boza 3031* ^9^	14.2 ± 1.4	
					*Boza 3119* ^9^	18.0 ± 1.5	
					*Ferreyra 3792* ^9^	15.3 ± 1.6	
					*Kenehira 5* ^9^	15.9 ± 1.1	
					*Kessler 14608* ^9^	17.1 ± 1.1	
					*Laegaard 20465* ^9^	19.8 ± 2.0	
					*Laegaard 22351* ^9^	17.0 ± 1.9	
					*Leiva 741* ^9^	14.9 ± 1.2	
					*Leiva 1090* ^9^	16.4 ± 1.8	
					*Nuñez 8117* ^9^	16.3 ± 1.3	
					*Renvoize 4847* ^9^	17.7 ± 1.4	
					*Sánchez Vega 5322* ^9^	13.5 ± 1.1	
					*Smith 11076* ^9^	12.6 ± 1.0	
					*Soukup 3498* ^9^	16.7 ± 1.5	
					*Stork 9972* ^9^	16.6 ± 1.8	
					*Tovar 2371* ^9^	15.3 ± 1.4	
					*Velásquez 12* ^9^	16.3 ± 1.1	
					*West 3787* ^9^	13.2 ± 2.2	
*P.sacra*	Cult. Göttingen ^1^	*5.76* ± *0.26*			Cult. Göttingen ^1^	*20.2* ± *3.3*	octoploid / x = 24; perhaps also tetraploid / x = 12 or intermediates
	Cult. Göttingen ^1^	*5.72* ± *0.15*			Cult. Göttingen ^1^	*16.7* ± *3.3*	
					*Rosales 04* ^1^	*19.5* ± *0.8*	
					*Sylvester 644* ^9^	15.6 ± 1.4	
					*Sylvester 1262* ^9^	22.1 ± 1.2	
					*Sylvester 1270* ^9^	15.8 ± 1.3	
*P.triacontandra*			Cult. Göttingen ^1^	~*80*			tetraploid / x = 12; perhaps also lower ploidy levels
					*Beck 4976* ^1^	*18.9* ± *1.9*	
					*Kessler 3420* ^1^	*20.4* ± *1.1*	
					*Steudel 427* ^9^	13.5 ± 1.9	
					*Steudel 431* ^9^	14.1 ± 2.6	
					*Steudel 433* ^9^	18.0 ± 2.1	
**Subsection Besseria**							
*P.besseri*					*Kessler 2989* ^1^	*20.4* ± *2.8*	tetraploid / x = 12 or higher ploidy level
					*Kessler 2985* ^1^	*19.2* ± *1.9*	
*P.crista-galli*					*Beck 9343* ^1^	*16.6* ± *1.4*	tetraploid / x = 12
					*Kessler 3155* ^1^	*17.8* ± *2.1*	
*P.pallidistigma*					*Boza 3005* ^9^	17.2 ± 1.9	tetraploid / x = 12
					*Boza 3006* ^9^	17.1 ± 2.2	
					*Boza 3007* ^9^	18.5 ± 1.9	
					*Sylvester 1807* ^9^	16.4 ± 1.4	
					*Sylvester 1816* ^9^	17.3 ± 1.9	
					*Sylvester 1825* ^9^	18.5 ± 1.9	
*P.rugulosa*					*Ferreyra 2594* ^1^	*16.8* ± *1.9*	tetraploid / x = 12
*P.subtusalbida*					*Beck 7395* ^9^	22.1 ± 1.5	tetraploid / x = 12 and higher ploidy level
					*Kessler 216* ^9^	23.4 ± 2.4	
					*Ritter 1196* ^9^	15.9 ± 1.4	
**Subsection Incanaee**							
*P.fjeldsaoi*					*Mendoza 1019* ^9^	11.9 ± 2.6	diploid / x = 6
					*Mendoza 1032* ^9^	13.3 ± 1.4	
					*Mendoza 1057* ^9^	15.2 ± 1.7	
*P.incana*	3 ind. Sincholagua, Ec ^6^	*1.99* ± *0.34*					mainly diploid / x = 6 but also hexaploid / x = 18 (in cultivated plants only?)
	3 ind. Illinizas, Ec ^6^	*1.60* ± *0.14*					
	3 ind. Inga-Raya, Ec ^6^	*1.67* ± *0.30*					
	3 ind. Cayambe-Coca, Ec (pl) ^6^	*1.42* ± *0.10*					
	3 ind. Antisana, Ec (pl) ^6^	*4.67* ± *018*					
			16 ind. El Ángel, Ec ^2, 5, 6^	*(38 –) 42*			
			6 ind. Illinizas, Ec ^2, 5, 6^	*38*			
*P.incana*			30 ind. Cayambe-Coca, Ec ^2, 5, 6^	*(39 –) 42*			
			15 ind. Inga-Raya, Ec ^6^	*42*			
			15 ind. El Inga, Ec ^4^	*40–42*			
			15 ind. Papallacta, Ec ^4^	*41–42*			
			15 ind. El Ángel, Ec. ^4^	*40–42*			
					*Laegaard 102647* ^1^	*17.6* ± *2.0*	
					*Schmidt-Lebuhn 521* ^1^	*17.0* ± *2.3*	
					Illinizas, Ec ^8^	*9.7* ± *0.5*	
					*Boza 3066* ^9^	15.4 ± 1.8	
					*Boza 3095* ^9^	13.1 ± 1.5	
					*Laegaard 102282* ^9^	18.6 ± 2.2	
*P.incanoides*					*Kessler 3288* ^1^	*16.4* ± *1.6*	tetraploid / x = 12
					*Kessler 3293* ^1^	*18.3* ± *2.4*	
					*Beck 34512* ^9^	15.3 ± 1.9	
					*Kessler 2954* ^9^	13.2 ± 0.7	
*P.nana*	Cult. Göttingen ^1^	*2.93* ± *0.05*			Cult. Göttingen ^1^	*18.7* ± *1.7*	tetraploid / x = 12; also lower ploidy levels ?
	Cult. Göttingen ^1^	*2.96* ± *0.05*			Cult. Göttingen ^1^	*19.6* ± *1.6*	
					*Kessler 3514* ^1^	*20.3* ± *2.6*	
					*Kessler 3642* ^1^	*19.5* ± *1.3*	
					*Kessler 3501* ^9^	15.4 ± 1.8	
					*Kessler 3518* ^9^	13.1 ± 1.5	
					*Kessler 3519* ^9^	18.6 ± 2.2	
*P.tarapacana*	Cult. Göttingen ^1^	*3.02* ± *0.17*	Cult. Göttingen ^1^	~*80*	Cult. Göttingen ^1^	*17.4* ± *1.8*	tetraploid / x = 12
	Cult. Göttingen ^1^	*3.00* ± *0.16*			Cult. Göttingen ^1^	*16.9* ± *2.3*	
					*Kessler 3599* ^1^	*17.4* ± *2.2*	
					*Kumar 6* ^1^	*17.1* ± *1.1*	
					*Beck 9008* ^9^	14.9 ± 2.8	
					*Beck 19897* ^9^	16.1 ± 1.1	
					*Beck 32470* ^9^	15.0 ± 1.2	
					*Boza 3009* ^9^	14.9 ± 2.3	
					*Kessler 3599* ^9^	19.7 ± 2.0	
*P.tomentella*	43 ind. Ar ^7^	*2.90–3.01*			*Kessler 3188* ^1^	*17.9* ± *1.8*	tetraploid / x = 12
					*Kessler 3368* ^1^	*18.7* ± *2.0*	
					*Boza 3107* ^9^	16.6 ± 1.6	
					*Boza 3110* ^9^	13.9 ± 2.0	
					*Boza 3111* ^9^	15.3 ± 1.4	
					*Kessler 3200* ^9^	19.1 ± 2.5	

Assigning ploidy levels to species of *Polylepis* can be based on two different base numbers. Segovia-Salcedo (2014) and Zurita et al. (2013) used the base haploid chromosome number of 7 in the family Rosaceae as reference, thus interpreting a chromosome count of 42 as hexaploid (x = 6). Schmidt-Lebuhn et al. (2010) and Kessler et al. (2014) instead used the lowest number in the genus (2n = 42) as baseline, interpreting this as functionally diploid. The evolution of chromosome numbers in angiosperms is highly complex, with repeated polyploidization events commonly followed by reductions in chromosome numbers via aneuploidy and dysploidy (de Wet 1971; Adams and Wendel 2005; Tate et al. 2005; Jiao et al. 2011; Weiss-Schneeweiss et al. 2013). We consider that, ultimately, within a plant group, the crucial factor is the behaviour of the chromosomes, i.e. whether they behave as bivalents so that during chromosome pairing each chromosome has a single counterpart or as polyvalents where they can pair with several other chromosomes. This behaviour is unknown for *Polylepis* or related genera. For simplicity, we here use a base chromosome number of 2n = 42 as baseline for defining diploids.

Based on this approach, for guard cell length, based on comparison with chromosome counts and genome size measurements, Boza Espinoza et al. (2020b) proposed that diploidy is related to guard cell lengths of 9–15 µm, tetraploidy to 14–20 µm and higher ploidy levels to 18–23 µm, showing that some overlap occurs that may make it difficult to assign a specific measure to a ploidy level. For genome size, the diploid condition is related to 2C values around 1.4–1.7 pg, triploidy to 2.0–2.3, tetraploidy to 2.6–3.4 pg, hexaploidy to 4.6–4.9 pg and octoploidy to 5.7–5.8 pg. Finally, chromosome counts of around 42 correspond to diploids, around 84 to tetraploids and around 126 to hexaploids. However, numerous published counts differ notably from these values. For example, Caiza et al. (2018) and Segovia-Salcedo and Quijia-Lamiña (2014) reported chromosome counts of 59–77 for nine individuals of *P.ochreata* (as *P.sericea*) from Yanacocha, Ecuador. In this situation, it is unclear if these numbers reflect the difficulty of fully counting the tiny chromosomes or whether they correspond to real values with would suggest triploidy and other intermediate chromosome levels as a result of aneuploidy and dysploidy.

At present, for the 45 species of *Polylepis*, combined data on guard cell length, chromosome number and genome size are available for nine (20%) species, on guard cell length and genome size for another nine (20%) species and on guard cell length and chromosome number for three (7%) species, whereas for 24 (53%) species, only guard cell measurements are available (Table 1). Bearing in mind the potential limitations of incomplete data and some uncertainty in the interpretation of the data, we infer that, at present knowledge, 19 (42%) species are purely diploid, 15 (33%) purely tetraploid and one (2%) purely octoploid. The remaining eight (18%) species have mixed ploidy levels, with three (7%) being di- and tetraploid, two (4%) di- and hexaploid, one (2%) tetra- and hexaploid, one (2%) tetra- and octoploid and one (2%) di-, tri-, tetra- and hexaploid. While it is likely that further studies will reveal more cases of mixed ploidy, at least some well-studied species appear to consistently show diploid (*P.hieronymi*, *P.lanuginosa*, *P.simpsoniae*) or tetraploid (*P.tarapacana*, *P.tomentella*) conditions.

Placing the ploidy levels in an evolutionary context, in section Sericeae, most species are diploid, but mixed di- and polyploidy is present in *P.ochreata* and *P.pauta*. These two species overlap in northern Ecuador where they hybridize extensively and it is conceivable that the polyploid condition stems from this hybridization, as polyploidy is often correlated with hybridization (de Wet 1971; Tate et al. 2005).

In section Reticulatae, again most species are diploid, but polyploidy occurs in *P.microphylla* and *P.reticulata*. Interestingly, at least in *P.reticulata*, polyploidy is only known only from cultivated plants (Segovia-Salcedo 2014), suggesting that this condition may be related to domestication.

In section Australes, *P.australis* has been well studied and includes di-, tri-, tetra- and hexaploids, most likely due to autopolyploidiation (Kessler et al. 2014). These ploidy levels show a clear geographical distribution pattern, with populations from the northern Argentinean Andes being purely diploid and those from the central Andes tetraploid, whereas in the isolated Sierra de Córdoba, all four ploidy levels co-occur in mixed populations. This suggests that the triploid plants may be hybrids between the di- and tertraploid ones, but whether they are sterile primary hybrids or can reproduce by themselves is unresolved. Additionally, the degree of reproductive isolation and, hence, evolutionary independence between the diploid and tetraploid populations remains unknown.

Sections *Subsericantes* and *Incanaee* mainly includes tetraploid species. These sections on average occur at higher elevations and in more arid environments than the other sections, which corresponds well with the polyploid condition, since polyploids are well known to be over-represented at high latitudes and elevations (Masterson 1994; Brochmann et al. 2004), possibly because the different paralogs offer more adaptive potential (Chung et al. 2010). In contrast to the dominant tetraploidy in these sections, both *P.fjeldsaoi* and *P.incana* are diploid. *Polylepisincana* has been considered to be one of the most derived species in section Incanaee (Simpson 1979, 1986; Schmidt-Lebuhn et al. 2006a), so that a diploid condition is surprising if one assumes that *P.incana* is nested within a tetraploid clade. This suggests that the assumption that *P.incana* is a derived member of this section is wrong and that the evolution of this section is more complex than previously assumed. In any case, assuming that the sections recognized here, based on morphological and ecological similarity, are evolutionary units, we now can deduce that polyploidy evolved at least five times in the genus, possibly more often. However, considering that the evolution of *Polylepis* is probably reticulate (Kerr 2004; Schmidt-Lebuhn et al. 2006a), inferring the origins of polyploidy in *Polylepis* may be very difficult.

Finally, focusing on the taxonomic implications of ploidy levels in the genus, we found that, in some cases, closely related species have different ploidy levels, supporting their treatment as distinct species. For example, *P.fjeldsaoi* has previously been identified as *P.tomentella* (Mendoza and Cano 2012), but whereas the first species is diploid, the latter is consistently tetraploid. On the other hand, at least eight species include individuals of different ploidy levels. At least in *P.australis*, this is clearly a natural condition (Kessler et al. 2014), which raises the question as to how to treat the different ploidy levels taxonomically. It has been suggested that different ploidy levels within a “species” should be treated at species level if there is morphological, ecological or biogeographical evidence that they are evolutionarily largely independent units (Soltis et al. 2007). This approach has been taken in polyploid-apomict species complexes of other genera of Rosaceae, such as *Crataegus* (Talent and Dickinson 2007) and *Sorbus* (Robertson et al. 2010), but more information is needed before this approach can be applied to *Polylepis*. On the other hand, in several species, polyploidization is apparently linked to cultivation, as in *P.incana*, *P.racemosa* and *P.reticulata*. Polyploidization of cultivated plants is a common phenomenon either via auto- or allopolyploidization where higher ploidy levels are often associated with higher plant vigour and adaptive potential (Paterson 2005; Matsuoka 2011; Sattler et al. 2016). *Polylepis* has long been planted by Andean inhabitants as a source of building material, firewood and as fences (Kessler 1995d) and it is conceivable that natural or artificial hybrids have been favoured. For a more detailed discussion of this situation in *P.racemosa*, see under that species.

### ﻿Phylogenetic reconstructions

*Polylepis* belongs to the tribe Sanguisorbeae DC., which is characterized by cup-shaped hypanthium that entirely encloses the carpel(s), resulting in a perigynous position of the flower (Focke 1894; Robertson 1974; Eriksson et al. 2003; Potter et al. 2007). *Polylepis* has long been placed in close taxonomic proximity to *Acaena*, a genus of some 100 species of mainly evergreen, creeping herbaceous perennial plants and subshrubs found mostly in New Zealand, Australia and South America, with a few species extending to Hawai’i and California. Previously, Bitter (1911) argued for a derivation of *Polylepis* from a species group in *Acaena* that includes *A.elongata* because this group is characterized by multipinnate leaves and long dense racemes similar to those of *Polylepis*. Bitter (1911) also implicitly considered that the most ancestral species of *Polylepis* may be *P.multijuga* because it has long dense racemes and spine-covered fruits reminiscent of those of *Acaena*.

Simpson (1979, 1986) provided the first explicit ideas about the evolution of *Polylepis*, placing the species in three informal groups. The first of these groups, the *sericea* group (here section Sericeae), includes species with putatively ancestral traits such as large, multipinnate leaves with thin texture, and long inflorescences with numerous flowers. Most species of this group grow at relatively low and often humid conditions at the upper margin of the cloud forest, where *Acaena* also occurs. The second group is the *reticulata* group (our section Reticulatae), characterized by nitid, emarginated leaflets with pannose undersides of the leaflets. The species of this group occur at higher elevations than those of the first group and often also in more arid habitats. Finally, the large *incana* complex (our sections *Australes*, *Subsericantes* and *Incanaee*) is morphologically quite variable (which is why we placed the species in three sections), but includes many species with just one leaflet pair, densely pannose or even glandular lower leaflet surfaces and highly reduced inflorescences. While some species occur at low elevations and in humid regions, many are found in rather arid habitats and often at very high elevations. Simpson (1986) concluded that *Polylepis* likely originated from *Acaena* under humid cloud forest conditions and from there, gradually expanded its ecological amplitude to reach highly arid and cold environments of the high Andes, not accessible to any other native tree genus. This evolutionary trend was paralleled by morphological changes including reductions in growth height, leaflet size, number of leaflets, inflorescence length and number of flowers and an increase in leaf thickness. In the next study commenting on the evolution of the genus, Kessler (1995a) largely followed Simpson’s views.

The first attempt to study the evolutionary history of *Polylepis* using molecular methods was undertaken by Malin Kerr in an unfortunately largely unpublished PhD thesis (Kerr 2004). Using the chloroplast markers *trnL/F* and *Ahd1*, as well as a nuclear ITS locus of over 50 accessions of *Polylepis*, 26 accessions of *Acaena* and numerous other Rosaceae, she was able to confirm that *Polylepis* is nested within *Acaena*, rendering the latter genus paraphyletic. Based on her limited sampling, Kerr (2004) hypothesized that *Polylepis*, as a whole originated, from a hybridization event between ancestral members of the *Acaenaelongata* and *A.cylindristachya* species groups. She hypothesized that this hybridization event was probably allopolyploid, as suggested by her understanding that *Acaena* has a predominant ploidy level of 2n = 42 in *Acaena*, whereas *Polylepis* in her view has 2n = 84. However, we now know that 2n = 42 is also common and presumably ancestral in *Polylepis* (see Ploidy levels). Still, morphologically *Polylepis* combines traits of both the *A.elongata* group (spiny fruits) and the *A.cylindristachya* group (racemose inflorescences), supporting the idea of a hybrid origin of *Polylepis*. However, to complicate matters, Kerr (2004) also found indication that *P.quadrijuga* later hybridized with a member of AcaenasectionAncistrum (to which neither *A.elongata* nor *A.cylindristachya* belong). However interesting, the conclusions of Kerr (2004) were based on limited sampling and the use of the nuclear marker ITS which is notorious for having multiple copies, especially in polyploidy plants, potentially hampering phylogenetic reconstructions (Baldwin et al. 1995).

The second attempt at a molecular phylogenetic reconstruction of *Polylepis* was undertaken by Schmidt-Lebuhn et al. (2006a), based on 46 accessions of *Polylepis* and two accessions of *Acaena* using Amplified Fragment Length Polymorphisms (AFLP) of primarily the nuclear genome. Their results were quite similar to those obtained by Kerr (2004): very limited resolution within the genus and strong geographical clustering of the accessions, to the degree that samples of the same species would cluster with geographically proximate samples of other species rather than with their conspecifics. Combining this data with a morphological data matrix, they nevertheless obtained a phylogenetic hypothesis that clustered similar species in meaningful groups. These groups largely corresponded to Simpson’s (1979, 1986) classification: a basal grade corresponding to the *sericea* group (our section Sericeae), a monophyletic *reticulata* group (section Reticulatae), and a monophyletic *incana* group (sections *Australes*, *Subsericantes* and *Incanaee*) with *P.subsericans* as sister to the other species in the group. The latter species was placed by Simpson (1979) in her *sericea* group, but she commented on the intermediacy of the species between the *sericea* and *incana* groups and we here place it in a section of its own, together with two other species.

The latest molecular study of the phylogeny of *Polylepis* was conducted by M. Claudia Segovia S. in a PhD thesis that also remains largely unpublished (Segovia-Salcedo 2014). She used Next Generation Sequencing (Hyb-Seq) to analyze 256 nuclear genes and chloroplastic genomes of 25 *Polylepis* accessions. Her phylogenetic reconstruction showed a strong geographical signal and recovered groups of morphologically very different species that did not correspond to the groups proposed by Simpson (1979, 1986) and Schmidt-Lebuhn et al. (2006a). As Segovia-Salcedo (2014) did not include replicate samples of widespread species, it is impossible to assess whether samples of the same species from different geographical areas would be recovered close to each other in a phylogenetic reconstruction.

In conclusion, our understanding of the evolutionary history of *Polylepis* is still very incomplete. While morphological traits point to a plausible story of diversification and adaptation from humid cloud forests to arid high-elevation habitats, molecular data suggest a complex, reticulate evolutionary history. Additionally, while there is evidence that *Polylepis* is nested within *Acaena*, we refrain from merging both genera until a clearer picture of their evolutionary relationships emerges.

### ﻿Implication for species delimitation

Based on all the above, we can conclude that there is ample gene flow between populations of *Polylepis* assigned to different species. Although species can be distinguished on morphological, biogeographical and ecological grounds, it is likely that gene flow between populations of different species in close proximity have more gene flow between them than geographically remote populations of the same species. At the same time, the presence of different ploidy levels in at least eight species of the genus suggests that there may be barriers to gene flow within species.

In such a situation, the classical biological species concept of species being reproductively and evolutionarily independent units is hardly applicable (Mayr 2000). *Polylepis* may, thus, be a case where a genic species concept, rather than a genomic species concept is more appropriate. The genic species concept (Wu 2001) assumes that species identity is based on relatively few gene regions that determine the fundamental physiological or morphological traits that define a species and that the remainder of the genome can be exchanged between species without compromising species identity. In this view, species identity is not dependent on full reproductive isolation, but rather is determined by selection on relatively few genes. In contrast, the genomic species concept is based on the traditional view of reproductive isolation between species, leading to genetic divergence across the entire genome (Lexer and Widmer 2008). For *Polylepis*, all of this remains speculative and detailed genomic studies are needed to confirm the speciation mode in the genus.

## ﻿Ecology

*Polylepis* may well be the ecologically best-studied Andean tree genus. This is because it reaches the highest elevations of any tree genus in the Andes and because *Polylepis* forests are among the most threatened ecosystems in the neotropics. In the following, we briefly review some aspects of the physiology, ecology, and biogeography of *Polylepis* as they are relevant for our monographic work.

### ﻿Physiological adaptations

*Polylepis* typically forms the uppermost forest belt in the tropical Andes, although some species also grow at lower elevations in mixed forest stands with other tree genera. Due to the high elevations and accordingly low temperatures at which these trees occur, they have been the focus of a number of ecophysiological studies aiming to understand the adaptations to low temperatures. However, since the 45 species inhabit a wide range of habitats, ranging from moderate to extremely high elevations and arid to superhumid environments, the different species express a wide range of physiological adaptations that go beyond only adaptations to low temperatures.

The ability to tolerate the low nocturnal temperatures that are typical of tropical mountains is, in some species, achieved by daily osmotic adjustments and supercooling down to -9 °C as in *P.sericea* or freezing tolerance as in *P.australis* and *P.tarapacana* (Rada et al. 1996, 2001, 2009; Azócar et al. 2007). In the latter two species, leaf tissue freezes at temperatures between -3.5 °C and -9.2 °C, but frost injury is only observed at temperatures between -18 °C and -24 °C.

Adaptations to drought conditions are also frequent in *Polylepis* and include small, thick leaflets with wax layers and sunken stomata to reduce transpirational water loss (Macek et al. 2009). This is most clearly seen in *P.tarapacana* (García-Núñez et al. 2004; Toivonen et al. 2014). In arid areas at high elevations, solar radiation is very intense, leading to the development of protective pigments (González et al. 2007).

Photosynthetic rates of *Polylepis* range from 3 μmol·m^-2^·s^-1^ in *P.tarapacana* to 9 μmol·m^-2^·s^-1^ in *P.australis* (Azócar et al. 2007). Species inhabiting relatively low elevations with higher temperatures have higher mass-based maximum photosynthesis, stomatal conductance and leaf area than species from the higher and colder habitats (Toivonen et al. 2014). Conversely, species from humid habitats, where water stress is low, but where clouds and fog often reduce insolation, have higher light use efficiency and lower light saturation and compensation points (Toivonen et al. 2014).

Generally speaking, physiological traits in *Polylepis* are related either to the temperature or precipitation conditions at which they grow, revealing evolutionary specialization and adaptation of the species along environmental gradients.

### ﻿Reproduction and growth

*Polylepis* is wind-pollinated. Although most pollen is deposited at close distances to the trees (Kuentz et al. 2007), pollen flight of up to 80 km has been documented (Seltmann et al. 2009a), suggesting extensive gene flow between populations (see Pollination biology and seed dispersal). In *P.australis*, there is increased mortality in offspring resulting from short-distance crosses and increased vigour (N-metabolism capacity) in long-distance crosses, providing evidence for inbreeding depression (Seltmann et al. 2009b).

The seeds of *Polylepis* are not well adapted for long distance dispersal. Species in section Sericeae have spines that allow them to be transported in the fur of animals, but nothing is known about dispersal distances. In many other species, the nutlets have spiny ridges that do not appear to be adapted to any specific dispersal type (Simpson 1986) and which mostly accumulate close to the parent trees. Only *P.australis* and *P.neglecta* (section Australes) have thin wings apparently adapted to wind dispersal, but flight distances are only up to a few dozen meters (Renison et al. 2004).

*Polylepis* seeds typically have low germination rates, possibly associated with dormancy (Cuyckens et al. 2021) and seed germination is temperature-dependent, with maximum germination at about 20 °C in *P.besseri* (Gareca et al. 2012), which is relatively high for high mountain environments. Accordingly, regeneration by seeds often decreases with low temperatures at high elevations (Byers 2000; Hoch and Körner 2005; Gareca et al. 2007; Hertel and Wesche 2008; Wesche et al. 2008). This may be a result of decreasing germination, but also of reduced seed production (Cierjacks et al. 2008). In arid habitats, such as northern Chile, successful reproduction may only occur in exceptionally favourable (humid and warm) years, leading to the formation of age cohorts (Rundel et al. 2003).

The species of *Polylepis* are generally rather slow-growing, with radial growth rates typically in the order of 1 mm per year, although much variation exists (Domic and Capriles 2009; Jomelli et al. 2012; Montalvo et al. 2018). On the other hand, under optimal conditions, young trees can achieve height increments of up to 50 cm per year, especially in *P.racemosa*, which is the most commonly cultivated species because of its fast growth (Rosero et al. 2018). Tree height in *Polylepis* commonly decreases with elevation, but, in arid valleys, can also decrease at low elevations due to drought (Cierjacks et al. 2008; Hertel and Wesche 2008; Kessler et al. 2014). However, tree height is also influenced by more local conditions, so that, in arid regions, shaded (and hence more humid slopes) have taller trees (Kessler et al. 2007). In many forests, tree height is also limited by human extraction of tall trees (Toivonen et al. 2011; Kessler et al. 2014).

Dendrochronological studies on *P.tarapacana* in Bolivia suggest tree longevity of at least 700 years, with precipitation being positively and high summer temperatures (which increase drought stress) negatively related to radial growth (Argollo et al. 2004; Morales et al. 2004). Growth rates in this species decrease with elevation, but are higher in sheltered microhabitats (Domic and Capriles 2009). In *P.besseri*, radial growth is limited by the accumulation of reserves the year before ring formation, with a warm period before the growing season (humid period) increasing growth (Gareca et al. 2010). In *Polylepisaustralis*, tree vitality and growth are highest in the middle of the elevational distribution of the species, being limited at low elevations by high temperatures and drought, and at high elevations by low temperatures (Marcora et al. 2008). This species has lower growth when surrounded by rocks (Suarez et al. 2008). Such local control of growth has also been observed in *P.pepei* in Bolivia, where it is influenced by slope and substrate (moraine or scree slope) (Jomelli et al. 2012).

### ﻿Habitat associations

One the most conspicuous patterns in the distribution of *Polylepis* forests is that, frequently, they occur as isolated forest patches isolated from the closed treeline (Herzog 1923; Troll 1959; Hueck 1962; Vareschi 1970; Kessler 2002). It has long been debated whether this is a natural or human-induced pattern. Especially, earlier authors have argued that these patches occur in microclimatically favoured habitats, especially boulder slopes (Raimondi 1874; Weberbauer 1907, 1930; Herzog 1923; Troll 1929; Herrera 1943; Barreda 1951; Koepcke 1961; Cerrate 1979; Arnal 1983; Salgado-Laboriau et al. 1984; Ferreyra 1986). Walter and Medina (1969) proposed that boulder slopes allow warm air to penetrate deeper into the soil, allowing for better root development. However, soil temperature measurement by Kessler and Hohnwald (1998) have shown that boulder slopes are actually colder than adjacent fine-grained soils, as also known from other mountain regions (Wakonigg 1996). As an alternative explanation, Ellenberg (1958) proposed that rocky ground prevents the spread of fires and thus allows the survival of forests. The negative influence of fires has been documented by subsequent studies (Lægaard 1992; Kessler 2000; Kessler et al. 2014). Often, this also leads to a concentration of *Polylepis* stands to sheltered ravines and along watercourses (Weberbauer 1911; Troll 1959; Salgado-Laboriau et al. 1984; Rauh 1988).

Current understanding of this so-called “*Polylepis*-problem” suggests that the natural vegetation pattern would be a grassland-forest mosaic, with increasing grassland contribution towards higher elevations (Toivonen et al. 2011, 2018; Sylvester et al. 2014a, 2017). Nevertheless, because of the paucity of undisturbed habitats, it is not known which conditions determine the development of forest or grassland in these mosaics. Factors may involve water saturation of soils, cold air ponding or strong winds (Kessler 1995d; Sparacino et al. 2020). It is known, however, that unless fires further push the forests back (Kessler 2000), the expansion of forests into grasslands is very slow due to the low ability of *Polylepis* to colonize dense grasslands (Cierjacks et al. 2007a), suggesting that the forest-grassland mosaics are fairly stable over time.

Other habitat associations of *Polylepis* include a preference for cloud condensation belts in arid regions (Troll 1959; Koepcke 1961; Lauer 1982; Cabido and Acosta 1985) and avoidance of salty soils (Kessler 1995d). Beyond these general patterns, there are many species-specific habitat preferences. For instance, whereas most species of *Polylepis* form the canopy layer of the forests, *P.hieronymi* is a successional species that often colonizes raw soils on landslides and road banks and is then displaced by taller trees, such as *Podocarpusparlatorei* (Kessler 1995b).

The upper limit of the growth of *Polylepis* forests has been a matter of some debate. Jordan (1980) claimed that *P.tarapacana* reaches 5200 m on Volcán Sajama, but subsequent studies have highest trees (3 m tall) at 4820 m, with only shrubby plants reaching 5000 m (Hoch and Körner 2005). Similar elevations are reached by *P.subsericans* in Cuzco, Peru (S. Sylvester, pers. comm.) and *P.weberbaueri* in Ancash, Peru (T. Boza, pers. obs.). Along with *Juniperustibetica* forests at 4900 m in Tibet, these are the world’s highest forests (Miehe and Miehe 1994).

### ﻿History of *Polylepis* forest cover

It is generally accepted that the genus *Polylepis* evolved in the Andes from the genus *Acaena* (Simpson 1986). The age of the genus is unknown, but the oldest available pollen records show that it was already present 370,000 bp (years before present) in Peru and Bolivia (Hanselman et al. 2011) and 124,000 bp on the Bogotá Plateau, Colombia (van der Hammen and Hooghiemstra 2003). These and other more recent pollen records show that the pollen abundances of *Polylepis* varied substantially over time, suggesting that forest cover changed over time even prior to the arrival of humans about 12,000 bp. These fluctuations have been linked to climatic changes, with warm and wet periods being correlated with maximum *Polylepis* abundance (Gosling et al. 2009). Opposed to this, natural fires, especially during dry periods, reduced *Polylepis* cover. *Polylepis* forest cover was also patchy due to landscape heterogeneity with, for instance, flat valley bottoms with water-logged soils being devoid of tree cover (Gosling et al. 2009; Valencia et al. 2018). During arid and cold climatic phases, forests were, thus, restricted to climatic microrefugia, such as sheltered valleys (Valencia et al. 2016). Similar forest-grassland mosaics can currently be found in topographic refugia inaccessible to humans and their livestock (Sylvester et al. 2014a, 2017). That some *Polylepis* species had a natural fragmented distribution is also evidenced by geographic patterns of genetic diversity, as, for example, in *P.tarapacana* (Peng et al. 2015).

The arrival of humans increased fire frequencies, first by hunter-gatherers and later by agropastoralists with their livestock, leading to widespread reductions in *Polylepis* abundance (e.g., Graf 1979, 1981, 1983, 1992; Hansen et al. 1984; Ybert and Miranda 1984; Markgraf 1985; Hansen 1992; Baied and Wheeler 1993; Chepstow-Lusty et al. 2005; Urrego et al. 2011; Williams et al. 2011). For example, in Cochabamba, Bolivia, at a site where today *P.lanata* and *P.pepei* occur in small patches above the closed treeline, *Polylepis* forests were more widespread some 18,000–14,500 a bp, declining towards some 10,000 a bp, followed by a period almost without *Polylepis* until 6,400 a bp, followed by slight increase to current levels (Williams et al. 2011). These changes in the abundance of *Polylepis* were linked to changes in fire frequency, partly due to dryer climatic conditions and partly due to human activities.

In southernmost Ecuador, at the border with Peru, *Polylepis* populations are currently restricted to tiny populations, but pollen evidence shows that *Polylepis* was much more common in the early to mid-holocene (about 11,000–4,000 bp) (Rodríguez and Behling 2012; Villota and Behling 2013). Later, as climatic conditions became more humid and human impact led to increasing fire frequencies, *Polylepis* declined massively, being replaced by mixed humid montane forest and grassland.

The development of human cultures in the Andes took an important step forward some 6,000 bp with the domestication of camelids (Wing 1986; Wheeler 1985, 1988). The development of large-scale pastoralism led to the widespread ecosystem degradation (van der Hammen and Noldus 1985; Thompson et al. 1988). During the Incan period, up to 30 million people may have inhabited the central Andes (Earls 1976). An important component of this land use was the development of agroforestry (Chepstow-Lusty and Jonsson 2000; Chepstow-Lusty et al. 2009; Chepstow-Lusty 2011; Mosblech et al. 2012), including reforestation with *Alnus* in the last 1000 years (Chepstow-Lusty et al. 1998). After the Spanish conquista, forest destruction picked up again and continues until today in many regions (Jameson and Ramsay 2007).

### ﻿Biotic interactions

*Polylepis* forests harbour unique biodiversity, including a number of highly specialized, often range-restricted and threatened bird species (Fjeldså 1993; Cahill and Matthysen 2007; Lloyd 2008a; Astudillo et al. 2020; Quispe-Melgar et al. 2020). However, these birds generally do not influence the trees themselves, except for the Thick-billed Siskin (*Spinuscrassirostris*), which eats the seeds of *Polylepis* without dispersing them (Herzog et al. 2003).

*Polylepis* forests also provide habitats for many plant species, including the world’s highest vascular epiphytes (Sylvester et al. 2014b) and a diverse liverwort flora (Gradstein and León-Yañez 2020; Gradstein and Pócs 2021). Nothing is known of competitive or facilitative interactions with other plant species in the germination, seedling or growth stages. However, many populations of *Polylepis* species are parasitized by *Tristerixchodatianus* (Loranthaceae), a specialist hemiparasite that infects trees of the genus *Polylepis* (Amico et al. 2007). In *P.flavipila* forests, up to 48% of the trees can be infected, which leads to the death of branches about 15 years after infestation and, in heavily infested trees, eventually to the death of the whole tree (Arizapana et al. 2016; Camel et al. 2019b).

Fungal interactions are also important in *Polylepis* (Robledo and Renison 2010). *Polylepisaustralis* has vesicular arbuscular mycorrhizal symbionts (Menoyo et al. 2007, 2009; Soteras et al. 2013). The same is presumably true for all other species, but this remains to be studied. *Polylepistarapacana* is parasitized by the fungus *Paraleptosphaeria* (= *Leptosphaeria*) *polylepidis* (Leptosphaeriaceae, Pleosporales), which appears to lead to increased tree mortality (Macía et al. 2005; Coca-Morante 2012). Interestingly, this fungus, in turn, is infected by the mycoparasite *Sajamaeamycophila* (Dictyosporiaceae, Pleosporales), which may perhaps be used as a biocontrol of *P.polylepidis* (Piątek et al. 2020). In *P.tomentella*, species of three genera of Ascomycota have been found in stigmas and styles, which appears to negatively affect the germination of pollen grains by inhibiting pollen tube growth, although the fungi apparently are not able to penetrate the ovary (Domic et al. 2017).

## ﻿Conservation

*Polylepis* forests represent one of the most endangered habitats in the high Andes (Hensen 2002; Servat et al. 2002; Rundel et al. 2003; Navarro et al. 2005; Kessler and Schmidt-Lebuhn 2006; Cierjacks et al. 2008; Mendoza and Cano 2012; Boza Espinoza et al. 2019; Segovia-Salcedo et al. 2021). It has been estimated that over 90% of *Polylepis* forests have already been lost in Peru and Bolivia (Kessler 1995d; Fjeldså and Kessler 1996). Major threats caused by human activities include logging, chronic overgrazing and burning (Fjeldså 2002b; Renison et al. 2002; Boada and Campaña 2008; Cierjacks et al. 2008; Navarro et al. 2010; Renison et al. 2011; Renison et al. 2013; Sylvester et al. 2016) and more locally the expansion of the agricultural frontier and mining activities (Rundel et al. 2003; Guerrero 2009; Sornoza-Molina et al. 2018). These activities have taken place long before the arrival of European conquerors and likely have affected Andean ecosystems for millennia (Kessler and Driesch 1993; Kessler 1995d). On the other hand, the Andes are expected to undergo severe changes in the coming decades as a result of on-going land-use change and climate change (Malcolm et al. 2006; Beaumont et al. 2011), likely threatening *Polylepis* forests even more. *Polylepis* forests have a low ability to colonize dense grasslands and low seed dispersal ability, severely limiting the ability of the forests to spread and to track climatic conditions (Cierjacks et al. 2007a). Fragmentation of *Polylepis* forests often, but not always, leads to loss of genetic diversity, which is even evident from one generation to the next (Julio et al. 2008, 2011; Aragundi et al. 2011; Hensen et al. 2012; Quinteros-Casaverde et al. 2012; Gareca et al. 2013; Peng et al. 2015, 2017). As a result of all the above, *Polylepis* forests have been listed as one of the most endangered woodlands ecosystems in the world (UNEP-WCMC 2004) and the conservation of the remaining forests stands has been given high priority (Kessler 1995d; Fjeldså and Kessler 1996; Cierjacks et al. 2008).

Conservation of *Polylepis* forests is not only relevant for the trees themselves. The forests are rich in endemics species and represent hotspots of biodiversity (Fjeldså and Kessler 1996; Fjeldså and Hjarsen 1999; Sevillano-Ríos and Rodewald 2017). Many studies have been dedicated to the floristics, structure, distribution and biology of *Polylepis* forests in Ecuador, Peru and Bolivia (e.g., Hensen 1993, 1995; Kessler 1995a, b; Fjeldså and Kessler 1996; Cierjacks et al. 2007a; Cierjacks et al. 2007b; ECOAN 2005, 2006, 2007; Jameson and Ramsay 2007; Ames-Martínez et al. 2019). Additionally, the unique bird communities associated with *Polylepis* forests have been studied in much detail (e.g. Fjeldså 1987; Frimer and Nielsen 1989; Fjeldså 1992; Fjeldså and Kessler 2004; Lloyd 2008a, b; Lloyd and Marsden 2008).

In this context, the evaluation of the conservation status and the degree of threat to the species is necessary in order to successfully focus conservation action. Although the current online version of the IUCN Red List of Threatened Species (http://www.iucnredlist.org/) presents assessments of species of the genus *Polylepis*, it includes barely 15 species, with 14 species listed as “Vulnerable” (VU) and one as “Near Threatened”. Bearing in mind the novel taxonomic arrangement proposed here, we present a global assessment of the conservation status for the 45 species of *Polylepis*, applying the IUCN Criteria and Categories (Table 3). As a result, we categorize over four fifths of the species of *Polylepis* as threatened, with almost half of the species categorised as “Vulnerable” (47%), 24% as “Endangered” and 18% as “Critically Endangered”, but only 9% as “Least Concern” and 2% as “Near Threatened”.

**Table 3.** Conservation status of *Polylepis* species. Abbreviations: Ar = Argentina, Bo = Bolivia, Ch = Chile, Co = Colombia, Ec = Ecuador, Pe = Perú, Ve = Venezuela.

Species	Status	Criteria	Country	Conservation Areas
**Section Sericeae**				
**Subsection Lanuginosae**				
*P.lanuginosa*	EN	B1a+B2a, C1	Ec	Cajas National Park
*P.multijuga*	CR	A1, B1a+B2a, C1	Pe	None
**Subsection Pauta**				
*P.pauta*	VU	A1, B1a+B2a, C1	Ec	Cayambe-Coca National Park
				Antisana Ecological Reserve
*P.longipilosa*	CR	A2a, B1a+B2a, C1+C2	Ec	El Angel Ecological Reserve
*P.serrata*	VU	B1a+B2a, C1	Pe	Río Abiseo National Park
				Manu National Park
**Subsection Sericeae**				
*P.albicans*	VU	B1a+B2a	Pe	Huascarán National Park
*P.argentea*	VU	B1a+B2a	Pe	Otishi National Park
*P.canoi*	EN	B1a+B2a, C1	Bo, Pe	Otishi National Park
*P.frontinensis*	CR	B2ac, C2a	Co	Colibrí del Sol Private Reserve
*P.humboldtii*	CR	B2a, C2	Ec	Sangay National Park
*P.longipilosa*	CR	A2a, B1a+B2a, C1+C2	Ec	El Angel Ecological Reserve
*P.loxensis*	CR	A2a, B1a+B2a, C2a	Ec	None
*P.ochreata*	VU	B1a+B2a, C1	Co, Ec	El Angel Ecological Reserve (Ec)
				Yanacocha Reserve (Ec)
*P.sericea*	VU	B1a+B2a	Co, Ve	Sierra Nevada National Park (Ve)
				Sierra de la Culata National Park (Ve)
**Subsection Pepea**				
*P.pepei*	EN	A2a, B1a+B2a, C1, D1	Bo, Pe	Madidi National Park (Bo)
				Carrasco National Park (Bo)
*P.rodolfo-vasquezii*	VU	B1a+B2a, C1	Pe	Pui-Pui Protection Forest
**Section Reticulatae**				
*P.hieronymi*	VU	B1a+B2ac	Ar, Bo	Cordillera de Sama Biological Reserve (Bo)
*P.microphylla*	EN	B1a+B2ab	Ec, Pe	Sangay National Park (Ec)
				Cordillera Huayhuash Reserved Zone (Pe)
*P.occidentalis*	EN	A1, B1a+B2a, C1	Pe	None
*P.quadrijuga*	VU	A2a, B1a+B2a, D2a	Co	Chingaza National Natural Park
				Cocuy National Park
				Sumapaz National Natural Park
*P.reticulata*	VU	B1a+B2a, C1	Ec	Cajas National Park
				Llanganates National Park
				Pasochoa Ecological Reserve
				Yacuri National Park
*P.simpsoniae*	EN	A1, B1a+B2a, C2a	Ec	Cajas National Park
*P.weberbaueri*	VU	B1a+B2a	Pe	Huascarán National Park
**Section Australes**				
*P.australis*	LC	B1a+B2a	Ar	Quebrada del Condorito National Park
*P.neglecta*	VU	A1,2a, B1a+B2a, C2a	Bo	None
***Section Subsericantes***				
*P.flavipila*	EN	B1a+B2a, C2a	Pe	Nor Yauyos-Cocha Landscape Reserve
*P.pilosissima*	CR	A2a, B2a	Pe	Japani Private Conservation Area
*P.subsericans*	VU	B1a+B2a	Pe	Vilcanota Private Conservation Areas Network
**Section Incanaee**				
**Subsection Racemosae**				
*P.acomayensis*	EN	A2a, B1a+B2a, C2a	Pe	None
*P.incarum*	CR	A1, B1a+B2a, C1+C2a	Bo, Pe	None
*P.lanata*	VU	B1a+B2a, D2a	Bo	Carrasco National Park
*P.pacensis*	EN	A2b, B1a+B2a, C1	Bo	None
*P.racemosa*	LC	B1a+B2a	Ec, Pe	None
*P.sacra*	VU	B1a+B2a	Pe	Vilcanota Private Conservation Areas Network
*P.triacontandra*	EN	A1, B1a+B2a, C1	Bo, Pe	Apolobamba Integrated Management Natural Area (Bo)
**Subsection Besseria**				
*P.besseri*	VU	A1, B1a+B2a, C1	Bo	None
*P.crista-galli*	VU	A1, B1a+B2a, C2a	Ar, Bo	None
*P.pallidistigma*	VU	B1a+B2a	Pe	None
*P.rugulosa*	VU	B1a+B2a, C1	Ch, Pe	Lauca National Park (Ch)
				Islunga National Park (Ch)
				Las Vicuñas National Reserve (Ch)
				Salinas y Aguada Blanca National Reserve (Pe)
*P.subtusalbida*	VU	A1, B1a+B2a	Bo	Tunari National Park
**Subsection Incanaee**				
*P.fjeldsaoi*	VU	B1a+B2a, C2a	Pe	None
*P.incana*	LC	A1, B1a+B2a	Co, Ec, Pe	Cajas National Park (Ec)
				Illinizas Ecological Reserve (Ec)
				El Angel Ecological Reserve (Ec)
				Huascarán National Park (Pe)
				Cordillera Huayhuash Reserve Zone (Pe)
*P.incanoides*	EN	A1+A2a, B1a+B2a, D1	Bo	None
*P.nana*	CR	A1+A2a, B1a, C1+C2a, D2a	Bo	None
*P.tarapacana*	NT	A1+A2a, B1a+B2a, C1	Ar, Bo, Ch, Pe	Sajama National Park (Bo)
*P.tomentella*	LC	A1, B1a+B2a, C2a	Ar, Bo	None

Most of the species of *Polylepis* are present in at least one protected area, but 16 species have no such conservation actions taken to date. However, actual protection of the species in conservation areas leaves much to be desired. In many cases, no specific conservation actions are taken, in others, extractive activities continue within protected areas. Even where reforestation schemes are undertaken, these are often counterproductive, since alien species of *Polylepis* are used which can hybridize with the native species. For instance, *P.racemosa* is not native to Ecuador, but has been widely planted and is already hybridizing with the native species.

More generally, there is mixed success of protected areas in conservations terms which may show the limitations of strictly conservationist approaches that fail to take into consideration the needs of local human population (Joseph et al. 2021). Identification of development alternatives that reduce firewood collection and free-ranging ranching and implementation of a sustainable development plan are measures that may help to save these unique vegetation types. Such attempts have been undertaken, for example, for *P.australis* in Argentina, where restoration activities are tightly coupled with detailed research activities (Renison et al. 2002, 2004, 2005, 2006, 2010, 2011; Teich et al. 2005; Menoyo et al. 2007; Seltmann et al. 2007, 2009a, b; Julio et al. 2008, 2011; Suarez et al. 2008; Torres et al. 2008; Zimmermann et al. 2009; Giorgis et al. 2010; Peng et al. 2017). Since 2018, the NGOs Andean Ecosystem Association (ECOAN) and Global Forest Generation (GFG) have joined forces to create long-term alliances with partners in Colombia, Ecuador, Peru, Bolivia, Argentina and Chile within the Andean Action initiative. This initiative strengthens leadership capabilities and enhances the conservation, research and restoration of *Polylepis* forests through collective work and stable commitment to local communities and authorities. The goal is to restore and protect one million hectares of high Andean forests through effective and remunerable actions. This clearly is a pivotal time for the conservation of *Polylepis* forests, with increasing threats and increasing conservation activities.

## ﻿Taxonomic treatment

### ﻿ Polylepis

Taxon classificationPlantaeRosalesRosaceae

Ruiz & Pavón (1794:80)

14224528-8B0D-5290-8317-C4A21BCF6D4A

#### Type.

*Polylepisracemosa* Ruiz & Pavón.

#### Description.

***Trees or shrubs*** 1–32 m tall; bark shredding, multilayered with thin exfoliating layers; branches twisted showing a striking arrangement of the leaves, young shoots with leaves usually all closely clustered at the top, causing a shrub-like growth, while the basal internodes stretch rather significantly afterwards. ***Leaves*** alternate, imparipinnate with 1–7 pairs of lateral leaflets, obtrullate in outline, 1.3–19.5 cm long, 0.6–10.7 cm wide; rachises lanate, pannose, sericeous, tomentose, villous or glabrous; point of leaflet attachment with a tuft of long hairs; stipular sheaths apically acute, truncate or spurred, glabrous to densely covered with lanate, tomentose or villous hairs on the other surface; leaflets elliptic, ovate or obovate in outline, 0.3–5.4 cm long, 0.2–2.0 cm wide; margins entire, revolute, crenate to serrate, apically acute to deeply emarginate, attenuate, cuneate or unequally cordate, upper leaflet surfaces glabrous or sparsely to densely lanate, pilose, sericeous or tomentose; lower leaflet surfaces covered with very short pannose hairs, pannose mixed with another type of hairs or sparsely to densely lanate, pilose, sericeous, strigose, tomentose or villous. ***Inflorescences*** axillar, simple rarely branched, upright or pendant to 36.0 cm long, bearing 1–83 flowers; floral bracts 2.1–15.8 mm long, narrowly triangular. ***Flowers*** hermaphroditic; sepals 3–4; stamens 5–27, anthers orbicular with a dense tuft of straight white hairs in the upper half; styles fimbriate, 0.9–4.9 mm long, ovary inferior; carpel 1, ovule 1. ***Fruit*** indehiscent achene, fusiform turbinate with protuberances, flattened-spines, irregular flattened ridges, thick wings and ridges or thin wings, glabrous to densely sericeous, tomentose or villous, 1.7–15.1 mm long, 1.3–10.1 mm wide including spines.

### ﻿Key to species of the genus *Polylepis*

**Table:** 

1	Lateral leaflets 1 pair	**2**
–	Lateral leaflets 2–7 pairs	**21**
2	Lower leaflet surfaces densely pannose, hairs < 0.2 mm long	**3**
–	Lower leaflet surfaces sparsely to densely pilose, sericeous, strigose, tomentose or villous, hairs 0.4–2.0 mm long	**10**
3	Leaflet margins crenate	**4**
–	Leaflet margins entire or serrate	**6**
4	Stipular sheaths apically acute, outer sheath surfaces densely villous	***P.incana***
–	Stipular sheaths apically truncate, outer sheath surfaces glabrous to densely villous	**5**
5	Leaflets obovate; upper leaflet surfaces and rachises tomentose; central Peru	***P.fjeldsaoi***
–	Leaflets elliptic; upper leaflet surfaces and rachises villous; southern Peru	***P.pallidistigma***
6	Leaflets 0.7–1.2 cm long; inflorescences 0.7–1.5 cm long	**7**
–	Leaflets 1.3–3.2 cm long; inflorescences 2.2–8.0 cm long	**8**
7	Leaflets 1.0–1.2 cm long; upper leaflet surfaces often with dark sheen, glabrous to sparsely villous; central Bolivia	***P.nana***
–	Leaflets 0.7–0.8 cm long; upper leaflet surfaces rugose, glabrous, usually covered with a layer of yellowish resinous exudate; south-western Peru, north-western Chile, western Bolivia and north-western Argentina	***P.tarapacana***
8	Stipular sheaths with outer surfaces densely tomentose; 13–15 stamens per flower; fruits with 2–4 wide flattened hard irregular ridges, sparsely tomentose	***P.crista-galli***
–	Stipular sheaths with outer surfaces glabrous to densely villous; 15–23 stamens per flower; fruits with 3–4 ridges with a variable number and placement of flattened spines, densely villous	**9**
9	Leaflets 0.5–0.7 cm wide, with 7–15 teeth per side; leaflet apices obtuse to slightly acute; upper leaflet surfaces glabrous; 5–7 flowers per inflorescence; 15–19 stamens per flower; Dpto. Cochabamba (Bolivia)	***P.incanoides***
–	Leaflets 0.3–0.6 cm wide, with 5–10 teeth per side, leaflet apices round to emarginate; upper leaflet surfaces glabrous to sparsely villous; 4–5 flowers per inflorescence, 19–23 stamens per flower; Dptos. Potosi, Oruro, Chuquisaca, Tarija (Bolivia) and Jujuy (Argentina)	***P.tomentella***
10	Leaflet margins serrate	**11**
–	Leaflet margins entire or crenate	**13**
11	Lower leaflet surfaces sparsely to densely tomentose without underlying short hairs; 7–21 flowers per inflorescence	***P.racemosa***
–	Lower leaflet surfaces densely tomentose with a dense underlying layer of very short white hairs; 3–7 flowers per inflorescence	**12**
12	Leaflets 0.7–1.3 cm wide; leaflet apices acute; inflorescences 5.1–7.5(–8.9) cm long; Titicaca basin (Puno, Peru; La Paz, Bolivia)	***P.incarum***
–	Leaflets 0.4–0.6 cm wide; leaflet apices obtuse to emarginate; inflorescences 1.8–3.7 cm long; Depts. Cochabamba and north-western Potosi (Bolivia)	***P.subtusalbida***
13	Leaflets 0.9–2.2 cm long; leaflet apices deeply emarginate without projection; Ecuador	***P.reticulata***
–	Leaflets 0.9–3.3 cm long; leaflet apices round, obtuse, acute or emarginate with a mid-vein projection; Ecuador, Peru, Bolivia, Chile	**14**
14	Leaflet margins entire	**15**
–	Leaflet margins crenate	**16**
15	Leaflets 0.9–1.1 × 0.4–0.6 cm; upper leaflet surfaces glabrous to sparsely sericeous; lower leaflet surfaces sparsely to densely sericeous; inflorescences 0.9–1.1 cm long, with 1 flower	***P.rodolfovasquezii***
–	Leaflets (1.3–)1.7–2.8 × 0.5–0.7 cm; upper leaflet surfaces sparsely strigose; lower leaflet surfaces densely strigose; inflorescences (1.9–)2.5–4.9(–5.6) cm long, with 3–6 flowers	***P.subsericans***
16	Lower leaflet surfaces densely tomentose with a dense underlying layer of very short, white pannose hairs	**17**
–	Lower leaflet surfaces densely pilose, villous or tomentose without pannose hairs	**18**
17	Upper leaflet surfaces smooth to slightly rugose; lower leaflet surface hairs 0.6–0.8 mm long; 7–9 flowers per inflorescence; central Bolivia	***P.besseri***
–	Upper leaflet surfaces strongly rugose; lower leaflet surface hairs 0.8–1.0 mm long; 4–5 flowers per inflorescence; south-western Peru and north-western Chile	***P.rugulosa***
18	Upper leaflet surfaces sparsely to densely pilose; 3–5 flowers per inflorescence; 11–17 stamens per flower	**19**
–	Upper leaflet surfaces glabrous to sparsely villous; 5–13 flowers per inflorescence; 19–23 stamens per flower	**20**
19	Lower leaflet surfaces densely pilose, hairs 0.5–0.6 mm long	***P.flavipila***
–	Lower leaflet surfaces densely villous, hairs 1.0–1.2 mm long	***P.pilosissima***
20	Leaflet apices round to emarginate; upper leaflet surfaces sparsely villous; lower leaflet surfaces densely villous, hairs 0.9–11 mm long; inflorescences 2.0–4.0 cm long, with 5–7 flowers; south-central Peru	***P.acomayensis***
–	Leaflet apices acute; upper leaflet surfaces glabrous; lower leaflet surfaces densely tomentose, hairs 0.4–0.8 mm long; inflorescences (4.9–)5.5–7.0(–9.5) cm long, with 11–13 flowers; southern Peru and northern Bolivia	***P.triacontandra***
21	Lateral leaflets 4–7 pairs	**22**
–	Lateral leaflets 2–3 pairs	**38**
22	Lower leaflet surfaces glabrous to puberulous; fruits with 2–3(–4) irregular and pronounced, thin wings	***P.neglecta***
–	Lower leaflet surfaces sparsely or densely lanate, sericeous, villous or tomentose, hairs 0.3–2.3 mm long; fruits with variable numbers of flattened or long, hard spines	**23**
23	Leaflets 0.3–0.7 cm long; leaflet apices deeply emarginate; inflorescences 3.8–5.3 cm long, with 1–3 flowers	***P.microphylla***
–	Leaflets 1.1–5.4 cm long; leaflet apices slightly emarginate to emarginate; inflorescences 2.4–36.0 cm long, with 4–83 flowers	**24**
24	Lower leaflet surfaces sparsely or densely sericeous	**25**
–	Lower leaflet surfaces densely lanate, villous or tomentose	**31**
25	Leaflet margin entire or serrate	**26**
–	Leaflet margin slightly crenate to crenate	**28**
26	Leaflets (0.8–)1.1–1.5 cm wide; lower leaflet surface hairs 1.3–1.7 mm long	***P.canoi***
–	Leaflets 0.4–0.9 cm wide; lower leaflet surface hairs 0.2–0.9 mm long	**27**
27	Leaflets broadly obovate; upper leaflet surfaces glabrous with few hairs on the mid-vein	***P.loxensis***
–	Leaflets narrowly elliptic to elliptic; upper leaflet surfaces glabrous to sparsely sericeous	**28**
28	Lateral leaflet 3–4 pairs	**29**
–	Lateral leaflet 4–7 pairs	**30**
29	Leaflet margins slightly crenate at the apex; inflorescences 3.9–6.6(–7.8) cm long, with 18–21 flowers; northern Peru	***P.albicans***
–	Leaflet margins entire; inflorescences 13.0–17.9(–20.4) cm long, with 23–29 flowers; central Ecuador	***P.humboldtii***
30	Leaflets 1.6–3.0 cm long; leaflet margins entire to slightly serrate; lower leaflet surfaces of mature plants with hairs 0.3–0.5 mm long; north-western Ecuador and southern Colombia	***P.ochreata***
–	Leaflets (1.1–)1.4–1.6 cm long, leaflet margins crenate; lower leaflet surfaces of mature plants with hairs 0.3–0.4 mm long; north-eastern Ecuador	***P.pauta***
31	Lower leaflet surfaces densely lanate or villous, the hairs 0.7–1.8 mm long	**32**
–	Lower leaflet surfaces densely tomentose, the hairs 0.3–1.5 mm long	**35**
32	Leaflet margin entire to slightly crenate; northern Ecuador	***P.longipilosa***
–	Leaflet margins serrate; northern Colombia, Peru	**33**
33	Leaflets 0.4–0.8 cm wide, obovate; leaflet apices slightly emarginate; 7–15 flowers per inflorescence; north-western Colombia	***P.frontinensis***
–	Leaflets 0.8–2.0 cm wide, elliptic; leaflet apices acute or obtuse; 16–83 flowers per inflorescence; Peru	**34**
34	Leaflets 1.1–2.0 cm wide; leaflet apices obtuse; lower leaflet surfaces densely villous; inflorescences (15.4–)21.7–28.2(–36.0) cm long, with 47–83 flowers	***P.multijuga***
–	Leaflets 0.8–1.0(–1.2) cm wide; leaflet apices acute; lower leaflet surfaces densely lanate; inflorescences (7.6–)9.5–13.3(–17.3) cm long, with 16–35 flowers	***P.serrata***
35	Leaflet apices moderately emarginate; upper leaflet surfaces sparsely tomentose; Bolivia and Argentina	***P.hieronymi***
–	Leaflet apices deeply emarginate; upper leaflet surfaces glabrous; Colombia, Ecuador and Peru	**36**
36	Lateral leaflets (1–)2–3(–4) pairs; lower leaflet surface hairs 0.6–1.5 mm long; Ecuador	***P.reticulata***
–	Lateral leaflets 3–5 pairs; lower leaflet surface hairs 0.3–0.9 mm long; Colombia and Peru	**37**
37	Leaflets 0.5–0.8 cm wide; lower leaflet surface hairs 0.3–0.6 mm long; inflorescences 2.4–6.7 cm long, with 4–12 flowers; northern Peru	***P.occidentalis***
–	Leaflets 0.7–1.1 cm wide; lower leaflet surface hairs 0.7–0.9 mm long; inflorescences (6.0–)7.3–10.5(–12.3) cm long, with 11–19 flowers; north-eastern Colombia	***P.quadrijuga***
38	Lower leaflet surfaces glabrous to sparsely hispid, puberulous, or pannose, the hairs < 0.2 mm long	**39**
–	Lower leaflet surfaces sparsely to densely lanate, sericeous, tomentose or villous, the hairs 0.3–2.5 mm long	**42**
39	Leaflets 0.5–0.8 cm wide; leaflet margins crenate, with 5–8 teeth per side; lower leaflet surfaces glabrous to sparsely villous; southern Peru	***P.pallidistigma***
–	Leaflets 0.4–1.5 cm wide; leaflet margins serrate with 9–18 teeth per side; lower leaflet surfaces glabrous; Argentina and Bolivia	**40**
40	Leaflet margins with 12–18 teeth per side; leaflet apices acute; 14–27 flowers per inflorescence; Argentina and Bolivia	***P.neglecta***
–	Leaflet margins with 9–13 teeth per side; leaflet apices obtuse to emarginate; 5–12 flowers per inflorescence	**41**
41	Leaflets elliptic, lower leaflet surfaces glabrous to sparsely hispid; fruits with 2–3 irregular and pronounced thin wings, glabrous; Argentina	***P.australis***
–	Leaflets obovate, lower leaflet surfaces pannose; fruits with 2–4 wide flattened hard irregular ridges, sparsely tomentose; Argentina and Bolivia	***P.crista-galli***
42	Lower leaflet surfaces densely lanate, the hairs 1.3–2.5 mm long	**43**
–	Lower leaflet surfaces sparsely to densely sericeous, tomentose or villous, the hairs 0.3–2.0 mm long	**45**
43	Leaflets elliptic; upper leaflet surfaces glabrous to sparsely lanate; lower leaflet surface hairs 1.5–2.5 mm long, yellowish; 13–16 flowers per inflorescence; Ecuador	***P.lanuginosa***
–	Leaflets obovate to broadly obovate; upper leaflet surfaces sparsely lanate; lower leaflet surface hairs 1.3–1.5 mm long, whitish; 5–11 flowers per inflorescence; Peru and Bolivia	**44**
44	Leaflets (1.8–)2.2–2.7 × 0.9–1.4 cm; inflorescences (5.0–)6.1–4.9(–12.3) cm long; Bolivia	***P.lanata***
–	Leaflets 1.6–2.6 × 0.6–1.1 cm; inflorescences 5.0–8.8 cm long; Peru	***P.sacra***
45	Lower leaflet surfaces densely sericeous, the hairs 0.2–1.7 mm long	**46**
–	Lower leaflet surfaces sparsely to densely tomentose or villous, the hairs 0.4–2.0 mm long	**52**
46	Leaflets 0.8–1.3 × 0.2–0.7 cm; inflorescences 1.2–1.6(–3.5) cm long, with 3 flowers; southern Peru and Bolivia	***P.pepei***
–	Leaflets 1.2–3.9 × 0.4–1.5 cm; inflorescences 3.3–20.4 cm long, with 5–29 flowers; Venezuela to Bolivia	**47**
47	Leaflets (2.4–)3.4–3.9 × (0.8–)1.1–1.5 cm; lower leaflet surface hairs 1.3–1.7 mm long, yellowish; central Peru to Bolivia	***P.canoi***
–	Leaflets 1.2–2.8 × 0.4–1.0 cm; lower leaflet surface hairs 0.2–1.0 mm long, silky, whitish; Venezuela to central Peru	**48**
48	Lateral leaflets 3–5 pairs; lower leaflet surface hairs 0.2–0.6 mm long; Ecuador and north-western Peru	**49**
–	Lateral leaflets 2–3 pairs; lower leaflet surface hairs 0.6–1.0 mm long; Venezuela, Colombia, central Peru	**51**
49	Leaflet margins entire; inflorescences 13.0–17.9(–20.4) cm long, with 23–29 flowers	***P.humboldtii***
–	Leaflet margins slightly crenate at the apex or serrate; inflorescences 3.5–12.2 cm long, with 9–27 flowers	**50**
50	Leaflets elliptic; leaflet margins slightly crenate at the apex; north-western Peru	***P.albicans***
–	Leaflets broadly obovate; leaflet margins serrate; southern Ecuador	***P.loxensis***
51	Leaflets (1.9–)2.4–2.6 × 0.5–0.7 cm; upper leaflet surface sparsely to dense sericeous; inflorescences 7.2–8.1 cm long; central Peru	***P.argentea***
–	Leaflets 1.8–2.1 × 0.8–1.0 cm; upper leaflet surfaces glabrous; inflorescences 3.3–4.5 cm long; Venezuela and Colombia	***P.sericea***
52	Leaflet margins serrate	**53**
–	Leaflet margins entire to crenate	**55**
53	Lateral leaflets 3–4(5) pairs; lower leaflet surface densely villous; Colombia	***P.frontinensis***
–	Lateral leaflets 1–2 pairs; lower leaflet surfaces sparsely to densely tomentose; Ecuador, Peru and Bolivia	**54**
54	Leaflets (2.3–)3.1–3.9 × (0.7–)0.9–1.5 cm, apices round; inflorescences (4.2–)5.0–9.4(–11.7) cm long, with 7–21 flowers; Ecuador and Peru	***P.racemosa***
–	Leaflets 0.9–1.6 × 0.4–0.6 cm, apices obtuse to emarginate; inflorescences 1.8–3.7 cm long, with 3–4 flowers; Bolivia	***P.subtusalbida***
55	Leaflet apices acute, round, obtuse or emarginate; lower leaflet surfaces densely tomentose or villous with a dense underlying layer of very short pannose hairs; Peru and Bolivia	**56**
–	Leaflet apices moderately to deeply emarginate; lower leaflet surfaces densely tomentose without short pannose hairs; Ecuador to Argentina	**59**
56	Lower leaflet surface hairs 0.9–1.1 mm long; inflorescences 2.0–4.0 cm long; central Peru	***P.acomayensis***
–	Lower leaflet surface hairs 0.4–0.9 mm long; inflorescences 3.6–10.0 cm long; southern Peru to Bolivia	**57**
57	Leaflets (2.0–)2.6–3.3 × 0.7–1.1 cm, narrowly elliptic; 11–13 flowers per inflorescence; Dptos. Puno (Peru) and La Paz (Bolivia)	***P.triacontandra***
–	Leaflets 1.4–2.4 × 0.6–11 cm, narrowly obovate; 5–11 flowers per inflorescence; La Paz and Cochabamba (Bolivia)	**58**
58	Lateral leaflets 1–2(–3) pairs; stipular sheaths apically truncate; rachises and lower leaflet surfaces densely villous; Dpto. Cochabamba (Bolivia)	***P.besseri***
–	Lateral leaflet 2–3 pairs; stipular sheaths apically acute; rachises and lower leaflet surfaces densely tomentose; Dpto. La Paz (Bolivia)	***P.pacensis***
59	Leaflets (0.9–)1.3–1.6 × (0.4–)0.7–1.1 cm, broadly ovate; Ecuador	***P.simpsoniae***
–	Leaflets 0.9–2.2 × 0.4–1.1 cm, elliptic to obovate; Colombia to Argentina	**60**
60	Leaflets narrowly obovate; upper leaflet surfaces sparsely tomentose; Bolivia and Argentina	***P.hieronymi***
–	Leaflets narrowly elliptic to obovate; upper leaflet surfaces glabrous; Colombia to Peru	**61**
61	Lower leaflet surface hairs 0.3–0.6 mm long; Peru	**62**
–	Lower leaflet surface hairs 0.6–1.5 mm long; Colombia and Ecuador	**63**
62	Lateral leaflets 3–5 pairs; leaflets 1.1–1.9 cm wide; inflorescences 2.4–6.7 cm long	***P.occidentalis***
–	Lateral leaflets 2–3 pairs; leaflets 1.6–2.1 cm wide; inflorescences 8.6–9.7 cm long	***P.weberbaueri***
63	Lower leaflet surface hairs 0.7–0.9 mm long; inflorescences (6.0–)7.3–10.5(–12.3) cm long, with 11–19 flowers; north-eastern Colombia	***P.quadrijuga***
–	Lower leaflet surface hairs 0.6–1.5 cm long; inflorescences (2.3–)4.3–13.8 cm long, with 5–9 flowers; Ecuador	***P.reticulata***

### ﻿ Polylepis sect. Sericeaee

Taxon classificationPlantaeRosalesRosaceae

 T. Boza & M. Kessler, sect. nov.

680C4686-B388-5D0A-9D12-76FFE5BA8E03

urn:lsid:ipni.org:names:77301574-1

#### Diagnosis.

Trees or shrubs; lower leaflet surfaces with sericeous, lanate or villous hairs; fruits with a variable number and placement of flattened, thin or short spines, densely sericeous or villous.

#### Type.

*Polylepissericea* Wedd.

#### Notes.

The sectional epithet *Sericeae* is a plural adjective agreeing in gender with *Polylepis*. This section, already defined as a group by Simpson (1986), includes species that usually have sericeous hairs on the lower leaflet surfaces and/or stipule sheaths and whose leaves usually contain many pairs of leaflets. Furthermore, fruits in this section are turbinate with a variable number of slender spines. However, not all species share these traits. For instance, *P.frontinensis* and *P.multijuga* have villous hairs on the lower leaflet surfaces, but *P.frontinensis* has sericeous hairs on the stipule sheaths and *P.multijuga* has many leaflets pairs (5–7). In the same way, *P.lanuginosa* and *P.serrata* have lanate hairs, but both have many lateral leaflets pairs. *Polylepismultijuga*, *P.ochreata*, *P.pauta* and *P.serrata* are the species with most leaflet pairs (4–7), whereas *P.rodolfovasquezii* just has one pair. Moreover, *P.pepei* and *P.rodolfovasquezii* have turbinate, but slightly twisted fruits with short spines. This variation may reflect that this section is likely not monophyletic, but rather has been proposed to represent a basal grade within the genus (Schmidt-Lebuhn et al. 2006a).

The majority of species in this section are morphologically clearly distinct. Probably the two most similar species are *P.pepei* and *P.rodolfovasquezii*, which only differ in a few, partly overlapping characters. They might be treated at subspecies level, but as detailed in the Introduction, we decided not to accept infraspecific taxa because of the difficulty of deciding at which level to discriminate between species- and subspecies-level differentiation. Table 4 provides an overview of the arrangement of the taxa by different authors.

**Table 4.** Alignment of the species of the Polylepissect.Sericeae according to Bitter (1911), Simpson (1979), Segovia et al. (2018) and the present study.

Bitter (1911)	Simpson (1979)	Segovia et al. (2018)	This study
*P.albicans*	*P.sericea*	*P.sericea*	*P.albicans*
*P.ochreata*			*P.ochreata*
*P.hypargyrea*			*P.sericea*
*P.sericea*			
			*P.argentea*
			*P.frontinensis*
			*P.humboldtii*
			*P.loxensis*
—		*P.canoi*	*P.canoi*
*P.coriacea*	*P.lanuginosa*	*P.lanuginosa*	*P.lanuginosa*
*P.lanuginosa*			
*P.lehmannii*			
*P.multijuga*	*P.multijuga*	*P.multijuga*	*P.multijuga*
*P.annulatipilosa*	*P.pauta*	*P.pauta*	*P.pauta*
*P.pauta*			
*P.stuebelii*			
—			*P.longipilosa*
*P.serrata*			*P.serrata*
—	*P.pepei*	*P.pepei*	*P.pepei*
—	—	*P.rodolfo-vasquezii*	*P.rodolfo-vasquezii*

Within section Sericeae, we recognize four subsections, based on their morphological distinctness, as follows: subsection Lanuginosae (two species) with lanate or villous lower leaflet surfaces and densely villous fruits; subsection Pauta (three species) with 4–6 lateral leaflet pairs and lanate or sericeous lower leaflet surfaces; subsection Sericeae (eight species) with sericeous lower leaflet surfaces (except *P.frontinensis*) and fruits with flattened spines; and subsection Pepea (two species) with 1–2 lateral leaflet pairs, emarginate leaflet apices and densely sericeous, slightly twisted fruits with short spines.

##### Climatic niches in Polylepissect.Sericeae

**Figure 12.**
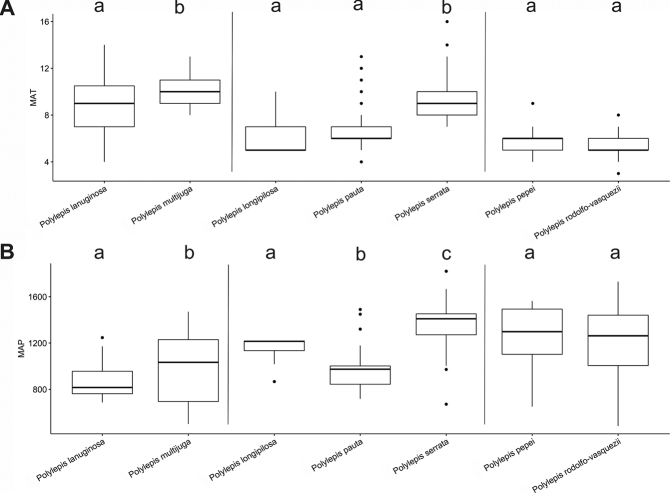
Box plots showing the climatic niches of the species of the subsections *Lanuginosae*, *Pauta* and *Pepea* in relation to Mean Annual Temperature (MAT) (**A**) and Mean Annual Precipitation (MAP) (**B**). The ends of each box represent the upper and lower quartiles and the median is indicated with a bold line inside the box; the whisker lines extend to the highest and lowest observations, except when observations are higher or lower than the interquartile range (i.e. outliers), in which case they are indicated by a dot. Box plots that share the same lowercase letters within each subsection are not significantly different at p = 0.05. Vertical lines represent subsectional divisions.

**Figure 13.**
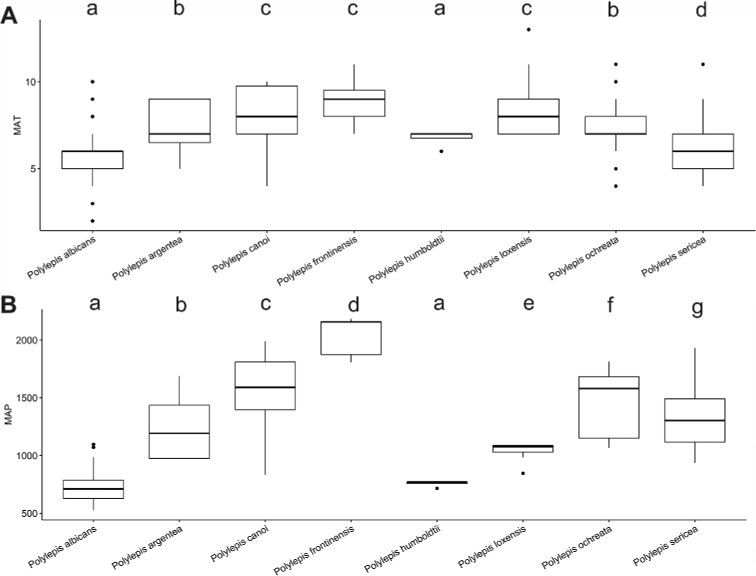
Box plots showing the climatic niches of the species of subsection Sericeae in relation to MAT (**A**) and MAP (**B**). See Fig. 12 for details on data presentation.

Many species of this section differ markedly in their climatic niches (Figs 12 and 13). For example, *Polylepisalbicans* and *P.sericea* from subsect. Sericeae and *P.pepei* and *P.rodolfovasquezii* from subsect. Pepea grow under the coldest conditions (about 5.5 °C Mean Annual Temperature – MAT), whereas other species, such *P.multijuga* (10.0 °C) from subsect. Lanuginosae and *P.serrata* (9.6 °C) from subsect. Pauta, grow under noticeably higher temperatures. These differences of 4–5 °C correspond to 800–1000 m in elevation. Regarding Mean Annual Precipitation (MAP), *P.frontinensis* grows under the most humid conditions (mean of 2048 mm MAP), followed by *P.canoi* (1572 mm). In contrast, *P.albicans* and *P.humboldtii* on average receive only 744 mm per year, whereas *P.lanuginosa* (847 mm), *P.pauta* (941 mm) and *P.multijuga* (986 mm) also grow in relatively dry conditions.

Focussing on the individual subsections, the two species in subsect. Lanuginosae show minor ecological differences and replace each other geographically. In subsect. Pauta, *P.longipilosa* and *P.pauta* from northern Ecuador have quite similar climatic niches and replace each other geographically, whereas *P.serrata* from Peru grows under substantially higher temperatures and higher precipitation. The two very similar species of subsect. Pepea have identical niches and complementary geographical distributions. These species clearly form a vicariant species pair, suggesting allopatric speciation after geographical isolation. Finally, in subsection Sericeae, there are major differences among almost all species, with only *P.albicans* and *P.humboldtii* having similar climatic niches, but these are geographically well separated. Indeed, all species of this subsection are geographical vicariants, except for *P.argentea* and *P.canoi*, which broadly overlap geographically, but have quite different niches, with *P.argentea* growing under colder and drier and *P.canoi* under warmer and more humid conditions. These marked ecological differences between species show that they are evolutionarily and ecologically independent lineages and support their treatment as separate species.

### ﻿Subsection Lanuginosae

Taxon classificationPlantaeRosalesRosaceae

T.Boza & M.Kessler subsect. nov.

90398B96-1487-55D3-A9B0-93635B4AA33D

urn:lsid:ipni.org:names:77301575-1

#### Diagnosis.

Trees; 3–6(–7) lateral leaflet pairs; lower leaflet surfaces lanate or villous; fruits with flattened spines, densely villous.

#### Type.

*Polylepislanuginosa* Kunth.

#### Notes.

The subsectional epithet *Lanuginosae* is a plural adjective agreeing in gender with *Polylepis*.

### ﻿1. Polylepis lanuginosa

Taxon classificationPlantaeRosalesRosaceae

Kunth, Nov. Gen. Sp. (quarto ed.) 6: 228. 1824.

9811E2A3-475A-51EA-8B8F-347747B11A87

Figs 14 , 15

Polylepis lehmannii Hieron. Bot. Jahrb. Syst.20: Beibl. 49: 29. 1895. Type. Ecuador. Azuay: west of Cuenca, *Lehmann 6487* (holotype: B destroyed; isotype: F!).
Polylepis coriacea Bitter Bot. Jahrb. Syst. 45: 603. 1911. Type. Ecuador. Chimborazo: Valley of Pangor *Spruce s.n* (holotype: W, photos at F!, MO!, US!).

#### Type.

**Ecuador. Chimborazo**: near Calpi “ad radicem montis Chimborazo”, June 1903, *Humboldt & Bondpland 2191* (holotype: P!; isotypes: P!, photo at F!).

**Figure 14.**
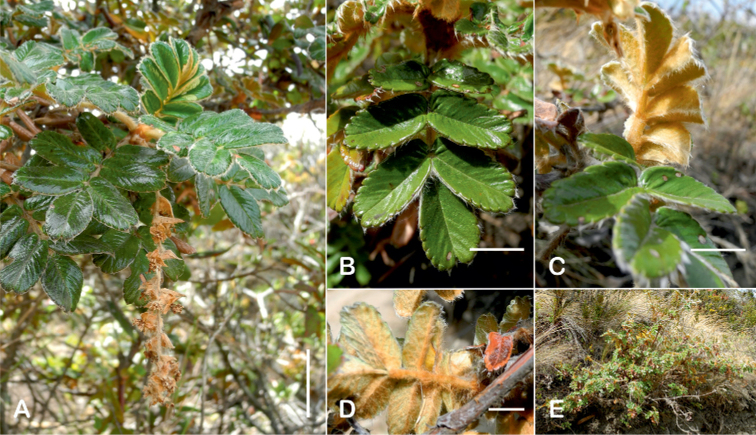
*Polylepislanuginosa* Kunth **A** flowering branch **B** upper leaf surface **C** leaves **D** lower leaf surface **E** habit. Scale bars: 2 cm (**A, C**); 1 cm (**B, D**). Photographs by T.E. Boza E.

#### Description.

***Trees*** 3–8 m tall. ***Leaves*** slightly congested at the branch tips, imparipinnate with 2–3 pairs of lateral leaflets, obtrullate in outline, (4.5–)5.2–7.7 × 3.4–4.5 cm; rachises densely villous, points of leaflet attachment with a tuft of long, lanate hairs; stipular sheaths apically acute with spurs, densely lanate on the outer surfaces; leaflets elliptic in outline, second pair from the terminal leaflet the largest, one of this pair 1.7–2.8 × 0.7–1.4 cm; margin crenate with 8–9 teeth, apically emarginate, basally unequally cordate; upper leaflet surfaces glabrous or sparsely lanate; lower leaflet surfaces densely lanate with yellowish hairs 1.5–2.5 mm long. ***Inflorescences*** branched at the base or simple, pendant, (4.3–)6.2–9.5(–13.0) cm long, bearing 13–16 flowers; floral bracts 3.8–5.5 mm long, narrowly triangular, densely lanate on the outer surface; rachises villous. ***Flowers*** 5.8–7.5 mm diam.; sepals 4, ovate, green, densely sericeous outside; stamens 13–15, anthers orbicular, with a dense tuft of straight white hairs on the upper half; styles fimbriate, 1.9–2.3 mm long. ***Fruits*** turbinate, with variable numbers and placement of flattened spines, densely villous; 4.4–6.0 × 6.1–7.4(–9.8) mm including spines. ***Diploid***.

**Figure 15.**
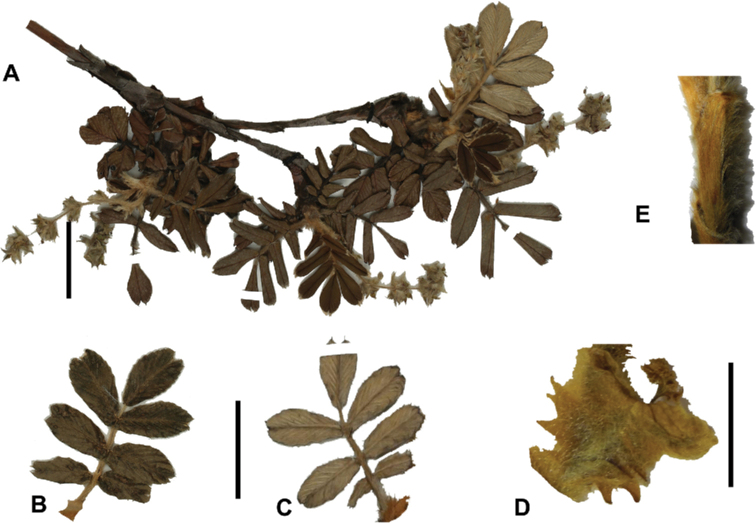
*Polylepislanuginosa* Kunth **A** flowering branch **B** upper leaf surface **C** lower leaf surface **D** fruit **E** stipular sheaths (**A***Romoleroux 584***B***Laegaard 53932***C***Harling 22858***D, E***Laegaard 55036*). Scale bars: 5 cm (**A**); 4 cm (**B, C**); 1 cm (**D**). Photographs by T. E. Boza E.

#### Distribution, habitat and ecology.

*Polylepislanuginosa* is endemic to central and southern Ecuador (Azuay, Bolívar, Cañar and Chimborazo) (Fig. 24). It occurs in Andean forests at 2300–4200 m elevation. It has been recorded within Cajas National Park where it forms patches mainly on hillsides. Among the *Polylepis* species that co-occur in this area (*P.incana*, *P.lanuginosa*, *P.reticulata*, *P.simpsoniae* and introduced *P.racemosa*), *P.lanuginosa* occupies the warmest habitats, has the largest foliar area (17.3 cm^2^) and the highest leaf mass (> 200 mg). In its forest habitat, *P.lanuginosa* is co-dominant with other tree species, such as *Oreopanaxandreanus*, *Weinmanniafagaroides* and *Sesseacorymbosa*, which often exceed it in height (Montalvo et al. 2018).

#### Conservation status.

Based on 17 collecting localities, the estimated EOO is 6,910 km^2^ and the occupied habitat or AOO is 96 km^2^. It is protected within Cajas National Park. The species was categorized as VU B1+2c by Oldfield et al. (1998) and as VU B1ab(iii) by León-Yánez et al. (2011). Small patches of *Polylepislanuginosa* growing close to roads are especially exposed to erosion and logging. We assess *P.lanuginosa* as Endangered (B1a+B2a, C1).

#### Notes.

*Polylepislanuginosa* is most similar to *P.multijuga*, with which it shares the leaflet shape and hair density. However, it differs from it and all other members of the genus by its branched inflorescences. Further, *P.lanuginosa* has crenate leaflets 1.7–2.8 cm long, whereas *P.multijuga* has serrate leaflets 2.9–5.4 cm long. In *P.lanuginosa*, the hairs on the lower leaflet surfaces are densely lanate, whereas in *P.multijuga*, they are densely villous. *Polylepislanuginosa* is also morphologically similar to *P.canoi*, but differs in its elliptic and shorter (1.7–2.8 cm long) leaflets (versus leaflets obovate and 2.4–3.9 cm long). Additionally, *P.lanuginosa* has shorter styles (1.9–2.3 mm long) than *P.canoi* (2.4–3.8 mm long).

#### Specimens examined.

**Ecuador. — Azuay**: Chaucha, Angas on western slope of western cordillera (due west of Cuenca), 02°55'S, 079°25'W, 3400 m, 05 January 1981, *Balslev 1507* (AAU!, NY, QCA!, S); Angas “Parroquia chaucha” colecciones en margenes de Río Angas, 3400 m, 02 August 1983, *Jaramillo 5464* (AAU!, NY, QCA!); *5468* (AAU!); *5478* (AAU!, MO!, NY, QCA!); Cuenca, Area Nacional de Recreación Cajas, collection made along Río Patul from the Comunidad Baute/Laguna Patul (watershed of Río Patul), 02°33'S, 079°21'W, 3500–4200 m, 05 February 2001, *Clark 6227* (QCA!, QCNE, US!); Molleturo, on the road from Las Cajas National Park to Molleturo, about 10 km from Molleturo, 02°50'S, 079°20'W, 3400 m, 19 September 1983, *Brandbyge 42264* (AAU!, MO!, QCA!); Cuenca-Molleturo road ca. 11 km W of pass in Las Cajas, 02°48'S, 079°18'W, 3350 m, 01 May 1992, *Lægaard 102637* (AAU!, QCA!); Páramo de Cajas, W of Cuenca, along new road, ca. 14 km W of pass, 02°48'S, 079°17'W, 3450 m, 31 March 1985, *Lægaard 53932* (AAU!); Páramo de Cajas, W of Cuenca, along new road, ca. 20 km W of pass, 02°48'S, 079°17'W, 2900 m, 31 March 1985, *Lægaard 53934* (AAU!, MO!, QCNE); Páramo de las Cajas, W of pass, 02°46'S, 079°15'W, 2500 m, 26 August 1985, *Lægaard 55036* (AAU!, MO!, QCA!); carretero Cuenca-Molleturo-Naranjal, 4.2 km de Molleturo, desvío a Río Blanco 16.4 km, 02°48'40"S, 079°23'07"W, 3630 m, 15 January 2003, *Ulloa 1203* (HA, MO!, US!); Sayausi, Area Recreacional Las Cajas, 02°49'S, 079°07'W, 3740–4070 m, *Romoleroux 1192* (AAU!); Zhud, at Panamericana, app. 3 km S of Zhud, 02°29'S, 079°00'W, 2800 m, 02 May 1992, *Lægaard 102697* (AAU!, QCA!); límite del parque nacional, 3359 m, 20 April 2012, *Barba BOP236* (QCA!); Río Blanco, Curiquinga, 3645 m, 05 May 2001, *Calle 1* (QCA!); Cuenca-Molleturo road, 49 km NW of Cuenca, 26 July 1982, *Clemants 2184* (AAU!, NY, QCA!); El Chorro ca. 6 km above Molleturo on road to Cuenca, 2800–2900 m, 07 March 1985, *Harling 22858* (AAU!, GB, MO!, QCA!); Molleturo, 2600–2700 m, 31 October 1988, *Harling 25539* (GB, MO!); Descente occidentale du páramo de Cajas vers Molleturo, 3350 m, 14 April 1988, *Huttel 1021* (QCA!); Vallée du río Angas, à 1 Km au-dessus du hameau d’Angas, zone trés humide, 3300 m, 10 May 1988, *Huttel 1112* (QCA!); Vertiente del Pacífico, 3200 m, 07 July 1995, *León 3601* (QCA!); Área recreacional Cajas, 3470 m, 21 September 2000, *Lizarzaburu 25* (QCA!); 3500 m, *Romoleroux 408* (NY); Parque Nacional Cajas, carretera Soldados, 3260 m, 04 April 2007, *Romoleroux 4461* (QCA!); 3040 m, 04 April 2007, *Romoleroux 4466* (QCA!); Vía Soldados Angas, al frente del caserío Angas, 3321 m, 19 August 2008, *Romoleroux 5030* (QCA!); Carretera Soldados-Angas, 3040 m, 04 April 2007, *Romoleroux GPI4466* (QCA!); Cajas, found along path from Cochapamba to Molleturo, 3500–3600 m, 22 July 1999, *Smeets 559* (QCA!); 2670–3275 m, *Steyermark 52599* (F!); 3160 m, *Valencia 458* (QCA!). — **Bolívar**: carretera Guaranda-Santiago-Totoras, 3000–3150 m, 21 February 1987, *Romoleroux 269* (AAU!, QCA!). **Cañar**: Molleturo, along Páramo road to Manu W of Cañar, W of pass, 02°33'S, 079°02'W, 3300–3700 m, 20 June 1988, *Lægaard 71563*, *71565*, *71569* (AAU!, QCNE); Molleturo, Páramo de Cajas, W of Cuenca, aong new road, ca. 11 km W of pass, 02°48'S, 079°17'W, 3200 m, 30 March 1985, *Lægaard 53911* (AAU!, MO!, QCA!); Zhud, along Panamericana, 4 km S of Zhud, 02°28'S, 079°00'W, 3000 m, 26 August 1985, *Lægaard 55030* (AAU!, MO!); along a paved road to Carshao, ca. 15 km off the Panamerican highway, 02°29'S, 079°00'W, 3180 m, 10 June 1999, *Sklenar 7115* (AAU!); carretera entre Dacur y Gun, 2250 m, 08 October 1999, *Bonifaz 3974* (QCA!); North rim of the valley of the río Cañar, between Tambo and Suscal, 3000 m, 23 April 1944, *Camp E-2773* (F!, NY, VEN); colecciones entre Zhud, Joyagshi, 3500 m, 31 December 2007, *Jaramillo 26120* (QCA!); 3300–3700 m, *Romoleroux 1563* (QCA!); 3270 m, *Romoleroux 384* (QCA!); 3100 m, *Romoleroux 387* (NY, QCA!); carretera entre Zhud y El Tumbo, 3011 m, 06 December 2007, *Romoleroux 4681* (QCA!); Oeste de Cañar, Km 10.5, Cerro Caucay, 3450 m, 27 April 1988, *Romoleroux 588* (AAU!, MO!); *Rose 2389* (NY). **Chimborazo**: Columbe, road Pallantanga-Riobamba, 01°55'S, 078°50'W, 2400–2900 m, 01 April 1993, *Romoleroux 1565* (AAU!, QCA!); Juan de Velasco, Pangor-Tepeyac, 01°48'S, 078°52'W, 3200 m, 09 February 1983, *Brandbyge 42059* (AAU!, MO!);, 3300 m, 03 May 1983, *Brandbyge 42153* (AAU!, MO!, QCA!); Colta (Cajabamba)–Pallatanga, km 27, 01°50'S, 078°53'W, 2880 m, 21 May 1990, *Jørgensen 91822* (AAU!); Km 64–68 on road Cumandá-Cajabamba, at Río Pangor, 01°55'S, 078°54'W, 2750–2800 m, 08 April 1985, *Lægaard 54125* (AAU!, MO!, QCA!), *54128* (AAU!, QCA!); road Pallatanga–Cajabamba, 32 km from Pallatanga, 01°51'S, 078°53'W, 3000 m, 28 August 1976, *Øllgaard 8959* (AAU!, MO!, NY, S). Penipe, Parroquia Puela, Palictahua, 01°31'05"S, 078°29'43"W, 2600 m, 20 January 1997, *Estudiantes ESPOCH 701* (CHEP); Colta, Pangor, puente del Río Agua Dulce, 3000 m, 31 January 2007, *Caranqui 1659* (QCA!); Colta. Pangor, Achín alto, 3140 m, 21 May 2013, *Caranqui 2290* (QCA!); Comunidad de Tauris, Zona Zagin, 3700–3900 m, 03 September 2009, *Cárate 1202* (QCA!); Comunidad de Ambrosio Lazo, Quebrada de Cumbo, 3374 m, 07 June 2009, *Cárate 623*, *624* (QCA!); Páramo cerca de la comunidad Yerba Buena, Cantón Pallatanga, 3374 m, 19 July 2012, *Peyre 315* (QCA!); 2800–3200 m, *Romoleroux 373* (NY, QCA!); Vía Cajabamba–Pallatanga (km 24 desde la Y), entrada a Pangar, 3298 m, 27 December 2011, *Romoleroux 5696* (QCA!); Vía Cajabamba-Palatanga (km 24 desde la Y), entrada al Pangar, 3298 m, 27 December 2011, *Romoleroux 5697* (QCA!); Carretera Alausi-Cañar, km 16, localidad Achupallas, 3400 m, 26 April 1988, *Romoleroux 574* (AAU!, QCA!); Cañar, 2 km al sur de Zhud, 2850 m, 27 April 1988, *Romoleroux 584* (AAU!, QCA!); 3250 m, *Romoleroux 591* (QCA!), W Andes of Cuenca, *Lehmann 6487* (F!).

### ﻿2. Polylepis multijuga

Taxon classificationPlantaeRosalesRosaceae

Pilg., Bot. Jahrb. Syst. 37: 536. 1906.

A3593890-074D-530B-907A-909C09FC989D

Figs 16 , 17

#### Type.

**Peru. Cajamarca**: at Chugur near Hualgayoc, 2700–3000 m, May 1904, *Weberbauer 4098* (holotype: G!; isotypes: MOL!; photos at F!, MO!).

**Figure 16.**
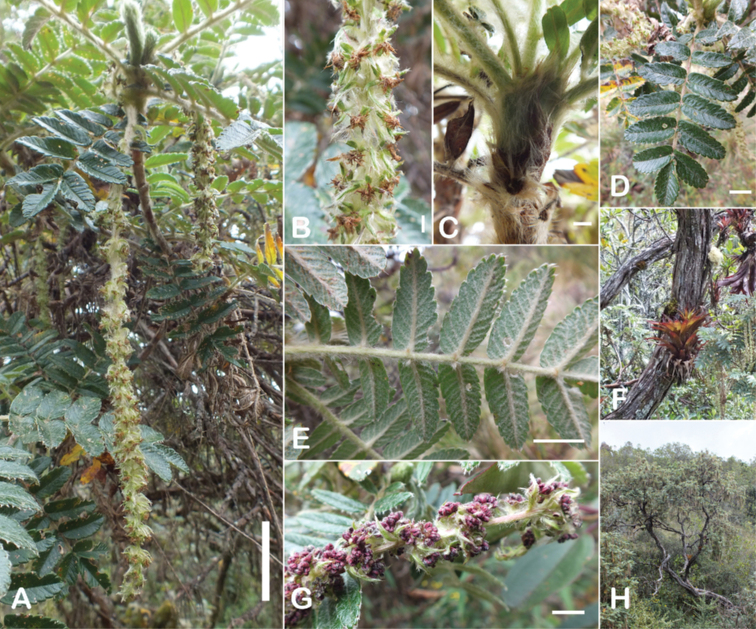
*Polylepismultijuga* Pilg **A** flowering branch **B** flowers **C** stipular sheaths **D** upper leaf surface **E** lower leaf surface **F** bark **G** flowers **H** habit (**A–H***Boza & Urquiaga 3008*). Scale bars: 4 cm (**A**); 1 cm (**B**); 2 mm (**C**); 2 cm (**D, E**); 8 mm (**G**). Photographs by E.G. Urquiaga F.

#### Description.

***Trees*** 5–15 m tall. ***Leaves*** slightly congested at the branch tips, imparipinnate with 5–7 pairs of lateral leaflets, obtrullate in outline, (11.0–)14.6–19.5 × 6.2–9.1(–10.7) cm; rachises densely lanate, points of leaflet attachment with a tuft of long, lanate hairs; stipular sheaths apically acute with spurs, densely lanate on the outer surfaces; leaflets elliptic in outline, second pair from the terminal leaflet the largest, one of this pair 2.9–3.6(–5.4) × 1.1–2.0 cm; margin serrate with 6–10 teeth, apically obtuse, basally unequally cordate; upper leaflet surfaces glabrous or sparsely villous; lower leaflet surfaces densely villous with whitish hairs 0.9–2.3 mm long. ***Inflorescences*** pendant, (15.4–)21.7–28.2(–36.0) cm long, bearing 47–83 flowers; floral bracts 7.6–9.7 mm long, narrowly triangular, densely villous on the outer surface; rachises villous. ***Flowers*** 6.4–7.5 mm diam.; sepals 4, ovate, green, densely lanate outside; stamens 7–13, anthers orbicular, with a dense tuft of straight white hairs on the upper half; styles fimbriate, 1.9–3.8 mm long. ***Fruits*** turbinate, with variable numbers and placement of irregular spines, densely villous; 4.5–9.6 × 6.0–10.1 mm including spines. ***Diploid***.

**Figure 17.**
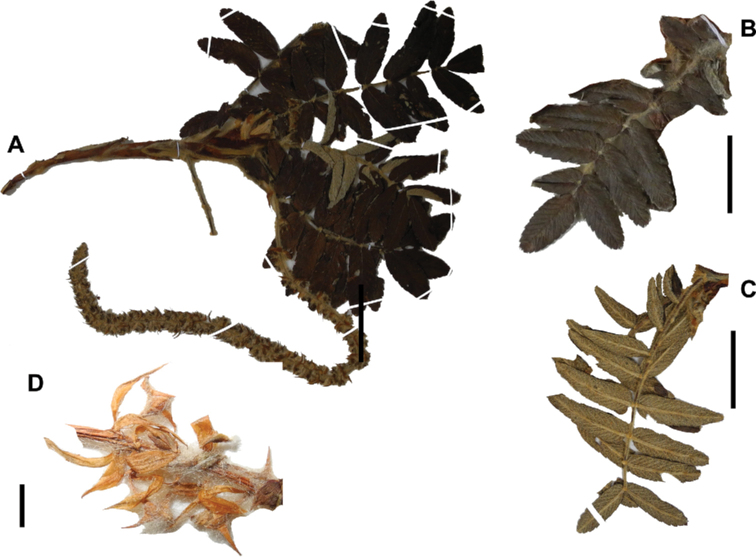
*Polylepismultijuga* Pilg **A** flowering branch **B** upper leaf surface **C** lower leaf surface **D** fruit. (**A, B***Weigend 98/330***C***Castillo 786***D***Ferreyra 20908*). Scale bars: 6 cm (**A**); 2 cm (**B, C**); 8 mm (**D**). Photographs by T. E. Boza E.

#### Distribution, habitat and ecology.

*Polylepismultijuga* is restricted to northern Peru (Fig. 24). It grows mainly in the upper montane forest at 2700–3750 m elevation, usually in mixed forest with species of *Cyathea*, *Escallonia*, *Gynoxys* and *Weinmannia* (Weberbauer 1911). Its branches are often covered with epiphytes (Simpson 1979).

#### Conservation status.

Based on 22 localities, the EOO for *Polylepismultijuga* is estimated at 17,200 km^2^ and the AOO at 116 km^2^. The species was categorized as VU (B1+2c, D2) in the World List of Threatened Trees (Oldfield et al. 1998). Later, based on its fragmented habitat affected by human disturbance, it was listed as EN (B1ab(iii)) in the Red List of Peru (SERFOR 2006; León-Yánez et al. 2011). It does not occur in any protected area and mining and forestry activities have led to its disappearance at several former locations (Guerrero 2009). Deforestation of remnant *P.multijuga* patches and extended reforestation with *Pinus*, which dries and acidifies the soils, are further reducing populations. The small, fragmented, and declining populations of this species are characterized by low genetic diversity (Quinteros-Casaverde et al. 2012). Based on its fragmented and degraded distribution, continuing habitat loss, and lack of habitat protection, we assess *P.multijuga* as Critically Endangered (A1, B1a+B2a, C1).

#### Notes.

*Polylepismultijuga* is similar to *P.canoi* but has 5–7 pairs of elliptic leaflets, whereas the latter has 2–4 pairs of obovate leaflets. *Polylepismultijuga* also has longer inflorescences (15.4–36.0 cm) with 47–83 flowers (*P.canoi* 8.2–14.5 cm, 12–26 flowers). *Polylepismultijuga* is also similar to *P.ochreata*, but differs by having larger and broader leaflets (2.9–5.4 × 1.1–2.0 cm versus 1.6–3.0 × 0.5–0.7 cm) and densely villous lower leaflet surfaces (densely sericeous in *P.ochreata*). *Polylepisochreata* also has shorter inflorescenses (8.1–17.4 cm) with fewer flowers (21–49).

#### Specimens examined.

**Peru. Amazonas**: Chachapoyas, Dist. Leymebamba, surroundings of La Esperanza, 06°49'04"S, 077°43'01"W, 3200–3300 m, 27 June 2010, *Glenn 411* (CAS, COL!, F!, K, MO!, P!); Dist. Leymebamba. Río El Jardín, 06°55'52"S, 077°43'09"W, 3370 m, 30 June 2009, *Gruhn 173* (MO!); Dist. Leymebamba, a 2 Km de la Laguna de Los Cóndores, ruta hacia Leymebamba, 2700–2950 m, 18 August 1998, *Quipuscoa 1329* (MO!); Quintecocha. Dist. Leymebamba, vicinity of guard cabin at Quintecocha, 06°51'33"S, 077°42'15"W, 3134 m, 12 July 2008, *Rothrock 239* (BRIT, MO!); Balsas, Chuquillurco, ruta a Calla Calla, 3400 m, 06 October 2001, *Sánchez 11018* (MO!); Chachapoyas-Cajamarca road, jalca de Calla-Calla, 30–37 km from Leimebamba Natural grassland, ‘Jalca’, and ‘ceja de selva’ just leaving the pass entering the Marañon valley, 06°50'S, 077°50'W, 3500–3600 m, 04 September 1983, *Smith 5037* (MO!, USM!); Leimebamba, Oseres, 06°58'05"S, 077°39'57"W, 2542 m, 22 May 2015, *Vega 257* (HAO, MO!); road Balsas to Chachapoyas, upper eastern Calla-calla slopes descending from pass, 3000–3300 m, 02 June 1998, *Weigend 98/330* (USM!). Luya, Distr. Conila-Cohechan, 06°16'25"S, 078°00'10"W, 3050 m, 23 August 2012, *Bussmann 17289* (MO!); Colcamar, 3200–3300 m, 24–26 June 1948, *Pennell 15632* (USM!). **Cajamarca**: Chota, Bosque de Pagaibamba (Ocshawilca), al oeste del Chorroblanco, entre Huambos y Querocoto, 2500 m, 18 October 1987, *Sánchez 4588* (F!); Paccha, al O de Chadin, 3650 m, 22 July 1993, *Sánchez 6586* (F!). Cutervo, Gruta de San Andres, 2200 m, 15 July 1990, *Llatas 2749* (F!, MO!). Hualgayoc, Dist. de Chugur, 06°43'08"S, 078°42'58"W, 3222–3568 m, 12 August 2009, *Castillo 786* (USM!); Hacienda Taulis, 13 km beyond Palmito junction towards La Playa, 2900 m, 02 September 1964, *Hutchison 6463* (MO!, USM!); Chugur, sobre la ruta de Perlamayo, 2950–3000 m, 20 March 1988, *Sánchez 4681* (AAU!, F!). San Miguel, El Prado, Hacienda Taulis, 06°59'02"S, 078°58'21"W, 3398 m, 01 June 2015, *Boza 3029*; *3070*; *3071*; *3072*; *3073*; *3074*; *3075*; *3076*; *3077*; *3078*; *3079*; *3080* (USM!, Z!); Quishuarpampa (Agua Blanca), 2900 m, 04 July 1986, *Mostacero 1201* (F!, MO!); Quishuarpampa (El Tingo–Jalca de las Estacas), 07°21'00"S, 077°50'00"W, 2950 m, 12 May 1977, *Sagástegui 8833* (MO!); Millán (El Tingo–Taulis), 3000 m, 20 June 1980, *Sagástegui 9536* (F!, MO!, USM!); Sobre el desvio a Tongot, entre Quilcate bajo y Catilluc, 3050 m, 13 September 1991, *Sánchez 5762* (MO!). Santa Cruz, Distr. Pulán, parte baja de la Quebrada Cocan, ladera Oeste, 3280 m, 02 November 2001, *Sánchez 11112* (MO!); Dist. Pulan, La Palma, 2800 m, 12 February 2007, *Santa 927* (USM!); ad Chugur versus Hualgayoc, 2700–2900 m, 1901–1929, *Weberbauer 4098* (G, MO!). **La Libertad**: Bolivar, District Uchumarca, Páramo in surroundings of Vira Vira/Lagunas La Quinua, 07°00'12"S, 077°45'07"W, 3670 m, 17 May 2011, *Bussmann 16931* (MO!). Huicungo, Dist. Uchumarca, 06°59'30"S, 077°43'07"W, 3140 m, 02 November 2012, *Paniagua 8642* (MO!). **Lambayeque**: Ferrenafe, Dos Puentes, arriba de Incahuasi, 2900–3000 m, 09 July 1987, *Ferreyra 20908* (USM!); road Incahuasi to Sinchihual and Tungula, 06°12'07"S, 079°17'57"W, 2897 m, 24 November 2014, *Weigend 9660* (USM!).

### ﻿Subsection Pauta

Taxon classificationPlantaeRosalesRosaceae

T.Boza & M.Kessler sect. nov.

5E643363-7777-578C-BF77-5521E9DC215A

urn:lsid:ipni.org:names:77301576-1

#### Diagnosis.

Trees; 4–6 lateral leaflet pairs; lowers leaflet surfaces lanate or sericeous; fruits with flattened or thin spines, densely villous.

#### Type.

*Polylepispauta* Hieron.

#### Note.

The subsectional epithet *Pauta* is a noun in apposition ['Nomen vernaculum: Pauta'; fide: Hieron., Bot. Jahrb. Syst. 21(3): 314. 1895].

### ﻿3. Polylepis longipilosa

Taxon classificationPlantaeRosalesRosaceae

T.Boza, K.Romoleroux & M.Kessler, Phytoxa 454(2): 116. 2020

635A5438-1E26-54E6-94C9-B90FF3E97D40

Figs 18 , 19

#### Type.

**Ecuador. Carchi**: Cantón Montúfar, Loma El Corazón (Bretaña), al sureste de Huaca, al este de la Colonia Huaqueña, Río Minas, 00°35'N, 077°42'W, 3200–3500 m, 9 Apr 1989, *Tipaz 35* (holotype: QCA!; isotypes: AAU!, MO!).

**Figure 18.**
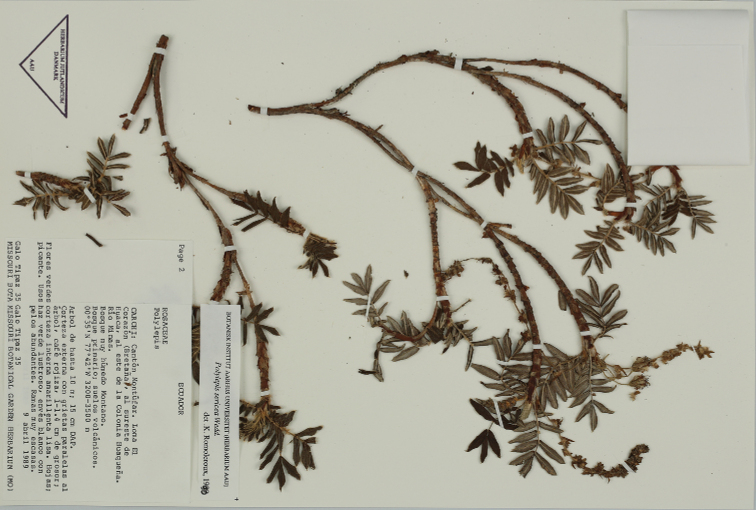
*Polylepislongipilosa* T.Boza, K.Romoleroux & M.Kessler. Isotype: *Tipaz 35* (AAU).

#### Description.

***Trees*** 5–10 m tall. ***Leaves*** strongly congested at the branch tips, imparipinnate with (4–)5–6 pairs of the lateral leaflets, obtrullate in outline, (3.8–)4.3–7.3 × 2.4–4.5 cm; rachises densely sericeous, points of leaflet attachment with a tuft of long, straight whitish to yellowish hairs; stipular sheaths apically truncate, densely sericeous in the upper surface; leaflets narrowly to ovate in outline, second pair from the terminal leaflet the largest, one of this pair 1.4–2.2 × 0.4–0.5 cm; margins entire to slightly crenate with 6–7 teeth, apically slightly emarginate seemingly acute by the prolongation of hairs, basally unequally cordate; upper leaflet surfaces glabrous; lower leaflet surfaces densely lanate with whitish silky hairs 1.1–1.6 mm long. ***Inflorescences*** pendant, (6.8–)11.1–16.6 cm long, bearing 19–29 flowers; floral bracts 6.1–9.4 mm long, narrowly triangular, glabrous on the outer surface; rachises densely sericeous. ***Flowers*** 4.8–5.5 mm diam.; sepals 4, ovate, green, glabrous outside; stamens 8–10, anthers orbicular, with a dense tuft of straight white hairs on the upper half; styles fimbriate, 1.6–2.0 mm long. *Fruits* turbinate, with variable numbers and placement of flattened spines, densely sericeous; 3.9–6.7 × 4.3–7.5 mm including spines. ***Diploid***.

**Figure 19.**
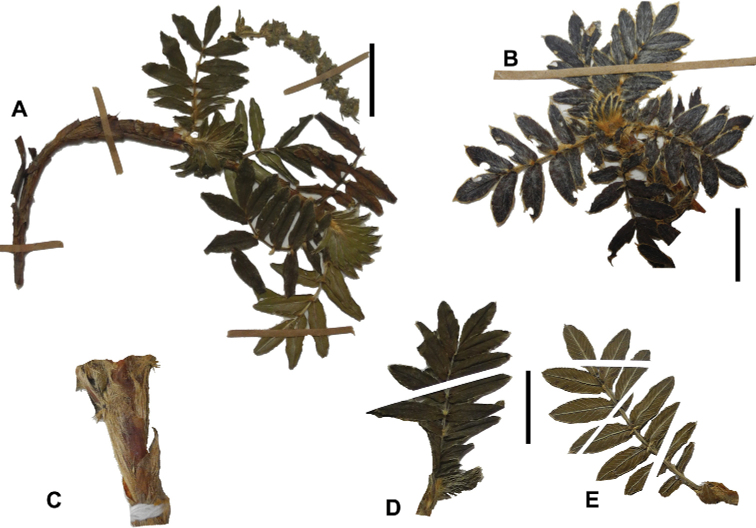
*Polylepislongipilosa* T.Boza, K.Romoleroux & M.Kessler **A** Flowering branch **B** young leaves **C** stipular sheaths **D** upper leaf surface **E** lower leaf surface (**A***Ramsay 911***B***Hutel 1390***C***Laegaard 54965***D, E***Salgado 239a*). Scale bars: 6 cm (**A**); 3 cm (**B, D, E**). Photographs by E. G. Urquiaga F.

#### Distribution, habitat and ecology.

*Polylepislongipilosa* is restricted to north-western Ecuador (Carchi) (Fig. 24). It is highly likely that the species also occurs in adjacent Colombia. It grows in humid páramo habitats at 3200–3900 m elevation where it often co-occurs with *P.ochreata*, with which it hybridizes (Romoleroux 1996). It grows in mixed forest with *Espeletiapycnophylla*, *Weinmannia* sp. and *Oreopanax* sp. (Boada et al. 2008).

#### Conservation status.

The EOO for *Polylepislongipilosa* is estimated as 17,689 km^2^, the AOO is assessed at 60 km^2^ and it is known from eight locations. It occurs in Reserva Ecológica El Angel. However, the Andean Forest and páramos at Carchi have, in recent years, come under increasing threat from timber cutting and forest burning and advancement of the agricultural frontier, which has contributed to the fragmentation and destruction of high Andean ecosystems (Boada et al. 2008). Based on its degraded and fragmented distribution, we assess *P.longipilosa* as Critically Endangered (A2a, B1a+B2a, C1+C2a).

#### Notes.

The populations of *Polylepis* from Carchi on the southern slopes of Volcán Chiles and on the road between Maldonado and Tulcán have previously been identified either as *P.ochreata* (Simpson 1979; Romoleroux 1996, as *P.sericea*) or *P.pauta* (Romoleroux 1996). Here, we treat it as a distinct species which is morphologically closest to *P.ochreata* and *P.pauta*. The most obvious differences between *P.longipilosa* and these species are its longer (1.1–1.6 mm), densely lanate hairs compared to the shorter (0.3–0.5 mm), densely sericeous hairs of *P.ochreata* and relatively short (0.4–0.9 mm), sparsely sericeous hairs of *P.pauta*; the hairs of *P.longipilosa* are concentrated on the leaflet veins (as is also the case in *P.pauta*), whereas in *P.ochreata*, the hairs are generally evenly distributed. *Polylepislongipilosa* also differs from the other two species by the leaflet apex, with *P.longipilosa* having acute or rarely emarginate leaflet apices, whereas *P.ochreata* and *P.pauta* have always emarginate leaflet apices. Additionally, *P.longipilosa* has shorter styles (1.6–2.0 mm), whereas *P.ochreata* and *P.pauta* have styles 2.1–2.6 mm and 2.2–3.0 mm long, respectively. Romoleroux (1996) reported hybrids between *P.longipilosa* and *P.ochreata* (as *P.sericea*).

There are three distinctive collections of the *P.pauta/sericea* complexes that Boza Espinoza et al. (2020a) were unable to assign to species: Loja, Cerro Chinchilla, *J. Jaramillo 7312B*, AAU!, QCA!; Pichincha, Laguna Mojanda, *Brandbyge 42200*, AAU!, MO!, NY, QCA! and *Molau 2294*, AAU!, GB, QCA!. These collections resemble *P.longipilosa*, but have different leaflet shape (obovate vs. ovate) with serrate margins (vs. entire to slightly crenate), longer lower leaflet surface hairs (1.5–1.8 mm vs. 1.1–1.6 mm) and longer styles (2.7–3.0 mm vs. 1.6–2.0 mm). Despite targeted fieldwork at both collecting localities, we have been unable to study this form in the field. The specimens at Mojanda were collected in a well-known hybrid zone and although they are not simply intermediate between the potential parent species (*P.incana*, *P.ochreata*, *P.pauta*) so that they cannot simply be interpreted as hybrids, it is possible that some unique gene combinations in hybrids might lead to morphotypes that fall outside of the morphological range of the parent taxa. In Loja, the situation is somewhat different, because the only species present there is *P.loxensis*, so that a hybrid origin is less likely, although in a wind-pollinated genus, such as *Polylepis*, pollen dispersal might conceivably take place over long distances. Should these collections represent one or even two separate species, then, on present knowledge, they would be very rare or even on the verge of extinction.

#### Specimens examined.

**Ecuador. Carchi**: Tulcán, carretera Túlcan-Tufiño-Maldonado-Chical col. en km 12 de Tufiño, cerca de las lagunas, 00°48'N, 077°55'W, 3900 m, 23 April 1993, *Freire & Andersen 2547* (AAU!); road Tulcán-Maldonado, near Volcán Chiles, 00°48'N, 077°56'W, 3850–4000 m, 16 August 1985, *Laegaard 54965* (AAU!, MO!, QCA!), *54967A*, *54967B*, *54967C* (AAU!), *54967D*, *54967E*, *54967F* (AAU!, QCA!); along the road from Tulcán to Volcán Chiles, 3900 m, 6 October1995, *Sklenár & Kosteckova 1412A* (QCA!); camino Tufiño, sitio Agua Hediondas, en la base del Volcán Chiles, límite con Colombia, 00°48'N, 077°54'W, 3500 m, 8 November 1993, *Palacios 11847* (AAU!, MO!, QCNE); carretera entre Tulcán y Maldonado, faldas del Volcán Chiles, punto más alto del cruce de carretera, 00°45'N, 077°59'W, 3800 m, 19 May 1991, *Palacios & Rubio 7349* (AAU!, MO!); southern slopes of Volcan Chiles, 00°49'N, 077°57'W, 3600 m, *Ramsay 911* (QCA!, QCNE); route de Tufiño a Maldonado, 10 km après Tufiño, zone très humide, 3850 m, 06 July 1988, *Huttel 1390* (QCA!); carretera San Gabriel-Shután alto, 3500 m, 25 March 1989, *Jaramillo-Asanza 10862* (QCA!); comuna La Esperanza, páramo de El Artezón, sector Monte Redondo, 3789 m, 18 September 2007, *Salgado 220B*, *239A* (QCA!).

### ﻿4. Polylepis pauta

Taxon classificationPlantaeRosalesRosaceae

Hieron., Bot. Jahrb. Syst. 21: 313. 1895.

856F71D3-CEE2-51FE-9F7C-598271F0C715

Figs 20 , 21

Polylepis annulatipilosa Bitter, Bot. Jahrb. Syst. 45: 596. 1911. Type. Ecuador. Pichincha: Andes of Quito, *Jameson 16* (lectotype, designated by Simpson 1979, pg. 27: W, isolectotypes: G, GH; photos at F!, MO!, US!).
Polylepis stuebelii Hieron., Bot. Jahrb. Syst. 21: 313. 1896. Type. Ecuador. Napo: E slope of Cerro Quilindaña near Bambasacha, 3700 m, *Stübel 204* (holotype: B destroyed; photos at F!, MO!, NY!, US!).

#### Type.

Ecuador. Pichincha: “Corredor Machai”, 3900 m, Oct 1871, *Stübel 232a* (holotype: B destroyed; photos at F!, GH!, MO!).

**Figure 20.**
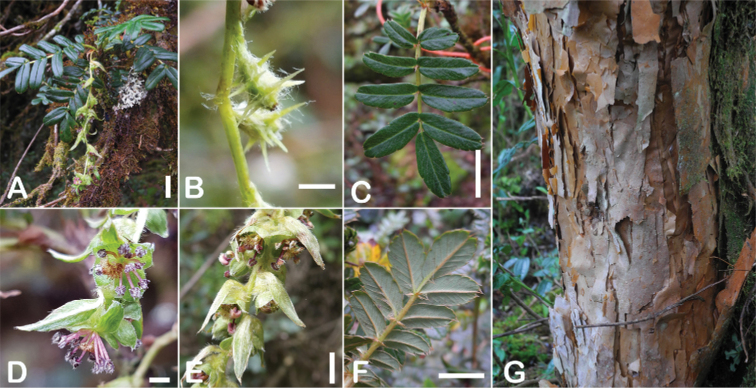
*Polylepispauta* Hieron **A** Inflorescence **B** fruits **C** upper leaflet surface **D** flowers **E** flowers **F** lower leaflet surface **G** bark. Scale bars: 2 cm (**A, C, F**); 2 mm (**B, D, E**). Photographs **A–C, F–G** T. E. Boza E. **D** M. Kessler **E** E.G. Urquiaga F.

#### Description.

***Trees*** 2–12 m tall. ***Leaves*** strongly congested at the branch tips, imparipinnate with 4–5(–6) pairs of the lateral leaflets, obtrullate in outline, 3.2–4.9 × 2.2–3.0 cm; rachises sparsely sericeous, points of leaflet attachment with a tuft of long, straight whitish hairs; stipular sheaths apically acute with spurs, glabrous to sparsely sericeous (adult) or densely sericeous (juvenile) in the upper surface; leaflets elliptic in outline, second pair from the terminal leaflet the largest, one of this pair (1.1–)1.4–1.6 × 0.5–0.6 cm; margin crenate with 4–6 teeth, subcoriaceous, apically emarginate, basally unequally cordate; upper leaflet surfaces glabrous or sparsely sericeous with few hairs on the mid-veins; lower leaflet surfaces sparsely sericeous with whitish hairs 0.4–0.9 mm long. ***Inflorescences*** pendant, (8.1–)12.6–14.3(–19.3) cm long, bearing 9–15(–21) flowers; floral bracts (9.1–)10.0–12.2 mm long, narrowly triangular, densely sericeous on the outer surface; rachises densely villous. ***Flowers*** 6.0–7.4(–9.2) mm diam.; sepals 4, ovate, green, densely sericeous outside; stamens 9–15, anthers orbicular, with a dense tuft of straight white hairs on the upper half; styles fimbriate, 2.2–3.0 mm long. ***Fruits*** turbinate, with variable numbers and placement of flattened spines, densely sericeous; (2.6–)3.4–5.5 × 3.3–6.0(–8.2) mm including spines. ***Tetraploid***, ***aneuploid***; perhaps also ***diploid***.

**Figure 21.**
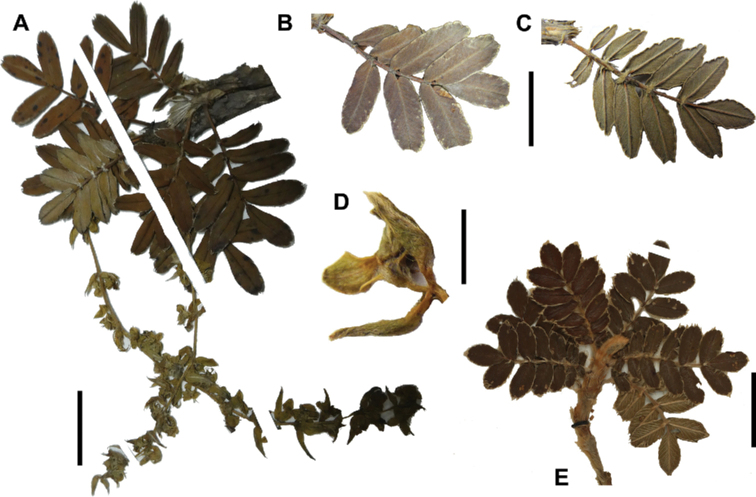
*Polylepispauta* Hieron **A** flowering branch **B** upper leaf surface **C** lower leaf surface **D** fruit **E** young leaves (**A***Zambrano G147***B***Kessler 2750***C***Kessler 2753***D***Laegaard 103115***E***Romoleroux 654*). Scale bars: 4 cm (**A**); 2 cm (**B, C**); 5 mm (**D**); 3 cm (**E**). Photographs by E. G. Urquiaga F.

#### Distribution, habitat and ecology.

*Polylepispauta* occurs in the north-eastern Cordillera Oriental of Ecuador (Fig. 24). It grows in high Andean Forest at 2600–4200 m elevation, often mixed with other tree species such as *Gynoxysacostae*, *Solanumstenophyllum* and *Hesperomelesobtusifolia* (Cierjacks et al. 2007b, 2008). Many populations are restricted to small, isolated forest patches, but in Napo, Ecuador, the species shows no evidence of genetic isolation by distance (Aragundi et al. 2011). Larger forest patches with broader elevational ranges have higher genetic diversity than forest patches on steeper slopes and at higher elevations, possibly due to increasing vegetative reproduction with elevation (Cierjacks et al. 2007a; Aragundi et al. 2011). In the same general region, Cierjacks et al. (2008) found a decrease of the number of inflorescences with elevation. Overall, reproductive success is low and decreases with elevation, being higher in the forest interior than outside and even higher when the litter layer is removed (Cierjacks et al. 2007b). Forests of *P.pauta* support a diverse bryophyte flora with numerous endemic species (Gradstein and León-Yañez 2018).

#### Conservation status.

The EOO for *Polylepispauta* is estimated as 1,590 km^2^, the AOO is assessed at 132 km^2^ and it occurs at 14 locations. Large populations occur within Cayambe-Coca National Park and Antisana Ecological Reserve, but there is genetic evidence for a loss of genetic diversity due to forest fragmentation (Aragundi et al. 2011). Moderate cattle grazing increases seedling abundance, presumably due to the removal of the litter layer (Cierjacks et al. 2008). We assess *P.pauta* as Vulnerable (A1, B1a+B2a, C1).

#### Notes.

Simpson (1979) noticed that it is possible to morphologically differentiate various geographical populations within *P.pauta* as defined by her to include the populations here separated under *P.longipilosa* and *P.serrata*. Based on our fieldwork in Ecuador to clarify the taxonomic position of the ecologically well-studied populations to the east of Quito, we recognize two species based on their morphology, ecology and geographical distribution: the populations of the north-eastern Cordillera Oriental as *P.pauta* and the northern population as *P.longipilosa* (Boza Espinoza et al. 2020a). *Polylepispauta* differs from *P.longipilosa* by having shorter leaflets (1.1–1.6 cm versus 1.4–2.2 cm in *P.longipilosa*) with crenate margins and emarginate apex (versus entire to slightly crenate margins and acute to rarely emarginate apex), shorter hairs (0.4–0.9 mm versus 1.1–1.6 mm) and fewer flowers per inflorescence (9–21 versus 19–29). Occasionally, specimens of *P.pauta* resemble those of *P.ochreata* in having the same number of lateral leaflets, but leaflet margins are crenate in *P.pauta* and entire to slightly serrate in *P.ochreata*.

An outstanding feature of *P.pauta* are the differences in leaflet number, shape and indument between young and adult plants. On young plants, leaves look very similar to those of *P.longipilosa*, whereas as the plants mature, the leaves become almost glabrous and have 4–5 lateral leaflet pairs.

#### Specimens examined.

**Ecuador. Cotopaxi**: 2 km S of Paso de la Virgen on road Quito-Baeza, 00°20'S, 078°13'W, 3850–4000 m, 16 May 1984, *Lægaard 52133* (AAU!, QCA!). **Imbabura**: González Suárez, Lagunas de Mojanda, Laguna Negra, 00°08'N, 078°15'W, 3700 m, 22 September 1990, *Øllgaard 98194* (AAU!); vía hacia la laguna de Mojanda, 00°08'N, 078°15'W, 3500–3700 m, 02 November 1987, *Romoleroux 475* (AAU!, QCA!). Otavalo, road from Otavalo to lagunas Mojanda, ca. 3 km before the lakes, 00°10'N, 078°17'W, 3500–3700 m, 23 October 1983, *Balslev 4450* (AAU!, QCA!). Quiroga, Cotacachi, Reserva Ecológica Cotacachi-Cayapas, 00°18'N, 078°22'W, 3300–3350 m, 02 March 1992, *Peñafiel 1091* (MO!). Tocachi, Laguna Grande de Mojanda, 15 km S of Otavalo, 00°08'N, 078°16'W, 3750 m, 14 May 1985, *Eriksen 59359* (AAU!); *59374* (AAU!). Mojanda, Tomauco, 3309 m, 05 June 2008, *Salgado 428* (QCA!); Mojanda, Tomauco, 3274 m, 05 June 2008, *Salgado 458* (QCA!); Páramo de Mojanda, on the SW slope of the peak Nudo de Mojanda, 4130 m, 06 November 2007, *Sklenar 10746* (QCA!). **Napo**: Oyacachi, 0°12'S, 78°8'W, 3680 m, 08 April 2012, *Homeier 4948* (QCA!); N of Volcán Los Puntos, 00°12'S, 078°10'W, 4200 m, 27 July 1985, *Lægaard 54756A* (AAU!); N of Volcán Los Puntos, 00°12'S, 078°10'W, 3850–3900 m, 28 July 1985, *Lægaard 54756B* (AAU!); N of Volcán Los Puntos, 00°12'S, 078°10'W, 3850–3900 m, 28 July 1985, *Lægaard 54756C* (AAU!); *54756D* (AAU!); *54756E* (AAU!); *54756F* (AAU!); *54761* (AAU!, MO!); Reserva Ecológica Oyacachi, 00°13'49"S, 078°08'44"W, 3915 m, 16 December 2008, *Romoleroux 5346* (MO!, QCA!). Papallacta, Oyacachi, 0°18'6"S, 78°8'28"W, 3970 m, 10 June 2009, *Homeier 4191* (QCA!); Páramo de Papallacta, 00°20'S, 078°10'W, 12 January 2015, *Kessler s.n* (Z!); Pifo-Papallacta, 3–5 km E of Paso de La Virgen, Páramo-swamp, 00°21'S, 078°11'W, 3700–3900 m, 09 June 1992, *Lægaard 103115* (AAU!, GOET!, QCA!); 3 km E of Paso de la Virgen on road Pifo-Papallacta, 00°20'S, 078°11'W, 3950–4050 m, 02 June 1985, *Lægaard 54442* (AAU!, QCA!); *54443* (AAU!); *54444* (AAU!, MO!); *54446* (AAU!); *54447* (AAU!, MO!, QCA!); *54448* (AAU!, MO!, QCA!); *54449* (AAU!, QCA!); *54450* (AAU!, MO!); *54451* (AAU!, MO!); *54452* (AAU!, MO!); along road Pifo-Papallacta, E of Paso de la Virgen, 00°21'S, 078°11'W, 3750–3850 m, 21 June 1985, *Lægaard 54558B* (AAU!); *54558C* (AAU!); *54558D* (AAU!); *54559* (AAU!, MO!, QCA!); *54560* (AAU!, MO!); *54561* (AAU!); road Quito–Baeza, 7–8 km NW of Laguna de Papallacta (Páramo de Guamaní), 00°19'S, 078°08'W, 3800 m, 20 July 1976, *Øllgaard 8156* (AAU!, MO!, NY); Reserva Ecológica Oyacachi, 00°17'46"S, 078°08'49"W, 3927 m, 20 September 2008, *Romoleroux 5194* (MO!, QCA!); carretera Quito–Baeza, páramo above Papallacta, 00°21'S, 078°10'W, 3400–3700 m, 28 May 1987, *van der Werff 9638* (AAU!, GB, MO!). Pintag, Paso de Guamaní, quebrada, about +-4 km E Paso de Guamaní, on road to Papallacta, 00°20'S, 078°20'W, 3900 m, 26 March 1967, *Sparre 15029* (AAU!, S). Quijos, Parroquia Papallacta, 00°21'S, 078°11'W, 3700 m, 28 May 1990, *Cerón 10054* (MO!); Reserva Ecológica Antisana, carretera Pifo-Baeza, Páramo de la Virgen, 00°20'S, 078°12'W, 3960 m, 23 November 1998, *Freire 2852* (AAU!, ILLS, MO!, QCNE); Reserva Ecológica Antisana, carretera Pifo–Baeza, Páramo de la Virgen, 00°23'S, 078°12'W, 3730 m, 24 November 1998, *Freire 2870* (ILLS, MO!, QCNE); Reserva Ecológica Antisana, Páramo de Guamaní, carretera Pifo-Papallacta, La Virgen, 00°20'S, 078°12'W, 4140 m, 24 July 1998, *Vargas 1946* (AAU!, ILLS, MO!, QCNE); carretera Quito-Tena via Baeza km 52, 3820 m, 03 August 1984, *Dodson 14832* (MO!); 8 kms de la población de Oyacachi, siguiendo el sendero hacia Cochapamba, 3500 m, 12 March 1991, *Gavilánez 462* (QCA!); carretera Oyacachi-Papallacta, colecciones a 11 km de la Laguna de Loreto, 3800 m, 27 April 1998, *Guerrón 343* (QCA!); Papallacta, 3400–3600 m, 16 August 1990, *Jaramillo 11832* (AAU!, MO!); Papallacta, 3400 m, 17 August 1990, *Jaramillo 11842* (MO!); Páramo de Guamaní, road Quito Papallacta, 4000 m, 04 March 1979, *Kieft 228* (QCA!); 3 km E of Paso de la Virgen on road Pifo-Papallacta, 3951–4050 m, 06 February 1985, *Lægaard 54452* (QCA!); along road Pifo-Papallacta, E of Paso de la Virgen, 3750 m, *Lægaard 54558* (QCA!); along road Pifo-Papallacta, E of Paso de la Virgen, 3750–3850 m, 21 June 1985, *Lægaard 54560* (QCA!); N of Volcán Los Puntos, 3850 m, *Lægaard 54756* (QCA!); N of Volcán Los Puntos, 3850–3900 m, 28 July 1985, *Lægaard 54757* (QCA!); N of Volcán Los Puntos, 3851–3900 m, 28 July 1985, *Lægaard 54758* (QCA!); *54759* (QCA!); Oyacachi, Yarupaccha, 3620–3680 m, 16 January 1996, *Navarrete 1449* (QCA!); Reserva Ecológica Oyacachi, 3940 m, 28 January 2007, *Romoleroux 4282* (QCA!); *4297* (QCA!); Reserva Ecológica Oyacachi, 3895 m, 23 February 2007, *Romoleroux 4340* (QCA!); Reserva Ecológica Oyachachi, 3465 m, 08 March 2008, *Romoleroux 4751* (QCA!); Páramo de Guamaní, alrededores de la laguna de Papallacta, 3900–4000 m, 06 December 1987, *Romoleroux 491* (AAU!, NY, QCA!); Reserva Ecológica Oyachachi, 3929 m, 13 September 2008, *Romoleroux 5167* (QCA!); 3681 m, 06 February 1985, *Romoleroux 5168* (QCA!); 3917 m, 14 April 2009, *Romoleroux 5475* (QCA!); 3880 m, 16 May 2009, *Romoleroux 5489* (QCA!); Reserva Ecológica Oyacachi, 3560 m, 2 February 2007, *Romoleroux A4321* (QCA!); Páramo de la Virgen, 3904 m, 29 September 2004, *Salgado 1* (QCA!); about 3 km W of Oyacachi, 3550 m, 27 March 1996, *Ståhl 2278* (QCA!); crescit prope Bambasacha in declivibus orientalibus mentis Quilindaña sitis, 3700 m, s.d., *Stübel 204* (B, F!, MO!, NY, US!). **Pichincha**: Cayambe, carretera Cayambe-Hda. Piamonte-Patapamba, 00°02'S, 078°04'W, 3700 m, 04 December 1993, *Freire 2606* (AAU!, QCA!). Papallacta, Along road Pifo-Papallacta, E of Paso de la Virgen, 00°21'S, 078°11'W, 4200–4300 m, 20 June 1985, *Lægaard 54558A* (AAU!); Pichincha-Napo, base del Volcán Antisana, entrada por Pintag hacia laguna Micacocha, campamento de EMAP, 00°27'S, 078°10'W, 4000–4100 m, 09 October 1990, *Romoleroux 1117* (AAU!, QCA!); Pichincha-Napo, base del Volcán Antisana, entrada por Pintag hacia laguna Micacocha campamento EMAP, 00°27'S, 078°10'W, 4000–4100 m, 09 October 1990, *Romoleroux 1118* (AAU!, QCA!); Páramo de la Virgen, camino antiguo, 0°20'S, 78°12'W, 3938 m, 20 September 2004, *Salgado 3A* (QCA!). Pifo, Mount Guamaní, 0°20'S, 78°33'W, 3600–3800 m, 15 September 1939, *Asplund 8767* (QCA!); 2 km west of La Virgin on the road from Pifo to Papallacta, 00°17'S, 078°12'W, 3950–4050 m, 20 May 1984, *Brandbyge 42638* (AAU!, MO!); Pifo-Papallacta (new road) app. 1 km W of Paso de la Virgen, 00°19'S, 078°13'W, 3700 m, 16 April 1992, *Lægaard 102327* (AAU!, GOET!); 2 km S of Paso de la Virgen on road Quito-Baeza, 00°20'S, 078°13'W, 4000–4200 m, 19–20 May 1984, *Lægaard 52134* (AAU!); *52135* (AAU!); *52138* (AAU!, MO!); *52162* (AAU!); *52176* (AAU!, QCA!); road Pifo–Papallacta, near Paso de la Virgen, 00°19'S, 078°13'W, 4000–4100 m, 13 March 1985, *Lægaard 53849* (AAU!, MO!, QCA!); road Pifo-Papallacta, 3 km W of Paso de la Virgen, 00°18'S, 078°14'W, 3700–3900 m, 07 August 1985, *Lægaard 54901A*; *54901B*; *54901C*; *54901D*; *54902AA*; *54902K*; *54902M*; *54902P*; *54902S*; *54902U*; *54902W*; *54902Y* (AAU!); at Paso de la Virgen, 00°18'S, 078°12'W, 4000–4050 m, 28 November 1985, *Lægaard 55729* (AAU!, GOET!, MO!); carretera Quito–Papallacta, 1 km al este de la cumbre (La Virgen), 00°20'S, 078°15'W, 3800 m, 06 October 1986, *Neill 7378A* (AAU!, MO!, QCA!); 2 km al E de la cumbre de la carretera Pifo-Papallacta (La Virgen), 00°20'S, 078°15'W, 3900 m, 28 November 1987, *Neill 8018* (AAU!, GB, MO!, QCA!, QCNE); Vía Baeza, 1 km antes del cruce de la Virgen, 00°18'S, 078°12'W, 3950 m, 01 March 1989, *Palacios 3994* (AAU!, MO!); carretera Quito-Papallacta km 40–53, 00°16'S, 078°15'W, 3300–3800 m, 27 December 1992, *Romoleroux 1507* (AAU!, QCA!); 00°21'S, 078°13'W, *Romoleroux 353* (QCA!); Páramo de Guamaní, on the left side of the road Quito-Papallacta, 0°19'S, 78°12'W, 4000 m, 28 June 1997, *Sklenár 2019* (QCA!). Quito, Parroquia Pifo, carretera Quito–Baeza, Páramo de la Virgen, 00°14'S, 078°20'W, 3500–3900 m, 25 April 1992, *Cerón 18792* (MO!); *Jameson 16* (MO!); road from Quito via Pifo to Papallacta, 00°34'S, 078°19'W, 3950 m, 04 July 2014, *Kessler 14602*; *14603*; *14604*; *14605* (Z!); Pass on Quito-Papallacta road, 3800–3900 m, 06 April 1991, *Kessler 2750* (GOET!); *2755* (GOET!); Páramo de Guamaní, carretera Pifo-Papallacta, Km 27, 00°19'S, 078°12'W, 3960 m, 13 June 1990, *León 1149* (QCA!); Baeza-Quito km 53, 00°20'S, 078°12'W, 4200 m, 08 July 2002, *Schmidt-Lebuhn 378* (GOET!, QCA!). Tabacundo, at highest pass on road Mojanda-Tabacundo, 00°07'N, 078°15'W, 4030 m, 08 April 2001, *Lægaard 21538A*; *21538B* (AAU!). Tocachi, Páramo de Mojanda, at Laguna Negra and S-side of Laguna Grande, 00°08'N, 078°16'W, 3800 m, 14 May 1985, *Lægaard 54316B* (AAU!, MO!, QCA!); *54330* (AAU!, MO!); *54333* (AAU!, MO!, QCA!); *54336* (AAU!); *54346* (AAU!, MO!, QCA!); Lagunas Mojanda, 00°07'N, 078°16'W, 3800 m, 30–31 Jul 1992, *Palacios 10210* (AAU!, MO!); *10239* (AAU!, MO!); Lagunas de Mojanda, ca. Laguna Grande, 00°08'N, 078°16'W, 3700–3800 m, 01 June 1988, *Romoleroux 654* (AAU!, QCA!); 3400–3500 m, *Acosta-Solís 8379* (F!); 3700–4000 m, s.d., *Asplund 18244* (S); alrededores de la Laguna Grande de Mojanda Cajas, 3960 m, 27 February 1999, *Jaramillo 20986* (QCA!); the pass on Quito-Papallacta road, 3800–3900 m, 06 April 1991, *Kessler 2749* (GOET!, MO!); *2750* (GOET!, MO!); *2753* (GOET!, MO!); *2754* (GOET!, LPB, MO!); Páramo de la Virgen, 3100 m, 01 November 2006, *Muñoz 4* (QCA!); Laguna grande de Mojanda-Cajas, 3800 m, 19 September 2011, *Pérez 5117* (QCA!); 3960 m, 01 August 1975, *Little 22* (MO!).

### ﻿5. Polylepis serrata

Taxon classificationPlantaeRosalesRosaceae

Pilg., Bot. Jahrb. Syst. 37: 536. 1906.

4549CD91-2930-5ECC-AE1D-6A6E4B73FC75

Figs 22 , 23

Polylepis serrata var. parcipila Bitter, Bot. Jahrb. Syst. 45: 593. 1911. Type. Peru. Cusco: La Convencion, Yanamanche, between Cusco and Santa Ana, 3500–3800 m, *Weberbauer 4954* (holotype: B destroyed; isotype: Vratisl).
Polylepis serrata var. psilanthera Bitter, Bot. Jahrb. Syst. 45: 593. 1911. Type. Based on Polylepisserrata Pilg.

#### Type.

Peru. Huanuco: Huamalics, southeast of Monzon, 3400–3500 m, 1903, *Weberbauer 3354* (holotype: B destroyed; photos at MO!, US).

**Figure 22.**
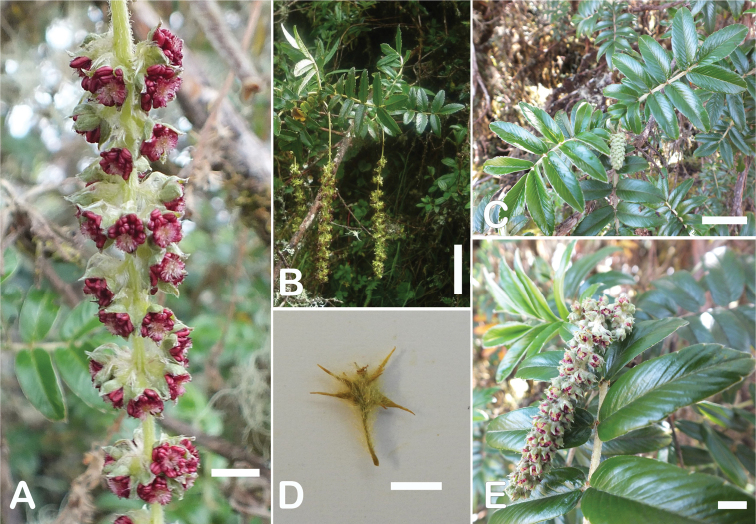
*Polylepisserrata* Pilg **A** inflorescence **B** flowering branch **C** leaves **D** fruit **E** inflorescence. Scale bars: 5 mm (**A**); 3 cm (**B**); 2 cm (**C**); 1 cm (**E**); 3 mm (**D**). Photographs by E.G. Urquiaga F.

#### Description.

***Trees*** 3–27 m tall. ***Leaves*** slightly congested at the branch tips, imparipinnate with 4–7 pairs of lateral leaflets, obtrullate in outline, (5.4–)6.7–8.7(–11.1) × (3.2–)3.9–5.7(–6.4) cm; rachises densely tomentose; points of leaflet attachment with a ring of short tomentose hairs around; stipular sheaths apically acute with spurs, densely lanate on the outer surfaces; leaflets elliptic in outline, second pair from the terminal leaflet the largest, one of this pair (1.8–)2.4–3.5 × 0.8–1.0(–1.2) cm; margin serrate with 4–5 teeth, apically acute, basally unequally cordate; upper leaflet surfaces glabrous or sparsely lanate mainly in the mid-vein depression; lower leaflet surfaces densely lanate with whitish hairs 0.7–1.2 mm long. ***Inflorescences*** pendant, (7.6–)9.5–13.3(–17.3) cm long, bearing 16–35 flowers; floral bracts 3.4–4.5 mm long, narrowly triangular, densely villous on the outer surface; rachises villous. ***Flowers*** 5.2–5.9 mm diam.; sepals 4, ovate, green, densely sericeous outside; stamens (4–)6–14, anthers orbicular, with a dense tuft of straight white hairs on the upper half; styles fimbriate, 1.2–2.3 mm long. ***Fruits*** turbinate, with variable numbers and placement of thin spines, densely villous; (3.8–)6.1–6.7 × 5.6–8.8 mm including spines. ***Diploid***.

**Figure 23.**
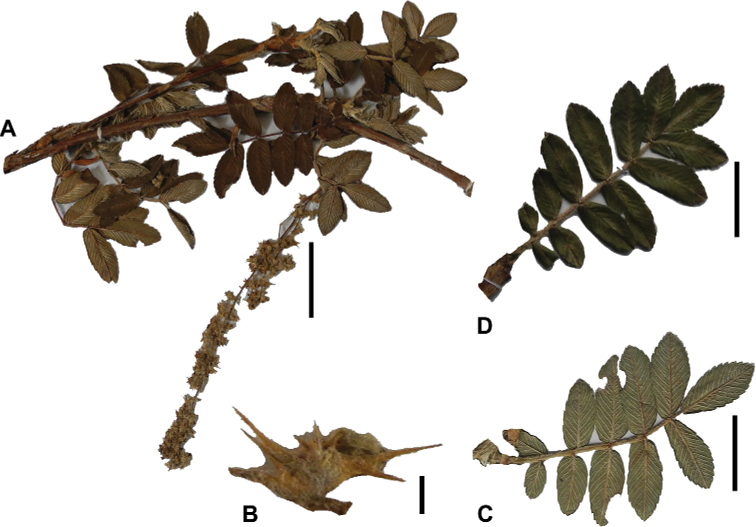
*Polylepisserrata* Pilg **A** flowering branch **B** fruit **C** lower leaf surface **D** upper leaf surface (**A, B***Arce s.n***C, D***Toivonen 90*). Scale bars: 3 cm (**A, C, D**); 4 mm. Photographs by T. E. Boza E.

#### Distribution, habitat and ecology.

**Figure 24.**
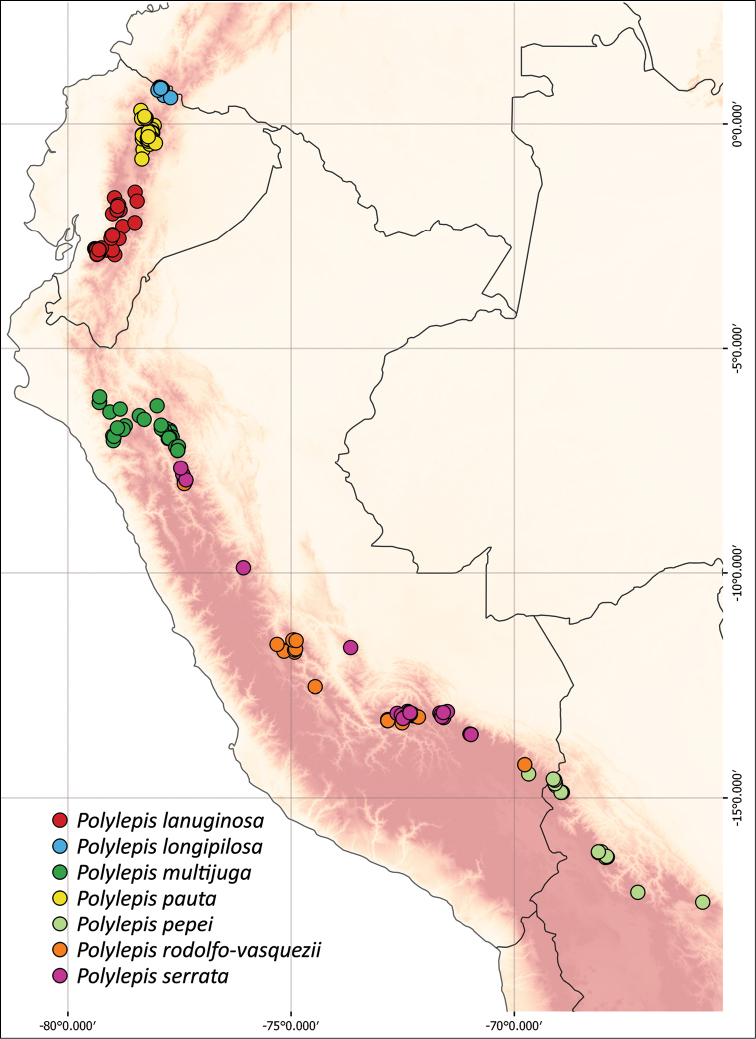
Geographical distribution of the species of the subsections *Lanuginosae*, *Pauta* and *Pepea*.

*Polylepisserrata* is distributed from San Martín to Cusco, Peru (Fig. 24), where it occurs at 2000–3950 m elevation. It grows in relatively wet habitats usually mixed with or at the upper edge of the montane cloud forest, commonly with species of the genera *Oreopanax* and *Weinmannia* (Young 1993). It often also co-occurs with *P.canoi* and towards its upper distribution grades into forests of *P.rodolfovasquezii* (Boyle 2001; Kessler et al. 2014). This is one of the tallest *Polylepis* species, with heights of up to 27 m and diameters of up to 80 cm recorded (Toivonen et al. 2011). In a study in Cuzco, Peru, vegetative reproduction was found to be absent at 3000 m, but increased to around 80% at 3500–3800 m (Toivonen et al. 2011). Based on pollen records, *Polylepis* of presumably this species was common at Laguna de Chochos in San Martín, Peru, at the beginning of the Holocene some 10,000–6,000 bp and later declined (Bush et al. 2005). The ecophysiology of this species has been studied by Toivonen et al. (2014; as *P.pauta*).

#### Conservation status.

The EOO for *Polylepisserrata* is estimated as 68,454 km^2^, the AOO is assessed at 100 km^2^ and it occurs at 18 locations. In Peru, was categorized as Near Threatened (SERFOR 2006, as *P.pauta*). It is restricted to small areas of eastern Peru where it is protected within Río Abiseo and Manu National Parks. However, the habitat of *P.serrata* is threatened by fires and forest destruction. We assess this species as Vulnerable (B1a+B2a, C1).

#### Notes.

This species is quite similar to *P.pauta* and, in fact, it was treated as a junior synonym by Simpson (1979). Nevertheless, Simpson already pointed out the morphological diagnosability of geographical populations of *P.pauta* as defined by her. We consider that the recognition of *P.serrata* is justified, based on morphological, ecological and geographical grounds, and suggest that this taxon should be re-instated at species level. *Polylepisserrata* differs from *P.pauta* by having longer leaflets (1.8–3.5 cm versus 1.7–2.2 cm long) with different type and relatively shorter hairs (0.7–1.2 mm, lanate versus 0.9–1.9 mm, sericeous). *Polylepisserrata* further has shorter styles (1.2–2.3 mm; *P.pauta*: 2.3–2.5 mm).

*Polylepisserrata* also is morphologically similar to *P.ochreata*, with which it shares the number of leaflets (4–7 pairs). The most obvious differences between *P.serrata* and this species are the leaflet width, margin, apex and hair type and length, with *P.serrata* having elliptic leaflets 0.8–1.2 cm long, with acute apex and longer lanate hairs (0.7–1.2 mm) on the lower surface, whereas *P.ochreata* has narrowly elliptic leaflets 0.5–0.7 cm long, emarginate apex and short sericeous hairs (0.3–0.5 mm) on the lower surface.

#### Specimens examined.

**Peru. Cusco**: Calca, Lares Cuncani, 07 June 1991, *Tupayachi 1505* (CUZ!). La Convención, Prov. Machupicchu, Chakimayu, 3235 m, 01 September 2002, *Arce s.n* (CUZ!, USM!); Batiyayoc 13°08'01"S, 072°19'45"W, 3705 m, 01 October 2002, *Arce s.n* (CUZ!); Dist. Santa Teresa, Uchuyillaspay, 13°07'23"S, 072°37'30"W, 3883 m, 22 September 2005, *Huamantupa 7018* (CUZ!, MO!); Dist. Echarate, Huayopata, San Luis, 13°04'43"S, 072°23'25"W, 2800 m, 30 March 2006, *Huamantupa 7526* (CUZ!, MO!, USM!); Potrero, Bosque de Ukumuriyoc, 3600 m, 01 October 2002, *Palomino 1737* (QCA!); Dist. Huayopata, Panticalle, Abra Málaga 13°08'02"S, 072°19'41"W, 3690 m, 30 May 2006, *Toivonen 88* (CUZ!); *89* (CUZ!); *90* (CUZ!); *91* (CUZ!); Cerca Canchayoc, 3600 m, 29 June 1967, *Vargas 19872* (CUZ!); Canchayoc, 3650–4000 m, 10 January 1968, *Vargas 20086* (CUZ!); Canchayoc, 3700 m, 23 April 1980, *Vargas 23311* (CUZ!); Yanamanche Quellomayo, 3600–4000 m, 25 July 1944, *Vargas 4457* (CUZ!). Paucartambo, Challabamba, Pumataki, 13°09'16"S, 071°38'33"W, 3671 m, 10 December 2014, *Boza 3024* (USM!, Z!); Challabamba, between Acjanaco and Tres Cruces, 13°10'07"S, 071°37'58"W, 3450 m, 10 December 2014, *Boza 3025* (USM!, Z!); Trocha Ericsson, Acjanaco, Parque Nacional Manu, 3250–3350 m, 01 September 1990, *Cano 4041* (USM!); Qollatambo, Parque Nacional Manu, 3700–3800 m, 10 September 1990, *Cano 4319* (USM!); Tres Cruces, Parque Nacional Manu, 3600–3700 m, 06 March 1991, *Cano 4588* (USM!); Cerro Chapuyoc, Challabamba, ParqueNacional Manu, 3350–3450 m, 15 March 1991, *Cano 4689* (USM!); Valle del Pilcopata, near Accanaco Pass, turnoff to Tres Cruces,13°13'S, 071°35'W, 3500 m, 15 December 1983, *Foster 7548* (MO!, USM!); Acjanaco, Parque Nacional Manu, Trocha Ericsson, 3000–3200 m, 22 July 1991, *Huapaya 221* (USM!); Region of Acanacu and the Cordillera or Tres Cruces, 3290–3500 m, 07 December 1978, *Luteyn 6386* (AAU!, MO!, USM!); Pillahuata, alrededores, Tres cruces, 130 km de Cusco en el camino hacia Pilcopta, 13°05'S, 071°30'W, 2000 m, 04 April 1987, *Núñez 7749* (CUZ!, MO!, USM!); Km 130 hacia Kosñipata; incluye Acjanacu, Pillahuata, parte alta del Parque Nacional del Manu y ceja de selva hacia Kosñipata, 13°05'S, 071°30'W, 2600 m, 30 October 1987, *Núñez 8482* (CUZ!, MO!); Tres Cruces, Parque Nacional Manu, 3500–3700 m, 01 April 1989, *Tovar 10081* (USM!); Abra de Acjanaco-Tres Cruces de Oro, carretera Acjanaco-Pillahuata, 13°07'S, 071°40'W, 3700 m, 13 November 1986, *Tupayachi 44*; *Tupayachi 45* (CUZ!, MO!); Entre Paucartambo y Acjanacu, Abra de Acjanacu, 3500 m, 25 January 1960, *Vargas 13130* (CUZ!); Hda. Pillco, Valle de Paucartambi, 2800 m, 12 April 1967, *Vargas 19242* (CUZ!); Abra de Acjanacu, 3500 m, 20 June 1986, *Vargas 24009* (CUZ!); Quebrada de Acjanacu, 3500 m, 11 December 1942, *Vargas 3004* (CUZ!, MO!); Chacapampa, 1800–2000 m, 01 December 1950, *Vargas 9909* (CUZ!, MO!). Quispicanchis, Marcapata; 176 km from Cusco on road to Maldonado, Marcapata remmant forest to Cocha, 13°25'S, 070°54'W, 3150 m, 08 March 1991, *Núñez 13151* (CUZ!, MO!); entre Abra Walla Walla y Marcapata a 210 km de Cusco, 13°25'S, 070°54'W, 2800–4600 m, 21–25 April 1988, *Núñez 9032* (CUZ!, IBE, MO!); Huaillai-Marcapata, junto al río Araza, 2900 m, 11 December 1943, *Vargas 3765* (CUZ!); Marcapata, 15–16 February 1929, *Weberbauer 7803* (A!, MO!). Urubamba, Pakaymayu, 13°14'11"S, 072°29'38"W, 3861 m, 01 June 2002, *Arce s.n* (USM!); entre San Luis y Abra Malaga, 13°06'S, 072°22'W, 3500 m, 16 October 2002, *Lehnert 444* (GOET!); Machu Picchu’, in Urcoscancha, a pampa above the village of Palcay, 13°09'30"S, 072°31'53"W, 3645 m, 05 July 1982, *Peyton 792* (MO!); Lado Oriental de Cumbre Málaga, 01 October 1984, *Rivas s.n* (USM!); Machupicchu, campamento (km 90), 13°11'17"S, 072°26'10"W, 3070 m, 02 August 2006, *Toivonen 1* (CUZ!); Dist. Machupicchu, campamento (km 90), 13°11'17"S, 072°26'10"W, 3070 m, 02 August 2006, *Toivonen 2* (CUZ!); Dist. Machupicchu, Pakaymayu, 13°14'9"S, 072°29'36"W, 3760 m, 24 August 2006, *Toivonen 24*, *25*; *26*; *27*; *30* (CUZ!); Dist. Machupicchu. Pakaymayu, 13°14'9"S, 072°29'36"W, 3760 m, 13 September 2006, *Toivonen 3760* (CUZ!). **Huánuco**: Pachitea, Dist. de Umari, Comunidad Campesina de San Marcos, 3400 m, 04 March 2010, *Beltrán 6740* (USM!); *6760* (USM!); 01 July 1903, *Weberbauer 3354* (B, MO!). **Junín**: Satipo, Cordillera Vilcabamba, Río Ene slope, near summit of divide, 11°39'36"S, 073°40'02"W, 3320 m, 14 June 1997, *Boyle 4398* (USM!). **San Martín**: Mariscal Caceres, Primer derrumbe y laguna del pato y asociadas del P. N. del Río Abiseo, 3250–3450 m, 24 June 1996, *Cano 7265* (USM!); Dist. de Huicungo, Parque Nacional Río Abiseo, Callejón Rojas, 3600–3700 m, 06 July 2011, *Castillo 1026* (USM!); *959* (USM!); Dist. de Huicungo, zona de Alpamachay, 3200–3300 m, 14 June 2001, *León 5224* (USM!); Río Abiseo National Park; forest on edge of Laguna de Chochos, NW corner of park, 07°00'S, 077°00'W, 3300 m, 19 May 1986, *Young 3175* (F!, MO!, USM!); Río Abiseo National Park, jucture of Quebrada Misquichilca and Quebrada Quimar, 4 km SE of Condormarca, 07°00'S, 077°00'W, 3500 m, 05 June 1986, *Young 3552* (F!, MO!, USM!); forest on the edge of Laguna de Chochos, Chochos, NW corner of Río Abiseo National Park, 07°S, 077°W, 3300 m, 17 July 1987, *Young 4863* (MO!, USM!).

### ﻿Subsection Sericeae

Taxon classificationPlantaeRosalesRosaceae

T.Boza & M.Kessler sect. nov.

8530C80D-A7CD-50C4-A208-AB2B423DBA65

urn:lsid:ipni.org:names:77301627-1

#### Diagnosis.

Trees; 2–7 lateral leaflet pairs; lower leaflets sericeous or villous; fruits with flattened spines, densely sericeous or villous.

#### Type.

*Polylepissericea* Wedd.

#### Note.

The subsectional epithet *Sericeae* is a plural adjective agreeing in gender with *Polylepis*.

### ﻿6. Polylepis albicans

Taxon classificationPlantaeRosalesRosaceae

Pilger, Bot. Jahrb. Syst. 37: 535. 1906.

D441C010-9188-52BA-874B-FD625D219A6A

Figs 25 , 26

#### Type.

Peru. Ancash: Cordillera Blanca above Caraz, Jun 1903, *Weberbauer 3229* (holotype: B destroyed; photos at F!, GH!, NY!).

**Figure 25.**
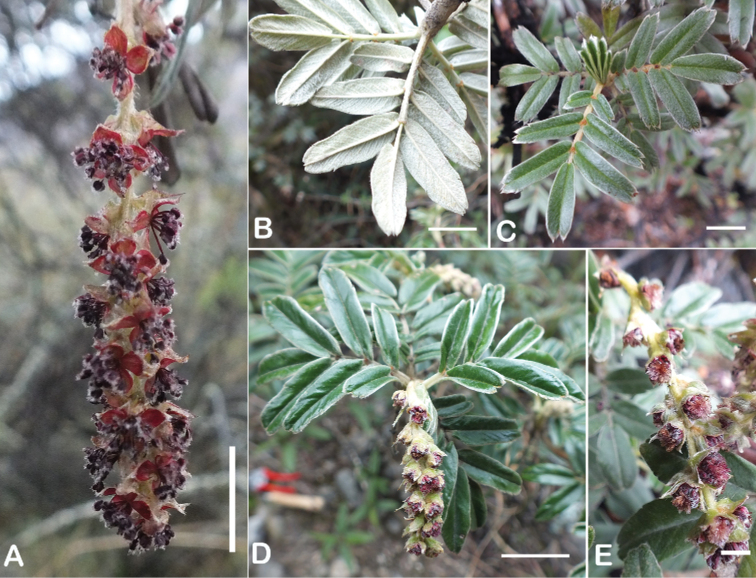
*Polylepisalbicans* Pilger **A** inflorescence **B** lower leaflet surface **C** upper leaflet surface **D** branch apex with young inflorescence **E** young inflorescence (**A***Boza & Urquiaga 3014***B***Boza & Urquiaga 3013***C–E***Boza & Urquiaga 3015*). Scale bars: 1 cm (**A, B, C**); 2 cm (**D**); 0.4 cm (**E**). Photographs by E. G. Urquiaga F.

#### Description.

***Trees*** 3–7(12) m tall. ***Leaves*** strongly congested at the branch tips, imparipinnate with 3–4 pairs of lateral leaflets, obtrullate in outline, 3.5–4.9 × (2.5–)2.8–3.4 cm; rachises densely sericeous, points of leaflet attachment with a tuft of long, straight hairs, with ferruginous resin at leaflet insertion; stipular sheaths apically acute with spurs, densely sericeous on the outer surfaces; leaflets elliptic in outline, second pair from the terminal leaflet the largest, one of this pair 1.4–2.0 × 0.4–0.7 cm; margin slightly crenate at the apex with 4–5 teeth, strongly revolute, coriaceous, apically emarginate, basally unequally cordate; upper leaflet surfaces glabrous or sparsely sericeous; lower leaflet surfaces densely sericeous with short silky hairs 0.3–0.5 mm long. ***Inflorescences*** pendant, 3.9–6.6(7.5) cm long, bearing 18–21 flowers; floral bracts 5.5–6.9 mm long, narrowly triangular, densely sericeous on the outer surface; rachises sericeous. ***Flowers*** 3.4–7.5 mm diam.; sepals 3–4, ovate, green, densely sericeous outside; stamens 7–18, anthers orbicular, with a dense tuft of straight white hairs on the upper half; styles fimbriate, 1.4–3.2 mm long. ***Fruits*** turbinate, with variable numbers and placement of flattened spines, densely sericeous; 3.1–5.6 × 2.3–5.6 mm including spines. ***Diploid***.

**Figure 26.**
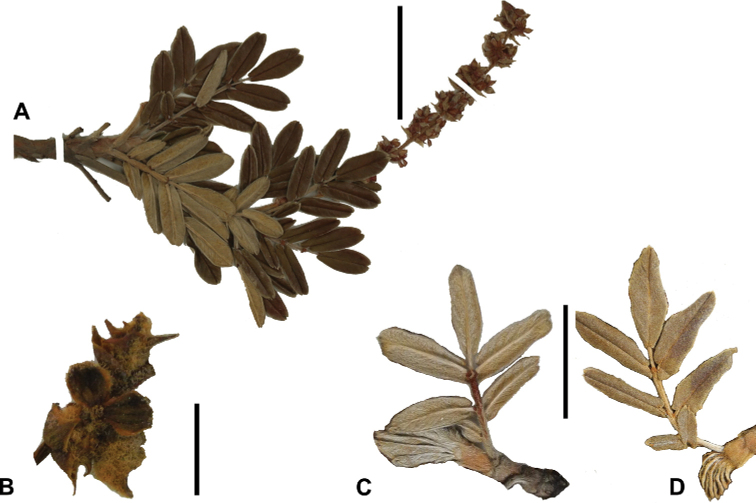
*Polylepisalbicans* Pilger **A** flowering branch **B** fruit **C** lower leaf surface **D** upper leaf surface (**A***Boertman 53***B***Smith 8210***C***Schimidt-Lebuhn 510***D***Lasermann II11*). Scale bars: 4 cm (**A**); 5 mm (**B**); 1.5 cm (**C, D**). Photographs by T. E. Boza E.

#### Distribution, habitat and ecology.

*Polylepisalbicans* occurs in north-western Peru in the Cordillera Blanca in Ancash and in the adjacent high Andes of La Libertad (Fig. 41). It grows in semi-humid montane forest at 3400–4950 m elevation, often alongside *P.weberbaueri* (Fig. 41). Where they co-occur, *P.albicans* tends to grow at lower elevations (maximum probability of occurrence at 3750–3900 m) than *P.weberbaueri* (4400 m) (Morales et al. 2018) and to generally grow in warmer and drier habitats than *P.weberbaueri* (Sevillano-Ríos and Morales 2021). Along with this, seedling density of *P.albicans* decreases with elevation, whereas that of *P.weberbaueri* increases (Morales et al. 2018). At 4350–4700 m elev. in Paria Valley, most trees of *P.albicans* are 4–7 m tall (maximum 10–12.5 m) with diameters of 10–20 cm (maximum 50–60 cm) (Castro and Flores 2015). The *Polylepis* forests in this region harbor diverse bird communities with a substantial proportion of threatened species, some of which are specialized to *Polylepis* forests (Sevillano-Ríos et al. 2011; Sevillano-Ríos and Rodewald 2017, 2021).

#### Conservation status.

The Extent of Occurrence (EOO) for *P.albicans* is estimated as 13,028 km^2^, the area of occupancy (AOO) is assessed at 164 km^2^ and it is known from 24 locations. It occurs in Huascarán National Park which encompasses almost the entire Cordillera Blanca. However, illegal mining occurs within the Park, becoming a direct threat to the species. *Polylepisalbicans* is subject to reforestation activities in Huascarán National Park (Fuentealba and Sevillano 2016). We assess the species as Vulnerable (B1a+B2a).

#### Notes.

This species was described by Pilger (1906), based on material from Caraz, Cordillera Blanca, Peru. It was synonymized under *P.sericea* by Simpson (1979) who mentioned that populations of *P.sericea* from the Cordillera Blanca are distinct by having leaflets with pronounced pubescence in both sides. Based on its distinct morphology and ecology, this taxon was re-instated at species level by Boza Espinoza et al. (2019). *Polylepisalbicans* differs from *P.sericea* by a sparse to dense sericeous hair cover on the upper leaflet surfaces and on the leaf rachises (versus glabrous in both cases in *P.sericea*), shorter hairs (0.3–0.5 mm versus 0.7–1.0 mm) and commonly reddish glandular hairs at leaflet bases (lacking in *P.sericea*). Occasionally, specimens of *P.albicans* resemble those of *P.argentea* in leaflet shape, but leaflet margins are slightly crenate in *P.albicans* and entire in *P.argentea*. Further, *P.argentea* differs from *P.albicans* by the lower number of flowers in the inflorescence (5–9 versus 18–21).

To us, the reddish glands, thick leaf texture and often emarginated leaflet apices suggest that *P.albicans* may include some genetic elements from *P.weberbaueri*, with which it co-occurs, but this remains to be tested by molecular studies.

#### Specimens examined.

**Peru. Ancash**: Carhuaz, Sonquenua, Shilla, 4020 m, 21 December 1989, *Arce & Sánchez 188* (MO); Valley of Río Marcará, 2.5 hours from Vicos on trail to Lejiacocha, 09°19'00"S, 077°31'00"W, 3600 m, 11 March 1964, *Hutchison & Wright 4325* (F, MO, USM); Shacshicucho, 4050 m, 26 August 1978, *Mostacero et al. 569* (MO); Huascarán National Park; Quebrada Ulta, north side of valley; S-facing, moderate to gentle slopes, 09°07'S,077°32'W, 3930 m, 29 July 1985, *Smith 11410* (MO, USM); Huascarán National Park. N-side of main valley, Quebrada Honda, 09°18'S, 077°25'W, 4200 m, 03 October 1985, *Smith et al. 11641* (F, MO, USM); Huascarán National Park, mouth of Quebrada Ishinca, 09°23'S, 077°29'W, 3880 m, 15 February 1985, *Smith et al. 9597* (F, MO, USM). Huaraz, Quebrada Quillcayhuanca, 4200 m, 30 October 1989, *Arce & Martel 163* (MO); Quilcayhuanca, 09° 29'53.8"S, 77°24'59.6"W, 3831 m, 08 November 2014, *Boza & Urquiaga 3022* (USM, Z); Lance, 4500 m, 04 June 2015, *Boza &Urquiaga 3144* (USM, Z); Llanganuco, 09°04'47"S, 077°38'36"W, 4445 m, 03 June 2015, *Boza & Urquiaga 3145* (USM, Z); *3146* (USM, Z); *3147* (USM, Z); Ulta, 09°06'S, 077°32'W, 4300 m, 07 June 2015, *Boza & Urquiaga 3152* (USM, Z); *3153* (USM, Z); Llaca, 09°26'S, 077°26'W, 07 June 2015, *Boza & Urquiaga 3154* (USM, Z); *3155* (USM, Z); *Boza & Urquiaga 3156* (USM, Z); 28 May 1982, *Cerrate 7696* (MO, USM); Comprado en la feria de plantas medicinales de Huaraz, 07 July 1988, *Cerrate 9123* (USM); Huascarán National Park, Quebrada Shallap, 09°30'S, 077°24'W, 3900 m, 20 February 1985, *Smith et al. 9709* (F, MO, USM). Huari, Llanganuco, 4366 m, 29 November 2007, *Lasermann I12* (USM); Huascarán National Park, southside of Quebrada Carhuazcancha, 09°28'S, 077°15'W, 4200 m, 06 May 1986, *Smith et al. 12255* (MO, USM); Huascarán National Park, Quebrada Pachachaca, a lateral valley of Quebrada Rurichinchay, 09°27'S, 077°16'W, 3860 m, 12 June 1986, *Smith et al. 12542* (F, MO); Huascarán National Park, Quebrada de Yuraccocha, a lateral valley of Quebrada Rurichinchay, 09°22'S, 077°17'W, 4300 m, 16 June 1986, *Smith et al. 12737* (MO, USM); Acopalca, 09°20'25"S, 077°12'19"W, 3300 m, 11 August 2010, *Xue-Jun 194* (USM). Huaylas, Paron, 09°02'13"S, 77°43'52"W, 3357 m, 07 November 2014, *Boza & Urquiaga 3016* (USM, Z); Huascarán National Park, Quebrada Parón, 09°01'S, 077°43'W, 3760 m, 08 May 1985, *Smith 10606* (MO); Huascarán National Park, 09°00'S, 077°41'W, 4200 m, 29 September 1985, *Smith 11537* (MO, USM); Huascarán National Park, Parón Valley, 09°00'S, 077°42'W, 4150 m, 01 January 1985, *Smith & Goodwin 8924* (AAU, F, MO, USM); Huascarán National Park, Parón Valley, 09°01'S, 077°43'W, 3700 m, 01 January 1985, *Smith & Goodwin 8939* (MO, USM); Huascarán National Park, western flank of Cordillera Blanca, Alpamayo–Cashapampa trail, 08°53'S, 077°45'W, 3950 m, 13 March 1985, *Smith & Valencia 10013* (MO, USM); Huascarán National Park, lower slopes of Cerro Pakla, 08°49'S, 077°57'W, 4300 m, 09 April 1986, *Smith et al. 12055* (AAU, F, MO, USM); Huascarán National Park, Quebrada Santa Cruz at base of and entering Quebrada Artizonraju, 08°55'S, 077°36'W, 4800 m, 16 January 1985, *Smith et al. 9298* (F, MO, USM). Yungay, Ruta Vaqueria–Portachuelo, 3900 m, 05 November 1989, *Arce 165* (MO); Huaytajirca, en el Dist. de Yanama, procedencia Matca (Yanama), 16 December 1989, *Arce & Abilio 186* (MO); 30 km, hacia arriba, Parque Nacional de Huascaran, 3850 m, 10 March 1983, *Beck 7914* (GOET, MO); Llanganuco, 09°03'04"S, 77°36'42"W, 3852 m, 07 November 2014, *Boza & Urquiaga 3013* (USM, Z); Llanganuco encima de Yungay, 4000 m, 27 June 1966, *Ferreyra 16860* (MO); Llanganuco arriba de Yungay, 4200 m, 14 December 1967, *Ferreyra & Blount 18727* (GOET, MO, USM); Llanganuco, arriba de Yungay, 3900 m, 22 October 1965, *Ferreyra & Tryon* 16503 (MO, USM); slopes below Laguna de LLanganuco in quebrada de Llanganuco ca. 25 km above Yungay, just above and below the lake, 4100 m, 27 June 1966, *Gabriel & Schunke 3826* (A, F); Dist. Yungay, Laguna de Llanganuco, 3800 m, 17 February 1968, *Gutiérrez 249* (F); Laguna de Llanganuco, 3800 m, 19 February 1968, *Gutiérrez 249-AGR* (MO); Quebrada Llanganuco, cerca de la laguna y el albergue, 3850 m, 04 July 1981, *Peréz 62* (USM); Laguna Llanganuco, 3400 m, 01 November 1984, *Sagástegui & Dillon 12315* (F, MO); near Laguna Llanganuco, 09°03'54"S, 077°38'00"W, 4300 m, 14 August 2002, *Schmidt-Lebuhn 507* (USM); near laguna Llanganuco, 09°03'54"S, 077°38'00"W, 4300 m, 14 August 2002, *Schmidt-Lebuhn 510* (USM); Huascarán National Park, Lake Llanganuco, 09°05'S, 077°39'W, 3860 m, 16 August 1984, *Smith 8210* (MO); Huascarán National Park, Llanganuco sector, Quebrada Demanada, side valley to Nevado Pisco, 09°02'S, 077°37'W, 4250 m, 13 April 1985, *Smith & Cautivo 10302* (MO, USM); Huascarán National Park, Quebrada Ranincuray, 09°00'S, 077°33'W, 3850 m, 11 January 1985, *Smith et al. 9049* (AAU, F, MO, USM); Huascarán National Park, Morococha at largest lake, 08°55'S, 077°35'W, 4550 m, 14 January 1985, *Smith et al. 9215* (AAU, F, MO, USM); Llanganuco P. N. Huascarán, 09°07'00"S, 077°37'00"W, 3475 m, 07 August 2010, *Xue-Jun 25* (USM). Cordillera Blanca near Ingenio in upper Pumapampa Valley, 11°04'S, 077°36'W, 4350 m, 15 February 1987, *Boertmann 53* (AAU); Quebrada Ishinca, Cordillera Blanca, 09°23'S, 077°28'W, 3950 m, 23 August 1988, *Frimer & Nielsen 101* (AAU); Quebrada Matará in Quebrada Ulta, Cordillera Blanca, 09°07'S, 077°32'W, 4250 m, 03 September 1988, *Frimer & Nielsen 104* (AAU); Quebrada Ulta, Cordillera Blanca, 09°06'S, 077°32'W, 4050 m, 02 September 1988, *Frimer & Nielsen 107* (AAU); *108* (AAU); Quebrada Rurichinchay, Cordillera Blanca, 09°21'S, 077°18'W, 4000 m, 06 Oct 1988, *Frimer & Nielsen 118* (AAU); *123* (AAU); Quebrada Rurec, Cordillera Blanca, 09°25'S, 077°17'W, 3950 m, 11 October 1988, *Frimer & Nielsen 125* (AAU); *Frimer & Nielsen 126* (AAU); Quebrada Carhuasccancha. Cordillera Blanca, 09°29'S, 077°15'W, 3900 m, 15 October 1988, *Frimer & Nielsen 132* (AAU); Querada Paron, Cordillera Blanca (W of Paron), 09°00'S, 077°41'W, 4150 m, 18 August 1988, *Frimer & Nielsen 42*; *43*; *44*; *45*; *59* (AAU); Quebrada Ishinca, Cordillera Blanca, 09°23'S, 077°28'W, 3950 m, 23 August 1988, *Frimer & Nielsen 70*; *71*; *73* (AAU); Quebrada Ishinca, Cordillera Blanca, 09°23'S, 077°28'W, 3950 m, 23 August 1988, *Frimer & Nielsen 74*; *99* (AAU); road from Yungay to Yauya, vicinity of Lagunas Llanganuco, 09°02'S, 077°35'W, 3800 m, 10 July 1982, *Gentry et al. 37376* (MO, USM); Llanganuco, 4377 m, 29 November 2007, *Lasermann II/1* (USM); Cordillera Blanca, Laguna Paron, 30 km NE of Caraz in northern Huascaran National Park, 4100 m, 10 October 1988, *Peterson s.n* (MO); Cordillera Blanca, East of Yungay, Laguna de Llanganuco, 3800 m, 05 April 1988, *Renvoize & Lægaard 5066* (AAU); Cordillera Blanca. 35 km east of Yungay, 4000 m, 05 April 1988, *Renvoize & Lægaard 5074* (AAU, MO); *5075* (AAU); 40 km east of Yungay., 4350 m, 05 April 1988, *Renvoize & Lægaard 5088A*; *5088B* (AAU); Llanganuco Valley, 09°00'S, 077°30'W, 1500 m, August 1959, *Tothill 174* (F); 3700 m, 1901–1929, *Weberbauer 3229* (B, MO); Parque Nacional Huascarán. Llanganuco, 11 July 1982, *Zardini 1535* (MO). **La Libertad**: Sánchez Carrión, señal Huayllides, 07°53'S, 078°02'W, 4100 m, 21 August 1982, *Smith 2278* (MO, USM).

### ﻿7. Polylepis argentea

Taxon classificationPlantaeRosalesRosaceae

T.Boza & H.Quispe, Syst. Bot.44(2): 327. 2019.

E249F1C3-AE88-5F1B-AA23-6D3F39F5AF85

Figs 27 , 28

#### Type.

Peru. Junín: Concepción, Dist. de Andamarca, a 2.5 km de la localidad de Alhuay, 11°41'30"S, 74°54'01"W, 4150 m, 10 Oct 2017, *H.R. Quispe M. 85* (holotype: CUZ!; isotypes: USM!, Z!).

**Figure 27.**
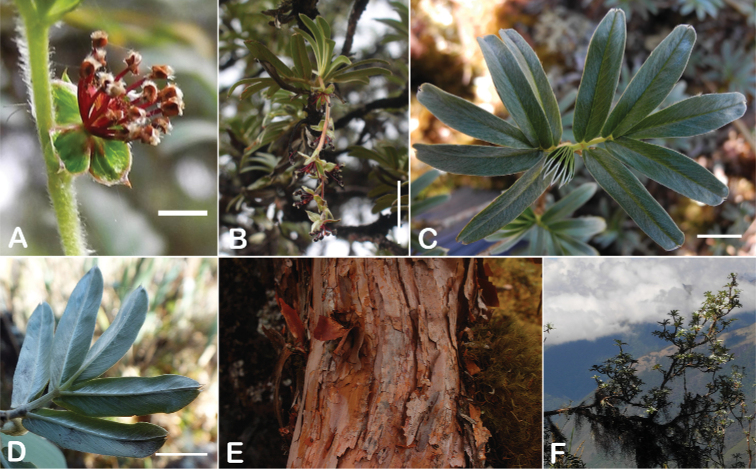
*Polylepisargentea* T.Boza & H.R. Quispe **A** flower **B** inflorescence **C** upper leaflet surface **D** lower leaflet surface **E** bark **F** branching patterns (**A, C, D, F***Boza & Urquiaga 3036***B, E***Quispe 85*). Scale bars: 3 mm (**A**); 3 cm (**B**); 1 cm (**C, D**). Photographs **A, C, F** T. E. Boza E. **B, E** H. R. Quispe **D** E. G. Urquiaga F.

#### Description.

***Trees*** 4–7 m tall. ***Leaves*** strongly congested at the branch tips, imparipinnate with 2 pairs of lateral leaflets, obtrullate in outline, (2.9)3.3–4.3 × (2.6–)3.3–4.3 cm; rachises densely sericeous, points of leaflet attachment with a tuft of long, straight hairs, sometimes with resin at leaflet insertion; stipular sheaths apically acute with spurs, densely sericeous on the outer surfaces; leaflets narrowly elliptic in outline, second pair from the terminal leaflet the largest, one of this pair (1.9)2.4–2.6 × 0.5–0.7 cm; margin entire, coriaceous, apically acute to slightly retuse, basally unequally cordate; upper leaflet surfaces almost glabrous with some hairs on the mid-veins to densely sericeous with silky hairs throughout; lower leaflet surfaces densely sericeous with silky hairs 0.6–0.9 mm long. ***Inflorescences*** pendant, 7.2–8.1 cm long, bearing 5–6(–9) flowers; floral bracts 4.5–5.6 mm long, narrowly triangular, densely sericeous on the outer surface; rachises sericeous. ***Flowers*** 7–9 mm diam.; sepals 3–4, ovate, green, densely sericeous outside; stamens 7–10, anthers orbicular, with a dense tuft of straight white hairs on the upper half; styles fimbriate, 2.7–4.4 mm long. ***Fruits*** turbinate, with variable numbers and placement of flattened spines, densely sericeous; 2.3–2.5 × 3.5–5.3 mm including spines. ***Diploid***.

**Figure 28.**
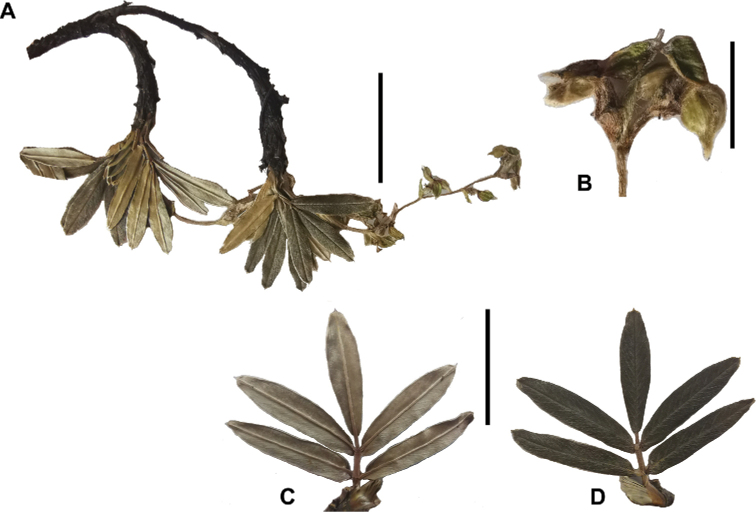
*Polylepisargentea* T.Boza & H.R. Quispe **A** flowering branch **B** fruit **C** lower leaf surface **D** upper leaf surface (**A, C, D***Quispe 85***B***Quispe 87*). Scale bars: 4 cm (**A**); 2 mm (**B, C**); 3 cm (**D**). Photographs by T. E. Boza E.

#### Distribution, habitat and ecology.

*Polylepisargentea* has been found in central Peru at La Mar (Ayacucho), Concepcion, Huancayo and Satipo (Junin) and La Convencion and Urubamba (Cusco) (Fig. 41). It grows mainly in humid Andean Forest at 3400–4400 m elevation. It often co-occurs with *P.canoi*, *P.rodolfovasquezii* and *P.serrata* (Quispe-Melgar et al. 2018) and although hybrids are not yet known, these might occur. In the Cordillera Vilcabamba, *Polylepisargentea* dominates the forest, with *P.canoi* intermixed among it and *P.serrata* in hilltop forest (Boyle 2001). The slightly different colours of the foliage of each species of *Polylepis* make this gradation obvious even from a distance (Boyle 2001).

#### Conservation status.

The estimated Extent of Occurrence (EOO) for *P.argentea* is 23,788 km^2^ and the Area of Occupancy is 40 km^2^. It is known from just eight locations, but several of these encompass forests of several square kilometers. Boyle (2001) described extensive forest of this species in the remote Cordillera Vilcabamba, which is largely protected in Otishi National Park. We assess *Polylepisargentea* as Vulnerable (B1a+B2a).

#### Notes.

*Polylepisargentea* seems morphologically closest to *P.sericea* and *P.canoi* with which it shares similar lower leaflet surface hair type and density. The most obvious differences between *P.argentea* and these species is leaflet size, with *P.argentea* having leaflets of 1.9–2.6 × 0.5–0.7 cm, whereas *P.canoi* has leaflets of 2.4–3.9 × 0.8–1.5 cm and *P.sericea* of 1.8–2.1 × 0.8–1.0 cm. Further, *P.argentea* has shorter hairs (0.6–0.9 mm versus 1.3–1.7 mm) than *P.canoi*. In *P.canoi*, the hairs on the lower leaflet surfaces are yellowish and often most pronounced on the secondary veins, whereas in *P.argentea*, they are silky and more evenly distributed. *Polylepisargentea* has the upper leaflet surfaces with a few hairs on mid-veins whereas *P.sericea* has totally glabrous upper leaflet surfaces. Additionally, the inflorescence length and number of flowers per inflorescence differ between the species, with *P.argentea* having inflorescences 7.2–8.1 cm long with 5–9 flowers, compared with values of 3.3–4.5 cm and 9–15 flowers in *P.sericea* and 8.2–14.5 cm and 12–26 flowers in *P.canoi*. The three species can also be distinguished by the number of stamens and style length, with *P.argentea* having 7–10 stamens and styles 2.7–4.4 mm long, whereas the other two species have 13–15 stamens and styles 2.4–3.8 mm in *P.canoi* and 1.9–2.5 mm in *P.sericea*.

*Polylepisargentea* was first collected by B. Boyle during scientific expeditions carried out in 1997 and 1998 to the isolated Cordillera Vilcabamba where he recorded three species of *Polylepis* (Boyle 2001). The first one, here called *P.argentea*, he called *Polylepis* sp1 and described as “a tree of 4–5 m with rather small silvery-tomentose leaflets” (specimen *Boyle 4149*) dominating the forest. A second species of *Polylepis* (here *P.canoi*) “with fewer, darker green and nearly glabrous leaflet”, which he called Polylepiscf.sericea (*Boyle 4151*), occurred patchily within stands of *Polylepis* sp1, as well as in monospecific stands. The third species mentioned was Polylepiscf.pauta (here *P.serrata*) (*Boyle 4398*), described as “a common tall tree (to 25 m high) in the tall hilltop forest”.

#### Specimens examined.

**Peru. Ayacucho**: La Mar, Dist. Tambo, Estera Community, sector Muyuorco, 12°54'19"S, 073°48'17"W, 3637 m, 29 June 2015, *Boza 3036*; *3096*; *3097*; *3098*; *3099*; *3100*; *3101*; *3102*; *3103*; *3104*; *3105*; *3106* (USM!, Z!). **Cusco**: La Convención, Dist. Huayopata Abra Málaga, 13°08'05"S, 072°19'18"W, 3802 m, 13 June 2015, *Boza 3032*; *3082*; *3083*; *3084* (USM!, Z!); Cuzco. Provincia La Convención, Bosque Qulcamachay, 4200 m, 01 October 2002, *Palomino 2030* (QCA!); Dist. Huayopata, localidad Panticalle, Abra Málaga, 13°08'02"S, 072°19'32"W, 3725 m, 30 May 2006, *Toivonen 84*; *85*; *86*; *87* (CUZ!). Urubamba, Inkatambo, 13°18'06"S, 072°31'44"W, 4340 m, 01 September 2002, *Arce s.n* (USM!); Qésqa, 13°17'51"S, 072°24'57"W, 4000 m, 01 October 2002, *Arce s.n* (USM!); Abra Málaga, 13°08'43"S, 072°18'09"W, 4318 m, 01 October 2002, *Arce s.n* (CUZ!); Inkatambo 13°18'06"S, 072°31'44"W, 3840 m, 01 September 2002, *Arce s.n* (CUZ!); Dist. Ollantaytambo, Huaytampo, 13°10'47"S, 072°21'10"W, 3650 m, 07 November 2002, *Calatayud 1035* (CUZ!, F!, MO!, USM!); Santuario Histórico Machu Pichu, camino Inca, Km 88–112, por puente Ruinas, 13°18'S, 072°07'W, 2000–4100 m, 20–21 June 1988, *Núñez 9204* (MO!); Dist. Ollantaytambo, localidad Abra Málaga, 13°09'02"S, 072°18'09"W, 4230 m, 29 May 2006, *Toivonen 15* (CUZ!); *16* (CUZ!); Dist. Ollantaytambo, localidad Huaytampo, 13°10'31"S, 072°21'03"W, 3800 m, 06 July 2006, *Toivonen 95* (CUZ!); *96* (CUZ!). **Junín**: Concepcion, Andamarca, 11°41'30"S, 074°54'01"W, 2300 m, 14 June 2002, *Martinez 18* (USM!); Dist. de Andamarca, a 2.5 km de la localidad de Alhuanay, 11°41'30"S, 074°54'01"W, 4150 m, 10 October 2017, *Quispe 85* (CUZ!, USM!, Z!). Huancayo, Dist. de Santo Domingo de Acobamba, a 5 km de la localidad de Callanca, 11°45'43"S, 074°55'15"W, 4200 m, 12 October 2017, *Quispe 87* (CUZ!, USM!, Z!). Satipo, Satipo/La Convencion Cordillera Vilcabamba Río Ene slope, near summit of divide, 11°39'30"S, 073°40'02"W, 3350 m, 07 June 1997, *Boyle 4149* (USM!).

### ﻿8. Polylepis canoi

Taxon classificationPlantaeRosalesRosaceae

W.Mend., Rev. Peruana Biol. 12(1): 104–106. 2005.

F6ECA250-9F5D-5712-84BC-16CD1792B126

Figs 29 , 30

#### Type.

Peru. Cusco: La Convención, Cordillera del Vilcabamba, 30 km caminando de la Hacienda Luisiana y del Río Apurimac, 3400 m, 17 Jul 1968, *T.R. Dudley 11180* (holotype: MO!; isotypes: NA, F!).

**Figure 29.**
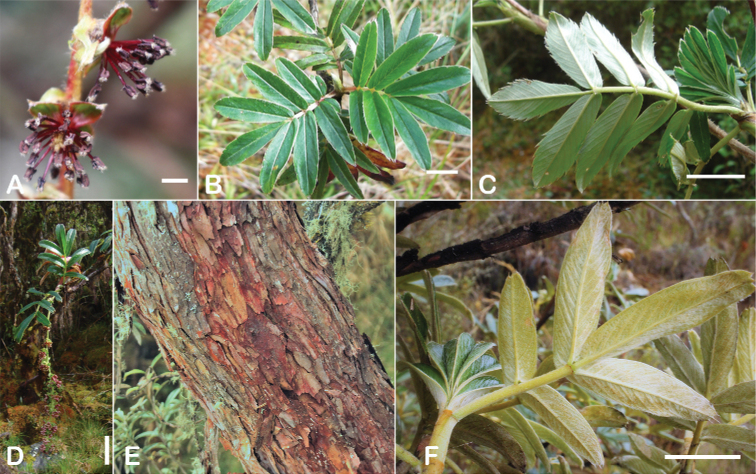
*Polylepiscanoi* W.Mend **A** flowers **B** upper leaflet surface **C** lower leaflet surface **D** flowering branch **E** bark **F** upper leaflet surface. Scale bars: 3 mm (**A**); 2 cm (**B–D, F**). Photographs **A, B** H. Huaylla **C, D** A. Fuentes **E, F** H. R. Quispe.

#### Description.

***Trees*** 4–7(9) m tall. ***Leaves*** strongly congested at the branch tips, imparipinnate with 2–3(4) pairs of lateral leaflets, obtrullate in outline, (4.0–)7.9–9.4 × (4.2–)6.7–7.5 cm; rachises densely sericeous, points of leaflet attachment with a tuft of long, straight yellowish hairs, with ferruginous resin at leaflet insertion; stipular sheaths apically acute with spurs, glabrous in both surfaces; leaflets obovate in outline, second pair from the terminal leaflet the largest, one of this pair (2.4–)3.4–3.9 × (0.8–)1.1–1.5 cm; margin entire to slightly serrate with 4–6 teeth, coriaceous, apically slightly emarginate, basally unequally cordate; upper leaflet surfaces glabrous or with sparse sericeous hairs; lower leaflet surfaces densely sericeous with yellowish hairs 1.3–1.7 mm long. ***Inflorescences*** pendant, 8.2–14.5 cm long, bearing 12–17(26) flowers; floral bracts 7.0–15.8 mm long, narrowly triangular, densely sericeous on the outer surface; rachises sericeous. ***Flowers*** 7.8–11.2 mm diam.; sepals 3–4, ovate, green, densely sericeous outside; stamens 13–15, anthers orbicular, with a dense tuft of straight white hairs on the upper half; styles fimbriate, 2.4–3.8 mm long. ***Fruits*** turbinate, with variable numbers and placement of flattened spines, densely sericeous; 5.2 × 7.5 mm including spines. ***Diploid***.

**Figure 30.**
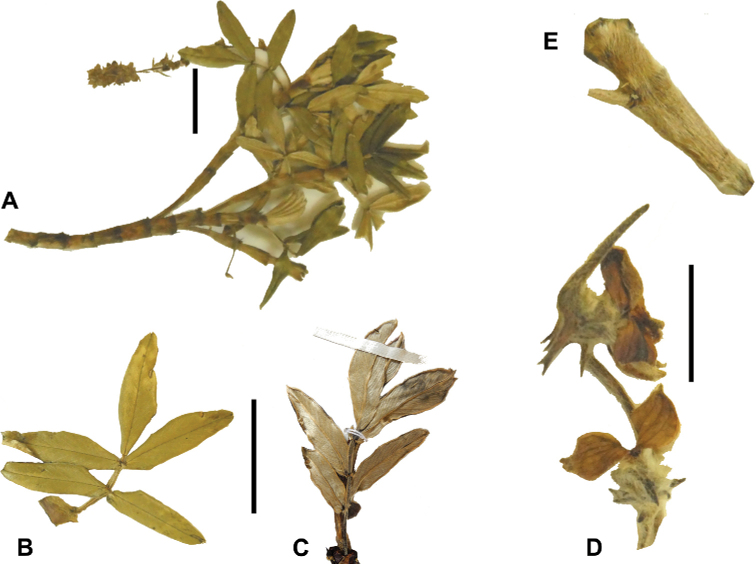
*Polylepiscanoi* W.Mend. **A** flowering branch **B** upper leaf surface **C** lower leaf surface **D** fruits **E** stipular sheaths (**A***Amez & Quispe s.n***B, D, E***Brandbyge 511***C***Boyle 4151*). Scale bars: 4 cm (**A**); 3 cm (**B, C**); 5 mm (**D**). Photographs by E. G. Urquiaga F.

#### Distribution, habitat and ecology.

*Polylepiscanoi* is distributed from the central-south-eastern Peruvian Andes to the central Bolivian Andes (Fig. 41). The species occurs in wet Andean Forest at 3150–4500 m elevation. It co-occurs with *P.argentea* and *P.serrata* in the Cordillera Vilcabamba in Peru, where it forms large pure stands and also grows intermixed with *P.argentea* (Boyle 2001). In Bolivia, where it was long known as *P.sericea*, it is only known from a few scattered localities where it has been recorded co-occurring with *P.lanata* (Kessler 1995b). In Peru, maximum tree height decreases from 9 m at 3700 m to 4 m at 4250 m elev. (Toivonen et al. 2011; as *P.sericea*). Along the same elevational gradient, the proportion of vegetative reproduction increases from 0% to 70% (Toivonen et al. 2011).

#### Conservation status.

The EOO is estimated as 98,800 km^2^ and AOO as 84 km^2^. The species is known from 17 locations in Peru and Bolivia. In Peru, it has been categorized as EN (B1ab(iii)) (León-Yañez et al. 2006) and in Bolivia, as EN (B1ab(i,ii,iii)) (Arrázola et al. 2012, as *P.sericea*). Agricultural expansion, logging, cattle, burning of surrounding grasslands and mining are threats for this species (Arrázola et al. 2012). We assess *Polylepiscanoi* as Endangered (B1a+B2a, C1).

#### Notes.

*Polylepiscanoi* seems morphologically closest to *P.ochreata* and *P.sericea*. However, it has obovate and larger (2.4–3.9 × 0.8–1.5 cm) leaflets than the other two species, which have elliptic and smaller (1.8–2.7 × 0.5–1.0 cm) leaflets. Additionally, *P.canoi* has longer hairs (1.3–1.7 mm) than the other two species (0.7–1.2 mm).

This species was treated as endemic to Peru by Mendoza (2005) when he described it. Boza Espinoza et al. (2019) revised its distribution to extend it to Bolivia. The specimens from Puno (Peru) and La Paz and Cochabamba (Bolivia) were previously determined as *P.sericea* (e.g., Kessler 1995a). Furthermore, the specimen cited by Schmidt-Lebuhn et al. (2006a) as the first record of *P.pauta* for Bolivia was re-identified as *P.canoi* by Boza Espinoza et al. (2019).

#### Specimens examined.

**Bolivia. Cochabamba**: Chapare, Mayka Mayu, 17°12'S, 065°58'W, s.d., *Hensen 2248* (BOLV, LPB, MO!, TEX); Maycamayu, ca. 60 Km N Sacaba, 17°12'S, 065°58'W, 3300 m, 11 August 1991, *Kessler 2874* (GOET!); *2875* (GOET!); *2877* (GOET!); *2878* (AAU!); *2879* (GOET!, MO!); *2880* (GOET!). **La Paz**: Bautista Saavedra, Area Natural de Manejo Integrado Apolobamba, bajada de Waricunca, mas allá de Chaka, por el antiguo camino Sorapata-Apolo, 14°53'19"S, 068°47'04"W, 3550 m, 28 March 2009, *Fuentes 13589* (BOLV, LPB, MA, MO!, USZ); Area Natural de Manejo Integrado Apolobamba, sector Chaka, bosque continuo al SE del campamento cerca de la cueva, por el antiguo camino Laji Sorapata-Apolo, 14°53'32"S, 068°47'12"W, 3461 m, 30 March 2009, *Fuentes 13634* (LPB, MO!, QCA!, USZ); *13639* (BOLV, LPB, MO!, QCA!, USZ); Área Natural de Manejo Integrado Apolobamba. Bajada de Wuaricunca, más allá de Chaka, por el antiguo camino Hilo-Hilo – Apolo, 14°53'11"S, 068°47'04"W, 3550 m, 06 April 2009, *Fuentes 13897* (BOLV, LPB, MA, MO!, QCA!, USZ); Area Natural de Manejo Integrado Madidi, Hilo Hilo. Sobre el Río Tumamayu en la localidad de Laji Sorapata, 14°53'14"S, 068°51'52"W, 4182 m, 10 April 2009, *Loza 635A* (LPB, MA, MO!); *645* (LPB, MO!, QCA!, USZ); Área Natural de Manejo Integrado Apolobamba, Hilo Hilo, Juchuy Queñua a medio día de Laji Sorapata, 14°54'52"S, 068°48'08"W, 3879 m, 16 April 2009, *Loza 757* (LPB, MO!); *775* (BOLV, LPB, MA, MO!, USZ); *788* (LPB, MO!, QCA!, USZ); Chaka Machay(Laji), 14°53'S, 068°47'W, 3300 m, 14 September 2002, *Zenteno 1507* (LPB). Franz Tamayo, Área Natural de Manejo Integrado Apolobamba, Keara bajo, 14°42'09"S, 069°04'35"W, 3500 m, 21 November 2007, *Araujo 4078* (LPB, MO!); Área Natural de Manejo Integrado Apolobamba, Hilo Hilo, Chaka, sobre la senda hacia Amantala, 14°53'16"S, 068°47'16"W, 3576 m, 16 August 2009, *Cayola 3417* (BOLV, LPB, MA, MO!, USZ); Parque Nacional Madidi, entre Queara y Mojos, sector Mosquito, 14°39'37"S, 068°57'54"W, 3400 m, 26 February 2008, *Fuentes 12028* (BOLV, LPB, MO!, QCA!, USZ); Parque Nacional Madidi, Puina Viejo, ca. 3 km río abajo por camino al W del río, 14°34'58"S, 069°06'24"W, 3316 m, 21 June 2005, *Fuentes 8549* (LPB, MO!); Parque Nacional Madidi, Hilo Hilo, arriba de la mina Kanupata en la localidad de Laji Sorapata, 14°52'28"S, 068°51'15"W, 4182 m, 11 April 2009, *Loza 671* (BOLV, HSB, LPB, MA, MO!, NY, QCA!, USZ); Bosque de Queñuari, 14°54'31"S, 069°01'07"W, 4275 m, 28 September 2006, *Palabral 489* (LPB); Senda Pelechuco-Mojo, sector Tambo Quemado, a media hora del campamento siguiendo senda Pelechuco Moxos, 14°41'03"S, 068°58'22"W, 3455 m, 01 May 2003, *Paniagua 5710* (LPB, MA, MO!). Larecaja, bosque de a localidad de Hirola, pasando Lipichi, 15°26'41"S, 068°10'57"W, 3881 m, 05 November 2008, *Palabral 705* (LPB). Murillo, 8 km after Palca on the road to Iquico, 4000 m, 10 November 1967, *Vuilleumier 342* (MO!).

**Peru. Cusco**: La Convención, Cordillera de Vilcabamba, above Camp 7, ca. 30 km walking distance from Hacienda Luisiana and the Apurimac River, 12°30'S, 074°30'W, 3400 m, 17 July 1968, *Dudley 11180* (F!, MO!, NA); usually on eastern slopes ca. 30 km walking distance NE from Hacienda Luisiana and the Apurimac River, 12°30'S, 073°30'W, 3400 m, 19 July 1968, *Dudley 11221* (F!, USM!). **Junín**: Jauja, Dist. Molinos, Comunidad Curimarca, Jucha, 11°33'53"S, 075°18'58"W, 3893 m, 10 November 2016, *Ames s.n* (Z!). Satipo, Dist. de Pampa Hermosa, Comunidad de Toldopampa, Tasta, 11°26'08"S, 074°53'58"W, 3754 m, 04 October 2016, *Ames s.n* (Z!); Junin/Cusco Prov. Satipo/La Convención, Cordillera Vilcabamba. Río Ene, slope near summit of divide, 11°39'30"S, 073°40'02"W, 3350 m, 07 June 1997, *Boyle 4151* (USM!). **Puno**: Limbani, Huancasayani, on road to Limbani just east of Abra Aricoma, 14°13'S, 069°42'W, 3750 m, 28 March 1987, *Boertmann 129* (AAU!, QCA!); Huancasayani between Abra Aricoma and Limbani, 14°13'S, 069°42'W, 3750 m, 28 March 1987, *Brandbyge 511* (AAU!).

### ﻿9. Polylepis frontinensis

Taxon classificationPlantaeRosalesRosaceae

T.Boza & M.Kessler sp. nov.

C91C084C-3A22-5564-8D92-F3FD9698F05B

urn:lsid:ipni.org:names:77301629-1

Figs 31 , 32

#### Diagnosis.

This species differs from *Polylepisquadrijuga*Bitter (1911) in having obovate leaflets with shorter villous hairs and a lower number of stamens and from *P.sericea* Wedd. (1857) by obovate leaflets (versus elliptic leaflets), serrate leaflet margins (versus entire) and longer styles.

**Figure 31.**
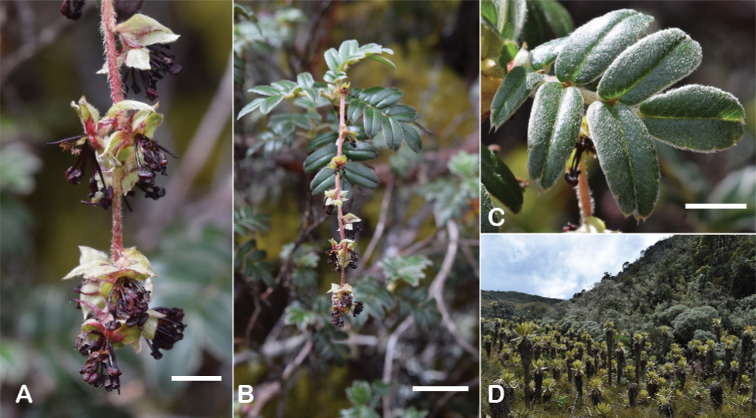
*Polylepisfrontinensis* T.Boza & M.Kessler **A** flowers **B** flowering branch **C** upper leaflet surface **D** habit. Scale bars: 5 mm (**A**); 2 cm (**B**); 1 cm (**C**). Photographs by M. J. Sanin.

#### Type.

Colombia. Antioqui: Urrao, Páramo Frontino, 06°30'N, 76°07'W, 3400 m, 5 Sep 2000, *J.A. Perez & N. Parra 1477* (holotype: MEDEL!)

**Figure 32.**
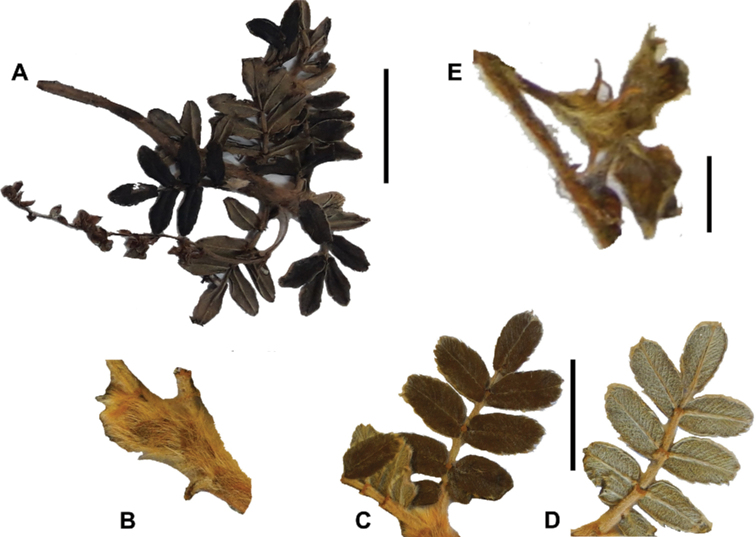
*Polylepisfrontinensis* T.Boza & M.Kessler **A** flowering branch **B** stipular sheaths **C** upper leaf surface **D** lower leaf surface **E** fruits (**A***Alvarez 84***B–E***Kessler 14777*). Scale bars: 5 cm (**A**); 3 cm (**C, D**); 3 mm (**E**). Photographs by T. E. Boza E.

#### Description.

***Trees*** 4–8 m tall. ***Leaves*** only slightly congested at the ends of the branches, imparipinnate with 3–4(5) pairs or lateral leaflets, obtrullate in outline, (2.5–)3.3–5.2 × (1.8–)2.3–3.5 cm; rachises villous, point of leaflet attachment with a tuft of long, straight hairs, slightly resinous, stipular sheaths acute at the apex with spurs, densely sericeous on the outer surface; leaflets obovate in outline, second pair from the terminal leaflet the largest, one of this pair (1.1–)1.4–2.0 × 0.4–0.8 cm; margin serrate with 5–6 teeth, coriaceous, apically slightly emarginate, basally unequally cordate; upper leaf surfaces glabrous with few trichomes in the mid-vein depression; lower surfaces densely villous with hairs 1.4–1.8 mm. ***Inflorescences*** pendant, 6.3–10.6 cm long, bearing 7–15 flowers; floral bracts 4.5–5.4 mm long, narrowly triangular, sparsely villous on the outer surface; rachises sparsely villous. ***Flowers*** 7.5–8.2 mm diam.; sepals 3–4, ovate, densely villous outside; stamens 9–11; styles fimbriate, 2.8–3.2 mm long. ***Fruits*** turbinate, with variable number of spines, densely villous; 3.3–3.6 × 4.7–5.6 cm including spines. ***Diploid***.

#### Distribution, habitat, and ecology.

*Polylepisfrontinensis* occurs in north-western Colombia in the Páramo Frontino, also called Páramo del Sol (Fig. 41). It grows at the upper limit of humid montane cloud forest at 2900–3680 m elevation. *Polylepisfrontinesis* forms 4.9% (132.74 ha) of the total area of *Polylepis* forest identify for Colombia (Fadiño and Caro 2009, as *P.quadrijuga*). It occurs in small populations in a matrix of mixed forest dominated by species of the genera *Escallonia*, *Hesperomeles*, *Myrsine* and *Weinmannia* (Rangel-Ch and Arellano 2010). Some populations occur along streams, where they are mixed with *Gynoxysbaccharoides* (Rangel-Ch and Arellano 2010).

#### Etymology.

This species is named after the Páramo Frontino to which its distribution appears to be restricted.

#### Conservation status.

*Polylepisfrontinensis* is restricted to the upper humid montane cloud forest limit in the Páramo Frontino. Its estimated extent of occurrence (EOO) and area of occupancy (AOO) are 24 km^2^. The area of distribution of the species is largely unprotected, except for the private Reserva Colibrí del Sol which only includes a few hundred individuals of this species (M. Kessler, pers. obs.). In addition, there is evidence of clearance of *Polylepis* forests elsewhere in the páramo (Rangel-Ch and Arellano 2010). We assess the species as Critically Endangered (B2ac, C2a).

#### Notes.

The populations of *Polylepis* from Páramo Frontino have previously been identified either as *P.quadrijuga* or *P.sericea* (e.g., Rangel-Ch and Arellano 2010, Fajardo-Gutierrez et al. 2018). Indeed, *P.frontinensis* resembles *P.quadrijuga* in having 3–4 lateral leaflet pairs and long inflorescences with numerous flowers. However, it has obovate leaflets with villous hairs 1.4–1.8 mm long, whereas *P.quadrijuga* has elliptic leaflets with tomentose hairs 0.7–0.9 mm long. Additionally, *P.frontinensis* is morphologically similar to *P.lanuginosa* and *P.sericea* with which it shares similar lower leaflet surface hair density and the number of stamens (13–15). The most obvious differences between *P.frontinensis* and these species is leaflet shape, with *P.frontinensis* having obovate leaflets, whereas the other two species have elliptic leaflets. Furthermore, *P.frontinensis* has longer styles (2.8–3.2 mm) than *P.lanuginosa* and *P.sericea* (1.9–2.5 mm). In *P.lanuginosa*, the hairs on the lower leaflet surfaces are yellowish and lanate, whereas in *P.frontinensis* they are whitish and villous. The three species can also be distinguished by the leaflet margins, with *P.frontinensis* having serrate margins, *P.lanuginosa* crenate margins and *P.sericea* entire margins.

Considering the morphological intermediacy of *P.frontinensis* with *P.quadrijuga* and *P.sericea*, we hypothesize that this species might be of hybridogenic origin between members of section SericeaesubsectionSericeae and section Reticulatae.

#### Specimens examined.

**Colombia. Antioquia**: Urrao, Vereda El Chuscal, sector Alto de las campanas, hacia La Laguna Campanas, 06°27'46"N, 076°07'37"W, 3830 m, 20 June 2013, *Alvarez 84* (HUA!, MO!); Páramo del Sol, Sector Alto del Burro, 3600 m, 17 April 2011, *Alzate 4168* (HUA!); Chical, Reserva ProAves “Colibrí del Sol” Páramo frontino, 06°26'22"N, 076°05'47"W, 3300 m, 04 February 2015, *Kessler 14772*; *14773*; *14774*; *14775*; *14776*; *14777* (Z!); Páramo de Frontino, Llano Grande, 3460 m, 06 January 1984, *Londoño 51* (HUA!,MEDEL); Páramo de Frontino, 06°30'N, 076°07'W, 3400 m, 05 September 2000, *Perez & Parra 1477* (MEDEL); Páramo de Frontino, 3450 m, 22 September 1994, *Renteria 10555* (HUA!); Páramo de Frontino, Zona situada entre el 15 y la Esperanza, 2980–3680 m, 18 May 1985, *Renteria 4038* (HUA!); Páramo de Frontino, sitio Llano grande, 06°27'24"N, 076°07'22"W, 3380 m, 10 September 1986, *Roldán 315* (COL!, MO!, HUA!); Páramo frontino. Camino de Puente Largo al cerro Cuchilla de Frontino, 06°30'N, 076°07'W, 3600–3800 m, 19 July 1995, *Sánchez 2244* (COL!, MEDEL); Páramo de Frontino. Camino entre Puente Largo y Llano Grande, 06°30'N, 076°07'W, 3550–3600 m, 20 July 1995, *Sánchez et al. 2289* (MEDEL); Páramo El Sol, Vereda La Encarnación, 06°29'12"N, 076°06'33"W, 3518 m, 24 May 2014, *Sarrazola 699* (HUA!). Páramo Frontino, near Llano Grande, 3450 m, 26 October 1976, *Boeke 235* (MEDEL, MO!).

### ﻿10. Polylepis humboldtii

Taxon classificationPlantaeRosalesRosaceae

T.Boza, K.Romoleroux & M.Kessler, Phytoxa 454(2): 113. 2020

2EDD9FF2-7E47-58C5-A1B5-7640747C7AEA

Figs 33 , 34

#### Type.

Ecuador. Chimborazo: Lagunas de Atillo, 02°08'S, 78°34'W, 3465 m, 17 Dec 2019, *K. Romoleroux, T.E. Boza E. & E. Bastidas 6199* (holotype: QCA!; isotype: Z!).

**Figure 33.**
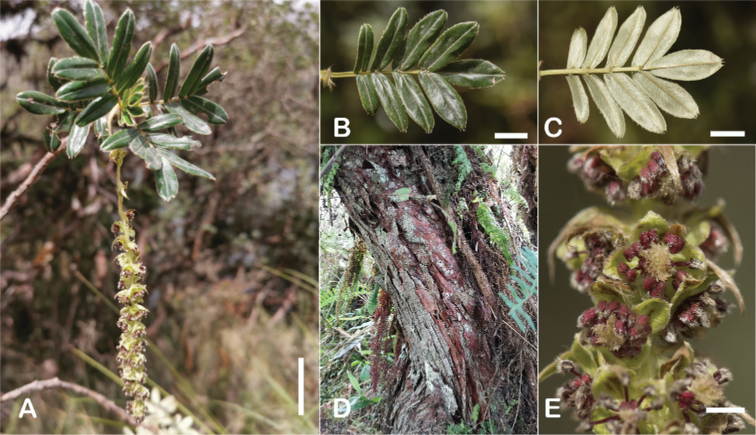
*Polylepishumboldtii* T.Boza, K. Romol. & M.Kessler **A** flowering branch **B** upper leaflet surface **C** lower leaflet surface **D** bark **E** flowers (**A–E***Romoleroux et al. 6199*). Scale bars: 3 cm (**A**); 1 cm (**B, C**); 3 mm (**E**). Photographs **A, D** T. E. Boza E. **B, C, E** E. Bastidas.

#### Description.

***Trees*** 4–12 m tall. ***Leaves*** strongly congested at the branch tips, imparipinnate with 3–4 pairs of the lateral leaflets, obtrullate in outline, 4.5–6.3 × 3.4–4.3 cm; rachises densely sericeous, points of leaflet attachment with a tuft of long, straight whitish hairs; stipular sheaths apically acute with spurs, densely sericeous in the upper surface; leaflets elliptic in outline, second pair from the terminal leaflet the largest, one of this pair 1.8–2.8 × 0.6–0.9 cm; margin entire, apically emarginate, basally unequally cordate; upper leaflet surfaces glabrous; lower leaflet surfaces densely sericeous with whitish hairs 0.2–0.4 mm. long. ***Inflorescences*** pendant, 13.0–17.9(–20.4) cm long, bearing 23–29 flowers; floral bracts 9.3–11.1 mm long, narrowly triangular, densely sericeous on the outer surface; rachises glabrous. ***Flowers*** 7.4–8.4 mm diam.; sepals 4, ovate, green, densely sericeous outside; stamens 9–15, anthers orbicular, with a dense tuft of straight white hairs on the upper half; styles fimbriate, 1.9–2.9 mm long. ***Fruits*** turbinate, with variable numbers and placement of flattened spines, densely sericeous; 3.3–5.1 × 3.1–7.4 mm including spines. ***Diploid***.

**Figure 34.**
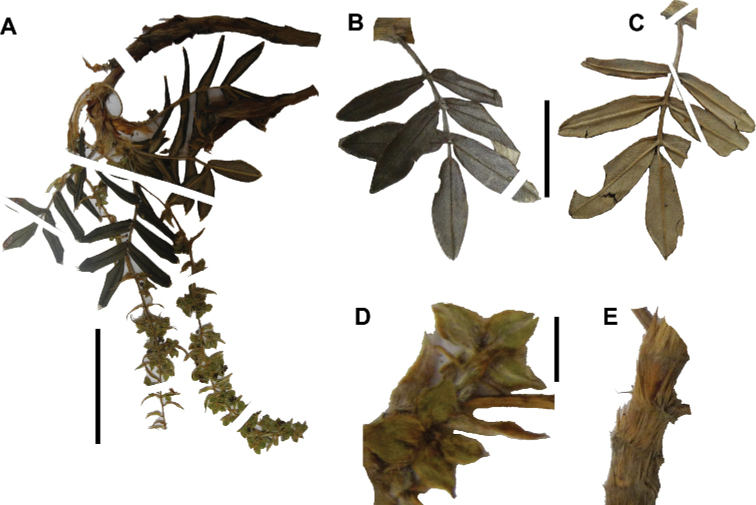
*Polylepishumboldtii* T.Boza, K.Romoleroux & M.Kessler **A** flowering branch **B** upper leaf surface **C** lower leaf surface **D** fruit **E** stipular sheats (**A, D***Carate 187***B***Carate 185***C, E***Carate 188*). Scale bars: 7 cm (**A**); 2 cm (**B, C**); 5 mm (**D**). Photographs by T. E. Boza E.

#### Distribution, habitat and ecology.

*Polylepishumboldtii* is restricted to Chimborazo Province in Ecuador (Fig. 41). It occurs in small populations in mixed Andean Forest at 3800–4000 m elevation.

#### Conservation status.

The AOO is estimated as 4 km^2^ and it has been collected at only two locations in Ecuador. Although it is protected within Sangay National Park, burning of the páramo grassland matrix likely affects the remaining *Polylepis* forest patches. Therefore, we assess *P.humboldtii* as Critically Endangered (B2a, C2).

#### Notes.

*Polylepishumboldtii* seems morphologically closest to *P.sericea* with which it shares similar leaflet shape, margin, apex and upper and lower leaflet surfaces hairs type and density. The most obvious differences between these species are leaflet hair length, with *P.humboldtii* having shorter hairs than *P.sericea* (0.2–0.4 mm versus 0.7–1.0 mm) and longer inflorescences (13.0–20.4 cm) with more flowers (23–29) than *P.sericea* (3.3–4.5 cm, 9–15 flowers). Additionally, *P.humboldtii* occurs in central Ecuadorean Andes, whereas *P.sericea* is distributed from western Venezuela to central Colombia.

#### Specimens examined.

**Ecuador. Chimborazo**: Alausí, Achupallas, alrededores, 2°17'S, 78°39'W, 3300 m, 11 July 2013, *Caranqui 2565* (QCA!); Lagunas de Atillo, 2°8'S, 78°34'W, 3465 m, 13 April 2009, *Carate et al. 184; 185; 188* (QCA!).

### ﻿11. Polylepis loxensis

Taxon classificationPlantaeRosalesRosaceae

T.Boza, K.Romoleroux & M.Kessler, Phytoxa 454(2): 118. 2020.

52023F49-F5E1-5397-B8C8-BCFB38396A93

Figs 35 , 36

#### Type.

Ecuador. Loja: Laguna Chinchilla, 03°36'20"S, 079°23'08"W, 3610 m, 21 Dec 2019, *T.E. Boza E. & C. Medina 3185* (holotype: QCA!; isotypes: Z!, CUZ!).

**Figure 35.**
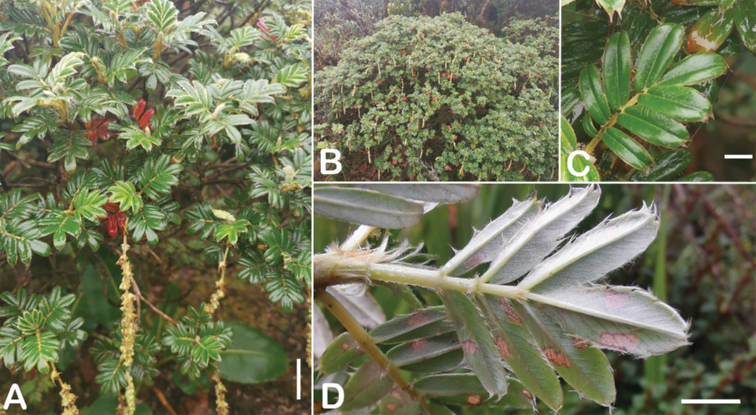
*Polylepisloxensis* T.Boza, K. Romol. & M.Kessler **A** flowering branch **B** habit **C** upper leaflet surface **D** lower leaflet surface (**A–D***Boza & Medina 3185*). Scale bars: 2 cm (**A**); 0.5 cm (**C, D**). Photographs by T. E. Boza E.

#### Description.

***Trees*** 4–10 m tall. ***Leaves*** strongly congested at the branch tips, imparipinnate with 3–4(–5) pairs of the lateral leaflets, obtrullate in outline, 2.6–3.6 × 2.1–3.2 cm; rachises densely sericeous, points of leaflet attachment with a tuft of long, straight whitish hairs; stipular sheaths apically truncate, densely sericeous in the upper surface; leaflets narrowly to broadly obovate in outline, second pair from the terminal leaflet the largest, one of this pair 1.2–1.6 × 0.5–0.8 cm; margin serrate at apex with 3–4 teeth, apically emarginate, basally unequally cordate; upper leaflet surfaces glabrous with few hairs on mid-depression; lower leaflet surfaces densely sericeous with whitish silky hairs 0.2–0.6 mm long. ***Inflorescences*** pendant, (3.5–)4.3–10.5(–12.2) cm long, bearing 9–27 flowers; floral bracts 5.5–6.3 mm long, narrowly triangular, densely sericeous on the outer surface; rachises densely sericeous. ***Flowers*** 4.4–5.2 mm diam.; sepals 4, ovate, green, densely sericeous outside; stamens 7–9, anthers orbicular, with a dense tuft of straight white hairs on the upper half; styles fimbriate, 1.7–2.0 mm long. ***Fruits*** turbinate, with variable numbers and placement of flattened spines, densely sericeous; 1.7–3.0 × 1.4–1.5 mm including spines. ***Diploid***.

**Figure 36.**
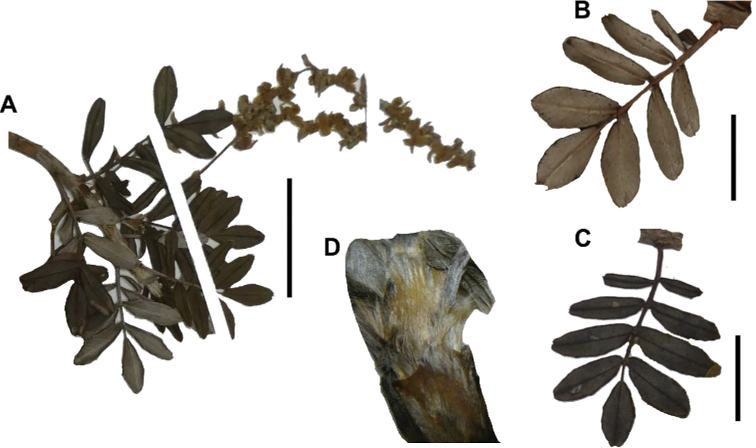
*Polylepisloxensis* T.Boza, K.Romoleroux & M.Kessler **A** flowering branch **B** lower leaf surface **C** upper leaf surface **D** stipular sheaths (**A, B***Laegaard 19109***C***Lewis 3804***D***Jørgensen 2228*). Scale bars: 5 cm (**A**); 2 cm (**B, C**). Photographs by E. G. Urquiaga F.

#### Distribution, habitat and ecology.

*Polylepisloxensis* is restricted to south-western Ecuador (Fig. 41). It has been collected in southern Azuay Province (Nabon) and at Laguna Chinchilla and Fierro Urco in northwest Loja Province bordering with El Oro Province. The páramos in this region are part of the physiographic unit called ‘Páramos del Sur’ of the Western Cordillera or Ecuador. The species occurs in the humid páramo at 2650–3700 m elevation, where it grows in mixed forest with *Gynoxiscuicochensis*, *Brachyotumledifolium* and *Weinmanniaglabra* (Lassermann 2009). In this region, a new, endemic species of hummingbird has recently been discovered (Sornoza-Molina et al. 2018), which together with this new *Polylepis* species suggests that this mountain region may be a center of endemism.

#### Conservation status.

*Polylepisloxensis* is known from six locations with an EOO of 728 km^2^ and an estimated AOO of 32 km^2^. No conservation actions have been taken to date. The area is heavily grazed by cattle and horses, pine plantations occupy large extensions and a large proportion of the area is under gold mining concessions (Sornoza-Molina et al. 2018). Based on its restricted distribution, fragmented and degraded habitat with low populations size and lack of habitat protection, we assess *P.loxensis* as Critically Endangered (A2a, B1a+B2a, C2a).

#### Notes.

*Polylepisloxensis* is most similar to *P.ochreata*, with which it shares the emarginate leaflet apices and subcordate leaflet bases and similar dense, short, white silky hair on the lower leaflet surfaces. Indeed, they were treated as conspecific by Boza Espinoza et al. (2019), but later, Boza Espinoza et al. (2020a) recognized their morphological and ecological distinctness. The two species differ in number of leaflet pairs, with *P.loxensis* having 3–4(–5) and *P.ochreata* 4–7. *Polylepisloxensis* further has shorter inflorescences (3.5–12.2 cm) bearing 9–27 flowers, fewer stamens (7–9) and shorter styles (1.7–2.0 mm), whereas *P.ochreata* has longer inflorescences (8.1–17.4 cm) bearing 21–49 flowers, more stamens (9–15) and longer styles (2.1–2.6 mm).

#### Specimens examined.

**Ecuador. Azuay**: Nabón, 3°28'20"S, 79°02'24"W, 2800–3300 m, 15 November 2008, *Salgado 1419* (LOJA!); **Loja**: Loja, Fierro Urco, 03°36'20"S, 079°23'08"W, 3610 m, 19 December 2019, *Boza & Medina 3184* (QCA!, Z!, CUZ!); Fierro Urco, Saraguro-Loja, km 12.4 turnoff towards Fierro Urco, km 23.8, 03°43'10"S, 079°19'18"W, 3840 m, 6 December 1994, *Jørgensen et al. 1240* (AAU!, LOJA!, MO!); road San Lucas–Saraguro, km 9, turn off to Fierro Urco, 11 km to the pass, 03°43'03"S, 079°19'25"W, 3630 m, 4 November 2000, *Jørgensen et al. 2228* (QCA!); ca. 10 km along road to Fierro Urco, 03°41'S, 079°01'W, 2850 m, 8 September 1998, *Laegaard 19109* (AAU!, LOJA!, QCA!); Fierro Urco, grass Páramo 12 km to the left (northbound) from the Panamericana highway, 03°43'S, 079°19'W, 3600–3650 m, 9 June 1999, *Sklenár & Laegaard 7096* (AAU!, GOET!); ca. km 12 along Páramo road to Fierro Urco, 03°41'S, 079°01'W, 3650 m elev., 9 June 1999, *Laegaard & Sklenár 20279* (AAU!, LOJA!, QCA!); Páramo of Fierro Urco SW of Saraguro, 03°43'S, 079°19'W, 3500 m elev., 21 November 1996, *Lewis et al. 2121* (AAU!); road Loja-Cuenca, km 50, track to Fierro Urco, km 5–7, 03°41'S, 079°17'W, 3150–3350 m elev., 25 October 1996, *Lewis & Lozano2724* (AAU!,LOJA!, MO!, QCA!); road Loja-Saraguro, km 52, track to Fierro Urco, km 10, 03°42'S, 079°18'W, 3350–3450 m elev., 17 January 1997, *Lewis et al. 2932* (AAU!, LOJA!, MO!); road Loja–Saraguro, 8.5 km N of San Lucas, track to Fierro Urco, km 11, 03°43'10"S, 079°19'18"W, 3550 m elev., 15 January 1998, *Lewis & Hughes 3804* (AAU!, LOJA!, MO!, QCA!); Fierro Urco, 03°41'S, 079°22'W, 3700 m elev., 11 January 1995, *P. Lozano 172* (LOJA!); Saraguro, Laguna Chinchilla, 03°36'20"S, 079°23'08"W, 3610 m elev., 21 December 2019, *Boza & Medina 3186* (QCA!, Z!, CUZ!), cerro Chinchilla, parroquía Celén, 03°35'44"S, 079°20'17"W, 3000 m elev., 19 September 1984, *Jaramillo 7332* (QCA!), *7335* (GB, QCA!); Laguna Chinchilla, 03°36'17"S, 079°23'49"W, 11 November 2008, *Salgado et al. 1392; 1394* (LOJA!).

### ﻿12. Polylepis ochreata

Taxon classificationPlantaeRosalesRosaceae

(Wedd.) Bitter, Bot. Jahrb. Syst. 45: 597–598. 1911.

D7CD40A2-3AF0-5BDD-9E71-905260BA8013

Figs 37 , 38

Polylepis ochreata var. integra Bitter, Bot. Jahrb. Syst. 45: 598, fig. 4. 1911. Type. Ecuador. Imbabura: Volcan Mojanda, Mar. 1901, Sodiro s.n. (holotype: FI n.v.; isotype: GOET!).
Polylepis subintegra Benoist, Bull. Soc. Bot. France 81: 326. 1934. Type. Ecuador. Pichincha: W slopes of Cerro Pichincha, Taurichupa, 4000 m, 28 Nov 1930, *Benoist 3356* (holotype: P!).

#### Basionym.

*Acaenaochreata* Wedd., Chlor. And. 2: 240. 1855.

#### Type.

Ecuador. Pichincha: W slopes of Cerro Pichincha, 3600 m, May 1856, *Jameson 73* (lectotype, designated by Simpson 1979, pg. 28: P; isolectotypes: A!, G!, GH!, US!; photos at F!, MO!, US!).

**Figure 37.**
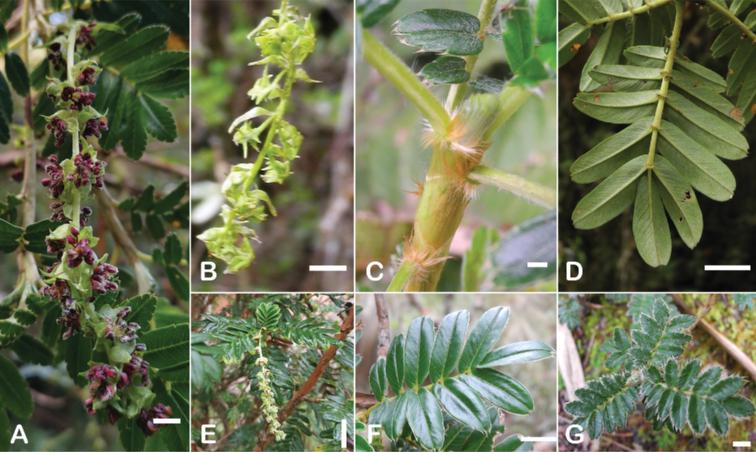
*Polylepisochreata* (Wedd.) Bitter **A** inflorescence **B** fruits **C** stipular sheaths **D** lower leaflet surface **E** flowering branch **F** upper leaflet surface **G** juvenile leaves. Scale bars: 5 mm (**A, B**); 3 mm (**C**); 1 cm (**D, F, G**); 3 cm (**E**). Photographs by T. E. Boza E.

#### Description.

***Trees*** 2–10 m tall. ***Leaves*** strongly congested at the branch tips, imparipinnate with 4–7 pairs of the lateral leaflets, obtrullate in outline, (3.9–)4.4–7.0 × 2.9–4.7 cm; rachises glabrous to densely sericeous, points of leaflet attachment with a tuft of long, straight whitish hairs; stipular sheaths apically acute, glabrous to sparsely sericeous (adult) or densely sericeous (juvenile) in the upper surface; leaflets narrowly elliptic in outline, second pair from the terminal leaflet the largest, one of this pair 1.6–3.0 × 0.5–0.7 cm; margin entire to slightly serrate with 4–6 teeth, coriaceous, apically emarginate, basally unequally cordate; upper leaflet surfaces glabrous; lower leaflet surfaces densely sericeous with whitish silky hairs 1.3–2.0 mm long in juvenile plants and 0.3–0.5 mm long in adult plants. ***Inflorescences*** pendant, 8.1–15.5(–17.4) cm long, bearing (21–)26–49 flowers; floral bracts 5.9–12.8 mm long, narrowly triangular, densely sericeous on the outer surface; rachises sericeous. ***Flowers*** 6.6–9.0 mm diam.; sepals 4, ovate, green, densely sericeous outside; stamens 9–13, anthers orbicular, with a dense tuft of straight white hairs on the upper half; styles fimbriate, 2.1–2.6 mm long. ***Fruits*** turbinate, with variable numbers and placement of flattened spines, densely sericeous; 4.7–7.5 × 6.1–7.9 mm including spines. ***Diploid***, ***tetraploid*** and ***hexaploid***.

**Figure 38.**
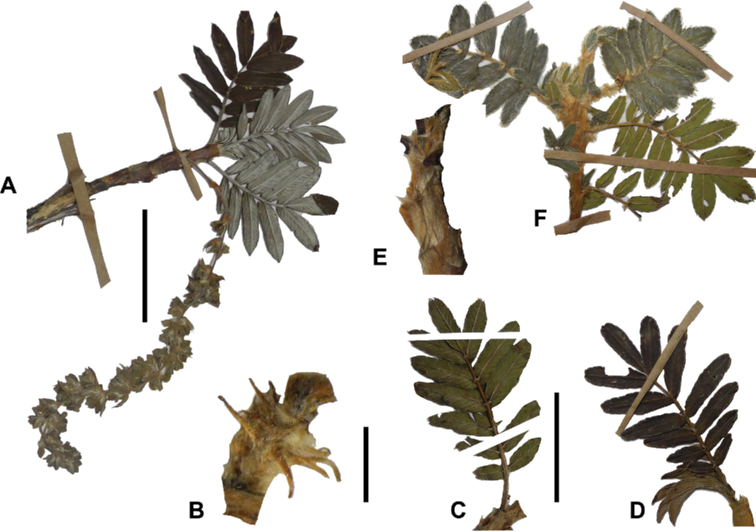
*Polylepisochreata* (Wedd.) Bitter **A** flowering branch **B** fruit **C** lower leaf surface **D** upper leaf surface **E** stipular sheaths **F** young leaves (**A***Romoleroux 350***B***Laegaard 55665***C***Romoleroux 5413***D***Romoleroux 300***E***Clark 5820***F***Ericksen 59086*). Scale bars: 6 cm (**A**); 6 mm (**B**); 4 cm (**C, D**). Photographs by E. G. Urquiaga F.

#### Distribution habitat and ecology.

*Polylepisochreata* is distributed in the Andes of Ecuador and in Nariño, southernmost Colombia (Fig. 41). It occurs at 2950–4350 m elevation in humid montane forest habitats. In northernmost Ecuador, *P.ochreata* often co-occurs with *P.longipilosa*, with which it hybridizes (Romoleroux 1996). These species have quite similar morphological characters which has complicated the taxonomic classification, mainly in the Province of Carchi, where they occur in sympatry. *Polylepisochreata* occurs in mixed population with other woody species, such as *Brachyotumledifolium* and *Miconialatifolia* (Salgado 2008). Pollen viability of *P.ochreata* has been measured as 60% (Caiza et al. 2018). In Colombia, the forest remnants of *P.ochreata* harbor the *Polylepis* specialist bird species *Conirostrumbinghami* (= *Oreomanesfraseri*) (Valderrama and Verhelst 2007).

#### Conservation status.

The EOO is estimated at 7,525 km^2^ and AOO at 112 km^2^. The species is known from 16 locations. In Colombia, remnants of *P.ochreata* forests are under pressure by the expansion of potato cropland, so that the Cumbal population has been assessed as EN and Chile’s population as VU (Rangel-Ch and Arellano 2007, as *P.sericea*). In Ecuador, *P.ochreata* is protected within the El Angel Ecological Reserve in Carchi and Yanacocha Reserve in Pichincha, where it is also subject to reforestation activities. Nevertheless, based on its restricted and fragmented distribution and continuing population losses, we assess *P.ochreata* as Vulnerable (B1a+B2a, C1).

#### Notes.

Described by Bitter in 1911, this species was synonymized under *P.sericea* by Simpson (1979), a course of action followed by Romoleroux (1996). It was re-instated at species level by Boza Espinoza et al. (2019) based on its distinctive morphology characters, including having four or more leaflet pairs, glabrous to sparsely sericeous leaf rachises and leaflet margins, short (0.3–0.5 mm long), evenly distributed hairs on the lower leaflet surfaces and 21–43 flowers per inflorescence. *Polylepisochreata* is most similar to *P.albicans* and *P.argentea*, with which it shares the elliptic leaflet shape and subcordate bases of the leaflets. However, the three species differ in number of lateral leaflet pairs, with *P.ochreata* having 4–7 pairs, *P.albicans* 3–4 and *P.argentea* 2. *Polylepisochreata* has (21)26–49 flowers per inflorescence, whereas *P.albicans* has 18–21 and *P.argentea* 5–6(–9).

Boza Espinoza et al. (2019) considered that *P.ochreata* is the only member of the *sericea* complex in Ecuador. However, K. Romoleroux recognized further variation in the country, resulting in the separation of both *P.humboldtii* and *P.loxensis* from *P.ochreata* by Boza Espinoza et al. (2020a).

#### Specimens examined.

**Ecuador. Bolívar**: Guaranda, Parroquia Salinas, recorrido entre los Arrayanes y Pambabuela, 01°22'06"S, 079°03'47"W, 3615 m, 10 Febrero 2005, *Vargas López 4696* (AAU!, K, MO!, QCNE, US!). **Carchi**: Cumbal, 00°48'19"N, 077°53'03"W, 3500 m, *Bensman 418* (MO!, WIS); Km 31 west of Tulcán on road to Maldonado, 00°52'N, 077°55'W, 3900 m, 21 June 1984, *Todzia 2485* (MO!). La Libertad (Alizo), 00°45'N, 077°59'W, *Asplund 17037* (S); Páramos de El Angel S of Volcán Chiles, 00°45'N, 077°58'W, 3850 m, 14 March 1985, *Eriksen 59086* (AAU!, MO!). Maldonado, Volcán Los Chiles, along road 9 km W of Tufiño, 00°49'N, 077°57'W, 3500 m, 10 March 1992, *Lægaard 101661* (AAU!, GOET, QCA!); Tufiño, Road Tulcán-Maldonado, near Volcán Chiles, 00°48'N, 077°56'W, 3850–4000 m, 16 August 1985, *Lægaard 54966* (AAU!, MO!, QCA!); S slopes of volcán Chiles, 14–16 km W of Tufiño on road to Maldonado, 0–1 km S of the road, 00°47'N, 077°57'W, 3850–3900 m, 18 January 1988, *Molau 2536* (AAU!, GB, MO!, QCA!); a 33 km de Tulcán, 00°48'N, 077°54'W, 3900 m, *Romoleroux 173* (AAU!, QCA!); Carretera Tulcán-Tufiño-Maldonado, 00°47'N, 077°57'W, 3800–3900 m, 12 October 1986, *Romoleroux 189* (AAU!, QCA!); Tulcán, 33.4 km W of Tulcán on road to Maldonado, Páramo de Chilos on Colombia border, 00°48'19"N, 077°53'03"W, 3900 m, 22 September 1979, *Gentry 26342* (AAU!, MO!, QCA!). **Cotopaxi**: Toacaso, Quebrada Faldiguera, 00°41'S, 078°45'W, 3750 m, 16 February 1991, *Jørgensen 93000* (AAU!, MO!, QCA!). Imbabura: Gonzalez Suarez, Laguna Mojanda, camino, forêt d’altitude, 00°08'N, 078°15'W, 2500 m, 01 February 1996, *Billiet 6762* (BR, MO!). La Merced de Buenos Aires: at road Chauasqui–Merced de Buenos Aires, km 20, near pass, 00°33'N, 078°17'W, 3700–3850 m, 10 December 1984, *Lægaard 53475* (AAU!, MO!, QCA!). Otavalo: forested path to Laguna Mojanda (La via antigua a Mojanda por el cementerio), 00°10'00"N, 078°15'00"W, 3800 m, 31 December 2000, *Clark 5820* (QCA!, US). San Rafael, W slopes of Volcán Cayambe, 00°10'00"N, 078°15'00"W, 3700–3900 m, 27 July 1967, *Sparre 17789* (AAU!, S). **Napo**: Nono, N-side of Volcán Pichincha above Hacienda Yanacocha, 00°07'S, 078°34'W, 3950–4050 m, 02 June 1985, *Lægaard 54457* (AAU!, MO!, QCA!). **Pichincha**: along, Northern slopes of Cerro Corazón, 2–4 km W along on the road to Hacienda El Pongo, 00°28'S, 078°36'W, 3100–3200 m, 13 May 1979, *Holm-Nielsen 18007* (AAU!, MO!); Corazón, 00°31'53"S, 078°39'36"W, 3260 m, *Sodiro s.n.* (AAU!); Lloa, Volcán Atacazo, W slope, 17 km from San Juan, 00°20'S, 078°38'W, 2850 m, 25 August 1980, *Holm-Nielsen 25115* (AAU!); *25148* (AAU!); Volcán Atacazo, SW slope, km 19 from San Juan, 00°21'S, 078°39'W, 2900 m, 25 August 1980, *Holm-Nielsen 25169* (AAU!); West-side of Volcán Atacazo, along drinkwater canal, 00°20'S, 078°38'W, 3700–3750 m, 11 August 1984, *Lægaard 52639* (AAU!, MO!, QCA!); *52641* (AAU!); along drinkwater-canal on W-side of Atacazo, ca. 5 km S of Campamento, 00°20'S, 078°38'W, 3700–3800 m, 24 October 1984, *Lægaard 53256* (AAU!); along drinkwater-canal on W-side of Atacazo, ca. 5 km S of Camparmento, 00°20'S, 078°38'W, 3750 m, 28 October 1984, *Lægaard 53259* (AAU!); *53260* (AAU!); along drinkwater-canal on W-side of Volcan Atacazo, 00°20'S, 078°38'W, 3200 m, 24 November 1985, *Lægaard 55665* (AAU!, GOET, MO!, QCA!); Volcán Atacatzo, 00°20'S, 078°37'W, 3500 m, *Mille 364* (US); carretera Quito–San Juan–San José de la Victoria, 00°17'53"S, 078°38'20"W, 2900–3400 m, 24 December 1987, *Zak 3265* (AAU!, GB, MO!); Nono, Camino Yanacocha NW of Volcan Pichincha, 00°05'S, 078°33'W, 3200–3800 m, 03 October 1981, *Balslev 2049* (AAU!, MO!, NY, QCA!); 28 November 1930, *Benoist 3356* (P); Yanococha, faldas noroccidentales, 00°07'S, 078°35'W, 22 March 1987, *Jaramillo Asanza 9573* (AAU!, NY, QCA!); *9588* (AAU!, QCA!); N-side of Volcán Pichincha above Hacienda Yanacocha, 00°07'S, 078°34'W, 3800 m, 04 June 1985, *Lægaard 54458* (AAU!, MO!); *54459* (AAU!); *54462* (AAU!, QCA!); *54463*; *54467* (AAU!, MO!); *54474*; *54476*; *54477* (AAU!, MO!, QCA!); Carretera Quito-Nanegalito-Santa Ana del Tablón, desvío Hda Yanacocha km 1–10 desde el desvío, 00°07'S, 078°34'W, 3500–3600 m, 06 December 1992, *Romoleroux 1495A* (AAU!); Yanacocha, 3617 m, 28 November 2008, *Romoleroux 5342* (QCA!); Yanacocha, sector La Despensa, 00°07'52"S, 078°35'06"W, 3837 m, 14 Febrero 2009, *Romoleroux 5413* (MO!, QCA!); Reserva Yanacocha, Trocha “Inca” 1–600 m, 00°06'44"S, 078°34'24"W, 3536 m, 11 June 2011, *Ulloa Ulloa 2171* (MO!, QCA!); carretera Quito-Nono-Tandayapa, desviación a Yanacocha en la localidad de Guanto-Pugro, en la hacienda “Alto Perú”, estribaciones N.O. del Volcán Pichincha, 00°05'S, 078°35'W, 3200–3300 m, 17 November 1987, *Zak 2946* (AAU!, GB, MO!); Quito, SW-slopes of volcan Atacazo, 00°20'S, 078°35'W, 3650 m, 11 October 1984, *Brandbyge 42817* (AAU!, MO!, QCA!); SW-slopes of volcán Atacazo, 00°20'S, 078°35'W, 3700–3800 m, 28 October 1984, *Brandbyge 42837* (AAU!, MO!, QCA!); Volcán Pichincha, N slopes, road to Hda. Yanacocha from pass on Quito-Nono road, km 7–11.2, 00°07'S, 078°33'W, 3600–3500 m, 12 October 1991, *Øllgaard 99187* (AAU!); Carretera a San Juan-Atacazo, km 1–12, 00°20'S, 078°35'W, 3700–4000 m, 02 September 1990, *Romoleroux 1060* (AAU!, QCA!); Tocachi, 00°08'N, 078°16'W, 3260 m, *Asplund 17103* (S); 00°08'N, 078°16'W, *Benoist 4549* (S); NW side of Pichincha, 00°08'N, 078°16'W, *Fagerlind s.n* (S); 00°08'N, 078°16'W, *Holmgren 664* (S); 00°08'N, 078°16'W, *Jameson s.n* (MO!); Páramo de Mojanda, at Laguna Negra and S-side of Laguna Grande, 00°08'N, 078°16'W, 3800 m, 14 May 1985, *Lægaard 54316A* (AAU!, QCA!); 00°08'N, 078°16'W, *Romoleroux 1495* (AAU!, QCA!); *243* (QCA!); *245* (NY, QCA!); *305* (QCA!); 00°08'N, 078°16'W, 3700 m, *Romoleroux 350* (QCA!).

### ﻿13. Polylepis sericea

Taxon classificationPlantaeRosalesRosaceae

Wedd., Chlor. Andina 2: 238. 1857.

86C022DB-082A-5733-ADA6-8EDE72C5681A

Figs 39 , 40

Polylepis hypargyrea Bitter, Bot. Jahrb. Syst. 45: 600. 1911. Type. Venezuela. Páramo de la Culata, Sierra Nevada *Moritz 1120* (holotype: B destroyed; isotypes: BM!; photos at F!, GH!).
Polylepis quindiensis Cuatrecasas, Revista Acad. Colomb. Ci. Exact. 4: 343 .1941. Type. Colombia. Caldas: Cordillera Central, W of Macizo del Quindio, Nevado del Ruiz, 3400–3500 m, 5 May 1940, *Cuatrecasas 9327* (holotype: COL!; isotypes: BC!,US!).

#### Type.

Venezuela. Mérida: Sierra Nevada, 3500 m, Jun 1847, *Funck & Schlim 1546* (lectotype, designated by Simpson 1979, pg. 28: P!; isolectotypes: G!; phot at F!).

**Figure 39.**
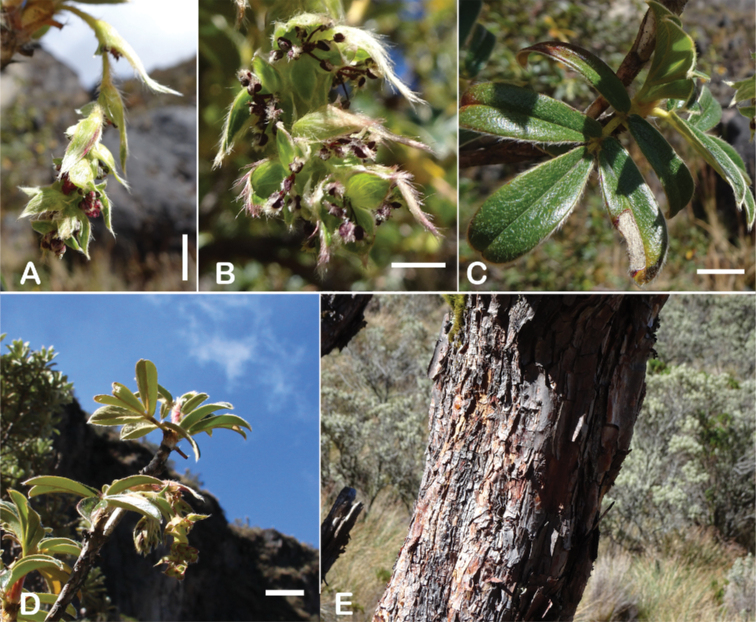
*Polylepissericea* Wedd **A** inflorescence **B** flowers **C** leaves **D** flowering branch **E** bark. Scale bars: 5 mm (**A**); 2 mm (**B**); 1 cm (**C, D**). Photographs by A. Möhl.

#### Description.

***Trees*** 3–7(12) m tall. ***Leaves*** strongly congested at the branch tips, imparipinnate with 2–3(–4) pairs of lateral leaflets, obtrullate in outline, 3.9–4.2 × 2.5–3.8 cm; rachises glabrous, points of leaflet attachment with a tuft of long, straight whitish hairs; stipular sheaths apically acute with spurs, almost glabrous with some hairs at the edges on the outer surfaces and glabrous in the inner surfaces; leaflets elliptic in outline, second pair from the terminal leaflet the largest, one of this pair 1.8–2.1 × 0.8–1.0 cm; margin entire, coriaceous, apically emarginate to retuse, basally unequally cordate; upper leaflet surfaces glabrous; lower leaflet surfaces densely sericeous with whitish hairs 0.7–1.0 mm long. ***Inflorescences*** pendant, 3.3–4.5 cm long, bearing 9–15 flowers; floral bracts 4.1–6.4 mm long, narrowly triangular, densely sericeous on the outer surface; rachises sericeous. ***Flowers*** 4.2–8.1 mm diam.; sepals 4, ovate, green, densely sericeous outside; stamens 13–15, anthers orbicular, with a dense tuft of straight white hairs on the upper half; styles fimbriate, 1.9–2.5 mm long. ***Fruits*** turbinate, with variable numbers and placement of flattened spines, densely sericeous; 4.0–7.4 × 3.4–9.6 mm including spines. ***Diploid***.

**Figure 40.**
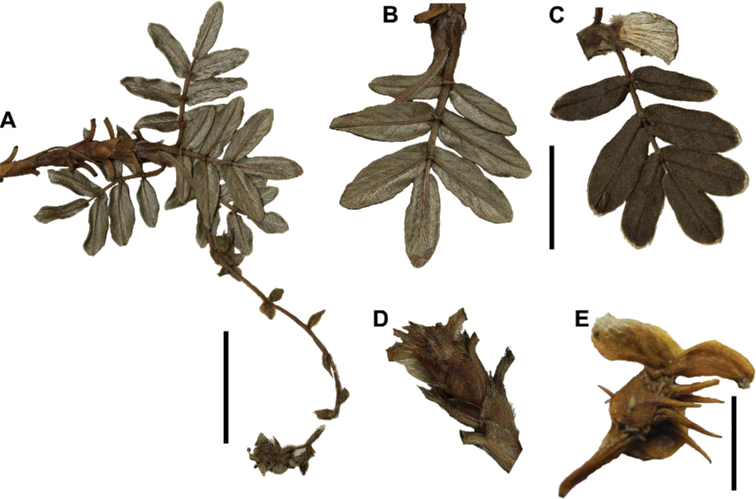
*Polylepissericea* Wedd **A** flowering branch **B** lower leaf surface **C** upper leaf surface **D** stipular sheaths **E** fruit (**A, B***Aristeguieta 7886***C, D***Berry 3812***E***Schwabe 1987*). Scale bars: 2 cm (**A–C**); 5 mm (**E**). Photographs by T. E. Boza E.

#### Distribution, habitat and ecology.

**Figure 41.**
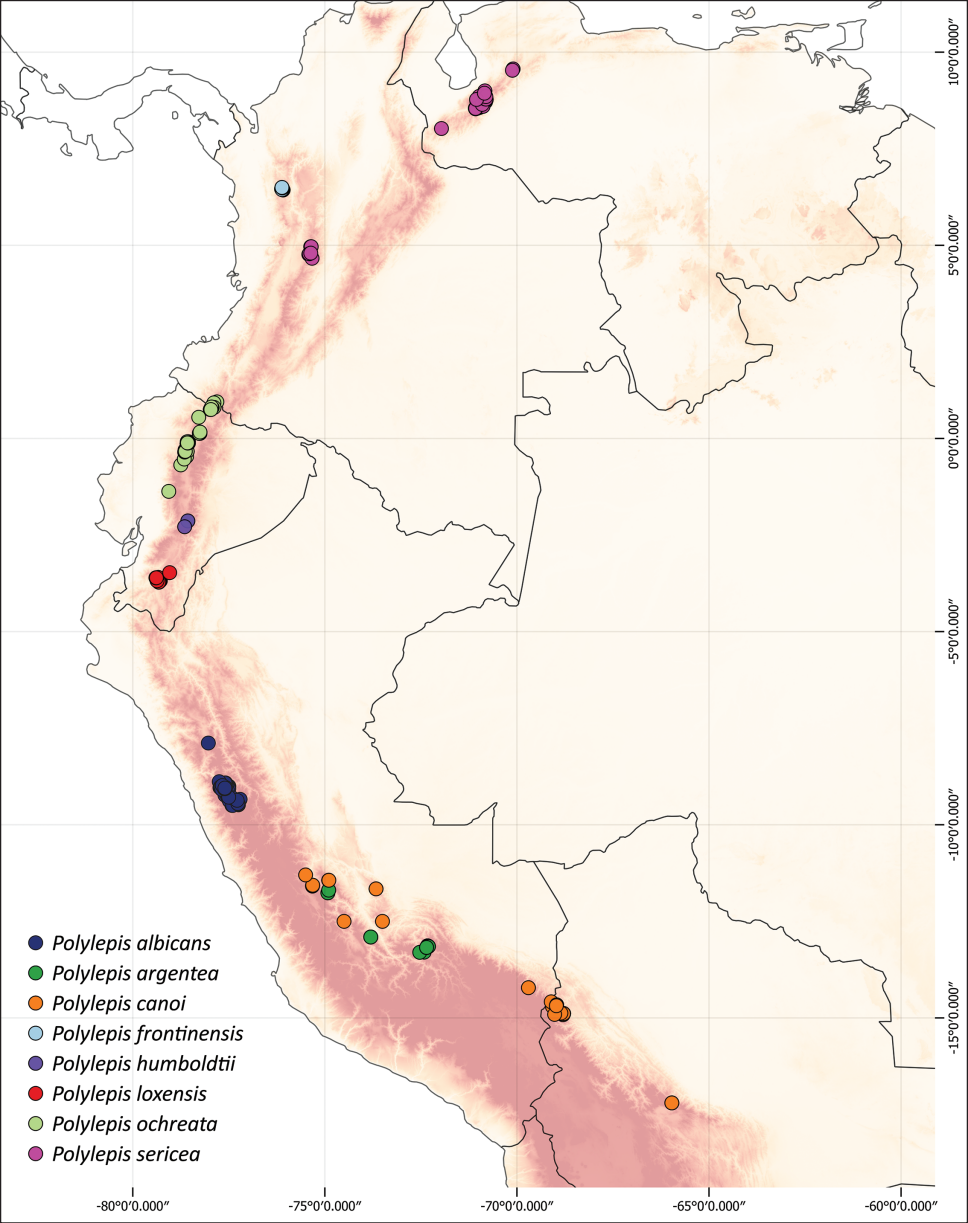
Geographical distribution of the species of subsection Sericeae.

*Polylepissericea* is found in two distinct geographic areas, the Cordillera de Mérida in the Andes of western Venezuela and the Cordillera Central of Colombia in Caldas, Quindio and Risaralda Departments (Fig. 41). It grows at 2800–4300 m elevation in humid montane forest, where it is the only species of the genus. In Venezuela, *P.sericea* mostly grows as homogeneous forest and sometimes mixed with *Hesperomelesglabrata* and *H.pernettyoides* (Arnal 1983). In Colombia, it represents 61.9% (1893.07 ha) of the total *Polylepis* forest estimated for this country (Fadiño and Caro 2009). In the Cordillera Central, it grows mixed with *Myrsineparvifolia*, *Miconiasalicifolia* and *Gynoxysbaccharoides* (Rangel-Ch and Arellano 2007). This species has been subject to detailed ecological and ecophysiological studies in Venezuela which, among other aspects, revealed that the net photosynthesis is highest at leaf temperatures of 13 °C, but is still 80% of this maximum at 3 °C and that it has high concentrations of carbohydrates in its leaves that allow supercooling down to -9 °C (Rada et al. 1985, 1996, 2009; Goldstein et al. 1994). Leaf anatomy changes with elevation to account for lower temperatures and increasing water stress at high elevations (Colmenares-Arteaga et al. 2005). As in many species of the genus, natural regeneration is highest along forest margins and in open forests, where shading is low enough to allow for seedling growth, but where herb cover is too low to outcompete them (Rada et al. 2011).

#### Conservation status.

The estimated Extent of Occurrence (EOO) for *Polylepissericea* is 36,560 km^2^. The Area of Occupancy (AOO) is 100 km^2^. The species is known from 16 locations. It is protected in Venezuela within the Sierra Nevada and Sierra de la Culata National Parks, with some minor relicts in the highest areas of the Trujillo State, where more than 50% of the remnant forest of *P.sericea* are conserved (Arnal 1983). In Colombia, forest remnants of *P.sericea* are protected within Los Nevados National Park. We assess *P.sericea* as Vulnerable (B1a+B2a).

#### Notes.

In her seminal taxonomic revision of the genus *Polylepis*, Simpson (1979) adopted a broad species concept of *P.sericea*, with the result that it was long considered the most widespread species of the genus. However, Boza Espinoza et al. (2019) subdivided the species into five morphologically, geographically and ecologically different species, namely *P.albicans*, *P.argentea*, *P.canoi*, *P.ochreata* and *P.sericea*. This treatment is also supported by the fact that, in Peru, two of these species (*P.argentea* and *P.canoi*) co-occur in mixed forests without interbreeding (Boyle 2001). Later, Boza Espinoza et al. (2020a) further separated *P.humboldtii* and *P.loxensis* from *P.ochreata*, resulting in the current recognition of seven species within what Simpson (1979) recognized as the single species *P.sericea*.

As defined by Boza Espinoza et al. (2019) and here, *P.sericea* can be distinguished from the most similar species *P.ochreata* by the number of leaflet pairs (2–3(–4) versus 4–7), leaflet margin (entire versus entire to slightly serrate), leaflet hair length (0.7–1.0 mm versus 0.3–0.5 mm), inflorescence length (3.3–4.5 cm versus 8.1–17.4 cm) and flower number (9–15 versus 21–49).

#### Specimens examined.

**Colombia. Caldas**: Pereira, El Cisne, Laguna del Otúm, 04°46'N, 075°25'W, 3900–4200 m, 20 March 2009, *Vargas 20063* (COL!). Villamaría, Cordillera central, vertiente occidental; cabeceras del río Otún, Laguna del Mosquito y plan del Villar, 04°58'N, 075°21'W, 3650–3750 m, 26 November 1946, *Cuatrecasas 23257* (COL!); Cordillera Central, vertiente occidental, vert. sudoeste del Ruiz, El Prisco, páramos, 04°58'N, 075°22'W, 3500–3600 m, 05 May 1940, *Cuatrecasas 9327* (COL!). **Quindío**: Salento, Vereda Cocóra; below Nevado del Quindio, 3800 m, 20–22 May 1989, *Luteyn 12974* (MO!). **Risaralda**: Pereira, Cordillera central, en el paso de la Laguna del Otúm hacia la Quebrada Africa, 04°47'N, 075°24'W, 4300 m, 09 February 1980, *Jaramillo 6276* (COL!).

**Venezuela. Lara**: Morán, Páramo del Jabon (Vertiente Oriental), 09°34'N, 070°06'W, 3100–3400 m, 02 November 1969, *Cuatrecasas 28216* (MERF); Páramo Jabón, camino al páramo Cendé, 09°34'N, 070°06'W, 3000–3200 m, 30 December 1999, *Riina 1036* (VEN). **Mérida**: Caracciolo Parra Olmedo, Páramo La Culata en quebrada, 08°46'43"N, 071°03'04"W, 3581 m, 07 October 2006, *Bonifacino 2541* (VEN). Justo Briceño, Páramos de Laguna Grande, 08°48'N, 070°56'W, 21 January 1929, *Pittier 13253* (MO!, VEN). Libertador, Parque Nacional Sierra Nevada. Loma Redonda Teleferico station and south, 08°33'N, 071°05'W, 4068 m, 20 May 1988, *Dorr 5220* (AAU!); Pico Bolivar, 08°33'N, 071°02'W, 4200 m, 17 January 1968, *Walter 443* (GOET!). Miranda, carretera hacia Piñango, Páramo Piedras Blancas, Dtto. Rangel, 09°00'N, 070°50'W, 3700 m, 03 March 1982, *Aymard 1050* (MO!); Dist. Justo Briceño. Páramo y chirivital en la vertiente NW del Alto del Totumo, hoya del Río Chirurí, a 19.5 km de El Aguila por la carretera a Piñango, 08°51'N, 070°49'W, 3900–4000 m, 02 April 1982, *Berry 3812* (MO!); *3844* (MO!, VEN); de El Aguila a Piñango, 08°56'24"N, 070°50'47"W, 3820 m, 03 August 2010, *Grande 2565* (VEN). Pueblo Llano, Andes de Merida/Steilhang oberhalb Laguna Negra, 08°56'N, 070°41'W, 3500–3700 m, 01 August 1958, *Schwabe s.n* (GOET!); Andes de Merida, 08°56'N, 070°41'W, 4000 m, 01 January 1973, *Schwabe s.n* (GOET!). Rangel, Margenes del Río Chama, cerca de Apartadevos, 08°47'N, 070°51'W, 01 July 1971, *Aristeguieta 7886* (MO!); Quebrada de la Mucuchache, SE de la entrada, 3600 m, 16 June 1981, *Briceño 298* (VEN); Dist. Rangel, cascada SE of Laguna de Mucubaji and below Pico Mucuñuque, Parque Nacional Sierra Nevada, 08°48'N, 070°49'W, 3600–3800 m, 15 June 1988, *Dorr 5524* (MO!, VEN); Sierra Nevada, 08°36'N, 070°53'W, 3800 m, 20 July 1934, *Farenholtz 1833* (GOET!); Sierra Nevada, 08°36'N, 070°53'W, 4000 m, 27 July 1934, *Farenholtz 1927* (GOET!); Quebrada Yoyo, 08°43'N, 070°49'W, 3880 m, 12 April 1930, *Gehriger 73* (MO!, VEN); Distr. Rangel. Sierra Nevada de Santo Domingo, road between Laguna de Mucubaji and Laguna Negra, 08°47'N, 070°48'W, 3400 m, 03 July 1979, *Kieft 87* (MO!, VEN); moraine at the head of the valley above L. Mucubají, on a small rocky cliff just above and east of the lowest falls, 08°47'N, 070°49'W, 3650 m, 21 July 1972, *Loveless 1722* (MO!); Sierra Nevada, 08°36'N, 070°53'W, s.d., *Moritz 1120* (MO!); La Nevada, 08°36'N, 070°53'W, 3352 m, 21 December 1904, *Schlim 1546* (MO!); Berghange oberhalb Laguna Negra/Páramo, 08°46'N, 070°48'W, 3700 m, s.d., *Schwabe s.n* (GOET!); Páramo de Mucubají, Páramo vegetation around Cascadas along the trail to Laguna Negra Páramo, 08°46'49"N, 070°49'16"W, 3640 m, 12 October 2007, *Sklenar 10240* (VEN); Caserio Mifafi, camino quebrada de río Chama-Caserio Mucumpis a través del páramo Piedra Blanca (entrada por la carretera Apartaderous-Pico Aguila), 08°48'N, 070°50'W, 14 August 1980, *Stergios 2116* (MO!); Páramo seco y húmedo en el sector de Sto. Domingo de Mucubají los alrededores de la Laguna de Mucubají, 08°46'N, 070°49'W, 29 May 1986, *Stergios 8378* (MO!). Santos Marquina, Sierra Nevada. Páramo alrededores de la Laguna Verde proximo Picos Humboldt y Bonpland, near edge of la LagunaVerde, 08°34'N, 070°59'W, 4000 m, 04 December 1959, *Barclay 10034* (MO!); Cerro de Caballo, 08°32'N, 070°54'W, 3600–3850 m, 25 November 1959, *Barclay 9816* (MO!); Sierra Nevada; alrededores de la Laguna Coromoto. Trail to Laguna Verde, 08°34'N, 071°00'W, 3300–3500 m, 03 December 1959, *Barclay 9951* (MO!), Parque Nacional Sierra Nevada, Mérida, Páramo Media Luna, 300 m Westl der Teleferico-Station Loma Redonda, 3920 m, 10 January 1995, *Berg 517* (VEN); Páramo del Aguila, 10 March 1951, *Croizat 66* (VEN); alrededores inmediatos de la Laguna Brava (Páramo de la Laguna Brava), sector del Páramo de los Granates, Sierra de Santo Domingo, Cordillera de los Andes, 3300 m, 20 May 1971, *López-Figueiras 8728* (VEN); Páramo, Los Colorados, 3900 m, 01 May 1988, *López del Pozo 416* (VEN); Páramo, 3550 m, July 1988, *López del Pozo 944* (VEN); Parque Nacional Sierra Nevada, Laguna Negra, 17 September 1998, *Ramirez 5533* (VEN); Laguna Mucubají, above Los Apartaderos, 3625–3655 m, 21 July 1944, *Steyermark 57513* (VEN); Laguna Negra, 3520 m, 18 May 1952, *Varechi 962* (VEN). **Tachira**: Jauregui, Páramo Sumusica along the trail heading northwest from the mountain pass (road La Grita-San Jose de Bolivar), 08°01'31"N, 071°57'53"W, 3340 m, 17 October 2007, *Sklenar 10356* (VEN). **Trujillo**: Boconó, Mun. Carache, P.N. Dinira, arriba de Mesa Arriba, debajo del Pico Cendé, ladera SO, 09°32'N, 070°07'W, 3200 m, 01 April 1999, *Duno de Stefano 767* (MO!, VEN).

### ﻿Subsection Pepea

Taxon classificationPlantaeRosalesRosaceae

T.Boza & M.Kessler sect. nov.

7C130FFC-E805-5EB2-A382-058161A6E821

urn:lsid:ipni.org:names:77301637-1

#### Diagnosis.

Shrubs or trees, 1–2 lateral leaflet pairs; lower leaflet surfaces lanate or sericeous; fruit slightly twisted with short spines, densely sericeous.

#### Type.

*Polylepispepei* B.B. Simpson.

#### Note.

The subsectional epithet *Pepea* is a noun in apposition.

### ﻿14. Polylepis pepei

Taxon classificationPlantaeRosalesRosaceae

B.B. Simpson, Smithsonian Contr. Bot. 43: 32. 1979.

DE85F3FD-EC36-59E7-9EDC-4689BF5E160A

Figs 42 , 43

#### Type.

Bolivia. Cochabamba: 77 km after Chapare on the road to Todos Santos, 4200 m, 4 Jan 1968, *Vuilleumier 465* (holotype: US!; isotypes: MO!, NY!, P!, TEX!,US!, VEN!).

**Figure 42.**
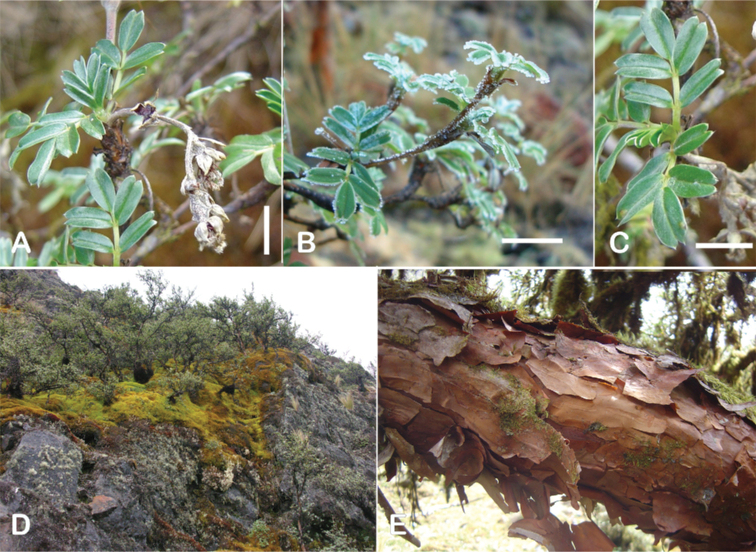
*Polylepispepei* B.B. Simpson. **A** flowering branch **B** leaves **C** upper leaf surface **D** habit **E** bark. Scale bars: 1 cm (**A, C**); 2 cm (**B**). Photographs **A–C, E** A. Fuentes **D** J. Quisbert.

#### Description.

***Shrubs or trees*** 2–7(9) m tall. ***Leaves*** strongly congested at the branch tips, imparipinnate with 2 pairs of leaflets, obtrullate in outline, (1.3–)1.7–2.6 × 1.2–2.0 cm; rachises densely sericeous, points of leaflet attachment with a tuft of long; stipular sheaths apically truncate or with spurs, densely lanate on the outer surfaces; leaflets elliptic in outline, second pair from the terminal leaflet the largest, one of this pair 0.8–1.3 × 0.2–0.7 cm; margin entire, apically emarginate or tridentate due to a projection of the mid-vein, basally unequally cordate; upper leaflet surfaces sparsely to densely sericeous; lower leaflet surfaces densely sericeous with whitish hairs 0.6–0.9 mm long. ***Inflorescences*** upright, 1.2–1.6(–3.5) cm long, bearing 3 flowers; floral bracts 4.0–7.3 mm long, narrowly triangular, densely sericeous on the outer surface; rachises densely sericeous. ***Flowers*** 4.9–5.9 mm diam.; sepals 3–4, ovate, green, densely sericeous outside; stamens 5–9, anthers orbicular, with a dense tuft of straight white hairs on the upper half; styles fimbriate, 3.0–4.9 mm long. ***Fruits*** turbinate often slightly twisted, with variable numbers and placement of short spines, densely sericeous; 2.3–5.7 × 1.9–3.9 mm including spines. ***Diploid***.

**Figure 43.**
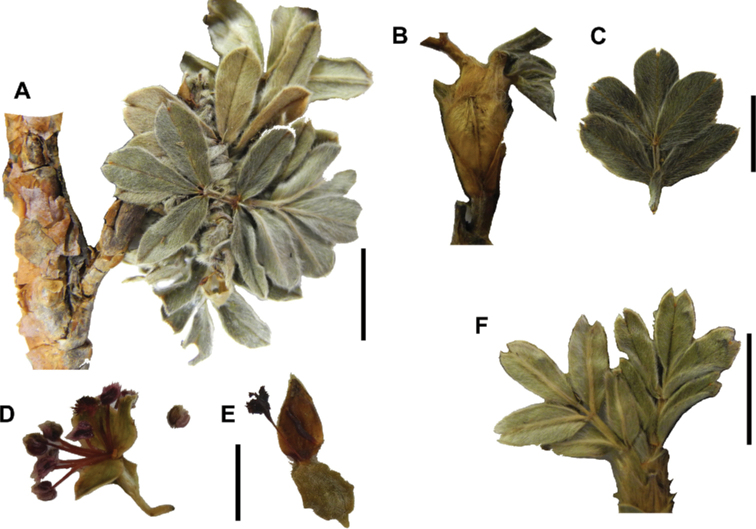
*Polylepispepei* B.B. Simpson **A** flowering branch **B** stipular sheaths **C** upper leaf surface **D** flower **E** fruit **F** lower leaves surface (**A, C–E***Kessler 3386***F***Beck 14680***D***Beck 11859*). Scale bars: 2 cm (**A**); 1.5 cm (**B, C**); 6 mm (**D**). Photographs by T. E. Boza E.

#### Distribution, habitat and ecology.

*Polylepispepei* has been found in northern to central Bolivia and southern Puno (Peru) where it has been collected at one locality in San Antonio de Putina Province close to the border with Bolivia (Fig. 24). It grows at 3550–4800 m elevation, where it typically forms the uppermost forests, often in isolated patches high above the closed treeline, often on rocky slopes (Sylvester et al. 2017). Forest remnants in sites that are topographically inaccessible to grazing animals and man-made fires support a unique flora with many previously undescribed species (Sylvester et al. 2016) that presumably represents remnants of the natural potential vegetation (Sylvester et al. 2014a). Such inaccessible forest remnants are also characterized by a high proportion (on average 30%) of dead, standing trees, which support a diverse flora of epiphytic bryophytes and lichens (Sylvester et al. 2017). In accessible forests, such dead trees are harvested by the local inhabitants as firewood (Toivonen et al. 2018). The world’s highest vascular epiphytes (*Melpomene* spp., Polypodiaceae) have been found at elevations of up to 4550 m in forest of *P.pepei* (Sylvester et al. 2014b). These forests also support the hemiparasite *Tristerixlongebracteatus* (Desr.) Barlow & Wiens (Loranthaceae) at over 4600 m elevation (Sylvester et al. 2014b). Stands of *P.pepei* are often very dense, with numerous trunks of relatively small diameters of around 10 cm (Toivonen et al. 2018). Radial tree growth of the species is enhanced by rains in the dry season and varies depending on local conditions including slope and substrate (Jomelli et al. 2012). Vegetative reproduction increases with elevation, to the degree that the uppermost stands have no reproduction by seeds at all (Hertel and Wesche 2008; Toivonen et al. 2018).

#### Conservation status.

*Polylepispepei* is known from 12 locations with an EOO of 35,111 km^2^ and an estimated AOO of 68 km^2^. *Polylepispepei* was categorized as VU (A1c) in the World List of Threatened Trees (Oldfield et al. 1998). Later, it was classified as EN (B1b(i,iii)) in the Red List of Threatened Flora of Bolivia (Arrázola and Coronado 2012). It is protected within Madidi and Carrasco National Parks of Bolivia. A stand above Unduavi has been focus of conservation attention due to the presence of the critically endangered bird species *Anairetesalpinus* (Navarro et al. 2010). Stands of *P.pepei* are severely threatened by livestock activities that involve annual burns of the grasslands that often extend into the forests. This species survives low to moderate levels of direct use by local extraction of firewood (Navarro et al. 2010). We assess *P.pepei* as Endangered (A2a, B1a+B2a, C1, D1).

#### Notes.

*Polylepispepei* is very similar to *P.rodolfovasquezii*. It differs by having two pairs of lateral leaflets (versus one pair in *P.rodolfovasquezii*) and longer inflorescences (1.2–1.6(–3.5) cm) bearing three flowers, whereas *P.rodolfovasquezii* has shorter inflorescences (0.9–1.1 cm) bearing just one flower. Additionally, *P.pepei* may be confused with *P.subsericans* and *P.flavipila* because they all share short leaflets and inflorescences. *Polylepispepei* differs from these by having two pairs of lateral leaflets and sericeous hairs, whereas the other two species have one pair of lateral leaflets and strigose hairs in *P.subsericans* and pilose hairs in *P.flavipila*.

#### Specimens examined.

**Bolivia. Cochabamba**: Chapare, Km 74 Camino antiguo a los yungas del Chapare entrando por Aguirre, 3760 m, 24 April 1999, *Mercado 2207* (MO!); 77 km. after Cochabamba on the road to Todos Santos, 4200 m, 04 January 1967, *Vuilleumier 465* (MO!, NY, US!). Tiraque, El Ronco, ceja de monte yungena, 17°00'05"S, 065°39'20"W, 3930 m, 11 May 2005, *Alcázar-Johansen 403* (BOLV); El Ronco, Ceja de monte yunguena, 17°00'05"S, 065°39'20"W, 3710 m, 11 May 2005, *Johansen 403* (MO!). **La Paz**: Bautista Saavedra, Area Natural de Manejo Integrado Apolobamba, Hilo Hilo, a una hora y media de Pallalani en direccion a Laji Sorapata, sobre el camino, 14°52'40"S, 068°55'34"W, 4300 m, 06 April 2009, *Loza 589* (LPB, MA, MO!, USZ); *590* (LPB, MO!, QCA!, USZ). Franz Tamayo, Parque Nacional Madidi, Queara, sector Quecara, Llantai Cunca, 14°39'01"S, 069°05'01"W, 21 April 2008, *Fuentes 12687* (BOLV, CTES, HSB, LPB, MA, MO!, QCA!, USZ); Area Natural de Manejo Integrado Apolobamba, Keara, hacia el NW, 14°41'03"S, 069°05'35"W, 4151 m, 17 June 2005, *Fuentes 8282* (LPB, MA, MO!, QCA!); Area Natural de Manejo Integrado Apolobamba, Waca Cocha, 4.7 km al SE de Keara, 14°43'47"S, 069°04'17"W, 18 June 2005, *Fuentes 8341* (LPB, MO!, QCA!); Area Natural de Manejo Integrado Apolobamba, Hilo Hilo, frente a Pallalani, 14°52'49"S, 068°57'09"W, 4286 m, 05 April 2009, *Loza 587* (LPB, MO!, QCA!, USZ); *588* (BOLV, LPB, MO!, QCA!, USZ); Parque Nacional Madidi, Queara nuevo, Chuñuña, queñual al N del pueblo, 14°41'04"S, 069°05'36"W, 4100 m, 09 April 2008, *Paco 1* (BOLV, DAV, HSB, LPB, MA, MO!, USZ); Area Natural de Manejo Integrado Apolobamba, Queara nuevo Toilcacocha, 14°41'12"S, 069°05'17"W, 3930 m, 11 April 2008, *Paco 80* (LPB, MA, MO!, QCA!, US!); Apolobamba, Puina, cerca de Queñuapata, 14°36'26"S, 069°05'52"W, 4365 m, 10 April 2008, *Quisbert 801* (BOLV, LPB, MA, MO!, NY, USZ); *810* (LPB, MA, MO!, USZ); Apolobamba, entre la comunidad de Puina y cerro k’akepununa, 14°36'24"S, 069°05'47"W, 4458 m, 11 April 2008, *Quisbert 821* (LPB, MA, MO!, USZ); *825* (BOLV, LPB, MA, MO!, QCA!, USZ); Apolobamba, Palomani, 14°34'58"S, 069°07'38"W, 4286 m, 12 April 2008, *Quisbert 844* (BOLV, HSB, LPB, MA, MO!, QCA!, USZ); *848* (BOLV, LPB, MA, MO!, USZ). Inquisivi, 15 Km N Villa Victoria, ca. 15 km SE Quime, 17°06'S, 067°14'W, 4050 m, 05 December 1991, *Kessler 3385* (AAU!, GOET!, MO!); *3386* (AAU!, GOET!, MO!). Murillo, entre Pongo y Unduavi, MIna 50, subiendo hacia la Mina SAn Luis, 3960 m, 28 October 1994, *Beck 21532* (LPB); Pongo bajanado a los Yungas, del pueblo Pongo subiendo a los restos del bosque de *Polylepispepei*, 16°19'32"S, 067°57'26"W, 3950 m, 10 January 2007, *Beck 29771* (LPB); Valle del Zongo entrando arriba de Botijalca (Tiquimani) haia el Este, Umapalca media hora y entrando en Valle Latera, 16°12'S, 068°03'W, 4000 m, 31 January 2004, *Beck 30014* (LPB); 14.8 km N of the pass at the head of The Zongo Valley, 16°13'S, 068°07'W, 3850–4050 m, 11 April 1987, *Brandbyge 584* (AAU!); *854* (MO!); Valle del Río Zongo. 14.8 km al norte de la cumbre, 16°12'S, 068°07'W, 3900–4000 m, 20 February 1987, *Solomon 16172* (LPB, MO!); 17.0 km al este de La Cumbre (vieja estación de ferrocarril) por el camino a Unduavi (4.2 km al oeste de Unduavi), 16°19'S, 067°55'W, 3350 m, 11 April 1988, *Solomon 18267* (LPB, MO!). Nor Yungas, arriba de Unduavi subiendo aproximadamente 45 min hacia los bosques de *Polylepispepei*, 16°18'S, 067°56'W, 4120 m, 13 September 2016, *Escobari 78* (LPB). Sud Yungas, debajo de Unduavi, subiendo el valle de Cerromarca, 3450 m, 28 August 1988, *Beck 14680* (LPB).

**Peru. Puno**: San Antonio de Putina, Tocko-Tocko, 14°43'56"S, 69°36'21"W, 4560 m, 10–12 June 1969, *Vargas 21596* (CUZ!).

### ﻿15. Polylepis rodolfovasquezii

Taxon classificationPlantaeRosalesRosaceae

L.Valenzuela & I.Villalba, Arnaldoa 22(2): 335, f. 1–2. 2015.

2F7B3106-3AD3-56BF-9734-9BCFD0C0F3E9

Figs 44 , 45

#### Type.

Peru. Junin: Satipo, Pampa Hermosa, rural community of Santa Rosa de Toldopampa, buffer area of the Bosque de Proteccion Pui-Pui, 4221 m, 11°29'33.5"S, 74°56'37.8"W, 21 Apr 2015, *Valenzuela & Rojas 28873* (holotype: HOXA!; isotypes: MO!, USM!).

**Figure 44.**
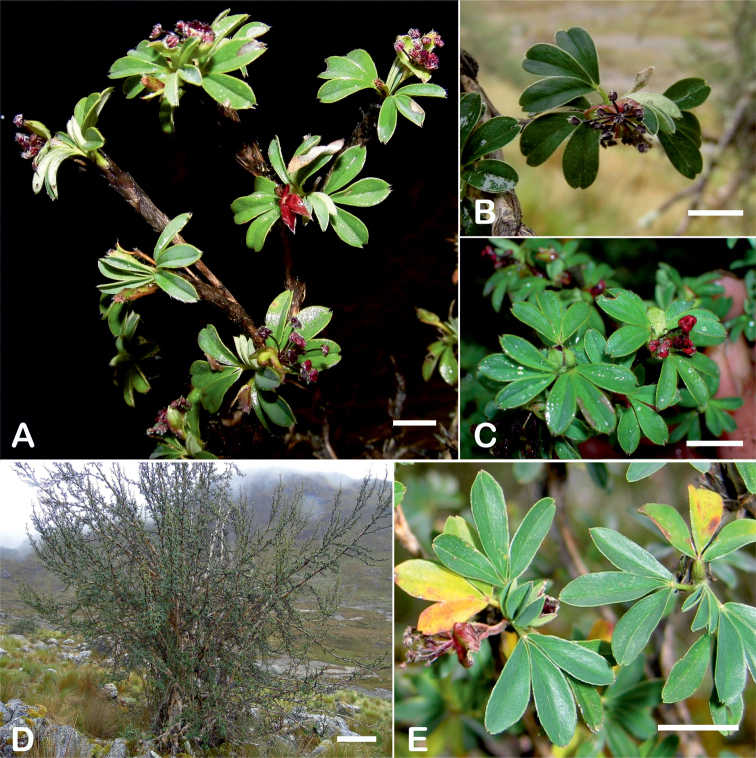
*Polylepisrodolfovasquezii* L.Valenzuela & I.Villaba **A** flowering branch **B** flower **C** flower **D** habit **E** leaves (**A, B, D***Boza et al. 3169*). Scale bars: 5 mm (**A**); 1 cm (**B, C, E**). Photographs **A, B, D** G. Vargas **C, E** T.E. Boza E.

#### Description.

***Shrubs or trees*** 1–8 m tall. ***Leaves*** strongly congested at the branch tips, imparipinnate with 1 pair of lateral leaflets, obtrullate in outline, 1.4–1.6 × 1.5–2.0 cm; rachises glabrous, points of leaflet attachment with a tuft of long hairs; stipular sheaths apically with spurs, sparsely sericeous on the outer surfaces; leaflets elliptic in outline, second pair from the terminal leaflet the largest, one of this pair 0.9–1.1 × 0.4–0.6 cm; margin entire, apically emarginate with a projection of the mid-vein, basally unequally cordate; upper leaflet surfaces glabrous to sparsely sericeous; lower leaflet surfaces sparsely to densely sericeous with whitish hairs 0.8–1.0 mm long. ***Inflorescences*** upright, 0.9–1.1 cm long, bearing 1 flower; floral bracts 4.0–4.8 mm long, narrowly triangular, densely sericeous on the outer surface; rachises glabrous. ***Flowers*** 5.7–6.6 mm diam.; sepals 3, ovate, green, densely sericeous outside; stamens 9–10, anthers orbicular, with a dense tuft of straight white hairs on the upper half; styles fimbriate, 3.6–4.1 mm long. ***Fruits*** turbinate often slightly twisted, with variable numbers and placement of short spines, densely sericeous; 4.8–6.0 × 2.6–2.9 mm including spines. ***Diploid***.

**Figure 45.**
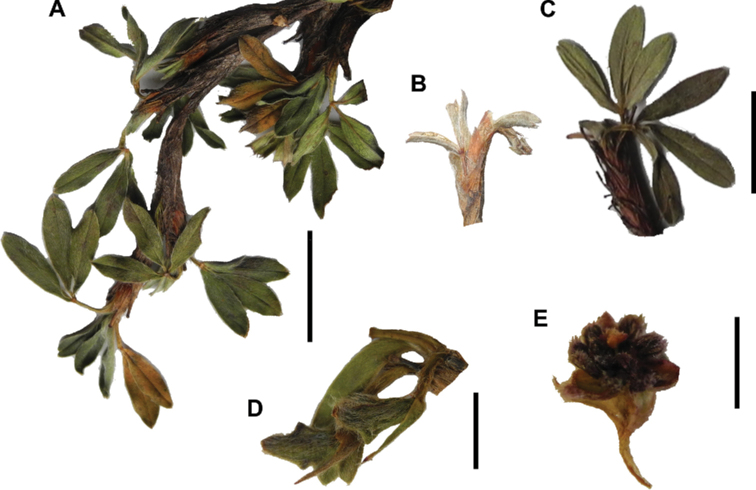
*Polylepisrodolfovasquezii* L.Valenzuela & I.Villalba **A** flowering branch **B** stipular sheaths **C** lower and upper leaf surface **D** fruit **E** flower (**A, B***Arce s.n***C–E***Toivonen 71*). Scale bars: 2 cm (**A**); 1 cm (**C**); 5 mm (**D**); 4 mm (**E**). Photographs by T. E. Boza E.

#### Distribution, habitat and ecology.

*Polylepisrodolfovasquezii* is distributed along the Eastern Cordillera of central Peru from San Martin to Cusco and Puno (Fig. 24). It occurs on wet rocky slopes at 3700–4750 m elevation. It commonly grows with trees of the genera *Escallonia*, *Gynoxys*, *Hesperomeles* and *Clethra* (Valenzuela and Villalba 2015). Growth of this species is most strongly correlated to growth season temperature (Requena-Rojas et al. 2020a, 2020b). *Polylepisrodolfovasquezii* remnants harbor several threatened bird species, including *Anairetesalpinus* (Quispe-Melgar et al. 2018, 2020).

#### Conservation status.

The estimated EOO is 164,207 km^2^ and AOO is 116 km^2^. The species is known from 19 locations. Although it is protected within the Pui-Pui Protection Forest in Junin, the species was categorized as VU for Peru (SERFOR 2006, as *P.pepei*). Burning activities for the expansion of pastures for livestock grazing are causing a reduction of the small remnants, even within the protected area. We assess *P.rodolfovasquezii* as Vulnerable (A1, B1a+B2a, C1).

#### Notes.

Specimens of this recently described species were long identified as *Polylepispepei*, based on their very similar morphology. However, *P.rodolfovasquezii* differs from *P.pepei* by having only one pair of lateral leaflets (versus two pairs) and shorter inflorescences (0.9–1.1 cm long) bearing just one flower (versus 1.2–1.6(–3.5) cm long bearing three flowers). When *Polylepisrodolfovasquezii* was described by Valenzuela and Villalba (2015), they did not realize that their newly described species had been treated as *P.pepei* since Simpson (1979). Nevertheless, Simpson (1979) already mentioned that “the collection from Peru has only one pair of leaflets, reduced inflorescences and denser covering of trichomes on the under-leaflet surfaces”. Despite these differences from the Bolivian specimens, she recognized just the single taxon *P.pepei*. This was presumably the result of the low number of specimens known for the two species at that time and the broad species concept adopted by Simpson. Clearly, the two species are closely related allopatric taxa that could conceivably also be treated as subspecies.

*Polylepisrodolfovasquezii* also resembles *P.subsericans* and *P.flavipila*. It differs from these in its shorter inflorescence (0.9–1.1 cm) bearing just one flower, whereas in *P.subsericans*, the inflorescences are 1.9–5.6 cm long with 3–6 flowers and in *P.flaviplia* 2.7–4.4 cm long with 3–5 flowers.

#### Specimens examined.

**Peru. Cusco**: La Convención, bosque de Mandor, 4200 m, 01 October 2004, *Palomino 2043* (QCA!); Dist. Santa Teresa, Mountain edges on the lower Eastern portion of the Phachaq valley, Yanama, 13°17'11"S, 072°50'13"W, 4232 m, 28 April 2012, *Sylvester 1451* (Z!); Dist. de Ollantaytambo, Mountain edges on the lower Eastern portion of the Phachaq valley, Yanama, 13°17'12”S, 072°50'13"W, 4211 m, 01 May 2012, *Sylvester 1501* (Z!); Dist. de Santa Teresa, grazed slopes in the central Pacchaq valley on the East side of the river Yanama, 13°15'40"S, 072°50'17"W, 4268 m, 04 May 2012, *Sylvester 1558* (Z!); Dist. Santa Teresa, Mountain edges on the lower Eastern portion of the Phachaq valley, Yanama, 13°17'12"S, 072°50'13"W, 4174 m, 05 May 2012, *Sylvester 1564* (Z!); Dist. de Ollantaytambo, topmost forest found on the lower North side of the lower Phachaq valley, Yanama, 13°17'01"S, 072°50'01"W, 4566 m, 15 May 2012, *Sylvester 1597* (Z!); *1598* (Z!). Urubamba, Q’esqa, 3960 m, 01 September 2002, *Arce s. n.* (USM!); Abra Malaga, 13°08'46"S, 072°18'14"W, 4284 m, 01 October 2002, *Arce s.n* (USM!); Paljay, 13°08'46"S, 072°18'14"W, 4177 m, 01 September 2002, *Arce s.n* (CUZ!); Chaupiwayco, 13°14'59"S, 072°29'10"W, 4290 m, 01 May 2002, *Arce s.n* (CUZ!); Piñasniocj, Panticalla pass, 3600 m, 15 July 1915, *Cook 1241* (US!); *1837* (US!); Cañon above Peñas ruins towards Nevado Veronica, Peñas Cañon beyond Ollantaytambo on road to Abra Malaga, 4100 m, 26 August 1989, *Driesch s.n* (GOET!); Cumbre Malaga, 01 October 1984, *Rivas s.n* (USM!); Dist. de Ollantaytambo, Congunayoc; 3.5 km 175 South of the village Thastayoc, on SE facing slope facing towards Ollantaytambo, 13°10'26"S, 072°16'06"W, 4438 m, 09 March 2012, *Sylvester 1392* (Z!); 13°10'24"S, 072°16'14"W, 4415 m, 09 March 2012, *Sylvester 1393* (Z!); 13°10'26"S, 072°16'06"W, 4427 m, 10 March 2012, *Sylvester 1396* (Z!); 13°10'22"S, 072°16'11"W, 4417 m, 10 March 2012, *Sylvester 1397* (Z!); 13°10'25"S, 072°16'14"W, 4414 m, 10 March 2012, *Sylvester 1398* (Z!); Machupicchu, Warmiwañuska, 13°14'21"S, 072°29'06"W, 4235 m, 13 September 2006, *Toivonen 67*; *68*; *69*; *70*; *71*; *72*; *73*; *74*; *75*; *80* (CUZ!); Ollantaytambo, Abra Malaga, 13°08'40"S, 072°17'51"W, 4340 m, 10 May 2006, *Toivonen 82* (CUZ!); Dist. Machupicchu, Microcuenca Pacaymayo; laguna Pacaymayo, 13°13'48"S, 072°29'48"W, 3900 m, 26 June 2001, *Tupayachi 5049* (CUZ!); Machupicchu Microcuenca Cusichaca, Sisaypampa Abra Palkay, 13°20'00"S, 072°30'44"W, 4100 m, 28 June 2001, *Tupayachi 5155* (CUZ!); 4350 m, 01 July 1915, *Bingham 2068* (US!). **Junín**: Concepcion, Dist. de Comas, localidad de Pomamanta, 11°44'24"S, 075°09'39"W, 4400 m, 23 August 2017, *Quispe 76* (CUZ!, USM!, Z!). Satipo, Pampa Hermosa, Toldopampa, 11°29'34"S, 074°56'37"W, 4160 m, 02 August 2016, *Boza 3169*; *3170*; *3171*; *3172*; *3173*; *3174*; *3175*; *3176*; *3177*; *3178* (USM!, Z!); Pampa Hermosa. Toldopampa, 13°12'15"S, 075°20'22"W, 4131 m, 02 August 2016, *Boza 3179* (USM!, Z!); *3180* (USM!, Z!); Dist. Pampa Hermosa, Comunidad Campesina Santa Rosa de Toldopampa, 11°29'34"S, 074°56'38"W, 4221 m, 21 April 2015, *Valenzuela 28873* (HOXA, MO!, USM!). **Puno**: Limbani, Huancasayani on road to Limbani just east of Abra Aricoma, 14°13'S, 069°42'W, 3750 m, 28 March 1987, *Boertmann 130* (AAU!); *512* (AAU!). **San Martín**: Mariscal Caceres, Dist. de Huicungo, Callejón de Corneadas, 07°57'46"S, 077°23'23"W, 3925 m, 11 June 2001, *León 5153* (USM!); Dist. Huicungo, en pirca, debajo del camino de abra Ventanas y Laguna Colorada, 08°00'53"S, 077°23'30"W, 3924 m, 20 June 2010, *León 5539* (USM!). San Martín, Dist. de Huicungo, cerca a Laguna Colorada, camino al abra Ventanas, 3900 m, 18 June 2001, *León 5260* (USM!).

### ﻿ Polylepis section Reticulatae

Taxon classificationPlantaeRosalesRosaceae

 T. Boza & M.Kessler sect. nov.

8A9BC764-5C68-5D65-8359-361E1F779966

urn:lsid:ipni.org:names:77301638-1

#### Diagnosis.

Trees or shrubs, lower leaflet surfaces tomentose; apices emarginate; fruits with variable numbers and placements of flattened, almost cylindrical or long spines, densely lanose, tomentose or villous.

#### Type.

*Polylepisreticulata* Hieron.

#### Notes.

The sectional epithet *Reticulatae* is a plural adjective agreeing in gender with *Polylepis*. Section Reticulatae, first informally recognized by Simpson (1979) and later recovered as monophyletic in the phylogenetic analysis of Schmidt-Lebuhn et al. (2006a), contains species with relatively few lateral leaflets pairs, rugose or shiny upper leaflet surfaces, emarginate leaflet apices and felt-like covering on the lower leaflet surfaces. All species placed in this section have the lower leaflet surfaces with an evenly distributed dense layer of short, white to yellowish pannose hairs, admixed with short to moderately long tomentose hairs. As in section Sericeae, species in this section have fruits with straight or recurved spines. *Polylepismicrophylla*, *P.occidentalis* and *P.quadrijuga* have many lateral leaflet pairs (3–6), but all three have rugose or shiny upper leaflet surfaces and emarginate leaflet apices. The most distinct species of this section is *P.hieronymi*, which has sparsely tomentose upper leaflet surfaces and almost cylindrical fruits with long spines. This is also the geographically most remote species, being separated by over 1500 km from the other members of the section. *Polylepismicrophylla* also has atypical, turbinate fruits. *Polylepisquadrijuga* is similar in some ways to *P.frontinensis* and *P.lanuginosa* of section Sericeae, but these species do not have the dense layer of short pannose hairs admixed with tomentose hairs on the lower leaflet surfaces. Table 5 provides an overview of the arrangement of the taxa by different authors.

**Table 5.** Alignment of the taxa of the Polylepissect.Reticulatae according to Bitter (1911), Simpson (1979), Segovia et al. (2018) and the present study.

Bitter (1911)	Simpson (1979)	Segovia et al. (2018)	This study
*P.brachyphylla*	*P.reticulata*	*P.reticulata*	*P.reticulata*
*P.nitida*			
*P.reticulata*			
			*P.occidentalis*
*P.hieronymi*	*P.hieronymi*	*P.hieronymi*	*P.hieronymi*
*P.microphylla*	*P.weberbaueri*	*P.microphylla*	*P.microphylla*
*P.weberbaueri*		*P.weberbaueri*	*P.simpsoniae*
			*P.weberbaueri*
*P.quadrijuga*	*P.quadrijuga*	*P.quadrijuga*	*P.quadrijuga*

##### Climatic niches in Polylepissect.Reticulatae

**Figure 46.**
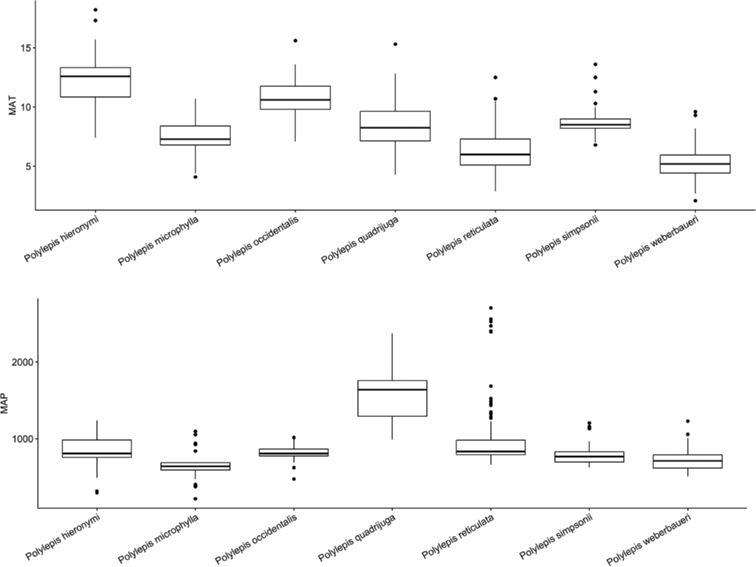
Box plots showing the climatic niches of the species of section Reticulatae in relation to MAT (**A**) and MAP (**B**). See Fig. 12 for details on data presentation.

Many species of this section differ notably in the Mean Annual Temperature (MAT) of their climatic niches, with only *P.quadrijuga* and *P.simpsoniae* not being statistically different (Fig. 46). *Polylepishieronymi* grows under the highest temperatures (mean of 12.3 °C MAT), followed by *P.occidentalis* (10.7 °C), whereas *P.reticulata* (6.4 °C) and *P.weberbaueri* (5.2 °C) grow under the coldest conditions. These differences of up to 7 °C correspond to elevational differences of well over 1000 m. Regarding Mean Annual Precipitation (MAP), most of the species in this group grow under relatively arid conditions with similar averages of precipitation (807–835 mm MAP). Species growing in even drier areas are *P.microphylla* (675 mm MAP) and *P.weberbaueri* (731 mm), whereas those growing in most humid conditions are *P.reticulata* (1021 mm) and *P.quadrijuga* (1638 mm). Most species are allopatric, but in Ecuador, *P.reticulata* and *P.simpsoniae* co-occur close to each other and have distinct climatic niches, with *P.reticulata* growing under colder and more humid and *P.simpsoniae* under warmer and drier conditions.

### ﻿16. Polylepis hieronymi

Taxon classificationPlantaeRosalesRosaceae

Pilger, Bot. Jahrb. Syst. 37: 534. 1906.

BA7A0573-C62B-52DD-A1CF-0E972F309CC6

Figs 47 , 48

Polylepis hypoleuca (Weddell) Bitter, Bot. Jahrb. Syst. 45: 607. 1911.
Polylepis racemosa β hypoleuca Weddell, Chlor. Andina 2:238. 1857 [1861]. Basionym. Type. Bolivia. Tarija: between Tarija and San Luis, July-August 1846, *Weddell 4607* (lectotype, designated by Simpson 1979, pg. 23: P).
Polylepis racemosa var. albotomentella Kuntze, Revis. Gen. Pl. 3: 77. 1898. Type. Argentina. Córdoba: Sierra de Córdoba, Los Gigantes, *Kurtz 6926* (holotype: NY!).
Polylepis australis var. bijuga Bitter (1911: 624) Nom. illeg.
Polylepis hieronymi var. d*olicholopha*Bitter (1911:609). Nom. illeg.
Polylepis hieronymi var. saltensis Bitter, Bot. Jahrb. Syst. 45: 609. 1911. Type. Argentina. Salta: near Pampa Granda, pass “El Alizar”, 2400–2600 m, 1900, *Nelson 12584* (holotype: S).

#### Type.

Bolivia. Tarija: Salinas, Cuesta de Polla, Valle del Tambo, June 1873, *Lorentz & Hieronymus 938a* (holotype: B destroyed; isotypes: G!, GOET!, NY!).

**Figure 47.**
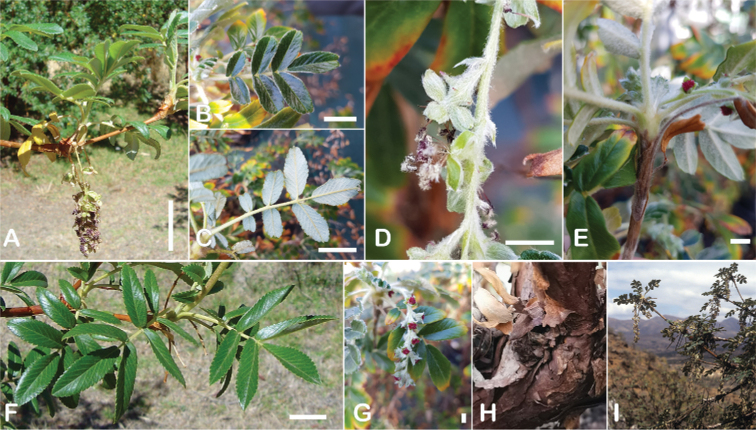
*Polylepishieronymi* Pilger **A** flowering branch **B** upper leaf surface **C** lower leaf surface **D** flower and young fruit **E** stipule sheaths **F** leaves **G** flowering branch **H** bark **I** flowering branch. Scale bars: 3 cm (**A**); 1 cm (**B, C, F**); 5 mm (**D, E, G**). Photographs by M. Kessler.

#### Description.

***Trees*** 3–8(–25) m tall. ***Leaves*** slightly congested at the branch tips, imparipinnate with 3–4 pairs of lateral leaflets, obtrullate in outline, (3.3–)3.6–5.2 × 2.1–3.1 cm; rachises densely tomentose, points of leaflet attachment with a tuft of long, lanate hairs; stipular sheaths apically truncate with spurs, densely sericeous on the outer surfaces; leaflets narrowly obovate in outline, second pair from the terminal leaflet the largest, one of this pair (1.2–)1.5–2.1 × 0.6–1.0 cm; margin crenate with 5–7 teeth, apically emarginate, basally unequally cordate; upper leaflet surfaces sparsely tomentose; lower leaflet surfaces densely tomentose with whitish hairs 0.8–1.1 mm long. ***Inflorescences*** pendant, (4.5–)5.6–7.5(–8.1) cm long, bearing 13–25 flowers; floral bracts 3.2–6.3 mm long, narrowly triangular, densely lanate on the outer surface; rachises villous. ***Flowers*** 5.4–6.5 mm diam.; sepals 4, ovate, green, densely sericeous outside; stamens 9–19, anthers orbicular, with a dense tuft of straight white hairs on the upper half; styles fimbriate, 2.3–3.9 mm long. ***Fruits*** almost cylindrical, with variable numbers and placement of long spines, densely lanose; (4.1–)6.0–6.4(–8.3) × 3.5–7.0 mm including spines. ***Diploid***.

**Figure 48.**
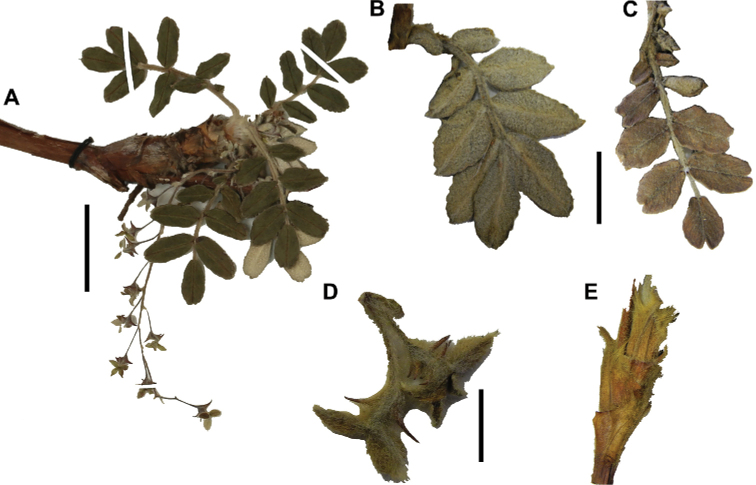
*Polylepishieronymi* Pilger **A** flowering branch **B** lower leaf surface **C** upper leaf surface **D** fruit **E** stipular sheaths (**A***Kessler 3114***B***Kessler 3306***C***Kessler 3308***D***Kessler 3319***E***Kessler 3318*). Scale bars: 2 cm (**A–C**); 5 mm (**D**). Photographs by T. E. Boza E.

#### Distribution, habitat and ecology.

*Polylepishieronymi* occurs in Boliviano-Tucumanic forests at 1500–3450 m elevation (Fig. 61). It grows in relatively dry areas as a pioneer species that often colonizes landslides and roadsides in mixed forests with *Podocarpusparlatorei* and *Alnusacuminata* (Kessler 1995b; Gareca et al. 2010). Trees of *P.hieronymi* often grow as isolated individuals or small stands mixed in forest dominated by typical high Yungas species. It also occurs on very steep slopes with shallow soils that do not permit persistence of taller trees (Bellis et al. 2014). Thus, the ecology of *P.hieronymi* is different from that of almost all other *Polylepis* species, which usually dominate the canopy of the forest they belong to (Renison et al. 2013). *Polylepis* specialist birds are absent in forests of *P.hieronymi*, possibly because they do not form large, mature forests (Bellis et al. 2014). *Polylepishieronymi* occasionally co-occurs with *P.australis* in Salta and Jujuy (Argentina) and with *P.crista-galli* in Chuquisaca (Bolivia), but hybrids have not been found to date.

#### Conservation status.

Based on 28 collecting localities, the estimated EOO is 150,691 km^2^ and the AOO is 148 km^2^. It was categorized as VU (B1+2c) in the World List of Threatened Trees (Oldfield et al. 1998). In Bolivia, it occurs in Cordillera de Sama Biological Reserve in Tarija. We assess *P.hieronymi* as Vulnerable (B1a+B2ac).

#### Notes.

Sterile individuals of *P.hieronymi* can be confused with sterile plants of *P.besseri*, also because both species broadly overlap in distribution. Both species have quite similar leaflet shapes and texture, numbers of lateral leaflet pairs and densely tomentose lower leaflet surfaces. If no fruits are available (spiny in *P.hieronymi*, with broad ridges in *P.besseri*), they are best distinguished by the sericeous hairs on the stipular sheaths in *P.hieronymi* and tomentose hairs in *P.besseri*. *Polylepishieronymi* also somewhat resembles *P.neglecta* in having 3–4 lateral leaflet pairs and relatively long inflorescences with many flowers. However, it has narrowly obovate leaflets with crenate margin and tomentose hairs 0.8–1.1 mm long, styles 2.3–3.9 mm long and spiny fruits, whereas *P.neglecta* has elliptic leaflets with serrate margins and glabrous to puberulous lower surfaces, shorter styles 1.5–2.2 mm long and winged fruits.

#### Specimens examined.

**Argentina. Jujuy**: Capital, Tiraxi, Alto Salviar, ladera E, 08 November 1989, *Tupayachi s.n* (MO!). **Salta**: Guachipas, Cuesta del La jar, 1600–1700 m, 07 February 1983, *Novara 3142* (MO!).

**Bolivia. Chuquisaca**: Azurduy, Saliendo de Azurduy hacia el río Pilcomayo, 20°12'52"S, 064°26'37"W, 3114 m, 15 October 2007, *Cervantes 187* (HSB, MO!); Belisario Boeto, Trayecto Villa Serrano hacia la comunidad de Tampa Mayu y Nuevo Mundo, 19°00'04"S, 064°18'55"W, 2297 m, 12 December 2007, *Cervantes 157 B* (HSB, MO!); 1 km S Nuevo Mundo on road to Padilla, 19°28'S, 064°10'W, 2200 m, 07 October 1991, *Kessler 3306* (GOET!, LPB); *3307* (AAU!, GOET!, MO!); *3308* (AAU!, GOET!, MO!); *3309* (GOET!); 8 km SW Nuevo Mundo on road to Padilla, 19°25'S, 064°11'W, 2500 m, 07 October 1991, *Kessler 3317* (GOET!); *3318* (GOET!); *3321* (GOET!); próximo a Lagunillas, 2240 m, 24 January 1988, *Murguía 128* (LPB); Hernando Siles, subiendo de la Hacienda Guzman para el Abra, 20°17'16"S, 064°02'56"W, 1993 m, 24 December 2005, *Peñaranda 22* (HSB, MO!, QCA!); Oropeza, Municipio de Yotala, Canton Huayllas. Comunidad Pitatorillas, 19°09'06"S, 065°20'48"W, 3374 m, 23 September 2007, *Jiménez 376* (HSB, MO!, QCA!). Sud Cinti, Cerro Cobre Khasa, between Culpina and El Palmar, 20°48'S, 064°34'W, 3100 m, 21 September 1991, *Fjeldså s.n* (GOET!). Tomina, 25 km hacia Montegudo, 19°03'S, 064°16'W, 2400 m, 01 October 1983, *Beck 9345* (BOLV, GOET!, LPB, MO!, NY); ca. 20 km SE Padilla on road to Monteagudo, 19°03'S, 064°16'W, 2450 m, 07 October 1991, *Kessler 3319* (AAU!, GOET!); Trayecto Lima Bamba – EL Villar, 19°33'01"S, 064°19'55"W, 2553 m, 13 October 2007, *Portal 138* (HSB, MO!). **Santa Cruz**: Mairana, within the Flora de la Region del Parque Nacional Amboro, but above the 700 m contour, 18°06'30"S, 063°57'00"W, 2000–2100 m, *Nee 43429* (MO!, NY). Manuel Maria Caballero, Parque Nacional Amboró. San Juan del Potrero; entre Yunguillas y cabeceras del río Zapallar, 17°53'S, 064°25'W, 2300–2400 m, 12–13 May 1992, *Vargas 1350* (MO!, NY, USZ). Vallegrande, between “Mataralcito” and “El Palmar” on road from Valle Grande to Tierras Nuevas, 17 km by air ESE of Valle Grande, 18°32'00"S, 065°57'00"W, 2150 m, 29 December 1988, *Nee 37403* (NY); camino de Tierras Nuevas a Vallegrande, 18°30'27"S, 063°55'04"W, 2249 m, 31 July 2011, *Parada-Gutierrez 3580* (MO!, USZ); camino hacia Khasa Monte, sobre la cima de la serrania, 18°38'09"S, 064°02'01"W, 2550 m, 04 August 2011, *Parada-Gutierrez 3671* (MO!, USZ); camino del Cruce hacia Alto Seco, 18°44'45"S, 064°06'53"W, 2717 m, 08 July 2011, *Parada-Gutierrez 3746* (MO!, USZ); Senegilla a 17 km de Vallegrande, 18°40'03"S, 064°01'55"W, 2400 m, 20 August 2012, *Parada-Gutierrez 4828* (MO!, USZ); a 4 km al norte de Postrervalle sobre el camino a Mairana, 2000 m, 13 November 1999, *Saldias 6192* (USZ). Vallegrande, 2363 m, 24 August 2008, *Arroyo 4007* (QCA!); Meson at Samaipata, 2200 m, 01 March 1911, *Herzog 1786a* (GOET!). **Tarija**: Arce, 43 km hacia Padcaya, Huancanqui, 2500–2600 m, 20 November 1986, *Beck 14080* (GOET!); cerca de Camacho, 2600 m, 17 December 1987, *Beck 16067* (GOET!); bajando del Abra del Cerro Cabildo hacia el S via estancia Cabildo, 2350 m, 29 January 1988, *Beck 16240* (GOET!); ca. 5 km W Padcaya, 21°54'S, 064°46'W, 2200 m, 18 September 1991, *Kessler 3114* (AAU!, GOET!); *3646* (GOET!); detras de Padcaya, 2450 m, 23 January 1988, *Liberman 1637* (GOET!); Municipio Padcaya, Cantón Emborozú, Reserva Natural Alarachi, recorrido a cima más alta de la Zona Alarachi, próximo al Cerro Yauparuna, 22°10'44"S, 064°36'33"W, 2260–2380 m, 16 September 2004, *Serrano 4828* (MO!); 39.9 km S of jct. of road to Entre Rios, on road to Padcaya, 21°54'S, 064°41'W, 2100–2200 m, 29 April 1983, *Solomon 10218* (LPB, MO!, NY). O’Connor, ca. 5 Km W Padcaya, 21°54'S, 064°46'W, 2200 m, 18 September 1991, *Kessler 3113* (AAU!, GOET!, MO!); *3115* (GOET!, MO!); ca. 70 km on road from Tarija to Entre Rios, 21°26'S, 064°19'W, 2200 m, 20 September 1991, *Kessler 3123*; *3124* (AAU!, GOET!); *3125* (GOET!, MO!); *3660* (AAU!, GOET!, MO!); 21.1 km on road to entre Rios, 21°27'S, 064°20'W, 1900 m, 01 October 1983, *Solomon 10918* (LPB, MO!). Valle del Tambo bei Tarija, 10 June 1973, *Hieronymus 938* (GOET!); Cult. at Jardin Botanico La Paz 2000 from seeds, s.d., *Kessler 12625* (GOET!). s.d., *Cárdenas 3906* (US!); *3907* (US!); Salinas, Cuestas de Polla, in valle Tambo, June 1873, *Hieronymus 938a* (B, F!); s.d., *Hieronymus 938a* (B, MO!).

### ﻿17. Polylepis microphylla

Taxon classificationPlantaeRosalesRosaceae

(Wedd.) Bitter, Bot. Jahrb. Syst. 45: 611. 1911.

3B228EC0-2986-5891-A60E-A787B5D6F50A

Figs 49 , 50

Polylepis microphylla var. polyarthotricha Bitter, Bot. Jahrb. Syst. 45: 612. 1911. Type. *Goudot 1* (holotype: W).

#### Basionym.

Polylepislanuginosavar.microphylla Weddell, Chlor. Andina 2:238.1861.

#### Type.

Ecuador. Chimborazo: *Humboldt & Bonpland 3141* (holotype: P!; isotypes: F!, GOET! US!).

**Figure 49.**
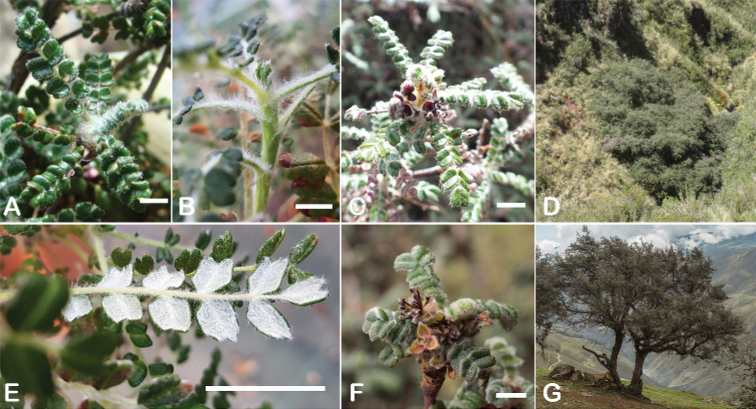
*Polylepismicrophylla* (Wedd.) Bitter **A** leaves **B** stipule sheaths **C** flowering branch **D** habit **E** lower leaf surface **F** flowering branch **G** habit. Scale bars: 1 cm (**A, E**); 5 mm (**C, F**); 1 mm (**B**). Photographs **A, C** T.E. Boza E. **D, F** E.G. Urquiaga F. **B, E** M. Kessler **G** E. Bastidas.

#### Description.

***Shrubs and trees*** 1.5–8 m tall. ***Leaves*** strongly congested at the branch tips, imparipinnate with 3–6 pairs of lateral leaflets, obtrullate in outline, (1.3–)2.0–3.5 × (0.6–)1.2–1.5 cm; rachises densely tomentose often intermixed with twisted dark red hairs; stipular sheaths apically acute with spurs, densely tomentose on the outer surfaces; leaflets broadly elliptic in outline, second pair from the terminal leaflet the largest, one of this pair 0.3–0.7 × 0.2–0.5 cm; margin entire, apically deeply emarginate, basally unequally cordate; upper leaflet surfaces glabrous or sparsely tomentose mainly in the mid-vein depression; lower leaflet surfaces densely tomentose with whitish hairs 0.8–1.0 mm long. ***Inflorescences*** branched at the base or simple, pendant, 3.8–5.3 cm long, bearing 1–3 flowers; floral bracts 2.2–2.5 mm long, narrowly triangular, densely tomentose on the outer surface; rachises tomentose. ***Flowers*** 4.0–6.4 mm diam.; sepals 4, ovate, green, glabrous outside; stamens 9–11, anthers orbicular, with a dense tuft of straight white hairs on the upper half; styles fimbriate, 2.6–3.5 mm long. ***Fruits*** turbinate, with variable numbers of long spines, densely tomentose; 2.7–4.3 × 1.3–2.1 mm including spines. ***Diploid*** and ***tetraploid***.

**Figure 50.**
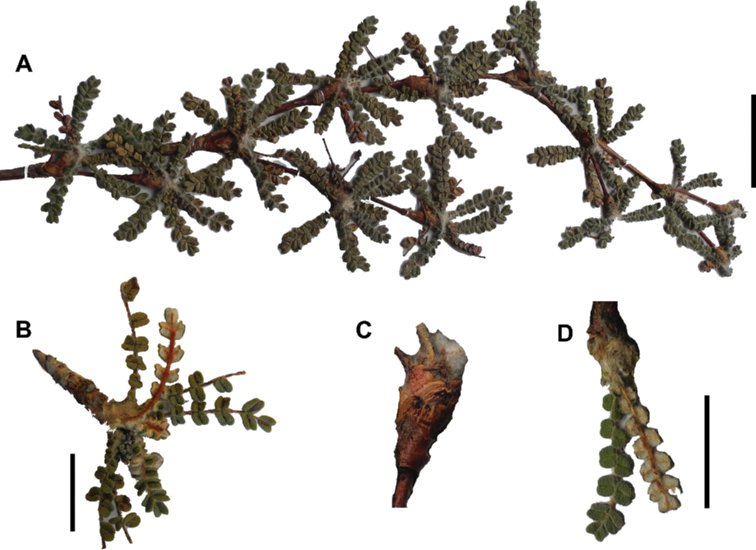
*Polylepismicrophylla* (Wedd.) Bitter **A** branch **B** leaves **C** stipular sheaths **D** upper and lower leaves surface (**A***Toivonen 98***B***Boza 3001***C***Toivonen 19***D***Irazabal 205*). Scale bars: 2 cm (**A–C**); 5 mm (**D**). Photographs by T. E. Boza E.

#### Distribution, habitat and ecology.

*Polylepismicrophylla* occurs in small, isolated populations in the central Ecuadorian Andes on the slopes of Volcán Chimborazo, in north-western Peru in the high Andes of Cajamarca, the Cordillera Blanca and the adjacent Cordillera Huayhuash at the boundaries of Ancash and Lima and in Arequipa and Cusco (Fig. 61). It grows mainly in arid zones at 3150–4550 m elevation where it usually forms small groves, but many populations in areas strongly affected by human activities contain mostly small shrubs. However, in the Cordillera Huayhuash, extensive stands with trees about 3–5 m tall can be found. The populations in Cusco are close to Incan ruins and might represent historical transplantations (Kessler and Schmidt-Lebuhn 2006).

#### Conservation status.

The EOO is estimated as 407,902 km^2^ and AOO as 100 km^2^. The species is known from 18 locations. The two forests in Ecuador form a single genetic population (Bastidas-León et al. 2021). It is protected within Sangay National Park in Ecuador and Huascarán National Park and Cordillera Huayhuash Reserved Zone (within Huayllapa Private Conservation Area) in Peru. The species was categorized as VU (B1+2c) in the World List of Threatened Trees (Oldfield et al. 1998). Lately, based on its restricted distribution in Chimborazo, *P.microphylla* was categorized as CR (B2ab(iii)) in Ecuador (León-Yánez et al. 2011; Tejedor et al. 2014, 2015). In Peru, where it is more widespread, *P.microphylla* was categorized as EN (SERFOR 2006). At many of its locations, the species grows in habitats that are strongly affected by human activities including grazing and burning. We assess *P.microphylla* as Endangered (B1a+B2ab).

#### Notes.

*Polylepismicrophylla* was included in *P.weberbaueri* by Simpson (1979), based on material from Cañar (Ecuador) that contains lower mature leaves like those of *P.weberbaueri* (here assigned to *P.simpsoniae*) and upper young leaves similar to those of the specimens placed under *P.microphylla* by Bitter (1911). Later, *P.microphylla* was considered as a distinct species by Romoleroux (1996), based on newly collected material from Chimborazo that does not have such differences between mature and younger leaves. *Polylepismicrophylla* differs from *P.simpsoniae* by smaller leaflet size (0.3–0.7 × 0.2–0.5 cm versus 0.9–1.6 × 0.4–1.1 cm), longer leaflet hairs (0.8–1.0 mm versus 0.5–0.7 mm) and inflorescences with lower number of flowers (1–3 versus 3–5).

#### Specimens examined.

**Ecuador. Chimborazo**: Alausí, camino Totoras-Charicando, 02°11'30"S, 078°42'18"W, 3500 m, 08 July 2004, *Caranqui 1205B* (CHEP); Shumit, Pucara Achupallas, 3986 m, 04 September 2009, *Cárate 1200* (QCA!); carretera Alausí-Achupallas-Osogoche, 3300–3400 m, 10 August 1987, *Romoleroux 372* (AAU!, NY, QCA!); Vía Achupallas-Osogoche, km 14.5, localidad Zula, 3600 m, 24 March 2001, *Romoleroux 3995* (QCA!); Alausí-Achupallas, Páramo Parada 1, 3576 m, 17 March 2007, *Romoleroux 4414* (QCA!); Alausí-Achullapas, 3655 m, 17 March 2007, *Romoleroux 4435* (QCA!); Vía Totoras Achupallas, Lagunas de Osogoche, 3626 m, 08 November 2008, *Romoleroux 5325* (QCA!); Vía desvío Osogoche-Achupallas, 3589 m, 28 December 2011, *Romoleroux 5704* (QCA!); Vía Achupallas-lagunas de Osogoche, km 11.5, 3000 m, 26 April 1988, *Romoleroux 576* (AAU!, MO!, QCA!); Vía Achupallas-lagunas de Osogoche km 11.5, 3650 m, 26 April 1988, *Romoleroux 577* (AAU!, NY, QCA!); 3650 m, s.d., *Romoleroux 578* (AAU!, NY, QCA!); Vía Achuapallas-Lagunas de Osogoche, km. 15, 3650 m, 26 April 1988, *Romoleroux 579* (AAU!, MO!, NY, QCA!); 3650 m, *Romoleroux 580* (AAU!, NY, QCA!); Vía Achupallas-Lagunas de Osogoche, km 15, 3650 m, 26 April 1988, *Romoleroux 581* (AAU!, NY, QCA!); Vía Osogoche-Achupallas, 3610 m, 30 September 2016, *Romoleroux 6126* (QCA!); Vía Osogoche-Achupallas, 3610 m, 30 September 2016, *Romoleroux 6127* (QCA!); Vía Osogoche-Achupallas, 02°15'53"S, 078°42'09"W, 3610 m, 09 March 2017, *Romoleroux 6148* (QCA!); Alausí-Achupallas, 3655 m, 17 March 2007, *Romoleroux GPI4435* (QCA!), Quitesian Andes, s.d., *Cothouy s.n* (NY); 1857–1864, *Spruce s.n* (MO!).

**Peru. Ancash**: Huaylas, Caraz, Laguna Parón, flanco norte, 4170 m, 27 April 2013, *Baldeón 7816* (USM!); Caraz, Laguna Paron, *Irazábal 199* (CUZ!); Yungay, Parque Nacional de Huascaran, laguna Paron, 08°59'55"S, 077°41'26"W, 4200 m, 11 February 1997, *Tupayachi 3271* (CUZ!). **Cajamarca**: San Miguel, San Miguel de Pallaques, road Agua blanca to Oyotum, Ponga la Mesa, 3500–3600 m, 14 October 2000, *Weigend 2000/748* (F!, USM!). **Cusco**: Acomayo, Queuñayocpampa, 14°04'01"S, 071°35'08"W, 3940 m, 01 April 2003, *Arce s.n* (USM!); Rondocan, localidad Parara, 13°47'10"S, 071°45'08"W, 4085 m, *Pfuro AS-133* (Z!). Calca, Pisac, 13°25'S, 071°51'W, 3400 m, 10 February 2003, *Lægaard 22250* (AAU!). Cusco, Huacoto, 13°30'54"S, 071°51'19"W, 3960 m, 01 May 2003, *Arce s.n* (USM!); Chacan, 13°29'04"S, 071°59'26"W, 3805 m, 17 September 2014, *Boza 3001*; *3002* (USM!, Z!); Cuzco, Chacan camino al grupo arqueológico, borde de la microcuenca Chacan, 3600 m, 03 October 2000, *Galiano 3999* (QCA!); Huacoto, 3991 m, *Irazábal 204* (CUZ!); Chacan S of Cusco, 13°29'S, 072°00'W, 3900 m, 10 February 2003, *Lægaard 22350* (AAU!, US!); San Jerónimo, localidad de Huacoto, 13°31'06"S, 071°51'32"W, 3940 m, 25 May 2006, *Toivonen 19* (CUZ!); *20* (CUZ!); *21* (CUZ!); Chacan, 14 June 2006, *Toivonen 98* (CUZ!); Chacan, 14 June 2006, *Toivonen 99* (CUZ!); Ruinas de Pisac, 3200 m, 01 May 2002, *Toivonen s.n* (CUZ!); Chacan, 3600 m, 28 April 1993, *Tupayachi 2280* (CUZ!). Quispicanchis, Dist. Huaro, Urpay, 13°41'01"S, 071°38'22"W, 3200 m, 01 November 2002, *Galiano 4512* (AMAZ, CUZ!, HUT, MO!, MOL, USM!). Urubamba, Cusco. Prov. Urubamba, Yucay, Hatum Wayko, 3500 m, 09 September 2001, *Herrera 4175* (QCA!); Yucay, 3833 m, *Irazábal 207* (CUZ!); Yucay, cerro Turukuntur, 3750 m, 13 January 1991, *Tupayachi 1460* (CUZ!). **Lima**: Cajatambo, Huayllapa. Dist. de Copa, cerro empinado a 115 km del pueblo, 3360 m, 13 May 2001, *Callupe 1* (USM!).

### ﻿18. Polylepis occidentalis

Taxon classificationPlantaeRosalesRosaceae

T.Boza & M.Kessler sp. nov.

B5D9DE97-3676-5D90-9F6E-E89252E7A25E

urn:lsid:ipni.org:names:77301640-1

Figs 51 , 52

#### Diagnosis.

Resembles the Ecuadorian species, *P.reticulata* Hieron. by having 3–5 lateral leaflet pairs and similar type and density of hairs, but differs by its shorter hairs (0.3–0.6 mm vs. 0.6–1.5 mm), shorter inflorescences (2.4–6.7 cm vs. 2.3–13.8 cm) and shorter styles (1.5–2.0 mm vs. 2.6–3.9 mm).

**Figure 51.**
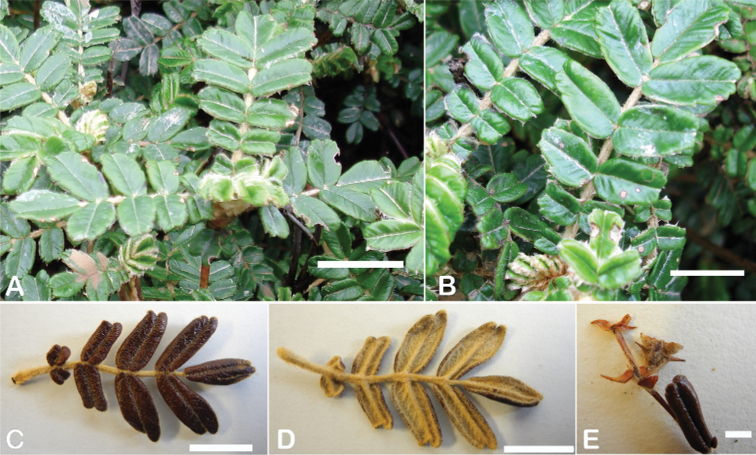
*Polylepisoccidentalis* T.Boza & M.Kessler **A** leaves **B** leaf **C** upper leaf surface **D** lower leaf surface **E** fruit. Scale bars: 2 cm (**A**); 1 cm (**B–D**); 3 mm (**E**). Photographs **A, B** M. Richter **C–E** T.E. Boza E.

#### Type.

Peru: Piura: Huancabamba, Talanco, 2900 m, 5 Nov 1976, *A. Sagastegui A.8635* (holotype: MO!; isotype: UMO!).

**Figure 52.**
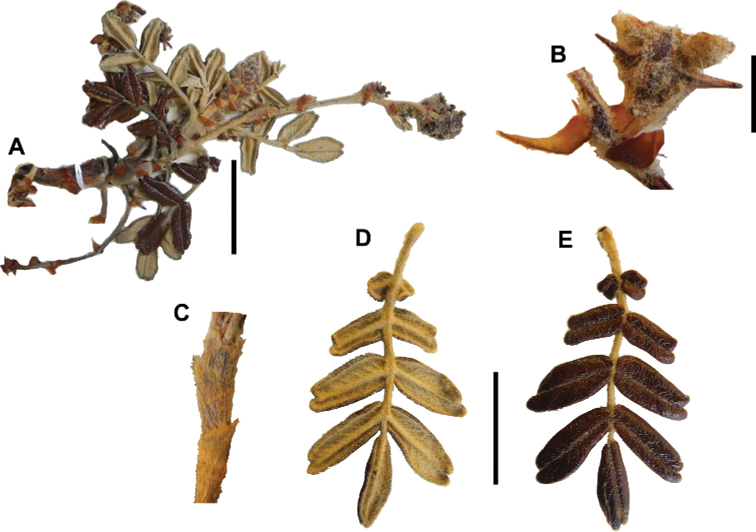
*Polylepisoccidentalis* T.Boza & M. Kessler **A** flowering branch **B** fruit **C** stipular sheaths **D** lower leaf surface **E** upper leaf surface (**A***Dillon 4145***B, D, E***Diaz 4012***C***Beltran 6933*). Scale bars: 4 cm (**A**); 4 mm (**B**); 2 cm (**D, E**). Photographs by T. E. Boza E.

#### Description.

***Trees*** 3–15 m tall. ***Leaves*** slightly congested at the branch tips, imparipinnate with 3–5 pairs of lateral leaflets, obtrullate in outline, 3.1–4.7 × 1.8–2.9 cm; rachises densely tomentose, points of leaflet attachment with a tuft of long, lanate hairs; stipular sheaths apically acute with spurs, densely lanate on the outer surfaces; leaflets elliptic in outline, second pair from the terminal leaflet the largest, one of this pair 1.1–1.9 × 0.5–0.8 cm; margin entire or slightly crenate at the apex with 3–6 teeth, apically emarginate, basally unequally cordate; upper leaflet surfaces glabrous; lower leaflet surfaces densely tomentose with whitish hairs 0.3–0.6 mm long. ***Inflorescences*** pendant, 2.4–6.7 cm long, bearing 4–12 flowers; floral bracts 6.8–7.6 mm long, narrowly triangular, densely lanate on the outer surface; rachises tomentose. ***Flowers*** 6.8–7.6 mm diam.; sepals 4, ovate, green, densely tomentose outside; stamens 9–15, anthers orbicular, with a dense tuft of straight white hairs on the upper half; styles fimbriate, 1.5–2.0 mm long. ***Fruits*** turbinate, with variable numbers and placement of flattened spines, densely villous; 3.8–4.1 × 5.8–7.1 mm including spines. ***Diploid***.

#### Distribution, habitat and ecology.

*Polylepisoccidentalis* is distributed in the high mountains of western Peru from Huancabamba (Piura) to Pataz (La Libertad) (Fig. 61). The species occurs in relatively dry forest at 2200–3990 m elevation.

#### Etymology.

The species epithet “occidentalis” (Latin: western) refers to the distribution range occupying the western mountains in Peru.

#### Conservation status.

The EOO for *Polylepisoccidentalis* is estimated as 12,906 km^2^ and the AOO at 52 km^2^. It is known from 11 locations. It has been found in monospecific forests at the southern margins of the Huancabamba Andean Depression, which forms an important dispersal corridor for Andean tree species (Richter et al. 2009; Peters et al. 2014). However, collections from Cajamarca, La Libertad and Lambayeque are mostly old and the collection areas have undergone heavy deforestation. Based on the reduction of its restricted distribution and its habitat degradation, we assess *P.occidentalis* as Endangered (A1, B1a+B2a, C1).

#### Notes.

The populations of PolylepissectionReticulatae from western Peru have been previously identified either as *P.reticulata* (Lassermann 2009) or *P.weberbaueri* (Simpson 1979; Lassermann 2009; Mendoza and Cano 2012; Peters et al. 2014) (Fig. 34). Certainly, *P.occidentalis* resembles *P.reticulata* in having 3–5 lateral leaflet pairs and same type and density of hairs. However, it has shorter lower leaflet surface hairs (0.3–0.6 mm long), shorter inflorescences (2.4–6.7 cm long) and shorter styles (1.5–2.0 mm long), whereas *P.reticulata* has lower surface hairs 0.6–1.5 mm long, inflorescences (2.1–)4.3–13.8 cm long and styles 2.6–3.9 mm long. Additionally, *P.occidentalis* occurs in western Peru, whereas *P.reticulata* is distributed in Ecuador. *Polylepisoccidentalis* is morphologically also similar to *P.weberbaueri*, with which it shares similar leaflet size and lower leaflet surface hair type and density. The most obvious differences between *P.occidentalis* and *P.weberbaueri* are leaflet pair number, inflorescence length, style length and number of stamens, with *P.occidentalis* having 3–5 leaflet pairs, inflorescences 2.4–6.7 cm long, styles 1.5–2.0 mm long and 9–15 stamens, whereas *P.weberbaueri* has 2–3 leaflet pairs, inflorescences 8.2–9.7 cm long, styles 2.7–3.2 mm long and 19–21 stamens.

Simpson (1979) considered *P.reticulata* and *P.weberbaueri* to occur in both Ecuador and Peru, with the populations of each species separated by the low elevation Huancabamba depression. Interestingly, however, defined in this way in Ecuador, *P. “reticulata*” occurs at higher elevations than *P. “weberbaueri*”, whereas in Peru, the reverse is the case. In addition to the morphological traits outlined above, we consider this intriguing distributional pattern to support treatment as four species, with the original *P.reticulata* restricted to Ecuador and *P.weberbaueri* to Peru and the respective other populations here described as *P.occidentalis* (Peruvian *P. “reticulata*”) and *P.simpsoniae* (Ecuadorean *P. “weberbaueri*”).

#### Specimens examined.

Peru. Cajamarca: Cajamarca, Dist. La Encañada, San Pedro de Lipiac, 07°03'38"S, 078°20'00"W, 3738 m, 07 September 2010, *Beltrán 6933* (USM!). Chota, Llama; caserío Callampampa; afluente de Huarimarca, 2800–3000 m, 19 January 1990, *Díaz 4012* (MO!, USM!); Laguna Yahuarcocha (arriba de Incahuasi), 07°34'45"S, 077°58'29"W, 3600 m, 14 September 1985, *Sagástegui 12896* (GOET!, IBE, MO!); Tucupampa (Llama-Huambos), 2500 m, 17 March 1997, *Sagástegui 15950* (F!, MO!); Miracosta, entre Miracosta y Pampa del Lirio, 06°23'S, 079°15'W, 3250 m, 11 November 2000, *Sánchez 10285* (MO!); Huambos, 2000 m, 11 September 1956, *Soukup 4457* (F!). Cutervo, carretera entre Llama y Huambos, Tunaspampa, 06°31'52"S, 079°05'26"W, 2600–2900 m, 21 April 1988, *Díaz 2879* (F!, MO!). Huambos, 65 km E of bridge over Río Maichil, 25 km W of Huambos, 06°25'S, 079°00'W, 2700 m, 09 February 1988, *Gentry 61439* (MO!, USM!). Llama, El Pargo, 4.2 km E of Llama, ca. 1.4 km SE of Tunas Pampas, 06°30'S, 079°31'W, 3000 m, 08 September 1991, *Gentry 74575* (MO!, USM!). **La Libertad**: Pataz, Chirimachay, 07°00'S, 077°00'W, 3450 m, 24 February 1986, *Young 2966* (MO!). **Lambayeque**: Ferrenafe, Ca. 7 km NW of Incahuasi; near Cerro Punamachay on trail to Laguna Hualtaco, 3300–3550 m, 16 November 1984, *Dillon 4145* (MO!, USM!). **Piura**: Huancabamba, 38 km above Canchaque, just below summit, 05°18'08"S, 079°29'54"W, 3120 m, 13 September 1964, *Hutchison 6578* (A!, MO!, USM!); Talaneo (Jalca - Cixse), 2900 m, 05 September 1976, *Sagástegui 8635* (MO!, UMO). Unknown minor area, La Cruz, 04 June 1961, *Acleto 364* (GOET!, USM!); Paso de la Cruz Blanca, 3307–3154 m, 13 November 2007, *Lasermann I5-8* (USM!); Paso de la Cruz Blanca, 3307 m, 13 November 2007, *Lasermann V/3* (USM!).

### ﻿19. Polylepis quadrijuga

Taxon classificationPlantaeRosalesRosaceae

Bitter, Bot. Jahrb. Syst. 45: 613, pl. 6. 1911.

A147270C-4E84-5C28-B5E1-FCB977ED9B8A

Figs 53 , 54

Polylepis boyacensis Cuatrecasas, Revista Acad. Colomb. Ci. Exact. 4: 343, f. 13–14. 1941. Type. Colombia. Boyacá: Cordillera Oriental, Páramo de Santa Rosita between Belen and Susacon, 3300–3400 m, 3 August 1940, *Cuatrecasas 10374* (holotype: P!; isotypes: COL!, US!).
Polylepis cocuyensis Killip & Cuatrecasas, Revista Acad. Colomb. Ci. Exact. 5(17): 33. F. 19.1942. Type. Colombia. Boyacá: Nevado del Cocuy in the Quebrada of San Paulino, El Morrón, 3800 m, 11 September 1938, *Cuatrecasas 1405* (holotype: US!; isotypes: P!, U!).

#### Type.

Colombia. Cundinamarca: Cordillera Oriental, Páramos de Bogota, Muzo, May 1844, *Goudot s.n* (holotype: P!).

**Figure 53.**
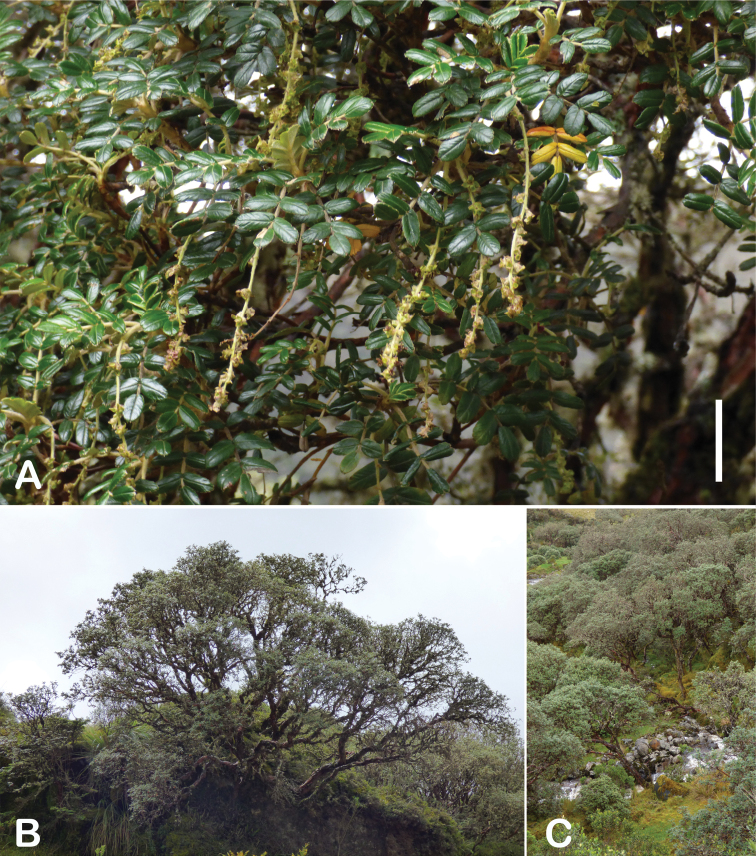
*Polylepisquadrijuga* Bitter **A** flowering branches **B** habit **C** habit. Scale bars: 3 cm (**A**). Photographs by D. Cabrera.

#### Description.

***Trees*** 2–10 m tall. ***Leaves*** slightly congested at the branch tips, imparipinnate with 3–4 pairs of lateral leaflets, obtrullate in outline, (4.2–)4.4–5.3(–6.5) × 2.4–3.2 cm; rachises densely tomentose, points of leaflet attachment with a tuft of long, lanate hairs; stipular sheaths apically acute with spurs, densely lanate on the outer surfaces; leaflets elliptic in outline, second pair from the terminal leaflet the largest, one of this pair 1.5–2.0 × 0.7–1.1 cm; margin entire or slightly crenate with 4–6 teeth, apically emarginate, basally unequally cordate; upper leaflet surfaces glabrous; lower leaflet surfaces densely tomentose with whitish hairs 0.7–0.9 mm long. ***Inflorescences*** pendant, (6.0–)7.3–10.5(–12.3) cm long, bearing 11–19 flowers; floral bracts 4.3–4.7 mm long, narrowly triangular, densely lanate on the outer surface; rachises tomentose. ***Flowers*** 6.8–9.5 mm diam.; sepals 4, ovate, green, densely tomentose outside; stamens 9–19, anthers orbicular, with a dense tuft of straight white hairs on the upper half; styles fimbriate, 3.7–4.6 mm long. ***Fruits*** turbinate, with variable numbers and placement of flattened spines, densely villous; 4.6–6.5(–7.2) × 5.1–7.2 mm including spines. ***Diploid***.

**Figure 54.**
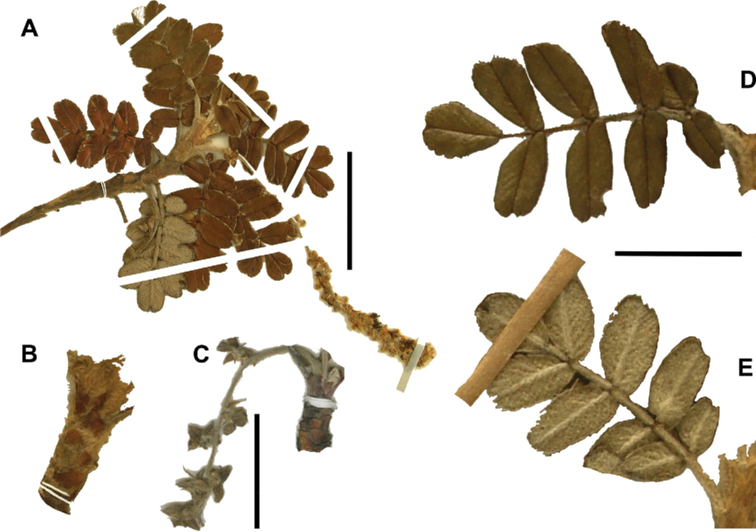
*Polylepisquadrijuga* Bitter **A** flowering branch **B** stipular sheaths **C** inflorescence **D** upper leaf surface **E** lower leaf surface (**A***Montenegro 2717***B***Huerta 6565***C***Cuatrecasas 27741***D, E***Stancik 2463*). Scale bars: 3 cm (**A**); 4 cm (**C**); 2 cm (**D, E**). Photographs by J. M. Vélez.

#### Distribution, habitat, and ecology.

*Polylepisquadrijuga* is found in north-eastern Colombia from Páramo de la Rusia (Santander) across Páramo Guina, Páramo Belen, Páramo Guantiva and Sierra Nevada Cocuy (Boyacá) to Maciso de Sumapáz and Chingaza National Natural Park (Cundinamarca) (Fig. 61). It grows in humid Páramos at 2200–4250 m elevation. *Polylepisquadrijuga* represents 33.7% (1032.2 ha) of the total area of *Polylepis* forest identified for Colombia (Fadiño and Caro 2009). It is often associated with *Diplostephiumtenuifolium* (Asteraceae) and *Escalloniamyrtilloides* (Escalloniaceae) (Rangel-Ch and Arellano 2010).

#### Conservation status.

The estimated EOO for *Polylepisquadrijuga* is 16,286 km^2^ and the AOO is 84 km^2^. It is known from 16 locations. *Polylepisquadrijuga* is protected within Chingaza and Sumapaz National Natural Parks and Cocuy National Park. Although the species occurs within these protected areas, it is also found in heavily fragmented forest regions. Perhaps the most worrying area is the corridor Rusia-Guantiva Páramos because of the absence of protected areas that would help to mitigate the current pressures (Rangel-Ch and Arellano 2007). The rising of the potato crop line is a strong threat to the permanence of these forests (Rangel-Ch and Arellano 2010), as is the reduction of the potential range due to climate change (Caballero-Villalobos et al. 2021). We assess *P.quadrijuga* as Vulnerable (A2a, B1a+B2a, D2a).

#### Notes.

*Polylepisquadrijuga* resembles *P.frontinensis*. It differs from it by its elliptic leaflets with tomentose hairs 0.7–0.9 mm long, whereas *P.frontinensis* has obovate leaflets with villous hairs 1.4–1.8 mm long. Further, *P.quadrijuga* is morphologically similar to *P.occidentalis*, with which it shares elliptic leaflets, with deeply emarginate apices, entire to slightly crenate margins and densely tomentose lower leaflet surfaces. However, *P.quadrijuga* has broader leaflets 0.7–1.1 cm, longer lower leaflet surface hairs 0.7–0.9 mm, longer inflorescences 6.0–12.3 cm, bearing 11–19 flowers and longer styles 3.7–4.6 mm, whereas *P.occidentalis* has leaflets 0.5–0.8 cm width, lower leaflet hairs 0.3–0.6 mm long, inflorescences 2.4–6.7 cm long with 4–12 flowers and styles 1.5–2.0 mm long.

#### Specimens examined.

**Colombia. Boyacá**: Duitama, Corregimiento El Carmen, vía a Virolin, Páramo de La Rusia, 3400–3500 m, 19 November 1994, *Betancur 5610* (COL!); Páramo de Rusia. Km 3 from the summit of the road Duitama-Abendaños, 3500 m, 12 February 1999, *Stancík 2384* (COL!). El Cocuy, Cordillera Oriental, Sierra Nevada del Cocuy, Quebrada de San Paulino próximo Alto Ritacuva, 3500 m, 07 April 1959, *Barclay 7303* (COL!, MO!); Cocuy National Park, Concavito Valley, 06°25'39"N, 072°19'15"W, 4000 m, 07 February 2017, *Olivares 570* (Z!). Guicán, Guican. Alto de la Cueva limite Cocuy-Guican, Finca Eudoro Carreño y Margarita Carreño, estación del Himat, 06°24'33"N, 072°22'40"W, 3930 m, 14 October 1996, *Cruz 164* (COL!). Susacon, 20 km passing Sussacon in direction to soata, 3150 m, 23 February 1999, *Stancík 2463* (COL!). Cordillera Oriwntal; Páramo de Guina-Santa Rosita, 3300–3400 m, 03 August 1940, *Cuatrecasas 10374* (US!); NNW de Duitama, Páramo de La Rusia, Avenida de Peñas Negras (Buenos Aires), 3550 m, 11 September 1989, *Cuatrecasas 27741* (COL!); Hoya del Río Cusiana, Vadohondo, 2880 m, 31 March 1973, *Cuatrecasas 28708* (COL!); Mpio. de Guican, vereda La Guaca, El Junco, 3716 m, 23 July 1981, *López s.n* (COL!); arriba de la confluencia con el río Upía, Río Olarte, 3015 m, 27 February 1955, *Molano s.n* (COL!); Páramo de Colorado, 3200 m, 11 May 1960, *Montenegro 2717A* (COL!). Cundinamarca: Pasca, Vereda Cajita, borde de Laguna Cajitas en el Páramo de Chisacá, 3700 m, 04 October 1983, *Sarmiento 2000* (COL!). Santafé de Bogotá, Macizo de Sumapáz; Cuchilla La Rabona, 04°04'40"N, 074°13'00"W, 3900–3950 m, 17 July 1981, *Díaz 2902B* (COL!, MO!); Bogotá, D.C. Páramo de Sumapaz. ladera de la laguna Tunjos (Chisacá), 04°16'36"N, 074°12'17"W, 3725 m, 19 February 1997, *Franco 5632* (COL!, MO!). Subachoque, Vereda Tobal, 2800 m, 09 November 2003, *Hernández 1407* (COL!); carretera Cogua-San Cayetano, cebeceras Río Guandoque, 3 km al SW de la Laguna Verde, 3400 m, 01 June 1972, *Cleef 4213* (COL!); Macizo de Bogotá, eastern slopes of Páramo de Chisaca. Quebrada de Santa Rosa, subPáramo, 3300–3350 m, 16 September 1961, *Cuatrecasas 25999* (COL!); Dist. Especial de Bogotá; cordillera Oriental; lagunas de Chisacá; 40 km al N de Sumapaz, 3300–4000 m, 10 September 1959, *García 17192* (AAU!); Fomeque, Páramo de Chingaza, a orillas de La Laguna, 3200 m, 03 November 1966, *Huertas 6565* (COL!); Mpio. Bogota, Páramo de Sumapaz, entre la Laguna de Chisaca y Santa Rosa, 01 May 1989, *Mahecha 10121* (COL!); Usme, al sur de Bogotá, 01 January 1944, *Ranghel s.n* (COL!); Macizo de Sumapaz, cerca a Santa Rosa, 3600–3700 m, 30 September 1963, *Uribe 4491* (COL!). **Santander**: Encino, Vereda Rionegro, Microcuenca de la quebrada Chontales, 05°59'N, 073°04'W, 3400–3500 m, 15 December 1999, *Galindo 300* (COL!).

### ﻿20. Polylepis reticulata

Taxon classificationPlantaeRosalesRosaceae

Hieron., Bot. Jahrb. Syst. 21: 312. 1896.

D6716462-D904-5CE7-B3EE-C85333DEE64A

Figs 55 , 56

Polylepis brachyphylla Bitter, Bot. Jahrb. Syst. 45: 616. 1911. Type. Ecuador. Bolivar: Volcán Chimborazo, slopes towards Guaranda, 4 November 1856, *Remy s.n* (holotype: P!; isotypes: GOET!, P!).
Polylepis nitida Bitter, Bot. Jahrb. Syst. 45: 615. 1911. Type. Ecuador. Tungurahua: Minza at Volcan Tungurahua, 3600 m, *Stübel 287* (holotype: B destroyed; photos at F!, GH, MO!, NY, US!).

#### Type.

Ecuador. Pichincha: near Las Calderas de Pasachoa and Rumiñaqui, *Stübel 20a.* (holotype: B destroyed; photos at F!, GH, NY, US).

**Figure 55.**
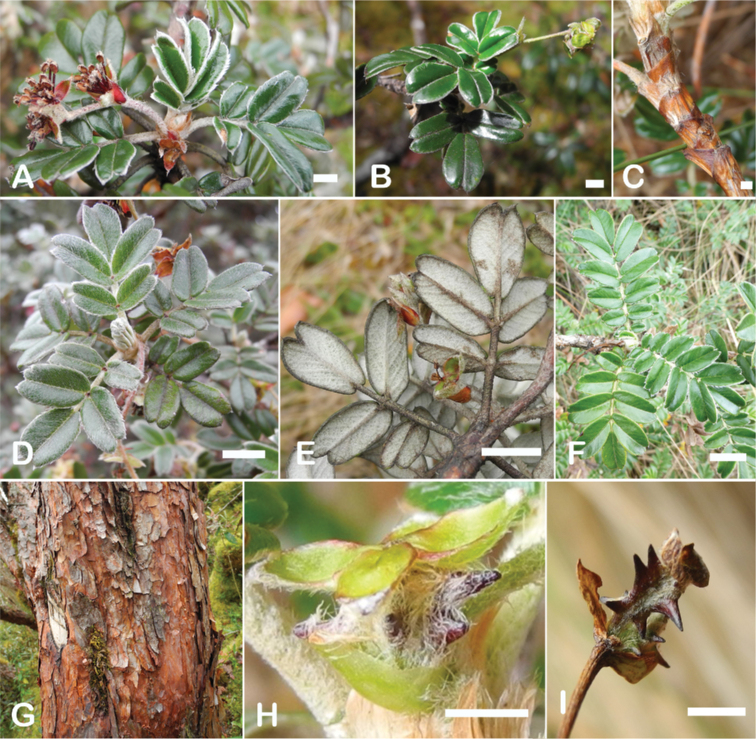
*Polylepisreticulata* Hieron **A** flowering branch **B** leaves **C** stipule sheaths **D** upper leaflet surfaces **E** lower leaflet surfaces **F** juvenile leaves **G** bark **H** fruit **I** dried fruit. Scale bars: 5 mm (**A, B**); 1 cm (**C–F**); 3 mm (**H**); 2 mm (**I**). Photographs by T. E. Boza E.

#### Description.

***Trees*** 2–15 m tall. ***Leaves*** slightly congested at the branch tips, imparipinnate with (1–)2–3(–4) pairs of lateral leaflets, obtrullate in outline, (1.3–)2.6–4.2(–7.0) × (1.5–)2.2–3.6 cm; rachises densely tomentose, points of leaflet attachment with a tuft of long, lanate hairs; stipular sheaths apically acute with spurs, densely lanate on the outer surfaces; leaflets elliptic to obovate in outline, second pair from the terminal leaflet the largest, one of this pair (0.9–)1.2–2.2 × 0.4–1.1 cm; margin entire or slightly crenate at the apex with 3–5 teeth, apically deeply emarginate, basally unequally cordate; upper leaflet surfaces glabrous; lower leaflet surfaces densely tomentose with whitish hairs 0.6–1.5 mm long. ***Inflorescences*** pendant, (2.3–)4.3–13.8) cm long, bearing 5–9 flowers; floral bracts 3.1–8.0 mm long, narrowly triangular, densely lanate on the outer surface; rachises tomentose. ***Flowers*** 6.2–12.2 mm diam.; sepals 4, ovate, green, densely tomentose outside; stamens 7–9, anthers orbicular, with a dense tuft of straight white hairs on the upper half; styles fimbriate, 2.6–3.9 mm long. ***Fruits*** turbinate, with variable numbers and placement of flattened spines, densely villous; 3.4–6.0 × 2.9–8.3 mm including spines. ***Diploid*** and ***hexaploid***.

**Figure 56.**
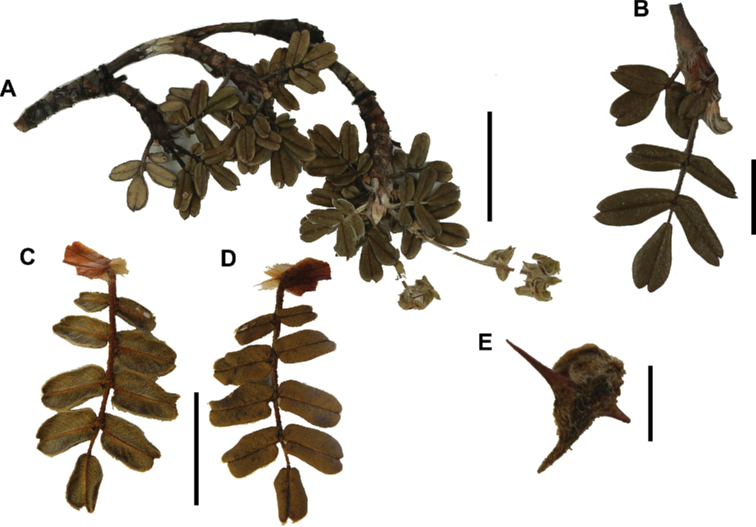
*Polylepisreticulata* Hieron **A** flowering branch **B** leaves **C** lower leaf surface **D** upper leaf surface **E** fruit (**A***Laegaard 102646***B***Laegaard 20601***C–E***Laegaard 52886*). Scale bars: 3 cm (**A, C, D**); 2 cm (**B**); 5 mm (**E**). Photographs by T. E. Boza E.

#### Distribution, habitat and ecology.

*Polylepisreticulata* is found in Carchi, Imbabura, Pichincha, Tungurahua, Bolivar, Chimborazo, Azuay and Loja (Ecuador) (Fig. 61). It grows under relatively dry conditions (except at Llanganates and Pasochoa that are more humid) in Andean forest at 2750–4600 m elevation. It often co-occurs with *P.simpsoniae* in Azuay. In Sacha Huayco Forest (Chimborazo) at 4000–4500 m elevation, the canopy layer is comprised almost completely by *P.reticulata*, with *Gynoxys* sp. dominating the subcanopy (Osha 2000). At 4300 m elevation in Chimborazo Wildlife Production Reserve, trees of *P.reticulata* are, on average, 2.8 m tall (maximum 8.2 m) with diameters of 8.6 cm (maximum 16.9 cm) (Castillo et al. 2017). In Cuenca (Ecuador), the minimum temperature threshold of vegetative growth activity was found to be close to 6 °C (Saravia et al. 2016). In the same forest, mean annual litterfall is 3.77 Mg ha^-1^, representing 51% of the canopy leaf biomass, indicating a leaf life span of 1.98 years and a mean decomposition rate of 0.38 year^-1^ (Pinos et al. 2017). Diameter growth rates measured over seven years average 1.2 mm/year and the average carbon sequestration rate is 2.6+/-0.3 Mg C. ha^-1^ year^-1^, which is high for this generally slow-growing genus (Montalvo et al. 2018). Tree rings of *P.reticulata* are characterized by semi-ring porosity and minor changes in fiber wall thickness between latewood and earlywood. Tree ring width is more related to temperature than precipitation (Alvites et al. 2019). Based on an AFLP study, clonal propagation affects the fine-scale genetic structure within a spatial distance of 3 m (Peng et al. 2015). In a forest of *P.reticulata*, the soil had low microbiological quality with low biological activity, even though there were some beneficial nematodes representing a great advantage for the elimination of insect pests that could affect *Polylepis* (Castillo et al. 2017). Hybrids with *P.pauta* have been found in Pichincha.

#### Conservation status.

The EOO for *Polylepisreticulata* is estimated as 33,103 km^2^, the AOO is assessed at 324 km^2^ and it is known from 40 locations. Most of the populations occur in protected areas. There are populations in the Cajas (Azuay), Llanganates (Tungurahua) and Yacuri (Loja) National Parks, Chimborazo Wildlife Production Reserve, Illinizas Ecological Reserve (Pichincha) and in part of Pasochoa Ecological Reserve. However, *P.reticulata* was categorized as Vulnerable VU (A4c) (León-Yánez et al. 2011). Although the species occurs within national parks, its populations are heavily fragmented. We assess *P.reticulata* as Vulnerable (B1a+B2a, C1).

#### Notes.

*Polylepisreticulata* shows some degree of morphological differentiation between juvenile and adult leaves, with the young leaves having more lateral leaflet pairs (4 versus 1–3), slightly crenate margins (versus entire) and sparsely tomentose in the upper leaflet surfaces (versus glabrous). This differentiation was the basis for the recognition of three species by Bitter (1911) in what is now recognized to be a single species.

*Polylepisreticulata* is morphologically closest *P.occidentalis*, with which it shares similar leaflet size, margin types and number of lateral leaflets pairs. It differs by its longer lower leaflet surface hairs (0.6–1.5 mm), longer inflorescences ((2.3–)4.3–13.8 cm) and longer styles (2.6–3.9 mm), compared to the shorter lower leaflet surface hairs (0.3–0.6 mm), shorter inflorescences (2.4–6.7 cm) and shorter styles (1.5–2.0 mm) of *P.occidentalis*. *Polylepisreticulata* is also morphologically close to *P.simpsoniae*, with which it shares similar leaflet surface hair type and density. The most obvious differences between *P.reticulata* and this species are leaflet length (0.9–2.2 cm versus 0.9–1.6 cm), length of hairs on the lower leaflet surfaces (0.6–1.5 mm versus 0.5–0.7 mm) and longer inflorescences ((2.3–)4.3–13.8 cm versus (2.5–)3.7–3.9(–5.5) cm).

#### Specimens examined.

**Ecuador. Azuay**: Chaucha, Páramo de Soldados, 02°53'S, 079°18'W, 3700–4000 m, 28–29 August 1985, *Lægaard 55114C* (AAU!). Cuenca, Páramo de Cajas, 3650–3890 m, s.d., *Boeke 631* (MO!, QCA!); Parque Nacional El Cajas, 02°50'S, 079°12'W, 3500 m, 19 September 1983, *Brandbyge 42269* (MO!); Along the río Matadero, west Cuenca, 03 March 1945, *Camp E-2000* (MO!); along the río Matadero, west Cuenca, 3500 m, 03 March 1945, *Camp E-2000* (MO!, NY, US!); Area Nacional de Recreación Cajas, Laguna Toreadora and Virgen Machay, 02°46'S, 079°13'W, 4000–4400 m, 18 December 1995, *Clark 1794* (MO!, QCNE); Parque Nacional El Cajas, Laguna Toreador, 02°53'S, 079°08'W, 3500 m, 19 April 1998, *Clark 4992* (AAU!, MO!); Area Nacional de Recreación “Cajas” Sayausi-Molleturo road W of Cuenca, 02°53'S, 079°59'W, 3910 m, 21 June 1989, *Dorr 6391* (AAU!); Parque Nacional de Cajas, WNW of Cuenca, 02°52'S, 079°09'W, 3450 m, 28 December 1979, *Holm-Nielsen 20927* (AAU!); Parque de Recreación Cajas, 4000–4100 m, 02 September 1983, *Jaramillo 7185* (AAU!, GB, QCA!); Parque Reacreacional Cajas, Strasse von Cuenca nach Morlleturo, 3900 m, 14 March 1991, *Kessler 2746B* (GOET!); Cuenca-Molleturo road ca. 5 km W of pass in Las Cajas, 02°49'S, 079°16'W, 3350 m, 01 May 1992, *Lægaard 102646* (AAU!, GOET!, QCA!); Parque Nacional de Las Cajas, along road Soldados-Angas, in pass app. 13 km from Soldados, 02°53'S, 079°17'W, 4000 m, 02 May 1992, *Lægaard 102691* (AAU!, GOET!, QCA!); *102696* (AAU!, QCA!); Páramo de las Cajas W of Cuenca, 02°47'S, 079°14'W, 4000–4150 m, 02 September 1984, *Lægaard 52870* (AAU!, QCA!); *52878* (AAU!, MO!, QCNE); *52885* (AAU!, MO!, QCNE); *52888* (AAU!); *52891C* (AAU!); Páramo de Soldados SW of Cuenca, 02°53'S, 079°17'W, 3700–3800 m, 24 October 1984, *Lægaard 53243* (AAU!); Páramo de Soldados, SW of Cuenca, 02°53'S, 079°18'W, 3750–3850 m, 03 March 1985, *Lægaard 53800* (AAU!, MO!); Páramo de Cajas, W of Cuenca, along new road, ca. 6–8 km W of pass, 02°48'S, 079°17'W, 3800 m, 31 March 1985, *Lægaard 53935* (AAU!, QCA!); Páramo de Soldados, 02°53'S, 079°18'W, 3700–4000 m, 28–29 August 1985, *Lægaard 55098* (AAU!); *55098C* (AAU!); *55098D* (AAU!); *55113* (AAU!); *55114A* (AAU!); *55114B* (AAU!); Páramo de Soldados-Angas, at highest point of road, 02°52'S, 079°17'W, 4000 m, 14 February 1988, *Lægaard 70122* (AAU!, MO!); Area Nacional Recreacional Cajas, sector Llavinco, 02°50'S, 079°20'W, 3450–3550 m, 09 January 1991, *León 2606* (USM!); Parque Nacional Cajas, km 32.1 desde redondel Cuenca-Molleturo, sendero este alrededor de Laguna Toreadora, 3850–3870 m, 08 January 2003, *Ulloa 1013* (BRIT, HA, K, MO!, QCNE, US!); *1014* (HA, MO!, NY, QCNE); Parque Nacional Cajas, km 28 redondel Cuenca-Molleturo, sendero izquierdo Laguna Patoquinoas, 3730–3800 m, 12 January 2003, *Ulloa 1130* (HA, ILLS, MO!); Parque Nacional Cajas, km 35 Cuenca-Molleturo, Centro de Información, Laguna Toreadora sendero oeste, 3880–3890 m, 16 January 2003, *Ulloa 1234* (HA, ILLS, MO!, QCNE). Molleturo, Páramo de las Cajas, W of pass, 02°46'S, 079°15'W, 3700 m, 27 August 1985, *Lægaard 55047* (AAU!, MO!, QCA!); Area Nacional Recreacional Cajas, sector La Toreadora, 02°50'S, 079°20'W, 3950 m, 12 January 1991, *León 2624* (USM!); Area nacional Recreacional Cajas, sector La Toreadora, 02°50'S, 079°20'W, 3800 m, 13 January 1991, *León 2651* (QCA!, USM!); Carretera Sayausi-Molleturo km 10–31, Parque Recreacional Las Cajas, 02°50'S, 079°15'W, 3350–3950 m, 28 November 1992, *Romoleroux 1482* (AAU!, QCA!). San Antonio, 3–5 km W of pass at Las Cajas, W of Cuenca, 02°40'S, 079°14'W, 3800–4000 m, 22 October 1984, *Lægaard 53199* (AAU!); Las Cajas, near Laguna Toreadora, 02°43'S, 079°12'W, 3900 m, 13 September 1983, *Larsen 45141* (AAU!, QCA!). San Joaquin, Páramo de Soldados SW of Cuenca, 02°53'S, 079°17'W, 3700–3800 m, 24 October 1984, *Lægaard 53235*; *53235B*; *53235D*; *53235F*; *53235H* ; *53245*; *53246* (AAU!); between Cuenca and Soldados, 02°57'S, 079°10'W, 2800–3000 m, 28 August 1985, *Lægaard 55081* (AAU!); at Soldados, 02°57'S, 079°14'W, 3200–3400 m, 28 August 1985, *Lægaard 55084* (AAU!); Páramo de Soldadas-Angas, at highest point of road, 02°52'S, 079°17'W, 3950–4050 m, 14 February 1988, *Lægaard 70101* (AAU!); Páramo de Soldadas-Angas, at highest point of road, 02°52'S, 079°17'W, 3200–3300 m, 14 February 1988, *Lægaard 70101A* (AAU!); *70101B* (AAU!); *70101C* (AAU!); *70101D* (AAU!). Sayausi, Parque Nacional de Cajas, WNW of Cuenca, 02°52'S, 079°09'W, 3450 m, 28 December 1979, *Holm-Nielsen 20926* (AAU!); Páramo de las Cajas W of Cuenca, 02°47'S, 079°14'W, 4000–4150 m, 02 September 1984, *Lægaard 52879*; *52886*; *52890*; *52891*; *52891A*; *52891B*; *52891D* (AAU!); Cuenca–Area Recreacional Las Cajas, 26–30 km de Cuenca, 02°50'S, 079°06'W, 3360–3500 m, 17 November 1991, *Romoleroux 1187* (AAU!, QCA!). Zurucucho, Canton Cuenca, 3200 m, 11 August 1978, *Boeke 2622* (AAU!); ca. de la laguna La Toreadora, 3850 m, 26 December 1997, *Burnham 3007* (QCA!); Dudahuaycu, Mazán, 3300 m, 28 May 1994, *Chacón 211* (QCA!); Cantón Cuenca, collection made along río Patul from the Comuidad Baute Laguna Patul, watershed of río Patul, 3500–4200 m, 05 February 2001, *Clark 6229* (QCA!); Parque Nacional Cajas, morenas ca. de la Laguna Toreadora, 4025 m, 15 November 2000, *Endara 520* (QCA!); Parque Nacional Cajas, morrenas cerca de la Laguna de Soldados, 3830 m, 17 November 2000, *Endara 564* (QCA!); ca. 10 km W of Sayausid, 3300 m, 04 November 1982, *Harling 20218* (AAU!, GB); above Sayausi at first bridge over Río Tomebamba, 3200 m, 03 March 1985, *Harling 22671* (AAU!, GB, QCA!); Páramo de Cajas, versant oriental; zone très humide; relique forestière dégradée, 3650 m, 10 April 1989, *Huttel 1668* (QCA!); Páramo de Cajas, versant oriental, zone très humide, 3350–3950 m, 14 April 1988, *Huttel 995* (QCA!); carretera Cuenca–Sayausid-Area de Recreación Cajas, 3900 m, 16 August 1987, *Jaramillo 9898* (MO!); Parque Nacional Cajas, near headquarters on cuenca-Guayaquil road, 3900 m, 12 March 1991, *Kessler 2746* (QCA!); Parque Recreacional Cajas, Strasse von Cuenca nach Molleturo, 3900 m, 14 March 1991, *Kessler 2746A* (GOET!); Páramo de Soldados, 3700 m, s.d., *Lægaard 55096* (QCA!); *55114* (QCA!); at bridge 2 km below Soldados, 3200 m, s.d., *Lægaard 70082* (QCA!); at bridge 2 km below Soldados, 3200 m, s.d., *Lægaard 70083* (QCA!); s.d., *León 2527* (QCA!); colección en parcela del bosque, arriba del lago Dos Chorreras, 3940 m, 05 July 1995, *León 3541* (QCA!); arriba de laguna Chorreras, 3940 m, 05 July 1995, *León 3542* (AAU!, QCA!); colección en bosque en la base del valle de Cajas cerca del hotel Dos Chorreras y en la ladera SO de la propiedad Dos Chorreras, 3400 m, 06 July 1995, *León 3577* (QCA!); Vertiente oriental, 3100 m, 07 July 1995, *León 3611* (QCA!); Área recreacional Cajas, 4000 m, 21 September 2000, *Lizarzaburu 24* (QCA!); Fierroloma. Zorrocucho, ribera de Río Taitachugo, 3400 m, 20 May 1997, *Minga 125* (QCA!); Totorococha-Mazan valley, 3500–4000 m, 12 September 1987, *Ramsay 507* (QCA!); Parque Nacional Cajas, 3850–3900 m, 10 September 2001, *Romoleroux 4028* (QCA!); Area de Recreación Cajas. km 1 de la via Miguir-Naranjal, 3500, 16 August 1987, *Romoleroux 404* (QCA!); Hacienda Gulag, Masan, 3000–3200 m, 17 August 1987, *Romoleroux 417* (QCA!); carretera Soldados-Angas, 3620 m, 04 April 2007, *Romoleroux 4459* (QCA!); Bosque San Luis, 3939 m, 20 August 2008, *Romoleroux 5108* (QCA!); *5127* (QCA!); carretera Molleoto Naranjal, parches de bosque fuera del parque nacional Cajas, 3939 m, 21 August 2008, *Romoleroux 5157* (QCA!); Baños, 3300 m, 17 July 2006, *Salgado 165* (QCA!); Cajas, 3725–3830 m, 24 June 1999, *Smeets 432* (QCA!); Río Machangara, NW Cuenca, 3300–3400 m, 17 September 1967, *Sparre 18568* (AAU!, S); s.d., *Valverde 912* (MO!); carretera Sayausí-Quinona-Tres cruces, Area recreacional Cajas, 3500–4050 m, 10 April 1987, *Zak 1894* (AAU!). **Bolívar**: upper west mountain Chimborazo, 3500–4000 m, 19 June 1887, *Lehmann 4457* (MO!). **Cañar**: Vía Queseras-Rumiloma (Fund. Cordillera Tropical), 3353 m, 03 October 2006, *Salgado 1181* (QCA!). **Carchi**: Espejo, parche de bosque alrededor de laguna Rasococha, 00°44'N, 078°04'W, 3600 m, 09 November 1993, *Palacios 11897* (AAU!, MO!, QCNE). **Chimborazo**: Pungala, Near Alao, along Río Alao, 01°52'S, 078°30'W, 3200–3400 m, 21–22 September 1985, *Lægaard 55295* (AAU!). Riobamba, Reserva Faunistica Chimborazo, 01°32'41"S, 078°53'05"W, 4100 m, 01 June 2006, *Caranqui 1541* (CHEP, MO!); 3350–3500 m, 01 March 1944, *Acosta-Solís 7524* (F!); 3750 m, s.d., *Acosta-Solís 7696* (F!); 4000 m, s.d., *André 3933* (F!, US!); San Juan, Chimborazo, 4350 m, 05 November 2013, *Cardoso 4262* (QCA!); San Juan. Pasguazo Sambrano, 4100 m, 31 October 2013, *Cardoso 4266* (QCA!);carretera Penipe a Utuñac Paramito, 2800 m, 07 April 2008, *Jaramillo 26496* (QCA!); Sector Utuñac, Pijipamba, 3250–3330 m, 03 December 2008, *Jaramillo 28123* (QCA!); Sector Utuñac, Pijipamba, 2800 m, 04 December 2008, *Jaramillo 28132* (QCA!); E of Cordillera, 3400 m, s.d., *Rimbach 14 p.p.* (F!, NY, US!); 3400 m, s.d., *Rimbach 156* (US!); s.d., *Rimbach 498* (S); Vía Chambo-Guayllabamba-Aloa, 2900–3000 m, 09 August 1987, *Romoleroux 366* (NY, QCA!). **Imbabura**: 4000–4300 m, s.d., *Hirsch E-113* (NY). **Loja**: Manu, Cerro de Arcos W of road Manu-Zaruma, 03°34'S, 079°28'W, 3250–3600 m, 14 September 1999, *Lægaard 20601* (AAU!, MO!). **Napo**: 3 km E of Paso de la Virgen on road Pifo-Papallacta, 3950–4050 m, 06 February 1985, *Lægaard 54450* (QCA!); Parque Nacional Llanganates, 12 February 2009, *Salgado 820* (QCA!). **Pichincha**: Cotogchoa, Volcán Pasochoa, Páramo and forest remains, 00°27'S, 078°30'W, 3500 m, 16 September 1985, *Lægaard 55269A* (AAU!, MO!); *55278* (AAU!, MO!); Bosque Protector Pasochoa, 30 km SE de Quito, 00°27'S, 078°28'W, 2850–3900 m, 08 September 1986, *Romoleroux 161*; *171* (AAU!, MO!, QCA!). El Chaupi, road El Chaupi-Iliñiza, 00°37'S, 078°40'W, 3200–3700 m, 20 June 1985, *Lægaard 54547* (AAU!, QCA!); *54548* (AAU!, MO!); Volcán Iliñiza, N-side, 00°38'S, 078°41'W, 4200–4300 m, 20 June 1985, *Lægaard 54554*; *54555* (AAU!, MO!, QCA!). Rumiñahui, Bosque Protector Pasochoa, 00°22'S, 078°27'W, 3870–3900 m, 17 November 1990, *Cerón 12323* (MO!). Rumipamba, Volcán Pasochoa, 00°27'S, 078°30'W, 2900–3300 m, 16 September 1985, *Lægaard 55265* (AAU!, GOET!, MO!). Tambillo, Volcán Pasochoa, above house of Fundación Natura, 00°27'S, 078°31'W, 2800–3300 m, 27 April 1985, *Lægaard 54176* (AAU!, MO!, QCA!); Volcán Pasochoa, 00°27'S, 078°30'W, 3500 m, 16 September 1985, *Lægaard 55273* (AAU!, MO!). s.d., *Holmgren 962* (S); Volcán Iliñiza, N-side, 4200–4300 m, 20 June 1985, *Lægaard 54557* (QCA!); Volcán Pasochoa, Páramo and forest remains, 3500 m, 16 September 1985, *Lægaard 55266*; *55269B*; *55274* (QCA!); Bosque Protector Pasochoa, 30 km SE de Quito, 2850 m, 29 October 1988, *Paz 105* (MO!, QCA!); Faldas del Pasochoa, colección al Sur-Oeste, 3800 m, 03 March 1980, *Raza 167* (AAU!); Bosque Protector Pasochoa, 30 km SE de Quito, 2850–3900 m, 17 July 1986, *Romoleroux 141* (AAU!, QCA!); *162*; *164*; *166*; *295* (AAU!, QCA!); Pasochoa, 3556 m, 20 August 2008, *Romoleroux 5367* (QCA!); Volcán Rumiñahui, 4000 m, 19 December 2008, *Salgado 595* (QCA!). **Tungurahua**: Mocha, Parroquia Pinguili, Reserva Faunístic del Chimborazo, pared de valle en U, 2 km antes de la Base del Lado Oriental del Caryguayrazo, cerro Pocacochas, páramo de Salasaca entrando por Mocha, 01°24'S, 078°45'W, 3860 m, 02 July 1992, *Cerón 19203* (MO!). Pilaguin, Sabanza, about 5 km SE of Yatzapuzan, 01°19'S, 078°46'W, 3950–4050 m, 21 December 1982, *Brandbyge 42024* (AAU!, MO!); Río Negro, Cordillera de Llanganates, loma between Río Topoand Río Verde Grande, 3.5 km NW of Cerro Hermoso, 01°12'S, 078°18'W, 4000 m, 10 November 1980, *Holm-Nielsen 28297* (AAU!); Cordillera de Llanganates, NW of saddle between Río Topo and Río Verde Grande on W slope of Cerro Hermoso, 2.5 km from summit, 01°13'S, 078°18'W, 3950 m, 10 November 1980, *Holm-Nielsen 28330* (AAU!); *28371* (AAU!); Cordillera de los Llanganates, at Río Verde Grande at base of Cerro Hermoso, 2 km WSW of the summit, 01°13'S, 078°18'W, 3800 m, 11 November 1980, *Holm-Nielsen 28403* (AAU!); *28641* (AAU!); Cordillera de los Llanganates, loma 2.5 km W of Cerro Hermoso, 01°13'S, 078°18'W, 3800 m, 12 November 1980, *Holm-Nielsen 28662* (AAU!); Quinuales, 01°13'18"S, 078°18'16"W, 3890 m, 15 February 2010, *Romoleroux 5525* (MO!, QCA!, QCNE); *5529* (MO!, QCA!, QCNE); *5533* (MO!, QCA!, QCNE). Río Verde, Cordillera de los Llanganates, at Río Verde Grande at base of Cerro Hermoso, 2.5 km SW of the summit, 01°14'S, 078°18'W, 3700 m, 12 November 1980, *Holm-Nielsen 28538* (AAU!); *28599* (AAU!); *28609* (AAU!). San Fernando, Quebrada Huarcusacha, 01°15'S, 078°47'W, 3800–3900 m, 27 January 1983, *Brandbyge 42040* (AAU!, MO!); Near Calamaca, app. 20 km W of Ambato along old road Ambato-Guaranda, 01°16'S, 078°48'W, 3400–3900 m, 22 June 1985, *Lægaard 54569* (AAU!, QCA!); *54574* (AAU!, MO!, QCA!); *54576* (AAU!, MO!, QCA!). Santiago de Píllaro, Parque Nacional Llanganates, slopes of metamorphic rock, west of Cerro Hermoso, near saddle between headwaters of Río Verde and Río Topo, 01°11'40"S, 078°19'34"W, 3950 m, 12 November 1999, *Neill 11944* (MO!, QCNE); 3500 m, s.d., *Acosta-Solís 8736* (F!); 4200 m, s.d., *Asplund 8464* (S, WIS); Páramo de Yatsaputzan, 3600 m, 05 February 2010, *Caranqui 1912* (QCA!); Tamboloma, 3721 m, 29 April 2012, *Caranqui 2052* (QCA!); Minza mountain Travesia de Utañag, 3600 m, s.d., *Jameson s.n* (US!, K); 3400 m, s.d., *Penland 420* (F!, US!); Entrando por Baquerizo Moreno hacia el sector de Lagartococha, 3270 m, 01 March 2015, *Pérez 8424* (QCA!); Carretera Mocha-Hda. Atillo, 3000 m, 25 April 1988, *Romoleroux 567* (AAU!, MO!, QCA!); *570* (AAU!, QCA!); Sendero El Triunfo-Los Llanganates, localidad El Playón, 3600–3800 m, 02 March 1989, *Romoleroux 681* (AAU!, MO!, QCA!); 3200 m, s.d., *Rimbach 26* (F!); s.d., *Stübel 201* (MO!).

### ﻿21. Polylepis simpsoniae

Taxon classificationPlantaeRosalesRosaceae

T.Boza & M.Kessler sp. nov.

FF850D94-AFD8-5172-823F-6CB08BF48C65

urn:lsid:ipni.org:names:77301641-1

Figs 57 , 58

#### Diagnosis.

The species differs from *Polylepisweberbaueri* Pilg. in the smaller, broadly ovate leaflets (0.9–1.6 × 0.4–1.1 cm) and shorter inflorescences (2.5–5.5 cm long) bearing 3–5 flowers, with each flower having 11–13 stamens.

**Figure 57.**
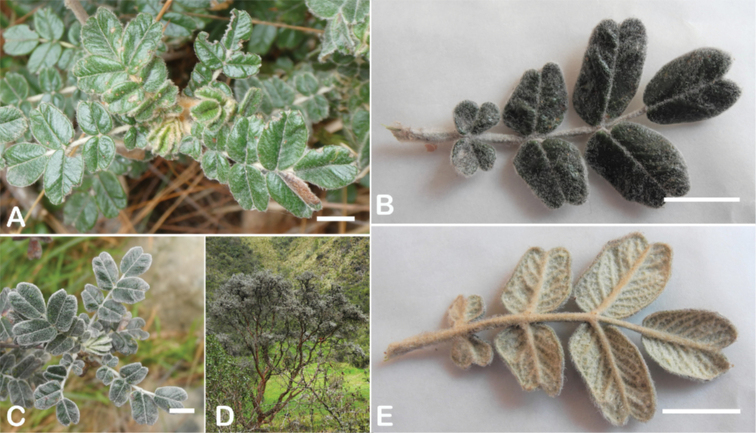
*Polylepissimpsoniae* T.Boza & M.Kessler **A** leaves **B** upper leaf surface **C** leaves **D** habit **E** lower leaf surface. Scale bars: 1 cm (**A–E**). Photographs by T.E. Boza E.

#### Type.

Ecuador: Cañar: along Páramo-road to Manu W of Cañar, 02°33'S, 79°02'W, 3300–3700 m, 20 June 1988, *S. Laegaard 71564* (holotype: QCA!; isotype: AAU!).

**Figure 58.**
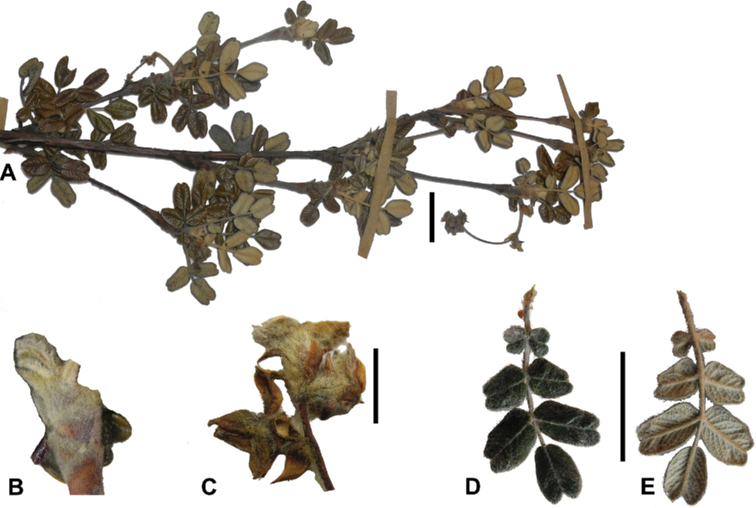
*Polylepissimpsoniae* T.Boza & M. Kessler **A** flowering branch **B** stipular sheaths **C** fruit **D** upper leaf surface **E** lower leaf surface (**A***Romoleroux 589***B***Laegaard 102679***C***Laegaard 55087***D, E***Laegaard 70084*). Scale bars: 2 cm (**A**); 5 mm (**C**); 3 cm (**D, E**). Photographs by T. E. Boza E.

#### Description.

***Trees*** 1–8 m tall. ***Leaves*** slightly congested at the branch tips, imparipinnate with 2–3 pairs of lateral leaflets, obtrullate in outline, (2.3–)2.8–5.8 × (1.5–)1.9–3.3 cm; rachises densely tomentose, points of leaflet attachment with a tuft of long, lanate hairs; stipular sheaths apically acute with spurs, densely lanate on the outer surfaces; leaflets elliptic in outline, second pair from the terminal leaflet the largest, one of this pair (0.9–)1.3–1.6 × (0.4–)0.7–1.1 cm; margin entire or slightly crenate with 2–4 teeth, apically emarginate, basally unequally cordate; upper leaflet surfaces glabrous; lower leaflet surfaces densely tomentose with whitish hairs 0.5–0.7 mm long. ***Inflorescences*** pendant, (2.5–)3.7–3.9(–5.5) cm long, bearing 3–5 flowers; floral bracts 3.3–6.1 mm long, narrowly triangular, densely lanate on the outer surface; rachises tomentose. ***Flowers*** (6.1–)6.7–9.1 mm diam.; sepals 4, ovate, green, densely tomentose outside; stamens 11–13, anthers orbicular, with a dense tuft of straight white hairs on the upper half; styles fimbriate, 2.8–3.3 mm long. ***Fruits*** turbinate, with variable numbers and placement of flattened spines, densely villous; 2.4–5.8 × 5.1–7.5 mm including spines. ***Diploid***.

#### Distribution, habitat, and ecology.

*Polylepissimpsoniae* occurs in southern Ecuador (Cañar and Azuay) (Fig. 61). It grows on relatively dry slopes at 2500–3800 m elevation, where it often co-occurs with *P.reticulata*. When they co-occur, *P.simpsoniae* tends to occur at lower elevation (maximum probability of occurrence at 3300–3700 m) than *P.reticulata* (3800–4000 m) (Montalvo et al. 2018).

#### Etymology.

We name the species in honor of Beryl B. Simpson (1942–), professor emeritus in the Department of Integrative Biology at the University of Texas at Austin, who, apart from her important contributions to the knowledge of *Polylepis* (Simpson 1979, 1986), has conducted remarkable research on tropical angiosperms.

#### Conservation status.

Based on 15 collecting localities, the estimated EOO is 12,905 km^2^, and the AOO is 92 km^2^. It has been collected in Cajas National Park, but populations there are heavily fragmented and degraded. Indeed, many *P.simpsoniae* forests occur at low elevations outside of the protected area where they are subject to continuing fragmentation and habitat degradation from livestock grazing and firewood extraction. We assess *P.simpsoniae* as Endangered (A1, B1a+B2a, C2a).

#### Notes.

*Polylepissimpsoniae* was treated as *P.weberbaueri* by previous authors (e.g. Simpson 1979; Romoleroux 1996), including within *P.weberbaueri* populations from central Ecuador and northern Peru. However, based on its distinct morphology and ecology, this taxon should be recognized as different from *P.weberbaueri. Polylepissimpsoniae* resembles *P.weberbaueri* in having 2–3 leaflet pairs and same type and density of hairs, but it has broadly ovate leaflets and smaller (0.9–1.6 × 0.4–1.1 cm) leaflets than *P.weberbaueri*, which has elliptic leaflets 1.6–2.1 × 0.6–0.9 cm. Further, *P.simpsoniae* has shorter inflorescences (2.5–5.5 cm) bearing 3–5 flowers, with each flower having 11–13 stamens, whereas *P.weberbaueri* has longer inflorescences (8.2–9.7 cm) bearing 9–11 flowers, with each flower having 19–21 stamens.

#### Specimens examined.

**Ecuador. Azuay**: Baños, Parque Nacional de Las Cajas, along road Soldados-Angas app. 2 km above Soldados, 02°56'S, 079°12'W, 3480 m, 02 May 1992, *Lægaard 102679* (AAU!, QCA!). Cuenca, Area Nacional de Recreación Cajas, collection made along Río Patul from the Comunidad Baute/Laguna Patul (watershed of Río Patul), 02°33'S, 079°21'W, 3500–4200 m, s.d., *Clark 6229* (QCA!, QCNE, US!). Jimbilla, along road Cuenca–Soldados, 03°50'S, 079°09'W, 3000–3300 m, 03 March 1985, *Lægaard 53795* (AAU!, MO!). San Joaquin, from Soldados eastwards along the road until Hda. San Vicente (west of Cuenca), 02°56'S, 079°10'W, 3200–3300 m, 05 January 1981, *Balslev 1546* (AAU!, NY, QCA!); Parque Nac. de Las Cajas, at entrance to the park at Soldados, 02°56'S, 079°13'W, 3900 m, 02 May 1992, *Lægaard 102677* (AAU!, GOET!); Páramo de Soldados, SW of Cuenca, 02°57'S, 079°14'W, 3020 m, 24 October 1984, *Lægaard 53234* (AAU!, MO!); between Cuenca and Soldados, 02°57'S, 079°10'W, 2800–3000 m, 28 August 1985, *Lægaard 55082* (AAU!); at Soldados, 02°57'S, 079°14'W, 2800–3000 m, 28 August 1985, *Lægaard 55083* (AAU!); *55087* (AAU!, GOET!, MO!); at bridge 2 km below Soldados, 02°56'S, 078°13'W, 3200 m, 14 February 1988, *Lægaard 70082* (AAU!, QCNE); *70084*; *70085* (AAU!, MO!); cerca al Río Yanuncay, carretera via San Joaquin-Soldados Angas, 02°56'S, 079°13'W, 3100 m, 07 April 2007, *Romoleroux 4445* (CHEP, ECUAMZ, QCA!). Sayausi, Las Cajas National Park. Wet páramo near roadside, 02°50'S, 079°12'W, 3500 m, 19 September 1983, *Brandbyge 42269* (AAU!, MO!, QCA!); from Soldados E along the road until Hcda. San Vicente (W of Cuenca), 3200–3300 m, 05 January 1981, *Balslev 1546* (QCA!); Zurucucho, Cantón Cuenca, Valley bottom, along rivers, 3200 m, 11 August 1978, *Boeke 2622* (QCA!); route de Cuenca à Angas, páramo de Cajas, peu au dessus du hameau de Soldados, 14 km en desous du col sur le versant intérieur, 3250 m, 05 October 1988, *Huttel 1136* (QCA!); Páramo de Soldados, SW of Cuenca, 3320 m, 24 October 1984, *Lægaard 53234* (QCA!); between Cuenca and Soldados, 2800–3000 m, 28 August 1985, *Lægaard 55082* (QCA!); at Soldados, 3200–3400 m, 28 August 1985, *Lægaard 55083* (QCA!); at Soldados, 3200 m, 28 August 1985, *Lægaard 55087* (QCA!); at bridge 2 km below Soldados, 3200 m, 14 February 1988, *Lægaard 70084* (QCA!); Parque Nacional Cajas, 3850–3900 m, 10 September 2001, *Romoleroux 4028* (QCA!); Est. San Pedro near Canar on steep slopes, 02 January 1977, *Simpson 8532* (US!, USM!); *8532a* (USM!); Cajas, Río Blanco, 3620 m, 28 August 1999, *Smeets 791* (QCA!). **Cañar**: Gualleturo, along Páramo–road to Manu W of Cañar, W of pass, 02°33'S, 079°02'W, 3700 m, 20 June 1988, *Lægaard 71561*; *71564* (AAU!, QCA!); *71567* (MO!). Ingapirca, Comunidad San Isidro de Vendeleche, 02°32'S, 078°50'W, 3200 m, 04 March 1983, *Brandbyge 42085* (AAU!, MO!, NY); Ingapirca Parish, Silante, 02°29'S, 078°49'W, 3450 m, 01 January 1992, *Kohn 1526* (MO!). Zhud, along road to Culebrillas ca. km 6–10, 02°26'S, 078°57'W, 3400–3500 m, 04 February 2000, *Lægaard 21006* (AAU!, MO!); at the antennas of Culebrillas ca. 17 km from Panamericana, 02°26'S, 078°57'W, 4000 m, 04 February 2000, *Lægaard 21031* (AAU!, MO!). Guapán, 3525 m, 28 November 2000, *Endara 760* (QCA!); along páramo road to Manu W of CaÒar. W of pass, 3300–3700 m, 20 June 1988, *Lægaard 71561* (QCA!); Pimo, entre Oyacshi y Zhud, 3250 m, 13 October 1987, *Romoleroux 392* (AAU!, NY, QCA!); Desde Cañar, km 10.5, Cerro Caucay, 3450 m, s.d., *Romoleroux 586* (AAU!, QCA!); Oeste de Cañar, Km 10.5, Cerro Cuacay, 3450 m, 27 April 1988, *Romoleroux 587* (AAU!, MO!, QCA!); *589* (AAU!, QCA!); s.d., *Rose 22722*; *22733*; *23792* (NY, US!); Cuenca del Río Dudas (Fund. Cordillera Tropical), 3230 m, 17 October 2006, *Salgado 1292* (QCA!). **Chimborazo**: Chimborazo/Cañar: between Santa Rosa and Joyagshi, near Tipococha, 3500 m, s.d., *Camp E-4088* (F!, NY, US!). **Tungurahua**: Bellavista, 3700 m, 05 December 2009, *Caranqui 1907* (QCA!).

### ﻿22. Polylepis weberbaueri

Taxon classificationPlantaeRosalesRosaceae

Pilg., Bot. Jahrb. Syst. 37: 535. 1906.

9ACA7CE9-9D18-512E-811C-BD1F84A22A87

Figs 59 , 60

#### Type.

Peru. Ancash: from Yanganuco to Yungay, 3800 m, 16 June 1903, *Weberbauer 3287* (holotype: B destroyed; photos at F, GH, MO!, NY).

**Figure 59.**
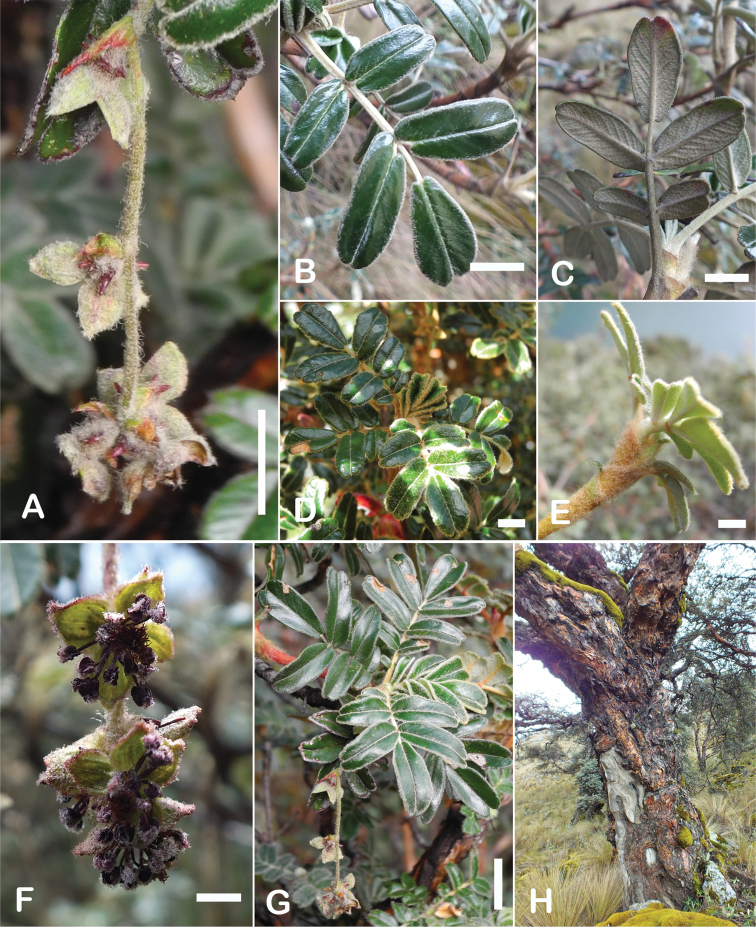
*Polylepisweberbaueri* Pilg **A** fruits **B** upper leaflet surface **C** lower leaflet surface **D** leaves **E** stipule sheaths **F** flowers **G** flowering branch **H** bark (**A, D–G***Boza & Urquiaga 3021***B, C, H***Boza & Urquiaga 3017*). Scale bars: 2 cm (**A**); 1 cm (**B–D**); 2 mm (**E**); 3 mm (**F**); 3 cm (**G**). Photographs **B, C, F–H** E. G. Urquiaga F. **A, D, E** T.E. Boza E.

#### Description.

***Trees*** 1–14 m tall. ***Leaves*** slightly congested at the branch tips, imparipinnate with 2–3 pairs of lateral leaflets, obtrullate in outline, (3.2–)5.2–5.8 × (2.7–)3.2–3.5 cm; rachises densely tomentose, points of leaflet attachment with a tuft of long, lanate hairs; stipular sheaths apically acute with spurs, densely lanate on the outer surfaces; leaflets elliptic in outline, second pair from the terminal leaflet the largest, one of this pair 1.6–2.1 × 0.6–0.9 cm; margin entire or slightly crenate at apex with 3–4 teeth, apically emarginate, basally unequally cordate; upper leaflet surfaces glabrous; lower leaflet surfaces densely tomentose with whitish hairs 0.4–0.5 mm long. ***Inflorescences*** pendant, 8.2–9.7 cm long, bearing 9–11 flowers; floral bracts 5.4–7.5 mm long, narrowly triangular, densely lanate on the outer surface; rachises tomentose. ***Flowers*** 8.6–10.4 mm diam.; sepals 4, ovate, green, densely tomentose outside; stamens 19–21, anthers orbicular, with a dense tuft of straight white hairs on the upper half; styles fimbriate, 2.7–3.2 mm long. ***Fruits*** turbinate, with variable numbers and placement of flattened spines, densely villous; 4.2–4.9 × 4.8–5.3 mm including spines. ***Diploid***.

**Figure 60.**
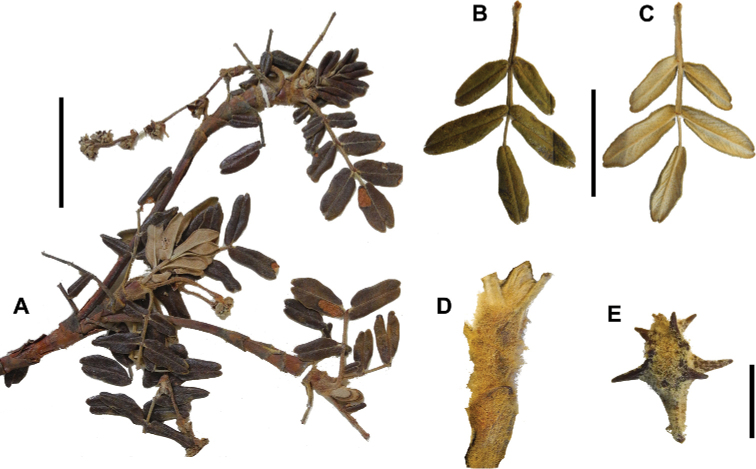
*Polylepisweberbaueri* Pilg **A** flowering branch **B** upper leaf surface **C** lower leaf surface **D** stipular sheaths **E** fruit (**A***Smith 10608***B–E***Boza 3017*). Scale bars: 5 cm (**A**); 3 cm (**B, C**); 3 mm (**E**). Photographs by T. E. Boza E.

#### Distribution, habitat and ecology.

**Figure 61.**
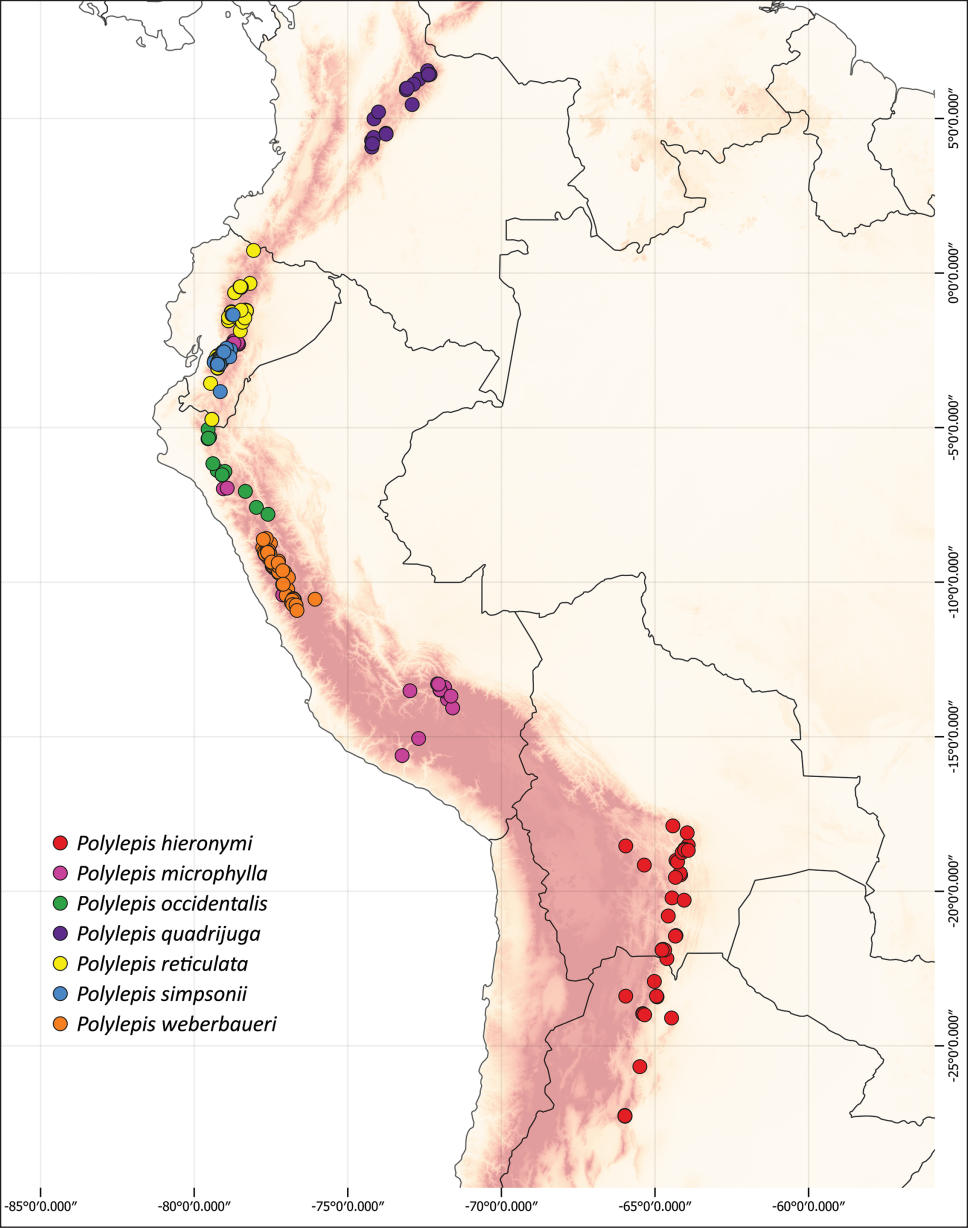
Geographical distribution of the species of section Reticulatae.

*Polylepisweberbaueri* is distributed in northern Peru in Ancash, Huánuco and Lima (Fig. 61). It occurs in dry and cold Andean habitats at 3500–4970 m elevation. Some stands of *P.weberbaueri* occur in semi-humid areas mixed with *Gynoxys*, *Oreocallis*, *Alnus*, *Berberis* and *Buddleja*, but it can also be found in large, homogeneous stands with dense vegetation cover and very robust trees (Sevillano-Ríos et al. 2011). It often co-occurs with *P.albicans*, but *P.weberbaueri* tends to occur at higher elevations (ECOAN 2005; Fuentealba and Sevillano 2016; Sevillano-Ríos and Rodewald 2017; Morales et al. 2018). In a study in Huascarán National Park, seedling density of *P.weberbaueri* increased with elevation and decreased with solar radiation (Morales et al. 2018). These forests also support the hemiparasite *Tristerixchodatianus* (Patschovky) Kuijt (Loranthaceae) at over 4400 m elevation (ECOAN 2005; Sevillano-Ríos et al. 2011). *Polylepisweberbaueri* forests at higher elevations (> 4000 m) harbor some of the most specialized and endangered bird species (Sevillano-Ríos et al. 2011; Sevillano-Ríos and Rodewald 2017).

#### Conservation status.

The EOO is estimated as 15 388 km^2^ and AOO as 224 km^2^. The species has been collected in 39 locations. It is protected within Huascarán National Park and was categorized as VU (SERFOR 2006). It is subject to reforestation activities in Huascarán National Park (Fuentealba and Sevillano 2016). We assess *P.weberbaueri* as Vulnerable (B1a B2a).

#### Notes.

*Polylepisweberbaueri* can be distinguished from the most similar species *P.occidentalis* by the number of lateral leaflet pairs (2–3 versus 3–5), inflorescence length (8.2–9.7 cm versus 2.4–6.7 cm), style length (2.7–3.2 mm versus 1.5–2.0 mm) and number of stamens (19–21 versus 9–15).

#### Specimens examined.

**Peru. Ancash**: Bolognesi, Pachapaque, Distr de Aquia, 3900 m, 01 August 1972, *Cerrate 2* (USM!); entre Llamac y Jahuacocha, 4900 m, 29 May 1954, *Cerrate 2314* (USM!); road from Abra Janashalla down to Huallanca, below Huansalá, 09°52'05"S, 076°59'27"W, 3390 m, 08 October 2007, *Weigend 8810* (USM!). Carhuaz, Sonquenua, Shilla, 3950 m, 21 December 1989, *Arce 187* (MO!); Huascarán National Park. Quebrada Ishinca, north side of valley, 09°23'S, 077°26'W, 4110–4350 m, 15 July 1985, *Smith 11169* (MO!, USM!); Huascarán National Park, Quebrada Ishinca, south side of valley, 09°23'S, 077°26'W, 4200–4260 m, 17 July 1985, *Smith 11236* (AAU!, F!, MO!, USM!); Huascarán National Park. Quebrada Ulta, on road to Ulta Pass, WNW-facing, steep slope, 09°08'S, 077°32'W, 4280 m, 29 July 1985, *Smith 11383* (MO!, USM!); *11659* (F!, MO!, USM!); Huascarán National Park, Quebrada Ishinca, 09°22'S, 077°25'W, 4400 m, 13 February 1985, *Smith 9535* (MO!, USM!); 09°23'S, 077°27'W, 4100 m, 14 February 1985, *Smith 9568* (AAU!, F!, MO!, USM!). Chavin de Huantar, Cordillera Blanca, Quebrada Pucavada (road between Huaraz and Chavin, on eastern side of pass), 09°40'S, 077°15'W, 4100 m, 16 February 1987, *Boertmann 62* (AAU!); *64* (AAU!). Cusca, Cordillera Blanca, Misquicyucu 20 km east of Yanac towards Sihuas, top of the pass, 08°37'S, 077°45'W, 4000 m, 20 February 1987, *Boertmann 74* (AAU!). Huantar, Quebrada Carhuascancha, Cordillera Blanca, 09°29'S, 077°15'W, 3900 m, 15 August 1988, *Frimer 131* (AAU!); *133* (AAU!); *134* (AAU!). Huaraz, Feria de plantas medicinales de Huaraz, 3052 m, 01 July 1988, *Amaro s.n* (USM!); Quebrada Pitec, 4000 m, 30 October 1989, *Arce 162* (MO!); Llaca, 09°28'52"S, 077°27'42"W, 3909 m, 08 November 2014, *Boza 3021* (USM!, Z!); Rajacolta, 4420 m, 06 June 2015, *Boza 3148*; *3149* (USM!, Z!); Ulta, 4450 m, 07 June 2015, *Boza 3150*; *3151* (USM!, Z!); Quebrada Shallap, Cordillera Blanca, 09°31'S, 077°24'W, 4000 m, 10 August 1988, *Frimer 16*; *17*; *4* (AAU!); Huascarán National Park, Quebrada LLaca, NW-slope of valley, 09°27'S, 077°27'W, 4200–4400 m, 24 May 1985, *Smith 10783* (MO!, USM!); Huascarán National Park, Quebrada Llaca, valley bottom and north side of valley, 09°27'S, 077°27'W, 4090 m, 13 July 1985, *Smith 11143*; *12432* (MO!, USM!); *8973* (AAU!, MO!, USM!); *9696* (MO!, USM!). Huari, 30 km N of Huari, 3960 m, 18 December 1979, *Aronson 993* (F!, MO!); 3000 m, 15 August 1983, *Basauri 3* (USM!); Cahuish, 09°41'30"S, 077°14'30"W, 4306 m, 08 November 2014, *Boza 3017*; *3018* (USM!, Z!); Chavín, antes del Tunel de Cahuish (Cuta Queñua), 3100–3200 m, 11 June 2002, *Cano 12385* (MO!, USM!); 09°33'S, 077°10'W, 3500 m, 14 February 2006, *Sayre 39* (USM!); Huascarán National Park, road to microwave tower passing Manto Mina, off Catac-Chavin road, 09°42'S, 077°15'W, 4400 m, 04 July 1985, *Smith 10974* (MO!, USM!); Huascarán National Park, upper terrace, Quebrada Pachachaca a lateral valley of Quebrada Rurichinchay Bogs, 09°23'S, 077°17'W, 4040–4200 m, 13 June 1986, *Smith 12586* (F!, MO!, USM!); Huascarán National Park. Catac-Chavin road, 44 km from Catac, 09°42'S, 077°13'W, 4200 m, 18 August 1984, *Smith 8287* (MO!, USM!); Huascarán National Park, 0.25 km from Cahuish Tunnel, 09°41'S, 077°14'W, 4450 m, 23 December 1984, *Smith 8752* (F!, MO!, USM!). Huaylas, Laguna Parón, 3860–4000 m, 15 October 1999, *Cano 10023* (USM!); Quebrada Paron, Cordillera Blanca (east of L. Paron), 08°58'S, 077°38'W, 4200 m, 16 August 1988, *Frimer 23*; *24*; *26* (AAU!); Huascarán National Park, Quebrada Parón, 09°01'S, 077°43'W, 3500–3760 m, 08 May 1985, *Smith 10608* (AAU!, F!, MO!, USM!); Huascarán National Park, Parón valley E of lake, 08°59'S, 077°38'W, 4250 m, 27 September 1985, *Smith 11477* (F!, MO!, USM!); Huascarán National Park. Quebrada Santa Cruz at base of and entering Quebrada Artizonraju, 08°55'S, 077°36'W, 4300–4800 m, 16 January 1985, *Smith 9308* (MO!, USM!); Huascarán National Park, environs of Hatuncocha, 08°52'S, 077°45'W, 4600 m, 12 March 1985, *Smith 9982* (AAU!, F!, MO!, USM!); Huascarán National Park. between Huiscash and Mirador, 08°53'S, 077°46'W, 3960–4400 m, 12 March 1985, *Smith 9988* (MO!, USM!). Pomabamba, Pumacocha, 4200 m, 11 December 1989, *Arce 175* (MO!); Pumacocha, 4300 m, 11 December 1989, *Arce 177* (MO!); Quenuarajra, 3600 m, 12 December 1989, *Arce 182* (MO!). San Marcos, San Marcos. Distr. Ccolla Chica, 09°40'28"S, 077°03'10"W, 5600 m, 04 May 2008, *Beltrán 6480* (USM!). Shilla, Quebrada Matará in Quebrada Ulta, Cordillera Blanca, 09°07'S, 077°32'W, 4250 m, 03 September 1988, *Frimer 105*; *106*; *114* (AAU!). Sihuas, Distr. Cashapampa, Pasacancha, 08°35'01"S, 077°39'09"W, 3972 m, 03 February 2011, *Gonzáles 1423* (USM!). Tarica, Quebrada Ishinca, Cordillera Blanca, 09°23'S, 077°28'W, 3950 m, 23 August 1988, *Frimer 72* (AAU!). Yungay, Yanama, 3950 m, 05 November 1989, *Arce 166* (MO!); Llanganuco, fuera del parque Huascarán, 3500 m, 29 May 1981, *Cerrate 7769* (USM!); ca. 25 km NE of Yungay, slopes below Laguna de Llanganuco, 3380 m, 28 January 1983, *Dillon 3117* (F!, MO!, USM!); slopes below Laguna de Llanganuco in quebrada de Llanganuco ca. 25 km above Yungay, 3900 m, 27 June 1966, *Edwin 3829* (F!); Llanganuco, 3700–3800 m, 02 May 1961, *Ferreyra 14377* (GOET!, MO!, USM!); 37 km east of Yungay, 4200 m, 05 April 1988, *Renvoize 5084* (AAU!, MO!); Huascarán National Park, Llanganuco sector, Quebrada Demanda, side valley to Nevado Pisco, 09°01'S, 077°37'W, 4600–4800 m, 13 April 1985, *Smith 10299* (MO!, USM!); *Smith 8794* (MO!, USM!); Huascarán National Park. Quebrada Ranincuray, 08°59'S, 077°34'W, 4000–4300 m, 12 January 1985, *Smith 9139* (AAU!, MO!, USM!). 4000 m, 24 August 1977, *Antunez de Mayolo 330* (MO!, USM!); Cordillera Blanca, East of Yungay Lagunas de Llanganuco lake side, 3800 m, 05 April 1988, *Renvoize 5067* (AAU!); 16 June 1903, *Weberbauer 3287* (B, MO!). **Huánuco**: Huallanca, Huansala 10 km from Huallanca, 09°51'S, 076°56'W, 3700 m, 14 February 1987, *Boertmann 48* (AAU!). **Lima**: Cajatambo, Cerro San Cristobal, 3950 m, 12 March 1990, *Arce 206* (MO!); Raura, 4600 m, 05 April 1988, *Rivas s.n* (USM!). Oyon, Laguna Guengue Grande, Quichas, 4200 m, 11 January 1990, *Arce 190* (MO!); Pueblo Quichas above Oyon, 10°36'S, 076°45'W, 4000 m, 26 February 1987, *Boertmann 81*; *82*; *85*; *87* (AAU!); Oyon, Laguna grande, 10°33'48"S, 076°44'25"W, 4005 m, 24 May 2015, *Boza 3026*; *3047*; *3048*; *3049*; *3050*; *3051*; *3052*; *3053*; *3054*; *3055*; *3056*; *3057* (USM!, Z!); Bosque de Polylepis de Maticuna, 10°39'11"S, 076°50'21"W, 3990 m, 08 August 1998, *Mendoza 173* (CUZ!). Ticlayan, Quichas, Bosque de Polylepis de Q’asacancha, 10°33'S, 076°04'W, 4200 m, 07 July 1998, *Mendoza 170* (CUZ!).

### ﻿Section Australes

Taxon classificationPlantaeRosalesRosaceae

T.Boza & M.Kessler sect.nov.

49BD39C3-A6B2-5CCE-A160-8CDE39F57E95

urn:lsid:ipni.org:names:77301642-1

#### Diagnosis.

Trees, 2–4 lateral leaflet pairs; lower leaflet surfaces glabrous, sparsely hispid or puberulous; fruits with irregular and pronounced wings, glabrous or sparsely hispid.

#### Type.

*Polylepisaustralis* Bitter.

#### Notes.

The sectional epithet *Australes* is a plural adjective agreeing in gender with *Polylepis*. Section Australes contains two species with glabrous leaflet surfaces, 2–4 lateral leaflet pairs and winged fruits. Especially the latter are unique in *Polylepis* and justify placing the two species in a section of its own. Table 6 provides an overview of the arrangement of the taxa by different authors.

**Table 6.** Alignment of the taxa of the Polylepissect.Australes according to Bitter (1911), Simpson (1979), Segovia et al. (2018) and the present study.

Bitter (1911)	Simpson (1979)	Segovia et al. (2018)	This study
*P.australis*	*P.australis*	*P.australis*	*P.australis*
—	—	*P.neglecta*	*P.neglecta*

##### Climatic niches in Polylepissect.Australes

**Figure 62.**
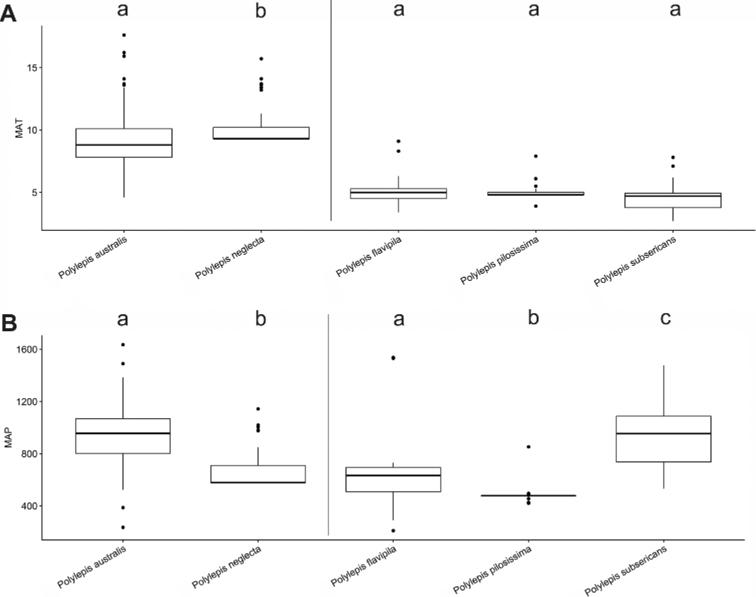
Box plots showing the climatic niches of the species of the subsections *Australes* and *Subsericantes* in relation to MAT (**A**) and MAP (**B**). See Fig. 12 for details on data presentation.

The climatic niches of the two species are quite similar, but *P.australis* grows in slightly warmer and substantially wetter areas than *P.neglecta* (Fig. 62). In addition, being the southernmost species of the genus, *P.australis* grows in a strongly seasonal climate with warm summers and cold winters, which is unique in the genus.

### ﻿23. Polylepis australis

Taxon classificationPlantaeRosalesRosaceae

Bitter, Bot. Jahrb. Syst. 45: 619. 1911.

5F18B78E-919C-5ADB-84C1-074874496E3C

Figs 63 , 64

Polylepis australis var. crenulata Bitter, Bot. Jahrb. Syst. 45: 625. 1911. Type. Based on Polylepisaustralis Bitter.
Polylepis australis var. glabra (O. Kuntze) Bitter, Bot. Jahrb. Syst. 45: 622. 1911.
Polylepis racemosa var. glabra O. Kuntze, Revis. Gen. Pl. 3(1–3): 77. 1898. Basionym. Type. Argentina. Córdoba: Sierra de Córdoba, *Schnyder 483* (holotype: B destroyed).
Polylepis australis var. glabrescens (O. Kuntze) Bitter, Bot. Jahrb. Syst. 45: 623. 1911.
Polylepis racemosa var. glabrescens O. Kuntze, Gen. Pl. 3(1–3): 77. 1898. Basionym. Type. Argentina. Catamarca: Cienaga, *Lorentz 310* (holotype: B destroyed).
Polylepis australis var. subcalva Bitter, Bot. Jahrb. Syst. 45: 623. 1911. Type. based on Polylepisaustralisvar.glabrescens (O. Kuntze) Bitter
Polylepis australis var. oblanceolata Bitter, Bot. Jahrb. Syst. 45: 623. 1911.
Polylepis racemosa var. pubescens O. Kuntze, Gen. Pl. 3(1–3): 77. 1898. Basionym. Type. Argentina. Córdoba: Sierra Achala, Cuesta de Copina, *Hieronymus s.n* (holotype: B destroyed).
Polylepis racemosa var. subresinosa O. Kuntze, Gen. Pl. 3(1–3): 77. 1898. Type. Argentina. Córdoba: Sierra Achala, Cuesta de Copina, *Hieronymus s.n* (holotype: B destroyed).
Polylepis racemosa Lar. *pubinervia* O. Kuntze, Gen. Pl. 3(1–3): 77. 1898. Type. Argentina. Cerro Champaqui, *F. Kurtz s.n* (holotype: B destroyed).
Polylepis australis var. fuscitomentella (O. Kuntze) Bitter, Bot. Jahrb. Syst. 45: 625. 1911.
Polylepis racemosa var. fuscotomentella O. Kuntze, Gen. Pl. 3(1–3): 77. 1898. Basionym. Type. Argentina. Oyada: *Lorentz 442* (holotype: B destroyed).
Polylepis australis var. latifoliolata Bitter, Bot. Jahrb. Syst. 45: 624. 1911. Type. Argentina. Córdoba: Sierra Achala, north of the Cuesta de Copina, *Hieronymus s.n* (holotype: B destroyed).
Polylepis australis var. tucumanica Bitter, Repert. Spec. Nov. Regni Veg. 12: 478. 1913. Type. Argentina. Tucumán: Cañada del Muñoz, Tafi del Valle, Jan 1912, *Castillon s.n* (holotype: B destroyed; isotypes: CORD!, GOET!).
Polylepis australis var. tucumanica subvar. latifrons Bitter, Repert. Spec. Nov. Regni Veg. 12: 479. 1913. Type. Based on Polylepisaustralis var. *tucumánica* Bitter.
Polylepis australis var. tucumanica subvar. majuscula Bitter, Repert. Spec. Nov. Regni Veg. 12: 478. 1913. Type. Argentina. Tucumán: La Queñoa, 2600 m, 11 Mar 1912, *Lillo 11257* (holotype: B destroyed; isotypes: CORD!, GOET!).
Polylepis australis var. tucumanica subvar. gracilescens Bitter, Repert. Spec. Nov. Regni Veg. 12: 479. 1913. Type. Argentina. Tucumán: Angostura, Tafi del Valle, *Castillon s.n* (holotype: B destroyed; isotypes: CORD!, GOET!, US!).
Polylepis australis var. tucumanica subvar. breviuscula Bitter, Repert. Spec. Nov. Regni Veg. 12: 479. 1913. Type. Argentina. Tucumán: La Cienaga, 2500 m, 19 Dec 1908, *Lillo 8767* (holotype: B destroyed; isotypes: CORD!, GOET!).

#### Type.

Argentina. Jujuy: Sierra Santa Barbara, 2500 m, 11 Ju1 1901, *Fries 264* (lectotype designated by Simpson 1979, pg. 56: S!; isolectotype: US!).

**Figure 63.**
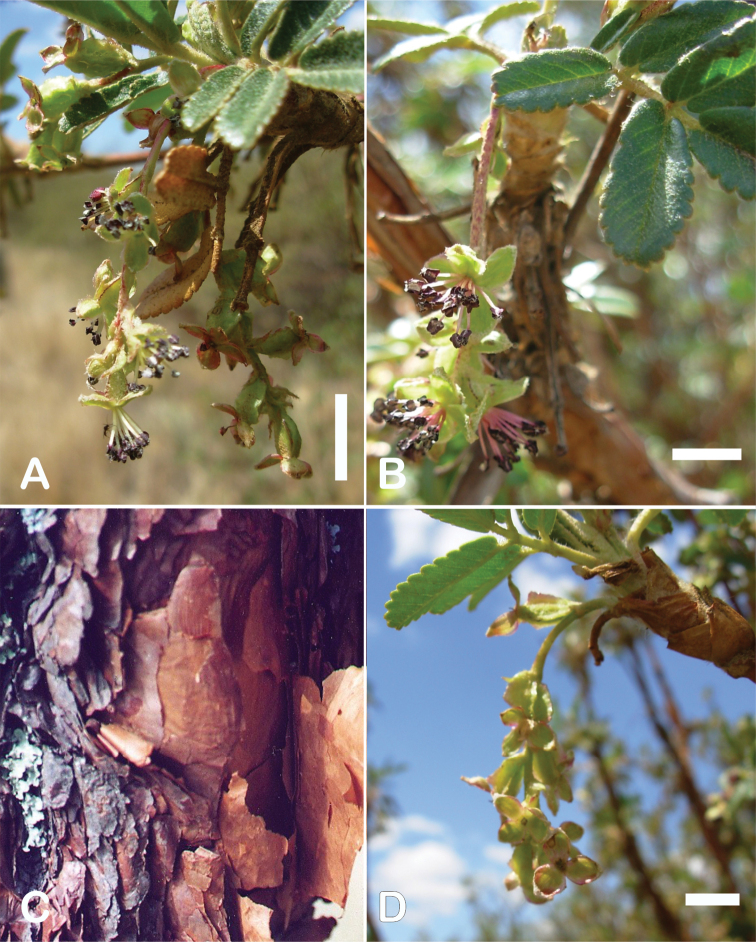
*Polylepisaustralis* Bitter **A** flowering and fruiting branches **B** flowers **C** bark **D** fruiting branch. Scale bars: 1 cm (**A, B**); 5 mm (**D**). Photographs by A. Fuentes.

#### Description.

***Trees*** 3–8 m tall. ***Leaves*** slightly congested at the branch tips, imparipinnate with 2–3 pairs of leaflets, obtrullate in outline, (2.0–)3.7–6.1(–7.2) × (1.8–)2.9–4.1(–5.3) cm; rachises sparsely hispid, points of leaflet attachment with a tuft of long white hairs; stipular sheaths glabrescent or sparsely to densely villous with long hairs; leaflets elliptic in outline, second pair from the terminal leaflet the largest, one of this pair (1.0–)1.6–2.9(–4.0) × 0.6–1.5 cm; margin serrate with 9–13 teeth, apically emarginate, basally unequally cordate; upper leaflet surfaces glabrous; lower leaflet surfaces glabrous or sparsely hispid. ***Inflorescences*** pendant, (1.8–)4.2–5.0(–7.3) cm long, bearing 5–12 flowers; floral bracts 2.1–3.1 mm long, narrowly triangular; rachises villous. ***Flowers*** 4.4–8.5 mm diam.; sepals 4, ovate, green, densely villous outside; stamens 10–22, anthers orbicular, with a dense tuft of straight white hairs on the upper half; styles fimbriate, 0.9–2.0 mm long. ***Fruits*** turbinate, with 2–3 irregular and pronounced thin wings, glabrous; 4.4–7.9 × 5.0–6.8 mm including wings. ***Diploid***, ***triploid***, ***tetraploid*** and ***hexaploid***.

**Figure 64.**
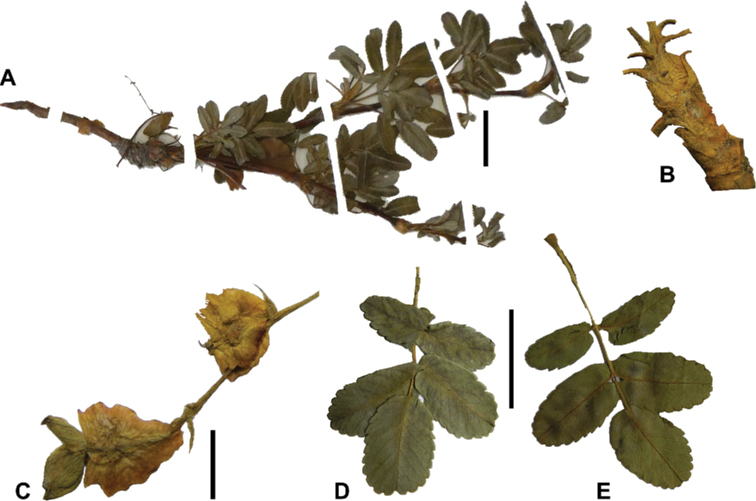
*Polylepisaustralis* Bitter **A** flowering branch **B** stipular sheaths **C** fruits **D** upper leaf surface **E** lower leaf surface (**A***Novara 6695***B–D***Kessler 3350***E***Kessler3348*). Scale bars: 6 cm (**A**); 5 mm (**B**); 2 cm (**D, E**). Photographs by E.G. Urquiaga F.

#### Distribution, habitat and ecology.

*Polylepisaustralis* is endemic to Argentina, where it is distributed from the northern Andes (Jujuy Province) to the central Sierra de Córdoba (Córdoba Province) Argentina (Fig. 73). It occurs in humid subtropical mountains, as well as in dry forest at 1230–3800 m elevation. The northernmost populations of *P.australis* have higher genetic differentiation and lower genetic population diversity than southern and central populations (Hensen et al. 2011). Possibly, gene flow in the northern stands is restricted by geographic isolation, whereas the southern and central populations may be connected by effective long-distance pollination (Hensen et al. 2011). Dendroclimatic analyses from central Argentina show that growth of *P.australis* at intermediate and high elevations growth is more strongly related to temperature than to rainfall (Lanza et al. 2018). Populations at the upper and lower elevational limits of the species are adapted to the respective climatic conditions, which should be considered in conservation action (Marcora et al. 2021). In many regions, *P.australis* is confined to areas with high rock proportion, to where they have been restricted due to fires and long-term grazing pressure (Suarez et al. 2008). *Polylepisaustralis* forms shrublands on ridges with poor, leached soils exposed to wind and frost and woodlands in sheltered ravines. The most critical stage of the species regeneration cycle in the shrublands is the transition from seedling to saplings, whereas in the woodlands, it is seed germination (Enrico et al. 2004). Overall, seed germination rates in *P.australis* are low at 0% to 14% (Enrico et al. 2004; Renison et al. 2004; Menoyo et al. 2009; Pollice et al. 2013). Germination rates are higher in areas with reduced livestock density which have lower insect predation and fungal fructifications (Menoyo et al. 2009). Seed production increases with tree height, with highest seed production in areas without livestock. Seed mass also increases with tree height, but there is no livestock effect (Pollice et al. 2013). Seed viability is associated with relatively undisturbed soils with no erosion, suggesting a connection to nutrients and/or water stress (Renison et al. 2004). Maximum seed dispersal distance in *P.australis* is 6 m and seedlings are found no more than 10 m from seed trees (Torres et al. 2008). However, extensive pollen flow between isolated fragments reduces the negative effects of seed quality resulting from reproductive isolation and inbreeding (Seltmann et al. 2009b). Seedling establishment is severely affected by livestock, so that there are more seedlings and seed trees in areas with less livestock than in degraded regions with historic and current grazing impact (Renison et al. 2006; Torres et al. 2008; Menoyo et al. 2009; Marcora et al. 2013). Livestock has considerable impact on soil degradation in the mountains of central Argentina (Renison et al. 2010), and these vegetation-soil alterations reduce the soil water storage capacity of *P.australis* woodlands (Poca et al. 2018). *Polylepis* forest in central Argentina have high polypore (wood-decay fungi) species richness in mature forest, with numerous threatened and rare species (*Postiacaesia*, *Fuscoporiagilva*, *Polyporusarcularius* and *Ceriporiaspissa*) found in the presence of large logs (Robledo and Renison 2010). Similarly, there is a high diversity of lichens (Rodriguez et al. 2020). In the southern Yungas (Jujuy Province), the *P.australis* forests have high bird diversity, but *Polylepis* specialist bird species are absent, possibly because these are small patches (Bellis et al. 2014). However, active forest restoration measures have locally increased bird diversity and abundance over 20 years (Barri et al. 2021).

#### Conservation status.

The EOO for *Polylepisaustralis* is estimated as 151,750 km^2^, the AOO is assessed at 604 km^2^ and it is known from 73 locations. *Polylepisaustralis* has the most extensive forest in the southern sector of the Quebrada del Condorito National Park and surrounding private fields in Córdoba Province. The best-preserved stands are located near the town of Los Molles at 1500–2650 m (Renison et al. 2013). In many stands of *P.australis*, there is evidence of fire that is set to induce grass regrowth as feed for livestock (Renison et al. 2002, 2006, 2013; Argibay and Renison 2018). Fire, logging and browsing affect *P.australis* forests and a reduction of such disturbances would increase the area covered by these forests and the vitality of the trees (Renison et al. 2011, 2013; Cáceres et al. 2021). However, moderate livestock densities are compatible with forest conservation, if properly managed (Giorgis et al. 2020). *Polylepisaustralis* is also affected by the invasion of exotic woody species, such as *Pinus* sp. planted in large areas in Córdoba Province, as well as other species like *Cotoneasterfranchetii*, *Betulapendula*, *Rosarubiginosa*, *Rubusulmifolius*, *Salixviminalis* and S.aff.fragilis (Renison et al. 2013). Vegetation cover is very low in *P.australis* forests, so that it is common to observe bare roots as an evidence of soil erosion (Cingolani et al. 2008; Renison et al. 2010, 2013). Nevertheless, *P.australis* can regenerate even under such conditions, possibly due to the long history of fire in the region (Cingolani et al. 2014; Torres and Renison 2017). *Polylepisaustralis* has been subject to reforestation activities in Córdoba since 2002 (Renison et al. 2002; Aráoz and Grau 2010; Renison et al. 2013). Seedling growth is six times slower in sowing experiments than the growth of planted *P.australis* (Landi and Renison 2010). Thus, planting and not seeding has become the preferred method to re-establish this species. In addition, the construction of terraces with *Poastuckertii* as a nursery plant has been suggested. Moreover, biparental inbreeding depression is found especially in plants that cross with nearby neighbours (Seltmann et al. 2009a). This is more important to progeny fitness and mortality than to germination. Additionally, crosses between fragments have higher reproductive output than within-fragment crosses. We assess *P.australis* as Least Concern (B1a+B2a).

#### Notes.

*Polylepisaustralis* is easy to recognize by its glabrous leaflets (sometimes the lower leaflet surface can be sparsely hispid) and by its winged fruits, a character shared only with *P.neglecta*. Specimens of *P.australis* can resemble those of *P.neglecta* in leaflet shape, size and margin, but leaflet apices are emarginate in *P.australis* and acute in *P.neglecta*. Further, *P.australis* differs from *P.neglecta* by it simple and villous inflorescences with 5–12 flowers, whereas *P.neglecta* usually has branched and glabrous inflorescences with 14–27 flowers.

*Polylepisaustralis* is morphologically quite variable, as evidenced by the large number of varieties recognized by Bitter (1911). More recent studies have shown that *P.australis* also has high variability of ploidy levels (Schmidt-Lebuhn et al. 2010; Kessler et al. 2014). In a study of 361 individuals, Kessler et al. (2014) found that, in the Andean part of the distributional range of the species, most populations are purely diploid (except at the southernmost tip of the Andean range), whereas in the isolated Sierra de Córdoba, tetraploids dominate, but there are also diploid and triploid plants and even a single hexaploid plant was found. This diversity of ploidy levels raises several important questions. First, the degree to which the di- and tetraploids are reproductively isolated and might, therefore, be treated as distinct taxa, is unknown. Soltis et al. (2007) have proposed that populations of a species with different ploidy levels should be treated as distinct species if there is evidence of reproductive isolation or if they are morphologically or ecologically distinct. In the case of *P.australis*, the different geographical ranges of the two main ploidy levels suggest that they have independent evolutionary trajectories. If this is confirmed, since the type collection of the species comes from the purely diploid part of the range, the unnamed form would be the tetraploid one. Following Soltis et al. (2007), a suitable name would be *Polylepistetra-australis*. Second, it is unknown whether the triploid individuals are only first-generation hybrids between di- and tetraploids or are also able to reproduce by apomixis, as is common in many Rosaceae. Indeed, the low rate of seed germination of many individuals (Enrico et al. 2004; Renison et al. 2004; Menoyo et al. 2009) may be linked to the triploid ploidy level, but this also remains to be explored.

#### Specimens examined.

**Argentina. Catamarca**: Andalgalá, East slope of Nevados de Aconquija, Estancia “Yunca Suma”, Los Queñoales, 27°22'S, 066°02'W, 2400 m, 20 February 1966, *Hawkes 3547* (MO!). **Córdoba**: Calamuchita, Sierra Grande (Falda E), al pie del Cerro Champaquí, 2200 m, 26 September 1952, *Hunziker 9942* (MO!); La Cumbrecita, 31°55'S, 064°15'W, 1450 m, 17 December 1978, *Solomon 4200* (MO!). Punilla, Dpto. Punilla, cerca del río Yatain, 18 November 1971, *Ancibor 2150* (MO!); Tanti, Ruta n° 20, km 757, 26 April 1963, *Ariza 1604b* (CORD); Cerro Los gigantes, 50 km west of Córdoba, 2000–2050 m, 27 January 1974, *Conrad 2455* (MO!); Barranca de Río Yuspe (Puente), 1780 m, 16 December 1949, *Meyer 15627* (GOET!). San Alberto, Pampa de Achala, 2200 m, 13 December 1945, *Hunziker 1398* (MO!); Pampa de Achala, ca. 40 km E Mina Clavero on road to Córdoba, 1900 m, 13 November 1991, *Kessler 3347* (AAU!, GOET!, MO!); *3348* (GOET!, MO!); *3349*; *3350* (AAU!). **Jujuy**: Capital, Lagunas de Yala, 2100–2300 m, 18 November 1986, *Charpin AC 20520* (MO!). Jujuy, Jala-Reyes, 30 October 1982, *Zardini 1587* (MO!). Sierra La Barbara, 2500 m, 11 July 2001, *Fries 264* (US!). Valle Grande, Camino a Altos de Calilegua, 2400 m, 31 October 1974, *Cabrera 25659* (MO!). **Salta**: Guachipas, Alto del Poronguito, 1900–2000 m, 07 February 1983, *Novara 3157* (MO!). Santa Victoria, 15 km. from Santa Victoria towards La Quiaca, 22°15'S, 065°04'W, 3300–3400 m, 05 April 1979, *Bothmer 6466* (MO!); Santa Victoria, ruta 5, alturas 10 km al W del pueblo, 2900 m, 14 May 1987, *Novara 6695* (GOET!, Z!); Santa Victoria, por el camino 5–10 km al W del pueblo, 2700–3000 m, 10 November 1988, *Novara 8210* (Z!); Santa Victoria Oeste, 22°15'00"S, 064°58'00"W, 21 January 1983, *Zardini 1676* (MO!); La Huerta, 31 January 1983, *Zardini 1903* (MO!); **San Luis**: Junín, Sierra de Comechingones, al este de Merlo, ruta prov. 5, 1200 m, 16 December 2000, *Leuenberger 4764* (GOET!); 5 km al este de Merlo (El Mirador), 26 January 2001, *Scarpa 435* (SI). **Tucumán**: Chicligasta, Saladillo, 1000 m, 18 May 1948, *Meyer 14063* (MO!); 1800 m, 10 March 1924, *Venturi 3010* (Z!); 2000 m, 13 December 1925, *Venturi 3990* (MO!). Tafí, Quebrada de los Alisos Tafi del Valle, 01 January 2012, *Castillón 89344* (US!); Tafí del Valle, 2000 m, 24 September 1949, *Palacios 19Ar106* (MO!); Tafí del Valle, 26°47'28"S, 065°43'48"W, s.d., *Palacios s.n* (MO!); Localidad La Cienaga, 2800 m, 29 January 1950, *Sleumer 204* (GOET!). Trancas, 01 August 2017, *Schreiter 321* (MO!); Tafi del Valle, 19 December 1965, *Walter 614* (GOET!).

### ﻿24. Polylepis neglecta

Taxon classificationPlantaeRosalesRosaceae

M. Kessler, Candollea 50(1): 140. 1995.

94018C15-F67B-5189-ADE8-9CE06264C321

Figs 65 , 66

#### Type.

Bolivia. Potosi: Prov. Bilbao, 31 km SW Acacio on road to Sacaca and Uncia, 18°06'S, 66°08'W, 3500 m, 22 Aug 1991, *Kessler 3410* (holotype: LPB!; isotypes: AUU!, F!, G!, GOET!, MO!, NY, US!, USM!).

**Figure 65.**
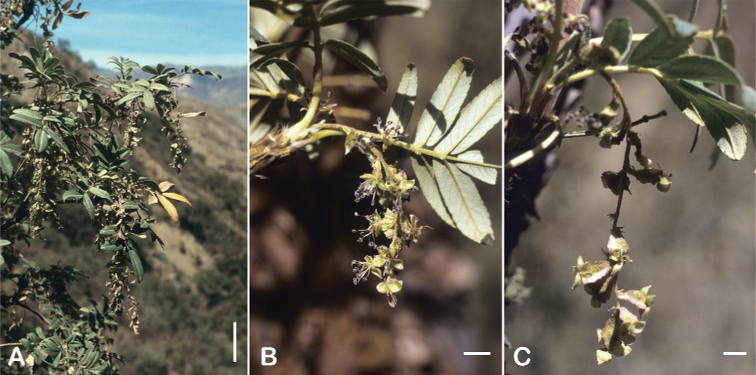
*Polylepisneglecta* M.Kessler **A** flowering branches **B** inflorescence and lower leaf surface **C** fruiting branch. Scale bars: 3 cm (**A**); 5 mm (**B, C**). Photographs by M. Kessler.

#### Description.

***Trees*** 4–12 m tall. ***Leaves*** slightly congested at the branch tips, imparipinnate with 3–4 pairs of leaflets, obtrullate in outline, (3.9–)4.6–7.1(–8.5) × (2.7–)3.2–5.3 cm; rachises glabrescent to sparsely hispid, points of leaflet attachment with a tuft of long, white hairs; stipular sheaths apically acute with spurs, glabrescent to sparsely hispid on the outer surfaces; leaflets elliptic in outline, second pair from the terminal leaflet the largest, one of this pair 1.6–2.9 × 0.6–1.2 cm; margin serrate with 12–18 teeth, apically acute, basally unequally cordate; upper leaflet surfaces glabrous; lower leaflet surfaces glabrous or puberulous. ***Inflorescences*** branched, pendant, (4.3–)5.2–12.7(–14.0) cm long, bearing 14–27 flowers; floral bracts 2.8–4.6 mm long, narrowly triangular, glabrous to sparsely hispid on the outer surface; rachises glabrous. ***Flowers*** 4.6–6.3 mm diam.; sepals 4, ovate, green, sparsely hispid outside; stamens 7–20, anthers orbicular, with a dense tuft of straight white hairs on the upper half; styles fimbriate, 1.5–2.2 mm long. ***Fruits*** turbinate, with 2–3(–4) irregular and pronounced wings, glabrous or sparsely hispid; 4.8–9.8 × 3.4–5.5 mm including spines. ***Diploid*** and ***hexaploid***.

**Figure 66.**
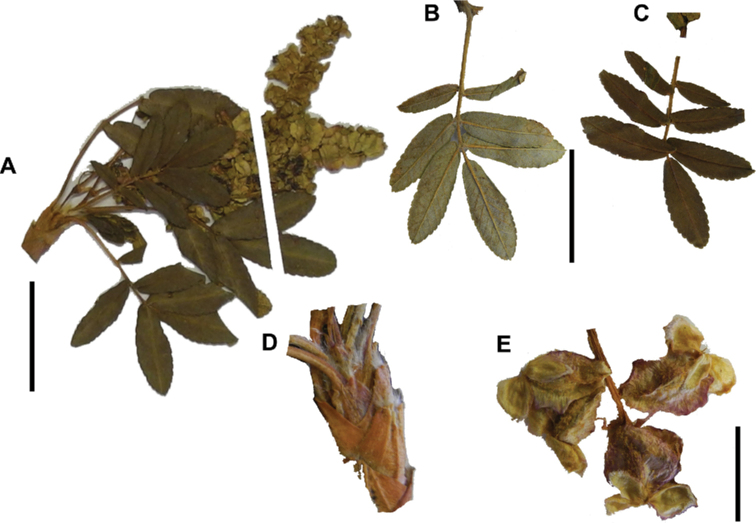
*Polylepisneglecta* M.Kessler **A** flowering branch **B** lower leaf surface **C** upper leaf surface **D** stipular sheaths **E** fruits (**A***Kessler 3533***B***Kessler 3536***C***Kessler 3628***D***Kessler 3634***E***Kessler 3019*). Scale bars: 4 cm (**A**); 3 cm (**B**); 9 mm (**E**). Photographs by T. E. Boza E.

#### Distribution, habitat and ecology.

*Polylepisneglecta* is endemic to central Bolivia. It occurs in the Boliviano-Tucumanic Forest at 2100–3700 m elevation (Fig. 73). It often occurs together with *P.hieronymi* and *P.besseri* or in mixed forest with *Podocarpusparlatorei*, *Alnusacuminata*, *Juglansaustralis*, *Azarasalicifolia*, *Fagaracoco*, *Prunustucumanensis*, *Schinusmicrophyllus* and *Escalloniamillegrana* (Kessler 1995b; Navarro and Maldonado 2002; Navarro et al. 2005; Gareca et al. 2010). In *P.neglecta*, a germination rate of 10% has been recorded (Vega et al. 2018). The seeds have low humidity, being able to be treated as orthodox seeds with the capability of being stored for a long time without losing their germinative power (Vega et al. 2018).

#### Conservation status.

The EOO for *Polylepisneglecta* is estimated as 24,431 km^2^, the AOO is assessed at 44 km^2^ and it is known from nine locations. The species was categorized as VU (A1acd, B1+2c) by Oldfield et al. (1998). Later, based on its severely fragmented habitat and the constant deterioration by collection of firewood for charcoal and fire regimens, it was listed as VU (B1ab(ii,iii)) in the Red List of Bolivia (Arrázola and Coronado 2012). It does not occur in any protected area and the agricultural expansion, logging and fire regime are reducing populations. Based on its fragmented and degraded distribution and the lack of habitat protection, we assess *P.neglecta* as Vulnerable (A1,2a, B1a+B2a, C2a).

#### Notes.

For morphological similarities, see under *P.hieronymi* and *P.australis*.

#### Specimens examined.

**Bolivia. Chuquisaca**: Azurduy, ca. 20 km NW Tarvita on road to Tarabuco, 19°54'S, 064°34'W, 2900 m, 24 September 1991, *Kessler 3434* (AAU!, GOET!); ca. 20 km NW Tarvita on road to Tarabuco, 19°54'S, 064°34'W, 2900 m, 24 September 1991, *Kessler 3435* (GOET!); *3436*; *3437* (AAU!, GOET!); *3625*; *3626*; *3627*; *3628*; *3629*; *3630*; *3631*; *3633*; *3634*; *3635*; *3663*; *3664*; *3665*; *3666*; *3667*; *3668* (GOET!). Belisario Boeto, trayecto Villa Serrano hacia la comunidad de Tampa Mayu y Nuevo Mundo hacia el Cuenca del Río Grande, 19°00'04"S, 064°18'55"W, 1250 m, 12 October 2007, *Cervantes 164* (HSB, MO!); 8 km SW Nuevo Mundo on road to Padilla, 19°25'S, 064°11'W, 2500 m, 07 October 1991, *Kessler 3316* (AAU!, GOET!); 18 km de Villa Serrano hacia Valle Grande, 18°57'S, 064°20'W, 2500 m, 27 August 1994, *Moraes 1840* (LPB); 18 m by road NE of Villa Serrano on road to Nuevo Mundo, 19°00'42"S, 064°20'04"W, 2550 m, 20 July 2004, *Nee 52789* (MO!, NY, USZ); Municipio de Villa Serrano, Comunidad de Ovejeros, 19°01'32"S, 064°20'20"W, 2489 m, 18 December 2003, *Portal 618* (HSB, MO!). Tomina, Padilla 26 kms hacia Monteagudo, 2600 m, 08 March 1981, *Beck 6323* (GOET!); ca. 20 km SE Padilla on road to Monteagudo, 19°03'S, 064°16'W, 2450 m, 07 October 1991, *Kessler 3320* (GOET!). **Cochabamba**: Mizque, 17°49'S, 065°26'W, 3200 m, 05 May 1987, *Estenssoro 802* (LPB). s.d., *Saravia 1165* (BOLV). **Potosí**: Alonso de Ibanez, antes de llegar a Chiro Q’asa, 18 March 1993, *Torrico 198* (GOET!); *199* (GOET!). Gral. Bilbao, 31 km SW de Acacio, camino Sacaca y Uncia, 18°06'S, 066°08'W, 3500 m, 22 August 1991, *Kessler 3019* (GOET!, LPB, MO!); 31 km SW Acacio on road to Sacaca and Uncia, 18°06'S, 066°08'W, 3500 m, 22 August 1991, *Kessler 3411* (AAU!, GOET!); *3522*; *3523*; *3524*; *3525*; *3526*; *3528*; *3529*; *3531*; *3532*; *3533*; *3534*; *3535*; *3536*; *3537*; *3538*; *3539*; *3540*; *3541*; *3542* ; *3543*; *3544*; *3545*; *3546*; *3547*; *3548*; *3550*; *3551*; *3552*; *3555*; *3647* (GOET!). San Pedro de Buena Vista, Bilbao 31 km. SW Acacio on road to Sacaca and Uncia, 18°06'S, 066°08'W, 3500 m, 22 August 1991, *Kessler 3410* (AAU!, G, GOET!, LPB, MO!, NY, US!, USM!). **Santa Cruz**: Comarapa, about 25 km from Pojo, the road Cochabamba–Santa Cruz about 215 km from Cochabamba, 17°50'S, 064°40'W, 2950 m, 21 April 1987, *Brandbyge 717* (AAU!). Manuel Maria Caballero, ca. 55 km W Comarapa on road to Cochabmaba, 17°50'S, 064°42'W, 2600 m, 06 October 1991, *Kessler 3302* (AAU!); ca. 50 km W Comarapa on road to Cochabamba, 17°50'S, 064°41'W, 2800 m, 06 October 1991, *Kessler 3303* (GOET!); Siberia, alrrededores de la escuela y tramo de 2 km al S bajando por el camino a Oconi. Ladera SSW, 17°50'S, 064°45'W, 25 July 2000, *Vargas 4975* (GOET!, NY, USZ).

### ﻿Section Subsericantes

Taxon classificationPlantaeRosalesRosaceae

T.Boza & M.Kessler sect. nov.

2176BDF2-1FFD-5A49-998A-F23B92E968C2

urn:lsid:ipni.org:names:77301643-1

#### Diagnosis.

Trees; 1 lateral leaflet pair; lower leaflet surfaces densely villous or strigose; fruits with 3–4 irregular flattened ridges with a series of spines, densely pilose.

#### Type.

*Polylepissubsericans* J.F. Macbr.

#### Notes.

The sectional epithet *Subsericantes* is a plural adjective agreeing in gender with *Polylepis*. Section Subsericantes contains species with pilose or strigose lower leaflet surfaces, one lateral leaflet pair and densely pilose fruits with 3–4 irregular flattened ridges bearing a series of spines. This peculiar combination of characters, which combines traits of sections *Sericeae* and *Incanaee*, was already noted by Simpson (1979). Schmidt-Lebuhn et al. (2006a) recovered the species *P.subsericans* as sister to those here placed in section Incanaee. Given their distinctness, we place this species and two closely related ones in a separate section. All three species only occur in central Peru, where they occupy parapatric ranges. Table 7 provides an overview of the arrangement of the taxa by different authors.

**Table 7.** Alignment of the taxa of the Polylepissect.Subsericantes according to Bitter (1911), Simpson (1979), Segovia et al. (2018) and the present study.

Bitter (1911)	Simpson (1979)	Segovia et al. (2018)	This study
*P.subsericans*	*P.subsericans*	*P.subsericans*	*P.subsericans*
*P.incana*		*P.flavipila*	*P.flavipila*
			*P.pilosissima*

##### Climatic niches in Polylepissect.Subsericantes

Climatic niches among the species of this section do not differ with respect to the temperature under which they grow, but have clear differences with respect to mean annual rainfall: *Polylepissubsericans* grows under the most humid conditions, *P.flavipila* has an intermediate position and *P.pilosissima* grows under the most arid conditions (Fig. 62).

### ﻿25. Polylepis flavipila

Taxon classificationPlantaeRosalesRosaceae

(Bitter) M.Kessler & Schmidt-Leb., Organisms Diversity Evol. 6(1): 69. 2006.

FAEBC8D3-0778-5DC3-A885-FC5A266CCD7C

Figs 67 , 68

#### Basionym.

Polylepisincanavar.flavipila Bitter., Bot. Jahrb. Syst. 45: 640. 1911.

**Figure 67.**
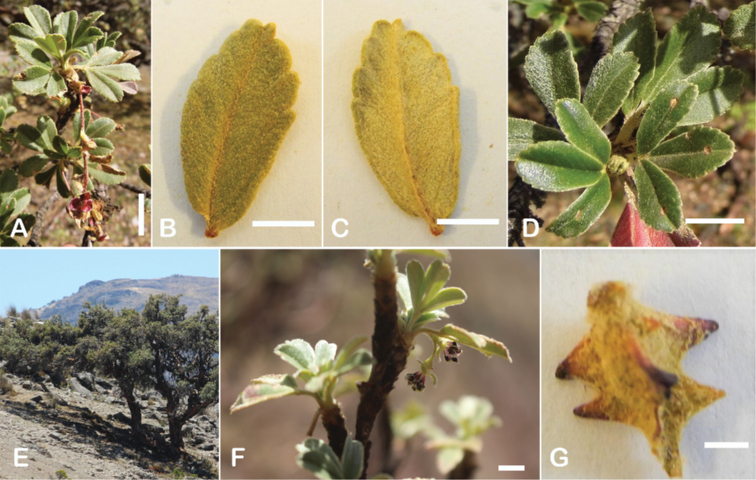
*Polylepisflavipila* (Bitter) M.Kessler & Schmidt-Leb **A** flowering branch **B** upper leaf surface **C** lower leaf surface **D** leaves **E** habit **F** flowers **G** fruit (**A, D***Boza et al. 3157***B, C, G***Quispe 79*). Scale bars: 1 cm (**A, D**); 5 mm (**B, C, F**); 1 mm (**G**). Photographs **A, D** G. Vargas **B, C, G** T.E. Boza E. **E, F** H.R. Quispe.

#### Type.

Peru. Huancavelica: Castro-Virreyna, western slopes of the Andes between Pisco and Ayacucho, 3900–4000 m, May 1910, *Weberbauer 5433* (holotype: B destroyed; isotypes: F!, GH!, GOET!, US!).

**Figure 68.**
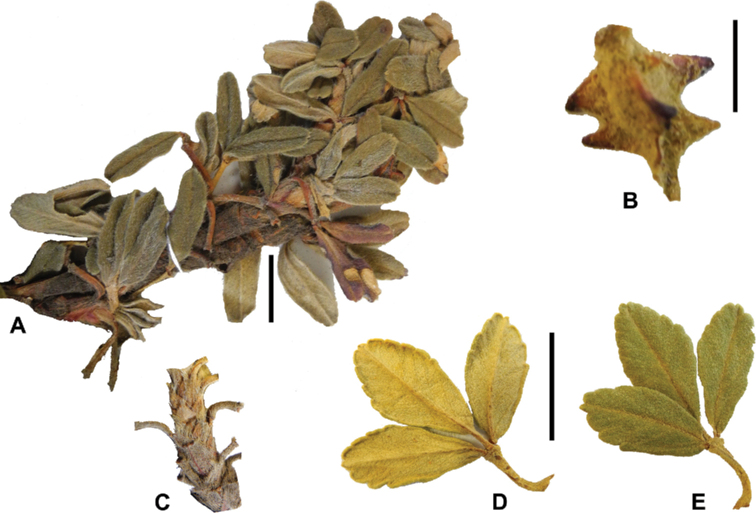
*Polylepisflavipila* (Bitter) M. Kessler **A** flowering branch **B** fruit **C** stipular sheaths **D** lower leaf surface **E** upper leaf surface (**A***Beltran 6391***B–E***Boza 3160*). Scale bars: 2 cm (**A, D, E**); 3 mm (**B**). Photographs by T. E. Boza E.

#### Description.

***Trees*** 3–8 m tall. ***Leaves*** slightly congested at the branch tips, imparipinnate with one pair of leaflets, obtrullate in outline, (1.9–)2.3–2.8 × 1.9–2.2 cm; rachises slightly villous, points of leaflet attachment with a tuft of long hairs; stipular sheaths not spurred, densely villous on the outer surfaces; leaflets obovate in outline, second pair from the terminal leaflet the largest, one of this pair (1.2–)1.6–2.0 × 0.6–0.8 cm; margin crenate with 4–6 teeth, apically acute or emarginate, basally cuneate; upper leaflet surfaces sparsely pilose; lower leaflet surfaces densely pilose with yellowish hairs 0.5–0.6 mm long. ***Inflorescences*** pendant, (2.7–)3.2–4.4 cm long, bearing 3–5 flowers; floral bracts 3.4–3.9 mm long, narrowly triangular, densely pilose on the outer surface; rachises pilose. ***Flowers*** 4.8–8.4 mm diam.; sepals 3–4, ovate, green to reddish, densely pilose outside; stamens 11–13, anthers orbicular, with a dense tuft of straight white hairs on the upper half; styles fimbriate, 2.4–3.2 mm long. ***Fruits*** turbinate, with 3–4 irregular flattened ridges with a series of spines, densely pilose; 4.1–5.2 × 2.4–4.2 mm including spines. ***Tetraploid***.

#### Distribution, habitat and ecology.

*Polylepisflavipila* is distributed on the western Andean slope of central Peru in the Departments of Lima, Huancavelica and Ayacucho (Fig. 73). It grows in relatively dry and cold areas at 3300–4660 m elevation. In Nor-Yauyos Cochas (Lima), forests of *P.flavipila* have high floristic diversity, with 282 vascular plant species recorded, including 41 species endemics to Peru and 13 species categorized as threatened in Peru (Trinidad and Cano 2016). The structure of *P.flavipila* forests is variable and depends on soil fertility and water availability (Camel et al. 2019b). The water potential of the trees is influenced by elevation and soil factors (Yaranga et al. 2021). An important factor that affects *P.flavipila* is the presence of the hemiparasite *Tristerixchodatianus*, which increases the mortality of the trees. The parasite damages the branches of the trees by causing water stress, forcing the host tree to increase its hydraulic conductivity which increases its vulnerability to drought and, about 15 years after colonization, leading to the death of the host branch (Camel et al. 2019a). If many branches are affected, ultimately the whole tree may die.

#### Conservation status.

The EOO for *Polylepisflavipila* is estimated as 21,371 km^2^, the AOO is assessed at 132 km^2^, and it is known from 22 locations. It was categorized as VU (A1acd, B1+2c) in the World List of Threatened Trees (Oldfield et al. 1998, as *P.subsericans*) and as VU B1ab(iii) in the Red List of Threatened Flora of Peru (Mendoza and León 2006). It is protected within the Nor Yauyos-Cocha Landscape Reserve. However, its populations are fragmented and severely threatened by overgrazing and logging and forest cover has been reduced by 53% between 1975 and 2020, based on satellite images (Ames-Martínez et al. 2021). We assess *P.flavipila* as Endangered (B1a+B2a, C2a).

#### Notes.

This species was described by Bitter (1911) as a variety of *P.incana* and was placed in synonymy with *P.subsericans* by Simpson (1979), but elevated to species rank by Kessler and Schmidt-Lebuhn (2006) due to its morphological distinctness. *Polylepisflavipila* differs from *P.subsericans* by leaflet shape and margin (obovate with crenate margin versus narrowly elliptic with entire to slightly serrate margin) and different types of hairs (pilose versus strigose).

#### Specimens examined.

**Peru. Ayacucho**: Huamanga, Vinchos, 13°20'55"S, 074°27'28"W, 3100–3600 m, 29 September 2003, *Mendoza & Roque 991* (MO!). **Huancavelica**: Castrovirreyna, Chaupipata, 11°29'34"S, 074°56'37"W, 4200 m, 02 June 2016, *Boza 3157*; *3158*; *3159*; *3160*; *3161*; *3162*; *3163*; *3164*; *3165*; *3166*; *3167*; *3168* (USM!, Z!); cordillera between Pisco and Ayacucho, 3900–4000 m, 01 May 1910, *Weberbauer 5433* (F!, GH!). Huancavelica, alrededores del Puente Licapa San Antonio, 13°22'39"S, 074°52'18"W, 4456 m, 19 June 2007, *Beltrán 6391* (USM!); Dist. de Manta, localidad San Luis, cerca de la carretera que va hacia San Luis, 12°37'08"S, 075°09'43"W, 4350 m, 15 September 2017, *Quispe 79* (CUZ!, USM!, Z!); Huaytamayoc-Tansiri, 4500 m, 01 May 1956, *Tovar 2552* (USM!). **Lima**: Canete, Cañete Valley, above Hortigal near madean, 12°57'S, 075°46'W, 3600 m, 07 March 1987, *Brandbyge 237* (AAU!). Yauyos, Laraos, 12°23'S, 075°49'W, 04 February 2000, *Beltrán 3394* (GOET!).

### ﻿26. Polylepis pilosissima

Taxon classificationPlantaeRosalesRosaceae

T.Boza & M.Kessler sp. nov.

758E90DD-14B9-51AE-81DC-EA8CBF9BF549

urn:lsid:ipni.org:names:77301644-1

Figs 69 , 70

#### Diagnosis.

This species differs from *Polylepisflavipila* (Bitter) M.Kessler & Schmid-Leb. (2006) in having longer leaflets with dense long hairs on the lower surfaces and crenate margins with more teeth per side.

**Figure 69.**
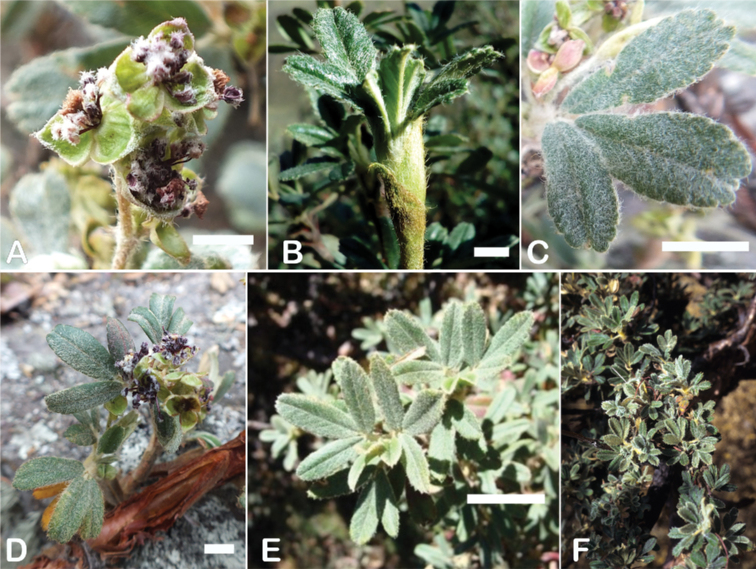
*Polylepispilosissima* T.Boza & M.Kessler **A** flowers **B** stipular sheaths **C** upper leaf surface **D** flowering branch **E** leaves **F** branch. Scale bars: 5 mm (**A, D**); 3 mm (**B**); 2 cm (**E**); 1 cm (**C**). Photographs **A–E** E.G. Urquiaga F **F** M. Kessler.

#### Type.

Peru. Lima: Huarochiri, Carapoma, Bosque de Japani, 11°38'11"S, 076°27'10"W, 3859 m, 11 Nov 2014, *T.E. Boza E. & E. Urquiaga 3023* (holotype: USM!; isotype: Z!).

**Figure 70.**
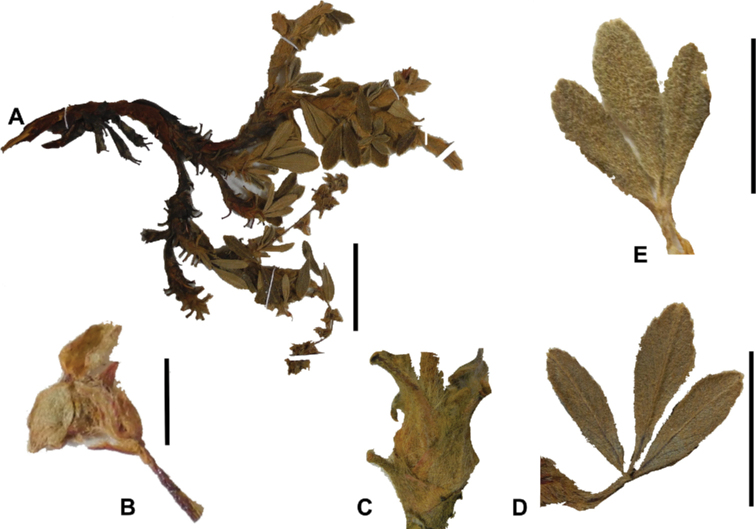
*Polylepispilosissima* T.Boza & M.Kessler **A** flowering branch **B** fruit **C** stipular sheaths **D** lower leaf surface **E** upper leaf surface (**A***Tovar 680***B, E***Isidoro s.n***C***Kessler 3591***D***Cerrate 1265*). Scale bars: 4 cm (**A**); 6 mm (**B**); 2 cm (**D, E**). Photographs by T. E. Boza E.

#### Description.

***Trees*** 4–8 m tall. ***Leaves*** slightly congested at the branch tips, imparipinnate with one pair of leaflets, obtrullate in outline, (1.9–)2.8–3.6 × 1.9–3.7 cm; rachises densely villous, points of leaflet attachment with a tuft of long hairs; stipular sheaths not spurred, densely villous on the outer surfaces; leaflets obovate in outline, second pair from the terminal leaflet the largest, one of this pair (1.6–)1.9–2.7 × 0.5–0.9 cm; margin crenate with 7–14 teeth, apically acute or emarginate, basally cuneate; upper leaflet surfaces densely pilose; lower leaflet surfaces densely pilose with yellowish hairs 1.0–1.2 mm long. ***Inflorescences*** pendant, (1.5–)2.1–5.2 cm long, bearing 3–5 flowers; floral bracts 2.5–3.9 mm long, narrowly triangular, densely villous on the outer surface; rachises villous. ***Flowers*** 7.3–8.5 mm diam.; sepals 3–4, ovate, green to reddish, densely villous outside; stamens 11–17, anthers orbicular, with a dense tuft of straight white hairs on the upper half; styles fimbriate, 2.5–3.9 mm long. ***Fruits*** turbinate, with 3–4 irregular flattened ridges with a series of spines, densely pilose; 5.4–6.4(–8.8) × 1.9–3.1(–5.1) mm including spines. ***Tetraploid***.

#### Distribution, habitat and ecology.

*Polylepispilosissima* is restricted to the Department of Lima, Peru (Fig. 73). The species occurs in dry and relatively cold areas at 3500–4400 m elevation. It grows in mixed forests with *Gynoxyisnitida* (Rivera Paucar 2018). The largest forest of *P.pilosissima* is “Japani forest”, located in Carampoma (Huarochiri, Lima) with 665 ha (Ñingle and Florencio 2013; Rivera Paucar 2018). It hosts a high diversity of birds (74 spp.), including endemic and threatened species, such as *Oreotrochilusmelanogaster*, *Ochthoecaoenanthoides* and *Conirostrumbinghami* (= *Oreomanesfraseri*) (Sembrero and Valencia 2016; Rivera Paucar 2018). Japani forest stores about 47,000 t of biomass and 24,500 t of carbon in the above-ground woody part (Rivera Paucar 2018).

#### Etymology.

The species epithet “pilosissima” refers to the characteristic dense, long pilose hairs of the species.

#### Conservation status.

The EOO for *P.pilosissima* is estimated as 5,129 km^2^, the AOO is assessed at 28 km^2^ and it is known from only five locations. The largest stand of this species is protected within the “Japani” Private Conservation Area. Based on its fragmented and restricted distribution, we assess *P.pilosissima* as Critically Endangered (CR A2a, B2a).

#### Notes.

The populations of *Polylepis* in Huarochiri Province (Lima) have previously been identified as *P.flavipila* (Kessler and Schmidt-Lebuhn 2006; Mendoza and Cano 2012). Indeed, *P.pilosissima* resembles *P.flavipila* in having one lateral leaflet pair, obovate leaflets with crenate margin with pilose hairs and short inflorescences with few flowers. However, it has leaflets (1.6–)1.9–2.7 cm long, crenate leaflet margins with 7–14 teeth per side, upper leaflet surfaces densely pilose and lower leaflet surface hairs 1.0–1.2 mm long, whereas *P.flavipila* has leaflets (1.2–)1.6–2.0 cm long, crenate margins with 4–6 teeth per side, upper surface sparsely pilose and lower leaflet surface hairs 0.5–0.6 mm long. Additionally, *P.pilosissima* is morphologically similar to *P.subsericans*, with which it shares lower leaflet surface hair density and length. However, *P.pilosissima* has obovate leaflets with crenate margins, pilose hairs which are dense on both leaflet surfaces and 11–17 flowers per inflorescence, whereas *P.subsericans* has narrowly elliptic leaflets with entire to slightly serrate apex margins, strigose hairs which are sparse on the upper leaflet surfaces and dense on the lower ones and 9–13 flowers per inflorescence.

#### Specimens examined.

**Peru. Lima**: Huarochiri, Quebrada Yanac in Valle Sta. Eulalia, 11°35'S, 076°27'W, 4000 m, 27 January 1987, *Boertmann 9* (AAU!); Carapoma, Bosque de Japani, 11°38'11"S, 076°27'10"W, 3859 m, 11 November 2014, *Boza 3023* (USM!, Z!); 10 km NE of Suchi, ca. 61 road km NE of Chosica on road to Huanza, 11°41'24"S, 076°34'48"W, 3900–4000 m, 06 May 1978, *Gentry 21638* (MO!, USM!); huacamachay (Alto río Sta. Eulalia), 4000 m, 09 October 1987, *Hocking s.n* (USM!); Comunidad Campesina de Llacuas, 06 November 1995, *Ignacio s.n* (USM!); Sta. Eulalia Valley, ca. 15 km NE Huansa, 11°37'S, 076°26'W, 3800 m, 06 September 1991, *Kessler 3063* (GOET!); *3064* (GOET!); *3426* (GOET!, LPB, MO!); *3427* (AAU!, GOET!, LPB, MO!); *3428* (AAU!); *3588* (GOET!); *3589* (GOET!); *3590* (GOET!); *3591* (AAU!, GOET!, LPB, MO!); *3593* (GOET!); *3653* (GOET!); arriba de Santa Eulalia, 01 March 1966, *Koepcke s.n* (USM!). Yauyos, entre Pallaca y Huacracocha a 14 Km. de Tupe, 4000 m, 22 January 1952, *Cerrate 1265* (GOET!, MO!, USM!).

### ﻿27. Polylepis subsericans

Taxon classificationPlantaeRosalesRosaceae

J.F.Macbr., Candollea 5: 367. 1934.

09203005-B93C-5889-A87A-8C5C851EF375

Figs 71 , 72

#### Type.

Peru. Ayacucho: Hacienda Tortorabamba, 3500–3600 m, May 1910, *Weberbauer 5487* (holotype: F!; isotypes: F!, G!)

**Figure 71.**
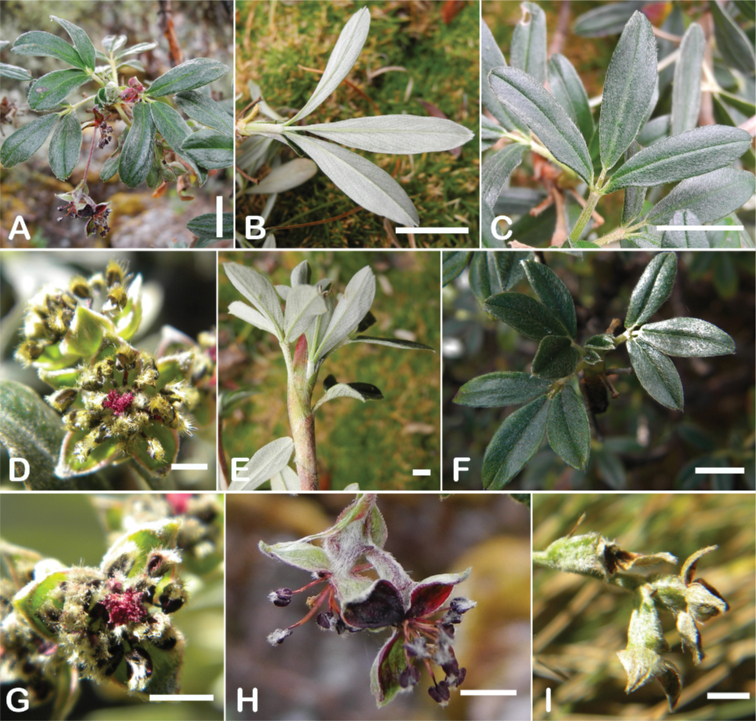
*Polylepissubsericans* J.F.Macbr **A** flowering branch **B** lower leaflet surface **C** upper leaflet surface **D** flowers **E** stipule sheaths **F** leaves **G** flower **H** flowers and fruits **I** fruits (**A–I***Boza & Urquiaga 3033*). Scale bars: 1 cm (**A–C, F**); 2 mm (**D**); 3 mm (**E, G–I**). Photographs by T.E. Boza E.

#### Description.

***Trees*** 3–10 m tall. ***Leaves*** slightly congested at the branch tips, imparipinnate with one pair of leaflets, obtrullate in outline, (1.7–)2.8–3.4 × 1.7–2.5 cm; rachises densely pilose, points of leaflet attachment with a tuft of long hairs; stipular sheaths apically acute with spurs, densely strigose on the outer surfaces; leaflets narrowly elliptic in outline, second pair from the terminal leaflet the largest, one of this pair (1.3–)1.7–2.8 × 0.5–0.7 cm; margin entire to slightly serrate at apex with 3–4 teeth, apically round or emarginate with the trichomes from the lower surface projecting into the notch, basally unequally cordate; upper leaflet surfaces sparsely strigose; lower leaflet surfaces densely strigose with yellowish hairs 0.7–1.2 mm long. ***Inflorescences*** pendant, (1.9–)2.5–4.9(–5.6) cm long, bearing 3–4(–6) flowers; floral bracts 3.2–6.6 mm long, narrowly triangular, densely pilose on the outer surface; rachises pilose. ***Flowers*** 7.7–9.6 mm diam.; sepals 4, ovate, green, densely pilose outside; stamens 9–13, anthers orbicular, with a dense tuft of straight white hairs on the upper half; styles fimbriate, 2.0–4.2 mm long. ***Fruits*** turbinate, with 3–4 irregular flattened ridges with a series of spines, densely pilose; 7.5–9.1 × 4.3–5.9 mm including spines. ***Tetraploid***.

**Figure 72.**
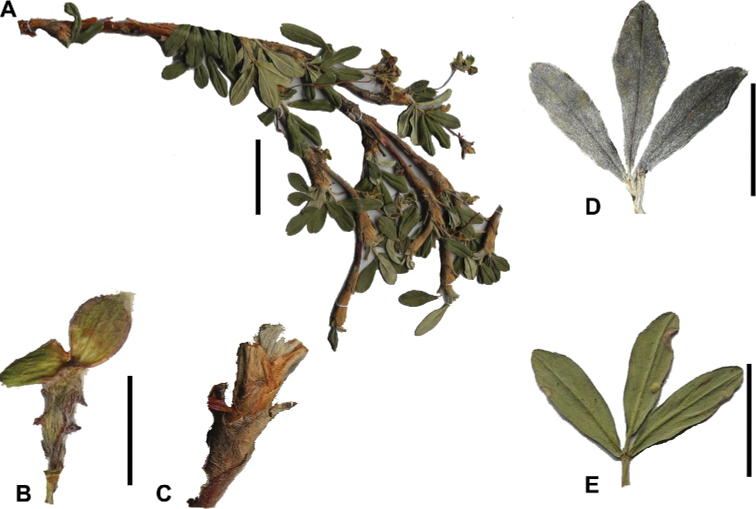
*Polylepissubsericans* J.F.Macbr **A** flowering branch **B** fruit **C** stipular sheaths **D** upper leaf surface **E** lower leaf surface (**A, E***Toivonen 38***B, C***Toivonen 40***D***Arce & Toivonen s.n*). Scale bars: 5 cm (**A**); 9 mm (**B**); 2 cm (**D, E**). Photographs by T. E. Boza E.

#### Distribution, habitat and ecology.

**Figure 73.**
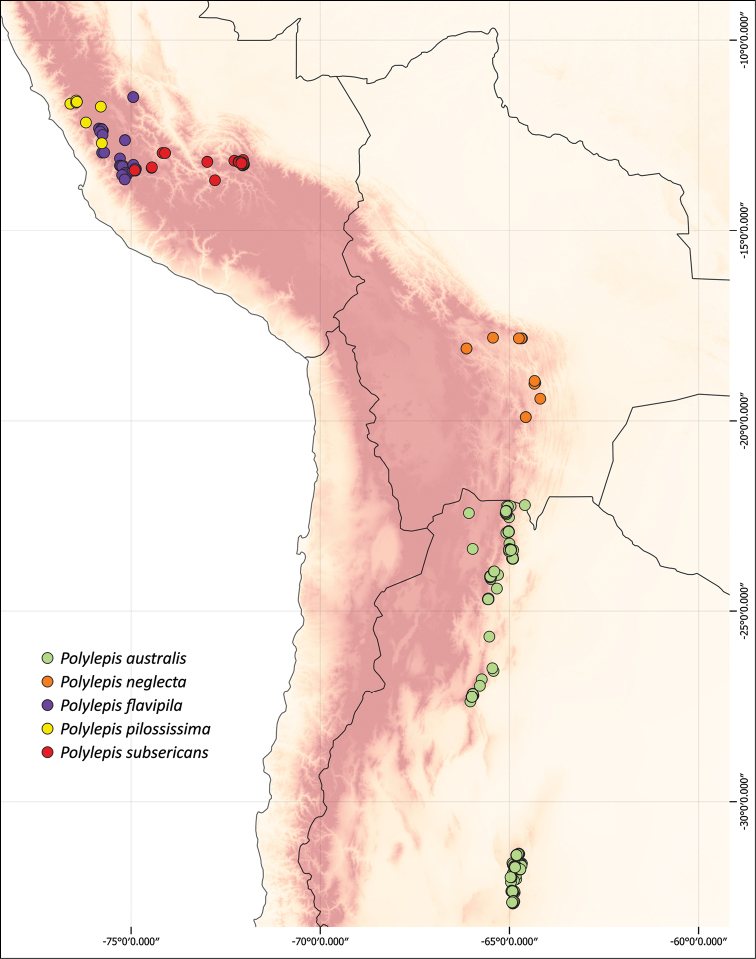
Geographical distribution of the species of the sections *Australes* and *Subsericantes*.

*Polylepissubericans* is distributed in the high mountains of southern Peru from Huaytará (Huancavelica) to (Urubamba) Cusco (Fig. 73). The species occurs in relatively dry and cold areas at 3760–4800(–5100) m elevation. Human activities cause variation of the forest structure, with forest in dry areas being more strongly degraded (Toivonen et al. 2011). During the rainy season, *P.subsericans* shows a positive correlation between temperature and tree-ring growth, pointing out the importance of temperature for its growth (Jomelli et al. 2012). *Polylepissubsericans* is amongst the highest-growing tree species in the world and in Vilcanota (Cusco), forests of *P.subsericans* reach mean maximum tree heights of 13 m at 4650 m (Kessler et al. 2014). These stands grow under mean growing season air temperatures of 3.8 °C and mean growing season soil temperatures of 4.6 °C. *Polylepissubericans* is adapted to high elevations and, relative to other species of *Polylepis*, has low levels of stomatal conductance and high levels of quantum use efficiency (Toivonen et al. 2014). The anatomical traits of the species have been studied in depth by Arroyo (2015). Stands of *P.subsericans* in the Vilcanota Mountains host a moderate diversity of plants (144 spp.) and specialist bird species (30 spp.) (Servat et al. 2002). Among the endemic birds, *Leptasthenuraxenothorax*, *Cinclodesexcelsior* and *Anairetesalpinus* are threatened by habitat reduction, making this habitat a priority for conservation (Fjeldså 1987; Fjeldså and Kessler 1996; Cardenas-Villavicencio and Arque 2010).

#### Conservation status.

The EOO for *Polylepissubsericans* is estimated as 15,223 km^2^, the AOO is assessed at 84 km^2^ and it is known from 11 locations. It has been categorized as EN (B1b(iii)) (Mendoza and León 2006; SERFOR 2006). In Cusco, it is protected within the Private Conservation Areas Network of the Vilcanota Mountain range. Since 2001, it has been subject to reforestation activities by ECOAN, a non-profit NGO dedicated to the conservation of endangered species and threatened Andean ecosystems. However, populations from Ayacucho have high anthropogenic impact from the surrounding communities to the point of almost disappearing completely (Mendoza and Roque 2007). We assess *P.subsericans* as Vulnerable (B1a+B2a).

#### Notes.

*Polylepissubsericans* may be confused with *P.rodolfovasquezii*, with which it hybridizes. They share the lateral leaflet pair number and more or less same leaflet hair length. *Polylepissubsericans* differs by having leaflets (1.3–)1.7–2.8 cm long, strigose hairs and longer inflorescences (1.9–5.6 cm) with 3–6 flowers, whereas *P.rodolfovasquezii* has leaflets 0.9–1.1 cm long, sericeous hairs and inflorescences 0.9–1.1 cm long with just one flower. For additional morphological similarities, see under *P.flavipila* and *P.pilosissima*.

#### Specimens examined.

**Peru. Apurimac**: Abancay, Bosque de Runtococha, 4150 m, 08 April 2003, *Palomino 3964* (QCA!). **Ayacucho**: Huamanga, Torobamba, 3500–3600 m, 01 May 1910, *Weberbauer 5487* (F!). La Mar, Laguna de Toctococha, 12°57'39"S, 074°05'57"W, 4200 m, 30 September 2003, *Mendoza 1013* (MO!). **Cusco**: Calca, Sacsamonte-Huarán, 13°12'58"S, 072°02'05"W, 4340 m, 01 May 2003, *Arce s.n* (USM!); Lares, Quishuarani, 13°08'34"S, 072°02'31"W, 4281 m, 17 January 2014, *Boza 3000* (USM!, Z!); *3121* (USM!, Z!); Bosque de Polylepis de Yanacocha, 13°16'59"S, 072°03'02"W, 4012 m, 17 August 1998, *Mendoza 112* (CUZ!); Dist. Calca, ledge situated on the prominent cliff 1.5 km South (170) of Cancha Cancha Village Huaran, 13°14'37"S, 072°01'14"W, 4545 m, 27 March 2011, *Sylvester 1013*; *1025*; *1027* (CUZ!, Z!); Dist. Calca, top of the prominent tower known by locals as “Kontorqayku” 5 km NE of Huaran, 13°16'06"S, 072°01'16"W, 4398 m, 17 May 2011, *Sylvester 1287* (CUZ!, Z!); on the SW corner of the topmost part of tower, 13°16'06"S, 072°01'16"W, 4410 m, 17 May 2011, *Sylvester 1328*; *1329*; *1344* (CUZ!, Z!); Dist. Calca, the eastern side of the valley 7.2 km 10 N from Huaran, 13°15'06"S, 072°00'57"W, 4649 m, 18 July 2011, *Sylvester 1354*; *1355*; *1359* (CUZ!, Z!); Dist. Calca, upper Potreros forest, on the N facing mountainside above the main Potreros forest. 4 km SW of Cancha Cancha Village, Huaran, 13°15'46"S, 072°02'51"W, 4726 m, 18 July 2011, *Sylvester 1361*; *1362*; *1364*; *1365* (CUZ!, Z!); Dist. de Calca, the Wakapacana forest, on the eastern side of the Valley 7.2 km 10 N from Huaran, 13°15'05"S, 072°00'56"W, 4637 m, 26 March 2012, *Sylvester 1408*; *1413* (CUZ!, LPB, Z!); Dist. Calca, within the SW facing forest at the top of the prominent tower known by local as “Kontorqayku” 5 km NE of Huaran, 13°16'07"S, 072°01'17"W, 4388 m, 11 June 2012, *Sylvester 1630*; *1690* (CUZ!, Z!); Prominent ledge situated on the prominent SW facing cliff face 1.5 km South (170) of Cancha Cancha Village Huaran, 13°14'34"S, 072°01'14"W, 4517 m, 09 March 2011, *Sylvester 804*; *809*; *813*; *820*; *868*; *875*; *876*; *880*; *881* (CUZ!, Z!); Dist. Calca. Ledge situated on the prominent cliff 1 km South (150) of Cancha Cancha Village Huaran, 13°14'35"S, 072°01'14"W, 4500 m, 27 March 2011, *Sylvester 937*; *947*; *948*; *973*; *975*; *977* (CUZ!, Z!). La Convención, Ccayara 18L 718405/ UTM 8540752, 4416 m, 01 August 2002, *Arce s.n* (CUZ!, USM!). Urubamba, Yanahuara, Mantanay, 13°11'57"S, 072°09'33"W, 4330 m, 17 June 2015, *Boza 3033*; *3085*; *3086*; *3087*; *3088*; *3089* (USM!, Z!); Dist. Urubamba, ACP Mantanay, 10 km up the valley from Yanahuara, by the side of Laguna Manalloqsa, in the small valley 3 km East of Laguna Ipsaycocha, 13°12'01"S, 072°08'42"W, 4640 m, 24 June 2012, *Sylvester 1714* (Z!); 13°12'02"S, 072°08'46"W, 4638 m, 03 February 2011, *Sylvester 405*; *408*; *512* (CUZ!, Z!); Dist. Urubamba, ACP Mantanay 10 km up the valley from Yanahuara, by the side of Laguna Manalloqsa, in the small valley 3 km of Laguna Ipsacocha, 13°12'08"S, 072°08'44"W, 4806 m, 03 February 2011, *Sylvester 518*; *531*; *536*; *544*; *575*; *576*; *585* (CUZ!, Z!); Dist. Huayllabamba, terrace situated on the N side of Laguna Qellococha and to the E of the waterfall 5 km N of Huayocari Village, 13°16'34"S, 072°03'09"W, 4343 m, 10 March 2011, *Sylvester 642*; *671*; *679* (CUZ!, Z!); 13°16'35"S, 072°03'01"W, 4227 m, 09 March 2011, *Sylvester 684*; *686*; *688*; *691* (CUZ!, Z!); 13°16'35"S, 072°03'03"W, 4226 m, 10 March 2011, *Sylvester 695*; *740* (CUZ!, Z!); Dist. de Huayllabamba. 5 km N of Huayocari village, 800 m NW of Laguna Qellococha along the path leading to higher lake, 13°16'32"S, 072°03'10"W, 4366 m, 10 March 2011, *Sylvester 741*; *742*; *749*; *751* (CUZ!, Z!); Mantanay, 13°11'08"S, 072°09'20"W, 4370 m, 19 July 2006, *Toivonen 11*; *12* (CUZ!); Dist. Huayllabamba, localidad Qelloqocha, 13°16'16"S, 072°03'09"W, 4350 m, 19 July 2006, *Toivonen 38*; *39*; *40*; *65* (CUZ!); Dist. Huayllabamba, entre Huayoccari y las lagunas de Yanacocha y Kellococha, 2900–4600 m, 17–18 July 1989, *Tupayachi 1137* (MO!); *1140* (MO!); Huayllabamba, Lagunas Yanachocha y Quellococha hacia San Juan, NE de Cusco, 13°16'S, 072°04'W, 2900–4600 m, 19 August 1989, *Tupayachi 1192*; *1212* (MO!); Localidad Yucay-Puyuc-Hueskana, 4200 m, 24 April 1993, *Tupayachi 2259* (CUZ!); Localidad Parte alta de Cuyuc, 4200 m, 28 May 1994, *Tupayachi 2554* (CUZ!); Pumahuanca, Bosque de Quenñaquemocuyo, 4400 m, 18 June 2004, *Tupayachi 4808* (QCA!); Dist. Huayllabamba, Laguna Yanaccocha y Kello ccocha, 13°21'15"S, 072°03'55"W, 3800–4200 m, 07 January 1989, *Tupayachi 859* (MO!); Localidad Mantanay, 13°12'02"S, 072°08'57"W, 4526 m, 01 May 2003, *Arce s.n* (CUZ!).

### ﻿ Polylepis section Incanaee

Taxon classificationPlantaeRosalesRosaceae

T.Boza & M.Kessler sect. nov.

3A02E00D-5BA7-57ED-A94E-FDA7BCFCCC92

urn:lsid:ipni.org:names:77301645-1

#### Diagnosis.

Trees or shrubs; lower leaflet surfaces glabrous or with hispid, puberulous, lanate, tomentose, or villous hairs; fruits with irregular flattened ridges with a series of spines, glabrous to densely hispid, tomentose or villous.

#### Type.

*Polylepisincana* Kunth.

#### Notes.

The sectional epithet *Incanaee* is a plural adjective agreeing in gender with *Polylepis*. Section Incanaee contains species with usually few lateral leaflet pairs (often only one), frequently glabrous upper leaflet surfaces, lower leaflet surfaces glabrous or with a dense layer of very short pannose hairs rarely mixed with tomentose hairs (as in *P.besseri* and *P.incarum*) or mostly with tomentose, lanate or villous hairs. Furthermore, fruits in this section bear 2–5 irregular, hard, flattened ridges with a series of spines. Table 8 provides an overview of the arrangement of the taxa by different authors.

**Table 8.** Alignment of the taxa of the Polylepissect.Incanaee according to Bitter (1911), Simpson (1979), Segovia et al. (2018) and the present study.

Bitter (1911)	Simpson (1979)	Segovia et al. (2018)	This study
*P.incana*	*P.incana*	*P.incana*	*P.incana*
	*P.besseri*	*P.incarum*	*P.incarum*
—		*P.lanata*	*P.lanata*
—			*P.sacra*
*P.triacontandra*		*P.triacontandra*	*P.triacontandra*
*P.subquinquefolia*			
*P.besseri*		*P.besseri*	*P.besseri*
		*P.subtusalbida*	*P.subtusalbida*
*P.pallidistigma*		*P.pallidistigma*	*P.pallidistigma*
*P.crista-galli*		*P.crista-galli*	*P.crista-galli*
*P.rugulosa*		*P.rugulosa*	*P.rugulosa*
*P.tenuiruga*			
—	—	*P.pacensis*	*P.pacensis*
—	*P.racemosa*	*P.racemosa*	*P.racemosa*
—	—	—	*P.acomayensis*
*P.tomentella*	*P.tomentella*	*P.tomentella*	*P.tomentella*
			*P.incanoides*
			*P.nana*
			*P.fjeldsaoi*
*P.tarapacana*		*P.tarapacana*	*P.tarapacana*

Within section Incanaee, we recognized three subsections, based on their morphological distinctness as follows: subsection Racemosae (9 species) with lanate, tomentose or villous lower leaflet surfaces, 2–4 lateral leaflet pairs and fruits densely covered by tomentose or villous hairs; subsection Besseria (5 species) with short pannose or tomentose lower leaflet surfaces, 1–2 lateral leaflet pairs and fruits with 2–5 flattened ridges with a series of spines; and subsection Incanaee (6 species) with pannose lower leaflet surfaces, one lateral leaflet pair and densely villous fruits. Within each subsection, species are essentially allopatric in distribution.

##### Climatic niches in Polylepissect.Incanaee

**Figure 74.**
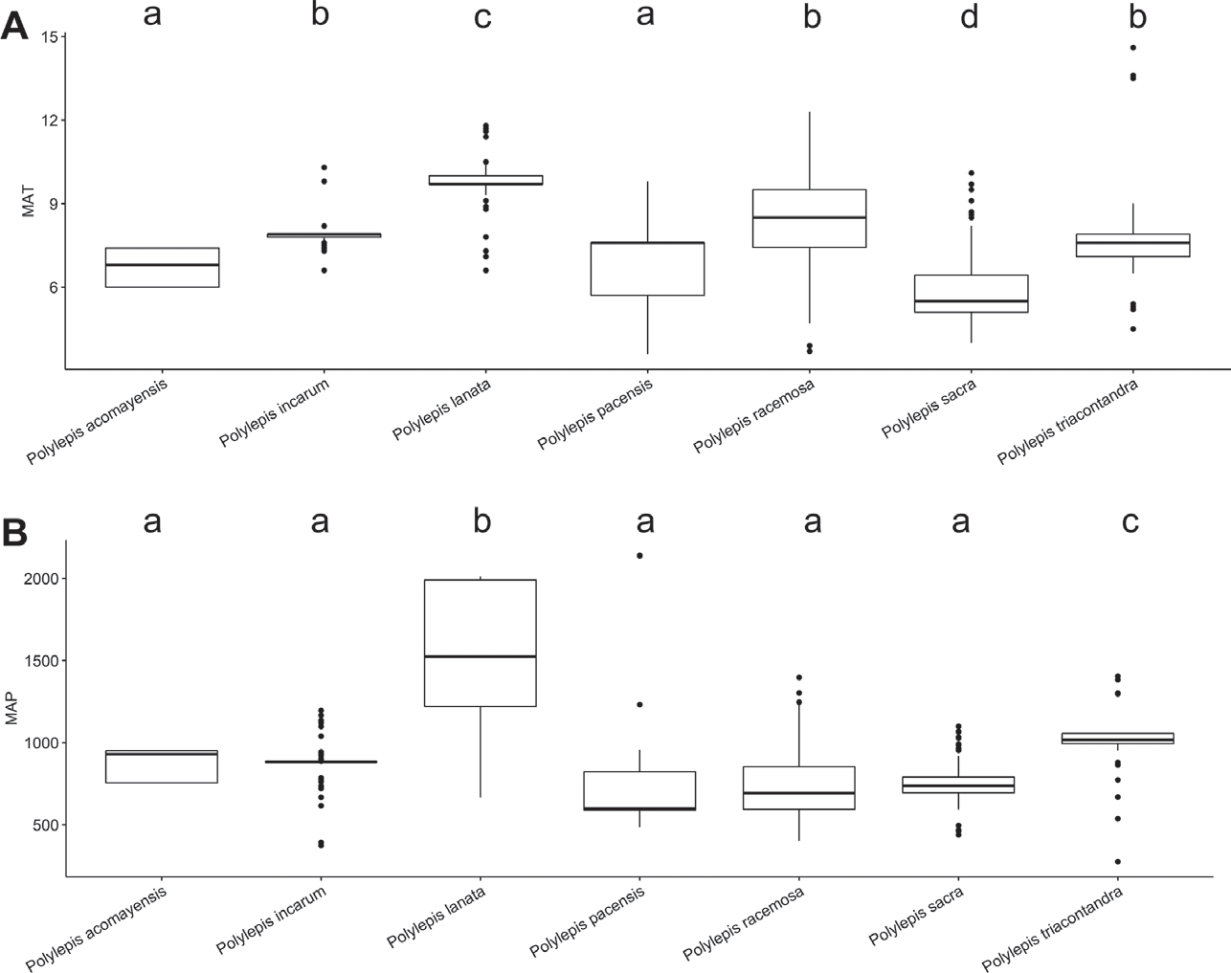
Box plots showing the climatic niches of the species of subsection Racemosae in relation to MAT (**A**) and MAP (**B**). See Fig. 12 for details on data presentation.

**Figure 75.**
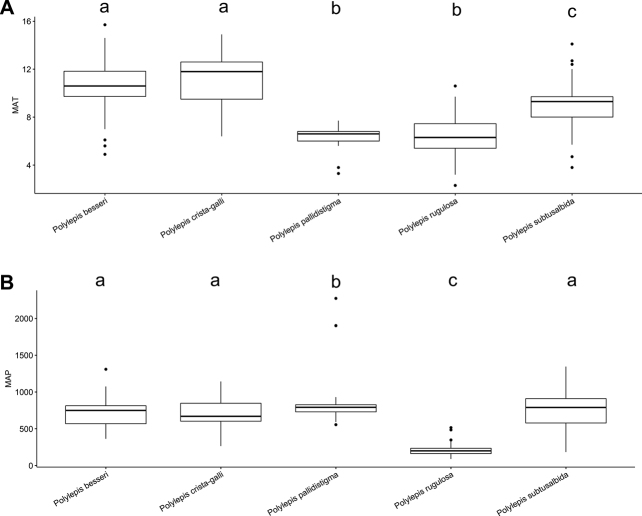
Box plots showing the climatic niches of the species of subsection Besseria in relation to MAT (**A**) and MAP (**B**). See Fig. 12 for details on data presentation.

**Figure 76.**
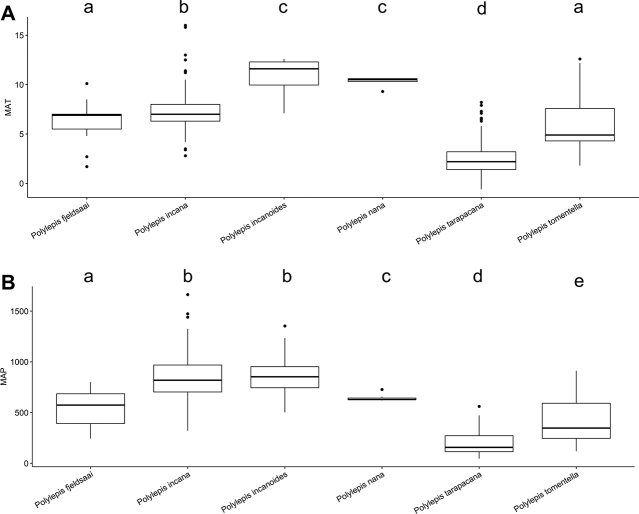
Box plots showing the climatic niches of the species of subsection Incanaee in relation to MAT (**A**) and MAP (**B**). See Fig. 12 for details on data presentation.

Climatic niches among the species of this group differ notably (Figs 74–76). *Polylepistarapacana* grows under the coldest conditions (mean of 2.6 °C Mean Annual Temperature, MAT); whereas other species, such *P.crista-galli* (11.2 °C), *P.incanoides* (11.0 °C), *P.besseri* (10.8 °C) and *P.nana* (10.4 °C), grow under noticeably higher temperatures. These differences of up to 7.6 °C correspond to about 1400 m elevation. Regarding Mean Annual Precipitation (MAP), *P.lanata* grows under the most humid conditions (mean of 1547 mm MAP), followed by *P.triacontandra* (1057 mm). In contrast, species growing in drier areas are *P.tarapacana* (180 mm MAP), *P.rugulosa* (210 mm) and *P.tomentella* (412 mm).

Focussing on the individual subsections, the five species of subsect. Besseria are allopatric and mostly have rather similar climatic niches, although most species have some level of climatic differentiation; only *P.besseri* and *P.crista-galli* are identical. Subsection Racemosae includes seven allopatric species that again mostly differ in either MAP or MAT, partly quite considerably. For example, *Polylepislanata* and *P.triacontandra* occur close to each other in Bolivia, but have ecological differences, with *P.triacontandra* growing under relatively colder and more humid conditions. Additionally, *P.lanata* and *P.sacra*, long thought to be the same species (Simpson 1979; Mendoza and Cano 2012), have distinct climatic niches, with *P.lanata* growing under much warmer and humid conditions than *P.sacra*. Within this subsection, only *P.acomayensis* and *P.pacensis* have identical climatic niches. Finally, in subsect. Incanaee, all species are climatically distinct. For instance, *Polylepisincanoides*, *P.nana* and *P.tomentella* all occur in Bolivia, but *P.tomentella* grows under comparatively cold and dry conditions, *P.incanoides* under warm and humid ones and *P.nana* under warm and dry ones.

### ﻿Subsection Racemosae

Taxon classificationPlantaeRosalesRosaceae

T.Boza & M.Kessler sect. nov.

50C59816-A6DE-5A75-B42C-F51B5329BF32

urn:lsid:ipni.org:names:77301646-1

#### Diagnosis.

Trees; 1–3 lateral leaflet pairs; lower leaflet surfaces glabrous or densely lanate, tomentose or villous; fruits with 2–5 irregular flattened ridges with a series of spines, densely pilose, tomentose or villous.

#### Type.

*Polylepisracemosa* Ruiz & Pav.

#### Note.

The subsectional epithet *Racemosae* is a plural adjective agreeing in gender with *Polylepis*.

### ﻿28. Polylepis acomayensis

Taxon classificationPlantaeRosalesRosaceae

T.Boza & M.Kessler sp. nov.

D30CD791-446F-5E6A-8A8E-2DAE336F0260

urn:lsid:ipni.org:names:77301647-1

Figs 77 , 78

#### Diagnosis.

This species differs from *P.triacontandra* Bitter by its smaller leaflets with different type and length of hairs and shorter inflorescences with lower number of flowers.

**Figure 77.**
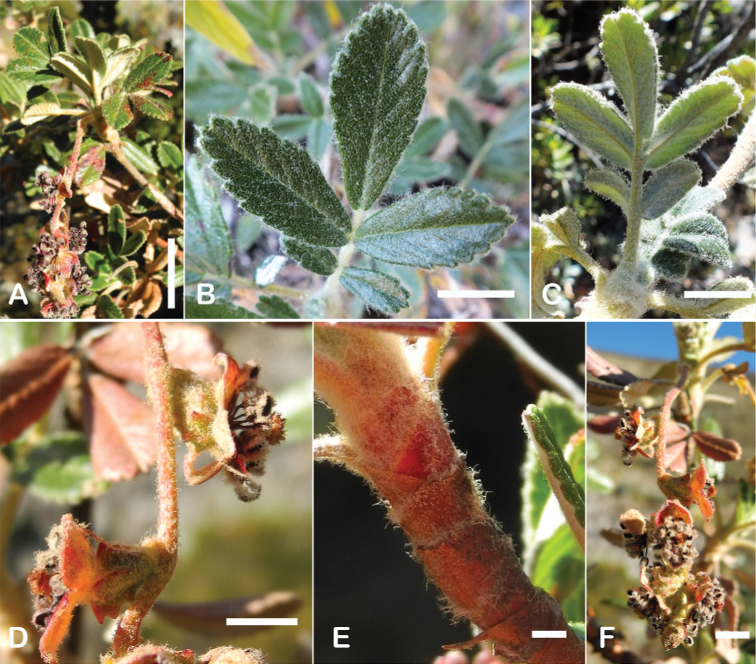
*Polylepisacomayensis* T.Boza & M.Kessler **A** flowering branch **B** upper leaf surface **C** lower leaf surface **D** fruits **E** stipular sheaths **F** flowers (**B***Boza & Urquiaga 3038***A, C, D, E, F***Boza & Urquiaga 3039*). Scale bars: 1 cm (**A–C**); 5 mm (**D, F**); 2 mm (**E**). Photographs **A, D, E, F** T.E. Boza E. **B, C** E.G. Urquiaga F.

#### Type.

Peru. Cusco: Acomayo. Rondocan, bosque de Llamacocha, 13°50'34"S, 072°02'31"W, 3933 m, 12 Jul 2015, *T.E. Boza E. & E. Urquiaga 3038* (holotype: USM!; isotype: Z!).

**Figure 78.**
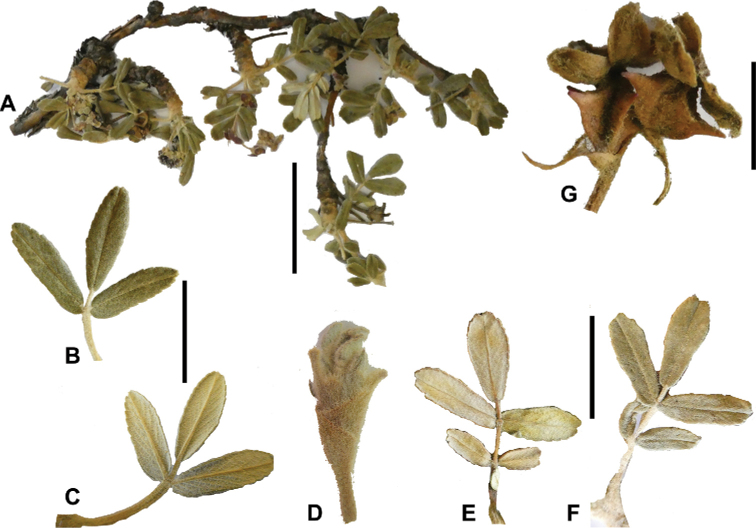
*Polylepisacomayensis* T.Boza & M.Kessler **A** flowering branch **B** upper leaf surface **C** lower leaf surface **D** stipular sheaths **E** lower leaf surface **F** upper leaf surface **G** fruits (**A, D, G***Boza 3135***B, C***Boza 3039***E, F***Boza 3133*). Scale bars: 7 cm (**A**); 3 cm (**B, C**); 2 cm (**E, F**); 7 mm (**G**). Photographs by E. G. Urquiaga F.

#### Description.

***Trees*** 3–8 m tall. ***Leaves*** slightly congested at the branch tips, imparipinnate with 1(–2) pair of leaflets, obtrullate in outline, (2.7–)3.5–4.5 × 2.6–3.3 cm; rachises densely villous, points of leaflet attachment with a tuft of long hairs; stipular sheaths apically truncate, densely villous on the outer surfaces; leaflets narrowly obovate in outline, second pair from the terminal leaflet the largest, one of this pair 1.6–2.3 × 0.5–0.9 cm; margin crenate with 6–9 teeth, apically round or emarginate, basally acute; upper leaflet surfaces sparsely villous; lower leaflet surfaces densely villous with whitish hairs 0.9–1.1 mm long mixed with pannose hairs, second pair of leaflets, if present, very small. ***Inflorescences*** pendant, 2.0–4.0 cm long, bearing 5–7 flowers; floral bracts 3.9–4.2 mm long, narrowly triangular, densely villous on the outer surface; rachises villous. ***Flowers*** 10.2–10.7 mm diam.; sepals 4, ovate, green, densely villous outside; stamens 19–25, anthers orbicular, with a dense tuft of straight white hairs on the upper half; styles fimbriate, 2.3–4.0 mm long. ***Fruits*** turbinate, with 2–3 irregular flattened ridges with a series of spines, densely villous; 4.2–5.7 × 3.2–4.4 mm including spines. ***Tetraploid***.

#### Distribution, habitat and ecology.

*Polylepisacomayensis* occurs in southern Cusco (Peru) in relatively cold and dry Andes at 3800–4160 m elevation (Fig. 92). It grows in mixed forests with *Escalloniaresinosa*.

#### Etymology.

This species is named after Acomayo, the Province to which its distribution appears to be restricted.

#### Conservation status.

The EOO for *Polylepisacomayensis* is estimated as 110 km^2^, the AOO is assessed at 12 km^2^ and it is known only from three locations. The species is unprotected. Logging, grazing and agriculture are the most important activities affecting these forests. We assess *P.acomayensis* as Endangered (A2a, B1a+B2a, C2a).

#### Notes.

*Polylepisacomayensis* resembles *P.triacontandra* in having 1(–2) lateral leaflets pairs as well as similar leaflet margin and density of hairs. However, it has narrowly obovate leaflets 1.6–2.3 × 0.5–0.9 cm long with villous hairs 0.9–1.1 mm long and inflorescences 2.0–4.0 cm long with 5–7 flowers, whereas *P.triacontandra* has narrowly elliptic leaflets (2.0–)2.6–3.3 × 0.7–1.1 cm long with tomentose hairs 0.4–0.8 mm long and inflorescences 4.9–9.5 cm long with 11–13 flowers. *Polylepisacomayensis* is also morphologically similar to *P.sacra* with which it shares similar leaflet size, margin and hair density. The most obvious differences between *P.acomayensis* and this species are the number of lateral leaflet pairs, with *P.acomayensis* having 1(–2) pairs, whereas *P.sacra* has 2–3 pairs. Furthermore, *P.acomayensis* has villous hairs 0.9–1.1 mm long and inflorescences 2.0–4.0 cm long whereas *P.sacra* has lanate hairs 1.3–1.5 mm long and inflorescences 5.0–8.8 cm long.

#### Specimens examined.

Peru. Cusco: Acomayo, Rondocan, Llamacocha, 13°50'53.6"S, 71°45'32.7"W, 3933 m, 12 July 2015, *Boza & Urquiaga 3038*; *3122*; *3123*; *3124*; *3125*; *3126*; *3127*; *3128*; *3129*; *3130*; *3131*; *3132* (USM!, Z!). Paruro, Accha, Kangal, 13°59'41.2"S, 71°48'42.3"W, 4194 m, 12 July 2015, *Boza & Urquiaga 3039*; *3133*; *3134*; *3135*; *3136*; *3137*; *3138*; *3139*; *3140*; *3141*; *3142*; *3143* (USM!, Z!).

### ﻿29. Polylepis incarum

Taxon classificationPlantaeRosalesRosaceae

(Bitter) M.Kessler & Schmidt-Leb., Oranisms Diversity Evol. 6(1): 69. 2006.

8662911E-93C2-518A-A254-625F439ECF3D

Figs 79 , 80

Polylepis besseri Hieron. subsp. incarum (Bitter) M.Kessler, Candollea 50(1): 157. 1995. Type. Based on Polylepisincarum Kunth subsp. Bitter.
Polylepis incana Kunth subsp. brachypoda Bitter, Bot. Jahrb. Syst. 45: 644. 1911. Type. Bolivia. Lago Titicaca, Isla del Sol, near Challa, *Seler 148a* (holotype: B destroyed).

#### Basionym.

PolylepisincanaKunthsubsp.incarum Bitter, Bot. Jahrb. Syst. 45: 643. 1911.

**Figure 79.**
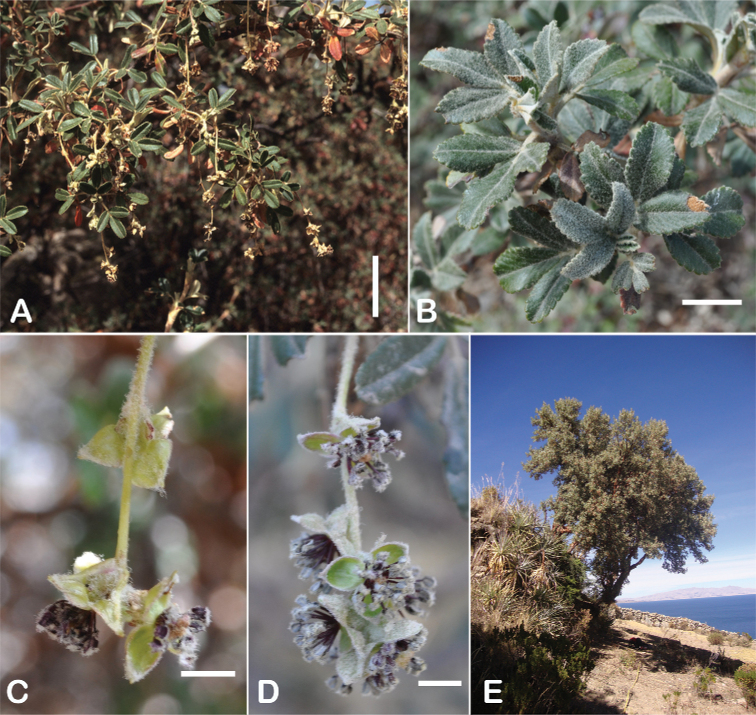
*Polylepisincarum* (Bitter) M.Kessler & Schmidt-Leb **A** flowering branches **B** leaves **C** flowers **D** flowers **E** habit. Scale bars: 3 cm (**A**); 2 cm (**B**); 5 mm (**C, D**). Photographs **A** M. Kessler **B–E** A. Domic.

#### Type.

Bolivia. Lago Titicaca, Isla del Sol near Challa, *Seler 148* (holotype: B destroyed).

**Figure 80.**
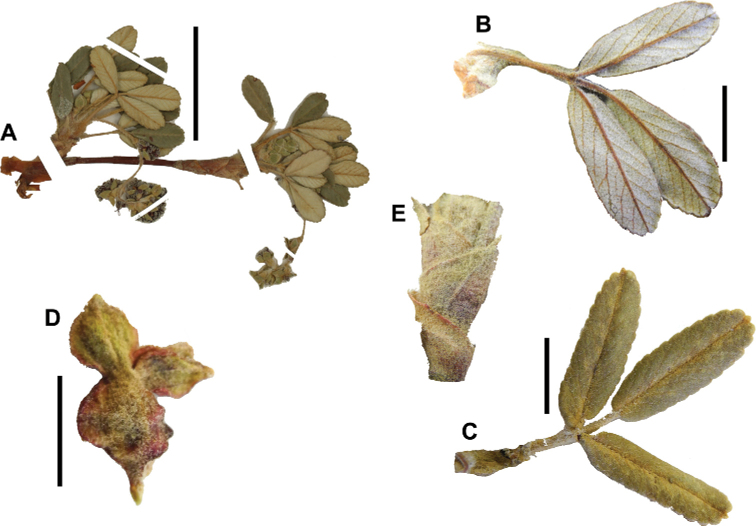
*Polylepisincarum* (Bitter) M.Kessler & Schmidt-Leb **A** flowering branch **B** lower leaf surface **C** upper leaf surface **D** fruit **E** stipular sheaths (**A***Laegaard 17730***B***Beck 7689***C***Kessler 3443***D, E***Kessler 3640*). Scale bars: 6 cm (**A**); 2 cm (**B, C**); 8 mm (**D, E**). Photographs by T. E. Boza E.

#### Neotype.

Bolivia. La Paz, Prov. Manco Capac, Isla del Sol, Yumani, 16°2'S, 69°8'W, 3980 m, 19 Feb 2005, *I. Jiménez & G. Cory 2716* (neotype, designated by Kessler and Schmidt-Lebuhn 2006, pg. 6: LPB!; isoneotypes: AAU!, GOET!, MO!, NY!).

#### Description.

***Trees*** 3–8 m tall. ***Leaves*** slightly congested at the branch tips, imparipinnate with one pair of leaflets, obtrullate in outline, 3.3–4.5(–5.1) × 2.8–4.0 cm; rachises densely tomentose, points of leaflet attachment with a tuft of long hairs; stipular sheaths apically acute with spurs, densely tomentose on the outer surfaces; leaflets broadly elliptic in outline, second pair from the terminal leaflet the largest, one of this pair (1.5–)2.2–3.6 × 0.7–1.3 cm; margin serrate with 8–11 teeth, apically acute, obtuse or slightly emarginate, basally unequally cordate to obtuse; upper leaflet surfaces glabrous; lower leaflet surfaces densely tomentose hairs 0.7–1.0 mm long, mixed with a dense layer of very short white pannose hairs. ***Inflorescences*** pendant, 5.1–7.5(–8.9) cm long, bearing 5–7 flowers; floral bracts 4.7–9.0 mm long, narrowly triangular, densely tomentose on the outer surface; rachises tomentose. ***Flowers*** 8.4–10.1 mm diam.; sepals 4, ovate, green, densely tomentose outside; stamens 15–21, anthers orbicular, with a dense tuft of straight white hairs on the upper half; styles fimbriate, 3.1–3.4 mm long. ***Fruits*** turbinate, with 2–5 flattened ridges with a series of spines, densely tomentose; 4.5–8.0 × 3.1–4.1 mm including spines. ***Tetraploid***.

#### Distribution, habitat and ecology.

*Polylepisincarum* is distributed in the areas surrounding Lake Titicaca in Puno (Peru) and La Paz (Bolivia) (Fig. 92). It occurs as small, isolated patches in rocky places at 3250–4110 m elevation where it is usually planted. In Bolivia, there are only five remaining forests as well as isolated individuals or small groups of trees at various locations around Lake Titicaca (Navarro et al. 2010) covering a total area of 45.12 ha (Domic et al. 2017). The population structure of *P.incarum* shows drastic differences, ranging from 0.6 individuals/100 m^2^ to 71 individuals/100 m^2^ (Domic et al. 2017). Patches of *P.incarum* forest in Bolivia host 58 species of plants, including *Hieraciumpadcayense* and *Calceolariabartsifolia*, which are endemic to the country (Hurtado et al. 2018).

#### Conservation status.

The EOO for *Polylepisincarum* is estimated as 20,736 km^2^, the AOO is assessed at 76 km^2^ and it is known from 12 locations. The species was categorized as EN (B2ab(i,ii,iii)) (Arrázola et al. 2012) and, due to its reduced distribution range, it was reclassified as CR (Domic et al. 2017) in Bolivia. Since the 70s, several reforestation campaigns have been carried out in the Bolivian Altiplano, which mostly promoted the use of exotic species (*Eucalyptus*, *Pinus* and *Cupressus*) over the *P.incarum* patches, due to the high demand for wood and rapid growth of these genera (Liberman et al. 2015; Domic et al. 2017). At many of its locations, the species grows in habitats that are strongly affected by human activities including livestock grazing, logging and burning. In addition, there is indication of reduced reproduction due to pollen limitation and reproductive incompatibility (López et al. 2021). We assess *P.incarum* as Critically Endangered (A1, B1a+B2a, C1+C2a).

#### Notes.

*Polylepisincarum* is morphological similar to *P.pallidistigma* with which it has been previously confused (Mendoza and Cano 2012). Both species share the elliptic leaflet shape, unequally cordate bases of the leaflets and number of flowers per inflorescence. However, *P.incarum* has one pair of lateral leaflets, serrate leaflet margins, lower leaflet surfaces densely tomentose with hairs 0.7–1.0 mm long and mixed with a dense layer of very short pannose hairs and inflorescences 5.1–8.9 cm long, whereas *P.pallidistigma* has 1(–2) lateral leaflet pairs, crenate leaflet margin, a dense layer of very short pannose hairs and inflorescences 2.7–6.0 cm long. *Polylepisincarum* is also similar to *P.subtusalbida*. It differs from this species by having larger and broader leaflets ((1.5–)2.2–3.6 × 0.7–1.3 cm versus 0.9–1.6 × 0.4–0.6 cm), lower leaflet surface densely tomentose with hairs 0.7–1.0 mm long and mixed with a dense layer of very short pannose hairs (glabrous to sparsely tomentose hairs 0.5–1.2 mm long in *P.subtusalbida*) and longer inflorescences (5.1–8.9 cm) with 5–7 flowers (*P.subtusalbida* 1.8–3.7 cm, 3–4 flowers).

#### Specimens examined.

**Bolivia. La Paz**: Camacho, Puerto Acosta 6 kms. hacia La Paz, 3950 m, 06 April 1982, *Beck 7689* (MO!). Copacabana, Lake Titicaca, pr. Ch’alla (Isla del Sol), s.d., *Seler 148* (B). La Paz, Jardín Botánico La Paz, s.d., *Kessler 12626* (GOET!). Manco Kapac, apróx. 5–10 min de la población de Copacabana, camino a Casani, comunidad Copacati, Ladera colindante Cerro Pumacato, 16°11'06"S, 069°04'43"W, 3934 m, 17 October 2014, *Bermejo PMC1*, (LPB, Z!); población de Copacabana, 19 October 2014, *Bermejo SAM7* (LPB, Z!); Chapampa, 1 km S Copacabana, 16°11'S, 069°06'W, 3900 m, 31 July 1991, *Kessler 2782*; *2783*; *2784*; *3438*; *3439*; *3440*; *3441*; *3442*; *3443*; *3444*; *3445*; *3446*; *3448*; *3449*; *3450*; *3451*; *3452*; *3453*; *3454*; *3455*; *3456*; *3457*; *3458*; *3459*; *3460*; *3461*; *3462*; *3463*; *3464*; *3465*; *3466*; *3467*; *3468*; *3469*; *3471*; *3472*; *3473*; *3474*; *3475*; *3476*; *3477*; *3645* (GOET!); Hac. Challa, Isla del Sol, 3850 m, 01 August 1991, *Kessler 3636*; *3637*; *3638*; *3639*; *3640* (GOET!); Cerro Kheñwani (Orientación Sur-W) ladera rocosa, 3900 m, 22 January 1986, *Liberman 1164* (GOET!). Murillo, ciudad de La Paz, Sector Kamirpata (Parche3) Dist. once, MacroDist. Periférica, area Forestal municipal, 16 September 2014, *Bermejo 4* (LPB); *PP-K 1* (LPB, Z!); *PP-K3* (LPB, Z!); *PP-K4* (LPB, Z!). Omasuyos, por la carretera entre Huarina y Kalaque, 16°09'04"S, 068°51'21"W, 3860 m, 22 February 2018, *Fuentes 20182* (LPB). Insel Coaty, Lake Titicaca, 3840 m, 01 March 1910, *Buchtien 4243* (GH!, GOET!); ciudad de La Paz, Av. Ballivian, 09 August 2010, *Steudel 447* (Z!); *449* (Z!); *Steudel 449b* (Z!).

**Peru. Cusco**: Quispicanchis, Dist. Huaro, Urpay, 13°41'01"S, 071°38'22"W, 3200 m, 01 November 2002, *Galiano 4475* (CUZ!, F!, MO!). **Puno**: Atuncolla, Lago Umayo, slope below Sillustani ruins, 4100 m, 04 September 2006, *Kessler 13534*; *13535*; *13536* (GOET!). Lake Titicaca, Lake Titikaka, on Taquile Island, 3800 m, 23 July 1995, *Lægaard 17730* (AAU!).

### ﻿30. Polylepis lanata

Taxon classificationPlantaeRosalesRosaceae

(Kuntze) M.Kessler & Schmidt-Leb., Organisms Diversity Evol. 6(1): 69. 2006.

F127FB1E-77C6-5CA8-A408-9DDE46899D80

Figs 81 , 82

Polylepis racemosa Ruiz & Pav. subsp. lanata (Kuntze) M. Kessler, Candollea 50(1): 147. 1995. Type: based on PolylepisracemosaRuiz & Pav.var.lanata Kuntze.

#### Basionym.

PolylepisracemosaRuiz & Pav.var.lanata Kuntze, Revis. Gen. Pl. 3(3): 77. 1898.

**Figure 81.**
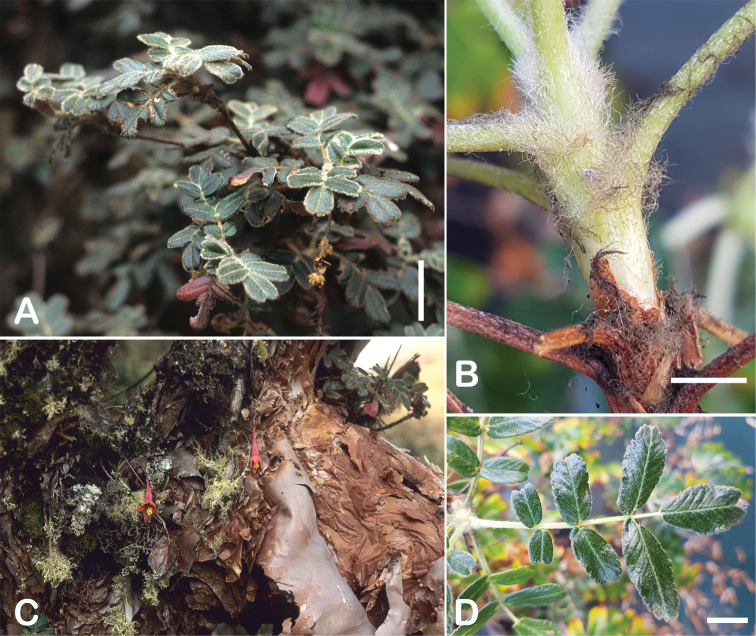
*Polylepislanata* (Kuntze) M.Kessler & Schmidt-Leb **A** flowering branch **B** stipule sheaths **C** bark **D** upper leaf surface. Scale bars: 5 cm (**A**); 1 cm (**D**); 3 mm (**B**). Photographs by M. Kessler.

#### Type.

Bolivia. Cochabamba: Tunari, 3000–4000 m, *Kuntze s.n* (holotype: B destroyed; isotype: NY).

**Figure 82.**
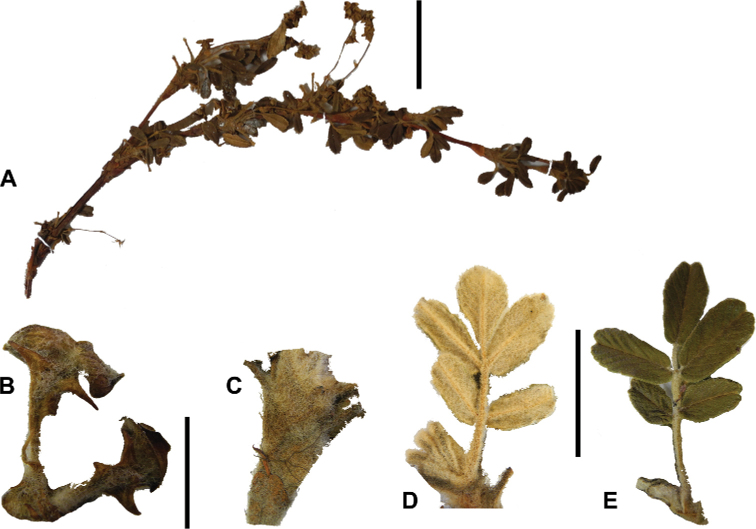
*Polylepislanata* (Kuntze) M.Kessler & Schmidt-Leb **A** flowering branch **B** fruit **C** stipular sheaths **D** lower leaf surface **E** upper leaf surface (**A***Tovar 5342***B***Beck 18100***C***Kessler 3402***D, E***Kessler 12629*). Scale bars: 10 cm (**A**); 10 mm (**B**); 5 cm (**D, E**). Photographs by T. E. Boza E.

#### Description.

***Trees*** 2–20(-32) m tall. ***Leaves*** slightly congested at the branch tips, imparipinnate with 2–3 pairs of leaflets, obtrullate in outline, (4.2–)6.6–8.1 × 2.9–5.1 cm; rachises densely lanate, points of leaflet attachment with a tuft of long hairs; stipular sheaths apically acute with spurs, densely lanate on the outer surfaces; leaflets obovate to broadly obovate in outline, second pair from the terminal leaflet the largest, one of this pair (1.8–)2.2–2.7 × 0.9–1.4 cm; margin crenate with 5–9 teeth, apically emarginate, basally unequally cordate; upper leaflet surfaces sparsely lanate; lower leaflet surfaces densely lanate with whitish hairs 1.4–1.5 mm long. ***Inflorescences*** pendant, (5.0–)6.1–12.3 cm long, bearing 5–7(–11) flowers; floral bracts 4.7–7.5 mm long, narrowly triangular, densely lanate on the outer surface; rachises lanate. ***Flowers*** 8.4–9.4 mm diam.; sepals 4, ovate, green, densely lanate outside; stamens 15–19, anthers orbicular, with a dense tuft of straight white hairs on the upper half; styles fimbriate, 2.5–3.2 mm long. ***Fruits*** turbinate, with 2–4 irregular flattened ridges with a series of spines, densely pilose; 5.5–10.0 × 4.9–7.7 mm including spines. ***Tetraploid***.

#### Distribution, habitat and ecology.

*Polylepislanata* is restricted to the Cordillera Tunari and the adjacent Cotacajes Valley in Cochabamba (Bolivia) (Fig. 92). It occurs at 2800–3970 m elevation in humid Yungas forest where it co-occurs with *P.canoi* in mixed forest with *Alnusacuminata*, *Valleastipularis*, *Prumnopithysexigua*, *Podocarpusglomerata*, *Weinmanniafagaroides* and *Oreopanax* spp. (Kessler 1995b). In the Tunari mountains at 3220–3420 m elevation, *P.lanata* grows in mixed forest dominated by typical Yungas species, such as *Hesperomeleslanuginosa* and *Escalloniamyrtilloides* (Navarro et al. 2005). In general, the Cochabamba region is considered as an important area for Neotropical bird conservation (Wege and Long 1995; Fjeldså and Kessler 1996). This region is one of the areas with the greatest diversity of birds of *Polylepis* forests in Bolivia, including species, such as *Pseudosaltator* (= *Saltator*) *rufiventris* and *Cranioleucapyrrhophia* which occur in drier areas and typical humid Yungas species, such as *Conirostrumferrugineiventre* and *Thlypopsisruficeps*, as well as specialists like *Conirostrumbinghami* (= *Oreomanesfraseri*) (Navarro et al. 2010). Forests of *P.lanata* are often isolated from the closed treeline because ground fires can spread through open *Polylepis* forest, but not through the humid cloud forest (Kessler 2000). An isolated forest patch at 3650 m elevation had a mean tree height of about 20 m, with individual trees reaching 32 m, making this one of the tallest species of *Polylepis* (Hertel and Wesche 2008).

#### Conservation status.

The EOO for *Polylepislanata* is estimated as 5,683 km^2^, the AOO is assessed at 60 km^2^ and it is known from 13 locations. It is protected within Carrasco National Park. The species was categorized as VU (B1+2c, D2) in the World List of Threatened Trees (Oldfield et al. 1998). However, later it was categorized as EN (B1+2ab(ii,iii,iv)) in the Red List of Threatened Flora of Bolivia (Arrázola and Coronado 2012). Logging, firewood, livestock and fire are threats for its ecosystem (Navarro et al. 2010; Arrázola and Coronado 2012). We assess the species as Endangered (B1a+B2a, D2a).

#### Notes.

*Polylepislanata* can be distinguished from the most similar species *P.triacontandra* by having 2–3 versus 1(–2) lateral leaflet pairs, emarginate versus acute to revolute leaflet apices and lanate, 1.4–1.5 mm long versus tomentose, 0.4–0.8 mm long hairs on the lower leaflet surfaces. *Polylepislanata* further has fewer flowers per inflorescence (15–19 versus 21–23) and shorter styles (2.5–3.2 mm versus 3.3–3.8 mm). Occasionally, specimens of *P.lanata* resemble those of *P.pacensis* in having the same number of lateral leaflet pairs, leaflet shape, apex and margins, but it differs in hair length and type (lanate, 1.4–1.5 mm versus villous, 0.4–0.9 mm). *Polylepislanata* has also been confused with *P.sacra* (see *P.sacra*). Hybrids of *P.lanata* with both *P.besseri* and *P.subtusalbida* have been reported by Kessler (1995b).

#### Specimens examined.

**Bolivia. Cochabamba**: Ayopaya, 3 km al N de Saila Pata, 16°54'S, 066°56'W, 3150 m, 14 November 1997, *Kessler 12440* (GOET!); Bosque situado a 5 km, subiendo en dirección Oeste de Independencia, cerca de la comunidad de Kuri Barranca, parte alta, 3500 m, 05 November 1987, *Mérida 4* (GOET!, MO!). Carrasco, Com. Lachujmayu, 3200 m, 22 November 1991, *Hensen 934* (GOET!); Surroundings of Monte Punco, 17°36'S, 065°17'W, 2800 m, 15 August 1991, *Kessler 2956* (GOET!); 3 Km N Cocapata off Cochabamba-Santa Cruz road, 17°34'S, 065°19'W, 3000 m, 15 August 1991, *Kessler 2961* (AAU!, GOET!, MO!); *2962* (GOET!); *2963* (AAU!, GOET!); *2964*; *2965* (GOET!, MO!); *3493* (GOET!); Lope Mendoza, 17°32'58"S, 065°21'58"W, 3453 m, 27 December 1998, *Mercado 1959* (MO!). Cercado, Ladera Sur del Parque Nacional Tunari, 17°05'42"S, 066°19'52"W, 3800 m, *Terán 4421* (BOLV). Chapare, Cantón Colomi 8 km al NW de Colomi, Candelaria Pie de gallo, zona Chimparancho, 17°10'00"S, 065°58'00"W, 3200 m, 23 April 1989, *Beck 18100* (GOET!, MO!); Mayka Mayu, 3300 m, 02 April 1991, *Hensen 2249* (MO!); Candelaria, 3300 m, 04 April 1991, *Hensen 2280* (GOET!); Mayka Mayu, 3300 m, 28 July 1990, *Hensen 850* (GOET!); Mayka Mayu, ca. 60 km N Sacaba, 17°12'S, 065°58'W, 3300 m, 11 August 1991, *Kessler 2850* (AAU!, GOET!); *2851* (AAU!); *2870*; *2871*; *2873*; *2876* (GOET!); *3402* (AAU!, GOET!, LPB, MO!); *3483*; *3484*; *3485*; *3486*; *3487*; *3488* (GOET!); ca. 4 km N Mayka Mayu, ca. 65 km from Sacaba, 17°12'S, 065°58'W, 3350 m, 13 August 1991, *Kessler 2933* (AAU!, GOET!); *3490*; *3491*; *3492* (GOET!); Km 76 camino antiguo a los yungas del Chapare entrando por Aguirre, 24 April 1999, *Mercado 2213* (MO!); 14.7 km N of Colomi (junction of the road to Candelaria) on road to the Chapare, 17°15'S, 065°53'W, 3300 m, 19 October 1985, *Solomon 14421* (LPB, MO!). Vinto, Tunari, 17°19'00"S, 066°21'00"W, 3000–4000 m, *Kuntze s.n* (B, NY). **La Paz**: Inquisivi, Unos 8 km. de Quime hacia Inquisivi, Camillaya arriba del pueblo, 16°58'S, 067°12'W, 3000 m, 29 September 1997, *Beck 24367* (MO!); along the trail and on slopes W of trail, between Pongo Chico and Laguna Naranjani, 16°59'S, 067°15'W, 08 July 1988, *Lewis 881028* (MO!). Irupana, Kakhani, 16°41'00"S, 067°36'00"W, 3322 m, 27 June 2004, *Chumacero 483* (LPB); *484* (LPB); *485* (LPB). Sud Yungas, camino a Lambate, 16°36'10"S, 067°42'25"W, 3386 m, 26 July 2009, *Palabral 670A* (LPB). Cult. at Jardín Botánico La Paz 2000, from seeds collected at Cochabamba: Mayka Mayu 1991, *Kessler 12629* (GOET!).

### ﻿31. Polylepis pacensis

Taxon classificationPlantaeRosalesRosaceae

M.Kessler & Schmidt-Leb., Organisms Diversity Evol. 6(1): 67, f. 1. 2006.

5DE6315D-477C-5F37-B11B-ECF4BCD5EE3B

Figs 83 , 84

#### Type.

Bolivia. La Paz: Murillo, sobre el camino de herradura entre Cohoni y Jalancha, 16.6853°S, 67.8356°W, 3853 m, *Mendez & Arcienga 18* (holotype: GOET!; isotype: LPB!).

**Figure 83.**
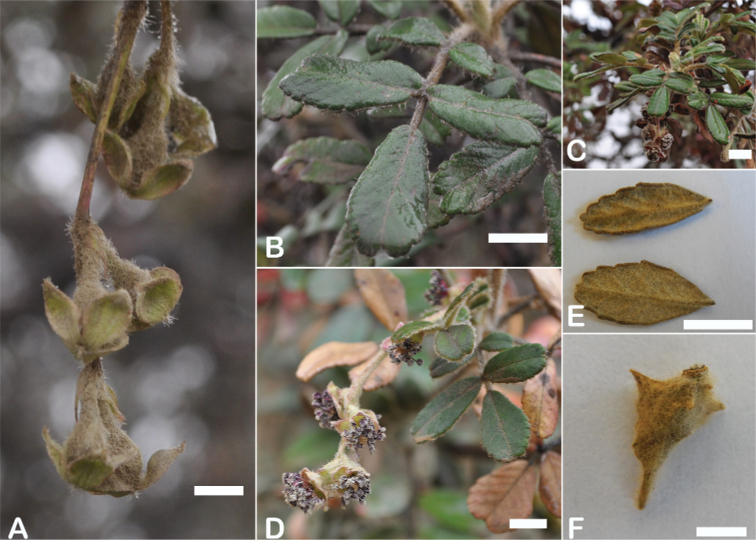
*Polylepispacensis* M.Kessler & Schmidt-Leb **A** fruits **B** upper leaf surface **C** flowering branch **D** flowers **E** lower leaflet surface **F** fruit. Scale bars: 1 cm (**B, C, E**); 5 mm (**D**); 3 mm (**A, F**). Photographs **A–D** A. Domic **E, F** T.E. Boza E.

#### Description.

***Trees*** 3–8 m tall. ***Leaves*** slightly congested at the branch tips, imparipinnate with 2–3 pair of leaflets, obtrullate in outline, 3.4–5.2 × 2.4–3.4 cm; rachises densely villous, points of leaflet attachment with a tuft of long hairs; stipular sheaths apically acute with spurs, densely villous on the outer surfaces; leaflets obovate in outline, second pair from the terminal leaflet the largest, one of this pair 1.7–2.4 × 0.6–0.8 cm; margin crenate, apically emarginate, basally cuneate; upper leaflet surfaces glabrous to sparsely villous; lower leaflet surfaces with an evenly distributed dense layer short white pannose hairs, admixed with villous whitish hairs 0.4–0.9 mm long. ***Inflorescences*** pendant, 3.7–7.7(–10.0) cm long, bearing 5–11 flowers; floral bracts 4.3–5.0 mm long, narrowly triangular, sparsely to densely villous on the outer surface; rachises densely villous. ***Flowers*** 6.5–8.1 mm diam.; sepals 4, ovate, green, densely pannose outside; stamens 17–23, anthers orbicular, with a dense tuft of straight white hairs on the upper half; styles fimbriate, 2.3–2.7 mm long. ***Fruits*** turbinate, with 3–4 irregular flattened ridges with a series of spines, densely pilose; 5.4–7.3 × 4.7–5.6 mm including spines. ***Tetraploid***.

**Figure 84.**
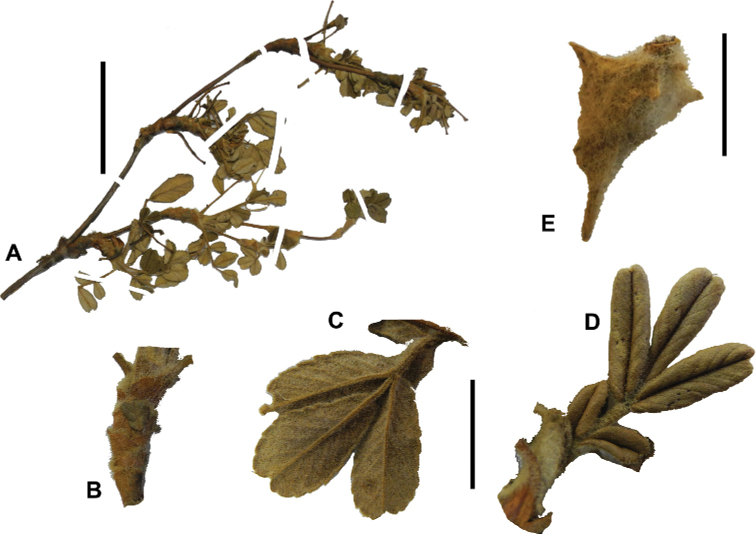
*Polylepispacensis* (Bitter) M.Kessler & Schmidt-Leb **A** flowering branch **B** stipular sheaths **C** lower leaf surface **D** upper leaf surface **E** fruit (**A–C***Beck 17832***D, E***Kessler 3032*). Scale bars: 7 cm (**A**); 2 cm (**C, D**); 5 mm (**E**). Photographs by T. E. Boza E.

#### Distribution, habitat and ecology.

*Polylepispacensis* is distributed in southern La Paz and western Cochabamba Departments (Bolivia) (Fig. 92). It occurs in relatively dry inter-Andean valleys at 3400–4700 m elevation. It co-occurs with *P.triacontandra* on Cerro Illimani (Kessler and Schmidt-Lebuhn 2006). Some forests of *P.pacensis* show an influence of humid Yungas forests, as indicated by the presence of *Oreopanaxmacrocephalus*, *Brachyotummicrodon* and *Phytolaccabogotensis*. In drier areas, close to inaccessible streams with steep slopes and, therefore, unsuitable for crops, *P.pacensis* co-occurs with other native species, such as *Escalloniaresinosa*, *Puya* sp., *Schinusmicrophylla* and *Azorellamultifida* (Hurtado et al. 2018). In a study in Cohoni (La Paz), natural regeneration was abundant and successful in places protected by topography where human and animal access is difficult, with an average of 1312 seedlings/ha. *Polylepispacensis* has a mean germination rate of 8%, which decreases with elevation (Mendez 2005; Vega et al. 2018).

#### Conservation status.

The EOO for *Polylepispacensis* is estimated as 15,486 km^2^, the AOO is assessed at 44 km^2^ and it is known from 44 locations. No conservation actions have been taken to date. It has been categorized as EN (B1ab(i,ii)) (Arrázola and Coronado 2012). Based on the small size of the remaining populations, the extraction of firewood represents a major threat. We assess *P.pacensis* as Endangered (A2b, B1a+B2a, C1).

#### Notes.

*Polylepispacensis* was considered by Simpson (1979) to belong to the broadly defined *P.racemosa*. Kessler (1995b) was unable to place the few specimens available at that time under either *P.triacontandra* (which occurs mainly to the north) and *P.lanata* (southwards). It was only when new herbarium collections were gathered that the taxonomic distinctness of the species became apparent (Kessler and Schmidt-Lebuhn 2006). *Polylepispacensis* is similar to *P.triacontandra*, but has 2–3 pairs of obovate leaflets with villous hairs, whereas the latter has 1(–2) pairs of narrowly elliptic leaflets with tomentose hairs. *Polylepispacensis* also has fewer flowers per inflorescence (5–11 versus 11–13) and shorter styles (2.3–2.7 mm versus 3.3–3.8 mm). For additional morphological similarities, see under *P.lanata*.

#### Specimens examined.

**Bolivia. Cochabamba**: Ayopaya, Piusilla, 3300 m, 30 March 1991, *Hensen 2134* (GOET!, LPB). **La Paz**: Bautista Saavedra, 2 kms. arriba de Chajaya, Quebrada de Janajj Wayq’o, 15°11'53"S, 069°00'14"W, 3740 m, 04 August 1985, *Beck 11350* (LPB, MO!). Inquisivi, Quime 7 km hacia Caxata, 3420 m, 19 February 1981, *Beck 4378* (LPB); 8 km W Quime on road to Caxata, 17°03'00"S, 067°17'00"W, 3350 m, 24 August 1991, *Kessler 3029* (AAU!); *3030* (AAU!, GOET!, MO!); *3031* (GOET!, MO!); *3032* (AAU!, GOET!, MO!). Murillo, Palca 28.5 km hacia Cohoni, 3440 m, 14 October 1990, *Beck 17832* (GOET!, LPB); aprox. 14 km de población Cohoni (Municipio de Palca) sobre ruta Cohoni–Cayimbaya, 16°41'51"S, 067°00'00"W, 3586 m, 10 November 2014, *Bermejo PP 2 S5* (LPB); Subcuenca de Cohoni, camino de herradura Cohoni–Jalancha, 16°39'36"S, 067°49'12"W, 4989 m, 05 January 2003, *Mendez 1*; *2* (LPB); *3* (GOET!, LPB); *4*; *5* (LPB); *6* (GOET!, LPB); *7*; *8*; *9*; *10*; *11*; *12*; *13* (LPB); *14* (GOET!, LPB); *15* (LPB); Sobre el camino carretero a 6 km de Cohoni hacia Palca, 16°40'48"S, 067°51'00"W, 3457 m, 05 January 2003, *Mendez 16* (GOET!, LPB); Sobre el camino de herradura entre Cohoni y Jalancha, 16°40'48"S, 067°49'48"W, 3856 m, 13 November 2003, *Mendez 17*; *18*; *19* (GOET!, LPB); Sobre el camino de herradura entre Cohoni y Jalancha, 16°40'12"S, 067°49'48"W, 3879 m, 13 November 2003, *Mendez 20* (GOET!, LPB); Subcuenca de Cohini, Cohoni lado de la Jalancha (caida de agua), 16°39'36"S, 067°49'12"W, 4173 m, 13 November 2003, *Mendez 21* (GOET!, LPB); ciudad de La Paz, Zona La Portada, Av. Final Kollasuyo interseccion con Av. Naciones Unidas, Plaza La Portada. Jardinera de plaza, 16°29'13"S, 068°09'57"W, 3995 m, 04 March 2016, *Vega MUNAY 1* (LPB). Palca, Catagna au pied S I’Illimani, 4511 m, *Pentland 47* (GOET!). Quime, about 10 km from Quime on the road to Caxata, 17°00'S, 067°12'W, 3400 m, 25 April 1987, *Brandbyge 769* (AAU!).

### ﻿32. Polylepis racemosa

Taxon classificationPlantaeRosalesRosaceae

Ruiz & Pav., Syst. Veg. Fl. Peruv. Chil. 139. 1798.

72D4D2ED-1163-59ED-9C98-BFAA371B9271

Figs 85 , 86

Polylepis villosa Humboldt, Bonpland and Kunth, Nov. Gen. Sp. (quarto ed.) 6: 228. 1824. Type. Peru. Caxamarca: 8784 ft. (2700 m), Aug 1802, *Bonpland 3685* (holotype: P!; isotype: P!).
Polylepis incana subsp icosandra Bitter, Bot. Jahrb. Syst. 45: 641. 1911. Type. Peru. Ancash: Cajatambo, between Tallenga and Piscapaccha, *Weberbaueri 2886* (holotype: B destroyed).
Polylepis incana subsp. micranthera Bitter, Bot. Jahrb. Syst. 45: 642. 1911. Type. Peru. Huanúco: Sierra, Caxamarquilla, without collector (holotype: W!).
Polylepis incana var. primovestita Bitter, Bot. Jahrb. Syst. 45: 645. 1911. Type. Peru. Caxamarquilla: *Ruiz s.n* An illegitimate name since it is based on the same collection as the type of P.racemosa.
Polylepis incana var. connectens Bitter (1911:645). Type. Peru, Huanúco: mountains of Huanúco, *Haenke s.n* (holotype: PR).

#### Type.

Peru. Caxamarquilla and Pillao *Ruiz s.n* (holotype: P!; isotype: G!).

**Figure 85.**
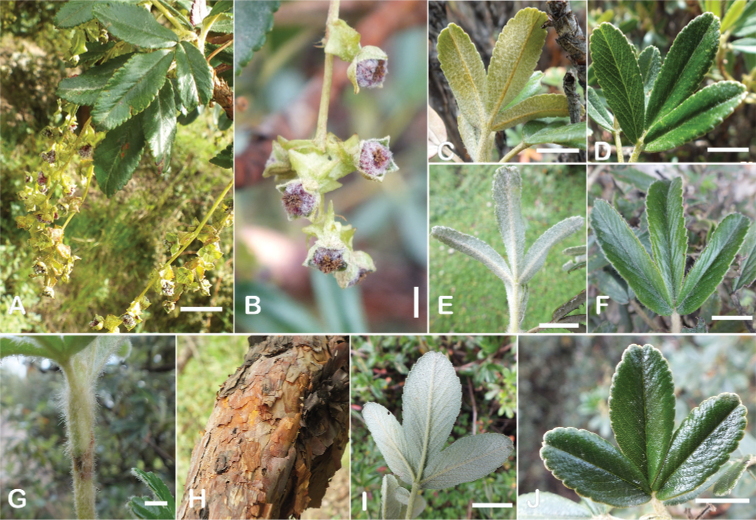
*Polylepisracemosa* Ruiz & Pav **A** flowering branch **B** flowers **C** lower leaflet surface **D** upper leaflet surface **E** lower leaflet surface **F** upper leaflet surface **G** stipule sheaths **H** bark **I** lower leaflet surface **J** upper leaflet surface (**A, B, H–J***Boza & Urquiaga 3030*. Cusco **C, D***Boza & Urquiaga 3020*. Ancash **E–G***Boza & Urquiaga 3118* Cajamarca). Scale bars: 1 cm (**A, C–F, I–J**); 5 mm (**B**); 2 mm (**G**). Photographs by E. G. Urquiaga F.

#### Description.

***Trees*** 2–15 m tall. ***Leaves*** slightly congested at the branch tips, imparipinnate with 1–2 pairs of leaflets, obtrullate in outline, (3.9–)4.5–5.8(–6.5) × (2.6–)3.1–4.6(–6.1) cm; rachises densely villous, points of leaflet attachment with a tuft of long hairs; stipular sheaths apically acute, glabrescent on the outer surfaces with long trichomes at the apex; leaflets obovate in outline, second pair from the terminal leaflet the largest, one of this pair (2.3–)3.1–3.9 × (0.7–)0.9–1.5 cm; margin serrate with 8–14 teeth, apically round, basally attenuate to unequally cordate; upper leaflet surfaces glabrous; lower leaflet surfaces sparsely to densely tomentose with yellowish hairs 0.4–1.5(–2.0) mm long. ***Inflorescences*** pendant, (4.2–)5.0–9.4(–11.7) cm long, bearing 7–21 flowers; floral bracts (3.8–)4.6–6.0 mm long, narrowly triangular, densely tomentose on the outer surface; rachises villous. ***Flowers*** (6.6–)7.8–8.1(–11.9) mm diam.; sepals 4, ovate, green, densely villous outside; stamens 9–19, anthers orbicular, with a dense tuft of straight white hairs on the upper half; styles fimbriate, 2.2–3.7 mm long. ***Fruits*** turbinate, with 2–5 irregular ridges with a series of spines, densely pilose; (4.2–)5.0–8.1(–10.3) × (3.3–)3.8–6.0 mm including spines. ***Tetraploid*** to ***octoploid*** with many intermediates.

**Figure 86.**
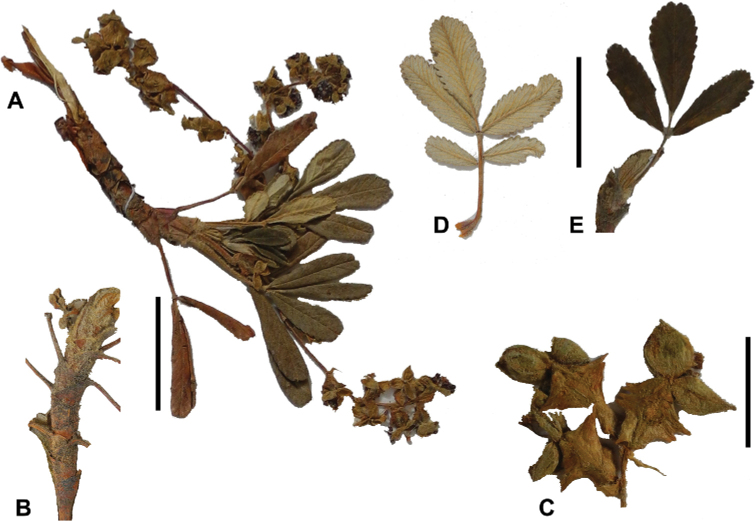
*Polylepisracemosa* Ruiz & Pav **A** flowering branch **B** stipular sheaths **C** fruit **D** lower leaf surface **E** upper leaf surface (**A, C***Vargas 186***B, E***Vargas 59***D***Vargas 58*). Scale bars: 4 cm (**A**); 10 mm (**C**); 3 cm (**D, E**). Photographs by T. E. Boza E.

#### Distribution, habitat and ecology.

*Polylepisracemosa* has been recorded from Cajamarca to Cusco in Peru and, in recent decades, has been introduced to Ecuador from Pichincha to Azuay (Fig. 92). It has been recorded at 2750–4660 m elevation. To our knowledge, all records of this species refer to planted individuals, mostly close to villages and houses. *Polylepisracemosa* is probably the fastest growing species of the genus (Montalvo et al. 2018; Rosero et al. 2018), presumably as a result of hybridogenic polyploidy and associated hybrid vigor. This species is currently being widely used in reforestation programmes in the Andes of Ecuador and Peru.

#### Conservation status.

The EOO for *Polylepisracemosa* is estimated as 461,319 km^2^, the AOO is assessed at 128 km^2^ and it is known from 24 locations. The species was categorized as VU (A1c) in the World List of Threatened Trees (Oldfield et al. 1998). In Peru, it has been categorized as CR (SERFOR 2006). This species is fast-growing, more ecologically adaptable than others in the genus and extensively used for reforestation and agroforestry. We assess *P.racemosa* as Least Concern (B1a+B2a), unless a natural population is found, which would then be of high conservation concern.

*Polylepisracemosa* has been observed to hybridize with other species of *Polylepis*, potentially threatening the genetic integrity of natural *Polylepis* populations. This process is especially apparent in Ecuador, where the species has, in recent decades, been widely planted even in conservation areas, such as Cajas National Park where first generation hybrids with all four native species can now be seen (Caiza et al. 2021). We strongly argue against using this species for afforestation projects.

#### Notes.

*Polylepisracemosa* can be distinguished from the most similar species *P.acomayensis* by having larger leaflets (2.3–3.9 × 0.7–1.5 cm versus 1.6–2.3 × 0.5–0.9 cm), serrate versus crenate leaflet margins, tomentose, 0.4–2.0 mm long versus villous, 0.9–1.1 mm long hairs on the lower leaflet surfaces, inflorescences 4.2–11.7 cm with 7–21 flowers versus 2.0–4.0 cm with 5–7 flowers and 9–19 versus 19–25 stamens. Occasionally, specimens of *P.racemosa* resemble those of *P.triacontandra* in having the same number of lateral leaflet pairs and same lower leaflet surface hair type (tomentose), but it differs in leaflet margin (serrate versus crenate), leaflet apices (round versus acute to revolute), lower leaflet surface hair length (0.4–2.0 mm versus 0.4–0.8 mm), number of flowers per inflorescence (7–21 versus 11–13) and number of stamens (9–19 versus 21–23).

**Figure 87.**
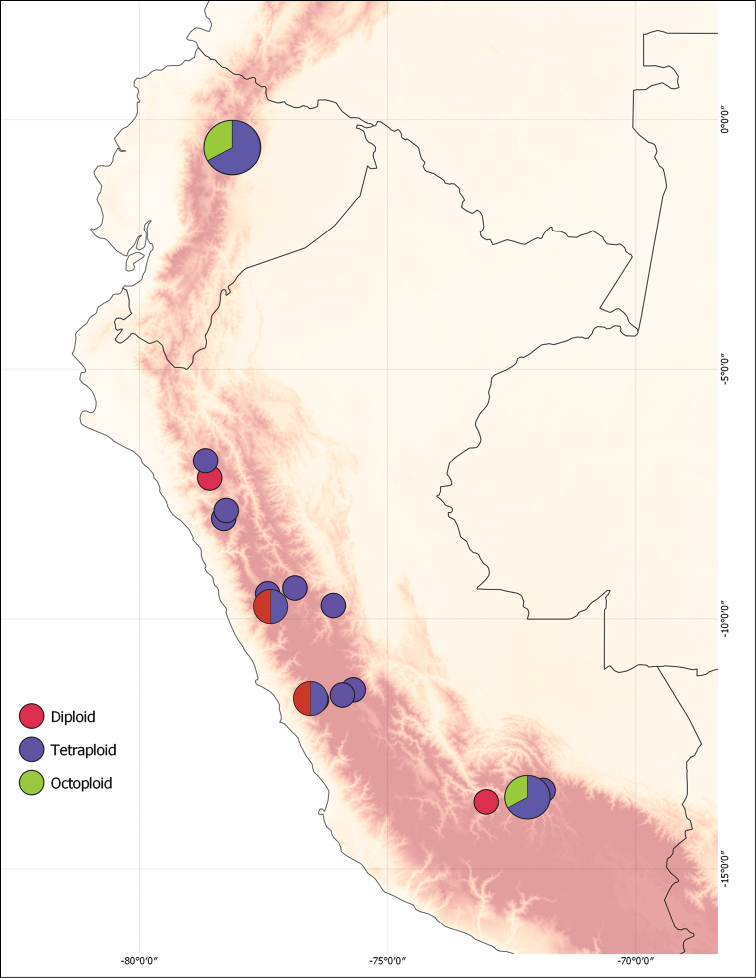
Geographical distribution of the ploidy levels in *Polylepisracemosa*. Pie chart sizes represent number of individuals studied: 1, 2, 3, >5 indiv.

*Polylepisracemosa* is the taxonomically most enigmatic species of the genus. No natural populations are known, but it is very fast growing and the most commonly cultivated species of the genus. Various ploidy levels have been documented in the species, with tetra- and octoploids dominating, but there are also indications of diploids and aneuploids (Fig. 87). We recognize three distinct morphotypes in this species: type A from Ecuador and northern Peru with leaflets 2.9–3.6 × 0.9–1.2 cm and lower leaflet surfaces densely tomentose with 1.5–2.0 mm long hairs (Fig. 85E, F), type B mostly from central Peru with leaflets 2.3–2.9.0 × 0.8–0.9 cm and lower leaflet surfaces sparsely tomentose with 0.8–1.1 mm long hairs (Fig. 85C, D) and type C mostly from southern Peru with leaflets 3.1–3.9 × 1.2–1.5 cm and lower leaflet surfaces sparsely tomentose with 0.4–0.5 mm long hairs (Fig. 85I, J).

We hypothesize that *P.racemosa* is a species of hybridogenic origin that had already been adopted by prehispanic Andean inhabitants because of its fast growth. It is unknown whether the original hybrid or hybrids originated naturally or because the parent species were cultivated in close proximity, as has been shown for species of *Leucaena* (Leguminosae) in Mexico (Hughes et al. 2007). Based on the relatively low ploidy level (tetra- and possibly diploids) and morphological intermediacy, we propose that type B may be closest to the ancestral hybrid and that *P.fjeldsaoi* or *P.incana* on the one hand and *P.flavipila* on the other hand are possible parent taxa. Once the species was widely cultivated, there probably were additional hybridization and polyploidization events, leading to the morphological and cytological variability seen today. Translocations of individuals between different human population centers would then lead to the complex present-day geographical distribution of the forms.

#### Specimens examined.

**Ecuador. Azuay**: Cuenca, carretera Molleturo, Naranjal, 3778 m, 21 August 2008, *Romoleroux 5155* (QCA!). **Chimborazo**: Riobamba, Parroquia Licto, comuna Molobog, vía Riobamba-Licto, 01°46'S, 078°36'W, 2900 m, 30 November 2004, *Caranqui 1303* (CHEP). **Pichincha**: Cayambe, Parroquia El Quinche, comunidad Chumillos, sitio arqueológico Quito Loma, a 10 km al este de El Quinche, 00°06'16"S, 078°12'43"W, 3650 m, 26 August 2009, *Asanza 2088* (QCNE). Papallacta, Papallacta along track from Termas de Papallacta to entrance of Cayambe-Coca NP, 00°21'33"S, 078°08'58"W, 3350 m, 04 July 2014, *Kessler 14608* (Z!).

**Peru. Ancash**: Casma, El Tambo, 3600 m, 25 August 1978, *Mostacero 531* (MO!). Huaraz, Quebrada Quillcayhuanca, 3620 m, 30 October 1989, *Arce 161* (MO!); Q. Quillcayhuanca, 3650 m, 30 October 1989, *Arce 164* (MO!); en vivero forestal de Yanama, procedencia: Qilllcayhuanca, 15 December 1989, *Arce 183* (MO!); Yupa, E of Huaraz on road to Pitec, agricultural land, trees along edge of fields, 09°31'S, 077°28'W, 3700 m, 22 May 1985, *Smith 10701* (AAU!, MO!, QCA!, USM!); just below the summit of Huaraz-Casma road, 09°33'S, 077°34'W, 3900 m, 05 June 1985, *Smith 10846* (MO!, USM!); plants purchased in the weekly herb market, the Thursday market, 09°31'S, 077°31'W, 4500 m, 05 June 1986, *Smith 12449* (MO!). Huari, en vivero forestal de Yanama, procedencia: Sapsha, en el Dist. de Chacas, 15 December 1989, *Arce 184* (MO!). Independencia, Pitec, Coordillera Blanca, 09°30'S, 077°30'W, 3800 m, 11 August 1988, *Frimer 10* (AAU!). Recuay, Cátac, cerca a Querococha, 09°42'57"S, 077°18'45"W, 4185 m, 08 November 2014, *Boza & Urquiaga 3020* (USM!, Z!); Laguna Querococha, 09°40'33"S, 077°19'54"W, 4000–4100 m, 11 June 2002, *Cano 12412* (MO!, USM!); Dist. Marca, 3600 m, 13 June 1998, *Gamarra 816* (USM!); Conococha-Pativilca, near Puente Santa Rosa, roadside slope, 4000 m, 16 August 2002, *Schmidt-Lebuhn 520* (GOET!, USM!); Huascarán National Park, sector Querococha, alluvial fan at W end of lake, 09°44'S, 077°20'W, 3950–4080 m, 05 July 1985, *Smith 11076* (MO!, USM!). San Marcos, the town of Chavin, Cordillera Blanca, 09°35'S, 077°10'W, 3150 m, 02 October 1988, *Frimer 116* (AAU!); the town of Chavin, Cordillera Blanca, 09°35'S, 077°10'W, 3150 m, 02 October 1988, *Nielsen 116* (AAU!). Ticapampa, Cord. Blanca Western end of Lag. Querococha, on road between Huaraz and Chavín, western side of pass, 09°42'S, 077°20'W, 4000 m, 17 February 1987, *Boertmann 71* (AAU!, QCA!). from Huaraz towards Casma, 3500 m, 04 April 1988, *Renvoize 5052* (AAU!). **Apurimac**: Andahuaylas, Dist. Pampachiri, 3700 m, 01 January 2004, *Vargas 186* (USM!). Carhuacahua, Pacachacas Valley, 3200 m, 07 November 1935, *West 3787* (MO!). **Ayacucho**: Huamanga, Quinua, centro urbano de Quinua, 3250 m, 01 May 2013, *Hurtado JAHH030* (USM!); Dist. de Quinua, centro urbano de Quinua, 3270 m, 01 May 2013, *Hurtado JAHH031* (USM!); Dist. Acocro; alrededor de Juiza, 09 April 2002, *Vargas 103* (USM!). La Mar, Laguna de Toctococha, 12°58'09"S, 074°00'00"W, 4200 m, 30 September 2003, *Mendoza 1015* (MO!); Aypacorral, camino a Uras. Dist San Miguel, 3100–3180 m, 04 April 2005, *Roque 4717* (USM!); Dist. Luis Carranza, alrededor de Sacsamarca, 22 February 2001, *Vargas 58* (USM!); Dist. Luis Carraza, alrededor de Sacsamarca, 22 February 2001, *Vargas 59* (USM!). Vilcas Huaman, Dist. Vilcashuaman, Cerro Anahuarje subiendo por Allaspina y Churiaccaza, 3800 m, 27 September 2004, *Baldeón 6141* (USM!); Dist. Vilcashuaman, Qapap Ñan (Camino Inca) por Viscachayoc, 13°39'18"S, 073°56'13"W, 3650–3680 m, 29 September 2004, *Baldeón 6159* (USM!); cerca del puente Rumichaca II, en la carretera Los Libertadores, Dist. Pilpichaca, 26 June 2001, *Roque 3328* (USM!). **Cajamarca**: Celendin, Celendin-Cajamarca km 28, cultivated as field border post, 08 August 2002, *Schmidt-Lebuhn 490* (GOET!, USM!); Celendin-Cajamarca rd, 27–40 km from Celendin, 07°02'S, 078°14'W, 3200–3400 m, 25 February 1984, *Smith 6247* (MO!, UMO, USM!). Encañada, Pampa del Toro on the road between Cajamarca and Celendin, 07°02'S, 078°16'W, 3400 m, 15 February 1987, *Brandbyge 99* (AAU!). Hualgayoc, Hualgayoc, 06°53'16"S, 078°40'05"W, 3683 m, 31 May 2015, *Boza & Urquiaga 3008*; *3118*; *3119* (USM!, Z!); Chugur, sobre la ruta a Perlamayo, 2950–3000 m, 20 March 1988, *Sánchez 4679* (AAU!). Magdalena, near Cumbe Mayo, 07°11'S, 078°35'W, 3500–3650 m, 26 January 2003, *Lægaard 22233* (AAU!). San Marcos, Bajada de la Totorilla, siguiendo el curso del Río Shitmalca, 3550 m, 31 October 1992, *Sánchez 6412* (F!, QCA!). San Miguel, Cajamarca-Bambamarca road, about 60 km from Cajamarca, 06°50'S, 078°40'W, 3800 m, 17 February 1983, *Smith 3499* (F!, MO!, USM!). San Pablo, Tumbaden, camino a Vista Alegre, entre la escuela de Antevo y Vista Alegre, 06°58'15"S, 078°42'59"W, 3300 m, 15 January 2004, *Sánchez 12517* (US!), 2687 m, August 1802, *Humboldt 3685* (B, F!, MO!); Cajamarca to Bambamarca, side road to Chugur (95 km from Cajamarca), 20 March 1988, *Renvoize 4846* (AAU!); Micuypampa, 66 km from Cajamarca towards Celendin, 3450 m, 26 1988, *Renvoize 4951* (AAU!); Cumbe Mayo. 16 km from Cajamarca, 3500 m, 27 March 1988, *Renvoize 4980* (AAU!); entre Cajamarca y Cumbe Mayo, km 13, 3200 m, 11 June 1990, *Sánchez 5322* (F!). **Cusco**: Cusco Sacsayhuaman, 13°30'35"S, 071°58'43"W, 3391 m, 11 June 2015, *Boza & Urquiaga 3030* (USM!, Z!); Cusco Tambomachay, 13°30'35"S, 071°58'43"W, 3550 m, 11 June 2015, *Boza & Urquiaga 3031* (USM!, Z!); Dist. Cusco, Chacan, 13°30'06"S, 071°59'03"W, 3500–3600 m, 21 March 2003, *Calatayud 1247* (CUZ!, F!, MO!); 100m von FuSptad nach Sacsayhuaman, 3600 m, 20 August 1989, *Kessler 391* (GOET!, Z!); Sacsayhuaman, 3600 m, 07 July 1999, *Lægaard 20465* (AAU!); Chacan S of Cusco, 13°29'S, 072°00'W, 3900 m, 10 February 2003, *Lægaard 22351* (AAU!); *22352* (AAU!); cultivated on the Plaza de Armas, 13°32'S, 071°59'W, 3400 m, 16 August 2000, *Schmidt-Lebuhn 39* (GOET!); cultivated on the Plaza de Armas, 13°32'S, 071°59'W, 3400 m, 22 August 2000, *Schmidt-Lebuhn 40* (GOET!); Calca, Taray Chitapampa, a 16 km de Cusco en la carretera Cusco-Urubamba, 3500 m, 16 May 1987, *Núñez 8117* (MO!, USM!). Chumbivilcas, Río de Velille, 3850 m, 18 December 1962, *Vargas 14090* (CUZ!). Cusco, C’orao, 3653 m, 01 May 2003, *Arce s.n* (USM!); Miskahuara (margenes del río), 3300 m, 01 February 1970, *Vargas 21978* (CUZ!); Pumamarca, 3400 m, 11 May 1946, *Vargas 6044* (CUZ!). Espinar, Yauri Virginniyoc, ca. 35 km de Yauri, camino de Yauri, puente viejo, Maucallacta hacia Suicutambo, por carretera y caminos de Herradura, 4100 m, 13 April 1987, *Núñez 7889* (MO!, USM!). Urubamba, Chicon, 3517 m, 01 April 2003, *Arce s.n* (CUZ!); Chincheros, around community of Taucca, 13°25'S, 072°00'W, 4050 m, 14 January 1982, *Davis 1578* (A!, USM!); Tankarpata, above Cusco airport, valley between dry steep hillsides, 13°31'S, 071°58'W, 3500 m, 23 July 1983, *Gentry 43217* (F!, MO!, USM!); Dist. Huayllabamba, entre Huayoccari y las lagunas de Yanacocha y Kellococha, 13°13'S, 072°16'W, 2900–4600 m, 17 July 1989–18 July 1989, *Tupayachi 1139* (MO!); Dist. Huayllabamba, Quebrada de Huayoccari-Laguna de Yanaccocha, 13°21'15"S, 072°03'55"W, 2900–3860 m, 21 December 1988, *Tupayachi 808* (MO!). 3300–3800 m, 19–21 May 1958, *Humbert 1958* (US!); San Sebastián, 25 March 1988, *Velásquez 12* (MO!). **Huánuco**: Huamalies, 17.5 km N of Llata by Singa, 09°23'S, 076°52'W, 3600 m, 16 September 1965, *Bird 1383* (MO!), Lauricocha, Jesus, río Lauricocha, ladera, Dist. San Miguel de Cauri, 3326 m, 08 August 2003, *Salvador 541a* (USM!). **Junín**: Huancayo, Concepción, 20 September 1982, *Aguilar s.n* (USM!); Pampas Valley, 3300 m, 24 May 1948, *Anderson 695* (US!); Chongos al sur de Huancayo, 3500 m, 24 March 1983, *Castañeda s.n* (USM!); Cerca a Huancayo, 3800 m, 01 July 1957, *Ferreyra 12418* (MO!, USM!); 3400 m, 03 December 1960, *Kunkel 5626* (GOET!); Huayucachi, 3260 m, 05 January 1974, *Rosales 5* (USM!); 3317 m, 01 March 1945, *Soukup 2720* (F!); Quilcas, alrededores de Shuckto-Loma, 3500 m, 09 January 1994, *Yarupaitán 1154* (USM!). Jauja, Common along at stream in sheltered valley east of Concepcion; foliage bluish-green, 3500 m, 09 January 1945, *Hodge 6221* (A!, US!); Quinual Dist. Mito, 3295 m, 14 August 1974, *Rosales s.n* (USM!). Junín, collected near Chongos Bajo, 3300 m, 19 February 1974, *Antunez de Mayolo 18* (USM!); Tarma, Huacapo near Tarma, 11°25'S, 075°42'W, 4030 m, 01 February 1987, *Boertmann 13* (AAU!); Cerca a cumbre entre Tarma y Oroya, 3500–3600 m, 29 June 1948, *Ferreyra 3792* (MO!, US!, USM!); Casa Blanca cerca de Tarma verdosas, 3300–3350 m, 08 April 1952, *Ferreyra 8259* (MO!, USM!); along shaded stream bank, 3000–3200 m, 20–22 April 1929, *Killip 21899* (A!, F!, US!); Palcamayo, 3000 m, 09 March 1960, *Kunkel 4439* (GOET!); 6 km north of Jauja on the road to Tarma, 07 January 1977, *Simpson 8544* (USM!). Yauli, La Oroya, 11°32'S, 075°53'W, 3700 m, 07 January 1983, *Smith 2973* (MO!, USM!), between La Quinua and Huariaca, Estancia La Aurora, rivershore, 3300 m, 24 June 1940, *Asplund 11944* (US!); Ataura, cerca Jauja, *Mucha s.n* (MO!, USM!); Runatullo, 3500 m, 01 July 1940, *Ridoutt 11775* (USM!); Tingopacha along a stream runing through the village, 07 January 1977, *Simpson 8543* (US!, USM!); Km 72 along 3-S from Jauja to La Oroya, 4 km south of Parco along the fence of a house, 09 January 1977, *Simpson 8549* (US!, USM!); North of Junin near a house along the road, 11 January 1977, *Simpson 8556* (USM!); Palca, 01 August 1947, *Soukup 3498* (F!). **La Libertad**: Otuzco, Ruta Salpo-Samne, 3200 m, 27 May 1993, *Leiva 741* (MO!, QCA!). Sanchez Carrion, Trujillo-Huamachuco road, about 10 km W of Huamachuco, 07°50'S, 078°15'W, 3400 m, 14 February 1983, *Smith 3354* (MO!, USM!). Santiago de Chuco, Quiruvilca, margen derecha del río Chuyunhual, 3620 m, 02 May 2003, *Cano 13106* (USM!); *13110* (USM!); Dist. Quiruvilca. El Sauco, 3605 m, 16 June 2013, *Gonzáles 2286* (USM!); Los Quinuales (al norte de Quiruvilca), 3775 m, 24 March 1994, *Leiva 1090* (F!, QCA!); Sauca (Santiago de Chuco), 3300 m, 16 June 1984, *Sagástegui 11941* (MO!). Trujillo, purchased in the Mayorista Market. Medicinal plant area, 08°07'S, 079°01'W, 12 January 2010, *Meyer 881* (MO!); purchased at Mercado Mayorista, 08°07'S, 079°01'W, 23 June 2008, *Rothrock 3* (MO!). **Lima**: Canta, Lachaqui, 05 October 1989, *Arce 101* (MO!); Lachaqui, camino al cerro Llamarume, 3850 m, 11 September 1991, *Vilcapoma 1202* (USM!). Chicla, Chicla on Route 20 from Lima to La Oroya, 12 January 1977, *Simpson 8560* (A!, MO!, USM!). Huarochiri, Carampoma, 3876 m, 18 November 1989, *Arce 167* (MO!); Vista Alegre, Río Blanco, 3600 m, 21 February 1990, *Arce 204* (MO!); San Juan de Tantaracnche, 3500 m, 22 March 1990, *Arce 207* (MO!). Oyon, Oyon, 3600–3700 m, 25 June 2000, *Cano 10818* (USM!); Cacray towards la Oroya, *Weberbauer 220* (F!). **Pasco**: Pasco, La Quinua, 3540 m, 01 December 1986, *Rivas s.n* (USM!); between Huanuco and Pampayacu, eastern side, 3657 m, 04 February 1927, *Kanehira 5* (A!, F!). 1839–1840, *Gay s.n* (USM!); San Buena Ventura, *Née s.n* (F!); 1778–1788, *Rodríguez s.n* (F!); 1779–1854, *Ruiz s.n* (G, MO!); entre Tarma y Oroya a 10 km de Tarma, 3600–3700 m, 30 June 1954, *Tovar 2371* (MO!).

### ﻿33. Polylepis sacra

Taxon classificationPlantaeRosalesRosaceae

T.Boza & M.Kessler sp. nov.

448EED94-117B-5083-8147-27C320F860A8

urn:lsid:ipni.org:names:77301648-1

Figs 88 , 89

#### Diagnosis.

This species differs from *Polylepislanata* (Kuntze) M.Kessler & Schmidt-Leb. in having slightly narrower leaflets, crenate leaflet margins with more teeth per side, shorter inflorescences with more stamens and longer styles. Further, it also has a distinct geographical distribution with a very different climatic niche.

**Figure 88.**
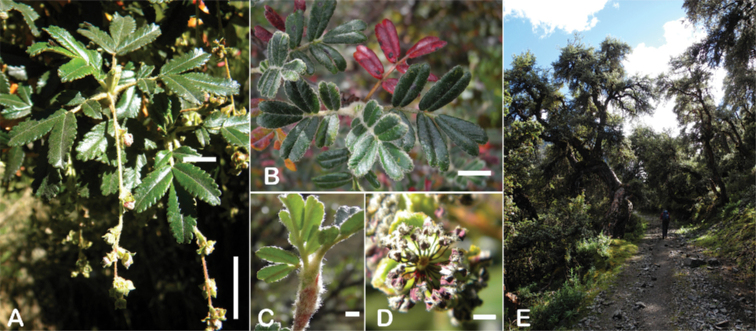
*Polylepissacra* T.Boza & M.Kessler **A** flowering branch **B** leaves **C** stipule sheaths **D** flower **E** habit. Scale bars: 2 cm (**A**); 1 m (**B, C**); 2 mm (**D**). Photographs by T.E. Boza E.

#### Type.

Peru. Cusco: Urubamba, Mantanay, 13°12'03"S, 072°09'20"W, 3350–3800 m, 7 Sep 2002, *J. Farfan et al. 281* (holotype: CUZ!; isotypes: AMAZ, HUT, MO!, USM!).

**Figure 89.**
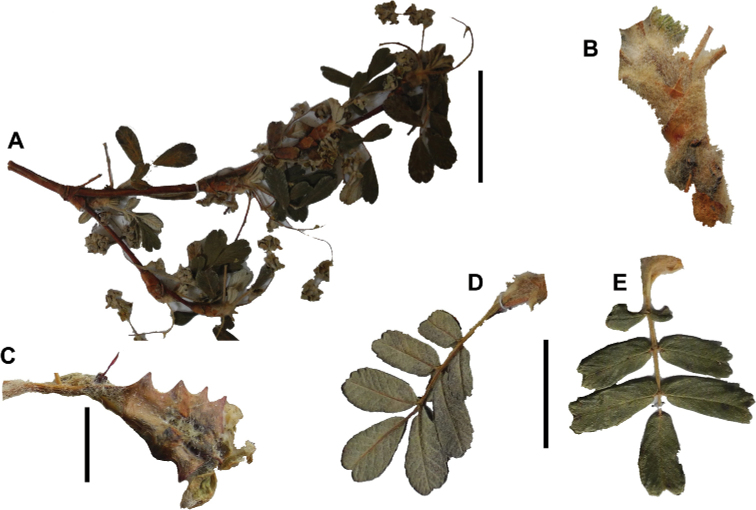
*Polylepissacra* T.Boza & M.Kessler **A** flowering branch **B** stipular sheaths **C** fruit **D** lower leaf surface **E** upper leaf surface (**A***Arce s.n***B, C***Tupayachi 137***D***Toivonen 44***E***Toivonen 8*). Scale bars: 8 cm (**A**); 7 mm (**C**); 3 cm (**D, E**). Photographs by T. E. Boza E.

#### Description.

***Trees*** 4–20 m tall. ***Leaves*** slightly congested at the branch tips, imparipinnate with 2–3 pair of leaflets, obtrullate in outline, (4.1–)5.1–7.1 × 2.0–4.9 cm; rachises densely lanate, points of leaflet attachment with a tuft of long hairs; stipular sheaths apically truncate, densely lanate on the outer surfaces; leaflets obovate in outline, second pair from the terminal leaflet the largest, one of this pair 1.6–2.6 × 0.6–1.1 cm; margin crenate with 6–11 teeth, apically emarginate, basally unequally cordate; upper leaflet surfaces sparsely lanate; lower leaflet surfaces densely lanate with whitish hairs 1.3–1.5 mm long. ***Inflorescences*** pendant, 5.0–8.8 cm long, bearing 5–11 flowers; floral bracts 5.0–7.5 mm long, narrowly triangular, densely lanate on the outer surface; rachises glabrous to villous. ***Flowers*** 8.2–11.0 mm diam.; sepals 4, ovate, green, densely lanate outside; stamens 23–27, anthers orbicular, with a dense tuft of straight white hairs on the upper half; styles fimbriate, 3.3–4.1 mm long. ***Fruits*** turbinate, with 2–3 irregular flattened ridges with a series of spines, densely pilose; (7.3–)10.3–15.1 × 6.0–8.2 mm including spines. ***Octoploid***, perhaps also ***tetraploid***.

#### Distribution, habitat and ecology.

*Polylepissacra* is distributed from central Peru in Huarochiri (Lima) and northern Junín to southern Peru in Abancay (Apurimac) and Cusco (Fig. 92). The species occurs in relatively dry valleys at 3300–4600 m elevation. It often co-occurs with *P.subsericans* and *P.rodolfovasquezii* in the Vilcanota Mountain range (Cusco), but generally occurs at lower elevations than these two species. At 4300 m elevation, a stand of *P.sacra* had a mean maximum tree height of 18.5 m, whereas a stand at 3980 m was only 15.9 m tall, presumably because of drier conditions in the arid valley bottom (Kessler et al. 2014). Average morphological and physiological features of *P.sacra* estimated at Vilcanota are a leaf area of 6.27 cm^2^, specific leaf area of 81.20 cm^2^ g^-1^, leaf thickness of 396.84 μm, epidermal cell length of 33.58 μm, chlorophyll parenchyma cell length of 95.77 μm, 11.02 stomata/mm^2^, rhytidome thickness of 3.75 mm, water content of 16.3%, relative water content of 77.6% and water saturation deficit of 22.1% (Arroyo 2015, as *P.racemosa*). The Cordillera Vilcanota has a high floristic diversity with over 144 plant species (Servat et al. 2002). The *Polylepis* forests in the region hold up to 60 species of birds (Yanacocha) (Servat et al. 2002) with a high number of endemic bird species (Fjeldså et al. 1999; Fjeldså 2002a) including the endangered species *Cinclodesexcelsior*, *Anairetesalpinus* and *Leptasthenuraxenothorax* and the near-threatened species *Leptasthenurayanacencis*, *Asthenesurubambensis*, *Conirostrumbinghami* (= *Oreomanesfraseri*) and *Xenodacnisparina* (Servat et al. 2002).

#### Etymology.

We name this species after the Sacred Valley of the Incas stretching from Pisac to Ollantaytambo in Cusco (Peru) where the species is well distributed.

#### Conservation status.

The EOO for *Polylepissacra* is estimated as 62,397 km^2^, the AOO is assessed at 120 km^2^ and it is known from 15 locations. In Cusco, it is protected within the Private Conservation Areas Network of the Vilcanota Mountain range. It has been subject to reforestation activities since 2001 by ECOAN, a non-profit NGO dedicated to the conservation of endangered species and threatened Andean ecosystems. However, at many of its locations, the species grows in habitats that are strongly affected by human activities including grazing and burning. We assess *P.sacra* as Vulnerable (B1a+B2a).

#### Notes.

The taxonomic status of Peruvian populations of *Polylepis* from the Sacred Valley (also known as Vilcanota Valley) related to *P.racemosa* has long been confused. These plants have previously been identified as *P.racemosa* (Simpson 1979; Lloyd 2008a, b; Lloyd and Marsden 2008) or as *P.lanata* (Mendoza and Cano 2012). However, Simpson (1979) already pointed out that collections from Lima (*Killip & Smith 21745*, *Asplund 11374*) with more than one pair of large leaflets and longer inflorescences with numerous flowers are a morphologically extreme type of *P.racemosa* as defined by her. We consider that the recognition of *P.sacra* as a distinct species is justified, based on morphological, ecological and geographical grounds. *Polylepissacra* resembles *P.lanata* in having 2–3 lateral leaflet pairs, lanate hairs and relatively long inflorescences. However, it has narrower leaflets with crenate margins with more teeth per side (0.6–1.1 cm with 6–11 teeth versus 0.9–1.4 cm with 5–9 teeth), shorter inflorescences (5.0–8.8 cm versus (5.0–)6.1–12.3 cm), more stamens per flower (23–27 versus 15–19) and longer styles (3.3–4.1 versus 2.5–3.2 mm).

#### Specimens examined.

**Peru. Apurimac**: Abancay, Bosque de Balcon, 4200 m, 16 June 2004, *Palomino 3924* (QCA!). **Cusco**: Anta, Anta, 13°28'17"S, 072°08'23"W, 3546 m, 01 March 2003, *Arce s.n* (USM!); Pucaccacca, 13°29'21"S, 072°07'59"W, 3635 m, 01 March 2003, *Arce s.n* (USM!); Santa Ana, El Chaccan, 3500 m, 16 November 1972, *Brunel 37* (MO!); Santa Ana, El Chaccan. Sub-xerofito, micro-thermico, 3490 m, 05 March 1973, *Brunel 596* (MO!). Calca, Arin North of Cusco, road Cusco-Urubamba, 13°20'S, 072°01'W, 2897 m, 20 July 1992, *Chávez 807* (CUZ!); Calca, the SW facing slopes found to the immediate E of the prominent tower known by locals as “Kontorqayku” 5 km NE of Huarán, 13°16'05"S, 072°01'08"W, 4245 m, 06 May 2011, *Sylvester 1196* (CUZ!, Z!); Calca, the SW facing slopes found to the immediate E of the prominent tower known by locals as “Kontorqayku” 5 km NE of Huarán, 13°16'06"S, 072°01'08"W, 4250 m, 07 May 2011, *Sylvester 1213* (CUZ!, Z!); Calca, the SW facing slopes found to the immediate E of the prominent tower known by locals as “Kontorqayku” 5 km NE of Huarán, 13°16'11"S, 072°01'06"W, 4230 m, 09 May 2011, *Sylvester 1223* (CUZ!, Z!); Calca, the SW facing slopes found to the immediate E of the prominent tower known by locals as “Kontorqayku” 5 km NE of Huarán, 13°16'13"S, 072°01'05"W, 4243 m, 10 May 2011, *Sylvester 1228* (CUZ!, Z!); Calca, the SW facing slopes found to the immediate E of the prominent tower known by locals as “Kontorqayku” 5 km NE of Huarán, 13°16'10"S, 072°01'07"W, 4237 m, 25 May 2011, *Sylvester 1230* (CUZ!, Z!); Calca, the SW facing slopes found to the immediate E of the prominent tower known by locals as “Kontorqayku” 5 km NE of Huarán, 13°16'05"S, 072°01'16"W, 4387 m, 25 May 2011, *Sylvester 1260* (CUZ!, Z!); Calca, top of the prominent tower known by locals as “Kontorqayku” 5 km NE of Huarán, 13°16'05"S, 072°01'16"W, 4395 m, 16 May 2011, *Sylvester 1262* (CUZ!, Z!); Calca, the SW facing slopes found 700 m E of the prominent tower known by locals as “Kontorqayku” 5 km NE of Huarán, 13°16'16"S, 072°01'05"W, 4242 m, 16 May 2011, *Sylvester 1270* (CUZ!, Z!); Calca, top of the prominent tower known by locals as “Kontorqayku” 5 km NE of Huarán, 13°16'09"S, 072°01'07"W, 4243 m, 16 May 2011, *Sylvester 1278* (CUZ!, Z!); Calca top of the prominent tower known by locals as “Kontorqayku” 5 km NE of Huarán, 13°16'10"S, 072°01'16"W, 4261 m, 16 May 2011, *Sylvester 1281* (CUZ!, Z!); Calca, top of the prominent tower known by locals as “Kontorqayku” 5 km NE of Huarán, 13°16'06"S, 072°01'16"W, 4405 m, 17 May 2011, *Sylvester 1288* (CUZ!, Z!); Calca, the SW facing slopes found to the immediate E of the prominent tower known by locals as “Kontorqayku” 5 km NE of Huarán, 13°16'14"S, 072°01'04"W, 4258 m, 25 May 2011, *Sylvester 1310* (CUZ!, Z!); Calca, top of the prominent tower known by locals as “Kontorqayku” 5 km NE of Huarán, 13°16'06"S, 072°01'17"W, 4403 m, 25 May 2011, *Sylvester 1318* (CUZ!, Z!); Calca, the grassy grazed land found to the immediate E of the prominent tower known by locals as “Kontorqayku” 5 km NE of Huarán, 13°16'09"S, 072°01'06"W, 4247 m, 25 May 2011, *Sylvester 1338* (CUZ!, Z!); Calca, the grassy grazed land found to the immediate E of the prominent tower known by locals as “Kontorqayku” 5 km NE of Huarán, 13°16'05"S, 072°01'03"W, 4297 m, 25 May 2011, *Sylvester 1339* (CUZ!, Z!); Calca, the grassy grazed land found to the immediate E of the prominent tower known by locals as “Kontorqayku” 5 km NE of Huarán, 13°16'05"S, 072°01'03"W, 4297 m, 25 May 2011, *Sylvester 1340* (CUZ!, Z!); Calca, the SW facing slopes found to the immediate E of the prominent tower known by locals as “Kontorqayku” 5 km NE of Huarán, 13°16'03"S, 072°01'02"W, 4310 m, 02 June 2011, *Sylvester 1342* (CUZ!, Z!); Calca, the SW facing slopes found to the immediate E of the prominent tower known by locals as “Kontorqayku” 5 km NE of Huarán, 13°16'04"S, 072°01'04"W, 4295 m, 02 June 2011, *Sylvester 1343* (CUZ!, Z!); Calca, Surayoc, in the deep forested valley 2 km NE of the small settlement of Churo and 6 km NE of Huarán, 13°15'44"S, 072°59'50"W, 4354 m, 17 July 2011, *Sylvester 1351* (CUZ!, Z!); *1352* (CUZ!, Z!); Surayoc, in the deep forested valley 2 km NE of the small settlement of Churo and 6 km NE of Huarán, 13°15'47"S, 071°59'49"W, 4071 m, 25 May 2011, *Sylvester 1403* (CUZ!, Z!); *1406* (CUZ!, Z!); Calca, the SW facing slopes found to the immediate E of the prominent tower known by locals as “Kontorqayku” 5 km NE of Huarán, 13°16'09"S, 072°01'06"W, 4253 m, 25 May 2011, *Sylvester 1684* (CUZ!, Z!); Calca, top of the prominent tower known by locals as “Kontorqayku” 5 km NE of Huarán, 13°16'05"S, 072°01'00"W, 4319 m, 07 June 2012, *Sylvester 1686*; *1687* (CUZ!, Z!); Calca along track from Huaran to Cancha Cancha, 3.5 km north of Huaran, 13°16'38"S, 072°01'44"W, 3377 m, 22 June 2013, *Sylvester 2236* (Z!, LPB). Cusco, Sacsayhuaman, 13°30'35"S, 071°58'43"W, 3391 m, 17 June 2015, *Boza & Urquiaga 3081* (CUZ!, USM!, Z!); San Jerónimo, Hauccoto, Pachatusan, 13°31'02"S, 071°48'39"W, 4400 m, 23 October 2004, *Galiano 6980* (AMAZ, CUZ!, F!, HUT, MO!, MOL, USM!). Espinar, Yauri, localidad Virginniyoc, 4100 m, 13 April 1987, *Núñez 7889B* (CUZ!). Urubamba, Chicón, 13°15'01"S, 072°05'16"W, 3517 m, 01 April 2003, *Arce s.n* (CUZ!, USM!); Yanahuara, Mantanay, 13°12'51"S, 072°09'46"W, 4078 m, 17 June 2015, *Boza & Urquiaga 3034*; *3090*; *3091* (CUZ!, USM!, Z!); above Urubamba, Chicon Valley below Nevado Chaiñapuerto, 13°15'S, 072°05'W, 3370–3550 m, 22 March 1987, *Brandbyge 456* (AAU!); Urubamba, Mantanay, 13°12'03"S, 072°09'20"W, 3350–3800 m, 07 September 2002, *Farfán 281* (CUZ!, F!, HUT, MO!, USM!); Urubamba, Pumahuanca, 13°12'36"S, 072°05'56"W, 3300–3850 m, 09 September 2002, *Farfán 341* (AMAZ, CUZ!, F!, HUT, MO!, MOL, USM!); Huaran ca. Yanachoca, 13°21'S, 072°03'W, 3000–4000 m, 26 January 1991, *Núñez 12568*; *12655* (CUZ!, MO!); Huayoccari to Yanacocha, Urubamba, NW from Cusco, 13°16'S, 072°04'W, 14 February 1987, *Núñez 6999*; *7066* (MO!); desde Huayocri hasta Yanacocha, cerca a Huayllabamba, 13°21'S, 072°03'W, 3000–3890 m, 25 June 1988, *Núñez 9222* (CUZ!, MO!); Pumahuanca, bosque de Queñaquenco-Cuyo, 4100 m, 16 June 2004, *Palomino 4809* (QCA!); Urubamba, 1.5 km 100 NE up the valley from the Munaycha college, 5 km North upvalley from Urubamba, 13°14'39"S, 072°04'46"W, 3865 m, 25 May 2011, *Sylvester 1349*; *1350* (CUZ!, Z!); Huayllabamba, grazed slope situated on the N side of Laguna Qellococha 500 m further N passing through the *Polylepis* forest 5 km N of Huayocari Village, 13°16'42"S, 072°03'00"W, 4168 m, 28 February 2011, *Sylvester 604*; *637*; *643*; *644* (CUZ!, Z!); Huayllabamba, grazed slope situated on the N side of Laguna Qellococha 500 m further N passing through the *Polylepis* forest 5 km N of Huayocari Village, 13°16'43"S, 072°02'56"W, 4182 m, 11 March 2011, *Sylvester 700*; *725* (CUZ!, Z!); Huayllabamba, grazed slope situated on the N side of Laguna Qellococha 500 m further N passing through tha *Polylepis* forest 5 km N of Huayocari Village, 13°16'43"S, 072°03'00"W, 4162 m, 12 March 2011, *Sylvester 732* (CUZ!, Z!); Huayllabamba, grazed slope situated on the N side of Laguna Qellococha 500 m further N passing through tha *Polylepis* forest 5 km N of Huayocari Village, 13°16'36"S, 072°03'03"W, 4199 m, 13 March 2011, *Sylvester 755* (CUZ!, Z!); Huayllabamba, grazed slope situated on the N side of Laguna Qellococha 500 m further N passing through tha *Polylepis* forest 5 km N of Huayocari Village, 13°16'42"S, 072°03'07"W, 4163 m, 13 March 2011, *Sylvester 764* (CUZ!, Z!); Huayllabamba, grazed slope situated on the N side of Laguna Qellococha 500 m further N passing through tha *Polylepis* forest 5 km N of Huayocari Village, 13°16'42"S, 072°03'07"W, 4229 m, 14 March 2011, *Sylvester 765*; *773* (CUZ!, Z!); Huayllabamba, grazed slope situated on the N side of Laguna Qellococha 500 m further N passing through tha *Polylepis* forest 5 km N of Huayocari Village, 13°16'42"S, 072°03'07"W, 4215 m, 14 March 2011, *Sylvester 781* (Z!); Huayllabamba, grazed land situated on the S side of Laguna Qellococha 5 km N of Huayocari Village 55 m S of Laguna Qellococha, 13°16'42"S, 072°03'01"W, 4151 m, 15 March 2011, *Sylvester 789* (Z!); *796* (Z!); Huayllabamba, Yanacocha, 13°17'12"S, 072°03'06"W, 3760 m, 07 June 2006, *Toivonen 43*; *44*; *45* (CUZ!); Huayllabamba, Yanacocha, 13°16'49"S, 072°59'06"W, 3960 m, 07 June 2006, *Toivonen 47*; *48* (CUZ!); Mantanay, 13°12'07"S, 072°09'32"W, 4100 m, 19 July 2007, *Toivonen 5; 6; 7; 8* (CUZ!); Huayllabamba, Yanacocha, 13°16'55"S, 072°03'00"W, 3950 m, 16 June 2006, *Toivonen 50*; *51* (CUZ!); Huayllabamba, Qelloqocha, 13°16'52"S, 072°03'07"W, 4180 m, 31 August 2006, *Toivonen 53*; *54*; *55*; *56* (CUZ!); Urubamba, Pumahuanca, 13°12'30"S, 072°05'53"W, 4150 m, 13 June 2006, *Toivonen 59* (CUZ!); Urubamba, Pumahuanca, 13°12'43"S, 072°05'51"W, 4140 m, 25 May 2006, *Toivonen 61* (CUZ!). Huayllabamba, entre quebrada de Huayaccari y las lagunas de Yanacocha y Kellococha parte alta y media, 13°21'15"S, 072°03'55"W, 3400–4200 m, 17–18 May 1989, *Tupayachi 1044* (MO!); entre la quebrada de Huayoccari y las lagunas de Yanacocha y Kellococha, hacia las laderas Sureste de las lagunas, 13°19'00"S, 072°02'00"W, 2900–3600 m, 19–23 June 1989, *Tupayachi 1092* (GH!, MO!); Huayllabamba, entre Huayoccari y las lagunas de Yanacocha y Kellococha, 13°13'S, 072°16'W, 2900–4600 m, 17–18 July 1989, *Tupayachi 1130* (MO!); *1138* (MO!); Huayllabamba, Lagunas Yanachocha y Quellococha hacia San Juan, NE de Cusco, 13°16'S, 072°04'W, 2900–4600 m, 19 August 1989, *Tupayachi 1212A* (MO!); *1213* (MO!); Huayllabamba, Huayaccari-Yanaccocha; camino a Yanaccocha, 13°21'S, 072°03'W, 3000–4200 m, 03 March 1986, *Tupayachi 137* (CUZ!, MO!); Huayllabamba. Laguna Yanaccocha y Kello ccocha, 13°21'15"S, 072°03'55"W, 3800–4200 m, 07 January 1989, *Tupayachi 854* (MO!); Huayllabamba, entre la quebrada Huayoccari, Lagunas de Yanacocha y Kellococha, 13°21'15"S, 072°03'55"W, 2900–3860 m, 12 February 1989, *Tupayachi 896* (MO!); Urubamba, Pumahuanca, 13°14'35"S, 072°06'50"W, 3543 m, 20 February 2006, *Valenzuela 6154* (AMAZ, CUZ!, HUT, MO!, USM!); Pumahuanca, 3450–3850 m, 25 October 1952, *Vargas 10799* (CUZ!); Quebrada Chicon, 3000 m, 14 August 1974, *Vargas 22604* (CUZ!); Sutocc to Pacchacc, 3500–3650 m, 15 November 1962, *Vargas 7855* (CUZ!); Yanahuara, 3600 m, 16 March 1950, *Vargas 9315* (CUZ!). **Junín**: Jauja, Llocllapampa, 3350 m, *Rosales 4* (USM!). Lago Chinchay Cocha Junin, Ondores, 11°04'S, 076°08'W, 4000 m, 05 February 1987, *Boertmann 17a* (AAU!). Llocllapampa, Parco entre Jauja y Oroya, 3200–3300 m, 24 November 1947, *Ferreyra 2833* (MO!, USM!). Tarma, Ayabamba, 27 April 1905, *Soukup 2536* (F!). **Lima**: Huarochiri, Vista Alegre, Río Blanco, 3923 m, 21 February 1990, *Arce 205* (MO!); 3400 m, 03 June 1940, *Asplund 11343* (US!); 3700 m, 04 June 1940, *Asplund 11374* (US!). San Mateo, Río Blanco; open hillside, 3000–3500 m, 15–17 April 1929, *Killip 21745* (A!, F!, US!).

### ﻿34. Polylepis triacontandra

Taxon classificationPlantaeRosalesRosaceae

Bitter, Bot. Jahrb. Syst. 45: 630. 1911.

B1CFF346-37F1-5C5B-8BF9-7B1C696B23A7

Figs 90 , 91

Polylepis racemosa Ruiz & Pav. subsp. triacontandra (Bitter) M. Kessler, Candollea 50: 144. 1995. Type: based on Polylepistriacontandra Bitter.
Polylepis subquinquefolia Bitter, Bot. Jahrb. Syst. 45: 636. 1911. Type. Peru. Puno: Sandia above Cuyoenyo, 3600 m, *Weberbaueri 931* (holotype: B destroyed, photos at F!, GH!, MO!, NY!, US!).

#### Type.

Bolivia. La Paz: Larecaja near Sorata, Cochipata, 3300 m, 9 Oct 1818, *Mandon 674* (lectotype designated by Simpson 1979, pg. 42: G!; isolectotypes: BM!, F! GH!, GOET!, K!, NY!, P!, S!, US!).

**Figure 90.**
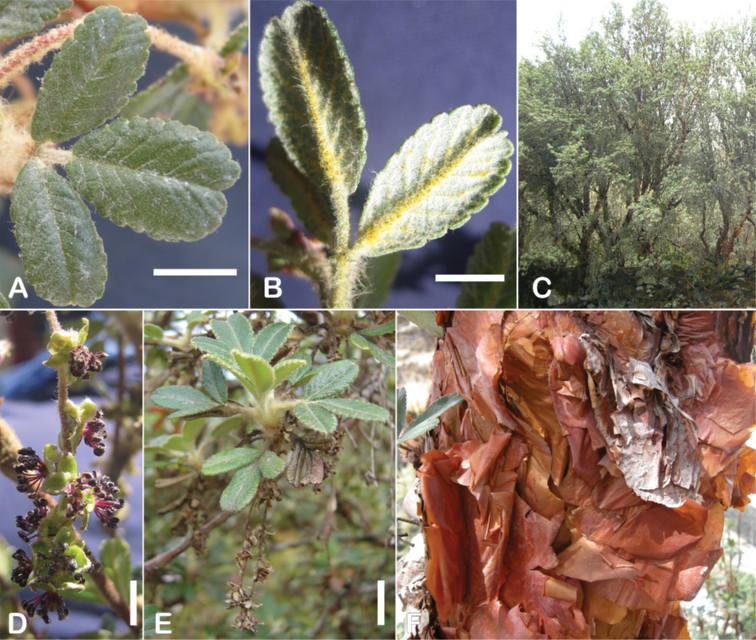
*Polylepistriacontandra* Bitter **A** Upper leaflet surface **B** lower leaflet surface **C** habit **D** flowers **E** flowering branch **F** bark. Scale bars: 1 cm (**A, B**); 5 mm (**D**); 2 cm (**E**). Photographs **A, B, D–F** A. Fuentes **C** A. Domic.

#### Diagnosis.

***Trees*** 1–10 m tall. ***Leaves*** slightly congested at the branch tips, imparipinnate with 1(–2) pair of leaflets, obtrullate in outline, (3.7–)4.4–4.9 × 3.2–4.1 cm; rachises densely lanate, points of leaflet attachment with a tuft of long hairs; stipular sheaths apically truncate, densely lanate on the outer surfaces; leaflets narrowly elliptic in outline, second pair from the terminal leaflet the largest, one of this pair (2.0–)2.6–3.3 × 0.7–1.1 cm; margin crenate with 8–12 teeth, apically acute to obtuse or slightly emarginate, basally cuneate; upper leaflet surfaces glabrous; lower leaflet surfaces densely tomentose with whitish hairs 0.4–0.8 mm long mixed with pannose hairs, second pair of leaflet, if present, very small. ***Inflorescences*** pendant, (4.9–)5.5–7.0(–9.5) cm long, bearing 11–13 flowers; floral bracts 5.9–7.6 mm long, narrowly triangular, densely villous on the outer surface; rachises villous. ***Flowers*** 5.8–8.2(–10.1) mm diam.; sepals 4, ovate, green, densely villous outside; stamens 21–23, anthers orbicular, with a dense tuft of straight white hairs on the upper half; styles fimbriate, 3.3–3.8 mm long. ***Fruits*** turbinate, with 2–4 irregular flattened ridges with a series of spines, densely villous; 4.9–5.7 × 3.4–5.7 mm including spines. ***Tetraploid***.

**Figure 91.**
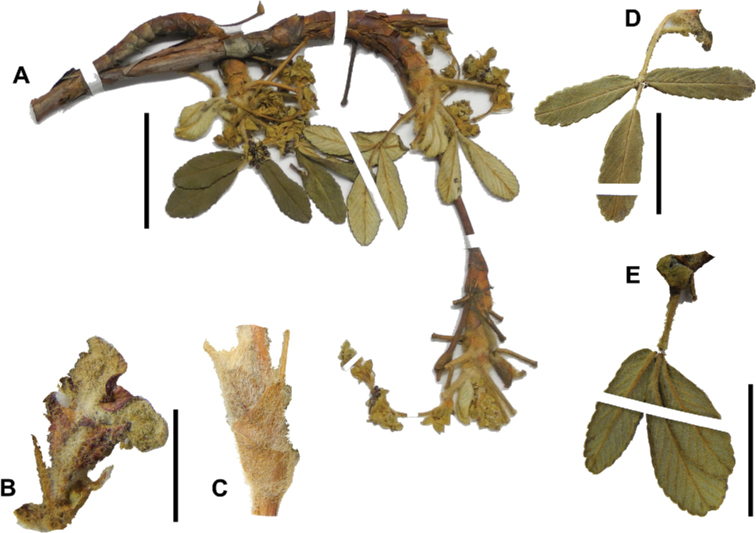
*Polylepistricontandra* Bitter **A** flowering branch **B** fruit **C** stipular sheaths **D** upper leaf surface **E** lower leaf surface (**A***Kessler 3572***B***Kessler 3567***C***Kessler 3571***D***Kessler 3582***E***Kessler 3573*). Scale bars: 7 cm (**A**); 5 mm (**B**); 3 cm (**D, E**). Photographs by T. E. Boza E.

#### Distribution, habitat and ecology.

**Figure 92.**
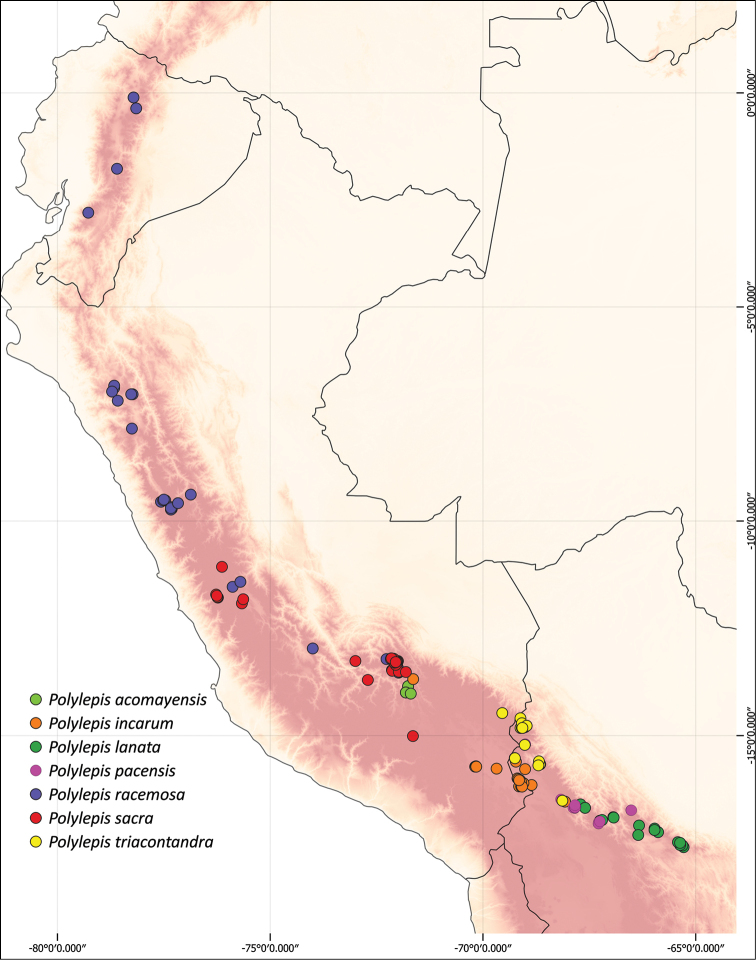
Geographical distribution of the species of subsection Racemosae.

*Polylepistriacontandra* has been found in southern Puno (Peru), where it has been collected at just one locality in Sandia Province, across the Cordillera Apolobamba to the northern Cordillera Real in La Paz (Bolivia) (Fig. 92). It grows on the upper limit of the humid Yungas forests at 2360–4310 m elevation. It has been often planted around communities near Titicaca Lake and in the City of La Paz (Kessler 1995b). Many populations are remnants growing in areas with abrupt topography, such as inaccessible headwaters, micro-basins and rocky slopes (Navarro et al. 2010).

#### Conservation status.

The EOO for *Polylepistriacontandra* is estimated as 27,030 km^2^, the AOO is assessed at 60 km^2^ and it is known from 12 locations. *Polylepistriacontandra* was categorized as VU (B1+2c, D2) in the World List of Threatened Trees (Oldfield et al. 1998). Later, it was classified as VU (B1b(i,ii,iii)) in the Red List of Threatened Flora of Bolivia (Arrázola et al. 2012). It is protected within the Apolobamba Integrated Management Natural Area. Although the species is under some protection, its populations are also heavily fragmented. We assess *P.triacontandra* as Endangered (A1, B1a+B2a, C1).

#### Notes.

*Polylepistriacontandra* resembles *P.subtusalbida* in having 1(–2) lateral leaflet pairs and tomentose hairs. However, it has narrowly elliptic leaflets with crenate margin, inflorescences 4.9–9.5 cm long with 11–13 flowers and styles 3.3–3.8 mm long, whereas *P.subtusalbida* has obovate leaflets with serrate margin, inflorescences 1.8–3.7 cm long with 3–4 flowers and styles 2.8–3.4 mm long. *Polylepistriacontandra* is also morphologically similar to *P.pallidistigma* with which shares the number of lateral leaflets and margin type. It differs by its leaflets (2.0–)2.6–3.3 × 0.7–1.1 cm long with tomentose hairs on the lower leaflet surfaces, and inflorescences 4.9–9.5 cm long with 11–13 flowers, compared to the smaller leaflets (1.2–.2.0 × 0.5–0.8 cm) with pannose hairs on the lower leaflet surfaces and shorter inflorescences (2.7–6.0 cm) with 5–6 flowers of *P.pallidistigma*. For additional morphological similarities, see under *P.lanata*.

#### Specimens examined.

**Bolivia. La Paz**: Bautista Saavedra, Chajaya, a few kilometers from Charazani, 15°13'S, 069°01'W, 3500 m, 30 March 1985, *Solomon 13346* (MO!). Camacho, Puerto Acosta, 3860 m, 05 April 1982, *Beck 7662* (GOET!). Franz Tamayo, Madidi, Apolobamba, Agua Blanca, Area Natural de Manejo Integrado Apolobamba, Agua Blanca y carretera hacia Antaquilla, 14°49'11"S, 069°07'00"W, 4031 m, 07 June 2008, *Fuentes 12780* (BOLV, LPB, MO!, QCA!, USZ); Area Natural de Manejo Integrado Apolobamba, Keara Bajo, 14°42'43"S, 069°05'03"W, 3500 m, 18 June 2005, *Fuentes 8390* (LPB, MO!, QCA!); Area Natural de Manejo Integrado Apolobamba, Pelechuco camino hacia Agua Blanca, 14°49'19"S, 069°07'01"W, 3593 m, 07 June 2008, *Huaylla 2566* (BOLV, LPB, MO!, QCA!, USZ); 2 km S Pelechuco on road to Ulla Ulla, 3700 m, 28 August 1991, *Kessler 3051*; *3052* (AAU!, GOET!); *3402* (AAU!); *3420* (GOET!, LPB, MO!); *3421* (GOET!); *3422* (AAU!, GOET!, LPB, MO!); *3423* (GOET!); *3424* (AAU!, GOET!); *3425* (GOET!, LPB, MO!); *3558*; *3559*; *3560*; *3561*; *3562*; *3563*; *3564*; *3565*; *3566*; *3567*; *3568*; *3569*; *3570*; *3571*; *3572*; *3573*; *3574*; *3575*; *3576*; *3578*; *3579*; *3580*; *3581*; *3582*; *3583*; *3584*; *3585*; *3586*; *3587* (GOET!); Apolobamba, Puina, 14°35'43"S, 069°07'05"W, 3747 m, 24 April 2008, *Quisbert 929* (LPB, MA, MO!, QCA!); Area Natural de Manejo Integrado Apolobamba, Pelechuco, 3647 m, 24 May 2009, *Reguerin 116* (QCA!); *116* (LPB, MO!, QCA!, USZ); Área Natural de Manejo Integrado Apolobamba, Pelechuco, 14°49'15"S, 069°04'07"W, 3614 m, 24 May 2009, *Reguerín 118* (BOLV, LPB, MO!, QCA!, USZ); *119* (LPB, MA, MO!, USZ); Área Natural de Manejo Integrado Apolobamba, Pelechuco, 14°49'17"S, 069°04'10"W, 3649 m, 25 May 2009, *Reguerín 121* (LPB, MA, MO!, USZ); Madidi, Apolobamba, Pelechuco-río abajo, Santa Ana, 14°46'14"S, 068°58'21"W, 2184 m, 16 May 2009, *Torrez 550* (LPB, MA, MO!, QCA!); *550* (QCA!); *553* (LPB, MO!, QCA!); *553* (QCA!); Madidi, Apolobamba, Pelechuco-río abajo, Santa Ana, 17°49'15"S, 069°04'01"W, 3547 m, 25 May 2009, *Torrez 586* (LPB, MA, MO!, NY, QCA!); 58.5 km N of Sorata on road to Quiabaya, 15°36'S, 068°41'W, 3600 m, 16 December 1981, *Solomon 6640* (MO!); Sorata, 15°40'03"S, 068°39'18"W, 3660 m, 31 July 2010, *Steudel 426*; *427*; *428*; *431*; *433* (Z!). Murillo, Calacoto (La Paz), ornamental plants, 16°31'S, 068°08'W, 3300 m, 15 August 1986, *Solomon 15492* (MO!).

### ﻿Subsection Besseria

Taxon classificationPlantaeRosalesRosaceae

T.Boza & M.Kessler sect. nov.

44E3675F-7BC4-5899-B3EF-630A93E6F74C

urn:lsid:ipni.org:names:77301649-1

#### Diagnosis.

Trees; 1–2(–3) lateral leaflet pairs; lower leaflet surfaces with a dense layer of very short, white pannose hairs sometimes mixed with long tomentose hairs; fruits with 2–5 irregular flattened ridges with a series of spines, sparsely to densely tomentose or villous.

#### Type.

*Polylepisbesseri* Hieron.

#### Note.

The subsectional epithet *Besseria* is a noun in apposition.

### ﻿35. Polylepis besseri

Taxon classificationPlantaeRosalesRosaceae

Hieron., Bot. Jahrb. Syst. 21(3): 312. 1896.

F6D770FA-D5D2-51C4-84D8-FB51C212F0D6

Figs 93 , 94

Polylepis besseri var. longipedicellata Bitter, Bot. Jahrb. Syst. 45: 629. 1911. Type. Bolivia. Cochabamba: Puna of Mizque, *d’Orbigny 495* (holotype: G!; isotypes: F! NY, US, photos at US!, GH!).

#### Type.

Bolivia. Capi: Mar 1890, *Bang 769* (lectotype: G!; isolectotypes: BR!, E!, F!, GH, MO!, NY (2)!, US!, W).

**Figure 93.**
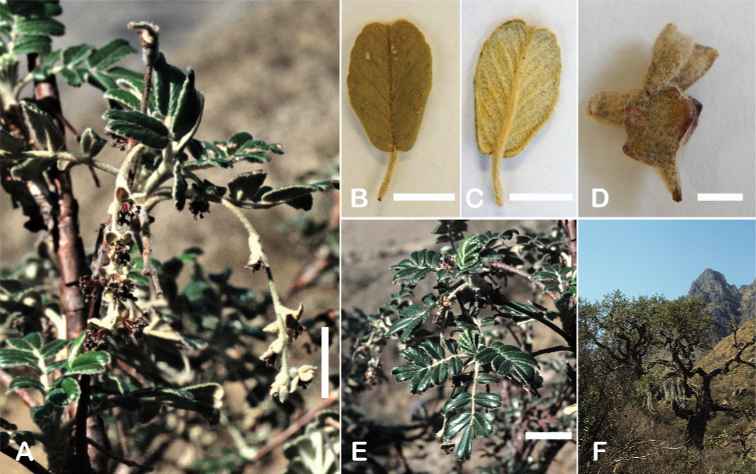
*Polylepisbesseri* Hieron **A** flowering branch **B** upper leaflet surface **C** lower leaflet surface **D** fruit **E** leaves **F** habit. Scale bars: 2 cm (**A, E**); 5 mm (**B, C**); 2 mm (**D**). Photographs **A, E, F** M. Kessler **B–D** T.E. Boza E.

#### Description.

***Trees*** 2–6 m tall. ***Leaves*** slightly congested at the branch tips, imparipinnate with 1–2(–3) pairs of leaflets, obtrullate in outline, 3.6–4.0 × 2.5–3.0 cm; rachises densely tomentose, points of leaflet attachment with a tuft of long hairs; stipular sheaths apically truncate or slightly spurred, densely tomentose on the outer surfaces; leaflets obovate in outline, second pair from the terminal leaflet the largest, one of this pair 1.4–1.6 × 0.6–1.1 cm; margin crenate with 5–8 teeth, apically obtuse or emarginate, basally unequally attenuate or cordate; upper leaflet surfaces smooth to slightly rugose, glabrous; lower leaflet surfaces densely tomentose hairs 0.6–0.8 mm long, with a dense layer of very short, white pannose hairs. ***Inflorescences*** pendant, (3.6–)4.9–8.9 cm long, bearing 7–9 flowers; floral bracts 4.7–4.9 mm long, narrowly triangular, sparsely tomentose on the outer surface; rachises sparsely to densely tomentose. ***Flowers*** 8.3–8.6 mm diam.; sepals 4, ovate, green, densely tomentose outside; stamens 13–23, anthers orbicular, with a dense tuft of straight white hairs on the upper half; styles fimbriate, 2.5–3.1 mm long. ***Fruits*** turbinate, with 2–5 flattened ridges with a series of spines, densely tomentose; 6.1–9.7 × 4.0–5.8 mm including spines. ***Tetraploid***.

**Figure 94.**
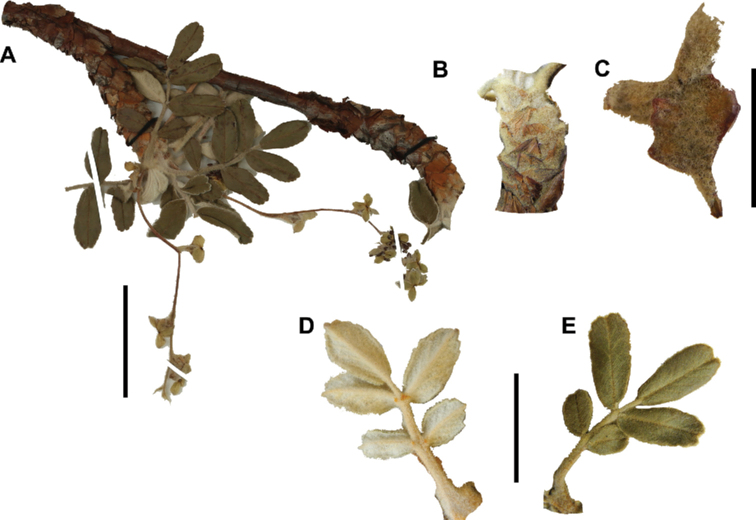
*Polylepisbesseri* Hieron **A** flowering branch **B** stipular sheaths **C** fruit **D** lower leaf surface **E** upper leaf surface (**A***Kessler 3210***B***ERTS 60***C–E***Kessler 2988*). Scale bars: 5 cm (**A**); 9 mm (**C**); 2 cm (**D, E**). Photographs by T. E. Boza E.

#### Distribution, habitat and ecology.

*Polylepisbesseri* is restricted to the Departments of Cochabamba. Chuquisaca and Potosí in Bolivia (Fig. 103). It occurs in relatively dry regions at 2200–4250 m elevation. Frequently, it forms pure stands, but it can also locally co-occur with *P.subtusalbida*, *P.tomentella* and *P.lanata* (Kessler 1995b). It usually grows with *Escalloniamyrtilloides* in northern Chuquisaca (Navarro et al. 2010). At Sacha Loma (Cochabamba), low annual growth rates (< 1 mm/y) have been reported (Gareca et al. 2010). There as well, it was found that > 90% of viable seeds germinate under appropriate laboratory conditions (Gareca et al. 2012). In the same region, there is a loss of genetic diversity and increasing differentiation in offspring related to adults, which suggest that *P.besseri* is experiencing genetic erosion and genetic drift (Gareca et al. 2013). Forests of *P.besseri* support specialist bird species, such as *Conirostrumbinghami* (= *Oreomanesfraseri*), *Leptasthenurayanacensis* and *Cardueliscrassirostris*, as well as other species that show marked affinity for this habitat, such as *Asthenesdorbignyi*, *Phacellodomusstraticeps*, *Polioxolmisrufipennis*, *Ochthoecaoenanthoides* and *Pseudosaltator* (= *Saltator*) *rufiventris* (Herzog et al. 2002; Herzog et al. 2003). Larger forests with larger trees have higher arthropod density, allowing for higher bird densities (Cahill et al. 2021). *Cardueliscrassirostris* is one of the few bird species that specifically feed on the seeds of *Polylepis* trees, but the effects of this remain unexplored.

#### Conservation status.

The EOO for *Polylepisbesseri* is estimated as 58,954 km^2^, the AOO is assessed at 76 km^2^ and it is known from 18 locations. *Polylepisbesseri* was categorized as VU (A1abc, B1+2c) in the World List of Tthreatened Trees (Oldfield et al. 1998). Later, it was classified as EN B2ab(ii,iii) in the Red List of Threatened Flora of Bolivia (Arrázola and Coronado 2012). It is not protected within any conservation area. *Polylepisbesseri* forests are under increasing threat from human activities and disturbance. We assess the species as Vulnerable (A1, B1a+B2a, C1).

#### Notes.

*Polylepisbesseri* is morphologically closest to *P.pacensis* with which it shares similar obovate leaflet shape with crenate margins. It differs from this in its longer (0.6–0.8 mm), densely tomentose hairs mixed with a dense layer of very short, white pannose hairs compared to the shorter (0.4–0.9 mm), densely villous hairs without pannose hairs of *P.pacensis*. Additionally, *P.besseri* can be distinguished from *P.rugulosa* by the number of lateral leaflet pairs (1–2(–3) versus 1) and lower leaflet hairs length (0.6–0.8 mm versus 0.8–1.0 mm) which, in *P.besseri*, is mixed with a very dense layer of very short pannose hairs that is absent in *P.rugulosa*. In Potosí, hybrids have been found with *P.neglecta* (Schmidt-Lebuhn et al. 2006a).

#### Specimens examined.

**Bolivia. Chuquisaca**: Belisario Boeto, Municipio Villa Serrano, Comunidad Nuevo Mundo, Piso Superior Tucumáno Boliviano, 18°59'20"S, 064°18'17"W, 2369 m, 17 August 2005, *Villalobos 56* (MO!). Oropeza, ca. 40 km W Sucre on road to Macha, 18°53'S, 065°26'W, 3300 m, 26 September 1991, *Kessler 3263* (GOET!, LPB); *3264* (AAU!, GOET!); *3265* (GOET!); *3266* (AAU!, GOET!); *3267* (AAU!, GOET!, LPB); Cerca de Río Ravelo, 3800 m, 15 June 1918, *Murguía 145* (GOET!, LPB); Localidad Chataquilla, 3400 m, 06 December 1989, *Murguía 377* (GOET!). Zudañez, 25 km S Icla on Tarabuco-Azurduy road, 19°27'S, 064°49'W, 3500 m, 24 September 1991, *Kessler 3208* (GOET!); *3209* (AAU!); *3210*; *3212* (AAU!, GOET!); 40 Km S Icla on Tarabuco Azurduy road, 19°33'S, 064°39'W, 3700 m, 24 September 1991, *Kessler 3223* (AAU!, GOET!). **Cochabamba**: Arque, proximidades a la comunidad de Kutimarca, Sumuruni, camino hacia Arque, 3850 m, 11 April 1999, *Mercado 2149* (MO!). Campero, Pallamiani Khasa, camino Aiquile-Rakaypampa, hacia Pallamiani Khasa-Lenkho, 3200 m, 09 May 1987, *Estenssoro 692* (GOET!, LPB). Carrasco, Entrando uno 23 km de la carretera (cerca Epizana) a la ciudadela incaica Inkallajta, 17°36'15"S, 065°24'57"W, 3400 m, 09 May 2014, *Beck 34452* (LPB); 6.6 km by road, NW Lopez Mendoza, at Km 98 from Cochabamba, 3250 m. Quebrado Majón, 17°33'20"S, 065°21'31"W, 3250 m, 15 May 1984, *Schmitt 108* (MO!); 5 km al este del puente sobre el Río López Mendoza por el camino entre Cochabamba y Santa Cruz (19 km al oeste de Epizana), 17°32'S, 065°22'W, 2900 m, 11 February 1987, *Solomon 16038* (LPB, MO!, NY). Cercado, Estancia Sipirita entre Apote y Tiquipaya, 2650 m, 18 January 1996, *De la Barra 656* (BOLV); Parque Nacional Tunari, N and above the center of Cochabamba, at about km. 11.5 above the entrance, about 1 km by switchback road above and NE of the recreation area at “km 10.”, 17°19'45"S, 066°08'15"W, 3500 m, 02 May 2005, *Nee 52934* (MO!, NY, USZ!). Chapare, Parque Tunari; the road to Laguna Wara Wara, 3600 m, 28 May 1994, *Ritter 1060* (GH!, MO!). Mizque, Cañada Pucara Mayu, a 37 km de Rodeo Mizque, entre Khewiña Khasa y Pucara Khasa, 17°49'S, 065°26'W, 3200 m, 08 May 1987, *Estenssoro 810* (GOET!, LPB); Semborreto entre Markilla y Rodeo, 3630 m, 20 June 1987, *Estenssoro 819* (LPB); Between Cerro Canto Monte and Mizque along Arani-Mizque road, 3600 m, 17 April 1987, *Fjeldså s.n* (LPB); ca. 30 Km NW Mizque on road to Arani, 2950 m, 17 August 1991, *Kessler 2985* (AAU!, GOET!, LPB); *2988* (AAU!, GOET!); *2989* (AAU!, GOET!); *2996* (GOET!, MO!). Tiraque, 12.8 km W of Koari, highway from Cochabamba to Epizana, 17°27'44"S, 065°41'01"W, 3475 m, 04 May 2007, *Nee 55332* (MO!); San Isidro, A Km 88 carretera Cochabamba-Santa Cruz, entrando hacia al norte, 5 km de la comunidad de San Isidro, 17°26'54"S, 065°31'38"W, 4300 m, 18 March 2006, *Zárate 2306* (BOLV, MO!); 106.8 km E of Cochabamba on Carretera Fundamental, 4100 m, 05 December 1979, *Davidson 3743* (F!, NY); Chapare Yungas de Espiritu, following the abandoned Chapare Road along the NW facing side of the Serrania de Callejas at the head waters of the río Espíritu Santo, ca. 50 km 25 N of W from Cochabamba, 17°12'S, 065°42'W, 4000 m, 01 December 1985, *Lewis s.n* (F!, LPB, MO!). **La Paz**: Murillo, Río Minasa, 1.5 km arriba del viejo puente del ferrocarril (ca. 3 km arriba de Villa Fatima, La Paz), 16°27'S, 068°07'W, 4000 m, 18 January 1987, *Solomon 15783* (LPB, MO!). **Potosí**: Charcas, de Ocuri hacia Pajri cuchu (bajando), 4000 m, 12 March 1993, *Torrico 119* (LPB). Jose M. Linares, a 2 km de Lajas hacia Tambillo, Serranía de Mataca, 3930 m, 05 April 1993, *Torrico 346* (LPB). Capi, March 1890, *Bang 769* (F!, G, GH!, MO!, NY, US!, W); Alturas de Chacatilla, 3500 m, 23 August 1980, *Erquicia 60* (GOET!); 23 January 1905, *Orbigny s.n* (MO!).

### ﻿36. Polylepis crista-galli

Taxon classificationPlantaeRosalesRosaceae

Bitter, Bot. Jahrb. Syst. 45: 633. 1911.

90DFE649-2754-57B5-A8F6-055E4CA5DF7E

Figs 95 , 96

Polylepis crista-gallii var. longiracemosa Bitter, Bot. Jahrb. Syst. 45: 634. 1911. Type. Bolivia. Tarija: Pinos, between Tarija and San Luis, 2500–2700 m, 1 Mar 1903, *Fries 1296* (holotype: S!).

#### Type.

Bolivia. Tarija: Tucumilla, 2500 m, *Fiebrig 2020* (lectotype, designated by Simpson 1979, pg. 42: G!; lectoisotypes: A!, E!, K!, M!, P).

**Figure 95.**
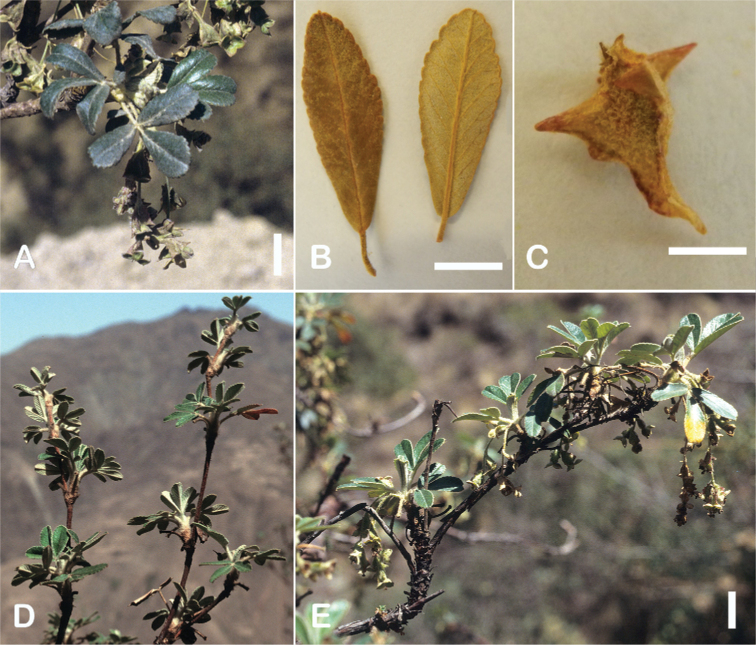
*Polylepiscrista-galli* Bitter **A** flowering branch **B** upper and lower leaflet surfaces **C** fruit **D** branching pattern **E** fruiting branch. Scale bars: 1 cm (**A, E**); 5 mm (**B**); 2 mm (**C**). Photographs **A, D, E** M. Kessler **B, C** T.E. Boza E.

#### Description.

***Trees*** 3–6 m tall. *Leaves* slightly congested at the branch tips, imparipinnate with (1–)2 pairs of leaflets, obtrullate in outline, 2.1–4.4(–6.2) × 1.8–2.8(5.8) cm; rachises densely tomentose, points of leaflet attachment with a tuft of long hairs; stipular sheaths apically truncate or slightly spurred, densely tomentose on the outer surfaces; leaflets obovate in outline, second pair from the terminal leaflet the largest, one of this pair 1.3–2.7(–3.2) × (0.4–)0.8–1.1(–1.4) cm; margin serrate with 9–13 teeth, apically obtuse or emarginate, basally unequally attenuate; upper leaflet surfaces slightly rugose, glabrous; lower leaflet surfaces with a dense layer of very short, white pannose hairs. ***Inflorescences*** pendant, (2.2–)3.4–5.0(–8.0) cm long, bearing 5–7 flowers; floral bracts 3.4–4.0 mm long, narrowly triangular, densely tomentose on the outer surface; rachises tomentose. ***Flowers*** 6.0–9.2 mm diam.; sepals 4, ovate, green, sparsely to densely tomentose outside; stamens 13–15, anthers orbicular, with a dense tuft of straight white hairs on the upper half; styles fimbriate, 1.6–2.3 mm long. ***Fruits*** turbinate, with 2–4 irregular flattened ridges with a series of spines, sparsely tomentose; 4.9–6.1 × 4.0–6.2 mm including spines. ***Tetraploid***.

**Figure 96.**
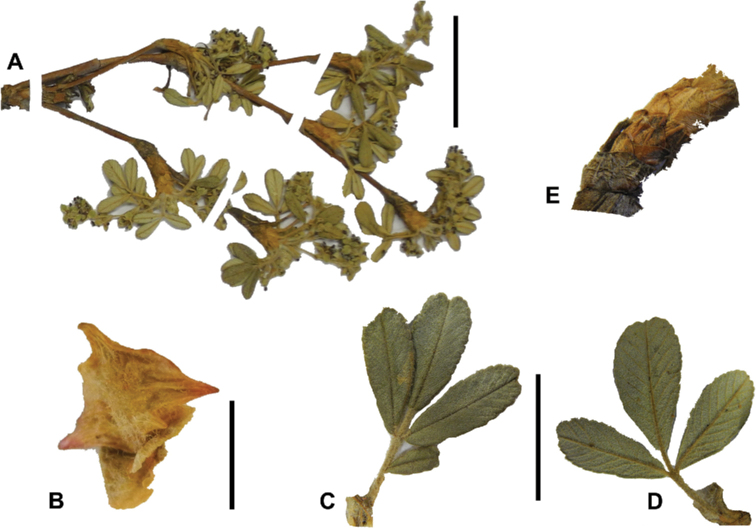
*Polylepiscrista-galli* Bitter **A** flowering branch **B** fruit **C** upper leaf surface **D** lower leaf surface **E** stipular sheaths (**A***Kessler 3174***B, C***Kessler 3155***D***Kessler 3661***E***Kessler 3172*). Scale bars: 9 cm (**A**); 4 mm (**B**); 4 cm (**C, D**). Photographs by T. E. Boza E.

#### Distribution, habitat and ecology.

*Polylepiscrista-galli* is distributed from central Bolivia to Jujuy (Argentina) (Fig. 103). It occurs in Boliviano-Tucumanic forests at 2200–4050 m elevation. It mostly grows as homogeneous forest and sometimes mixed with *Alnusacuminata* (Kessler 1995b) or *Escalloniahypoglauca* (Navarro et al. 2010). In Alto Calilegua (Jujuy), a stand of *P.crista-galli* has canopy cover of 35% and tree heights of 2–5 m height (Renison et al. 2013).

#### Conservation status.

The EOO for *Polylepiscrista-galli* is estimated as 46,634 km^2^, the AOO is assessed at 60 km^2^, and it is known from 14 locations. *Polylepiscrista-galli* was categorized as VU (A1acd, B1+2c) in the World List of Threatened Trees (Oldfield et al. 1998) and as VU (B2ab(ii,iii)) in the Red List of Threatened Flora of Bolivia (Arrázola and Coronado 2012). Later, it was categorized as EN B2ab(iii) in the Red List of Montane Tree species of the tropical Andes (Tejedor et al. 2014). No conservation actions have been taken to date. The area of occupation is in constant reduction, with small isolated and disconnect patches, and continuous population declines. We assess *P.crista-galli* as Vunerable (A1+ A2a, B1a+B2a, C2a).

#### Notes.

*Polylepiscrista-galli* is similar to *P.subtusalbida*, but differs by having leaflets 1.3–2.7(3.2) cm long (versus 0.9–1.6 cm in *P.subtusalbida*), lower leaflet surface with a dense layer of very short pannose hairs (versus glabrous to sparsely longer tomentose hairs 0.5–1.2 mm), 5–7 flowers per inflorescence (versus 3–4), 13–15 stamens per flower (versus 19–21) and styles 1.6–2.3 mm long (versus 2.8–3.4 mm). Perhaps most importantly, the fruits of *P.crista-galli* have larger, often red-tinted ridges, to which the species epithet refers.

*Polylepiscrista-galli* has been suggested to have a hybridogenic origin, with a member of section Australes as one parent and one of subsection Besseria as the other (Schmidt-Lebuhn et al. 2006a). This is supported by the fact that *P.crista-galli* is morphologically intermediate between the putative parents and is, in fact, indistinguishable from hybrids between *P.besseri* and *P.neglecta* found in Bolivia. Interestingly, the range of *P.crista-galli* perfectly fills the gap between the ranges of *P.australis* (to the south) and *P.neglecta* (north), suggesting that the formation of *P.crista-galli* might have obliterated part of a formerly continuous range of the progenitor of *P.australis* and *P.neglecta*.

#### Specimens examined.

**Bolivia. Chuquisaca**: Azurduy, Trayecto Azurduy-Río Pilcomayo, 20°12'52"S, 064°26'37"W, 3114 m, 14 October 2007, *Portal 146* (HSB, MO!). Belisario Boeto, 1 km S Mendoza, 2830 m, 29 April 1987, *Murguía 46* (GOET!, LPB). Nor Cinti, ca. 40 km E Culpina on road to Chillajara, 20°37'S, 064°46'W, 3000 m, 21 September 1991, *Kessler 3169* (AAU!, GOET!, LPB); *3170* (GOET!, MO!); *3171* (AAU!, GOET!, LPB); *3172* (GOET!, LPB); *3174* (AAU!, GOET!); 19.1 km S of Padcaya on the road to Camargo *Solomon 10646* (LPB, MO!). Sud Cinti, Cerro Cobre Khasa, between Culpina and El Palmar, 20°48'S, 064°34'W, 3100 m, 21 September 1991, *Fjeldså s.n* (GOET!). Tomina, Bajando de Sombreros (Cordillera Mandinga) hacia el Muncipio de Azurduy, 19°56'57"S, 064°31'24"W, 2758 m, 14 October 2007, *Cervantes 175* (HSB, MO!). Yamparaez, Sucre, ca. 50 kms. hacia Tarabuco, 3270 m, 07 March 1981, *Beck 6203* (MO!). **Potosí**: Chayanta, Ravelo 20 kms. hacia Sucre, 3200 m, 30 September 1983, *Beck 9343* (GOET!, LPB, MO!). Jose M. Linares, Serranía entre Lajas y Tambillo, 3900 m, 05 April 1993, *Torrico 344*; *345* (LPB); a 2 km de Lajas hacia Tambillo, Serranía de Mataca, 3930 m, 05 April 1993, *Torrico 348* (LPB); Guerraloma, 2800 m, 01 May 1959, *Cárdenas 5724* (US!). **Tarija**: Arce, de Camacho subiendo hacia Rejera, 2600 m, 01 November 1987, *Beck 14309* (GOET!, LL, LPB, MO!, TEX). Mendez, Cerca Trancas, 3000 m, 13 May 1986, *Bastian 1313* (GOET!); Strasse Carichi-Mayu-Leon Cancha, Passhohe, 2800 m, 01 February 1982, *Gerold 97* (GOET!, LPB); Above Tarija on road to Villazon, 21°29'S, 064°55'W, 2900 m, 17 September 1991, *Kessler 3109* (AAU!); 35 km W Tarija on road to Villazón, 21°29'S, 064°55'W, 3000 m, 20 September 1991, *Kessler 3165* (GOET!); 40 km W Tarija on road to Villazón, 21°29'S, 064°55'W, 3300 m, 20 September 1991, *Kessler 3166* (GOET!). O’Connor, Tarija 53 kms. hacia Entre Ríos, 2360 m, 22 October 1983, *Beck 9641* (AAU!, MO!); ca 5 km pass on Tarija-Villa Montes road, 21°27'S, 064°22'W, 2500 m, 18 September 1991, *Kessler 3110* (AAU!, GOET!, LPB); ca 6 Km E of pass on Tarija-Entre Ríos road., 21°27'S, 064°26'W, 2300 m, 20 September 1991, *Kessler 3152* (GOET!, LPB, MO!); *3154*; *3155*; *3156* (AAU!, GOET!, LPB); *3431*; *3432* (GOET!); ca. 70 km on road from Tarija to Entre Rios, 2200 m, 20 September 1991, *Kessler 3661* (MO!). Tucumilla, pr. Tarija, 2600 m, *Fiebrig 2020* (A!, B, G, MO!, P!); 21°27'S, 064°26'W, 2800 m, 20 September 1991, *Kessler 3153* (GOET!, LPB); Cuenca del Río Camacho, entrando por el Río Lanurejoy, subiendo al margen izquierdo del río, frente a la Comunidad de Camacho, 2490 m, 20 December 1987, *Liberman 1514* (GOET!); entrando por el Río Carbonejo, 2520 m, 03 February 1988, *Liberman 2019* (GOET!); Rincón de la Victoria, 2000 m, 07 November 1974, *Zuerpe 5126* (NY).

### ﻿37. Polylepis pallidistigma

Taxon classificationPlantaeRosalesRosaceae

Bitter, Bot. Jahrb. Syst. 45: 645. 1911.

BE81CBD0-1A9C-56FB-854E-A55D71EAE268

Figs 97 , 98

#### Type.

Peru. Puno: Prov. Azangaro, Muñani, ca. 3650 m, *Weberbauer 1369* (holotype: B destroyed; isotypes: F!, GOET!).

**Figure 97.**
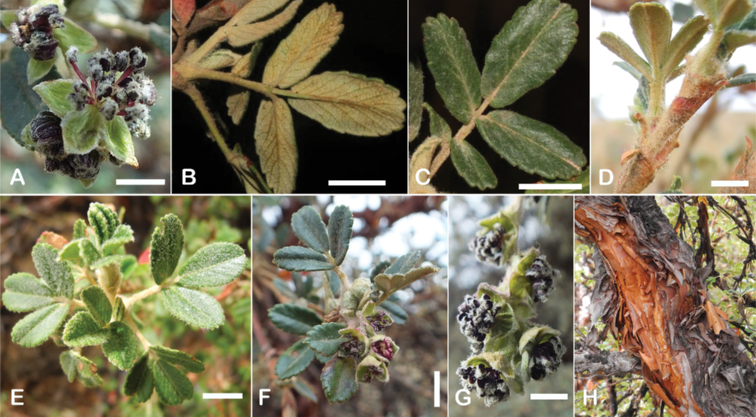
*Polylepispallidistigma* Bitter **A** flowers **B** lower leaf surface **C** upper leaf surface **D** stipule sheaths **E** leaves **F** flowering branch **G** inflorescence **H** bark. Scale bars: 3 mm (**A**); 1 cm (**B, C**); 2 cm (**F**) 5 mm (**G**). Photographs **A, D–H** E.G. Urquiaga Flores **B, C** W. Arque.

#### Description.

***Trees*** 2–13 m tall. ***Leaves*** slightly congested at the branch tips, imparipinnate with 1(–2) pairs of leaflets, obtrullate in outline, 2.6–4.0 × 2.8–4.0 cm; rachises densely villous, points of leaflet attachment with a tuft of long hairs; stipular sheaths apically truncate, densely villous on the outer surfaces; leaflets elliptic in outline, second pair from the terminal leaflet the largest, one of this pair 1.2–2.0 × 0.5–0.8 cm; margin crenate with 5–8 teeth, apically round or emarginate, basally unequally cordate; upper leaflet surfaces slightly rugose, glabrous to sparsely villous; lower leaflet surfaces with a dense layer of very short, white pannose hairs. ***Inflorescences*** pendant, 2.7–6.0 cm long, bearing 5–6 flowers; floral bracts 4.2–4.9 mm long, narrowly triangular, densely villous on the outer surface; rachises villous. ***Flowers*** 6.8–9.2 mm diam.; sepals 4, ovate, green, densely villous outside; stamens 17–21, anthers orbicular, with a dense tuft of straight white hairs on the upper half; styles fimbriate, 3.3–3.6 mm long. ***Fruits*** turbinate, with 2–3 irregular flattened ridges with a series of spines, densely villous; 4.4–5.9 × 3.8–5.9 mm including spines. ***Tetraploid***.

**Figure 98.**
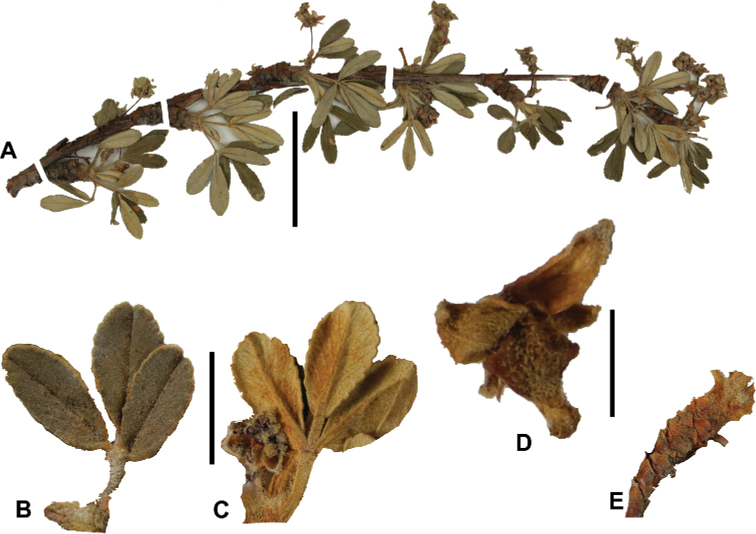
*Polylepispallidistigma* Bitter **A** flowering branch **B** upper leaf surface **C** lower leaf surface **D** fruit **E** stipular sheaths (**A***Boertman 139***B, C, E***Fernandez 544***D***Tovar 5342*). Scale bars: 6 cm (**A**); 2 cm (**B, C**); 5 mm (**D**). Photographs by T. E. Boza E.

#### Distribution, habitat and ecology.

*Polylepispallidistigma* is distributed in southern Peru from southern Cusco to Puno (Fig. 103). It grows on relatively dry slopes at 3700–4580 m elevation in a region with calcareous rocks. It typically grows on rocky outcrops (Yallico 1992, as *P.incana*). Average tree height of *P.pallidistigma* is 2.5–5.0 m with diameters of 5–15 cm (ECOAN 2006). *Polylepispallidistigma* patches harbour some of the most specialized and endangered of bird species, such as *Anairetesalpinus* (Endangered) and *Leptasthenurayanacensis* (Near Threatened) in the forests of Lawa Lawani and Chingo (Carabaya Province), *Asthenesarequipae* and *Leptasthenuraandicola* in the Torno (Huancane Province), Bellavista and Quilcapuncu forests (San Antonio de Putina Province) and *Conirostrumbinghami* (= *Oreomanesfraseri*) (Near Threatened) present in all evaluated forests (ECOAN 2006).

#### Conservation status.

The EOO for *Polylepispallidistigma* is estimated as 17,670 km^2^, the AOO is assessed at 112 km^2^ and it is known from 17 locations. No protection action has been taken to date. We assess *P.pallidistigma* as Vulnerable (B1a+B2a).

#### Notes.

*Polylepispallidistigma* was treated as a synonym of *P.besseri* by previous authors (e.g., Simpson 1979; Kessler 1995b) and a synonym of *P.subtusalbida* by Mendoza and Cano (2012). However, based on its distinct morphology, ecology and distribution, we consider that this taxon should be recognized as different from *P.besseri* and *P.subtusalbida*. *Polylepispallidistigma* differs from *P.besseri* by having longer and narrower elliptic leaflets 1.2–2.0 × 0.5–0.8 cm with the lower leaflet surfaces with a dense layer of very short of pannose hairs, whereas *P.besseri* has shorter and broader obovate leaflets 1.4–1.6 × 0.6–1.1 cm with the layer of pannose hairs and mixed with densely tomentose hairs. Further, *P.pallidistigma* has a lower number of flowers per inflorescence (5–6) and longer styles (3.3–3.6 mm), whereas *P.besseri* has 7–9 flowers and styles 2.5–3.1 cm long. *Polylepispallidistigma* differs from *P.subtusalbida* by larger elliptic leaflets (1.2–2.0 × 0.5–0.8 cm versus 0.9–1.6 × 0.4–0.6 cm) with different margins (crenate versus serrate), different hair type of the lower leaflet surfaces (pannose versus tomentose), relatively longer inflorescences (2.7–6.0 cm versus 1.8–3.7 cm) with more flowers per inflorescence (5–6 versus 3–4) and longer styles (3.3–3.6 mm versus 2.8–3.4 mm).

#### Specimens examined.

**Peru. Cusco**: Espinar, Zona del Mamaniwaita, 3900–4100 m, 13 July 1967, *Vargas 19893* (CUZ!). **Puno**: Azangaro, Putina, 4200 m, 04 April 1959, *Vargas 12528* (CUZ!); Carabaya, Coasa, Chingo, 14°02'25"S, 70°08'07"W, 4299 m, 07 October 2014, *Boza 3005* (USM!, Z!); Coasa. Chingo, 14°02'49"S, 070°09'19"W, 4511 m, 07 October 2015, *Boza 3006* (USM!, Z!); Entre Macusanin Nuñoa, 4000 m, 29 February 1948, *Vargas 7139* (CUZ!, MO!). Huancane, rock outcrop 450 m East of the Huancane coliseo, 15°11'40"S, 069°45'26"W, 3910 m, 06 November 2012, *Sylvester 1822* (CUZ!, Z!); 900 m East up the slope from the Huancane coliseo in the centre of the main *Polylepis* forest, 15°11'32"S, 069°45'21"W, 3999 m, 06 November 2012, *Sylvester 1825*; *1827* (CUZ!, Z!); 700 m East up the slope from the Huancane coliseo in the centre of the main *Polylepis* forest, 15°11'35"S, 069°45'26"W, 3927 m, 06 November 2012, *Sylvester 1829* (CUZ!, Z!); 500 m East up the slope from the Huancane coliseo in the centre of the main *Polylepis* forest, 15°11'38"S, 069°45'28"W, 3892 m, 06 November 2012, *Sylvester 1830*; *1831* (CUZ!, Z!). Lampa, Quebrada Metara, 15°10'S, 070°22'W, 3880 m, 01 April 1987, *Boertmann 136*; *137*; *138*; *139* (AAU!); Pampa Changanchaca between Lampa and Pucara, 15°10'S, 070°22'W, 3800 m, 02 April 1987, *Boertmann 141* (AAU!); Inticancha in lower Quebrada Metara N of Centro Yaurinco, 15°10'S, 070°22'W, 3850–4000 m, 01 April 1987, *Brandbyge 569*; *573* (AAU!); Cerro Chacapacha, 3900–4000 m, 21 May 1988, *Del Carpio 728*; *729* (USM!); Cara-cara, cerca a Pucará, 3900 m, 27 September 1984, *Fernández 544* (USM!); Inticancha in lower Quebrada Metara N of Cerro Yaurinco, 15°10'S, 070°22'W, 3850–4000 m, 01 April 1987, *Kessler 573* (AAU!); Areas rocosas, Lamparaquen, 15°19'00"S, 070°27'00"W, 4130 m, 06 June 2009, *Montesinos 2675* (USM!); SW facing slope 900 m NE of a large lake 8.5 km East of Lampa, close to the hamlet Chañacahua, 15°23'11"S, 070°17'22"W, 3986 m, 06 November 2012, *Sylvester 1802* (CUZ!, Z!); Base of south facing cliff on the south facing slope overlooking the hamlet of Chañacahua, 15°22'54"S, 070°17'04"W, 4075 m, 06 November 2012, *Sylvester 1816* (CUZ!, Z!); Alrededores de Pucará, 3850–3900 m, 12 March 1966, *Tovar 5342* (USM!); Laderas de Pucará, 3850 m, 03 November 1966, *Vargas 18293* (CUZ!); Lampa, 3900–4400 m, 25 March 1988, *Velásquez 1*; *2*; *3*; *4*; *8*; *9*; *11* (MO!); Melgar, Ñuñoa, 14°28'24"S, 070°35'43"W, 4038 m, 06 October 2014, *Boza 3003* (USM!, Z!); Ñuñoa, 14°25'38"S, 070°34'01"W, 4094 m, 06 October 2014, *Boza 3004* (USM!, Z!). Palca, Palca, 15°15'08"S, 070°32'05"W, 3960 m, 04 April 2005, *Aedo 11124* (USM!). Puno, Slopes east of Puno, 3800 m, 29 January 1975, *Antunez de Mayolo 87* (USM!); On dry places, 3125 m, 02 January 1920, *Shepard 150* (A!). San Antonio de Putina, Quilcapuncu, 14°54'20"S, 069°45'18"W, 3952 m, 08 October 2014, *Boza 3007* (CUZ!, USM!); Near Puno, 4000 m, 01 July 1936, *Soukup 365* (F!).

### ﻿38. Polylepis rugulosa

Taxon classificationPlantaeRosalesRosaceae

Bitter, Bot. Jahrb. Syst. 45: 638. 1911.

E2AEE210-567F-5DBC-BD54-80D7DC7D5557

Figs 99 , 100

Polylepis tenuiruga Bitter, Bot. Jahrb. Syst. 45: 635. 1911. Type. Chile. Without precise locality, *Besser s.n* (holotype: B destroyed, photos at F!, GH, NY!).

#### Type.

Peru. Arequipa: Pampa behind train station, Arequipa to Puno line, 3800 m, *Weberbauer 4881* (holotype: B destroyed; isotype: WRAT!).

**Figure 99.**
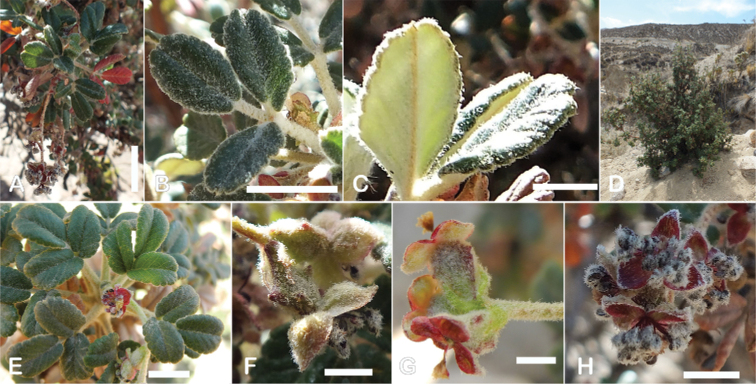
*Polylepisrugulosa* Bitter **A** flowering branch **B** upper leaf surface **C** lower leaf surface **D** habit **E** leaves **F** fruit **G** fruit and style **H** flowers (**A–C, H***Boza & Urquiaga 3010***D–G***Boza & Urquiaga 3012*). Scale bars: 2 cm (**A**); 1 cm (**B, E**); 5 mm (**C, H**); 3 mm (**F, G**). Photographs **A–D, F, H** E.G. Urquiaga F. **E, G** T.E. Boza E.

#### Description.

***Trees*** 2–12 m tall. ***Leaves*** slightly congested at the branch tips, imparipinnate with pair of leaflets, obtrullate in outline, 2.0–2.8 × 1.6–2.5 cm; rachises densely tomentose, points of leaflet attachment with a tuft of long hairs; stipular sheaths apically truncate, densely tomentose on the outer surfaces; leaflets broadly obovate in outline, second pair from the terminal leaflet the largest, one of this pair 1.2–1.6 × 0.6–0.9 cm; margin crenate with 5–8 teeth, apically obtuse or emarginate, basally unequally attenuate; upper leaflet surfaces strongly rugose, glabrous; lower leaflet surfaces densely tomentose with whitish hairs 0.8–1.0 mm long, mixed with a dense layer of very short, white pannose hairs. ***Inflorescences*** pendant, 4.2–7.8 cm long, bearing 4–5 flowers; floral bracts 4.6–4.9 mm long, narrowly triangular, densely tomentose on the outer surface; rachises tomentose. ***Flowers*** 6.7–9.9 mm diam.; sepals 4, ovate, green, densely tomentose outside; stamens 11–15, anthers orbicular, with a dense tuft of straight white hairs on the upper half; styles fimbriate, 2. 1–3.5 mm long. ***Fruits*** turbinate, with 2–3 irregular flattened ridges with a series of spines, densely villous; 4.5–6.2 × 4.9–7.5 mm including spines. ***Tetraploid***.

**Figure 100.**
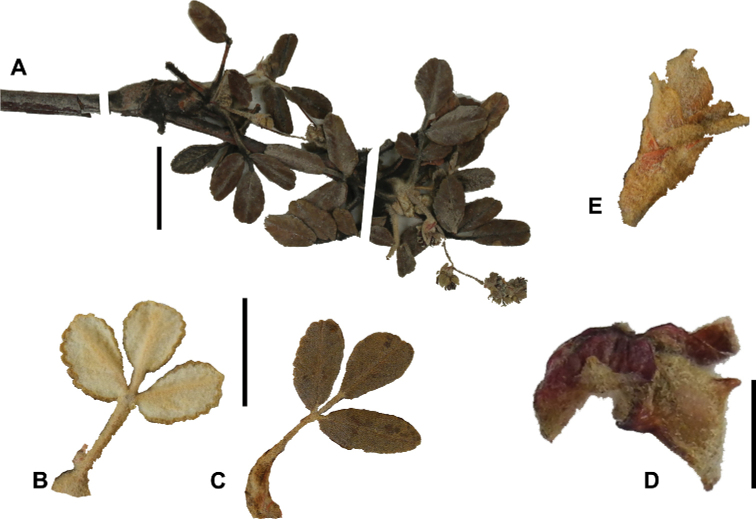
*Polylepisrugulosa* Bitter **A** flowering branch **B** lower leaf surface **C** upper leaf surface **D** fruit **E** stipular sheaths (**A***Boertman 145***B, D, E***Blanchard s.n***C***Rodriguez 4*). Scale bars: 2 cm (**A–C**); 3 mm (**C, D**). Photographs by T. E. Boza E.

#### Distribution, habitat and ecology.

*Polylepisrugulosa* is distributed in Peru from western Arequipa, southern Cusco, Moquegua and western Tacna to northern Chile (Arica and Parinacota) (Fig. 103). It occurs in very dry Andean habitats with rocky soils at 2900–4550 m elevation. In Peru, average tree height of *P.rugulosa* is 1.0–1.5 m, with diameters typically less than 10 cm and tree density of 583 individuals ha^-1^ (Rodríguez 2018). The highest density of adult trees and seedlings was found at 4300 m elevation (38% slope) and the lowest density and coverage at 4180 m elevation (20% slope), suggesting a preference for areas with steeper slopes or higher presence of rocks and shrubs. Seedling density was significantly higher under the crowns of *Polylepis* trees than under isolated bushes (Rodríguez 2018). *Polylepisrugulosa* populations in the pre-altiplano zone on the western slopes of the Andean Cordillera of northern Chile have an age structure that suggests highly episodic seedling establishment, due to sequences of wet years (Rundel et al. 2003). The maximum photosynthetic capacity of *P.rugulosa* in the wet season is 6.4 μmol m^-2^s^-1^, although it can maintain relatively high rates of photosynthesis throughout the year (Rundel et al. 2003). Furthemore, *P.rugulosa* responds to elevation by changing in morphology (short trees with small leaves) and ecophysiological responses (decreased transpiration rate, higher nutrient concentration and enrichment in the ^13^C isotope suggesting more water stress) (Macek et al. 2009). In a population at 3700 m elevation, *P.rugulosa* had 34% higher total chlorophyll content during the morning hours, apparently due to *de novo* synthesis of both Chl*a* and Chl*b* (García-Plazaola et al. 2015). In a dendrochronological study in Parinacota (Chile), average radial growth of *P.rugulosa* was 0.7 mm a^-1^ (Silva 2012). Growth of *P.rugulosa* is determined by precipitation during the wet season of the preceding and growth years (Jomelli et al. 2012). Based on Amplified Fragment Length Polymorphisms (AFLP), extensive gene flow has been found to occur within and between Chilean populations (Schmidt-Lebuhn et al. 2006b). *Polylepisrugulosa* patches harbour specialized and endangered bird species (Miranda and Cardozo 2008; Samata et al. 2019).

#### Conservation status.

The EOO for *Polylepisrugulosa* is estimated as 35,293 km^2^, the AOO is assessed at 172 km^2^ and it is known from 24 locations. The extent of *P.rugulosa* forests in Arequipa (Peru) is estimated at 76,566 ha, where large forests are found around Ampato Volcano (20,114 ha), Pichupichu Volcano (5,395 ha) and at the high Andes of Yura (Esquerra 3,249 ha and Palca 1,071 ha) (Jururo 2018). Only small extents of *P.rugulosa* forest are currently protected in Chile within Lauca and Volcán Islunga National Parks, Las Vicuñas National Reserve and in Peru in Chili-Quilca (Arequipa) in the buffer zone of Salinas y Aguada Blanca National Reserve. The species was categorized as VU (A1c) in the World List of Threatened Trees (Oldfield et al. 1998). In Peru, it has been categorized as VU (SERFOR 2006) and, in Chile, as VU (Benoit 1989; Miranda and Cardozo 2008). *Polylepisrugulosa* has been harvested for fuel in the early 20^th^ century and is severely impacted by the mining activity in Chile (Rundel et al. 2003). We assess *P.rugulosa* as Vulnerable (B1a+B2a, C1).

#### Notes.

*Polylepisrugulosa* is similar to *P.subtusalbida*, but has just one lateral leaflet pair and broader leaflets (0.6–0.9 mm wide) with crenate margins, whereas the latter has 1(–2) pairs and narrower leaflets (0.4–0.6 mm wide) with serrate margin. *Polylepisrugulosa* also has longer inflorescences (4.2–7.8 cm) with 4–5 flowers and 11–15 stamens (*P.subtusalbida* 1.8–3.7 cm, 3–4 flowers and 19–21 stamens). Mendoza and Cano (2012) considered that both *P.rugulosa* and *P.subtusalbida* occur in southernmost Peru, but according to our assessment of these species, only *P.rugulosa* occurs in this region, whereas *P.subtusalbida* is endemic to central Bolivia. For additional morphological similarities, see under *P.besseri*.

#### Specimens examined.

**Chile. Arica and Parinacota**: Putre, 3500 m, 02 May 1987, *Fjeldså s.n* (AAU!). **Tarapacá**: Región de Tarapacá, Strasse von Zapahuira zum Portezuelo de Chapiquiña, s.d., *Hellwig 513* (G).

**Peru. Arequipa**: Arequipa, Cerca a Chiguata. SE de Arequipa, 3000–3500 m, 23 September 1966, *Arenas 34* (USM!); Dist. Pocsi; Tuctumpaya, 3200 m, 03 April 2003, *Cáceres 3212* (USM!); El Cimbral-Chiguata, 3900 m, 09 November 1996, *Cáceres 47* (CUZ!); **Arequipa/Puno**:. Sihuata-La Cumbre, 3800–4000 m, 13 November 1947, *Ferreyra 2594* (GOET!, MO!, US!, USM!); Chiguata, arriba de Miraflores, 3800 m, 10 November 2002, *Quipuscoa 2810* (F!); Chiguata, 16°23'11"S, 071°20'19"W, 4000–4250 m, 13 February 2003, *Quipuscoa 2857* (F!); Simbral, carretera de Chiguata-Juliaca, 3500–4000 m, 30 May 1999, *Roque 880* (USM!); road to Chacayani to Chiva, 16 January 1977, *Simpson 8570c*; *8570d* (USM!); Hacia el Cimbral, 3750 m, 11 April 1959, *Vargas 12687* (CUZ!); Chiguata-Cimbral, 3700–4000 m, 13 October 1976, *Vargas 22849* (CUZ!). Caylloma, Huambo. Dist. de Huambo, 4000 m, 03 October 1990, *Rodriguez 2* (USM!); Dist. de Huambo, 4000 m, 01 August 1990, *Rodriguez 3* (USM!); Huambo, Dist. Huambo, 3800–4200 m, 01 August 1990, *Rodriguez 4* (USM!); *5* (USM!). Cayma, Estancia Cabrarias. Chacani. Norte de Arequipa, 3500–4000 m, 14 January 1966, *Arenas 12* (USM!); Nevado Chachani, along dry stream-beds in open rocky valley, 4000–4200 m, 14 April 1925, *Pennell 13295* (F!, US!); Volcan Chachani, on the road to the summit. Km 32 on the road from Chachani to Chivay in shallow ravines, 16 January 1977, *Simpson 8570* (A!, MO!); *8570b* (USM!). Chiguata, 10 km above Chihuata (W slope of Misti), 16°24'S, 071°24'W, 4270 m, 07 April 1987, *Boertmann 144*; *145*; *146*; *147* (AAU!). Condesuyos, alturas cordilleranas, 4200–4600 m, 24 April 1967, *Vargas 19466* (CUZ!). Cotahuasi, Camino a Cotahuasi, 3735 m, 30 June 2004, *Cáceres 3824* (USM!). Huambo, Huambo (Quebrada Sau Sau), 4000 m, 29 August 1987, *Espinoza 7* (USM!). La Union, Dist. Cotahuasi; vista del condor, 3200 m, 05 June 2002, *Cáceres 5457* (USM!). Orcopampa, Orcopampa-Huancurama, 3875 m, 17 August 1987, *Huamani 11* (USM!). Pampacolca, Chuquibamba-Pampacolca, 4000m, 13 August 1987, *Huamani 8* (USM!). Yura, Yura-Huanca, 3200 m, 09 June 1999, *Roque 1048* (USM!); Quebrada Chontahuayco y Monte Barranco, 4020 m, 01 August 1987, *Espinoza 1* (USM!); 4050 m, 11 August 1987, *Espinoza 5* (USM!); Nevado de Coropuno, 06 May 1987, *Fjeldså s.n* (AAU!); Between lake Salinas and Arequipa, 3962 m, 01 February 1943, *Sandeman 3816* (F!). **Cusco**: Ushcopata Valley, above Sicuani, 3700 m, 09 April 1913, *Cook 123* (US!). **Moquegua**: Gral. Sanchez Cerro, Dist. Ubinas, Zona Silvestre Querapi, 3880 m, 25 January 2004, *Blanchard s.n* (USM!); Dist. Ubinas, Tassa, camino en Mollemoco, 3800 m, 12 July 2006, *Montesinos 1185* (USM!); Ladera de Mollemoco, Tassa Dist. Ubinas, 16°11'00"S, 070°42'00"W, 3690 m, 01 June 2011, *Montesinos 3175* (USM!). Mariscal Nieto, Entre Chuculay y Qda. Cuellar, 3550–3600 m, 16 December 1995, *Arakaki 257* (USM!); Carumas, 16°52'18"S, 070°39'49"W, 4086 m, 09 October 2014, *Boza 3010* (USM!, Z!); Torata, Quebrada Cuellar, 16°57'57"S, 070°41'02"W, 4224 m, 09 October 2014, *Boza 3011* (USM!, Z!); Between Torata and Carumas, km 75–76 from Moquegua to Puno, 3600 m, 14 February 1983, *Dillon 3347* (F!, MO!, US!); ca. 67 km NE of Moquegua on road to Carumas, 3680 m, 15 November 1986, *Dillon 4807* (F!, USM!); Cordillera above Torata, forming grove along brook, 3900–4000 m, 14 February 1925–15 February 1925, *Weberbauer 7470* (F!, US!). Torata, Ponton Cuellar, 16°59'15"S, 070°41'38"W, 3950 m, 12 April 2005, *Aedo 11309* (USM!). **Tacna**: Candarave, Candarave, Comunidad Viltahuira, 3600 m, 25 October 2000, *Cáceres 82* (USM!); Candarare, Yucamani, 3100–3400 m, 09 December 1997, *La Torre 1987* (MO!, USM!); Tarata, Susapaya, 3950–4100 m, 06 December 1997, *Arakaki 762* (MO!, USM!); Ticaco, 17°25'26"S, 070°03'27"W, 3458 m, 10 October 2014, *Boza 3012* (USM!, Z!); Poma, 3900–4430 m, 04 December 1997, *Cano 7962* (MO!, USM!); Cordillera Barroso, 4200–4580 m, 26 March 1998, *Cano 8166* (MO!, USM!); *8167*; *8245* (USM!); Ticaco, 3600–4000 m, 31 March 1998, *Cano 8334* (USM!); Ticaco (Quebrada Ticalaco), 3300–3800 m, 16 June 1998, *Cano 8420* (USM!); Cerros al SE de la Cordillera barroso, 4000–4270 m, 28 March 1998, *La Torre 2153* (USM!); 4 km northeast of Tarata, 3840 m, 27 January 1952, *Pearson 33* (F!); Entre Tarata y Chila, 3850 m, 31 January 1984, *Rivas s.n* (USM!); De Tarata a Chila, 3590 m, 01 January 1984, *Rivas s.n* (USM!); Bajando a Tarata, 4300 m, 06 April 1959, *Vargas 12557* (CUZ!); bajando Tarata, 4300 m, 06 April 1959, *Vargas 12557* (CUZ!); bajando Tarata, 3900–4100 m, 05 August 1967, *Vargas 19927* (CUZ!); Candarave, 21 February 2009, *Morales 1* (CUZ!); Cala-cala, 4100 m, 21 October 1976, *Bernardi 16739* (F!); Pampa Arrieros Arequipa-Puno, 1901–1929, *Weberbauer 4841* (B, F!, MO!).

### ﻿39. Polylepis subtusalbida

Taxon classificationPlantaeRosalesRosaceae

(Bitter) M. Kessler & Schmidt-Leb. Organisms Diversity Evol. 6(1): 69. 2006.

3066FCFD-1F7B-5BE3-A0F1-5D808A688FD4

Figs 101 , 102

Polylepis besseri subsp. subtusalbida (Bitter) M. Kessler, Candollea 50: 154. 1995. Type. based on Polylepissubtusalbida (Bitter) M. Kessler & Schmidt-Leb.
Polylepis racemosa var. tomentosa Kuntze, Revis. Gen. Pl. 3(3): 77. 1898. Type. Bolivia. Cochabamba: between Challa and Tapacari, 3600–4000 m, 18 Mar 1892, *Lorenz & Hieronymus s.n* (holotype: B destroyed; isotypes: NY!, UC!, US!).
Polylepis besseri var. abbreviata Bitter, Bot. Jahrb. Syst. 45: 628. 1911. Nom. illeg. (based on the type of P.racemosavar.tomentosa Kuntze).

#### Basionym.

Polylepisincanasubsp.subtusalbida Bitter, (1911:640).

**Figure 101.**
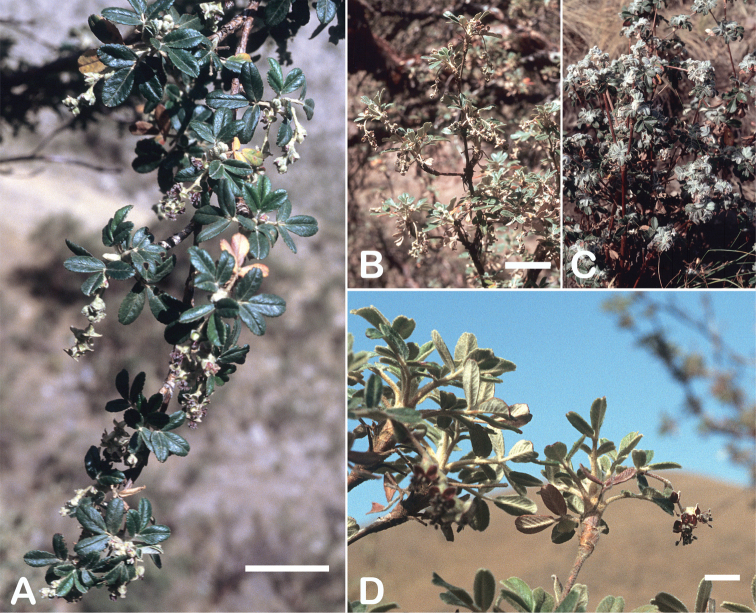
*Polylepissubtusalbida* (Bitter) M. Kessler & Schmidt-Leb **A** fruiting branch **B** flowering branch **C** leaves **D** leaves. Scale bars: 2 cm (**A, B**); 1 cm (**D**). Photographs by M. Kessler.

#### Type.

Bolivia. Cuesta Duraznillos, 2400–2600 m, Dec 1907, *Herzog 712* (holotype: Z!).

**Figure 102.**
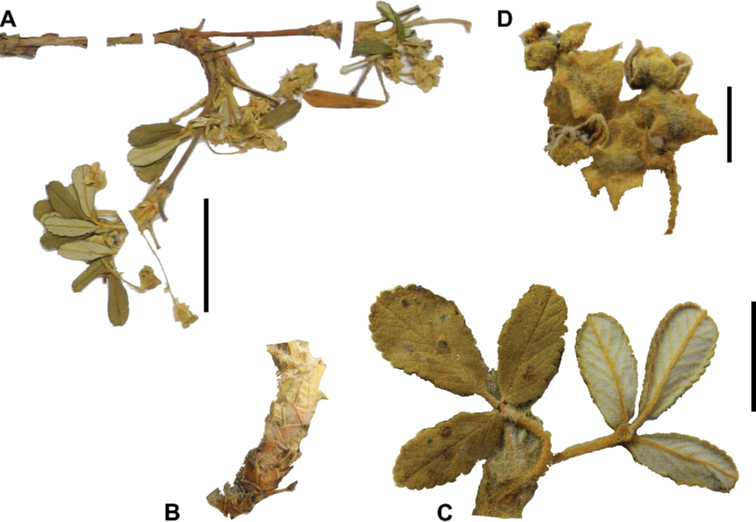
*Polylepissubtusalbida* (Bitter) M.Kessler & Schmidt-Leb **A** flowering branch **B** stipular sheaths **C** upper & lower leaf surfaces **D** fruits (**A, D***Kessler 3414***B***Kessler 3556***C***Kessler 3648*). Scale bars: 4 cm (**A**); 1.5 cm (**C**); 8 mm (**D**). Photographs by T. E. Boza E.

#### Description.

***Trees*** 3–10 m tall. ***Leaves*** slightly congested at the branch tips, imparipinnate with 1(–2) pairs of leaflets, obtrullate in outline, 1.8–2.1(–2.9) × 1.6–2.2 cm; rachises densely tomentose, points of leaflet attachment with a tuft of long hairs; stipular sheaths apically truncate, sparsely to densely tomentose on the outer surfaces; leaflets obovate in outline, second pair from the terminal leaflet the largest, one of this pair 0.9–1.6 × 0.4–0.6 cm; margin serrate with 7–10 teeth, apically obtuse or emarginate, basally unequally attenuate; upper leaflet surfaces smooth to slightly rugose, glabrous to sparsely tomentose, lower leaflet surfaces densely tomentose hairs 0.5–1.2 mm long, mixed with a dense layer of very short, white pannose hairs. ***Inflorescences*** pendant, 1.8–3.7 cm long, bearing 3–4 flowers; floral bracts 3.9–4.8 mm long, narrowly triangular, densely tomentose on the outer surface; rachises densely tomentose. ***Flowers*** 7.7–8.2 mm diam.; sepals 4, ovate, green, densely tomentose outside; stamens 19–21, anthers orbicular, with a dense tuft of straight white hairs on the upper half; styles fimbriate, 2.8–3.4 mm long. ***Fruits*** turbinate, with 2–5 irregular flattened ridges with a series of spines, sparsely tomentose; 6.8–8.2 × 4.4–5.3 mm including spines. ***Tetraploid***.

#### Distribution, habitat and ecology.

**Figure 103.**
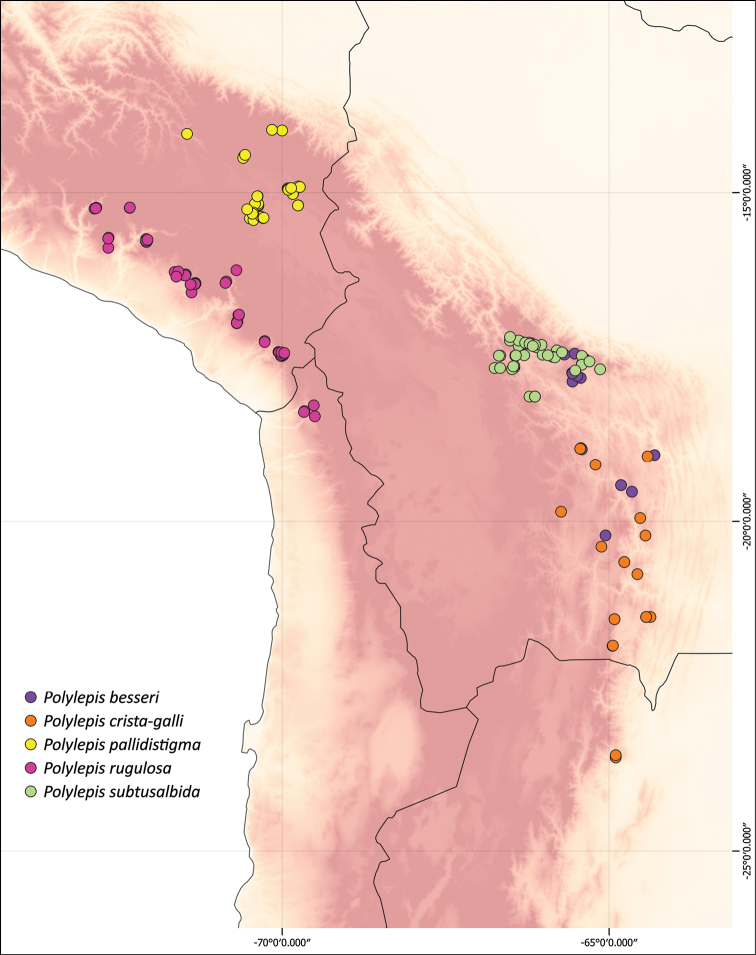
Geographical distribution of the species of subsection Besseria.

*Polylepissubtusalbida* is distributed along the northern and western margins of the Cochabamba Basin and adjacent Potosí in Bolivia (Fig. 103). The species occurs in relatively dry areas at 2650–4450 m elevation. The vegetation, ecology and conservation of these forests has been studied in detail by Hensen (1994, 1995, 2002). Hybrids between *P.subtusalbida* and *P.incanoides*, *P.besseri* and *P.lanata* have been reported in Cochabamba (Kessler 1995b).

#### Conservation status.

The EOO for *Polylepissubtusalbida* is estimated as 149,135 km^2^, the AOO is assessed at 164 km^2^ and it is known from 29 locations. It is represented by numerous stands varying in extent and structure on the mountainous slopes in Tunari National Park and stands surrounding Chayanta, Queñoani and Colcha, except for the Tiraque mountain range where these forests have been practically exterminated by the land use of the dense populations historically settled in the area (logging, crops, livestock and annual grasslands burns) and reduced to scattered remnants in the form of isolated individuals or small groups in very steep topography (Navarro et al. 2010). *Polylepissubtusalbida* is formally protected within Tunari National Park where large areas of *Polylepis* forest have been planted with *Eucalyptusglobulus* and *Pinusradiata* (Navarro et al. 2010). It was classified as VU (B1b(ii,iii)) in the Red List of Threatened Flora of Bolivia (Arrázola and Coronado 2012). We assess *P.subtusalbida* as Vulnerable (A1, B1a+B2a).

#### Notes.

For morphological similarities, see under *P.crista-galli*, *P.pallidistigma*, and *P.rugulosa*.

#### Specimens examined.

**Bolivia. Cochabamba**: Arani, Cuenca a Infiernillos, 3800 m, 24 February 1991, *Hensen 1012* (GOET!, LPB); Alalay, Cabecera de Valle, 3700 m, 28 March 1991, *Hensen 2063* (GOET!, LPB); Mojon 1 km N Cochabamba-Sta Cruz road, 17°29'S, 065°25'W, 3000 m, 14 August 1991, *Kessler 2941*; *2942* (AAU!, GOET!, LPB). Arque, Quillacollo 59 km hacia Oruro, 3580 m, 31 March 1979, *Beck 933* (LPB); 79 Km from Cochabamba on road to Oruro, 17°41'S, 066°29'W, 3750 m, 28 August 1991, *Kessler 2999*; *3000* (GOET!, LPB, MO!); km 68 on the road from Cochabamba to La Paz, 17°38'S, 066°27'W, 3500 m, 25 October 1985, *Solomon 14353*; *14535* (LPB, MO!). Carrasco, Rodeo Grande, at km 140 on road from Cochabamba to Santa Cruz., 3000 m, 09 February 1971, *Hawkes 4400* (MO!); Zapata Rancho, 3300 m, 19 March 1991, *Hensen 1918* (LPB); Incallajta ruins, 15 km S Monte Punco, 17°37'S, 065°25'W, 3000 m, 15 August 1991, *Kessler 2981* (GOET!, LPB). Cercado, Sapanani Alto, 3600 m, 07 January 1991, *Hensen 2332* (LPB); Sapanani Alto, 3800 m, 07 April 1991, *Hensen 2333* (GOET!, LPB); Sapanani, 3600 m, 28 July 1990, *Hensen 855* (LPB); Sacaba, steep slope above Río Hura Hura; ca. 1300 m beyond the junction with Quebrada Kuhlu, 3400 m, 17 March 1994, *Ritter 645* (GH!); Quebrada Chaqui Mayu above the City of Cochabamba, 3600 m, 15 April 1994, *Ritter 818* (GH!); Ladera Sur del Parque Nacional Tunari, 17°16'37"S, 066°19'09"W, 3400 m, *Terán 4526* (BOLV). Chapare, Melgar 2 km hacia Punata, 17°24'S, 065°48'W, 3400 m, 14 January 1995, *Beck 21712* (GOET!); Parque Tunari, 3700 m, 02 August 1990, *Hensen 875* (LPB); Parque Tunari, 3400 m, 02 August 1990, *Hensen 878* (GOET!, LPB); ca. 20 km above Sacaba on road to Palca, 17°19'S, 066°02'W, 3750 m, 13 August 1991, *Kessler 2940* (GOET!). Mizque, Totora 35 kms hacia Cochabamba, 3000 m, 28 March 1979, *Beck 861* (LPB, MO!). Morochata, P. N. Tunari, directly N of Cochabamba, 3650 m, 23 July 1989, *Kessler 216A* (GOET!, Z!). Punata, Camino Melga y Punata, 3500 m, 03 March 1991, *Hensen 1232* (LPB); entee Melga y Punata, 3500 m, 03 March 1991, *Hensen 1264* (GOET!, LPB); Abajo del cerro Tuti, 3800 m, 10 March 1991, *Hensen 1509* (GOET!, LPB); Quillacollo, Cochabamba 24 kms. hacia Morochata, 3180 m, 28 November 1981, *Beck 7395* (GOET!, LPB, MO!); Lampaya, 3600 m, 05 March 1991, *Hensen 1270* (LPB); entre Lampaya y Llanke, 3700 m, 05 March 1991, *Hensen 1315* (LPB); *1319* (GOET!); camino Tiquipaya-Titiri, 3500 m, 09 March 1991, *Hensen 1431* (LPB); entre San Miguel y Titiri, 3600 m, 16 March 1991, *Hensen 1686* (GOET!, LPB); Palca Pampar, 3600 m, 01 April 1991, *Hensen 2150* (LPB); Wayra Loma, 3600 m, 01 April 1991, *Hensen 2215* (LPB); Lapia, 3700 m, 04 April 1991, *Hensen 2300* (LPB); San Miguel, 3600 m, 13 April 1991, *Hensen 2400* (GOET!, LPB); Cerca de Wakaplaya, camino Sipe-Sipe-Kami, 3700 m, 13 October 1988, *Hensen 247* (GOET!, LPB); Tiquipaya-Apote, 3500 m, 13 October 1988, *Hensen 256* (GOET!, LPB); Lapia, camino Tiquipaya-Titiri, 3500 m, 06 December 1988, *Hensen 290* (LPB); Sipe Sipe-Lipichi, 3800 m, 30 March 1989, *Hensen 318* (LPB); Camino a Independencia, San Miguel, 3300 m, 01 August 1990, *Hensen 874* (GOET!, LPB); P.N. Tunari, directly N of Cochabamba, 17°15'00"S, 066°23'00"W, 3650 m, 23 July 1989, *Kessler 216* (AAU!, GOET!, MO!); 40 km after Cochabamba on the road to Morochata, 17°15'01"S, 066°30'59"W, 3390 m, 05 January 1968, *Vuilleumier 468* (GH!, MO!, US!). Tapacari, 68 km hacia Oruro, 3450 m, 03 November 1982, *Beck 9033* (LPB); 68 km hacia Oruro, 3450 m, 03 November 1982, *Beck 9034* (LPB); 74 km before Cochabamba on the road from Oruro, 3600 m, 03 November 1982, *Vuilleumier 474* (G, GH!, US!); Tapacarí, 84 km W of Cochabamba on the paved road La Paz-Cochabamba, 17°40'S, 068°45'W, 3800 m, 18 April 1987, *Brandbyge 668* (AAU!); 72 km W of Cochabamba 0.5 km down a small road (the old Cochabamba road), 17°40'S, 066°40'W, 3730 m, 18 April 1987, *Brandbyge 670* (AAU!); Tiraque, Quebrada alongside Río Talpasale, near to the junction with the road to Rancho Choto, 3680 m, 02 July 1994, *Ritter 1196* (GH!, MO!); Totora, road between Cochabamba and Santa Cruz 107 km from Cochabamba Geviñapampa, 17°34'S, 065°18'W, 3100 m, 21 April 1987, *Brandbyge 711a* (AAU!); P. N. Tunari, directly N of Cochabamba, 3650 m, 23 July 1989, *Kessler 216B*; *216C*; *216D*; *216E* (GOET!); 3600–4000 m, 18 March 1892, *Lorentz s.n* (B, NY, UC, US!); Cercado, Parque Tunari, 3500 m, 06 February 1986, *Pedrotti s.n* (GOET!, MO!). **Potosí**: Alonso de Ibanez, Pichata on Acasi-Uncia road, 18°06'S, 066°13'W, 3950 m, 23 August 1991, *Kessler 3023* (AAU!, GOET!). Gral. Bilbao, 22 km SW Acacio on road to Sacaca and Uncia, 18°06'S, 066°08'W, 3400 m, 22 August 1991, *Kessler 3021* (GOET!); *3412* (GOET!, LPB); *3413*; *3414* (AAU!, GOET!); *3415* (GOET!); *3416* (AAU!, GOET!, LPB, MO!); *3418*; *3419* (AAU!, GOET!); *3553*; *3554*; *3556*; *3557*; *3648*; *3650*; *3651* (GOET!); Cuesta de Duraznillos, 2400–2600 m, 01 December 1907, *Herzog 712* (Z!).

### ﻿Subsection Incanaee

Taxon classificationPlantaeRosalesRosaceae

T.Boza & M.Kessler sect. nov.

18D7C2D0-CABE-50E3-ABF4-949BFC5B58B8

urn:lsid:ipni.org:names:77301650-1

#### Diagnosis.

Trees or shrubs; one lateral leaflet pair; lower leaflet surfaces with a dense layer of very short, white or yellowish pannose hairs; fruits with 2–5 irregular flattened ridges with a series of spines.

#### Type.

*Polylepisincana* Kunth.

#### Note.

The subsectional epithet *Incanae* is a plural adjective agreeing in gender with *Polylepis*.

### ﻿40. Polylepis fjeldsaoi

Taxon classificationPlantaeRosalesRosaceae

T.Boza & M.Kessler sp. nov.

5BD8530A-6AE3-519B-A3C9-FF742678D226

urn:lsid:ipni.org:names:77301651-1

Figs 104 , 105

#### Diagnosis.

This species differs from *Polylepistomentella* Wedd. in having broader leaflets (0.6–0.7 cm vs. 0.3–0.6 cm) with crenate margins (vs. serrate) and a lower number of stamens per flower ((9–)11–17 vs. 19–23).

**Figure 104.**
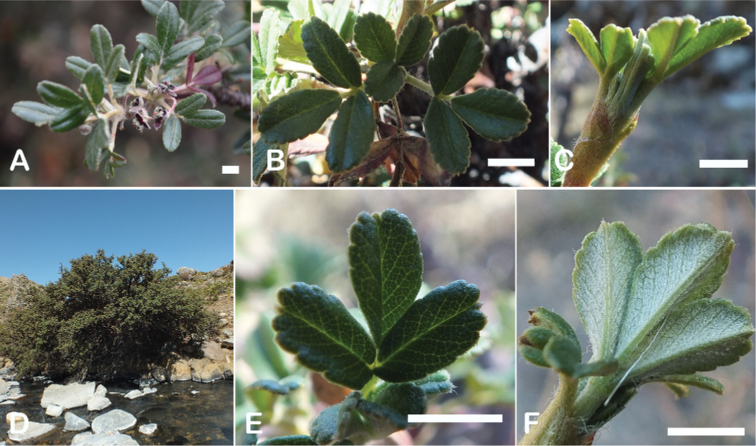
*Polylepisfjeldsaoi* T.Boza & M.Kessler **A** flowering branch **B** leaves **C** stipule sheaths **D** habit **E** upper leaf surface **F** lower leaf surface (**B–F***Boza & Urquiaga 3037*). Scale bars: 5 mm (**A**); 1 cm (**B, C, E, F**). Photographs **A** J. Chambi **B–F** E.G. Urquiaga F.

#### Type.

Peru. Ayacucho, Lucanas, Puquio, Queronta, l4°34'33"S, 74°04'57"W, 3879 m, 1 Jul 2015, *Boza & Urquiaga 3107* (holotype: USM!; isotypes: Z!).

**Figure 105.**
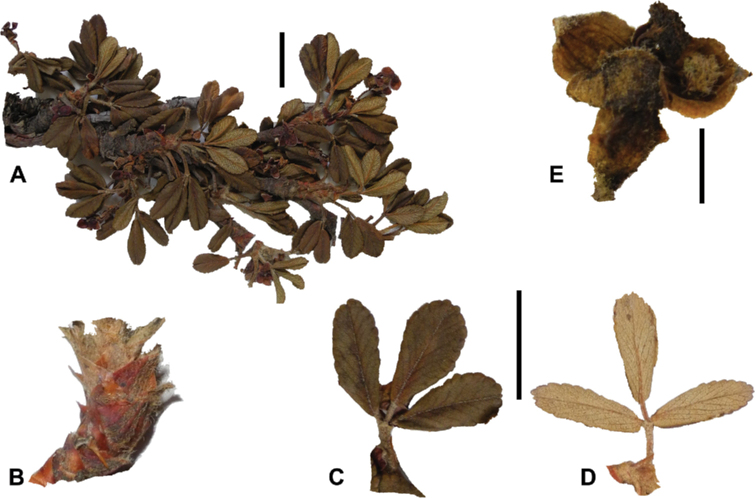
*Polylepisfjeldsaoi* T.Boza & M.Kessler **A** flowering branch **B** stipular sheaths **C** upper leaf surface **D** lower leaf surface **E** fruit (**A, C***La Torre 3234***B***Vargas 107***D, E***Weigend 97/678*). Scale bars: 2 cm (**A–D**); 3 mm (**E**). Photographs by T. E. Boza E.

#### Description.

***Trees*** to 2–10 m tall. ***Leaves*** slightly congested at the branch tips, imparipinnate with 2 pairs of leaflets, obtrullate in outline, 2.8–3.4 × 1.6–2.8 cm; rachises sparsely tomentose, points of leaflet attachment with a tuft of long hairs; stipular sheaths apically truncate, glabrescent on the outer surfaces; leaflets obovate in outline, second pair from the terminal leaflet the largest, one of this pair 1.2–2.1 × 0.6–0.7 cm; margin crenate with 5–10 teeth, apically obtuse to emarginate, basally unequally attenuate; upper leaflet surfaces glabrous to sparsely tomentose on mid-vein depression; lower leaflet surfaces with a dense layer of very short, white or yellowish pannose hairs. ***Inflorescences*** pendant, 3.4–6.8 cm long, bearing 3–5 flowers; floral bracts 3.9–6.2 mm long, narrowly triangular, sparsely villous on the outer surface; rachises sparsely villous. ***Flowers*** 6.8–7.0 mm diam.; sepals 3, ovate, green, glabrous or sparsely villous outside; stamens (9–)11–17, anthers orbicular, with a dense tuft of straight white hairs on the upper half; styles fimbriate, 2.4–3.3 mm long. ***Fruits*** turbinate, with 3–4 irregular flattened ridges with a series of spines, densely villous; 4.2–5.4 × 2.7–5.0 mm including spines. ***Diploid***.

#### Distribution, habitat and ecology.

*Polylepisfjeldsaoi* occurs in south-central Peru from southern Ayacucho to northern Caraveli (Arequipa) (Fig. 116). The species occurs in dry Andean habitats at 3300–4800 m elevation. It usually occurs in homogeneous stands.

#### Etymology.

We name the species in honor of Jon Fjeldså (1942–), professor and chief curator at the Zoological Museum of the University of Copenhagen (Denmark), in recognition of his pivotal contributions to the knowledge of the avifauna and biodiversity of *Polylepis* forests (Fjeldså 1987, 2002a, 2002b; Fjeldså and Krabbe 1990; Fjeldså and Kessler 1996; Fjeldså et al. 1999).

#### Conservation status.

The EOO for *Polylepisfjeldsaoi* is estimated as 51,627 km^2^, the AOO is assessed at 76 km^2^ and it is known from 17 locations. The species is largely unprotected, except for the Pampa Galeras National Park which includes a few small stands. We assess *P.fjeldsaoi* as Vunerable (B1a+B2a, C2a).

#### Notes.

The population of *Polylepis* from southern Ayacucho (Peru) has been previously identified as *P.tomentella* (Mendoza and Cano 2012). Simpson (1979) already reported some specimens from eastern Peru as possible hybrids between *P.tomentella* and *P.besseri* with crenate leaflets like *P.tomentella*, having the characteristic of densely pannose hairs of *P.besseri*. Indeed, *P.fjeldsaoi* resembles *P.tomentella* in having just one lateral leaflet pair, same leaflet shape and same type and density of lower leaflet hairs. However, it has broader leaflets (0.6–0.7 cm wide vs. 0.3–0.6 mm), crenate leaflet margins (vs. serrate), and fewer stamens ((9–)11–17 per flower vs. 19–23). Additionally, on present knowledge, *P.fjeldsaoi* is diploid, whereas *P.tomentella* is tetraploid. Furthermore, *P.fjeldsaoi* occurs in southern Peru, whereas *P.tomentella* is distributed from Bolivia to Argentina. Ecologically, *P.fjeldsaoi* grows under more humid conditions than *P.tomentella*, albeit with considerable overlap. *Polylepisfjeldsaoi* is also similar to *P.besseri*, with which it shares similar obovate leaflets with obtuse to emarginate apices and crenate margins. It differs from this species by its larger leaflets (1.2–2.1 × 0.6–0.7 cm vs. 1.4–1.6 × 0.6–1.1 cm), lower leaflet surfaces pannose (vs. pannose mixed with tomentose hairs 0.6–0.8 mm long) and inflorescences with fewer flowers (3–5 vs. 7–9).

#### Specimens examined.

**Peru. Apurimac**: Cotabambas, Tambobamba, Markarakay, 13°55'11"S, 072°10'41"W, 4120 m, 14 January 2020, *Paco s.n* (CUZ!). **Arequipa**: Caraveli, Cerca de Cahuacho, 15°27'32"S, 073°25'08"W, 4100–4300 m, 03 March 2002, *La Torre 3345* (USM!). **Ayacucho**: Huamanga, Hatumpampa-Vinchos, a la margen derecha del rio Vinchos, en el km 270–281 de la carretera Liberadores, 13°20'55"S, 074°27'28"W, 3100–3600 m, 29 September 2003, *Mendoza 991* (MO!). Leoncio Prado, Señal Cerro Palmaderes, near Pampa Galeras, between Nazca and Puquio, 14°40'S, 074°29'W, 3900 m, 11 March 1987, *Boertmann 103*; *104* (AAU!); Señal Cerro Palmaderas ca. 80 km above Nazca on road to Puquio, 14°40'S, 074°29'W, 3900 m, 11 March 1987, *Brandbyge 280* (AAU!); arriba del Puerto Toro Muerto km 77–78 Carretera Nazca Puquio, 14°41'23"S, 074°30'36"W, 3500–3520 m, 23 February 2002, *Cano 11877* (USM!); carretera a Minas Canarias aprox. km 15, 14°35'06"S, 074°25'08"W, 3800–3950 m, 23 February 2002, *Cano 11885* (USM!). Lucanas, Puquio, Queronta, 14°34'33"S, 074°04'57"W, 3879 m, 01 July 2015, *Boza 3037*; *3107*; *3108*; *3109*; *3110*; *3111*; *3112*; *3113*; *3114*; *3115*; *3116*; *3117* (USM!, Z!); near hacienda Pachan about 7 km W of Lucanas on Nazca-Puquio road, 14°36'S, 074°17'W, 3600 m, 12 March 1987, *Brandbyge 304* (AAU!); Puquio; 13 km de Puquio, 14°46'39"S, 074°01'04"W, 4075 m, 27 February 2002, *La Torre 3234* (USM!); Queronta, muy proximo a los baños termales de Bañochayoc, 14°35'S, 074°05'W, 3800–4000 m, 11 October 2003, *Mendoza 1017*; *1018*; *1019* (MO!); Reserva Nacional de Pampas Galeras. 563821/ 8374913, 3960 m, 25 April 2012, *Morales 4124* (USM!); Pampa Galeras, Chuquijara, a 4 km S. del Campamento, 4100 m, 08 April 1970, *Tovar 6755* (USM!); Pampa Galeras, 4000–4100 m, 02 December 1970, *Tovar 6801* (USM!); subiendo galeras, 01 July 2004, *Vargas 407* (USM!). Parinacochas, Incuyo, en las faldas del Volcán Sarasara, 15°20'S, 073°26'W, 3800–4000 m, 14 October 2003, *Mendoza 1029*; *1032*; *1039* (MO!); alrededor de la laguna de Ccaccapaqui, 12°00'S, 075°58'W, 3800–4000 m, 16 October 2003, *Mendoza 1050*; *1056*; *1057* (MO!). Puquio, Pampa Orccopa lakes E of Puquio Huachana SW of Yaurihuiri, 14°38'S, 073°57'W, 4300 m, 01 March 1987, *Boertmann 108* (AAU!); Pampa Orccopa Huachana SW of Yaurihuiri lakes E. of Puquio, 14°38'S, 073°57'W, 4300 m, 12 March 1987, *Boertmann 109* (AAU!); Laguna Yaurihuiri, about 205 km from Nazca on the road to Abancay, 14°38'S, 073°57'W, 4300 m, 13 March 1987, *Brandbyge 315* (AAU!), 1839–1840, *Gay s.n* (USM!).

### ﻿41. Polylepis incana

Taxon classificationPlantaeRosalesRosaceae

Kunth, Nov. Gen. Sp. (quarto ed.) 6: 227. 1824.

F3841197-5693-5848-9A56-F7F9D869953E

Figs 106 , 107

Polylepis incana subsp. villosistyla Bitter, Bot. Jahrb. Syst. 45: 642. 1911. Type. Ecuador. Chimborazo: Volcán El Altar, 3900 m, *Meyer 177* (holotype: B, destroyed).

#### Type.

Colombia. Los Pastos: Guachucal along the Río Blanco, 3150 m, Dec, *Bonpland 2191* (holotype: P!; isotypes: HAL!, P!; photo at F!).

**Figure 106.**
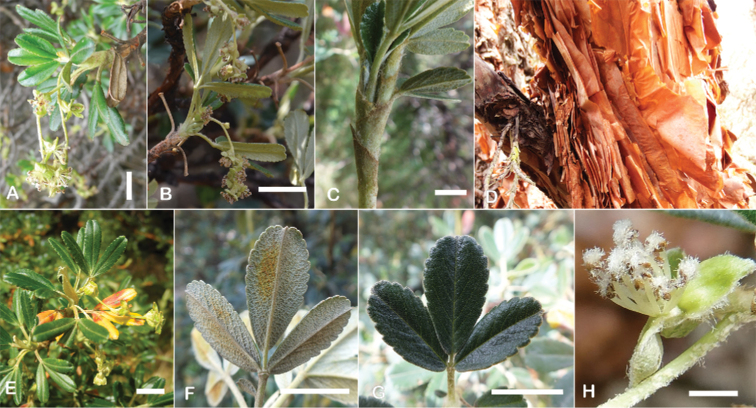
*Polylepisincana* Humboldt, Bonpland, and Kunth **A** flowering branch **B** flowering branch **C** stipule sheaths **D** bark **E** leaves **F** lower leaf surface **G** upper leaf surface **H** flower. Scale bars: 1 cm (**A, E–G**); 2 cm (**B**); 5 mm (**C**); 2 mm (**H**). Photographs **A, C–H** E.G. Urquiaga F **B** J. Chambi.

#### Description.

***Trees*** 2–15 m tall. ***Leaves*** slightly congested at the branch tips, imparipinnate with one pair of leaflets, obtrullate in outline, (1.9–)2.1–3.5(–4.4) × 1.8–2.9 cm; rachises glabrous to sparsely villous with resinous exudate, points of leaflet attachment with a tuft of long hairs; stipular sheaths apically acute, densely villous on the outer surfaces; leaflets elliptic to obovate in outline, second pair from the terminal leaflet the largest, one of this pair (1.4–)1.8–2.7 × 0.4–0.7 cm; margin crenate with 5–9 teeth, apically obtuse to emarginate, basally attenuate; upper leaflet surfaces glabrous; lower leaflet surfaces covered with very short, white pannose hairs and resinous exudate. ***Inflorescences*** pendant, (2.1–)2.7–7.1 cm long, bearing 5–11 flowers; floral bracts 3.8–4.9 mm long, narrowly triangular, densely villous on the outer surface; rachises villous. ***Flowers*** 5.4–8.8 mm diam.; sepals 4, ovate, green, densely villous outside; stamens 17–25, anthers orbicular, with a dense tuft of straight white hairs on the upper half; styles fimbriate, 2.1–3.0 mm long. ***Fruits*** turbinate, with 2–5 irregular flattened ridges with a series of spines, densely villous; 3.9–7.2 × 3.8–6.5 mm including spines. ***Diploid***, ***hexaploid*** in cultivated plants.

**Figure 107.**
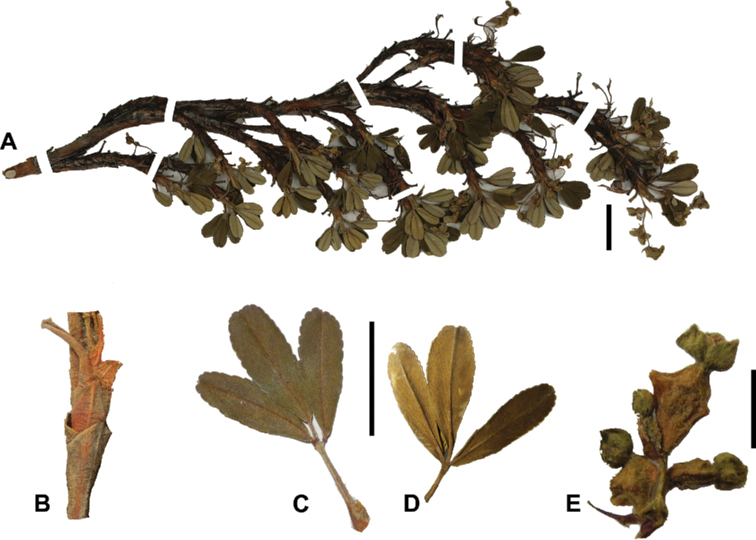
*Polylepisincana* Humboldt, Bonpland & Kunth **A** flowering branch **B** stipular sheaths **C** upper leaf surface **D** lower leaf surface **E** fruits (**A***Romoleroux 1191***B***Weigend 8811***C***Vargas 187***D***Weigend 5865***E***Weigend 7265*). Scale bars: 4 cm (**A**); 2 cm (**C, D**); 7 mm (**E**). Photographs by T. E. Boza E.

#### Distribution, habitat and ecology.

Natural populations of *P.incana* are known from southernmost Colombia to Ecuador and in central to southern Peru at 2150–4700 m elevation (Fig. 116). In Ecuador, *P.incana* often is mixed with low densities of *Gynoxysacostae* in unburned forests, but *G.acostae* has better survival after fire (Cierjacks et al. 2008). Based on an AFLP study, genetic diversity of seedlings in isolated forest patches is lower than in the adults, suggesting that current fragmentation leads to a loss of genetic diversity (Hensen et al. 2012). The density of seedlings and saplings of *P.incana* decreases notably with increasing elevation, so that vegetative reproduction becomes important at high elevations (Cierjacks et al. 2007b). Modelling the potential distribution of *P.incana* in Colombia shows that its climatic niche is not only present in the current distributional area of the Chiles and Cumbal volcanoes (Nariño), but also in the Cordillera Central where there are no reports of the species (Fajardo-Gutierrez et al. 2018). The species has been widely planted throughout and outside of its range, so that the naturalness of many populations may be questioned. Mature forests harbor more diverse small mammal communities than successional ones (Ojala-Barbour et al. 2019).

#### Conservation status.

The EOO for *Polylepisincana* is estimated as 1,445,546 km^2^, the AOO is assessed at 612 km^2^ and it is known from 101 locations. It is protected within El Cajas National Park, Illinizas and El Angel Ecological Reserves in Ecuador and Huascarán National Park and Cordillera Huayhuash Reserve Zone in Peru. The species was categorized as VU (A1a,c,d) in the World List of Threatened Trees (Oldfield et al. 1998). Later, it was classified as CR (SERFOR 2006). At many of its locations, the species grows in habitats that are strongly affected by human activities including grazing and burning. In Ecuador, survival of adult trees after a fire event was only 6.2% suggesting that single fire events strongly decrease adult and seedling populations (Cierjacks et al. 2008). Based on its wide distribution and occurrence in various protected areas, we assess *P.incana* as Least Concern (A1, B1a+B2a).

#### Notes.

As circumscribed here, *Polylepisincana* includes only those individuals with one lateral leaflet pair having the lower leaflet surfaces covered with very short, white pannose hairs and resinous exudate. *Polylepisincana* can be distinguished from the most similar species *P.tomentella* by it longer leaflets ((1.4–)1.8–2.7 cm vs. 1.3–2.1 cm), leaflet margins crenate versus serrate and numbers of flowers per inflorescence (5–11 versus 4–5). Individuals with 1–2 lateral leaflet pairs and tomentose hairs in the lower leaflet surface are referred to as *P.racemosa*. Simpson (1979) reported hybrids between these two species in central Peru, especially in the Pampa de Junín and from La Libertad. Romoleroux (1996) reported hybrids between *P.incana* and *P.ochreata* (as *P.sericea*), *P.pauta* and *P.reticulata*.

#### Specimens examined.

**Ecuador. Azuay**: Baños, P. N. de Las Cajas, along road Soldados-Angas aprox. 2 km above Soldados, 02°56'S, 079°12'W, 3300 m, 02 May 1992, *Lægaard 102678* (AAU!, GOET!, QCA!). Chaucha, P. N. LasCajas, Páramo de Soldados, road Cuenca-San Joaquín-Soldados, above Soldados, off road within the Park, km 51.7, 3680–3870 m, 08 January 2000, *Jørgensen 1711* (ILLS, MO!, QCNE); Páramo de Soldados, SW of Cuenca, 02°53'S, 079°18'W, 3000–3300 m, 03 March 1985, *Lægaard 53796* (AAU!, MO!); Páramo de Soldados, 02°53'S, 079°18'W, 3700–4000 m, 28–29 August 1985, *Lægaard 55098B* (AAU!). Checa (Jidcay), Chiquintad-Chanlud-Tuni, km 26.9, 02°43'S, 079°06'W, 3400–3500 m, 29 December 1990, *Jørgensen 92918* (AAU!). Coronel Lorenzo De Garaicoa (Pedregal), Area Nacional de Recreación Cajas, 40 km al w de Cuenca, 02°05'S, 079°20'W, 3450 m, 30 October 1986, *Neill 7384* (AAU!, MO!). Cuenca, Area Nacional de Recreación Cajas, collection made along Río Patul from the Comunidad Baute/Lagnua Patul (watershed of Río Patul), 02°33'S, 079°21'W, 3500–4200 m, 05 February 2001, *Clark 6228* (QCA!, QCNE, US!); Cuenca-Molleturo road. 45 k NW of Cuenca, 26 July 1982, *Clemants 2177* (AAU!, NY, QCA!); Cajas near headquarters on Cuenca-Guayaquil road, 3900 m, 12 March 1991, *Kessler 2745* (GOET!). Molleturo, Cuenca-Molleturo road ca. 5 km W of pass in Las Cajas, 02°49'S, 079°16'W, 3700 m, 01 May 1992, *Lægaard 102647* (AAU!, GOET!); Páramo de las Cajas. W of pass, 02°46'S, 079°15'W, 3500 m, 27 August 1985, *Lægaard 55043* (AAU!, MO!); *55046* (AAU!); Carretera Sayausí-Molleturo km 10–31, Parque Recreacional Las Cajas, 02°50'S, 079°15'W, 3750–3950 m, 28 November 1992, *Romoleroux 1485* (AAU!, QCA!). San Antonio, 3–5 km W of pass at Las Cajas, W of Cuenca, 02°40'S, 079°14'W, 3800–4000 m, 22 October 1984, *Lægaard 53197* (AAU!, MO!, QCA!); *53198* (AAU!, QCA!); *53203* (AAU!, MO!, QCA!). San Joaquín, San Joaquín-Soldados-Angas, km 33.1, 02°55'S, 079°15'W, 3470 m, 12 May 1990, *Jørgensen 92856* (AAU!, MO!); road Cuenca-Soldados, 02°57'S, 079°10'W, 3200–3300 m, 23 October 1984, *Lægaard 53232* (AAU!); Páramo de Soldados SW of Cuenca, 02°53'S, 079°17'W, 3700–3800 m, 24 October 1984, *Lægaard 53235A*; *53235C*; *53235E*; *53235G* (AAU!); at Soldados, 02°57'S, 079°14'W, 3200–3400 m, 28 August 1985, *Lægaard 55089* (AAU!, GOET!, MO!). Sayausi, Area Recreacional Las Cajas, 02°49'S, 079°07'W, 3740–4070 m, *Romoleroux 1191* (AAU!). 3200–4000 m, *Barclay 8932* (MO!, US!); 3700–4000 m, *Boeke 638* (QCA!); *Harling 24611* (GB, MO!, QCA!); 3140 m, 04 December 1990, *Jørgensen 92856* (AAU!); 3400–3500 m, 29 December 1990, *Jørgensen 92918* (AAU!, MO!); colección en ladera SO de la propiedad Dos Chorreras, 3995 m, 07 July 1995, *León 3595* (AAU!); 3750 m, *Molau 1477* (GB, QCA!); *Romoleroux 402* (NY, QCA!); 3900 m, *Steyermark 53038* (F!, NY); 3240 m, *Valencia 409* (QCA!). **Bolívar**: Guaranda, W of Crus de Los Arenales along road to Guaranda, 01°28'S, 078°57'W, 3500 m, 02 October 1985, *Lægaard 55372* (AAU!). Salinas, Km 5–10. Salinas-Guaranda, 01°27'S, 079°02'W, 2700 m, 01 October 1985, *Lægaard 55314* (AAU!, MO!). San Juan, along road Guaranda-Riobamba, km 27 at pass, 01°38'S, 078°51'W, 4150 m, 24 August 1985, *Lægaard 54997* (AAU!, MO!). **Carchi**: Chitan de Navarrete, Carretera antigua El Angel–Tulcán, desvío a La Esperanza, 00°40'N, 077°50'W, 3300 m, 03 September 1988, *Romoleroux 602* (AAU!, MO!, QCA!). Espejo, Reserva Ecológica El Angel, La Libertad-Morán, 00°45'N, 077°54'W, 3500 m, 30 October 1993, *Palacios 11615* (MO!, QCNE). La Libertad (Alizo), Hacienda La Esperanza, NE of El Angel, 00°39'N, 077°54'W, 3300 m, 08 October 1984, *Lægaard 53120* (AAU!, MO!, QCA!); Páramo de El Ángel-La Libertad, 00°42'07"N, 077°59'37"W, 3490 m, 05 October 2012, *Ulloa 2401* (MO!, QCA!). Tufiño, road Tulcan–Maldonado, near Volcán Chiles, 00°49'N, 077°56'W, 2000–3500 m, 16 August 1985, *Lægaard 54985* (AAU!); near Tufiño, 00°48'N, 077°51'W, 3050–3150 m, 17 August 1985, *Lægaard 54989* (AAU!, MO!). 3300–3800 m, *Acosta-Solís 10547* (F!); *Benoist 3623* (S); Páramo El Angel, colecciones entre El Angel, sector San jerónimo, 3500 m, 03 September 1988, *Jaramillo 10401* (AAU!, QCA!); just below Páramo El Angel, 18 February 1995, *Svenning 126* (AAU!). **Chimborazo**: Pablo Sexto, Collanes Valley, Páramo de los Altares, 01°40'S, 078°24'W, 3850 m, 03 September 1987, *Ramsay 391* (QCNE). Riobamba, Parroquia Quimiag. Sector San Miguel de Chancay, 01°39'57"S, 078°30'56"W, 3575 m, 07 December 2004, *Caranqui 1350* (CHEP), 3500 m, *Jaramillo 9539* (NY, QCA!). **Cotopaxi**: El Chaupi, road El Chaupi-Illiniza, 00°37'S, 078°40'W, 3400 m, 16 June 1984, *Lægaard 54541* (AAU!, QCA!); Illinizas, 3995–4103 m, 04 June 2011, *Ulloa 2046* (MO!, QCA!). Machachi, Parque Nacional Cotopaxi, Limpio Punga, 00°37'S, 078°27'W, 3830 m, 10 May 1984, *Lægaard 52103B* (AAU!, QCA!). Mulalo, Pansachi, 00°45'S, 078°30'W, 2700 m, 01 April 1983, *Brandbyge 42105* (AAU!, MO!, NY, QCA!); western part of Parque Nacional Cotopaxi, 00°41'S, 078°33'W, 3400 m, 16 June 1985, *Lægaard 54528* (AAU!, MO!, QCA!). San Juan de Pastocalle, Illiniza Sur, Corralpampa, 6–8 km de Pastocalle, 00°40'S, 078°40'W, 3300–3600 m, 12 February 1991, *Romoleroux 1226* (AAU!, MO!, QCA!); Illiniza Sur, Corralbamba, 6–8 km de Pastocalle, 00°40'S, 078°40'W, 3033–3600 m, 12 February 1991, *Romoleroux 1231* (AAU!, MO!, QCA!). 3830 m, *Argüello 453* (AAU!); 3400 m, *Asplund 6476* (S, US!). **Imbabura**: El Quinche, Laguna Grande de Mojanda, 15 km S of Otavalo, around laguna Grande and Laguna Negra, 00°08'S, 078°16'W, 3750 m, 13 May 1985, *Eriksen 59359* (AAU!). Gonzalez Suarez, Laguna Mojanda, at the southern part of Laguna Negra, 00°08'N, 078°15'W, 3700 m, 29 June 1983, *Brandbyge 42207* (AAU!, MO!, NY, QCA!). Otavalo, at Laguna Grande de Mojanda, 00°08'N, 078°17'W, 3850 m, 18–19 November 1985, *Lægaard 55661B* (AAU!); *Holmgren 913* (S). **Loja**: Manu, Río Negro, along side-road ca 8 km S of Manu-Saraguro road, from junction ca 8 km E of Manu, 03°34'S, 079°26'W, 3200 m, 13 September 1999, *Lægaard 20551* (AAU!, MO!). Saraguro, Manú, Río Negro, La Playa, 26 June 1994, *Vivar 4254* (AAU!). **Napo**: Papallacta, road Quito-Papallacta hot springs, 00°21'S, 078°10'W, 3500 m, 01 April 1998, *Clark 4991* (MO!); Pifo-Papallacta, 3–5 km E of Paso de La Virgen, 00°21'S, 078°11'W, 3700–3900 m, 09 June 1992, *Lægaard 103112* (AAU!); along río Pifo-Papallacta, E of Paso de la Virgen, 00°21'S, 078°11'W, 3750–3850 m, 21 June 1985, *Lægaard 54559E* (AAU!); Páramo de Guamaní alrededores de la laguna de Papallacta, 3900–4000 m, 06 December 1987, *Romoleroux 488* (AAU!). **Pichincha**: along Volcán Illiniza, NE slope below the refugio, 00°32'S, 078°41'W, 4000 m, 13 August 1980, *Holm-Nielsen 25019* (AAU!). Cayambe, Carretera Cayambe-Hda. Piamonte-Patapampa, 00°02'S, 078°04'W, 3700 m, 04 December 1993, *Freire 2603* (AAU!); Cangahua, vía Quito-Cayambe, 00°02'S, 078°15'W, 3500–3900 m, 8–12 February 1995, *Núñez 91* (MO!, QCNE). Checa (Chilpa), road Pifo-Papallacta, km 20, 00°10'S, 078°14'W, 3600 m, 30 September 1997, *Klitgaard 639* (AAU!). El Chaupi, Volcán Illiniza, NE slope below the refugio, 00°38'S, 078°42'W, 4300 m, 14 August 1980, *Holm-Nielsen 24968* (AAU!); Volcán Illiniza, N-side, 00°38'S, 078°41'W, 4200–4300 m, 20 June 1985, *Lægaard 54553* (AAU!, QCA!); *54556* (AAU!, QCA!); Loma Pilongo, NE slope of Nevado Illiniza, 00°38'S, 078°42'W, 3900–4200 m, 28 December 1987, *Molau 2241* (GB, MO!, QCA!). El Quinche, Laguna Grande de Mojanda, 00°08'S, 078°16'W, 3725–3750 m, 30 June 1988, *Romoleroux 662* (AAU!, QCA!). Machachi, 00°35'S, 078°21'W, 3480 m, 01 June 1985, *Nowak 162* (AAU!); camino Sangolquí a Limpiopungo cerca de dos y medio Km antes del cruce del río Pita para llegar a la estación de agua potable, 00°35'S, 078°21'W, 3480 m, 01 June 1985, *Nowak 163A* (AAU!); *162C* (AAU!). Mejía, Páramo, ca. 3 km NE of the volcano Illiniza Sur, 00°24'S, 078°42'W, 4000–4600 m, 19 March 1995, *Clark 479* (MO!, QCNE). Mulalo, Páramo at Hacienda Pauzacha south of Volcan Cotopaxi, 00°44'S, 078°29'W, 4000–4050 m, 28 November 1985, *Lægaard 55731* (AAU!, MO!). Pifo, about 4 km from La Virgin on the road from Pifo to Papallacta, 00°17'S, 078°12'W, 3750 m, 21 May 1984, *Brandbyge 42641* (AAU!, MO!); E of Papallacta pass, 00°18'S, 078°13'W, 3700–3800 m, 20 October 1984, *Jørgensen 56199* (AAU!, MO!, QCA!); Pifo-Pintag, in valley 2 ½ hours horseride above Inga Monserat, 00°19'S, 078°17'W, 3625–3725 m, 11 April 1992, *Lægaard 102261* (AAU!, GOET!, QCA!); Pifo-Pintag, in valley 2 ½ hours horseride above Inga Monserat, 00°19'S, 078°17'W, 3950 m, 12 April 1992, *Lægaard 102274* (AAU!, GOET!); Pifo-Pintag, in valley 2 ½ hours horseride above Inga Monserat, 00°19'S, 078°17'W, 3600–3625 m, 12 April 1992, *Lægaard 102282* (AAU!, GOET!, QCA!); Pifo (road to Ibarra)-Papallacta km 15 along new road, 00°16'S, 078°17'W, 2450 m, 16 April 1992, *Lægaard 102309* (AAU!); Pifo (road to Ibarra)-Papallacta km 18 along new road, 00°16'S, 078°17'W, 3700 m, 16 April 1992, *Lægaard 102319* (AAU!, QCA!); *102320* (AAU!, GOET!, QCA!); Páramo de Guamani, app. 5 km W of Paso de la Virgen, 00°19'S, 078°13'W, 3700–3800 m, 19–20 May 1984, *Lægaard 52189* (AAU!, QCA!); *52190* (AAU!, MO!, QCA!); road Pifo-Papallacta, km 10, 00°18'S, 078°14'W, 2900 m, 17 January 1985, *Lægaard 53487* (AAU!, MO!); Road Pifo-Papallacta, 3 km W of Paso de la Virgen, 00°18'S, 078°14'W, 3700–3900 m, 07 August 1985, *Lægaard 54878*; *54902A*; *54902B*; *54902C*; *54902D*; *54902E*; *54902F*; *54902G*; *54902H*; *54902I*; *54902L*; *54902N*; *54902Q*; *54902R*; *54902T* (AAU!); carretera Quito-Papallacta km 40–53, 00°16'S, 078°15'W, 3300–3500 m, 27 December 1992, *Romoleroux 1501* (AAU!, QCA!); carretera Pifo-Papallacta, sector Cuchaico, 00°15'S, 078°20'W, 3200 m, 11 September 1987, *Zak 3547* (AAU!, GB, MO!). Pintag, Cantón Mejía, Parroquia El Chaupi, faldas del Volcán El Corazón, 00°30'S, 078°25'W, 3300 m, 23 May 1988, *Zak 3685* (AAU!, MO!). Quito, Parroquia de Tumbaco, area de influencia de la Reserva Ecológica Antisana, 00°19'S, 078°16'W, 3700 m, 08 March 1994, *Alvarez 1344* (MO!, QCNE); *1389* (MO!, QCNE); Papallacta road, ca. 26.4 km E of Tumbaco, 00°13'N, 078°15'W, 3692 m, 07 November 1990, *Luteyn 14069* (AAU!, MO!); Pifo. Hacienda Los Andes, 00°13'S, 078°16'W, 3500 m, 24 January 1991, *Palacios 6907* (AAU!, MO!). Tocachi, at Laguna Grande de Mojanda, 00°08'N, 078°16'W, 3725–3750 m, 24–27 June 1984, *Lægaard 52345* (AAU!, QCA!); *52349* (AAU!, MO!); *52357* (AAU!); *52365* (AAU!, QCA!); Páramo de Mojanda, at Laguna Grande, 00°08'N, 078°16'W, 3700–3800 m, 10 November 1984, *Lægaard 53332*; *53333* (AAU!); *53334* (AAU!, MO!); *53336* (AAU!, MO!, QCA!); Páramo de Mojanda, at Laguna Negra and S-side of Laguna Grande, 00°08'N, 078°16'W, 3800 m, 14 May 1985, *Lægaard 54339* (AAU!, MO!); *54349* (AAU!, QCA!); Laguna Grande de Mojanda, 00°08'N, 078°16'W, 3725–3750 m, 30 June 1988, *Romoleroux 651* (MO!); *663* (AAU!, QCA!). 3500 m, *Acosta-Solís 8434* (F!); 3600–3800 m, *Asplund 8747* (MO!, QCA!); Napo-Pastaza; Cordillera Oriental; entre Pifo y el boquerón de Cerro de Corrales; Páramo de Guamaní, 3350 m, 15–16 August 1959, *Barclay 8932* (MO!); *Fagerlind s.n* (S); *Freire 23* (QCA!); ruta Tumboco Papallacta, 3600 m, 19 January 1979, *Halloy B-26* (AAU!); along the main road east of Quito towards Baeza, near Paso Guamaní, 3500 m, 17 April 1973, *Humbles 6303* (AAU!, GB, MO!, QCA!); 5 km W pass on Quito-Papallacta road, 3500 m, 06 April 1991, *Kessler 2756*; *2757*; *2758* (GOET!); *Kieft 229* (NY, QCA!); road Pifo-Papallacta, 3 km W of Paso de la Virgen, 00°18'S, 074°18'W, 3700–3900 m, 07 August 1985, *Lægaard 54902O* (AAU!); 3625–3950 m, *Luteyn 14457* (QCA!); 3480 m, *Nowak 162* (AAU!, QCA!); *162b* (QCA!); *163* (AAU!, QCA!); *Romoleroux 352*; *65* (QCA!); 3700 m, 12 January 1875, *Sodiro s.n.* (AAU!); Volcán Illiniza, northern slope, 4200 m, 19 April 1967, *Sparre 15625* (S); El Chaupi, along the road to Illiniza, 3300 m, 19 April 1967, *Sparre 15644* (AAU!, S). **Quito**: Pintag, road from Quito via Pifo to Papallacta, 00°29'S, 078°24'W, 3600 m, 04 July 2014, *Kessler 14600*; *14601* (Z!). Rumipamba, road from Quito via Pifo to Papallacta, 00°26'S, 078°25'W, 3450 m, 04 July 2014, *Kessler 14598*; *14599* (Z!). **Tungurahua**: San Fernando (Pasa San Fernando), near Calamaca, app. 20 km W of Ambato along old road Ambato-Guaranda, 01°16'S, 078°48'W, 3400–3900 m, 22 June 1985, *Lægaard 54563* (AAU!, QCA!); *Rose 22392* (NY); Tal Collanes, 3700 m, 09 August 1935, *Heinrichs 921* (GOET!); *Humboldt 2191* (MO!); 3700 m, *Sodiro s.n.* (QCA!); Illiniza, *Wagner 77* (GOET!); Vulkane Copac Urao und Condorarto, *Wagner s.n* (GOET!).

**Peru. Ancash**: Bolognesi, entre Ticllos y Llaclla, 3400 m, 26 May 1962, *Cerrate 4033* (USM!); Acas, 3700 m, 14 June 1979, *Cerrate 7483* (USM!); road from Abra Janashalla down to Huallanca, below Huansalá, 09°52'S, 076°59'W, 3390 m, 08 October 2007, *Weigend 8811* (USM!). Huaraz, Huascarán National Park, Quebrada Shallap, 09°30'S, 077°24'W, 3700–4000 m, 22 May 1985, *Smith 10713* (MO!, USM!). Huaylas, Huascarán National Park, Quebrada Santa Cruz between Hatun-quiswar and Lago Santa Cruz Chico, 08°55'S, 077°40'W, 4000–4100 m, 16 January 1985, *Smith 9270* (AAU!, F!, MO!, USM!). Independencia, Pitec, Cordillera Blanca, 09°30'S, 077°30'W, 3700 m, 11 August 1988, *Frimer 11* (AAU!). **Apurimac**: Andahuaylas, Pampachiri, Santa Rosa, 3700 m, 01 January 2004, *Vargas 187* (USM!). Aymaraes, Cotarusi, localidad de Sorak’asa rodal de titanka, 14°44'26"S, 073°33'43"W, 3944 m, 14 December 2006, *Huamantupa 8375* (CUZ!, F!, MO!). Cotaruse, Río Cotaruse, 7 km SW of Cotaruse, 14°27'S, 073°14'W, 3500 m, 14 March 1987, *Boertmann 115a*; *116a* (AAU!); Río Cotaruse, about 10 km above Cotaruse, 14°28'S, 073°14'W, 3550 m, 14 March 1987, *Brandbyge 351* (AAU!); Río Cotaruse. 7 km above Cotaruse, 14°27'S, 073°14'W, 3550 m, 14 March 1987, *Brandbyge 362* (AAU!). **Ayacucho**: Huamanga, Bosque de Ccenhuacucho, 3650 m, 01 November 2004, *Barrientos 10* (USM!); Bosque de Ccenhuacucho, 3700 m, 01 January 2005, *Barrientos 18* (USM!); Bosque de Ccenhuacucho, 3590 m, 01 November 2004, *Barrientos 6* (USM!); Vinchos, 13°21'15"S, 074°24'16"W, 3435 m, 28 June 2015, *Boza 3035*; *3092*; *3093*; *3094*; *3095* (USM!, Z!); Hatumpampa-Vinchos, a la margen derecha del Río Vinchos, en el km 270–281 de la carretera Liberadores, 13°20'55"S, 074°27'28"W, 3100–3600 m, 29 September 2003, *Mendoza 992* (MO!). Leoncio Prado, ca. 80 km from Nazca, 14°41'S, 074°30'W, 3600 m, 10 March 1987, *Brandbyge 270* (AAU!). Southern Peru aprox. 500 km S of Lima, near small lake Laguna de Parinacochas, 3800 m, 01 February 1986, *Brondal s.n* (AAU!). **Cusco**: Calca, rocky wayside on slopes east of Pisac, 3500–3600 m, 01 May 1925, *Pennell 13731* (A!, F!). Cusco, road to Pisaq, ca. 1 km after ruins of Sacsayhuaman, 13°30'07"S, 071°58'50"W, 3700 m, 10 September 2002, *Ackermann 261* (GOET!, USM!); Ccorca, 13°33'43"S, 072°01'41"W, 3990 m, 01 May 2003, *Arce s.n* (USM!); Huacoto, 13°30'46"S, 071°50'55"W, 3960 m, 01 May 2003, *Arce s.n* (CUZ!); K’enko ruins, above Cuzco, 3700 m, 20 August 1989, *Kessler 390* (GOET!); Plaza de Armas, 3450 m, 21 August 1989, *Kessler 392* (GOET!); Chucan S of Cusco, 13°29'S, 072°00'W, 3900 m, 10 February 2003, *Lægaard 22353* (AAU!); Ccorca, 13°33'42"S, 072°01'38"W, 3940 m, 29 May 2006, *Toivonen 102; 103* (CUZ!); alrededores de Cusco, 01 April 1936, *Vargas 340* (CUZ!); Callachaca, 3400 m, 26 December 1945, *Vargas 5502* (CUZ!, MO!). Paucartambo, Paucartambo Valley, Ccatcca, 3800 m, 01 August 1926, *Herrera 1129* (F!, US!); Huancarani, carretera a Paucartambo, 3500 m, 20 July 1963, *Vargas 14731* (CUZ!); 3 km east of Cusco; 3600 m, 01 October 1936, *West 8053* (MO!). **Huancavelica**: Huancavelica, Huancavelica, localidad de Ranracancha, 12°48'22"S, 075°03'06"W, 3850 m, 06 August 2017, *Quispe 73* (CUZ!, USM!, Z!); Cerro Santa Barbara, 3500–3600 m, 01 May 1958, *Tovar 3037* (US!, USM!). **Huánuco**: Ambo, 100 km S of Huanuco, 3360 m, 03 August 1978, *Aronson 601* (MO!). Chavinillo, Near Chavinillo, 48 km W of Huánuco towards La Union, 09°47'S, 076°35'W, 3800 m, 13 February 1987, *Boertmann 43*. Dos de Mayo, cerca de la mina de Huallanca, 3880 m, 20 March 1983, *Tovar 9797* (USM!); Huallanca, alrededores, 3700 m, 23 March 1983, *Tovar 9877* (USM!). Huallanca, Huansala, 10 km from Huallanca, 09°51'S, 076°56'W, 3700 m, 14 February 1987, *Boertmann 47* (AAU!). Huánuco, Lauricocha, San Miguel de Cauri, Laguna Lauricocha, lado sur oeste a pocos metros de la laguna, 3845 m, 08 August 2002, *Salvador 424* (USM!); Km 321 on Route 3 (north of Quinua), 11 January 1977, *Simpson 8554d* (A!, MO!, US!). Jacas Grande, 3 km south of Quivilla on road between Huánuco and La Union, 09°32'S, 076°41'W, 3900 m, 13 February 1987, *Boertmann 44* (AAU!); on road from Huánuco to Cerro de Pasco at ca. km 370, 3621 m, 04 August 1977, *Duncan 2700* (AAU!); along road from Huanuco to Cerro de Pasco at km 324, 3962 m, 05 August 1977, *Duncan 2701* (MO!, USM!); Llata, 3133 m, 21 August 1922, *Macbride 2249* (A!, US!); San Miguel de Cauri, Lauricocha, Jesus Río Lauricocha, 3326 m, 08 August 2003, *Salvador 541b* (USM!); Chiklin, 28 October 1927, *Sawada P83* (F!). **Junín**: Concepcion, road from Huancayo to San Vicente de Cañete, in San Jose de Quero, 12°05'23"S, 075°32'09"W, 3974 m, 22 September 2001, *Weigend 5865* (GOET!, USM!). **La Libertad**: Bolivar, Uchumarca, Shalca Pata, Uchumarca, 07°00'10"S, 077°47'29"W, 3360 m, 12 September 2010, *Monigatti 283* (MO!). Sanchez Carrion, La Arena, 11 km lineales al SO de Huamachuco, 818432, 9126738, 3060 m, 21 March 2006, *Roque 5042* (USM!); Santo Domingo y alrededores, 15 km lineales al SO de Huamachuco, 07°52'16"S, 078°08'36"W, 3150–3270 m, 05 July 2006, *Roque 5352* (USM!). Santiago de Chuco, Santuario Nacional de Calipuy, 800385 9078342 UTM, 3600 m, 12 December 2011, *Beltrán 7404* (USM!); 3100–3250 m, 08 June 1953, *López 983* (US!). **Lambayeque**: Ferrenafe, road from Ullupampa (Syn Uyurpampa) towards Cañaris (syn Kañaris) upper reaches of eastern flank of Cerro Negro/Cerro Tembladera, 06°10'02"S, 079°22'10"W, 3698 m, 02 May 2006, *Weigend 8566* (F!, USM!). **Lima**: Canta, Quebrada Minaquicche, en el distrito de Huaros, 10 October 1989, *Arce 154* (MO!); *155* (MO!); Cullhuay, alrededores de la población, 3500 m, 25 February 1992, *Vilcapoma 1590* (USM!). Huanuco, road from Junín to Huanuco, 9 km S of Chirtin near border with Pasco, 3660 m, 12 April 1977, *Gentry 19224* (MO!). Huarochiri, Quebrada Cayula, 3100 m, 21 November 2006, *Zapata s.n* (USM!); Parte baja del cerro Potrero Grande, Quichas, 3900 m, *Arce 191* (MO!); Chilcorral, Quichas, 3850 m, 13 January 1990, *Arce 193* (MO!); Pueblo Quichas above Oyon, 10°36'S, 076°45'W, 4000 m, 07 February 1987, *Boertmann 77*; *78*; *79* (AAU!); Oyon Laguna, 10°34'09"S, 076°45'29"W, 4048 m, 24 May 2015, *Boza 3027*; *3058*; *3059*; *3060*; *3061*; *3062*; *3063* (USM!, Z!); Near Oyón, Río Huaura, 3800 m, 29 November 1964, *Koepcke 1861bd* (US!); *186bd* (US!); carretera a Oyón, ladera del Río Quichas, a 1 km de Quichas, 3900–4000 m, 14 May 2004, *Salvador 993* (USM!). Yauyos, road from Yauyos to Jauja, 12°16'35"S, 075°42'14"W, 3700 m, 07 October 2002, *Weigend 7265* (GOET!). **Pasco**: Huariaca, 95 km S from Huanuco, on road to Cerro de Pasco, 10°25'S, 076°10'W, 3590 m, 15 July 1982, *Gentry 37484* (MO!). Pasco, between Cerro de Pasco and La Quinua, 5 km along highway above La Quinua, 3600 m, 21 June 1940, *Asplund 11847* (US!); Yarusyacán, La Quinua, 10°36'34"S, 076°10'47"W, 3600 m, 26 May 2015, *Boza 3028*; *3064*; *3065*; *3066*; *3067*; *3068*; *3069* (USM!, Z!); Pariamarca, 3720 m, 05 November 1985, *ONERN 106* (USM!); Pariamarca, 3720 m, 30 October 1985, *ONERN 107* (USM!); Pariamarca, 3720 m, 05 November 1985, *ONERN 108* (US!, USM!); La Quinua, 3540 m, 01 December 1986, *Rivas s.n* (USM!); Cerro de Pasco–Huanuco km 16, 10°39'12"S, 076°10'06"W, 4000 m, 22 August 2002, *Schmidt-Lebuhn 521* (GOET!, USM!); below Cerro de Pasco road to Ambo, 24 November 1945, *Shibert 2202* (US!); North of Quinua on route 3, 11 January 1977, *Simpson 8554a* (USM!); North of Quinua on Route 3, 11 January 1977, *Simpson 8554b*; *8554C* (USM!). Yanacancha, upper Río Huallaga at Pariamarca, 10°40'S, 076°10'W, 3800 m, 07 February 1987, *Boertmann 23*; *24*; *32*; *33* (AAU!). Km 317, ca. 20 km NE of Cerro de Pasco on route 3. W side of upper Río Huallaga, Huallaga Canyon, 12400 ft, 06 November 1975, *Davidson 3373* (MO!, US!); Cerro de Pasco, below Cerro de Pasco road to Ambo, 24 November 1945, *Seibert 2202* (MO!).

### ﻿42. Polylepis incanoides

Taxon classificationPlantaeRosalesRosaceae

(M.Kessler) T.Boza & M.Kessler, comb. et stat. nov.

0F9010CF-B98E-5C45-BB9F-D3F3AFDF68CD

urn:lsid:ipni.org:names:77301652-1

Figs 108 , 109

Polylepis tomentella subsp. incanoides M.Kessler, Candollea 50(1): 164. 1995. Type. based on Polylepisincanoides (M.Kessler) T.Boza & M.Kessler.

#### Type.

Bolivia. Dept. Cochabamba, Prov. Carrasco, surroundings of Monte Punco, l7°36'S, 65°17'W, 2800 m, 14 Aug1991, *Kessler 2954* (holotype: LPB!; isotypes: AUU!, GOET!).

**Figure 108.**
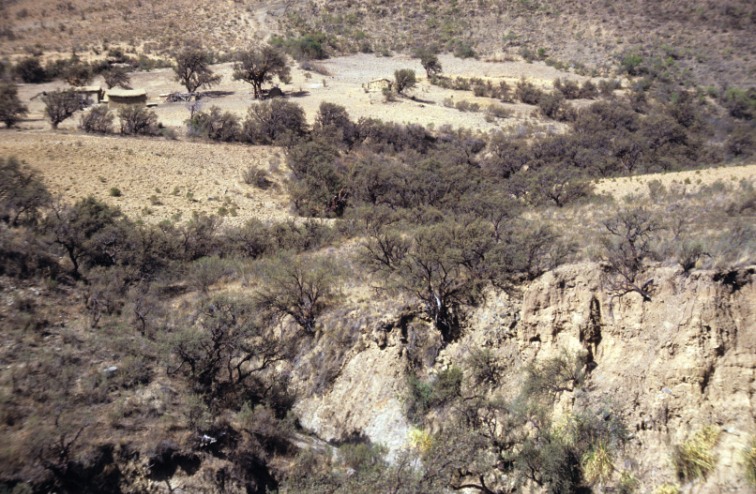
*Polylepisincanoides* (M.Kessler) T.Boza & M.Kessler. Habit. Photograph M. Kessler.

#### Description.

***Trees*** to 2–5 m tall. ***Leaves*** slightly congested at the branch tips, imparipinnate with one pair of leaflets, trullate in outline, 2.3–4.1 × 2.0–3.7 cm; rachises sparsely villous, points of leaflet attachment with a tuft of long hairs; stipular sheaths apically truncate, glabrescent to sparsely villous on the outer surfaces; leaflets obovate in outline, second pair from the terminal leaflet the largest, one of this pair 1.3–2.0 × 0.5–0.7 cm; margin serrate with 7–15 teeth, apically obtuse to slightly acute, basally unequally attenuate; upper leaflet surfaces glabrous to sparsely villous on mid-vein depression; lower leaflet surfaces with a dense layer of very short, white or yellowish pannose hairs. ***Inflorescences*** pendant, 3.2–3.7 cm long, bearing 5–7 flowers; floral bracts 2.6–4.6 mm long, narrowly triangular, densely villous on the outer surface; rachises villous. ***Flowers*** 5.5–7.1 mm diam.; sepals 3, ovate, green, glabrous or sparsely villous outside; stamens 15–19, anthers orbicular, with a dense tuft of straight white hairs on the upper half; styles fimbriate, 1.8–2.1(–2.7) mm long. ***Fruits*** turbinate, with 3–4 irregular flattened ridges with a series of spines, densely villous; 3.9–4.8 × 3.1–4.5 mm including spines. ***Tetraploid***.

**Figure 109.**
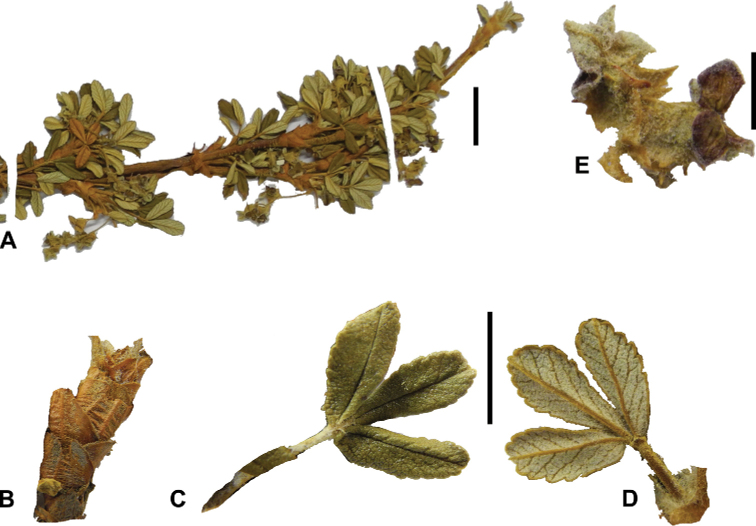
*Polylepisincanoides* (M.Kessler) T.Boza & M.Kessler **A** flowering branch **B** stipular sheaths **C** upper leaf surface **D** lower leaf surface **E** fruits (**A, C–E***Peterson 12723***B***Kessler 2953*). Scale bars: 8 cm (**A**); 2 cm (**C, D**); 4 mm (**D, E**). Photographs by T. E. Boza E.

#### Distribution, habitat and ecology.

*Polylepisincanoides* occurs in small populations in Cochabamba (Bolivia). It grows mainly in relatively dry areas at 2650–3750 m elevation (Fig. 116). It occurs as homogeneous stands or in mixed stands with *Podocarpusparlatorei*, *Alnusacuminata*, *Clethra* sp., *Weinmannia* sp., *Schinopsishaenkeana* and *Aspidospermaquebracho-blanco* at its distributional limits (Kessler 1995b). Kessler (1995b) reported hybrids between *P.incanoides* and both *P.besseri* and *P.subtusalbida* at their narrow sympatry zones.

#### Conservation status.

The EOO for *Polylepisincanoides* is estimated as 5,649 km^2^, the AOO is assessed at 64 km^2^, and it is known from 11 locations. No conservation action has been taken to date. The species was categorized as VU (B1+2c, D2) in the World List of Threatened Trees (Oldfield et al. 1998). However, based on its restricted distribution in Cochabamba, *P.incanoides* was categoriszed as EN (B2ab(i,ii,iii)) in Bolivia (Arrázola and Coronado 2012). The best-preserved remnant forests are found in the north-western mountains of Epizana (Navarro et al. 2010). Based on its fragmented distribution and degraded habitat, we assess *P.incanoides* as Endangered (A1+A2a, B1a+B2a, D1).

#### Notes.

*Polylepisincanoides* is very similar to *P.tomentella* and, in fact, it was treated as a subspecies of *P.tomentella* by Kessler (1995b). We here elevate *P.incanoides* to species rank based on morphological, ecological and biogeographical grounds. *Polylepisincanoides* differs from *P.tomentella* by leaflet apex (obtuse to slightly acute versus round to emarginate), inflorescence length (3.2–3.7 cm versus 2.8–5.3 cm), number of flowers (5–7 versus 4–5) and stamen number (15–19 versus 19–23). In addition, within its geographically restricted and isolated range, *P.incanoides* grows under significantly higher temperatures and levels of precipitation than *P.tomentella*.

#### Specimens examined.

**Bolivia. Cochabamba**: Arani, al borde de la carretera Cochabamba-Santa Cruz, 3400 m, 04 November 1988, *Hensen 222* (GOET!, LPB); Mojon, 1 km N Cochabamba-Sta Cruz road, 17°29'S, 065°25'W, 3000 m, 14 August 1991, *Kessler 2943* (GOET!, LPB); 6 km E Mojon on Cochabamba-Comarapa road, 17°30'S, 065°23'W, 2850 m, 05 October 1991, *Kessler 3287, 3288* (GOET!, LPB); *3289* (GOET!, MO!). Arque, proximidades a la comunidad de Kutimarca y Sumuruni, camino hacia Arque, 3850 m, 11 April 1999, *Mercado 2169* (MO!). Carrasco, Cochabamba 142 km hacia Santa Cruz, 16°58'25"S, 065°20'49"W, 3050 m, 24 March 1981, *Beck 6827* (GOET!, LPB, MO!); Cochabamba 161 km hacia Santa Cruz, 3100 m, 27 September 1981, *Beck 7047* (GOET!, LPB, MO!); Epizana, 3000 m, 01 November 1954, *Cardenas 5221* (US!); 2 km despues de Totora, 2900 m, 20 February 1979, *Ceballos 407* (G); Carretera Fundamental 4.7 km E Epizana, 2956 m, 05 December 1975, *Davidson 3760* (F!, NY); Montepunco, Canyon of Río Huairamayu (R. Montepunco) 40 km N of Totora, 2800 m, 22 August 1947, *Fosberg 28456* (NY); Llutupampa, 3300 m, 14 April 1991, *Hensen 2416* (LPB); *2420* (GOET!); Llutupampa, 3100 m, 14 April 1991, *Hensen 2450*; *2463*; *2473* (LPB); Surroundings of Monte Punco, 17°36'S, 065°17'W, 2800 m, 14 August 1991, *Kessler 2953* (GOET!); *2954*; *2955* (AAU!, GOET!); 3 km E Epizana on Cochabamba-Comarapa road, 17°40'S, 065°05'W, 3000 m, 05 October 1991, *Kessler 3290* (GOET!); 8 km E Epizana on Cochabamba-Comarapa road, 17°41'S, 065°04'W, 3000 m, 05 October 1991, *Kessler 3292* (GOET!); *3293* (AAU!, GOET!); 81 miles our of Cochabamba on road to Santa Cruz, 9400 ft, 13 November 1959, *Maguire 44482* (GH!, NY, US!); Km. 135 hacia Totora, proximidades del lugar llamado Cañada hornillas, 17°44'07"S, 065°11'28"W, 3000 m, 21 March 1999, *Mercado 1970* (MO!); 8.7 km al este de Epizana por el camino entre Cochabamba y Santa Cruz, 17°40'S, 065°09'W, 3200 m, 04 February 1987, *Solomon 15922* (MO!, NY); Subiendo el Río Mizque hacia Totora pasando Totora, 17°45'S, 065°12'W, 20 October 1995, *Torrico 713* (LPB). Mizque, Khuchu 23 km E Vacas, 3500 m, 03 March 1991, *Hensen 1924* (LPB). Totora, Totora and Duraznillo, 17°44'00"S, 065°11'00"W, 2500 m, 20 December 1921, *Steinbach 6031* (A!, G). a unos 97 km de la capital en dirección a Santa Cruz, matorral alto, 3565 m, 25 December 1982, *Fernández 3565* (MO!). **Potosí**: Charcas, de Acasio 23 km hacia Uncia, 1 km antes del desvío a San Pedro, 18°06'36"S, 066°06'26"W, 3450 m, 02 February 2015, *Beck 34512* (LPB); entre Caracollo y Cochabamba, Pongo, Kulku Mayu, 3800 m, 24 December 1982, *Fernández 7699* (MO!).

### ﻿43. Polylepis nana

Taxon classificationPlantaeRosalesRosaceae

(M.Kessler) T.Boza & M.Kessler, comb. et stat. nov.

13959745-400C-5725-A80E-25CF501A9BD0

urn:lsid:ipni.org:names:77301653-1

Figs 110 , 111

Polylepis tomentella subsp. nana M.Kessler (1995b: 166). Type. Based on Polylepisnana (M.Kessler) T.Boza & M.Kessler.

#### Type.

Bolivia. Dept. Cochabamba, Prov. Arani, 2 km W turn-off to Vacas from Arani-Mizque road, l7°33'S, 65°42'W, 3200 m, 18 Aug1991, *Kessler 3409* (holotype: LPB!; isotypes: AUU!, GOET!).

**Figure 110.**
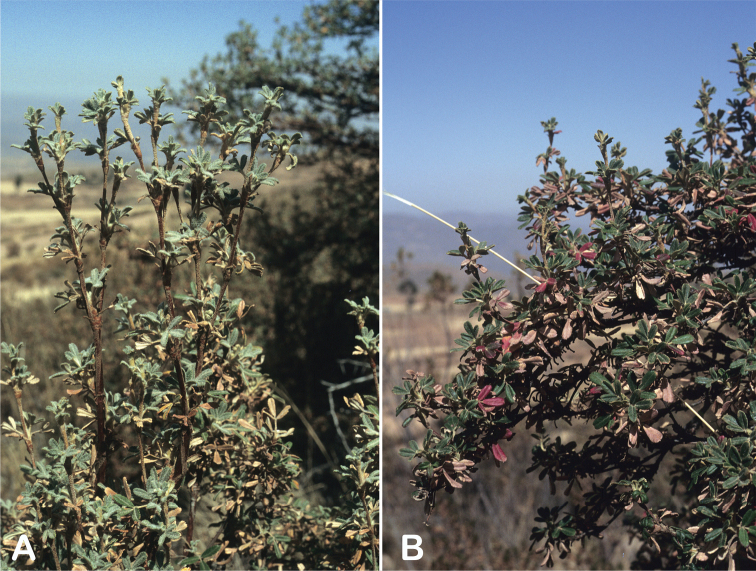
*Polylepisnana* (M.Kessler) T.Boza & M.Kessler **A** branching pattern **B** branching pattern. Photographs by M. Kessler.

#### Description.

***Shrub*** to 2 m tall. ***Leaves*** slightly congested at the branch tips, imparipinnate with one pair of leaflets, trullate in outline, 1.7–2.4 × 1.2–1.9 cm; rachises densely villous, points of leaflet attachment with a tuft of long hairs; stipular sheaths apically truncate or slightly spurred, densely villous on the outer surfaces; leaflets obovate in outline, second pair from the terminal leaflet the largest, one of this pair 1.0–1.2 × 0.3–0.5 cm; margin serrate with 5–8 teeth, apically acute, basally unequally attenuate; upper leaflet surfaces often with dark sheen, glabrous to sparsely villous; lower leaflet surfaces with a dense layer of very short, white or yellowish pannose hairs. ***Inflorescences*** pendant, 0.9–1.2 cm long, bearing 2–4 flowers; floral bracts 2.4–3.1 mm long, narrowly triangular, densely villous on the outer surface; rachises villous. ***Flowers*** 5.4–6.8 mm diam.; sepals 3, ovate, green, sparsely villous outside; stamens 12–15, anthers orbicular, with a dense tuft of straight white hairs on the upper half; styles fimbriate, 3.0–3.3 mm long. ***Fruits*** turbinate, with 3–4 irregular flattened ridges with a series of spines, densely villous; 5.0–7.7 × 3.0–3.3 mm including spines. ***Tetraploid***.

**Figure 111.**
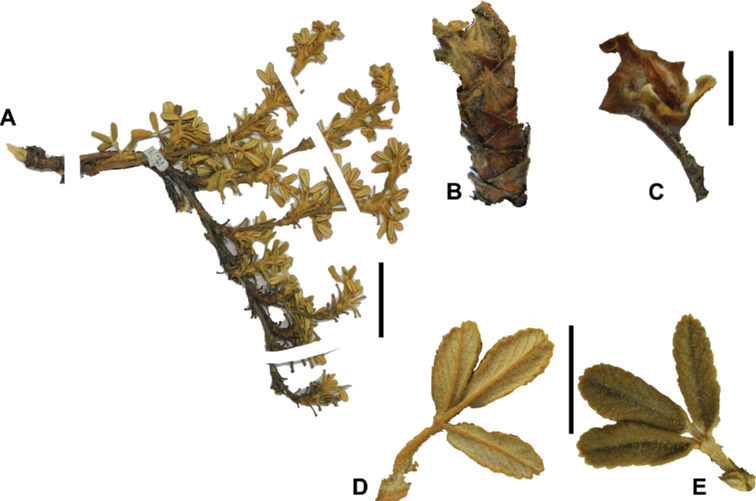
*Polylepisnana* (M.Kessler) T.Boza & M.Kessler **A** flowering branch **B** stipular sheaths **C** fruit **D** lower leaf surface **E** upper leaf surface (**A***Kessler 3512***B***Kessler 3495***C***Kessler 3513***D***Kessler 3642***E***Kessler 3520*). Scale bars: 8 cm (**A**); 6 mm (**C**); 1 cm (**D, E**). Photographs by T. E. Boza E.

#### Distribution, habitat and ecology.

*Polylepisnana* is restricted to Cochabamba (Bolivia) where it has been collected at just one locality in Arani Province (Fig. 116). It grows at 3200–3450 m elevation.

#### Conservation status.

The EOO for *Polylepisnana* is estimated as 8 km^2^, the AOO is assessed at 8 km^2^ and it is known from only one location. No conservation action has been taken to date. *Polylepisnana* was categorized as CR (B1+2c, C2b) in the World List of Threatened Trees (Oldfield et al. 1998). However, it was classified as EN (B1ab(i,ii,iii)) in the Red List of Threatened Flora of Bolivia (Arrázola et al. 2012). The only known locality consists of extensive, but heavily degraded stands in the Arani-Vacas area where they are strongly threatened by overgrazing, logging and burning of grasslands. We assess *P.nana* as Critically Endangered (A1+A2a, B1a, C1+ C2a, D2a).

#### Notes.

*Polylepisnana* can be distinguished from the most similar species *P.tomentella* by its shrubby growth form, smaller leaflets (1.0–1.2 × 0.3–0.5 cm vs. 1.3–2.0 × 0.5–0.7 cm) with acute apex (vs. round to slightly emarginated), shorter inflorescences (0.9–1.2 cm vs. 2.8–5.3 cm) with fewer flowers (2–4 vs. 4–5), fewer stamens per flower (12–15 vs. 19–25) and longer styles (3.0–3.3 mm vs. 2.4–2.5 mm).

#### Specimens examined.

**Bolivia. Cochabamba**: Arani, between Arani and Cañadas on the road to Vacas, 17°34'S, 065°40'W, 3100 m, 20 April 1987, *Brandbyge 708* (AAU!); Kewiñal, 3300 m, 24 March 1991, *Hensen 1949* (LPB); *2012* (LPB); 2 km after turnoff to Vacas on Mizque-Arani road, 17°33'S, 065°42'W, 3100 m, 18 August 1991, *Kessler 2998* (GOET!, LPB); 2 km W turnoff to Vacas from Arani-Mizque road, 17°33'S, 065°42'W, 3200 m, 18 August 1991, *Kessler 3403*; *3404* (GOET!, LPB); *3405* (GOET!); *3406*; *3407* (GOET!, LPB); *3408* (GOET!, LPB, USM!); *3409* (AAU!, GOET!); *3495*; *3501*; *3514*; *3518*; *3519*; *3521*; *3641* (GOET!); *3642* (GOET!); camino a Vacas, 3170 m, 11 January 1990, *Saravia 17* (LPB).

### ﻿44. Polylepis tarapacana

Taxon classificationPlantaeRosalesRosaceae

Phil. Anales Mus. Nac. Santiago de Chile. Segunda Sección–Botánica 8: 21. 1891.

119AD464-BBF1-5B72-937C-E584B18FBC40

Figs 112 , 113

Polylepis tarapacana var. multisquamata Bitter, Bot. Jahrb. Syst. 45: 654. 1911. Type. Chile. Tarapacá, 3900 m, *Philippi s.n* (holotype: B, destroyed). Probably an illegitimate name since it was based on a Phillippi specimen that was most likely part of the type collection of P.tarapacana.
Polylepis tarapacana var. sajamensis Bitter, Bot. Jahrb. Syst. 45: 654. 1911. Type. Bolivia. Oruro, Sajama, 4500 m, *Stübel 1* (holotype: B, destroyed).
Polylepis tarapacana var. brevifilamentosa Bitter, Bot. Jahrb. Syst. 45: 654. 1911. Type. Bolivia. Oruro, Tacna Perú, *Stübel 112* (holotype: B, destroyed).
Polylepis tarapacana var. pycnolopha Bitter, Bot. Jahrb. Syst. 45: 654. 1911. Type. Bolivia. Between La Paz and Tacna, 12 300–13 400 ft, 1838, *Pentland s.n* (holotype: P!).

#### Type.

Chile. Tarapacá, near Caña, 3900 m, *Philippi s.n* (lectotype, designated by Simpson 1979, pg. 46: SGO!; isolectotypes: GH, SGO!).

**Figure 112.**
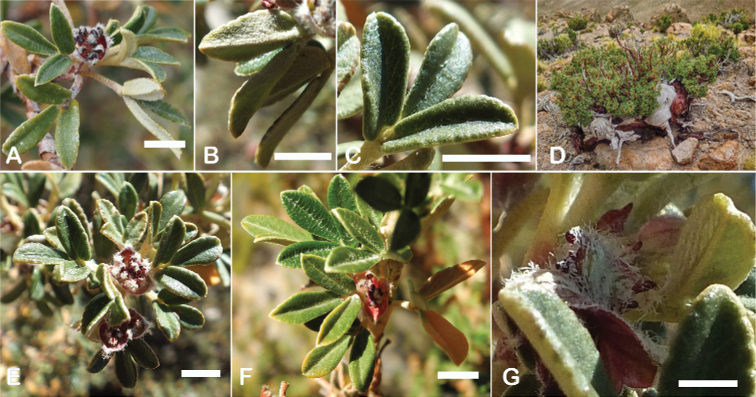
*Polylepistarapacana* Phil **A** flower **B** lower leaflet surface **C** upper leaflet surface **D** habit **E** flowering branch **F** flower **G** fruit (**B, C, E–G***Boza & Urquiaga 3009*). Scale bars: 5 mm (**A–C, E–F**); 3 mm (**G**). Photographs **A** A. Domic **B, C, E, G** E.G. Urquiaga F. **F** T.E. Boza E. **D** M. Cellini.

#### Description.

***Trees*** 1–5 m tall. ***Leaves*** slightly congested at the branch tips, imparipinnate with one pair of leaflets, obtrullate in outline, 1.3–1.7 × 0.9–1.2 cm; rachises densely pannose, points of leaflet attachment with a tuft of long hairs; stipular sheaths apically truncate, densely villous on the outer surfaces; leaflets obovate in outline, second pair from the terminal leaflet the largest, one of this pair 0.7–0.8 × 0.3–0.4 cm; margin entire or very slightly crenate with 3–4 teeth, apically obtuse or acute, basally unequally attenuate; upper leaflet surfaces rugose, glabrous, usually covered with a layer of yellowish resinous exudate; lower leaflet surfaces with a dense layer of very short, yellowish pannose hairs. ***Inflorescences*** pendant, 0.7–1.5 cm long, bearing 1–2 flowers; floral bracts 3.0–3.5 mm long, narrowly triangular, densely villous on the outer surface; rachises villous. ***Flowers*** 5.1–8.0 mm diam.; sepals 4, ovate, green, densely villous outside; stamens 9–13, anthers orbicular, with a dense tuft of straight white hairs on the upper half; styles fimbriate, 2.2–2.9 mm long. ***Fruits*** turbinate, with 3–4 irregular flattened ridges with a series of spines, densely villous; 4.1–5.2 × 3.1–7.2 mm including spines. *Tetraploid*.

**Figure 113.**
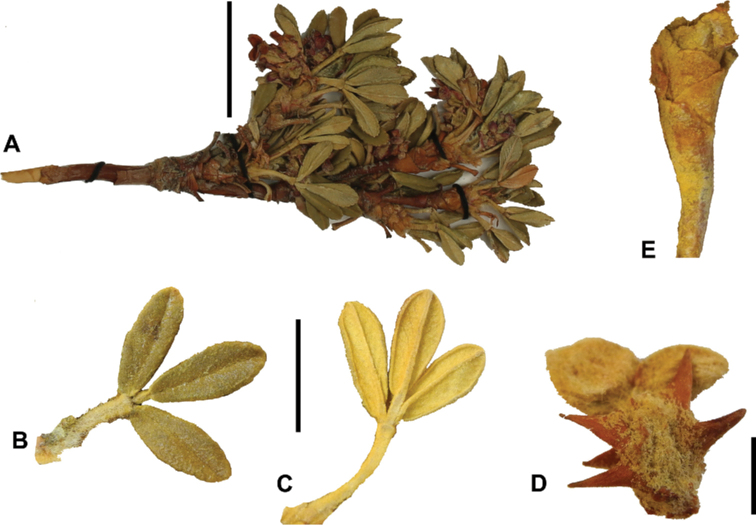
*Polylepistarapacana* Phil **A** flowering branch **B** stipular sheaths **C** upper leaf surface **D** lower leaf surface **E** fruit (**A, E***Peterson12947***B***Kessler 3595***C***Kessler 3604***D***Kessler 3597*). Scale bars: 3 cm (**A**); 1 cm (**C, D**); 2 mm (**E**). Photographs by T. E. Boza E.

#### Distribution, habitat and ecology.

*Polylepistarapacana* is distributed in the volcanic western Andean cordillera from southern Peru to south-western Bolivia, northern Chile and adjacent northwest Argentina at 3400–5013 m elevation (Fig. 116). It has long been considered to be the highest-growing tree species in the Western Hemisphere, with records up to 5200 m by Jordan (1980). However, such high records have not been confirmed and the true upper elevational limit of the species appears to be at around 5000 m. Regeneration of this species is favoured by thermically sheltered and moist microhabitats (Lien et al. 2021). In Oruro (Bolivia), maximum tree height, annual shoot increment and mean tree-ring width decreased with elevation, reaching only 3.3 m of maximum height and 34 cm maximum diameter at 4820 m, where also the highest density of seedlings was found (Hoch and Körner 2005). At higher elevations, there are only scattered individuals of shrubby growth form. Due to the highly seasonal climatic conditions in its distributional range, *P.tarapacana* forms clearly distinct annual rings, although it is common to find discontinuous rings and reaction tissue (Domic 2005). Tree ring chronologies of up to 705 years have been reconstructed for the Bolivian Altiplano (Argollo et al. 2006) and of up to 536 years in Chile (Moya and Lara 2011). The growth of *P.tarapacana* is positively related with precipitation in the year prior to the year of growth ring formation (Argollo et al. 2004; Domic 2005; Argollo et al. 2006; Domic and Capriles 2009; Moya and Lara 2011). The species has greater stomatal control when aridity increases and has a decoupling of physiological processes at leaf level versus wood formation depending on their sensitivity to climate (Rodriguez-Caton et al. 2021). The species is sensitive to the soil moisture content (and rainfall), distributing the biomass in small diameters and heights, with preferably multi-stemmed individuals, in order to retain the available moisture (Saavedra 2013). *Polylepistarapacana* trees have various diffusive and metabolic compensatory adjustments that enable high assimilation rates sustained by a photosynthetic apparatus exceptionally well adapted to the effect of low temperatures and drought (Jaramillo 2015). Extensive gene flow has been found to occur within and between Chilean populations of *P.tarapacana* (Schmidt-Lebuhn et al. 2006b; Moya and Lara 2011). Still, populations that were subject to less strong climatic fluctuations in the Pleistocene show higher genetic diversity than those in more climatically variable areas (Peng et al. 2015).

#### Conservation status.

The EOO for *Polylepistarapacana* is estimated as 127,498 km^2^, the AOO is assessed at 448 km^2^ and it is known from 69 locations. The species was categorized as NT in the World List of Threatened Trees (Oldfield et al. 1998). Based on its restricted distribution in these countries, *P.tarapacana* was categorized as Vulnerable in Chile (Benoit 1989) and Peru (SERFOR 2006). In Bolivia, where it is more widespread, *P.tarapacana* was categorized as VU (B1b(i,ii,iii)) (Arrázola et al. 2012). Main threats for the species are livestock grazing (camelids), grassland burning, firewood collection and farming activities. In Chile, it is also affected by mining activities which are partly compensated for by conservation measures. It is protected within Sajama National Park in Bolivia. We assess *P.tarapacana* as Near Threatened (A1+A2a, B1a+B2a, C1).

#### Notes.

In southern Bolivia, it is often difficult to differentiate between *P.tarapacana* and *P.tomentella*. In fact, Simpson (1979) considered both forms as conspecific, before separating them in a later publication (Simpson 1986). The challenge in distinguishing the two species is posed by a wide transition zone between both taxa, where populations present intermediate characters and do not show typical traits of either species (Simpson 1979; Kessler 1995b). However, throughout most of their distributional ranges, both species are quite distinct, with *P.tarapacana* having shorter leaflets (0.7–0.8 cm vs. 1.3–2.1 cm), entire to very slightly crenate leaflet margin and obtuse to acute apices (vs. serrate margins and round to emarginate apices) and shorter inflorescences (0.7–1.5 cm vs. 2.8–5.3 cm) with 1–2 flowers (vs. 4–5 flowers).

#### Specimens examined.

**Bolivia. La Paz**: Pacajes, Laguna Blanca, 14 km hacia la carretera a Tambo Quemado, 17°39'S, 068°43'W, 4110 m, 20 November 2006, *Beck 29620* (LPB); Santiago de Machaca, 27 km hacia Berenguela, 4130 m, 24 April 1982, *Beck 9008* (GOET!, LPB); ciudad de Piedra, 17°31'35"S, 068°53'17"W, 3888 m, 25 January 2012, *Terán 5195* (BOLV). Jardín Botánico La Paz, from seeds collected at La Paz Sajama 1991, *Kessler 12628* (GOET!). **Oruro**: Atahuallpa, Cerro Villa Pucarani en el salar de Coipasa, 19°19'S, 068°18'W, 4670 m, 04 November 1994, *Beck 21570* (GOET!). *21569* (GOET!, LPB, MO!). Sajama, Volcán Sajama, 29 April 1987, *Arctander s.n* (AAU!); al Norte del pueblo de Sajama, 4300 m, 31 May 1991, *Beck 19897* (LPB); de Turco 3 km hacia Curahua de Carangas, 3880 m, 18 March 1992, *Beck 21044* (GOET!, LPB); 4600 m, 01 August 1989, *Driesch s.n* (Z!); arriba del pueblo sajama, 4600 m, 01 June 1991, *Hensen 2610* (LPB, MO!); localidad Mamaniri proximo a la población de Sajama, 4170 m, 02 April 1991, *Huanca 69* (GOET!, LPB); próximo a la población de Sajama a los pies del Cerro Sajama lado nor-este, 4325 m, 02 April 1991, *Huanca 74* (GOET!); Cerro Sajama exposición nor-oeste, 4570 m, 03 April 1991, *Huanca 80* (GOET!); Cerro Sajama lado Nor-oeste, 4730 m, 03 April 1991, *Huanca 81* (GOET!); Tirata, 30 km W C. de Carangas on road to Sajama, 17°52'S, 068°32'W, 4100 m, 27 July 1991, *Kessler 2777* (GOET!, LPB, MO!); *2778* (AAU!, GOET!, LPB); 2 km W Tambo Colorado on road to Chile, 18°17'S, 069°02'W, 4500 m, 29 September 1991, *Kessler 3284* (GOET!); *3285* (AAU!, GOET!, MO!); *3286* (AAU!, GOET!, LPB); ladera del Río Sururia, 4650–4800 m, 20 April 1979, *Liberman 70* (GOET!, MO!); Nevado Sajama, ca. 240 km SSO La Paz, in gelichteten Bestanden an FuBe des Berges, 4300–4800 m, 19 July 1983, *Menzel s.n* (GOET!); 6 km NE Laguna, foothills of Nevado Sajama, 4200 m, 18°11'26"S, 068°51'13"W, 4200 m, 05 December 1984, *Schmitt 173* (MO!, NY); around bae of Volcano Sajama, 4350 m, 18 October 1967, *Vuilleumier 316* (GH!, US!); 2 km south of the town of Sajama on the road to Tambo Quemado, 05 September 1986, *Zeballos s.n* (MO!). **Potosí**: Enrique Baldivieso, Cerro Chuhuilla on Alota–Lag Hedionda road, 21°29'S, 067°50'W, 4500 m, 13 September 1991, *Kessler 3073*; *3074*; *3075* (AAU!, GOET!); *3076* (GOET!, LPB); ca. 20 km W Alota-Lag Hedionda road, 21°23'S, 067°43'W, 4050 m, 13 September 1991, *Kessler 3078* (GOET!). Quijarro, near pass on río Mulatos–Yura road, 19°43'S, 066°28'W, 4300 m, 11 September 1991, *Kessler 3065* (GOET!); *3066*; *3067* (GOET!, MO!); *3068* (GOET!); al Nor este en linea recta de la población Pulacayo aprox 3 km, 20°22'25"S, 066°41'11"W, 4402 m, 13 March 2010, *Zenteno 9828* (LPB). Sud Chichas, 20 mi E of Atoche and 1.5 mi above Santa Barbara on the southwest face of Nevada Choroloque, 4750 m, 15 March 1993, *Peterson 12947* (AAU!, GOET!). Sud Lipez, Quetana Chico 18 km hacia el Volcán Uturuncu, 22°13'28"S, 067°13'11"W, 4500 m, 26 September 2006, *Beck 32470* (LPB); 32 km E lag Colorada on road to Pena Barrosa, 22°12'S, 062°28'W, 4500 m, 14 September 1991, *Kessler 3083*; *3084* (AAU!, GOET!, LPB); *3429* (GOET!, USM!); *3430*; *3595*; *3596*; *3597*; *3598*; *3599*; *3600*; *3601*; *3602*; *3603*; *3604*; *3605*; *3606*; *3607*; *3608*; *3610*; *3611*; *3612*; *3613*; *3614*; *3615*; *3616*; *3617*; *3618*; *3619*; *3620* (GOET!); Cerro Tapaquillacha, 4600 m, 12 April 1980, *Libermann 171* (GOET!, LPB); 2.5 mi S of Yuray (Nuevo) San Antonio de Lipez on road towards Quetena Grande, 4300 m, 19 March 1993, *Peterson 13014* (AAU!, GOET!), 26 August 1991, *Killeen 2682*.

**Chile. Parinacota**: Putre, Tarapaca, Parinacota, Zapahuira, pres de puente Murmuntani, 18°15'S, 069°35'W, 3400 m, 05 November 1991, *Billiet 5467* (MO!). **Tarapacá**: Parinacota, Lac Chungara, 18°15'S, 069°10'W, 4580 m, 06 November 1991, *Billiet 5476* (MO!). ca. 5 km below Putre on road to Arica, 3500 m, 29 December 1995, *Landrum 8885* (MO!); *Philippi s.n* (MO!). **Peru. Puno**: Puno, El Collao, Santa Rosa, 16°45'35"S, 069°53'11"W, 4131 m, 09 October 2014, *Boza 3009* (USM!, Z!); Santa Rosa, 50 miles SSW of Llave, 4600 m, 25 July 1946, *Olivera 16* (US!). **Tacna**: Tacna, Cord. Volcán Tacora, Chislluma, 4500 m, 01 April 1926, *Werdermann 1143* (F!, GOET!, MO!, US!, Z!). Tarata, Laguna Casire, en borde y cercanias de la Laguna, 4700–4800 m, 03 April 1998, *La Torre 2389* (MO!, US!, USM!); *2440* (MO!, USM!); Chiluyo Chico, 4270 m, 07 December 1997, *Roque 503* (USM!); *Weddell s.n* (F!).

### ﻿45. Polylepis tomentella

Taxon classificationPlantaeRosalesRosaceae

Wedd., Chlor. Andina 2: 237, pl. 78. 1857 [1861].

575EC76B-E5AC-5847-A215-A34F4BD97BB0

Figs 114 , 115

Polylepis tomentella Wedd. subsp. tomentella M.Kessler (1995b:162). Type: based on Polylepistomentella Wedd.
Polylepis tomentella subsp. pentaphylla Bitter, Bot. Jahrb. Syst. 45: 648. 1911. Type. Argentina. Jujuy, Laguna Tres Cruces, Dec 1900–Feb 1901, *Clarin 11688* (holotype: S!).
Polylepis tomentella subsp. tetragona Bitter, Bot. Jahrb. Syst. 45: 649. 1911. Type. Argentina. Jujuy, Salina Grande nahe Jujuy, 3500 m, *Hauthal 141* (holotype: B destroyed; isotype: GOET!).
Polylepis tomentella subsp. dentatialata Bitter, Bot. Jahrb. Syst. 45: 650. 1911. Type. Bolivia. Potosí, in valle inter Chorolque at Tacna (Quechisla-Kasni), 3600–3800 m, *Hauthal 117* (holotype: B destroyed; isotype: GOET!).
Polylepis tomentella var. pilosior Bitter, Bot. Jahrb. Syst. 45: 647. 1911. Type. Bolivia. Chuquisaca, Cinti, Jan 1846, *Weddell 3947* (holotype: P!).

#### Type.

Bolivia. Chuquisaca, Cinti, Jan 1846, *Weddell 3927* (lectotype, designated by Simpson 1979, pg. 46: P!; isolectotype: P!).

**Figure 114.**
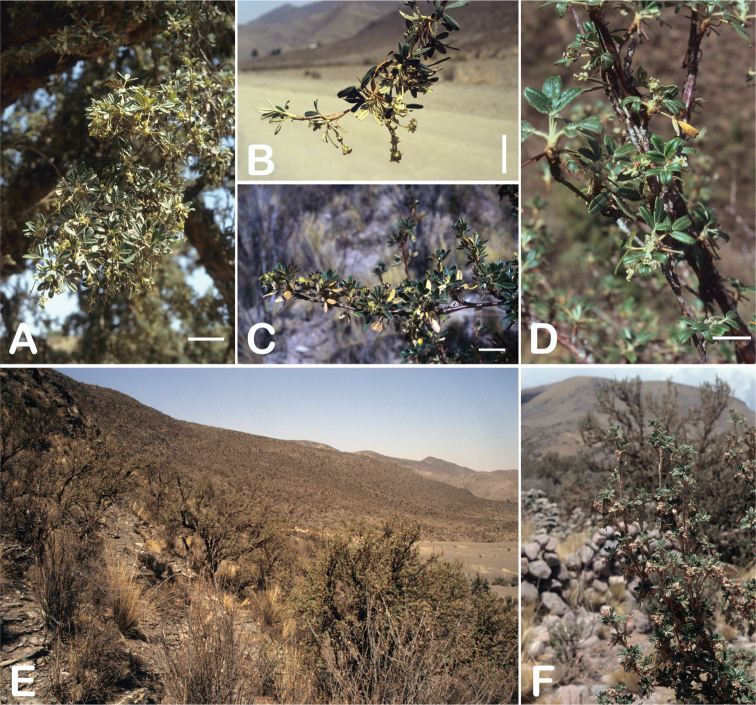
*Polylepistomentella* Wedd **A, B** flowering branches **C, D** leaves **E, F** habit. Scale bars: 3 cm (**A**); 2 cm (**B–D**). Photographs by M. Kessler.

#### Description.

***Trees*** 1.5–5 m tall. ***Leaves*** slightly congested at the branch tips, imparipinnate with one pair of leaflets, obtrullate in outline, 1.2–2.9 × 1.3–2.1 cm; rachises densely villous, points of leaflet attachment with a tuft of long hairs; stipular sheaths apically truncate or slightly spurred, densely villous on the outer surfaces; leaflets obovate in outline, second pair from the terminal leaflet the largest, one of this pair 1.3–2.1 × 0.3–0.6 cm; margin serrate with 5–10 teeth, apically round or emarginate, basally unequally attenuate; upper leaflet surfaces glabrous to sparsely villous; lower leaflet surfaces with a dense layer of very short, white or yellowish pannose hairs. ***Inflorescences*** pendant, 2.8–5.3 cm long, bearing 4–5 flowers; floral bracts 3.9–5.1 mm long, narrowly triangular, densely villous on the outer surface; rachises villous. ***Flowers*** 5.9–6.8 mm diam.; sepals 3, ovate, green, sparsely villous outside; stamens 19–23, anthers orbicular, with a dense tuft of straight white hairs on the upper half; styles fimbriate, 2.4–2.5 mm long. ***Fruits*** turbinate, with 3–4 irregular flattened ridges with a series of spines, densely villous; 6.2–6.9 × 4.2–5.9 mm including spines. ***Tetraploid***.

**Figure 115.**
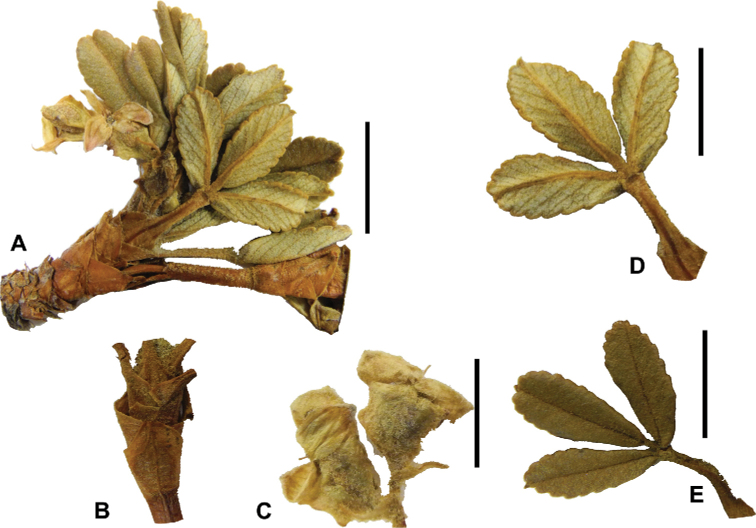
*Polylepistomentella* Wedd **A** flowering branch **B** stipular sheaths **C** fruit **D** lower leaf surface **E** upper leaf surface **E** fruits (**A, D***Schulte 22***B***Kessler 3191***C***Kessler 3366***E***Kessler 3201*). Scale bars: 2 cm (**A, D, E**); 6 mm (**C**). Photographs by T. E. Boza E.

#### Distribution, habitat and ecology.

**Figure 116.**
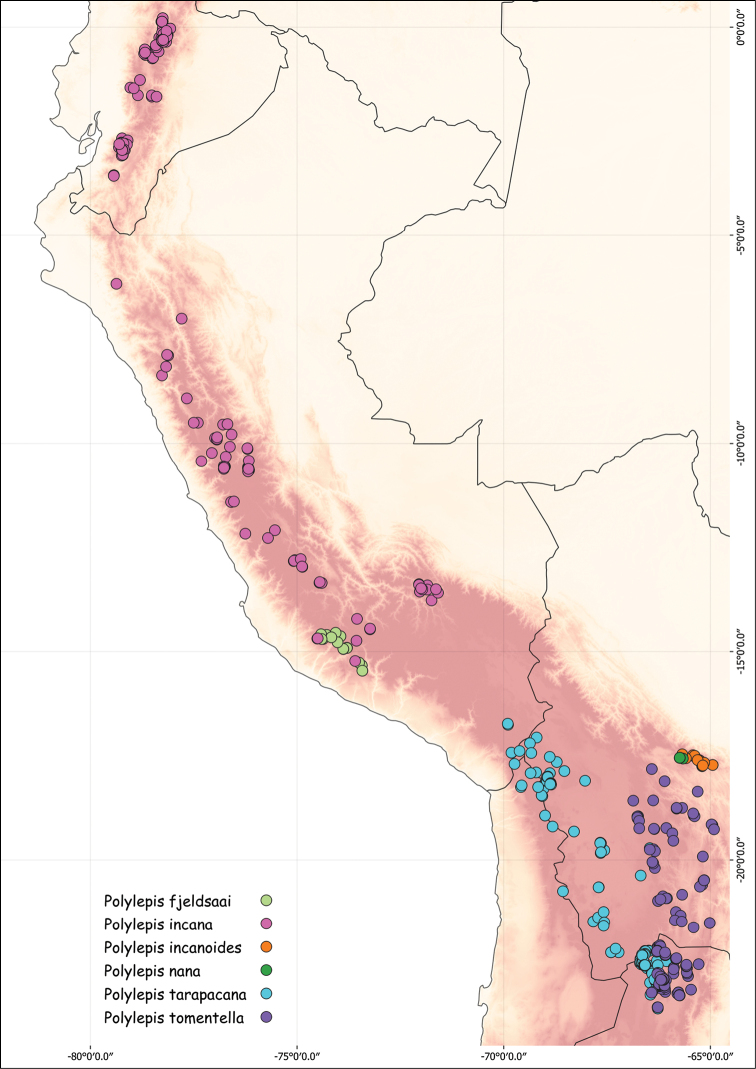
Geographical distribution of the species of subsection Incanaee.

*Polylepistomentella* is distributed in southern Bolivia and northwest Argentina at 2750–4950 m elevation (Fig. 116). The forests of this species are usually homogeneous, but are occasionally mixed with *Dasyphyllumhystrix* and *Buddleiaaromatica* (Navarro et al. 2005). *Polylepistomentella* flowers during the dry season (May–October) and fruits at the beginning of the wet season (August–December) (Domic et al. 2013). Trees produce three times more flowers (44.7 ± 6.4 flowers/branch) and fruits (24 ± 2.7 fruits/branch) than shrubs (15.86 ± 2.18 flowers/branch; 9.3 ± 1.3 fruits/branch) (Domic et al. 2013). Maternal tree size and seed mass have a positive effect of seed germination and survival (Domic et al. 2020). Populations of *P.tomentella* with different levels of human disturbance have also different percentage of saplings and seedings, with lower percentages in strongly disturbed than in moderately disturbed populations, even though reproductive individuals produce twice as many flowers and fruits. This suggests that fruit production does not limit regeneration and post-dispersal mechanisms may decrease seed germination and increase seedling mortality (Domic et al. 2014). *Polylepistomentella* has a high proportion of non-viable seed that are aborted due a variety of reasons, including a fungal infection that might reduce the fecundity of the host tree (Domic et al. 2017). Hybrids between *P.tomentella* and *P.besseri* have been reported in Bolivia (Kessler 1995b).

#### Conservation status.

The EOO for *Polylepistomentella* is estimated as 94,183 km^2^, the AOO is assessed at 436 km^2^, and it is known from 75 locations. The species was categorized as NT in the World List of Threatened Trees (Oldfield et al. 1998). Based on its fragmented habitat affected by human disturbance, it was listed as EN (B2b(i,iii)) in the Red List of Bolivia (Arrázola et al. 2012). No conservation actionshave been taken to date. Specific threats are traditional use for extensive livestock grazing, firewood extraction and additionally in rainy areas, for Andean tuber crops, cereals and fodder. We assess *P.tomentella* as VU (A1, B1a+B2a, C2a).

#### Notes.

*Polylepistomentella* can be distinguished from the most similar species *P.incana* by the leaflet margins (serrate versus crenate), lower leaflet surface (with resinous exudate versus without exudate) and inflorescences with fewer flowers (4–5 versus 5–11). For additional morphological similarities, see under *P.fjeldsaoi*, *P.incanoides*, *P.nana* and *P.tarapacana*.

#### Specimens examined.

**Argentina. Jujuy**: Cochinoca, Río Despensa, 4200 m, 19 January 1971, *Boelcke 7133* (MO!); Humahuaca, Tres Cruces, Cerros, 17 February 1971, *Cabrera 21424* (MO!); Hills 4 km N of Lagunilla on W side of Laguna Pozuelos, 3700 m, 20 November 1991, *Kessler 3357* (AAU!, GOET!, MO!); Hills 4 km N Lagunilla on W side of Laguna Pozuelos, 3700 m, 20 November 1991, *Kessler 3355* (AAU!); *3356* (GOET); *3358* (AAU!, GOET!). **Bolivia. Chuquisaca**: Nor Cinti, Muyuquiri 6 kms hacia Camargo, 3510 m, 23 March 1979, *Beck 686* (LPB, MO!); 30 Km N Camargo on road to Potosí, 20°30'S, 065°10'W, 3200 m, 22 September 1991, *Kessler 3177* (GOET!, MO!); *3178* (AAU!, GOET!, LPB); *3179* (AAU!, GOET!, LPB); *3184* (AAU!, GOET!, MO!); *3185* (GOET!); 32 km N Camargo on road to Potosí, 20°30'S, 065°10'W, 3200 m, 22 September 1991, *Kessler 3186* (GOET!, LPB, MO!); *3187* (AAU!, GOET!, MO!); *3188* (AAU!, GOET!, LPB); Cerro Poqueñita, 4050 m, 10 November 1990, *Serrano 5* (GOET!); Cerro Peticoya, 3500 m, 10 November 1990, *Serrano 6* (GOET!, LPB); along the road between Camargo and San Lucas, 3300 m, 10 December 1967, *Vuilleumier 427* (GH!, US!); Oropeza, Cajamarca ca. 30 km hacia Revelo, 3300 m, 09 October 1984, *Beck 8827* (GOET!, LPB); Comunidad Punilla a 25 km. de la ciudad de Sucre, en la ladera superior del Río Kollpamayu, orientación SW, 18°57'00"S, 065°23'00"W, 3260 m, 29 April 2000, *Gutiérrez 171* (MO!); ca. 35 km W Sucre on road to Macha, 18°53'S, 065°25'W, 3300 m, 26 September 1991, *Kessler 3258* (GOET!); *3259* (AAU!). Tomina, Pass on San Pedro-Keluya road, 19°55'S, 065°11'W, 3450 m, 22 September 1991, *Kessler 3189* (AAU!, GOET!); Pass on San Pedro-Keluya road, 19°55'S, 065°11'W, 3450 m, 22 September 1991, *Kessler 3190* (AAU!, GOET!, LPB); *3191*; *3192* (GOET!, MO!); 5 km W Tarabuco on road to Sucre, 19°09'S, 064°58'W, 3300 m, 22 September 1991, *Kessler 3199* (GOET!, MO!); *3200* (GOET!, MO!); Yamparaez, Tarabuco unos 10 km hacia Surce, 3500 m, 13 October 1984, *Beck 8858* (LPB, MO!); Tarabuco 10 km hacia Sucre, 19°10'S, 064°57'W, 3200 m, 29 October 1983, *Beck 9859* (LPB, MO!); 5 km W Tarabuco on road to Sucre, 19°09'S, 064°58'W, 3300 m, 23 September 1991, *Kessler 3201*; *3202* (AAU!, GOET!); 20 km S Tarabuco on road to Lola, 19°09'S, 064°58'W, 3300 m, 23 September 1991, *Kessler 3207* (AAU!, GOET!); Zudañez, 25 km S Icla on Tarabuco-Azurduy road, 19°27'S, 064°49'W, 3500 m, 24 September 1991, *Kessler 3213* (AAU!, GOET!, LPB); Cinti, Puna, *Weddell 3927* (P!). — **Cochabamba**: Aiquile, Lajamayu, 18°21'31"S, 065°18'29"W, 10 May 1995, *De la Barra 419* (MO!); Carrasco, Siberia, 17°48'11"S, 064°46'12"W, 2900 m, 16 April 2005, *Fernández 3594* (MO!); *3594* (BOLV); Siberia, 17°48'45"S, 064°45'59"W, 2950 m, 17 April 2005, *Fernández 3604* (MO!); *3604* (BOLV); El Churo-Sunchal, ca. de 10 km hacia Pojo, lado del camino carretero Comarapa-Cochabamba, 17°47'51"S, 064°47'24"W, 2780 m, 08 November 2003, *Vargas 7053* (MO!); Tiraque, El Ronco, 17°00'05"S, 065°39'20"W, 3470 m, 10 May 2005, *Alcázar-Johansen 387* (MO!); Andes de Cochabamba, 1826–1833, *Orbigny s.n* (MO!, P!); *Saravia 1154* (BOLV). — **Oruro**: Avaroa, Challapata, 4000 m, 01 April 1921, *Asplund 6169* (US!); Challapata, 4000 m, 01 April 1921, *Asplund 6170* (US!); a pocos km de Challapata, camino a Sevonuyo, 19°14'S, 066°04'W, 3790 m, 06 March 1991, *Huanca 31* (LPB); ca. 70 km W Potosi on road to Oruro, 19°14'S, 066°04'W, 3800 m, 22 November 1991, *Kessler 3366* (GOET!, LPB); *3367* (GOET!, LPB, MO!); *3368* (AAU!); *3369* (AAU!, GOET!, LPB); *3378* (AAU!, GOET!, LPB, MO!); *3379* (GOET!, LPB); 5 km S of Challapata, 18°57'S, 066°45'W, 3700 m, 22 November 1991, *Kessler 3380* (GOET!, LPB, MO!); Challapata, Cordillera Azanaques, 18°56'02"S, 066°43'13"W, 4676 m, 04 March 2006, *Torrico 378* (BOLV, MO!); Poopo, a 6 km de Challapata en dirección a Huari, 3750 m, 26 February 1991, *Navarro 200* (LPB); Pazña, a 1 km este del balneario de Urmiri pasando la ex central eléctrica, 18°34'36"S, 066°51'59"W, 3729 m, 13 March 2006, *Torrico 735* (BOLV, MO!). Sebastían Pagador, 1 km arriba de la tranca del pueblo de Huari, 18°59'S, 066°46'W, 3870 m, 13 December 2000, *Michel 2844* (LPB); 5 mi S of Challapata on road toward Uyuni, 3620 m, 07 March 1993, *Peterson 12723* (AAU!, GOET!); 4 mi E of Urmiri, 3770 m, 08 March 1993, *Peterson 12788* (AAU!, GOET!). — **Potosí**: Chayanta, ca. 25 km E Ocuri on Sucre-Macha road, 18°45'S, 065°41'W, 3600 m, 27 September 1991, *Kessler 3274* (GOET!, LPB); ca. 10 km Ocuri on road to Guadalupe, 18°46'S, 065°49'W, 3700 m, 27 September 1991, *Kessler 3277* (AAU!, GOET!); *3278* (GOET!, MO!); *3279* (AAU!, GOET!, LPB); *3280* (AAU!, GOET!, MO!); *3282* (AAU!, GOET!); *3283* (GOET!, LPB); entre Macha y Yocalla, 3600 m, 27 September 1991, *Torrico 216* (LPB); Nor Chichas, nr. Quechisla on road to Chorolque, 3600 m, 01 December 1931, *Cárdenas 24* (GH!); camino Lique entre Yurtuy, Cancha a la Laguna, 3800 m, 19 November 1987, *Schulte 22* (GOET!, LPB); cerca de Cotagaitilla, desde Cotagaita, al borde del río, 2800 m, 13 May 1993, *Torrico 502* (LPB); Sta. Barbara, on road from Quechisla, 4200 m, 22 February 1936, *West 6110* (GH!, MO!, UC); Quijarro, 32 km de Potosí en camino a Uyuni, 4100 m, s.d., *Liberman 229* (LPB); 4 mi SW of Vilacota on E facing slope above Lago, 3850–4130 m elev., 27 March 1993, *Peterson 13113* (AAU!, GOET!); 9 mi NE of Tica Tica on road towards Potosi, 3800 m elev., 29 March 1993, *Peterson 13132* (AAU!); 6 mi NE of Paratoya on road towards Potosi, 3650 m, 30 March 1993, *Peterson 13147* (AAU!, GOET!); zona W camino hacia Uyuni, Kilpani hacia Landara, 3850 m, 25 March 1993, *Torrico 222*, *223* (LPB); zona W camino hacia Uyuni, Colina antes de llegar a Calasaya, 4000 m, 25 March 1993, *Torrico 233* (LPB); Calasaya, 4000 m, 25 March 1993, *Torrico 234* (LPB); entre Cebadilla y Condoriri, 3860 m, 25 March 1993, *Torrico 256* (LPB); Sud Chichas, Cerro Pabellon 3 km W Atocha, 20°59'S, 066°16'W, 4000 m, 16 September 1991, *Kessler 3085* (AAU!, GOET!, LPB), *3086* (GOET!, MO!), *3087* (AAU!, GOET!, MO!), *3088*, *3089* (AAU!, GOET!, LPB), *3621* (GOET!, MO!), *3622* (GOET!, MO!), *3623* (GOET!, MO!), *3624* (GOET!); ca. 10 km N Salo on Atocha-Tupiza road, 21°15'S, 065°50'W, 4000 m, 16 September 1991, *Kessler 3107* (AAU!, GOET!, LPB); 10.5 mi N of San Vicente on road towards Atocha, 3930 m, 17 March 1993, *Peterson 12972* (AAU!, GOET!); Vilquiza, a 13 km de Tupiza, 3320 m, 25 March 1993, *Torrico 436* (LPB); Mina Isca-Isca above the village of La Torre north of Tupiza, 3875–3880 m, 02 December 1967, *Vuilleumier 402* (GH!, MO!); Queñua Pampa, al Este-Sur-Este en linea recta de Suripa aprox. 12.45 km, 21°37'21"S, 065°24'26"W, 3765 m, 05 March 2012, *Zenteno 11825* (LPB); arriba del Mirador de Boris al Oeste en linea recta de la ciudad de Tupiza approx. 14–15 km, 21°27'10"S, 065°51'29"W, 4009 m, 10 March 2012, *Zenteno 11974* (LPB); Santa Rosa al sur-sur-oeste en línea recta de la ciudad de Tupiza aprox. 5.67 km, 21°29'02"S, 065°41'35"W, 3439 m, 11 March 2012, *Zenteno 12043* (LPB); Tomas Frias, Challapata 124 km hacia Potosí, 4080 m, *Beck 674* (LPB); cerca de Ventilla, 4030 m, 17 February 1979, *Ceballos 255* (G); Orocoro, bajando hacia Yocalla, 3850 m, 17 February 1979, *Ceballos 273* (G); Thunarumi, próximo a la localidad Paco-grande, 3650 m, 19 April 1991, *Huanca 100* (GOET!, LPB); Thunarumi, próximo a la localidad Paco-grande, 3650 m, 19 April 1991, *Huanca 98* (LPB); 23 mi SW of Potosi, 4220 m, 30 March 1993, *Peterson 13150* (AAU!, GOET!); 27.7 km SE of Cruce Ventilla on road from Oruro to Potosi, 19°15'S, 066°22'W, 4000 m, 12 May 1983, *Solomon 10651* (LPB, MO!); 39 km northwest of Potosí on the road to Challapata, 3750 m, 14 December 1967, *Vuilleumier 450* (GH!, US!); 3500 m, 30 October 1983, *Beck 9860* (CUZ!, GOET!, LPB, MO!, QAME, TEX). — **Santa Cruz**: Vallegrande, A 19.5 km de Vallegrande camino a Pucara, 18°35'23"S, 064°07'23"W, 2721 m, 25 September 2011, *Arroyo 5821* (MO!, USZ). — **Tarija**: Avilez, Reserva Biológica Cordillera de Sama, Cordillera alta de Sama, 21°41'28"S, 065°08'56"W, 3900 m, 27 February 2007, *Zárate 2429* (BOLV, MO!); Mendez, Iscayachi, 5 km S turnoff to Tarija from Villazon–Camargo road, 21°31'S, 065°01'W, 3600 m, 17 September 1991, *Kessler 3094* (AAU!, GOET!); Iscayachi, 5 km S of turnoff to Tarija from Villazon-Camargo road, 21°31'S, 065°01'W, 3600 m, 17 September 1991, *Kessler 3095* (GOET!, LPB, MO!), *3096* (AAU!, GOET!), *3097*, *3098* (AAU!, GOET!, LPB). **Chile.***Besser s.n* (MO!).

##### Hybrids

###### *Polylepisincana* × *P.lanuginosa*

**Ecuador. Azuay**: área recreacional Cajas, 02°49'S, 79°07'W, 3740–4070 m, *Romoleroux 1192* (QCA!); Páramo de las Cajas, W of pass, 02°46'S, 79°15'W, 3500 m, 27 August 1985, *Lægaard 55043* (AAU!).

###### *Polylepisincana* × *P.pauta*

**Ecuador. Imbabura**: Páramo de Mojanda, at Laguna Grande, 00°08'N, 78°16'W, 3700–3800 m, 10 November 1984, *Lægaard 52349, 53333, 55661A* (AAU!); same locality as preceeding, 00°08'N, 78°16'W, 3725–3750 m, 30 June 1988, *Romoleroux 652, 661, 662, 670*, *673* (QCA!); Tomauco, 3309 m, 05 June 2008, *Salgado 427* (QCA!); Otovalo to Laguna Mojanda, 00°10'N, 78°20'W, 3770 m, 22 May 1989, *Smith 1992* (QCA!). — **Napo**: road Pifo-Papallacta, 3 km W of Paso de la Virgen, 00°18'S, 78°14'W, 3700–3900 m, 7 August 1987, *Lægaard 54877, 54900, 54902B, 54902C, 54902D, 54902I, 54902J, 54902L, 54902N, 54902O, 54902Q, 54902R, 54902T, 54902Z, 54903, 54904* (AAU!, QCA!); km 18 along the new road, 3700 m, *Lægaard 102310, 102321* (QCA!); Quijos, Oyacachi, 19.5 km al SE Cangahua Páramo, 3800 m, 00°04'S, 78°09'W, 30 June 2003, *Zambrano G147* (QCA!). — **Pichincha**: road El Chaupi-Illiniza, 3200–3700 m, 00°37'S, 78°40'W, 20 June 1985, *Lægaard 54546* (QCA!).

###### *Polylepisincana* × *P.ochreata*

**Ecuador. Imbabura**: Páramo de Mojanda, at Laguna Grande, 3725–3750 m, *Lægaard 52358, 53330, 53331, 53332, 53335, 53337, 54338, 54342, 54344, 54345, 54347, 54559, 54589, 55662* (AAU!, QCA!); Laguna Grande, 00°08'N, 78°16'W, 3725–3750 m, 30 June 1988, *Romoleroux 655, 656, 660, 664, 665, 666, 668, 669, 671, 672, 673* (AAU!, QCA, 3500–3700 m, 2 November 1987, *Romoleroux 478* (AAU!, NY, QCA!); road from Otovalo to Laguna Mojanda, 00°10'N, 078°20'W–00°15'N, 078°20'W, 3700 m, 22 May 1989, *Smith 1992* (MO!). — **Napo**: along road Pifo-Papallacta, E of El Paso de la Virgen, 3750–3850 m, *Lægaard 54559E* (AAU!); 3 km E of Paso de la Virgen, 3950–4050 m, 2 June 1985, *Lægaard 54445* (QCA!), 3–5 km E of Paso de La Virgen, 00°21'S, 78°11'W, 3700–3900 m, 9 June 1992, *Lægaard 103112* (QCA!).

###### *Polylepisincana* × *P.reticulata*

**Ecuador. Azuay**: Páramo de Soldados, 3700–4000 m, *Lægaard 55098A* (AAU!). — **Pichincha**: road El Chaupi-Illiniza, 3200–3700 m, *Lægaard 54546, 54557* (AAU!, QCA!).

###### *Polylepisincana* × *P.racemosa*

**Ecuador. Azuay**: Área Recreacional Las Cajas, 02°49'S, 79°07'W, 3740–4070 m, *Romoleroux 1192* (QCA!). — **Imbabura**: Laguna Grande de Mojanda, 00°08'N, 78°16'W, 3725–3750 m, 30 June 1988, *Romoleroux 651, 653, 661, 663* (QCA!)

###### *Polylepislaniginosa* × *P.reticulata*

**Ecuador. Cañar**: Oeste de Cañar, km 10.5, Cerro Caucay, 3450 m, 27 April 1988, *Romolerous 588* (QCA!).

###### *Polylepispauta* × *P.ochreata*

**Ecuador. Imbabura**: Laguna Grande de Mojanda, 3725–3800 m, 14 May 1985, *Lægaard 52360, 52367A, 54316, 54331, 54334, 54335, 54343, 54345* (AAU!, QCA!); 30 June 1988, *Romoleroux 650, 657, 658, 659, 667* (AAU!, QCA!); Mojanda alrededores de Laguna Grande, 3700–3800 m, 24 May 1987, *Romoleorux 339* (QCA!); camino hacia la laguna Mojanda, 3000–3500 m, 2 November 1987, *Romoleroux 476* (AAU!, QCA!); Laguna Mojanda en los alrededores de la orilla, 3770 m, 21 September 2000, *Lizarzaburu 21* (QCA!). — **Pichincha**: along drinking-water canal on W side of Cerro Atacazo, 3750 m, *Lægaard 53261* (AAU!). — **Napo**: Páramo de Guamani, about 4 km E of the pass on road to Papallacta, 3900 m, *Sparre 15029* (S); N of Cerro Puntas, 3850–3900 m, *Lægaard 54756G, 54757, 54758, 54759* (AAU!).

###### *Polylepissubsericans* × *P.rodolfovasquezii*

**Peru. Cusco**: Urubamba. ACP Mantanay 10 km in the valley from Yanahuara, by the side of Laguna Manalloqsa, in the small valley 3 km of Laguna Ipsacocha, 13°12'01"S, 072°08'47"W, 4627 m elev., 3 February 2011, *Sylvester 556, 568, 595* (CUZ!, Z!).

###### *Polylepisracemosa* × *P.incana*

**Peru. Ancash**: Mariscal Luzuriaga, en vivero forestal de Yanama, procedencia: Casca, 15 December 1989, *Arce 185* (MO!). — **Cajamarca**: Cajamarca to Bambamarca, side road to Chugur (95 km from Cajamarca), 20 March 1988, *Renvoize 4847* (AAU!). — **Cusco**: Cusco, Chacan, 13°29.38'S, 071°59.32'W, 3782 m, 07 November 2004, *Farfán 543* (CUZ!, F!, MO!, USM!); Acomayo, Rondocan, localidad Huacuy 13°46'21"S, 71°44'46"W, 3842 m, *Pfuro 130* (Z!); Urubamba, Trail behind Chincheros centre, past Antasaka rock to spring called Parqo, first kilometre out of the populated area, 13°24'S, 072°03'W, 3810 m, 12 January 1982, *Davis 1359* (A!, USM!); Chincheros, quebrada above Pojpoj waterfall, 13°23'S, 072°03'W, 3450–3550 m, 14 January 1982, *Davis 1466* (A!, USM!). — **La Libertad**: above Cachicadan; in quebradas, forming a grove, only large tree in quebradas towards top of hills north of Cachicadan, 2900 m, 25 November 1938, *Stork 9972* (F!). — **Lima**: Oyon, Laguna Guengue Grande, Quichas, 4215 m, 11 January 1990, *Arce 189* (MO!).

###### *Polylepisneglecta* × *P.subtusalbida*

**Bolivia. Potosi**: Bilbao, antes de Sakani Khasa, 3400 m, 18 March 1993, *Torrico 199* (LPB!); 31 km SW Acacio on road to Sacaca and Uncia, l8°06'S, 66°08'W, 3500 m, 22 August 1991, *Kessler 3417* (AAU!, GOET!, LPB!, USM!), *Kessler 3555* (GOET!).

###### *Polylepistriacontandra* × *P.incarum*

**Bolivia. La Paz**: Camacho, Puerto Acosta, 3680 m, 5 April 1982, *Beck 7662* (GOET!, LPB!); Puerto Acosta, Estancia Kerojani, 3900 m, 26 January 1980, *Jordan 51* (LPB!); Murillo, Palca 28.5 km hacia Cohoni, 3440 m, 14 October 1990, *Beck 17832* (GOET!, LPB!), río Minasa, 1.5 km arriba del viejo puente del ferrocarril (ca. 3 km arriba de Villa Fatima), l6°27'S, 68°07'W, 4000 m, 18 January 1987, *Solomon 15783* (LPB!, MO!, NY).

###### *Polylepistriacontandra* × *P.lanata*

**Bolivia. Cochabamba**: Ayopaya, 10 NW de Independencia, alredor de la Cima de la Loma, 3250 m, *Beck 14528* (GOET!, LPB). — **La Paz**: 8 km W Quime on road to Caxata, 17°03'00"S, 067°17'00"W, 3350 m, 24 August 1991, *Kessler 3028* (GOET!).

###### *Polylepislanata* × *P.subtusalbida*

**Bolivia. Cochabamba**: Ayopaya, cerca de Morochata, 3300 m, 1 August 1991, *Hensen 872* (LPB!); Carrasco, Zapata Rancho, 3200 m, 22 November 1989, *Hensen 945* (LPB!); Mojón, 3300 m, 19 March 1991, *Hensen 1765* (GOET!, LPB!).

###### *Polylepisbesseri* × *P.tomentella*

**Bolivia. Chuquisaca**: Zudafiez, 25 km S Icla on Tarabuco-Azurduy road, l9°27'S, 64°49'W, 3500 m, 24 September 1991, *Kessler 3209* (AAU!, GOET!, LPB!), *3211* (GOET!, LPB!).

###### *Polylepisbesseri* × *P.incanoides*

**Bolivia. Cochabamba**: Arani, 49.3 km E of bridge over the río Pucara (at Punata) on the road to Sta. Cruz, l7°26'S, 65°29'W, 3100–3300 m, 20 October 1985, *Solomon 14452* (NY, LPB!); Vacas (village Rodeo W of Vacas?), 3650 m, 17 April 1987, *Arctander s.n* (AAU!).

###### *Polylepissubtusalbida* × *P.incaniodes*

**Bolivia. Cochabamba**: Arani, 5 km E Mojón on Cochabamba–Comarapa road, l7°30'S, 65°24'W, 3000 m, 5 October 1991, *Kessler 3287* (AAU!, GOET!, LPB!); Carrasco, 6.6 km by road NW of Lopez Mendoza, at km 198 from Cochabamba, Q. Majón, 3250 m, 15 May 1984, *Schmitt 108* (MO!); Yana Qhara, 3300 m, 14 April 1991, *Hensen 2463* (LPB); Llutu Pampa, 3300 m, 14 April 1991, *Hensen 2489* (LPB!); 8 km E Epizana on Cochabamba–Comarapa road, 17°41'S, 65°04'W, 3000 m, 5 October 1991, *Kessler 3291* (GOET!); Rodeo Grande, at km 140 on road from Cochabamba to Santa Cruz, 3000 m, 9 February 1971, *Hawkes 4400* (MO!); a unos 97 km de la capital en dirección a Sta. Cruz, 3650 m, 25 December 1982, *Casas 7746* (NY).

## Supplementary Material

XML Treatment for Polylepis

XML Treatment for Polylepis sect. Sericeaee

XML Treatment for Lanuginosae

XML Treatment for Polylepis lanuginosa

XML Treatment for Polylepis multijuga

XML Treatment for Pauta

XML Treatment for Polylepis longipilosa

XML Treatment for Polylepis pauta

XML Treatment for Polylepis serrata

XML Treatment for Sericeae

XML Treatment for Polylepis albicans

XML Treatment for Polylepis argentea

XML Treatment for Polylepis canoi

XML Treatment for Polylepis frontinensis

XML Treatment for Polylepis humboldtii

XML Treatment for Polylepis loxensis

XML Treatment for Polylepis ochreata

XML Treatment for Polylepis sericea

XML Treatment for Pepea

XML Treatment for Polylepis pepei

XML Treatment for Polylepis rodolfovasquezii

XML Treatment for Polylepis section Reticulatae

XML Treatment for Polylepis hieronymi

XML Treatment for Polylepis microphylla

XML Treatment for Polylepis occidentalis

XML Treatment for Polylepis quadrijuga

XML Treatment for Polylepis reticulata

XML Treatment for Polylepis simpsoniae

XML Treatment for Polylepis weberbaueri

XML Treatment for Australes

XML Treatment for Polylepis australis

XML Treatment for Polylepis neglecta

XML Treatment for Subsericantes

XML Treatment for Polylepis flavipila

XML Treatment for Polylepis pilosissima

XML Treatment for Polylepis subsericans

XML Treatment for Polylepis section Incanaee

XML Treatment for Racemosae

XML Treatment for Polylepis acomayensis

XML Treatment for Polylepis incarum

XML Treatment for Polylepis lanata

XML Treatment for Polylepis pacensis

XML Treatment for Polylepis racemosa

XML Treatment for Polylepis sacra

XML Treatment for Polylepis triacontandra

XML Treatment for Besseria

XML Treatment for Polylepis besseri

XML Treatment for Polylepis crista-galli

XML Treatment for Polylepis pallidistigma

XML Treatment for Polylepis rugulosa

XML Treatment for Polylepis subtusalbida

XML Treatment for Incanaee

XML Treatment for Polylepis fjeldsaoi

XML Treatment for Polylepis incana

XML Treatment for Polylepis incanoides

XML Treatment for Polylepis nana

XML Treatment for Polylepis tarapacana

XML Treatment for Polylepis tomentella
